# Photoinduced Chloroamination Cyclization Cascade with *N*-Chlorosuccinimide: From *N*-(Allenyl)sulfonylamides to 2-(1-Chlorovinyl)pyrrolidines

**DOI:** 10.1021/acs.joc.2c01963

**Published:** 2022-10-26

**Authors:** Emanuele Azzi, Giovanni Ghigo, Lorenzo Sarasino, Stefano Parisotto, Riccardo Moro, Polyssena Renzi, Annamaria Deagostino

**Affiliations:** Department of Chemistry, University of Torino, Via P. Giuria 7, 10125, Turin, Italy

## Abstract

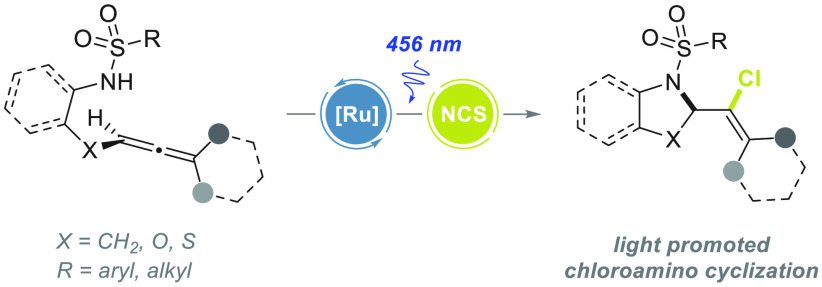

Here, we present an intriguing photoinduced chloroamination cyclization of allenes bearing a tethered sulfonylamido group to afford 2-(1-chlorovinyl)pyrrolidines and related heterocycles in the presence of *N*-chlorosuccinimide (NCS) as the chlorine source. An in depth experimental and computational mechanistic study revealed the existence of multiple reaction pathways leading to a common nitrogen centered radical (NCR). This key NCR can be, in fact, originated from (a) the oxidation of the deprotonated allene by the photoexcited state of the Ru-catalyst and (b) the photodissociation of the in situ formed *N*-chloroallene. The NCR formation triggers an intramolecular cyclization to a highly reactive pyrrolidine vinyl radical, which upon chlorination delivers the final product. Thus, NCS plays a dual role, serving both as an activator of the sulfonamido functionality and as the chlorinating agent.

## Introduction

**Scheme 1:**
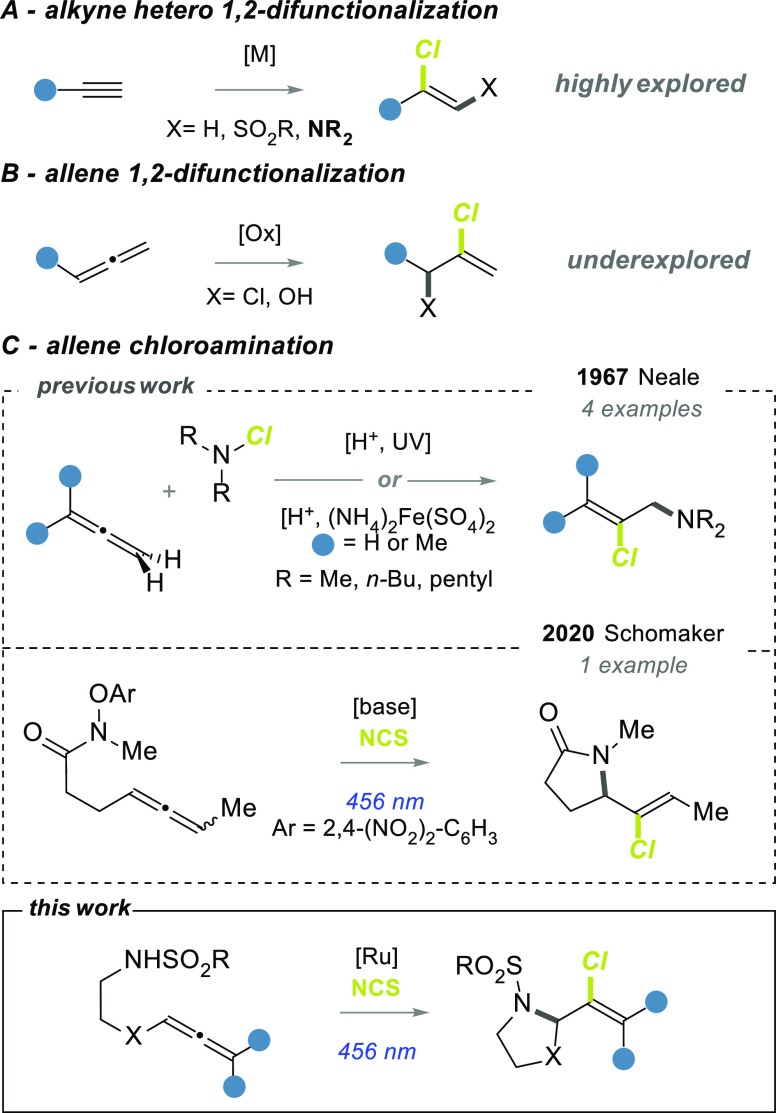
(A,B) General Strategies for the 1,2-Functionalization of Alkynes and Allenes; (C) Chloroamination of Allenes, Previous and Current Approaches

Alkenyl chlorides are ubiquitous across natural products,^1,2^ active pharmaceutical ingredients,^3^ and agrochemicals.^4^ In addition, they are relevant intermediates in the preparation of complex molecules and plastics through transition-metal catalyzed and radical reactions. Compared to heavier halides, only a limited number of known reactions deliver alkenyl chlorides in high yields and stereoselectivity. Such methods usually rely on the functionalization of benchmark chlorinated substrates (e.g., 1,1- and 1,2-dichloroethene) by Suzuki coupling^5^ and olefin metathesis,^6^ or on the copper-catalyzed retro-Finkelstein reaction which requires high temperatures and long reaction times.^7−9^ Other methods, like Hunsdiecker-type transformations, are restricted to α,β-unsaturated compounds.^10−13^ An alternative approach is the chlorination of alkynes and allenes. The strategy presents higher generality and, most importantly, allows the hetero 1,2-difunctionalization, which rapidly increases the molecular complexity of the product and opens to downstream transformations. Alkynes have extensively been used in hydrochlorination,^14−16^ chlorosulfonylation,^17−19^ and chloroamination^20−22^ (Scheme 1A), while the addition of chlorine to allenes is still underexplored (Scheme 1B). Dichlorination was observed using different chlorine donors (TMSCl, oxalyl chloride) and strong oxidizers, like KMnO_4_,^23^ and Selectfluor.^24^ The dichlorination of propadiene was accomplished using Cl_2_ in molten NaAlCl_4_–KAlCl_4_ eutectic at 140 °C.^25^ In 2018, Murphy reported the radical 1,2-dichlorination of aryllallenes in refluxing acetonitrile (CH_3_CN) with a chlorinated hypervalent iodine reagent, producing *E*/*Z* mixtures of vinyl chlorides. Even fewer examples of vicinal hetero-difunctionalizations are reported. Ma developed a regio- and stereoselective chlorohydroxylation of 1,2-allenyl phenylsulfoxides with stoichiometric CuCl_2_ and silica gel under ball milling.^26^ In 2020 Schomaker studied the addition of amidyl radicals to allenes producing *N*-heterocycles (Scheme 1C, middle).^27^ In this report, only in one example was *N*-chlorosuccinimide (NCS) utilized to quench the vinyl radical intermediate, affording the corresponding alkenyl chloride. The sole previous report of allene chloroamination is from 1967, by Neale, who published a study on the radical addition of dialkyl *N*-chloramines to 1,3-dienes, olefins, acetylenes, and allenes (Scheme 1C, top). A mixture of *N*-chloramine and allene in sulfuric and acetic acids generated the corresponding product via a free-radical chain mechanism, by the use of a Fe(II) catalyst or UV irradiation.^28^

Despite being essentially unexplored, the radical intramolecular chloroamination of allenes is an intriguing approach to provide chlorovinyl *N*-heterocycles. Here, we describe a photochemical chloroamino cyclization of allenes bearing a tethered sulfonylamido group to access 2-(1-chlorovinyl)pyrrolidines and related heterocycles (Scheme 1C, bottom). Such an approach allows both access to small saturated heterocycles, the presence of which is widespread in drugs, natural alkaloids, and organocatalysts, and the concomitant installation of a chloroalkenyl moiety, which can be further exploited for the lateral functionalization of the obtained molecules.

## Results and Discussion

Since our previous studies on the reactivity of neutral nitrogen-centered radicals generated from sulfonylhydrazones through photoredox catalysis,^29,30^ we became interested in other precursors of open-shell intermediates suitable for domino cyclization processes. Recently, we investigated the beneficial effect of blue-light irradiation on a Pd(0) catalyzed reaction between aryl bromides and allenyltosyl amides, thus developing a room temperature Heck reaction to access 2-(1-arylvinyl)pyrrolidines and piperidines.^31^ Similar substrates have already been subjected to haloamination with a brominating reagent (LiBr, NBS or 1-bromopyrrolidin-2-one) in combination with transition metal catalysis,^32−34^ or with iodine alone.^35^ As demonstrated by the recent blooming of chlorination protocols exploiting photoredox catalysis,^36^ we envisioned that a light mediated process could have been an ideal approach to tackle this chloroamino cyclization. Therefore, we selected a tosyl protected allenylamine (**1a**) as our model substrate. The feasibility of the reaction was tested reacting *N*-tosylhexa-4,5-dien-1-ylamine **1a** with 1 equiv of NCS in the presence of 5% mol of [Ru(bpy)_3_]Cl_2_ as the photocatalyst, 1 equiv of K_2_CO_3_ as the base in CH_3_CN under irradiation with a 40W blue LED. As shown in Table 1 entry 2, the desired chlorovinylpyrrolidine **2a** was obtained in 33% yield. The design of the reaction condition was inspired by a paper of Leonori and co-workers, which described the NCS-promoted chlorination of primary and secondary amines providing the corresponding aminium NCR.^37^ This latter was generated exploiting the combination of both acidic and photoredox conditions. In our opinion, the substitution of the amine, with a sulfonamide as the starting material and the acid with a base, would unveil a dual role for NCS. Indeed, it could serve both as an initiator of the sulfonamidic nitrogen reactivity and as a chlorinating agent of the double bond. To optimize the reaction conditions, the influence of the solvent, base, and photocatalyst was investigated (Table 1). It should be underlined that, according to the reaction conditions, a certain amount of *N*-chloro*-N*-tosylhexa-4,5-dien-1-ylamine **3a** was formed. Moreover, the tricyclic compound **2a′** was identified as byproduct and never produced with yields higher than 8% (see SI for full screening and characterization). The role of this species will be clarified in the reaction mechanism study. An extensive screening of the photocatalyst was performed, testing both metal complexes and fully organic compounds (see SI). [Acr-Mes]^+^(BF_4_)^−^ was revealed to be less efficient in CH_3_CN than [Ru(bpy)_3_]Cl_2_ affording pyrrolidine **2a** in 29% (Table 1, entry 3). It must be noticed that in this case the *N*-chlorinated allene **3a** was obtained in 14% yield. As shown in Table 1, entry 4, the usage of [Ru(bpy)_3_](PF_6_)_2_ was beneficial for the reaction outcome, causing an increase in yield up to 43%. Probably, this can be ascribed to the counterion PF_6_^–^, which increases the catalyst solubility.

**Table 1:**
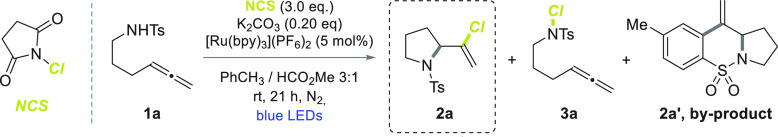
Screening and Optimization of the Reaction Conditions^a^

entry	deviation	**2a** [%]^b^	**3a** [%]^c^	byproduct [%]^c^
**1**	**none**	**56**	**0**	**4**
**2**	[Ru(bpy)_3_]Cl_2_ (5% mol), K_2_CO_3_ (1.0 equiv) in CH_3_CN	33	0	2
**3**	[Acr-Mes]^+^(BF_4_)^−^ (5% mol), K_2_CO_3_ (1.0 equiv) in CH_3_CN	29	14	<1
**4**	[Ru(bpy)_3_](PF_6_)_2_ (5% mol), K_2_CO_3_ (1.0 equiv) in CH_3_CN	43	0	4
**5**	KOH (1.0 equiv) in CH_3_CN	29	0	1
**6**	Cs_2_CO_3_ (1.0 equiv) in CH_3_CN	27	0	3
**7**	2,6-lutidine (1.0 equiv) in CH_3_CN	10	3	<1
**8**	K_2_CO_3_ (1.0 equiv) in PhCH_3_	47	0	5
**9**	K_2_CO_3_ (1.0 equiv) in CHCl_3_ or DMF	28	0	2/not obs
**10**	K_2_CO_3_ (1.0 equiv) in acetone	43	0	2
**11**	K_2_CO_3_ (1.0 equiv) in HCO_2_Me	31	0	4
**12**	K_2_CO_3_ (1.0 equiv) in PhCH_3_:CH_3_CN 3:1	46	0	4
**13**	K_2_CO_3_ (0.5 equiv)	52	0	4
**14**	NCS (2 equiv)	45	6	6
**15**	NCS (0.5 equiv)	10	0	4
**16**	no base or NCS	0	0	0
**17**	no irradiation	0	82	0
**18**	no catalyst	11	66	<1

a Reaction conditions: **1a** (0.20 mmol), base, and catalyst as indicated, anhydrous solvent(s) (4 mL total) under irradiation with 456 nm Kessil blue LED. See SI for the complete screening.

b Yield determined on isolated products.

c Yield determined after 2 repetitions with ^1^H NMR using dichloroethane and nitromethane as internal standards.

Subsequently, the effect of the base was studied. Neither a stronger base such KOH nor organic bases such 2,6-lutidine resulted beneficial for the reaction outcome. In all cases yields below 30% were observed (Table 1, entries 5 and 7, see also SI). Also the change of the cation was not advantageous; for example, Cs_2_CO_3_ produced the chlorivinylpirrolidine **2a** in only 27% (Table 1, entry 6). When the solvent was studied, only the apolar toluene demonstrated to be slightly more efficient than CH_3_CN raising the yield to 47% (Table 1, entry 8). Acetone performed as CH_3_CN (43%, Table 1, entry 10), whereas CHCl_3_ (28%, entry 9), DMF (28%, entry 9), and methyl formate (31%, entry 11) afforded the vinylpirrolidine **2a** in lower yields. Anyway, the apolar nature of toluene did not allow the catalyst to be completely solubilized, so different solvent mixtures were tested to increase the polarity of the reaction medium and, at the same time, to exploit the advantages offered by the use of toluene. So, a mixture of toluene/CH_3_CN 3:1 was tested at first and a 46% yield of **2a** was observed (Table 1, entry 12). On the contrary, the use of toluene/methylformate in ratio 3:1 ensured the recovery of product **2a** in 56% yield (Table 1, entry 15). Interestingly, when the equivalents of the base were reduced to a substoichiometric amount, we observed comparable yields; in fact, pyrrolidine **2a** was obtained in 52% when 0.5 equiv of K_2_CO_3_ were utilized (Table 1, entry 13) and 56% in the case of 0.2 equiv (Table 1, entry 1). The amount of NCS had a central role in the reaction performance, and an excess was always required. A decrease in yield was, in fact, observed reducing the equivalents of NCS from 3 to 2 (56% versus 45% yield; Table 1, entries 1 and 14). An additional loss in yield up to 10% was obtained with 0.5 equiv of NCS (Table 1, entry 15). Also, control experiments were accomplished. As reported in entry 16 of Table 1, the reaction needs both the base and NCS to afford the product. Interestingly, the absence of NCS also impeded the formation of any not-halogenated cyclization product, thus confirming the crucial role of such a chlorinating agent as the initiator of the process. In the absence of the photocatalyst, the chlorinated amide **3a** was recovered as the major product in 66% yield, whereas the desired pyrrolidine **2a** was obtained in only 11% yield (Table 1, entry 18). Finally, when the reaction was conducted without irradiation, the desired product was not observed. Surprisingly, a total conversion of the starting material to the chlorinated derivative **3a** as the unique product occurred in remarkable yield (82%, Table 1, entry 17). Therefore, the standard reaction conditions, described in entry 1, were considered as the best and were applied for the following studies. These reaction conditions included 5 mol % of [Ru(bpy)_3_](PF_6_)_2_, 0.2 equiv of K_2_CO_3_, 3 equiv of NCS in dry toluene/methylformate 3:1 under irradiation at 456 nm.

### Scope of the Reaction

**Scheme 2:**
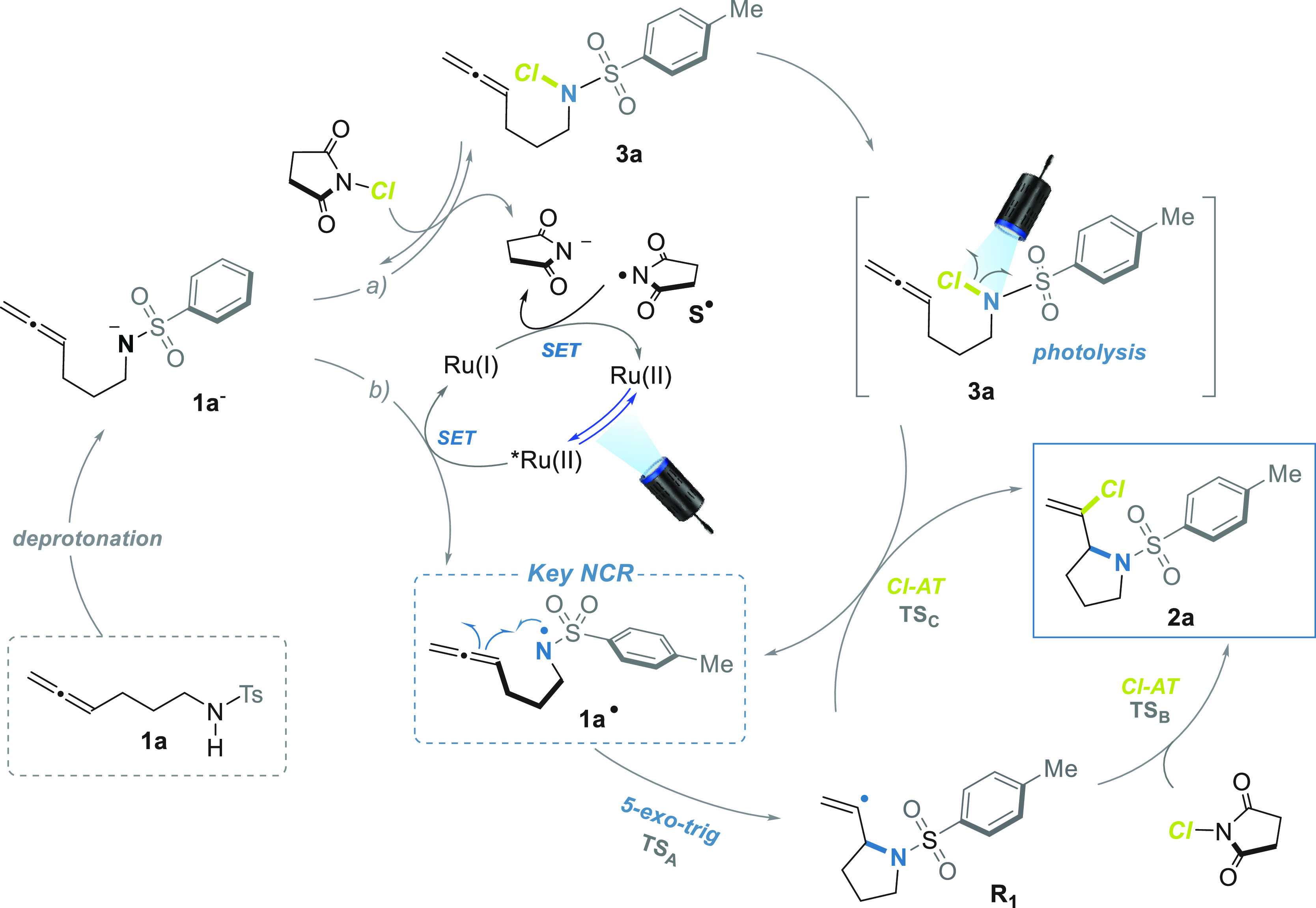
Summary of the Reaction Mechanism

Once the best conditions were determined, the scope of the reaction was explored, and the results are reported in Table 2. First, the influence of variations of the sulfonyl moiety were investigated. Both aryl- and alkylsulfonyl allenes were prepared following three different strategies (Mitsunobu reaction followed by a Crabbè–Ma homologation,^38,39^ Johnson–Claisen rearrangement,^38^ or the isomerization of propargyl(thio)ether).^40^ Those substrates afforded chlorovinyl pyrrolidines **2a**–**i** from moderate to good yields without significant differences between aromatic and aliphatic sulfonyl substitution. Indeed, a similar outcome was observed with mesyl compound **2h** (50% yield) and for the aromatic derivatives **2a**, **2b**, and **2c**, which were obtained in 56, 52, and 51%, respectively. Electron-poor arylsulfonyl allenes seemed to be slightly favored when compared to electron-rich derivatives. See, for example, *p*-methoxyphenyl (**2f**) and 2,3-dihydrobenzo[*b*][1,4]dioxane (**2g**) derivatives, which were recovered in 40% yield, whereas when the *p*-acetyl group was introduced on the phenyl ring, a yield of 50% for product **2e** was observed. Also, the presence of a chlorine in *para* position (**2d**) resulted in 40% yield. It must be noticed that both chloro and acetyl substituents were tolerated in reaction conditions. Finally, a decrease in yield was observed, when sterically hindered aliphatic (+)-camphor sulfonyl allene **1i** was utilized as the starting material. The resulting diastereoisomeric mixture was recovered in 32% yield and diastereomeric ratio of 1:1. Next, internal allenes were explored as starting materials. A decrease in yield was observed with the increase of the number of substituents on the double bond in the corresponding chlorovinyl pyrrolidines. Actually, trisubstituted olefins **2k**, **2l**, and **2m** were obtained in 48, 44, and 54% yield, respectively, superior to those of tetrasubstituted **2n**–**q** (from 28 to 32% yield, see Scheme 2). Notably, the nature of the substituents, whether aromatic or aliphatic, seemed not to influence the reaction outcome.

**Table 2:**
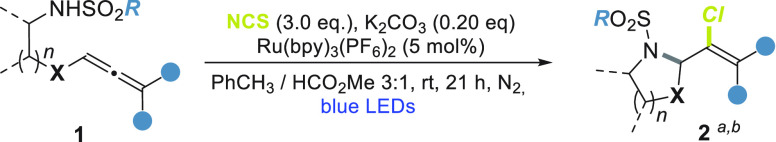

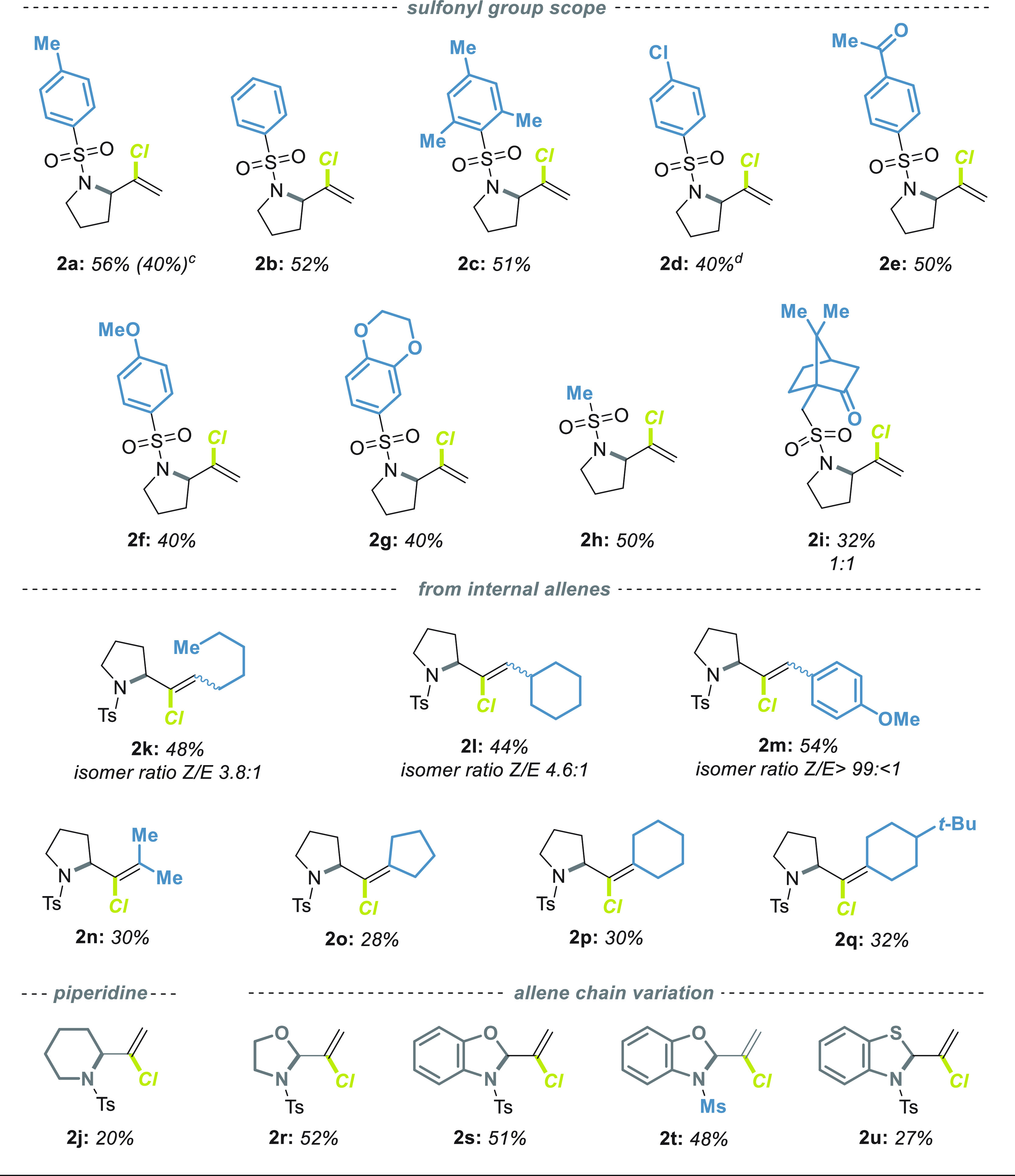
Scope of the Reaction^a^

a Standard reaction conditions: (a) **1** (0.20 mmol), K_2_CO_3_ (0.20 equiv, 0.04 mmol), NCS (3.0 equiv, 0.60 mmol), [Ru(bpy)_3_](PF_6_)_2_ (5 mol %, 0.01 mmol), anhydrous PhCH_3_ (3 mL), anhydrous HCO_2_CH_3_ (1 mL), N_2_ atmosphere irradiation with 456 nm light source for 21 h, blue light. (b) Yields determined on isolated products. (c) Reaction scaled up to 1 mmol, 36 h reaction time. (d) 36 h reaction time.

Finally, the variations in the chain moiety bearing the allene substituents were contemplated, aiming for a change in the nature of the produced heterocycle. With a seven carbon chain, we observed a drop in yield and the resulting piperidine **2j** was produced in only 20% yield, probably because the 1,5-HAT by the NCR might become a competitive process.^41^ Heteroatoms could also be incorporated, and the introduction of an oxygen to afford vinyl oxazoles and hydrogenated vinyl oxazolidines did not result in any significant change in the reaction outcome. Products **2r**, **2s**, and **2t** were obtained in yields comparable to that of analogue vinyl pyrrolidines regardless the substituent of the nitrogen atom (tosyl versus mesyl) and the rigidity of the system (52, 51, and 48%, respectively). The replacement of the oxygen with a sulfur atom to obtain the hydrogenated benzothiazole scaffold resulted instead in a drop in the yield (27% for product **2u**).

### Mechanistic Studies

**Figure 1:**
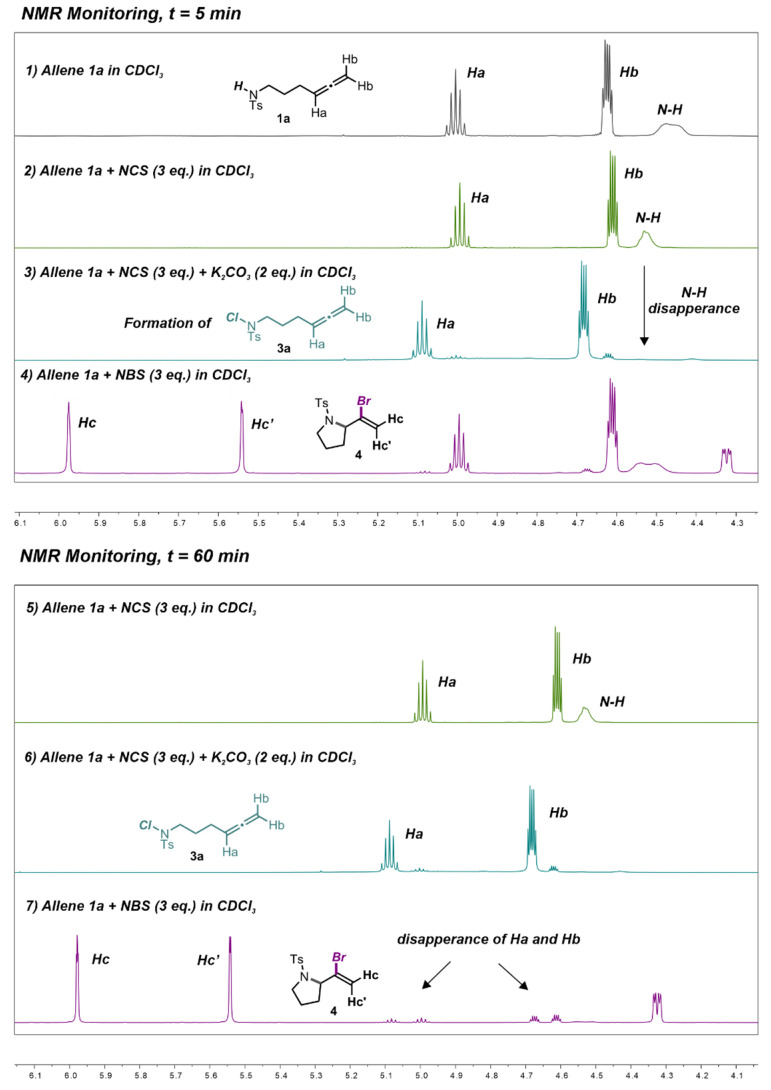
^1^H NMR monitoring of the reaction mixture components prior to irradiation. Top: Spectra recorded after the preparation of the solutions in CDCl_3_ (*t* = 5 min). Bottom: Spectra of the same solutions above recorded after 60 min from preparation. The same solutions were monitored every hour for 6 h.

To attempt an elucidation of the reaction mechanism, several tests were carried out both experimental and computational (see Section S1, Supporting Information—Computational Data (SI-CD), for the details). First, to further confirm the essential role played by the light and to exclude a possible thermal reaction due to the heating provided by the operating light source, the same transformation was realized under thermal conditions (see SI for the complete study). Only when the reaction was carried out at 90 °C, the vinylpyrrolidine **2a** was obtained in traces. Since the highest temperature recorded inside the reaction mixture during irradiation was 45 °C, we excluded the possibility of a thermal mechanism, as proved by the lack of pyrrolidine **2a** formation observed without irradiation at this temperature. In all cases the main product was the chlorinated sulfonamide **3a**. This compound is also the main product recovered in all the control experiments (Table 1, entries 17–18) in the presence of the base. Therefore, in order to clarify the fate of the allene starting material **1a** in the presence of K_2_CO_3_ and NCS, prior to the irradiation, an NMR investigation was conducted adding a component at a time to the allene **1a** in the NMR tube (Figure 1). The studied solutions were prepared in CDCl_3_, given the wide availability of CDCl_3_ as deuterated solvent and considering that a complete conversion of the starting material was obtained also in CHCl_3_ albeit the lower yield in product **2a** (28%, Table 1, entry 9). The evolution of the starting material was studied using both NBS (*N*-bromosuccinimide) and NCS as reactants. Since in our hypothesis, other two main species could be involved in the process together with allenyltosyl amine **1a**, the corresponding Na^+^ salt of allene **1a** (**Na**^**+**^**1a**^**–**^) and *N*-chloro allene **3a** were synthesized to be used as references (for their synthesis and complete characterization, see the SI).

As depicted in the stacked NMR spectra reported in Figure 1, no changes were observed when allene **1a** and NCS were mixed, whereas the concurrent presence of the base and NCS immediately triggered the formation of **3a**. Monitoring the same solution every 60 min for 6 h showed that the chlorinated species **3a** was not able to evolve to the desired product **2a** in the absence of light and catalyst. The same experiment, accomplished using NBS, afforded the corresponding bromo vinylpyrrolidine **4a** in 1 h, and *N*-brominated allene was completely consumed. This confirmed that, in the case of NBS, no irradiation was required, and a thermal pathway was followed. So, a first hypothesis is that in the presence of a base and NCS an equilibrium between the starting allene **1a** and chloro allene **3a** may be established. This has been confirmed by the DFT study (Scheme S1 in the Section S2 SI-CD). The deprotonation by K_2_CO_3_ is thermodynamically favored (Δ*G* = −5.1 kcal mol^–1^, Table S1a in the SI-CD) and yields the potassium salt allene **K**^**+**^**1a**^**–**^. This intermediate can rapidly react with NCS (Δ*G*^‡^ = 3.7 kcal mol^–1^, Figure S1, left) to generate the *N*-chloroallene **3a** and potassium succinimmide **K**^**+**^**S**^**–**^ (Table S1b). This reaction is isoergonic (Δ*G* = 0.04 kcal mol^–1^). On the contrary, the reaction of allene **1a** with NCS to form **3a** and the **K**^**+**^**S**, although thermodynamically feasible (Δ*G* = +0.7 kcal mol^–1^), is kinetically forbidden because its activation free energy barrier is very large (79.3 kcal mol^–1^, Figure S1, right, Table S1c).

**Figure 2:**
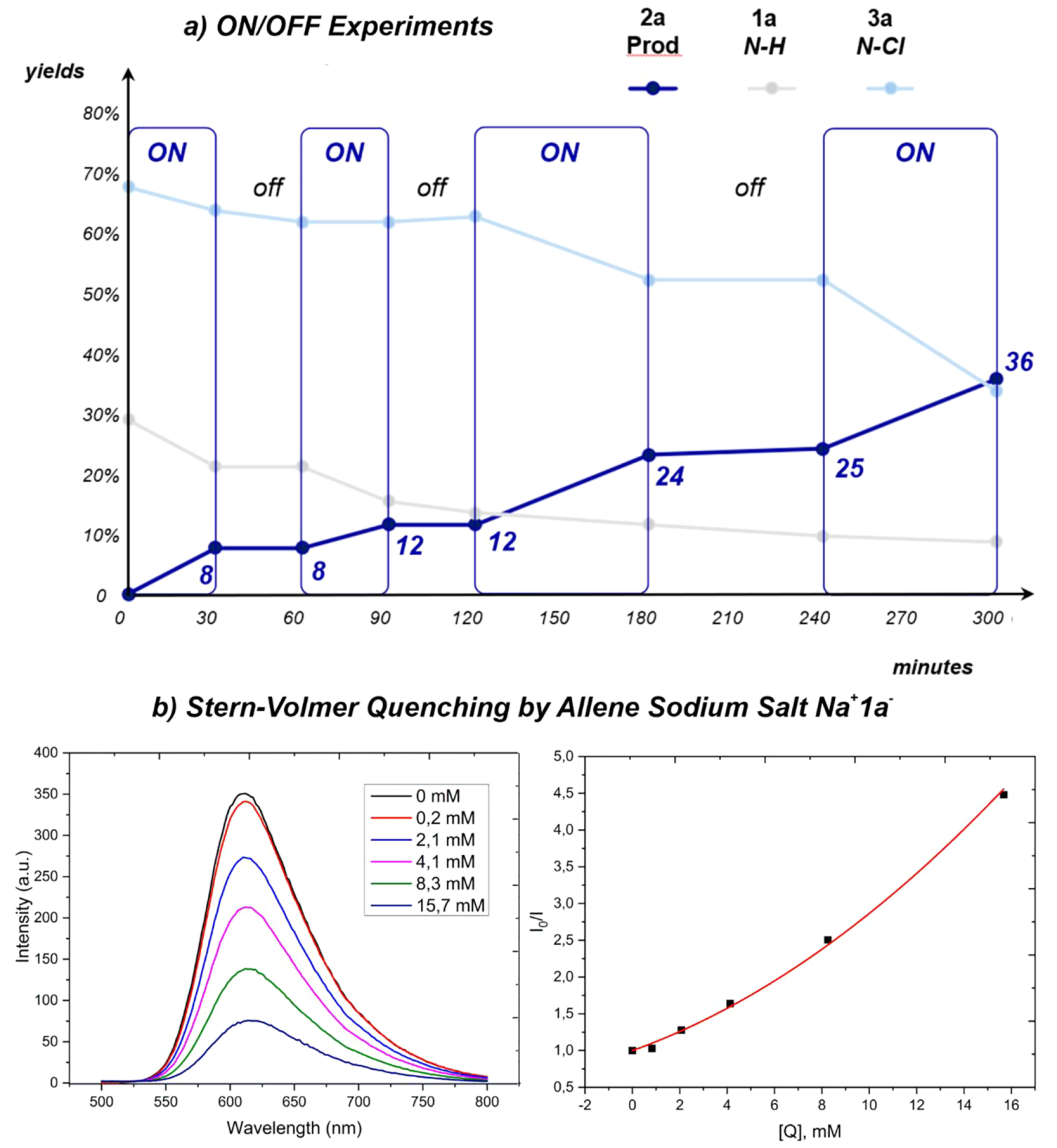
(a) The ON/OFF experiments: blue line, product **2a**; gray line, starting reagent allene **1a**; light blue line, chlorinated sulfonamide **3a**. (b) Fluorescence quenching experiments for [Ru(bpy)_3_](PF_6_)_2_. Left: Fluorescence emission with allene sodium salt **1a**^**–**^**Na**^**+**^ as the quencher. Right: Stern–Volmer plot for the quenching of [Ru(bpy)_3_](PF_6_)_2_ by **1a**^**–**^**Na**^**+**^.

In order to confirm the role of the visible light, ON/OFF experiments were performed (Figure 2a). As already suggested by previous investigations, we observed that a mixture of allene **1a** and *N*-chlorinated allene **3a** was found in 30:70 ratio at time = 5 min. After 300 min, the chlorovinyl pyrrolidine **2a** was recovered in 36%; 10% of starting allene **1a** together with a 35% of allene **3a** were still present. Anyway, the formation of product **2a** was observed only under irradiation, confirming the photocatalyzed nature of this process. Moreover, when the lamp was off, neither the consumption of allenes **1a** and **3a** nor the formation of **2a** took place. Moreover, quantum yield measurement (Φ = 0.54) was coherent with a photochemical mechanism excluding a radical chain.

Fluorescence quenching and Stern–Volmer experiments were also carried out (Figure 2b). Allenes **1a** and **3a**, the sodium salt of allene **Na**^**+**^**1a**^**–**^ and NCS were tested as the possible fluorescence quenchers of the excited state of [Ru(bpy)_3_](PF_6_)_2_. Despite the lower yield observed in CH_3_CN (Table 1, entry 4), this solvent was chosen for these measurements because it provided the complete dissolution of all the species involved in the fluorescence quenching experiments, thus ensuring the reproducibility of the measurements. NCS was not able to act as a quencher, even when the reaction conditions were reproduced in the presence of K_2_CO_3_ and of the allene, confirming the results reported by König and Lamar.^42,43^ Thus, an electrophilic amplification of NCS promoted by the oxidative quenching of the excited photocatalyst (PC*) had to be excluded. Furthermore, both allene **1a** and *N*-chloroallene **3a** were not able to provide fluorescence quenching. These findings suggested that a mechanism different from a typical photoredox process could be considered for the conversion of the chlorinated sulfonylamide **3a** into the final product **2a**. However, as evidenced in Figure 2b, the sodium salt of allene **Na**^**+**^**1a**^**–**^ was able to quench the excited species of [Ru(bpy)_3_](PF_6_)_2_. Thus, a plausible oxidation of this anion to the corresponding NCR **1a**^**•**^ is a process that cannot be excluded.

The radical nature of the process was then investigated using TEMPO as the radical scavenger. A drop in yield was noticed and the pyrrolidine **2a** was recovered in 15% yield in the presence of 35% of the starting allene **1a**. Anyway, neither adducts with TEMPO nor allene **3a** were detected. The same results were obtained using the chloroallene **3a** and the sodium salt of allene **1a**^**–**^**Na**^**+**^ as the starting materials.

To elucidate the role and the evolution of the putative reactive intermediates involved, the reactivity of the allene derivatives **3a** and **1a**^**–**^**Na**^**+**^ was explored. A selection of the results is reported in Table 3 (refer to the SI for the complete report). Given the fluorescence quenching experiments results, the anionic species **1a**^**–**^**Na**^**+**^ was first analyzed and reacted in the optimized reaction conditions. No loss in yield was observed (56% of **2a**) when compared to the reaction in which the allene **1a** is the starting material (Table 3a, entry 1). Instead, in the absence of the photocatalyst, only 13% conversion to product **2a** was observed with the formation of chlorinated **3a** as the major outcome (Table 3a, entry 2), whereas in the absence of the NCS and of the base no reaction was observed and allene **1a** was quantitatively recovered (Table 3a, entry 3). We deduced that **1a**^**–**^**Na**^**+**^ could be involved in two reaction pathways: chlorination by NCS to deliver the *N-*chloroallene **3a** and oxidation (upon reductive quenching of the photocatalyst) to the corresponding NCR that triggers an intramolecular aminochlorination of the allene moiety yielding the product **2a**. A similar investigation was then accomplished using *N*-chlorinated allene **3a** as the starting reagent. Also in this case, no deviation in yield was observed when compared to the results obtained in the case of allene **1a**, when **3a** was subjected to standard reaction conditions (Table 3b, entry 1).

**Table 3:**
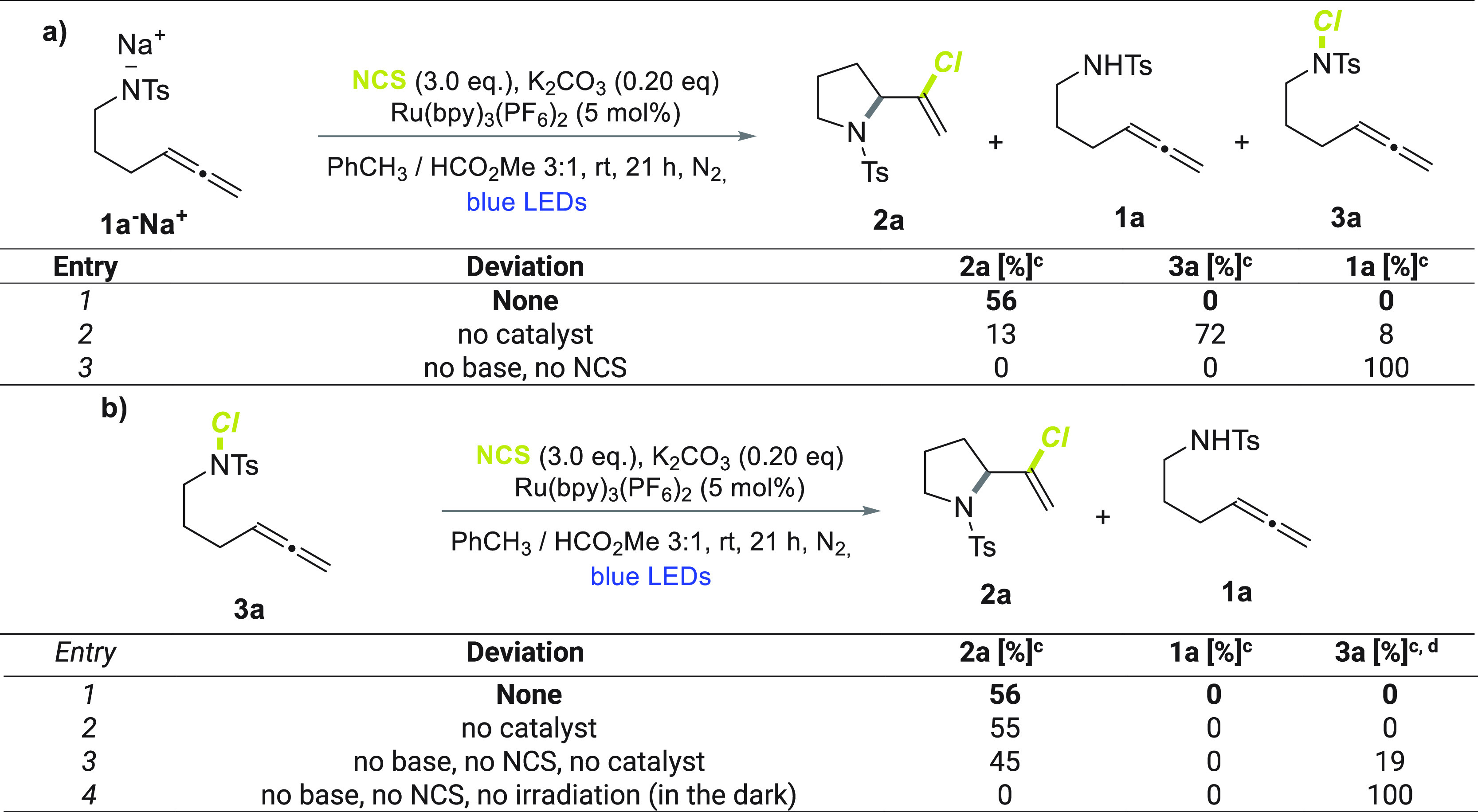
Screening of Sodium Salt of Allene **1a**^**–**^**Na**^**+**^ and *N*-Chloroallene **3a** Reactivity^a^

a Standard reaction conditions: (a) **1**^**–**^**Na**^**+**^ (0.20 mmol), K_2_CO_3_ (0.20 equiv, 0.04 mmol), NCS (3.0 equiv, 0.60 mmol), [Ru(bpy)_3_](PF_6_)_2_ (5 mol %, 0.01 mmol), anhydrous PhCH_3_ (3 mL), anhydrous HCO_2_CH_3_ (1 mL) under irradiation with 456 nm light source, blue light. (b) **3a** (0.20 mmol), K_2_CO_3_ (0.20 equiv, 0.04 mmol), NCS (3.0 equiv, 0.60 mmol), [Ru(bpy)_3_](PF_6_)_2_ (5 mol %, 0.01 mmol), anhydrous PhCH_3_ (3 mL), anhydrous HCO_2_CH_3_ (1 mL) under irradiation with 456 nm light source, blue light. (c) Yields were determined after 2 repetitions with ^1^H NMR using dichloroethane and nitromethane as internal standards. (d) Amount of unreacted **3a**.

**Figure 3:**
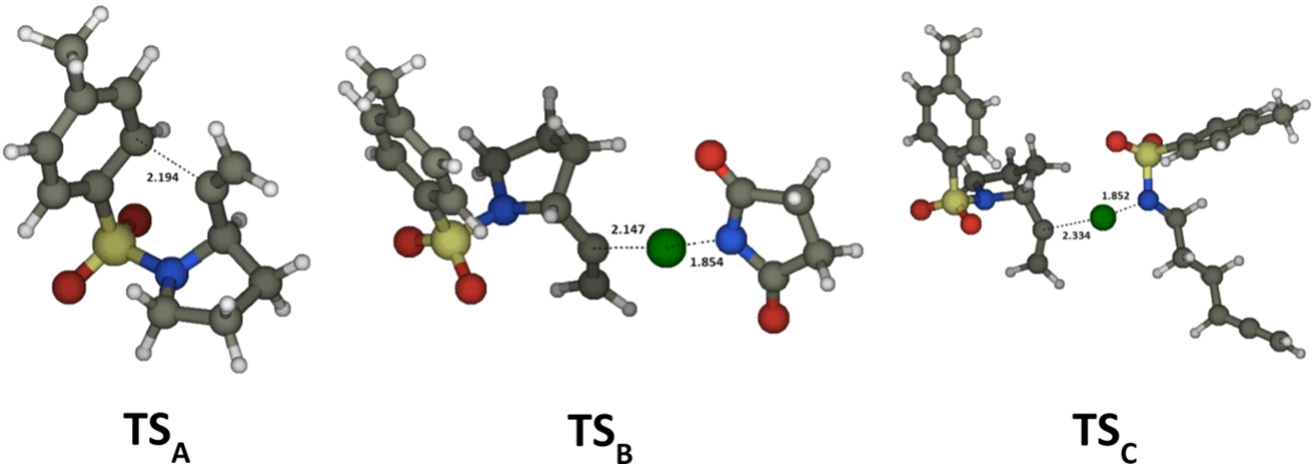
Transition structures: **TS**_**A**_ for the intramolecular cyclization of **1a**^**•**^ yielding the 1-(*N*-tosyl-pyrrol-1-yl)vinyl radical **R**_**1**_; **TS**_**B**_ for the Cl-AT from NCS to **R**_**1**_; **TS**_**C**_ for the Cl-AT from **3a** to **R**_**1**_.

Surprisingly, no erosion in **2a** yield was observed under irradiation in the absence of [Ru(bpy)_3_](PF_6_)_2_ (Table 3b, entry 2). Furthermore, vinyl pyrrolidine **2a** could be obtained in 45% yield starting from *N*-chlorinated allene **3a** also in the absence of both base, NCS, and photocatalyst under blue light (Table 3b, entry 3). In this case, unreacted chloroallene **3a** was recovered in 19% yield. On the contrary, no reaction was observed in the dark employing also **3a** as the starting material (Table 3b, entry 4). Moreover, the unreacted chloroallene **3a** was recovered when the reaction was performed without irradiation. These findings suggested that the chloroamide **3a** could act as a source of the corresponding sulfonamidyl NCR **1a**^**•**^. However, the fluorescence quenching experiments excluded that **3a** could participate in the quenching of the excited state of the photocatalyst to yield such NCR **1**^**•**^. Therefore, we hypothesized that a photolytic cleavage of the N–Cl bond of **3a** occurs upon the interaction of this chloroamide species **3a** with blue light. Such homolysis would generate both the NCR **1a**^**•**^ species and a chlorine radical. This hypothesis was supported by the results of Alexanian and co-workers concerning the development of *N*-chloroamides as reagents for the site selective C–H chlorination provided by the direct visible light cleavage of the *N*-Cl bond.^44,45^ Also the computational study is in favor of this hypothesis (for a full discussion, see Section S3, SI-CD). While the homolysis of the N–Cl bond is thermodynamically prohibitive (Δ*G* = 43.7 kcal mol^–1^, Table S2a), the photodissociation from the first triplet state T_1_ (used as a model for the singlet first excited state S_1_) shows a free energy barrier of only 7.1 kcal mol^–1^ and leads to the energetically favored separation of the radicals **1a**^**•**^ and Cl^**•**^ (Δ*G* = −3.0 kcal mol^–1^, Table S2b). The dissociation to the separated radicals is energetically even more favored (Δ*E* = −22.0 kcal mol^–1^) when starting from the S_1_ state. All the experimental data collected agreed with a radical mechanism. The key radical is the NCR **1a**^**•**^, which can be generated by the oxidation of the deprotonated allene **1a**^**–**^, the photoexcited ^3^Ru^II^(bpy)_3_, and the photodissociation of **3a** (Scheme 2). Once the NCR **1a**^**•**^ has been generated (Scheme 2, for a full discussion of the reaction mechanism see Section S4, SI-CD), the formation of the pyrrolidine ring occurs through an intramolecular radical addition (**TS**_**A**_, Figure 3) of the *N*-centered radical to the allene moiety yielding the highly reactive vinyl radical **R**_**1**_. The step is fast (Δ*G*^‡^ = 9.5 kcal mol^–1^, *k*_A_ = 6.9 × 10^5^ s^–1^) and thermodynamically favored (Δ*G* = −11.1 kcal mol^–1^). As shown in Scheme 2, from the radical intermediate **R**_**1**_ two main pathways open: the radical Chlorine-Atom-Transfer (Cl-AT) from the NCS (**TS**_**B**_, Figure 3, Δ*G*^‡^ = 10.0 kcal mol^–1^, *k*_B_′ = 7.2 × 10^6^ × [NCS] s^–1^, Δ*G* = −15.3 kcal mol^–1^) yielding the main product **2a** and the succinimi-*N*-yl **S**^•^ (the latter will be reduced to the anion by ^2^Ru^I^(bpy)_3_ regenerating the photocatalyst); the Cl-AT from the *N*-chloro allene **3a** (**TS**_**C**_, Figure 3, Δ*G*^‡^ = 8.3 kcal mol^–1^, *k*_C_′ = 1.3 × 10^8^ × [ACl] s^–1^, Δ*G* = −40.3 kcal mol^–1^) also yielded the main product **2a** and a new NCR **1a**^•^. Both Cl-AT are bimolecular processes requiring the presence of the chlorine donor. Apart from the obvious choice of NCS, the experimental and computational study suggested that the same role can also be assumed by **3a** easily formed in the reaction environment (see above).

## Conclusions

In conclusion, in this article we report a cyclization chlorination domino process as the synthetic strategy to obtain chlorovinyl pyrrolidines by means of blue light and NCS on *N*-substituted sulfonyl allenes in good yields. In our hypothesis, two different pathways contribute to the formation of a common NCR triggering the chloroamination of the allene moiety. Thus, both the homolytic cleavage of the N–Cl bond from the in situ formed *N*-chlorinated sulfonamide and a photoredox event involving the excited state of the photocatalyst might be implicated. The use of visible light was fundamental to provide access to the heterocyclic scaffold and to the synthetically valuable alkenyl chloride moiety.

## Experimental Section

### Materials and Methods

Flasks and all equipment used for the generation and reaction of moisture-sensitive compounds were dried by an electric heat gun under a vacuum and backfilled with N_2_, then used under a N_2_ atmosphere. All commercially available reagents and solvents were used as received. Anhydrous solvents were purchased by Sigma-Aldrich or distilled as indicated by Armarego.^46^ Products were purified by preparative column chromatography on Macherey-Nagel silica-gel for flash chromatography, 0.04–0.063 mm/230–400 mesh. Reactions were monitored by TLC using silica-gel on TLC-PET foils Sigma-Aldrich, 2–25 μm, layer thickness 0.2 mm, medium pore diameter 60 Å. NMR spectra were recorded employing a Jeol ECZR instrument. ^1^H NMR spectra were recorded in CDCl_3_ or DMSO-*d*_6_ at 600 MHz. ^13^C{^1^H} NMR spectra were recorded in CDCl_3_ at 150 MHz. Chemical shifts were reported in ppm relative to the resonance of CHCl_3_ (δ = 7.26) for ^1^H NMR, or referred to the central peak of CDCl_3_ (δ = 77.0) for ^13^C NMR. ^13^C NMR spectra were measured with complete proton decoupling; thus, ^13^C NMR implies ^13^C{^1^H} NMR in the NMR characterization of new products. DEPT experiments were carried out with a DEPT-135 sequence. ^1^H NMR coupling constants (*J*) were reported in Hertz (Hz), and multiplicities are indicated as follows: s (singlet), bs (broad singlet), d (doublet), t (triplet), q (quartet), m (multiplet), dd (doublet of doublets), ddd (doublet of doublets of doublets), dm (doublets of multiplet), td (triplet of doublets), tm (triplet of multiplets). Structural assignments were made with additional information from gCOSY, gNOESY experiments. Complete characterization of the light source and the instruments employed is reported in the SI.

### Typical Procedure for the Synthesis of 2-Chlorovinyl Saturated Nitrogen Heterocycles (**2a**–**u**)

A 10 mL Schlenk tube containing a magnetic stirring bar was dried with a heat gun under a vacuum, and then the tube was backfilled with N_2_. Three mL of PhCH_3_ and 1 mL of HCO_2_CH_3_ were added via syringe and degassed with N_2_ for 20 min. Then NCS (3.0 equiv, 0.60 mmol, 80 mg), [Ru(bpy)_3_](PF_6_)_2_ (0.05 equiv, 0.01 mmol, 8 mg), and K_2_CO_3_ (0.20 equiv, 0.04 mmol, 5 mg) were added in one portion. The resulting mixture was stirred and degassed for 2 min. Then, the allene **1** (1.0 equiv, 0.20 mmol) was added via syringe under N_2_ and the mixture was stirred and degassed for additional 2 min. Finally, the tube was sealed and placed under irradiation with a Kessil A160PR Blue LED (456 nm) placed at 3 cm distance for 21 h under continuous stirring. In order to analyze the crude reaction mixture by NMR, the reaction was filtered over a short pad of silica and eluted with 25 mL of AcOEt. Then, the solvent was evaporated. The crude mixture purified by flash chromatography to afford product **2**.

#### 2-(1-Chlorovinyl)-1-tosylpyrrolidine (**2a**)

Following the described procedure, allene **1a** (0.2 mmol, 50 mg) was reacted to obtain 32 mg of 2-(1-chlorovinyl)-1-tosylpyrrolidine **2a** as a colorless oil (eluent: EP 92/8 Acetone, yield: 56%). The reaction was scaled up to 1 mmol of allene **1a** (250 mg) to obtain 114 mg of 2-(1-chlorovinyl)-1-tosylpyrrolidine **2a** (eluent: EP 92/8 Acetone, yield: 40%). ^1^H NMR (600 MHz, CDCl_3_, Me_4_Si) δ 7.73 (d, *J* = 8.1 Hz, 2H, Ar-*H*), 7.32 (d, *J* = 7.8 Hz, 2H, Ar-*H*), 5.57 (s, 1H, CCl=CH_a_*H*_*b*_), 5.33 (s, 1H, CCl=C*H*_*a*_H_b_), 4.32 (m, 1H, N–C*H*–CCl), 3.49 (m, 1H, N–C(*H*)H–CH_2_), 3.28 (m, 1H, N–C(*H*)H–CH_2_), 2.43 (s, 3H, C*H*_3_), 1.97 (m, 1H, N–CH_2_–C(*H*)H), 1.89 (m, 1H, N–CH_2_–C(*H*)H), 1.73 (m, 1H, N–CH–C(*H*)H), 1.66 (m, 1H, N–CH–C(*H*)H). ^13^C{^1^H} NMR (151 MHz, CDCl_3_, Me_4_Si) δ 143.7 (Cq), 141.8 (Cq), 135.0 (Cq), 129.8 (2 × CH), 127.6 (2 × CH), 113.7 (CH_2_), 64.3 (CH), 49.3 (CH_2_), 31.1 (CH_2_), 23.9 (CH_2_), 21.6 (CH_3_). The data matches the one reported in the literature.^47^ HRMS (ESI) *m*/*z* [M + H]^+^ calcd for C_13_H_17_ClNO_2_S 286.0663, found 286.0664. IR ν max (neat)/cm^–1^ 2921, 2873, 1634, 1597, 1334, 1155, 812, 662.

#### 2-(1-Chlorovinyl)-1-(phenylsulfonyl)pyrrolidine (**2b**)

Following the described procedure, allene **1b** (0.2 mmol, 47 mg) was reacted to obtain 28 mg of 2-(1-chlorovinyl)-1-(phenylsulfonyl)pyrrolidine **2b** as a colorless oil (EP/Acetone 90/10, 52% yield). ^1^H NMR (600 MHz, CDCl_3_, Me_4_Si) δ 7.84 (m, 2H, Ar-*H*), 7.59 (m, 1H, Ar-*H*), 7.52 (m, 2H, Ar-*H*), 5.55 (t, *J* = 1.5 Hz, 1H, CCl=CH_a_*H*_*b*_), 5.32 (d, *J* = 1.5 Hz, 1H, CCl=CH_a_*H*_*b*_), 4.35 (m, 1H, m, 1H, N–C*H*–CCl), 3.50 (m, 1H, N–C(*H*)H–CH_2_), 3.30 (m, 1H, N–C(*H*)H–CH_2_), 1.98 (m, 1H, N–CH_2_–(C*H*)H), 1.89 (m, 1H, N–CH–C(*H*)H), 1.74 (m, 1H, N–CH–C(*H*)H), 1.67 (m, 1H, N–CH_2_–(C*H*)H). ^13^C{^1^H} NMR (151 MHz, CDCl_3_, Me_4_Si) δ 141.7 (Cq), 138.0 (Cq), 132.9 (CH), 129.2 (2 × CH), 127.5 (2 × CH), 113.9 (CH_2_), 64.4 (CH), 49.3 (CH_2_), 31.1 (CH_2_), 23.9 (CH_2_). HRMS (ESI) *m*/*z* [M + H]^+^ calcd for C_12_H_15_ClNO_2_S 272.0507, found 272.0506. IR ν max (neat)/cm^–1^ 2956, 1633, 1445, 1347,1159, 1092, 892, 723, 587.

#### 2-(1-Chlorovinyl)-1-(mesitylsulfonyl)pyrrolidine (**2c**)

Following the described procedure, allene **1c** (0.2 mmol, 56 mg) was reacted to obtain 32 mg of 2-(1-chlorovinyl)-1-(mesitylsulfonyl)pyrrolidine **2c** as a colorless oil (EP/Acetone 95/5, yield 51%). ^1^H NMR (600 MHz, CDCl_3_, Me_4_Si) δ 6.91 (m, 2H, Ar-*H*), 5.19 (t, *J* = 1.6 Hz, 1H, CCl=CH_a_*H*_*b*_), 4.98 (d, *J* = 1.6 Hz, 1H, CCl=CH_a_*H*_*b*_), 4.52 (m, 1H, N–C*H*–CCl), 3.58 (m, 1H, N–C(*H*)H–CH_2_), 3.34 (m, 1H, N–C(*H*)H–CH_2_), 2.61 (s, 6H, *o-*C*H*_3_-Ar), 2.27 (s, 3H, *p-*C*H*_3_-Ar), 2.18–2.10 (m, 1H, N–CH–C(*H*)H), 2.09–1.94 (m, 2H, N–CH–C(*H*)H), 1.93–1.85 (m, 1H, N–CH_2_–C*H*_2_). ^13^C{^1^H} NMR (151 MHz, CDCl_3_, Me_4_Si) δ 142.7 (Cq), 141.8 (Cq), 140.3 (2 × Cq), 133.2 (Cq), 131.9 (2 × CH), 113.6 (CH_2_), 63.7 (CH), 48.8 (CH_2_), 31.9 (CH_2_), 24.5 (CH_2_), 23.0 (2 × CH_3_), 21.1 (CH_3_). HRMS (ESI) *m*/*z* [M + H]^+^ calcd for C_15_H_21_ClNO_2_S 314.0976, found 314.0974. IR ν max (neat)/cm^–1^ 2930, 2886, 1602, 1321, 1311, 1147, 674.

#### 1-((4-Chlorophenyl)sulfonyl)-2-(1-chlorovinyl)pyrrolidine (**2d**)

Following the described procedure, allene **1d** (0.2 mmol, 54 mg) was reacted to obtain, after 32 h, 24.5 mg of 1-((4-chlorophenyl)sulfonyl)-2-(1-chlorovinyl)pyrrolidine **2d** as a colorless oil (EP/Acetone 92/8, 40% yield). ^1^H NMR (600 MHz, CDCl_3_, Me_4_Si): 7.78 (dm, *J* = 8.1 Hz, 2H, Ar-*H*), 7.50 (dm, *J* = 8.1 Hz, 2H, Ar-*H*), 5.53 (d, *J* = 1.7 Hz, 1H, CCl=CH_a_*H*_*b*_), 5.32 (d, *J* = 1.7 Hz, 1H, CCl=C*H*_*a*_H_b_), 4.37 (m, 1H, N–C*H*–CCl), 3.48 (m, 1H, N–C(*H*)H–CH_2_), 3.33 (m, 1H, N–C(*H*)H–CH_2_), 2.05–1.98 (m, 1H, N–CH–C(*H*)H), 1.98–1.88 (m, 1H, N–CH_2_–(C*H*)H), 1.86–1.77 (m, 1H, N–CH–C(*H*)H), 1.77–1.69 (m, 1H, N–CH_2_–(C*H*)H). ^13^C{^1^H} NMR (151 MHz, CDCl_3_) δ 141.6 (Cq), 139.5 (Cq), 136.9 (Cq), 129.5 (2 × CH), 128.9 (2 × CH), 114.2 (CH_2_), 64.5 (CH), 49.3 (CH_2_), 31.2 (CH_2_), 24.0 (CH_2_). HRMS (ESI) *m*/*z* [M + H]^+^ calcd for C_12_H_14_Cl_2_NO_2_S 306.0117, found 306.0117. IR ν max (neat)/cm^–1^ 3095, 2975, 1634, 1346, 1181, 1086, 843, 755.

#### 1-(4-((2-(1-Chlorovinyl)pyrrolidin-1-yl)sulfonyl)phenyl)ethan-1-one (**2e**)

Following the described procedure, allene **1e** (0.2 mmol, 56 mg) was reacted to obtain 31 mg of 2-(1-chloro-2-cyclohexylvinyl)-1-tosylpyrrolidine **2e** as a colorless oil (EP/Acetone 85/15, yield 50%). ^1^H NMR (600 MHz, CDCl_3_, Me_4_Si) δ 8.07 (d, *J* = 8.5 Hz, 2H, Ar-*H*), 7.93 (d, *J* = 8.5 Hz, 2H, Ar-*H*), 5.52 (t, *J* = 1.7 Hz, 1H, CCl=CH_a_*H*_*b*_)), 5.31 (d, *J* = 1.7 Hz, 1H, CCl=C*H*_*a*_H_b_), 4.40 (m, 1H, N–C*H*–CCl), 3.52–3.47 (m, 1H, N–C(*H*)H–CH_2_), 3.39–3.35 (m, 1H, N–C(*H*)H–CH_2_), 2.65 (s, 3H, CO–C*H*_3_), 2.04–1.96 (m, 1H, N–CH–C(*H*)H), 1.97–1.89 (m, 1H, N–CH_2_–(C*H*)H), 1.88–1.76 (m, 1H, N–CH–C(*H*)H), 1.76–1.68 (m, 1H, N–CH_2_–(C*H*)H). ^13^C{^1^H} NMR (151 MHz, CDCl_3_, Me_4_Si) δ 196.9 (Cq), 142.3 (Cq), 141.5 (Cq), 140.2 (Cq), 129.0 (2 × CH), 127.8 (2 × CH), 114.3 (CH_2_), 64.5 (CH), 49.4 (CH_2_), 31.3 (CH_2_), 27.0 (CH_3_), 24.1 (CH_2_). HRMS (ESI) *m*/*z* [M + H]^+^ calcd for C_14_H_17_ClNO_3_S 314.0612, found 314.0612. IR ν max (neat)/cm^–1^ 1689, 1635, 1348, 903, 835.

#### 2-(1-Chlorovinyl)-1-((4-methoxyphenyl)sulfonyl)pyrrolidine (**2f**)

Following the described procedure, allene **1f** (0.2 mmol, 54 mg) was reacted to obtain 24 mg of 2-(1-chlorovinyl)-1-((4-methoxyphenyl)sulfonyl)pyrrolidine **2f** as a light yellow oil (eluent: from EP to EP/Acetone 80/20, yield: 40%). ^1^H NMR (600 MHz, CDCl_3_, Me_4_Si) δ 7.78 (d, *J* = 8.9 Hz, 2H, Ar-*H*), 6.99 (d, *J* = 8.9 Hz, 2H, Ar-*H*), 5.57 (t, *J* = 1.2 Hz, 1H, CCl=CH_a_*H*_*b*_), 5.32 (d broad, *J* = 1.2 Hz, 1H, CCl=C*H*_*a*_H_b_), 4.32 (dd, *J* = 8.5, 3.1 Hz, 1H, N–C*H*), 3.88 (s, 3H, OMe), 3.48 (ddd, *J* = 9.8, 7.2, 4.1 Hz, 1H, N–C(*H*)H–CH_2_), 3.28 (m, 1H, N–C(*H*)H–CH_2_), 1.98 (m, 1H, N–CH_2_–C(*H*)H), 1.88 (m, 1H, N–CH–C(*H*)H), 1.75 (m, 1H, CH–C(*H*)H–CH_2_), 1.67 (m, 1H, N–CH_2_–C(*H*)H–CH_2_). ^13^C{^1^H} NMR (151 MHz, CDCl_3_, Me_4_Si) δ 163.0 (Cq), 141.8 (Cq), 129.7 (Cq), 129.5 (2 × CH), 114.2 (2 × CH), 113.7 (CH_2_), 64.2 (CH), 55.6 (OCH_3_), 49.2 (CH_2_), 31.0 (CH_2_), 23.8 (CH_2_). HRMS (ESI) *m*/*z* [M + H]^+^ calcd for C_13_H_17_ClNO_3_S 302.0612, found 302.0615. IR ν max (neat)/cm^–1^ 1496, 1344, 1092, 833, 667.

#### 2-(1-Chlorovinyl)-1-((2,3-dihydrobenzo[*b*][1,4]dioxin-6-yl)sulfonyl)pyrrolidine (**2g**)

Following the described procedure, allene **1g** (0.2 mmol, 59 mg) was reacted to obtain 26 mg of 2-(1-chlorovinyl)-1-((2,3-dihydrobenzo[*b*][1,4]dioxin-6-yl)sulfonyl)pyrrolidine **2g** as a light yellow oil (eluent: from EP to EP/Et_2_O 50/50, yield: 40%). ^1^H NMR (600 MHz, CDCl_3_, Me_4_Si) δ 7.37 (d, *J* = 2.1 Hz, 1H, Ar-*H*), 7.33 (dd, *J* = 8.4, 2.2 Hz, 1H, Ar-*H*), 6.96 (d, *J* = 8.5 Hz, 1H, Ar-*H*), 5.58 (t, *J* = 1.0 Hz, 1H, CCl=CH_a_*H*_*b*_), 5.33 (d broad, *J* = 1.8 Hz, 1H, CCl=C*H*_*a*_H_b_), 4.29–4.33 (m, 5H, O–(C*H*_2_)_2_–O and N–C*H*–CH_2_), 3.48 (ddd, *J* = 10.3, 6.8, 4.2 Hz, 1H, N–C(*H*)H–CH_2_), 3.28 (m, 1H, N–C(*H*)H–CH_2_), 1.98 (m, 1H, CH–C(*H*)H), 1.88 (m, 1H, N–CH_2_–C(*H*)H), 1.75 (m, 1H, N–CH_2_–C(*H*)H), 1.68 (m, 1H, N–CH–C(*H*)H–CH_2_). ^13^C{^1^H} NMR (151 MHz, CDCl_3_, Me_4_Si) δ 147.5 (Cq), 143.5 (Cq), 141.8 (Cq), 130.4 (Cq), 121.2 (CH), 117.7 (CH), 117.0 (CH), 113.6 (CH_2_), 64.5 (CH_2_), 64.2 (CH), 64.2 (CH_2_), 49.2 (CH_2_), 31.0 (CH_2_), 23.7 (CH_2_). HRMS (ESI) *m*/*z* [M + H]^+^ calcd for C_14_H_17_ClNO_4_S 330.0561, found 330.0565. IR ν max (neat)/cm^–1^ 1633, 1344, 1285, 877, 699.

#### 2-(1-Chlorovinyl)-1-(methylsulfonyl)pyrrolidine (**2h**)

Following the described procedure, allene **1h** (0.2 mmol, 40 mg) was reacted to obtain 21 mg of 2-(1-chlorovinyl)-1-(methylsulfonyl)pyrrolidine **2h** as a colorless oil (eluent: EP 92/8 Acetone, yield: 50%). ^1^H NMR (600 MHz, CDCl_3_, Me_4_Si) δ 5.51 (d, *J* = 1.7, 1H, CCl=CH_a_*H*_*b*_), 5.34 (d, *J* = 1.7 Hz, 1H, CCl=C*H*_*a*_H_b_), 4.48 (m, 1H, N–C*H*–CH_2_), 3.55–3.52 (m, 1H, N–C(*H*)H–CH_2_), 3.45–3.42 (m, 1H, N–C(*H*)H–CH_2_), 2.87 (s, 3H, C*H*_3_), 2.18–1.99 (m, 3H, N–CH–C*H*_2_), 1.97–1.87 (m, 1H, N–CH_2_–C*H*_2_). ^13^C{^1^H} NMR (151 MHz, CDCl_3_, Me_4_Si) δ 141.9 (Cq), 114.6 (CH_2_), 64.1 (CH), 49.0 (CH_2_), 38.5 (CH_3_), 31.5 (CH_2_), 24.5 (CH_2_). HRMS (ESI) *m*/*z* [M + H]^+^ calcd for C_7_H_13_ClNO_2_S 210.0350, found 210.0354. IR ν max (neat)/cm^–1^ 2931, 1630, 1324, 1141, 1060, 1008, 970, 889.

#### 1-(((2-(1-Chlorovinyl)pyrrolidin-1-yl)sulfonyl)methyl)-7,7-dimethylbicyclo[2.2.1]heptan-2-one (**2i**)

Following the described procedure, allene **1i** (0.2 mmol, 69 mg) was reacted to obtain 22 mg of 2-(1-chlorovinyl)-1-tosylpyrrolidine **2i** as a colorless oil (eluent: EP 92/8 Acetone, yield: 32%, mixture of 2 isomers ratio 1:1). ^1^H NMR (600 MHz, CDCl_3_, Me_4_Si) δ 5.56 (s, 1H, CCl=CH_a_*H*_*b*_*isomer A*), 5.53 (s, 1H, CCl=CH_a_*H*_*b*_*isomer B*), 5.36 (s, 1H, CCl=C*H*_*a*_H_b_*isomer A*), 5.34 (s, 1H, CCl=C*H*_*a*_H_b_*isomer B*), 4.56–4.49 (m, 1H, N–C*H*–CCl, *both isomers*), 3.66–3.56 (m, 1H, N–CH_a_*H*_*b*_–CH_2_, *both isomers*), 3.50–3.44 (m, 1H, SO_2_–C(H)*H, both isomers*), 3.48–3.41 (m, 1H, N–C*H*_*a*_H_b_–CH_2_, *both isomers*), 2.92–2.82 (m, 1H, SO_2_–C(H)*H, both isomers*), 2–55–2.48 (m, 1H, C(=O)—C(H)*H, both isomers*), 2.40–2.30 (m, 1H, CH_2_–C(C)*H*–CH_2_, *both isomers*), 2.20–2.12 (m, 2H, N–CH_2_–(C*H*_2_)_2_, *both isomers*), 2.13–1.99(m, 3H, C—(C(*H*)H)_2_—CH—C(=O), N–CH_2_–(C*H*_2_)_2_, *both isomers*), 1.96–1.89 (m, 2 H, C(=O)-C(H)*H*, CH_2_–C(C)*H*-CH_2_, *both isomers*), 1.70–1.60 (m, 1H, C—(C(*H*)H)_2_—CH—C(=O), *both isomers*), 1.48–1.40 (m, 1H, C—(C(*H*)H)_2_—CH—C(=O), *both isomers*), 1.13 (s, 3H, C(CH_3_)C*H*_3_, *isomer A*), 1.11 (s, 3H, C(CH_3_)C*H*_3_, *isomer A*), 0.87 (s, 3H, C(CH_3_)C*H*_3_, *isomer A*), 0.86 (s, 3H, C(CH_3_)C*H*_3_, *isomer B*). ^13^C{^1^H} NMR (151 MHz, CDCl_3_, Me_4_Si): *mixture of isomers* δ 215.4 (Cq), 215.3 (Cq), 142.4 (Cq), 142.0 (Cq), 114.5 (CH_2_), 114.5 (CH_2_), 64.2 (CH), 64.0 (CH), 58.5 (Cq), 58.4 (Cq), 49.2 (CH_2_), 48.0 (Cq), 47.9 (CH_2_), 47.9 (Cq), 43.2 (CH), 42.8 (CH), 42.7 (CH_2_), 42.7 (CH_2_), 31.5 (CH_2_), 31.3 (CH_2_), 27.1 (CH_2_), 27.0 (CH_2_), 38.5 (CH_3_), 31.5 (CH_2_), 25.2 (CH_2_), 25.1 (CH_2_), 24.6 (CH_2_), 24.5 (CH_2_), 20.2 (CH_3_), 20.2 (CH_3_), 19.9 (CH_3_), 19.9 (CH_3_). HRMS (ESI) *m*/*z* [M + H]^+^ calcd for C_16_H_25_ClNO_3_S 346.1238, found 346.1235. IR ν max (neat)/cm^–1^ 2960, 2900, 1742, 1336, 1160, 573.

#### 2-(1-Chlorovinyl)-1-tosylpiperidine (**2j**)

Following the described procedure, allene **1j** (0.2 mmol, 53 mg) was reacted to obtain, 12 mg of 2-(1-chlorovinyl)-1-tosylpiperidine **2j** as a colorless oil (eluent EP 95/5 Acetone, yield: 20%). ^1^H NMR (600 MHz, CDCl_3_, Me_4_Si) δ 7.72 (dm, *J* = 8.1 Hz, 2H, Ar-*H*), 7.29 (dm, *J* = 8.1 Hz, 2H, Ar-*H*), 5.44 (t, *J* = 2.0 Hz, 1H, CCl=CH_a_*H*_*b*_), 5.38 (t, *J* = 2.0 Hz, 1H, CCl=C*H*_*a*_H_b_), 4.77 (m, 1H, N–C*H*–CH_2_), 3.74 (dm, *J* = 13.7, 1H, N–C(*H*)H–CH_2_), 3.09 (tm, *J* = 13.7, 1H, N–C(*H*)H–CH_2_), 2.43 (s, 3H, C*H*_3_), 2.22 (m, 1H, N–C(CCl)H–(C(H)*H*), 1.58–1.39 (m, 3H, N–CH_2_–(C*H*_2_), N–C(CCl)H–CH_2_–(C(H)*H*), 1.29 (m, 2H, m, 1H, N–C(CCl)H–(C(H)*H*), N–C(CCl)H–CH_2_–(C(H)*H*)). ^13^C{^1^H} NMR (151 MHz, CDCl_3_) δ 143.3 (Cq), 139.6 (Cq), 138.1 (Cq), 129.7 (2 × CH), 127.1 (2 × CH), 115.2 (CH_2_), 57.2 (CH), 41.8 (CH_2_), 26.6 (CH_2_), 24.4 (CH_2_), 21.6 (CH_3_), 18.8 (CH_2_). HRMS (ESI): Chemical Formula C_14_H_18_ClNO_2_S; *m*/*z* [M + K]^+^ calcd for C_14_H_18_ClKNO_2_S 338.0378, found 338.0378. IR ν max (neat)/cm^–1^ 2924, 2855, 1596, 1446, 1337, 1156, 1092, 956, 813, 653.

#### 2-(1-Chlorohept-1-en-1-yl)-1-tosylpyrrolidine (**2k**)

Following the described procedure, allene **1k** (0.2 mmol, 64 mg) was reacted to obtain 34 mg of 2-(1-chlorohept-1-en-1-yl)-1-tosylpyrrolidine **2k** as a colorless oil (EP/Acetone 90/10, yield 48%). Mixture of *E*/*Z* isomers, isomer A/B ratio 1:3.8.^1^H NMR (600 MHz, CDCl_3_, Me_4_Si) δ 7.70 (d, *J* = 8.2 Hz, 2H, Ar-*H isomer A*), 7.69 (d, *J* = 8.2 Hz, 2H, Ar-*H isomer B*), 7.27 (d, *J* = 8.2 Hz, 2H, Ar-*H isomer B*), 7.28 (d, *J* = 8.2 Hz, 2H, Ar-*H isomer A*), 5.82 (t, *J* = 7.8 Hz, 1H, CCl=C*H*, *isomer B*), 5.63 (t, *J* = 7.8 Hz, 1H, CCl=C*H*, *isomer A*), 4.81 (dd, *J* = 8.2, 3.5 Hz, 1H, N–C*H*–CH_2_, *isomer A*), 4.35 (dd, *J* = 8.2, 3.5 Hz, 1 H, N–C*H*–CH_2_, *isomer B*), 3.60–3.53 (m, 1H, N–C(*H*)H–CH_2_, *isomer A*), 3.47–3.40 (m, 1H, N–C(*H*)H–CH_2_, *isomer B*), 3.39–3.31 (m, 2H, N–C(*H*)H–CH_2_ mixture of isomers), 2.41 (s, 3H, Ar–C*H*_3_), 2.26–2.10 (m, 2H, C*H*_2_—C*H*=, *isomer A*), 2.12 (q, *J* = 7.3 Hz, 2H, C*H*_2_—C*H*=, *isomer B*), 2.04–1.84 (m, 3H, N–CH_2_–(C*H*_2_)_2_), 1.80–1.71 (m, 1H, N–CH–C(*H*)H, *isomer B*), 1.70–1.61 (m, 1H, N–CH_2_–C(*H*)H, *isomer B*), 1.51–1.22 (m, 6H, CH_3_–C*H*_2_–CH_2_, CH_2_–(C*H*_2_)_2_–CH_2_*isomer A*), 1.36 (quin, 2H, *J* = 7.3 Hz, CH_3_–C*H*_2_–CH_2_, *isomer B*), 1.33–1.23 (m, 4H, CH_2_–(C*H*_2_)_2_–CH_2_*isomer B*), 0.89 (t, *J* = 7.0 Hz, 3H, CH_2_–C*H*_3_), 0.88 (t, *J* = 7.0 Hz, 3H, CH_2_–C*H*_3_). ^13^C{^1^H} NMR (151 MHz, CDCl_3_, Me_4_Si): *Isomer A* δ 143.3 (Cq), 136.4 (Cq), 134.0 (Cq), 131.1 (CH), 129.5 (2 × CH), 127.4 (2 × CH), 58.2 (CH), 49.4 (CH_2_), 31.6 (CH_2_), 31.5 (CH_2_), 29.1 (CH_2_), 28.5 (CH_2_), 25.3 (CH_2_), 24.1 (CH_2_), 21.6 (CH_3_), 14.1 (CH_3_); *Isomer B* δ 143.4 (Cq), 135.7 (Cq), 133.7 (Cq), 129.6 (2 × CH), 127.9 (CH), 127.5 (2 × CH), 64.7 (CH), 49.2 (CH_2_), 31.5 (CH_2_), 31.3 (CH_2_), 28.3 (CH_2_), 28.1 (CH_2_), 24.1 (CH_2_), 22.5 (CH_2_), 21.6 (CH_2_), 14.1 (CH_3_). HRMS (ESI) *m*/*z* [M + H]^+^ calcd for C_19_H_29_ClNO_2_S 370.1602, found 370.1602. IR ν max (neat)/cm^–1^ 2954, 2857, 1348, 1157, 991, 586.

#### 2-(1-Chloro-2-cyclohexylvinyl)-1-tosylpyrrolidine (**2l**)

Following the described procedure, allene **1l** (0.2 mmol, 65 mg) was reacted to obtain 34 mg of 2-(1-chloro-2-cyclohexylvinyl)-1-tosylpyrrolidine **2l** as a colorless oil (EP/Acetone 90/10, yield 44%). Mixture of *E*/*Z* isomers, isomer A/B ratio 1:4.6. ^1^H NMR (600 MHz, CDCl_3_, Me_4_Si) δ 7.70 (d, *J* = 8.2 Hz, 2H, Ar-*H isomer A*), 7.69 (d, *J* = 8.2 Hz, 2H, Ar-*H isomer B*), 7.28 (d, *J* = 8.2 Hz, 2H, Ar-*H isomer B*), 7.28 (d, *J* = 8.2 Hz, 2H, Ar-*H isomer A*), 5.63 (d, *J* = 8.8 Hz, 1H, CCl=C*H isomer B*), 5.49 (d, *J* = 8.8 Hz, 1H, CCl=C*H isomer A*), 4.80 (dd, *J* = 8.2, 3.5 Hz, 1 H, N–C*H*–CH_2_, *isomer A*), 4.33 (dd, *J* = 8.2, 3.5 Hz, 1 H, N–C*H*–CH_2_, *isomer B*), 3.62–3.52 (m, 1H, N–C(*H*)H–CH_2_, *isomer A*), 3.47–3.41 (m, 1H, N–C(*H*)H–CH_2_, *isomer B*), 3.41–3.32 (m, 2H, N–C(*H*)H–CH_2_), 2.43 (s, 3H, Ar–C*H*_3_*isomer A*), 2.41 (s, 3H, Ar–C*H*_3_*isomer B*), 2.41–2.31 (m, 1H, —C*H*—CH=CCl), 2.06–1.83 (m, 1H, m, 1H, N–CH_2_–(C*H*_2_)_2_), 1.81–1.72 (m, 2H, m, 1H, N–CH_2_–(C*H*_2_)_2_), 1.71–1.59 (m, 5H, N–CH_2_–(C*H*_2_)_2_, (CH_2_)_2_–C*H*_2_-(CH_2_)_2_, cyclohexyl C*H*_2_) 1.37–1.12 (m, 4H, cyclohexyl C*H*_2_), 1.12–0.98 (m, 2H, CCl—C=CH—C(CH_2_)*H*_2_). ^13^C{^1^H} NMR (151 MHz, CDCl_3_, Me_4_Si) *Isomer A* δ 143.3 (Cq), 136.5 (CH), 136.4 (Cq), 135.8 (Cq), 129.6 (2 × CH), 127.4 (2 × CH), 58.5 (CH), 49.5 (CH_2_), 38.1 (CH), 33.6 (CH_2_), 32.7 (CH_2_), 32.7 (CH_2_), 25.9 (CH_2_), 25.8 (2 × CH_2_), 25.3 (CH_2_), 21.7 (CH_3_). *Isomer B* δ 143.4 (Cq), 135.8 (Cq), 133.0 (CH), 132.0 (Cq), 129.6 (2 × CH), 127.5 (2 × CH), 64.7 (CH), 49.3 (CH_2_), 37.5 (CH), 31.9 (CH_2_), 31.9 (CH_2_), 31.3 (CH_2_), 26.1 (CH_2_), 25.7 (CH_2_), 24.1 (CH_2_), 21.7 (CH_3_). HRMS (ESI) *m*/*z* [M + H]^+^ calcd for C_19_H_26_ClNO_2_S 368.1446, found 368.1447. IR ν max (neat)/cm^–1^ 2962, 1258, 1087, 1008, 788, 663.

#### (*Z*)-2-(1-Chloro-2-(4-methoxyphenyl)vinyl)-1-tosylpyrrolidine (**2m**)

Following the described procedure, allene **1m** (0.2 mmol, 71 mg) was reacted to obtain 40 mg 2-(1-chloro-2-(4-methoxyphenyl)vinyl)-1-tosylpyrrolidine **2m** as a colorless oil (EP/Acetone 90/10, 54% yield). Only one isomer was recovered. ^1^H NMR (600 MHz, CDCl_3_, Me_4_Si) δ 7.80 (dm, *J* = 8.1 Hz, 2H, Ar^1^-*H*), 7.56 (dm, *J* = 8.7 Hz, 2H, Ar^2^-*H*), 7.36 (dm, *J* = 8.1 Hz, 2H, Ar^1^-*H*), 6.88 (dm, *J* = 8.7 Hz, 2H, Ar^2^-*H*), 5.89 (s, 1H, CCl=C*H*—Ar^2^), 4.74 (m, 1H, N–C*H*–CH_2_), 3.86 (m, 1H, N–C(*H*)H–CH_2_) 3.82 (s, 3H, O–C*H*_3_), 3.55 (m, 1H, N–C(*H*)H–CH_2_), 2.45 (s, 3H, C*H*_3_), 2.33 (m, 1H, N–CH–C(*H*)H), 1.92 (m, 1H, N–CH–C(*H*)H), 1.78 (m, 1H, N–CH_2_–C(*H*)H), 1.10 (m, 1H, N–CH_2_–C(*H*)H). ^13^C{^1^H} NMR (151 MHz, CDCl_3_, Me_4_Si) δ 160.1 (Cq), 144.4 (Cq), 135.3 (Cq), 131.6 (2 × CH), 130.2 (2 × CH), 128.9 (Cq), 127.7 (2 × CH), 113.2 (2 × CH), 99.7 (Cq), 67.0 (CH), 66.7 (CH), 55.4 (CH_3_), 52.3 (CH_2_), 30.1 (CH_2_), 24.8 (CH_2_), 21.7 (CH_3_). HRMS (ESI) *m*/*z* [M + H]^+^ calcd for C_20_H_23_ClNO_3_S 392.1082, found 392.1073. IR ν max (neat)/cm^–1^ 2924, 1610, 1152, 1348, 1250, 1159, 1087, 1030, 841, 664.

#### 2-(1-Chloro-2-methylprop-1-en-1-yl)-1-tosylpyrrolidine (**2n**)

Following the described procedure, allene **1n** (0.2 mmol, 53 mg) was reacted to obtain 19 mg of 2-(1-chloro-2-methylprop-1-en-1-yl)-1-tosylpyrrolidine **2n** as a colorless oil (EP/Acetone 90/10, 30% yield). ^1^H NMR (600 MHz, CDCl_3_, Me_4_Si) δ 7.66 (dm, *J* = 8.1 Hz, 2H, Ar-*H*), 7.25 (dm, *J* = 8.1 Hz, 2H, Ar-*H*), 4.93 (m, 1H, N–C*H*–CH_2_), 3.62 (m, 1H, N–C(*H*)H–CH_2_), 3.38 (m, 1H, N–C(*H*)H–CH_2_), 2.40 (s, 3H, Ar–C*H*_3_), 2.01 (m, 2H, N–CH_2_–(C*H*_2_)_2_), 1.93 (m, 1H, m, 2H, N–CH_2_–C*H*_2_), 1.89 (s, 3H, ClC*=*C—C(*H*_3_)CH_3_), 1.73 (s, 3H, ClC*=*C—C(*H*_3_)CH_3_), 1.64 (m, 2H, N–CH_2_–(C*H*_2_)_2_). ^13^C{^1^H} NMR (151 MHz, CDCl_3_, Me_4_Si) δ 143.1 (Cq), 136.8 (Cq), 131.0 (Cq), 129.3 (2 × CH), 128.9 (Cq), 127.3 (2 × CH), 59.4 (CH), 49.3 (CH_2_), 31.4 (CH_2_), 25.3 (CH_2_), 22.4 (CH_3_), 21.6 (CH_3_), 20.6 (CH_3_). HRMS (ESI) *m*/*z* [M + H]^+^ calcd for C_15_H_21_ClNO_2_S 314.0976, found 314.0977. IR ν max (neat)/cm^–1^ 2920, 2850, 1340, 1185, 1152, 586.

#### 2-(Chloro(cyclopentylidene)methyl)-1-tosylpyrrolidine (**2o**)

Following the described procedure, allene **1o** (0.2 mmol, 61 mg) was reacted to obtain 19 mg of 2-(chloro(cyclopentylidene)methyl)-1-tosylpyrrolidine **2o** as a colorless oil (EP/Acetone 9/1, 28% yield). ^1^H NMR (600 MHz, CDCl_3_, Me_4_Si):δ 7.66 (d, *J* = 8.3 Hz, 2H, Ar-*H*), 7.25 (d, *J* = 8.3 Hz, 2H, Ar-*H*), 4.75 (m, 1H, N–C*H*–CH_2_), 3.62 (m, 1H, N–C(*H*)H–CH_2_), 3.38 (m, 1H, N–C(*H*)H–CH_2_), 2.67 (m, 1H, N–CH_2_–(C*H*_2_)_2_), 2.40 (s, 3H, Ar–C*H*_3_), 2.33–2.26 (m, 1H, N–CH_2_–(C*H*_2_)_2_), 2.25–2.15 (m, 2H, C=C—(C(H)*H*)_2_), 2.09–1.97 (m, 2H, N–CH_2_–(C*H*_2_)_2_), 1.97–1.89 (m, 1H, C=C—C_b_(*H*)H), 1.79–1.69(m, 2H, N–CH_2_–(C*H*_2_)_2_) 1.65 (m, 3H, C=C—C_a_(*H*)H, C=C—(CH_2_)_2_—(CH_2_)_2_). ^13^C{^1^H} NMR (151 MHz, CDCl_3_, Me_4_Si) δ 143.4 (Cq), 143.1 (Cq), 136.8 (Cq), 129.3 (2 × CH), 127.4 (2 × CH), 124.8 (Cq), 61.2 (CH), 49.3 (CH_2_), 33.4 (CH_2_), 31.2 (CH_2_), 31.2 (CH_2_), 27.6 (CH_2_), 25.8 (CH_2_), 25.2 (CH_2_), 21.6 (CH_3_). HRMS (ESI) *m*/*z* [M + H]^+^ calcd for C_17_H_23_ClNO_2_S 340.1133, found 340.1136. IR ν max (neat)/cm^–1^ 2923, 2865, 1598, 1347, 1154, 1091, 1001, 809, 670, 586 cm.

#### 2-(Chloro(cyclohexylidene)methyl)-1-tosylpyrrolidine (**2p**)

Following the described procedure, allene **1p** (0.2 mmol, 65 mg) was reacted to obtain 21 mg of 2-(chloro(cyclohexylidene)methyl)-1-tosylpyrrolidine **2p** as a white solid (EP/Acetone 9/1, 30% yield). ^1^H NMR (600 MHz, CDCl_3_, Me_4_Si) δ 7.68 (d, *J* = 8.3 Hz, 2H, Ar-*H*), 7.25 (d, *J* = 8.3 Hz, 2H, Ar-*H*), 5.01 (m, 1H, N–C*H*–CH_2_), 3.63 (m, 1H, N–C(*H*)H–CH_2_), 3.38 (m, 1H, N–C(*H*)H–CH_2_), 2.46–2.39 (m, 2H, C=C—C_a_(H)*H* and C=C—C_a′_(H)*H*), 2.40 (s, 3H, Ar–C*H*_3_,), 2.35–2.19 (m, 2H, C=C—C_a_(H)*H* and C=C—C_a′_(H)*H*), 2.08–1.93 (m, 3H, N–CH–(C*H*_2_)_2_, C*H*_2_*cyclohexyl*), 1.74–1.68 (m, 1H, C*H*_2_*cyclohexyl*), 1.67–1.60 (m, 2H, N–CH–(C*H*_2_)_2_), 1.59–1.48 (m, 4H, C*H*_2_*cyclohexyl*). ^13^C{^1^H} NMR (151 MHz, CDCl_3_, Me_4_Si) δ 143.0 (Cq), 138.2 (Cq), 136.9 (Cq), 129.4 (2 × CH), 127.4 (2 × CH), 126.5 (Cq), 58.8 (CH), 49.4 (CH_2_), 32.1 (CH_2_), 31.8 (CH_2_), 31.3 (CH_2_), 27.6 (CH_2_), 26.9 (CH_2_), 26.4 (CH_2_), 25.4 (CH_2_), 21.6(CH_3_). HRMS (ESI) *m*/*z* [M + H]^+^ calcd for C_18_H_25_ClNO_2_S 354.1289, found 354.1286. IR ν max (neat) cm^–1^ 2975, 2846, 1331, 1154, 1010, 579. mp 82 °C.

#### 2-((4-(*tert*-Butyl)cyclohexylidene)chloromethyl)-1-tosylpyrrolidine (**2q**)

Following the described procedure, allene **1q** (0.2 mmol, 71 mg) was reacted to obtain 26 mg of 2-((4-(*tert*-butyl)cyclohexylidene)chloromethyl)-1-tosylpyrrolidine **2q** as a colorless oil (EP/Acetone 9/1, 32% yield). Two conformers are observed in NMR spectra. ^1^H NMR (600 MHz, CDCl_3_, Me_4_Si): 7.69 (d, *J* = 8.3 Hz, 2H, Ar-*H, conformer B*), 7.64 (d, *J* = 8.3 Hz, 2H, Ar-*H, conformer A*), 7.25 (m, 4H, Ar-*H, both conformers*), 5.07 (m, 1H, N–C*H*–CH_2_, *conformer B*), 4.96 (m, 1H, N–C*H*–CH_2_, *conformer A*), 3.68 (m, 1H, N–C(*H*)H–CH_2_, *conformer B*), 3.62 (m, 1H, N–C(*H*)H–CH_2_, *conformer A)*, 3.39 (m, 2H, N–C(*H*)H–CH_2_, *both conformers*), 2.96–2.90 (m, 2H, C=C-(C(H)*H*)_2_, *conformer B*), 2.90–2.81 (m, 2H, C=C—(C(H)*H*)_2_, *conformer A*), 2.41 (s, 6H, Ar–C*H*_3_, *both conformers*), 2.07–1.79 (m, 12H, C=C—(C(H)*H*)_2_, N–CH_2_–(C*H*_2_)_2_, *both conformers*), 1.75–1.57 (m, 4H, C=C(CH_2_)_2_—C(H)*H*), N–CH_2_–(C*H*_2_)_2_, *both conformers*), 1.32–1.12 (m, 2H, C(*t*-Bu)H–(C(H)*H*)_2_, *both conformers*), 1.06–0.93 (m, 4H, C(*t*-Bu)H–(C(H)*H*)_2_, C(*t*-Bu)*H*–(C(H)*H*)_2_, *both conformers*), 0.86 (s, 9H, (C*H*_3_)_3_, *conformer A*), 0.83 (s, 9H, (C*H*_3_)_3_, *conformer B*). ^13^C{^1^H} NMR (151 MHz, CDCl_3_, Me_4_Si) δ 143.1 (Cq), 143.0 (Cq), 138.4 (Cq), 137.9 (Cq), 137.3 (Cq), 136.7 (Cq), 129.3 (2 × CH), 129.3 (2 × CH), 127.4 (2 × CH), 127.2 (2 × CH), 126.3 (Cq), 126.0 (Cq), 58.9 (CH), 58.8 (CH), 49.5 (CH_2_), 49.4 (CH_2_), 48.1 (CH), 48.0 (CH), 32.6 (Cq), 32.5 (Cq), 31.9 (CH_2_), 31.9 (CH_2_), 31.8 (CH_2_), 31.7 (CH_2_), 31.1 (CH_2_), 31.0 (CH_2_), 28.7 (CH_2_) 27.9 (CH_2_), 27.7 (3 × CH_3_), 27.6 (3 × CH_3_), 27.5 (CH_2_), 27.4 (CH_2_), 25.5 (CH_2_), 25.3 (CH_2_), 21.6 (CH_3_), 21.6 (CH_3_). HRMS (ESI) *m*/*z* [M + H]^+^ calcd for C_22_H_33_ClNO_2_S 410.1915, found 410.1912. IR ν max (neat)/cm^–1^ 2948, 2866, 1596, 1344, 1156, 1092, 664.

#### 2-(1-Chlorovinyl)-3-tosyloxazolidine (**2r**)

Following the described procedure, allene **1r** (0.2 mmol, 51 mg) was reacted to obtain 30 mg of 2-(1-chlorovinyl)-3-tosyloxazolidine **2r** as a colorless oil (EP/Acetone 92/8, 52% yield). ^1^H NMR (600 MHz, CDCl_3_, Me_4_Si) δ 7.75 (d, *J* = 8.3 Hz, 2H, Ar-*H*), 7.33 (d, *J* = 8.0 Hz, 2H, Ar-*H*), 5.72 (dm, *J* = 1.7, 1H, CCl=CH_a_*H*_*b*_), 5.64 (s, 1H, N–C*H*–O), 5.49 (d, *J* = 1.7 Hz, 1H, CCl=C*H*_*a*_H_b_), 3.97 (m, 1H, N–(C*H*_2_)_2_–O), 3.58 (m, 2H, N–(C*H*_2_)_2_–O), 3.50 (m, 1H, N–(C*H*_2_)_2_–O), 2.43 (s, 3H, C*H*_3_). ^13^C{^1^H} NMR (151 MHz, CDCl_3_, Me_4_Si) δ 144.6 (Cq), 138.5 (Cq), 134.5 (Cq), 130.1 (2 × CH), 127.9 (2 × CH), 116.7 (CH_2_), 90.8 (CH), 66.1 (CH_2_), 46.5 (CH_2_), 21.7 (CH_3_). HRMS (ESI) *m*/*z* [M + H]^+^ calcd for C_12_H_15_ClNO_3_S 288.0456, found 288.0455. IR ν max (neat)/cm^–1^ 2921, 1631, 1596, 1344, 1161, 1088, 912, 819, 661.

#### 2-(1-Chlorovinyl)-3-tosyl-2,3-dihydrobenzo[*d*]oxazole (**2s**)

Following the described procedure, allene **1s** (0.2 mmol, 60 mg) was reacted to obtain 34.2 mg of 2-(1-chlorovinyl)-3-tosyl-2,3-dihydrobenzo[*d*]oxazole **2s** as a colorless solid (EP/acetone 95:5, 51% yield). ^1^H NMR (600 MHz, CDCl_3_, Me_4_Si) δ 7.49 (dd, *J* = 7.8, 1.4 Hz, 1H, Ar-*H*), 7.45–7.40 (m, 2H, Ar–H), 7.13–7.08 (m, 2H, Ar-*H*), 6.97 (td, *J* = 7.8, 1.4 Hz, 1H, Ar-*H*), 6.89 (td, *J* = 7.7, 1.2 Hz, 1H, Ar-*H*), 6.63 (dd, *J* = 7.9, 1.1 Hz, 1H, Ar-*H*), 6.20 (bs, 1H, O–C*H*–N), 5.75 (dd, *J* = 2.0, 1.0 Hz, CCl=C(*H*)H), 5.43 (d, *J* = 2.1 Hz, 1H, CCl=C(*H*)H), 2.29 (s, 3H, *CH*_3_). ^13^C{^1^H} NMR (151 MHz, CDCl_3_, Me_4_Si) δ 151.2 (Cq), 145.3 (Cq), 136.6 (Cq), 132.9 (Cq), 130.0 (2 × CH), 128.8 (Cq), 127.7 (2 × CH), 127.0 (CH), 122.2 (CH), 117.9 (CH), 117.0 (CH_2_), 109.9 (CH), 94.2 (CH), 21.7 (CH_3_). HRMS (ESI) *m*/*z* [M + H]^+^ calcd for C_16_H_15_ClNO_3_S 336.0456, found: 336.0453. IR ν max (neat)/cm^–1^ 2924, 1635, 1595, 1477, 1165, 671. mp 142.1–143.6 °C.

#### 2-(1-Chlorovinyl)-3-(methylsulfonyl)-2,3-dihydrobenzo[*d*]oxazole (**2t**)

Following the described procedure, allene **1t** (0.2 mmol, 45 mg) was reacted to obtain 25 mg of 2-(1-chlorovinyl)-3-(methylsulfonyl)-2,3-dihydrobenzo[*d*]oxazole **2t** as a colorless oil (EP/acetone 95:5, 48% yield). ^1^H NMR (600 MHz, CDCl_3_, Me_4_Si): 7.39 (dd, *J* = 7.8, 1.3 Hz, 1H, Ar-*H*), 7.13 (td, *J* = 7.8, 1.3 Hz, 1H, Ar-*H*), 6.99 (td, *J* = 7.8, 1.1 Hz, 1H, Ar-*H*), 6.95 (dd, *J* = 8.0, 1.1 Hz, 1H, Ar-*H*), 6.40 (d, *J* = 0.7 Hz, 1H, O–C*H*–N), 5.81 (dd, *J* = 2.1, 0.9 Hz, 1H, CCl=C(*H*)H), 5.53 (d, *J* = 2.1 Hz, 1H, CCl=C(*H*)H), 2.85 (s, 3H, C*H*_3_). ^13^C{^1^H} NMR (151 MHz, CDCl_3_, Me_4_Si): 150.9 (Cq), 136.4 (Cq), 128.7 (Cq), 127.0 (CH), 122.6 (CH), 117.3 (CH_2_), 116.9 (CH), 110.2 (CH), 94.3 (CH), 36.5 (CH_3_). HRMS (ESI) *m*/*z* [M + H]^+^ calcd for C_10_H_11_ClNO_3_S 260.0143, found 260.0139. IR ν max (neat)/cm^–1^ 2933, 2888 1633, 1470, 1346, 1162, 1054, 742.

#### 2-(1-Chlorovinyl)-3-tosyl-2,3-dihydrobenzo[*d*]thiazole (**2u**)

Following the described procedure, allene **1u** (0.2 mmol, 65 mg) was reacted to obtain 19 mg of 2-(1-chlorovinyl)-3-tosyl-2,3-dihydrobenzo[*d*]thiazole**2u** as a colorless oil (EP/acetone 95:5, 27% yield). ^1^H NMR (600 MHz, CDCl_3_, Me_4_Si): 7.69 (s, 1H, Ar-*H*) 7.46 (d, *J* = 8.2 Hz, 2H, Ar-*H*), 7.15 (d, *J* = 8.2 Hz, 2H, Ar-*H*), 7.08 (s, 1H, Ar-*H*), 7.03 (s, 1H. Ar-*H*), 6.07 (s, 1H, S–C*H*–N), 5.68 (s, 1H, CCl=C(*H*)H), 5.36 (s, 1H, CCl=C(*H*)H), 2.36 (s, 3H, C*H*_3_). ^13^C{^1^H} NMR (151 MHz, CDCl_3_, Me_4_Si) δ 145.0 (Cq), 139.0 (Cq), 137.2 (Cq), 134.0 (Cq), 132.1 (Cq), 129.7 (2 × CH), 127.2 (2 × CH), 127.5 (CH), 125.8 (CH), 122.6 (CH), 120.5 (CH), 114.2 (CH_2_), 70.8 (CH), 21.7 (CH_3_). HRMS (ESI) *m*/*z* [M + H]^+^ calcd for C_16_H_15_ClNO_2_S_2_ 352.0227, found 352.0224. IR ν max (neat)/cm^–1^ 2992, 1554, 1469, 1455, 1156, 680.

## Supporting Information Available

Details on the computational method, additional discussions, absolute and relative energies, pictures of all structures (with imaginary frequencies for the TSs) and relative Cartesian coordinates (PDF)
Experimental procedures (PDF)
