# Supplementary material for: Photoinduced Chloroamination Cyclization Cascade with N-Chlorosuccinimide: From N-(Allenyl)sulfonylamides to 2-(1-Chlorovinyl)pyrrolidines
Source: J Org Chem. 2022 Oct 26;88(10):6420–33. doi: 10.1021/acs.joc.2c01963 (PMC10204091; doi:10.1021/acs.joc.2c01963)
Supplement: Supplementary file 1 — jo2c01963_si_001.pdf [file jo2c01963_si_001.pdf]

Supporting Information - Computational Data

Photoinduced Chloroamination Cyclization Cascade with *N*-Chlorosuccinimide: from *N*-(Allenyl)sulfonylamides to 2-(1-Chlorovinyl)pyrrolidines.

Emanuele Azzi, Giovanni Ghigo, Lorenzo Sarasino, Stefano Parisotto, Riccardo Moro, Polyssena Renzi* and Annamaria Deagostino

Department of Chemistry, University of Torino, Via P. Giuria 7, 10125, Turin, Italy

Corresponding author's email address: polyssena.renzi@unito.it

Table of Content

§ 1 Computational method. S-2

§ 2 The conversion of the allene **1a** to the *N*-chloroallene **3a**. S-3

§ 3 The generation of the NCR **1a$^{\bullet}$** from the photolysis of the *N*-chloro-allene **3a**. S-6

§ 4 The radical reaction mechanism from the *N*-Centered Radical **1a$^{\bullet}$**. S-11

§ 5 Pictures and Cartesian coordinates of optimized structures M06/def2-TZVP. S-16

§ 1 Computational method.

The structures of the reactants, intermediates and transition states have been optimized by using the density functional method (DFT)[1] with the functional M06[2,3] and the basis sets def2-SVP.[4] The electronic energy values where refined by re-optimization with the basis set def2-TZVP.[5] The nature of the critical points was characterized by using vibrational analysis[6] which also furnished the Zero Point Energies (ZPE) and entropies for the calculations of the Free Energies. These have been converted from the gas phase to the 1 M standard state at 1 atm and 298.15 K.[7] The solvent effects (toluene) were introduced in all calculations using the universal solvation model (SMD) by Truhalr *et al.*[8] The real environment is not the pure toluene but a more polar environment because of the presence of large amounts (1/4) of methylformiate for which, unfortunately, the solvent parameters are not available. The rate constants of the title reactions are calculated using canonical transition state theory(CTST):[9]

$$k = \sigma \bullet (k_B \bullet T / h_P) \bullet c \bullet \exp(-\Delta G^{\ddagger} / RT)$$

in which σ is the symmetry number, k_B the Boltzmann constant, h_P the Planck constant, c is 1 for monomolecular reactions and RT for bimolecular reactions. The singlet excited states have been optimized with the Time-Dependet DFT.[10] The calculations were performed by the quantum package Gaussian 16-A.03[11] The figures were obtained using the graphical program Molden.[12]

[1] R. G. Parr, Density Functional Theory of Atoms and Molecules, in: Horizons Quantum Chem., Springer Netherlands, 1980: pp. 5–15. DOI:10.1007/978-94-009-9027-2_2.

[2] Y. Zhao, D.G. Truhlar, The M06 suite of density functionals for main group thermochemistry, thermochemical kinetics, noncovalent interactions, excited states, and transition elements: Two new functionals and systematic testing of four M06-class functionals and 12 other function, *Theor. Chem. Acc.* 120 (2008) 215–241, DOI: 10.1007/s00214-007-0310-x.

[3] Y. Zhao, D.G. Truhlar, Density functionals with broad applicability in chemistry, *Acc. Chem. Res.* 41 (2008) 157–167, DOI: 10.1021/ar700111a.

[4] A. Schaefer, H. Horn, and R. Ahlrichs, "Fully optimized contracted Gaussian-basis sets for atoms Li to Kr," *J. Chem. Phys.*, 97 (1992) 2571–2577. DOI: 10.1063/1.463096.

[5] A. Schaefer, C. Huber, and R. Ahlrichs, "Fully optimized contracted Gaussian-basis sets of triple zeta valence quality for atoms Li to Kr," *J. Chem. Phys.*, 100 (1994) 5829–5835. DOI: 10.1063/1.467146 .

[6] J. Foresman, A. Frisch, Exploring chemistry with electronic structure methods, 1996, Gaussian Inc, Pittsburgh, PA, 1996, http://gaussian.com/expchem3/(accessed June 4, 2021).

[7] R.F. Ribeiro, A.V. Marenich, C.J. Cramer, D.G. Truhlar, Use of solution-phase vibrational frequencies in continuum models for the free energy of solvation, *J. Phys. Chem. B.* 115 (2011) 14556–14562, DOI: 10.1021/jp205508z.

[8] A. V. Marenich, C. J. Cramer, and D. G. Truhlar, "Universal solvation model based on solute electron density and a continuum model of the solvent defined by the bulk dielectric constant and atomic surface tensions," *J. Phys. Chem. B*, 113 (2009) 6378–6396. DOI: 10.1021/jp810292n .

[9] D. G. Truhlar, B. C. Garrett, and S. J. Klippenstein, Current Status of Transition-State Theory, *J. Phys. Chem.* **1996**, *100*, 12771–12800. DOI: 10.1021/jp953748q

[10] F. Furche and R. Ahlrichs, "Adiabatic time-dependent density functional methods for excited state properties," *J. Chem. Phys.*, 117 (2002) 7433–7447. DOI: 10.1063/1.1508368

[11] D.J. Frisch, M. J.; Trucks, G. W.; Schlegel, H. B.; Scuseria, G. E.; Robb, M. A.; Cheeseman, J. R.; Scalmani, G.; Barone, V.; Petersson, G. A.; Nakatsuji, H.; Li, X.; Caricato, M.; Marenich, A. V.; Bloino, J.; Janesko, B. G.; Gomperts, R.; Mennucci, B.; Hratch, Gaussian 16, Revision A.03, (2016).

[12] G. Schaftenaar, J.H. Noordik, Molden: A pre- and post-processing program for molecular and electronic structures, J. Comput. Aided. Mol. Des. 14 (2000) 123–134, DOI: 10.1023/A:1008193805436.

§ 2 The conversion of the allene 1aa to the *N*-chloroallene 3.

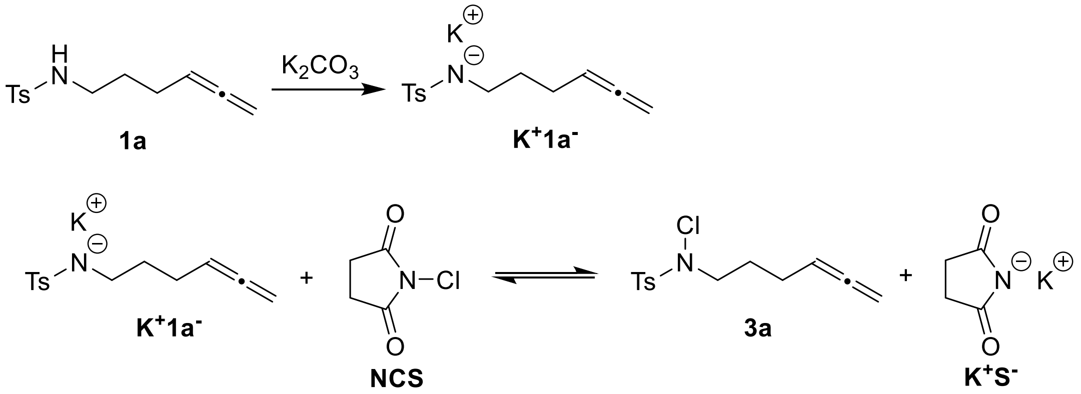

Scheme S-1. The conversion of the allene **1a** into the *N*-Chloro allene **3a**.

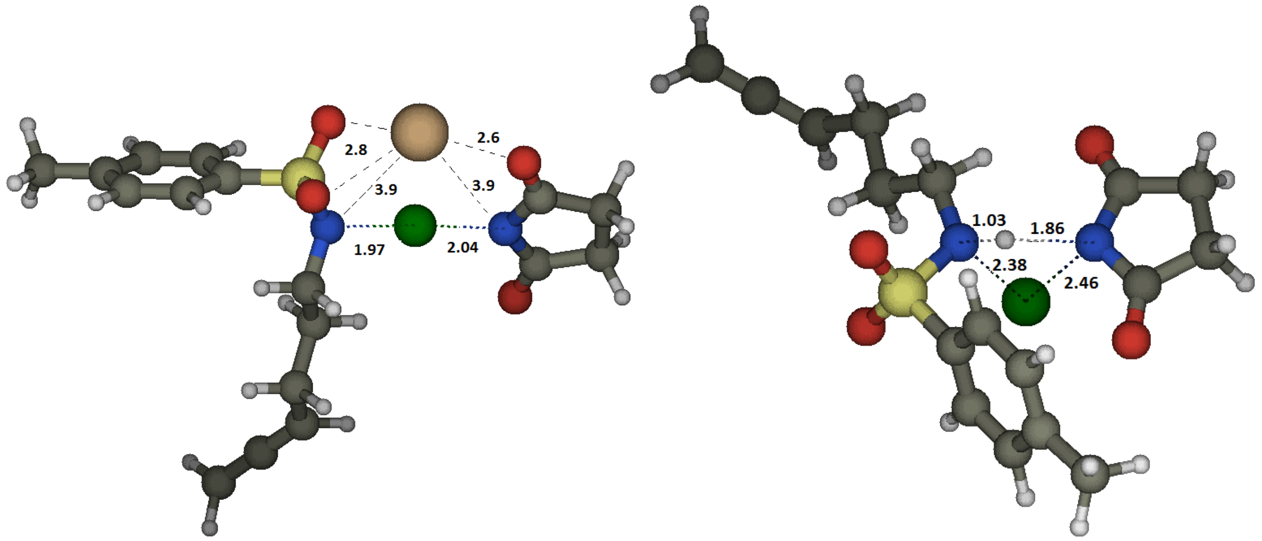

Figure S-1. The transition structures for the generation of the *N*-chloro-allene **3a** from the reaction of NCS with the potassium-allene **K$^+$1a$^-$** (left) and with the allene **1a** (right)..

Table S-1a. Calculated absolute and relative (in kcal mol^{-1}) energies of the deprotonation of **1** by K_2CO_3.

**Table S-1a**		E /au[a]	ΔE	ZPE / au[a]	δG / au[a]	E TZ(D)/ au[b]	ΔE	E^{0K} /au[c]	ΔE^{0K}	G TZ(D) / au[c]	Δn	ΔG^{298K}
Allene	**1a**	-1107.771260		0.274690	0.224308	-1108.720750		-1108.446060		-1108.496442		
K_2CO_3		-1463.307662		0.016895	-0.016459	-1463.744232		-1463.727337		-1463.760691		
**K_2CO_3 + Allene**		-2571.078922	**0.00**	0.291585	0.207849	-2572.464982	**0.00**	-2572.173397	**0.00**	-2572.257133	**0**	**0.00**
$KHCO_3$		-864.025213		0.027832	-0.002201	-864.386440		-864.358608		-864.388641		
K^+-Allene-Anion	**K^+1a^-**	-1707.060127		0.262633	0.209330	-1708.085874		-1707.823241		-1707.876544		
**$KHCO_3$ + Allene Anion K**		-2571.085340	**-4.03**	0.290465	0.207129	-2572.472314	**-4.60**	-2572.181849	**-5.30**	-2572.265185	**0**	**-5.05**

[a] M06/def2-SVP; [b] M06/def2-TZVP; [c] M06/def2-TZVP enegies combined with thermal corrections M06/def2-SVP.

Table S-1b. Calculated absolute and relative (in kcal mol^{-1}) energies of the generation of **3a** from **K^+1a^-** and NCS.

**Table S-1b**		E /au[a]	ΔE	ZPE / au[a]	δG / au[a]	E TZ(D)/ au[b]	ΔE	E^{0K} /au[c]	ΔE^{0K}	G TZ(D) / au[c]	Δn	ΔG^{298K}
K^+-Allene-Anion	**K^+1a^-**	-1707.060127		0.262633	0.209330	-1708.085874		-1707.823241		-1707.876544		
N-ChloroSuccinimmide	**NCS**	-819.625317		0.081360	0.048204	-820.161245		-820.079885		-820.113041		
**AK + NCS**		-2526.685444	**0.00**	0.343993	0.257534	-2528.247119	**0.00**	-2527.903126	**0.00**	-2527.989585	**0**	**0.00**
**TS_{Cl-Exc} K^+-Allene + NCS**		-2526.706675	**-13.32**	0.344886	0.280441	-2528.261045	**-8.74**	-2527.916159	**-8.18**	-2527.980604	**-1**	**3.74**
N-ChloroAllene	**3a**	-1567.173516		0.264483	0.213056	-1568.270206		-1568.005722		-1568.057149		
K^+-Succinimmide	**K^+S^-**	-959.515812		0.079820	0.045996	-959.978366		-959.898546		-959.932370		
***N*-ChloroAllene + KSA**		-2526.689329	**-2.44**	0.344304	0.259053	-2528.248572	**-0.91**	-2527.904268	**-0.72**	-2527.989519	**0**	**0.04**

[a] M06/def2-SVP; [b] M06/def2-TZVP; [c] M06/def2-TZVP enegies combined with thermal corrections M06/def2-SVP.

Table S-1c. Calculated absolute and relative (in kcal mol^{-1}) energies of the of **3a** from **1a** and NCS.

**Table S-1c**		E /au[a]	ΔE	ZPE / au[a]	δG / au[a]	E TZ(D)/ au[b]	ΔE	E^{0K} /au[c]	$ΔE^{0K}$	G TZ(D) / au[c]	Δn	$ΔG^{298K}$
Allene	**1a**	-1107.771260		0.274690	0.224308	-1108.720750		-1108.446060		-1108.496442		
N-ChloroSuccinimmide	**NCS**	-819.625317		0.081360	0.048204	-820.161245		-820.079885		-820.113041		
**Allene + NCS**		-1927.396577	**0.00**	0.356050	0.272512	-1928.881995	**0.00**	-1928.525945	**0.00**	-1928.609483	**0**	**0.00**
TS_{Cl-Exc} **Allene + NCS**		-1927.297455	**62.20**	0.353799	0.292455	-1928.772589	**68.65**	-1928.418790	**67.24**	-1928.480134	**-1**	79.27
N-ChloroAllene	**3a**	-1567.173516		0.264483	0.213056	-1568.270206		-1568.005722		-1568.057149		
Succinimmide	**SH**	-360.224868		0.091370	0.060806	-360.611951		-360.520581		-360.551145		
***N*-ChloroAllene + SA**		-1927.398384	**-1.13**	0.355853	0.273862	-1928.882157	**-0.10**	-1928.526304	**-0.23**	-1928.608295	**0**	**0.75**

[a] M06/def2-SVP; [b] M06/def2-TZVP; [c] M06/def2-TZVP enegies combined with thermal corrections M06/def2-SVP.

§ 3 The generation of the NCR 1a˙ from the photolysis of the *N*-chloro-allene 3a.

The experiments demonstrated that the reaction can take place even without the photocatalyzer but in the presence of the light. This would be possible if we consider an alternative pathway that involves the *N*-chloro-allene **3a** (Figure S-2a, left) that can be generated from NCS and the allene **1a** in the presence of the base. The homolysis of the N–Cl bond is thermodynamically prohibitive (ΔG = 43.7 kcal mol^{-1}, Table S-2a) but the VIS-UV spectrum of *N*-chloro-allene **3a** shows a weak absorption below 350 nm that can activate the molecule. We verified this hypothesis by TD-DFT calculations and we found that the first excited state of **3** presents a complicated electronic structure that involves several MOs in the electronic transition. The differential density map (Figure S-2a, right) shows that it corresponds to an electronic transition from the n_N and some of the π orbitals mainly to the σ^* of the N–Cl bond.

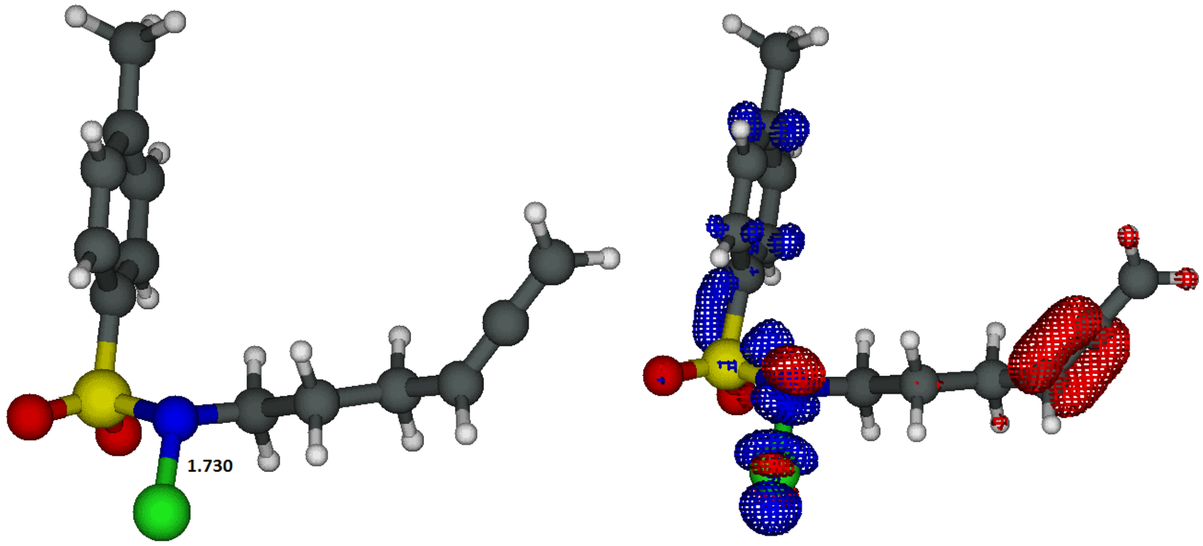

Figure S-2a. Molecular structure of the *N*-chloro-allene **3a** (left) and differential density map of its first excited state (right, the red areas correspond to a reduction in the electronic density when going from S_0 to S_1, and the blue areas correspond to an increase in the electronic density).

As a consequence of the population of the σ_{NCl}^*, the optimization of the S_1 state (located 62.4 kcal mol^{-1} above **3a** in term of energy, ΔE, Table S-2b) leads to an elongation of the N–Cl bond from 1.730 to 2.355 Å (Figure S-2b, left) that can prelude to its dissociation. The electronic structure of the optimized S_1 state sees a large population of the σ_{NCl}^* (Figure S-2b, right).

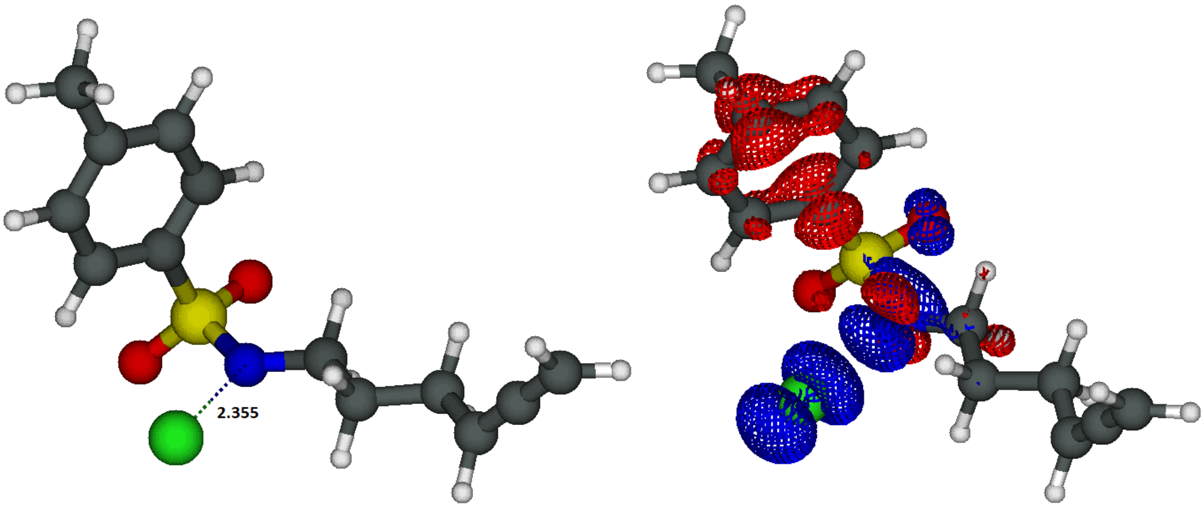

Figure S-2b. Molecular structure (left) and differential density map of the first excited state S_1 of the *N*-chloro-allene **3a** (right, the red areas correspond to a reduction in the electronic density when going from S_0 to S_1, and the blue areas correspond to an increase in the electronic density.

Serious problem in the convergence of the TD-DFT calculations prevented to study the potential energy surface of the S_1 state. Fortunately, the first triplet state T_1 (Table S-2c, ΔE = 40.9 and ΔG = 36.6 kcal mol^{-1} with respect to **3a**) shows molecular (Figure S-2c, left) and electronic structures (Figure S-2c, right, the spin density map corresponds to a $n_N \rightarrow \sigma_{NCl}$* transition) similar to that of the S_1 state.

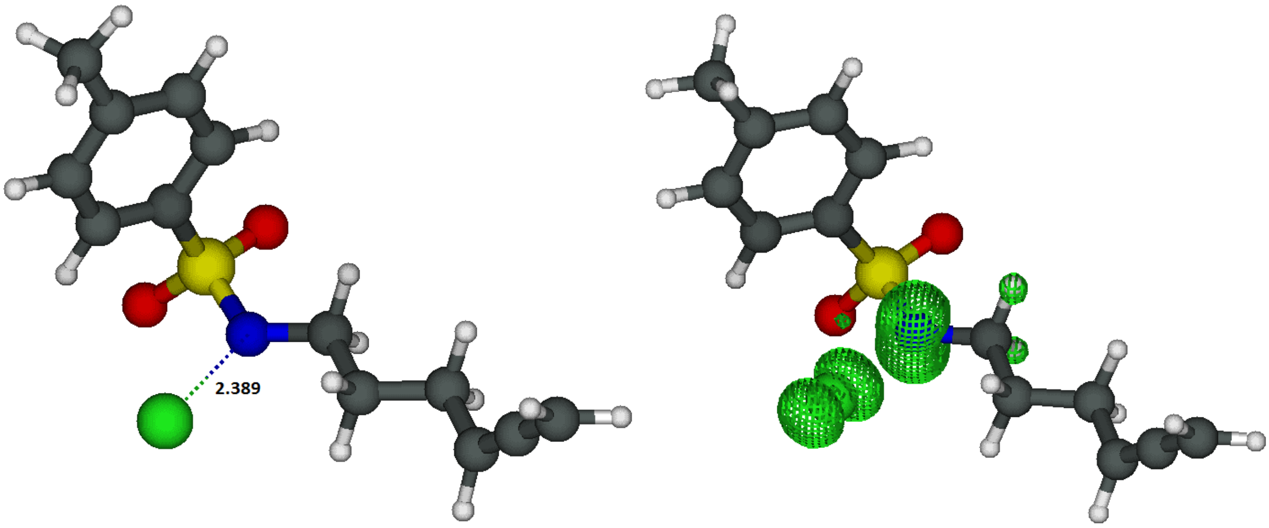

Figure S-2c. Molecular structure (left) and spin density map of the first excited triplet state T_1 of the *N*-chloro-allene **3a** (right).

The T_1 state of the *N*-chloro-allene **3a** can easily break the N–Cl bond (Figure S-2d, left, $\Delta G^‡$ = 7.1 kcal mol^{-1} with respect to the T_1 state) to form a complex (Figure S-2d, right, ΔG = 1.5 kcal mol^{-1}) between the chlorine radical and the allenyl *N*-radical **1a•** that can dissociates to the separated radicals (ΔG = -3.0 kcal mol^{-1}). **1a•** is the intermediate that start the radical chain that lead to the final products as illustrate above (see § 4).

Because the T_1 and the S_1 states share similar molecular and electronic structures we believe that the easy photo-dissociation of the N–Cl bond that we calculated for the T_1 state can be extended to the S_1 with the advantage that from this state, being 21.5 kcal mol^{-1} above the T_1 state, the dissociation to the separated radicals is energetically more favored (ΔE = -22.0 kcal mol^{-1} with respect to S_1)

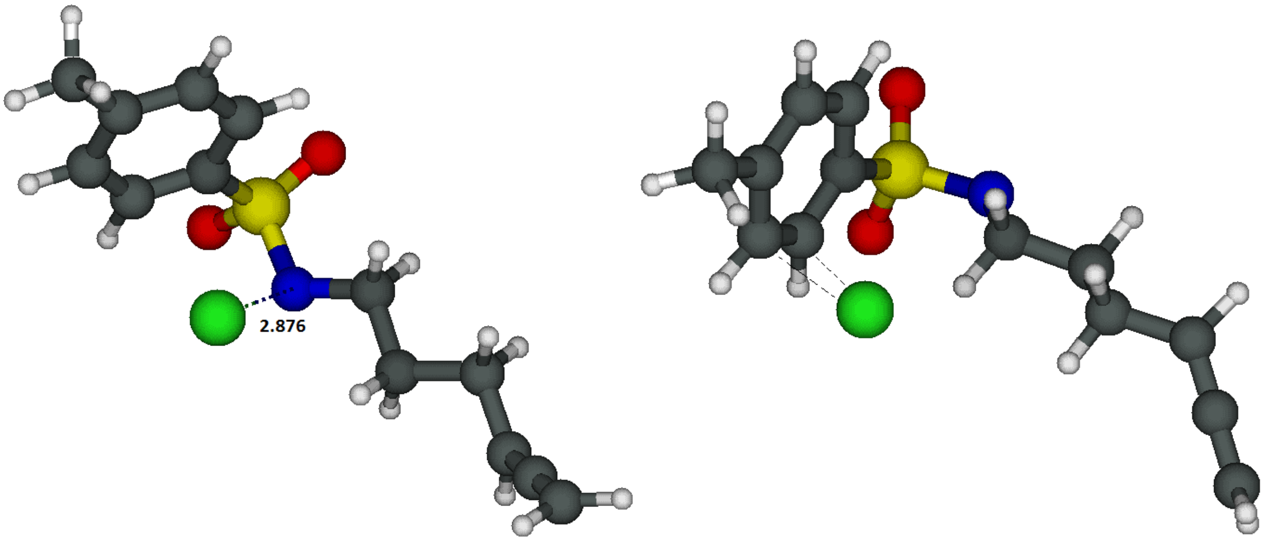

Figure S-2d. Transition structure (left) and radicals couple (right) of the N–Cl bond break from the first excited triplet state T_1 of the *N*-chloro-allene **3a**.

Table S-2a. Calculated absolute and relative (in kcal mol^{-1}) energies of N–Cl bond homolysis in **3a**.

**Table S-2a**		E /au^{a}	**ΔE**	ZPE / au^{a}	δG / au^{a}	E TZ(D)/ au^{b}	**ΔE**	E^{0K} /au^{c}	**$ΔE^{0K}$**	G TZ(D) / au^{c}	**Δn**	**$ΔG^{298K}$**
N-ChloroAllene	**3a**	-1567.173516	**0.00**	0.264483	0.213056	-1568.270206	**0.00**	-1568.005722	**0.00**	-1568.057149	**0**	**0.00**
Cl*		-459.985647		0.000000	-0.015677	-460.129905		-460.129905		-460.145582		
De-H^{+}-Allene Rad.	**$1a^{•}$**	-1107.103002		0.260254	0.208013	-1108.052955		-1107.792701		-1107.844942		
**Cl* + Allene Rad.**		-1567.088649	**53.25**	0.260254	0.192336	-1568.182860	**54.81**	-1567.922605	**52.16**	-1567.990523	**1**	**43.70**

^{a} M06/def2-SVP; ^{b} M06/def2-TZVP; ^{c} M06/def2-TZVP enegies combined with thermal corrections M06/def2-SVP.

Table S-2b. Calculated absolute and relative (in kcal mol^{-1}) energies of the first singlet excited state S_1 of **3a**.

**Table S-2b**		E /au^{a}	**ΔE**	ZPE / au^{a}	δG / au^{a}	E TZ(D)/ au^{b}	**ΔE**	E^{0K} /au^{c}	**$ΔE^{0K}$**	G TZ(D) / au^{c}	**Δn**	**$ΔG^{298K}$**
N-ChloroAllene	**3a**	-1567.173516	**0.00**	0.264483	0.213056	-1568.270206	**0.00**	-1568.005722	**0.00**	-1568.057149	**0**	**0.00**
**S_1 *N*CA $^{1}3a^{*}$**	**$^{1}3a^{*}$**	-1567.077485	**60.26**			-1568.170782	**62.39**					

^{a} M06/def2-SVP; ^{b} M06/def2-TZVP; ^{c} M06/def2-TZVP enegies combined with thermal corrections M06/def2-SVP.

Table S-2c. Calculated absolute and relative (in kcal mol^{-1}) energies of the first triplet excited state T_1 of **3a**.

**Table S-2c**		E /au	ΔE	E^{0K} /au	G^{298K} /au	E TZ(D)/ au	ΔE	E^{0K} /au	ΔE^{0K}	G TZ(D) / au		ΔG^{298K}
**Triplet *N*-ChloroAllene**	^{3}**3a**	-1567.113094	**37.92**	0.261509	0.206204	-1568.205077	**40.87**	-1567.943568	**39.00**	-1567.998873	**0**	**36.57**
**Triplet *N*-ChloroAllene**		-1567.113094	**0.00**	0.261509	0.206204	-1568.205077	**0.00**	-1567.943568	**0.00**	-1567.998873	**0**	**0.00**
**$TS_{Cl-Det.}$ *vs* ^{3}3**		-1567.102606	**6.58**	0.260479	0.207413	-1568.195016	**6.31**	-1567.934537	**5.67**	-1567.987603	**0**	**7.07**
**Cpl [Cl* * 1*] *vs* ^{3}3**		-1567.110364	**1.71**	0.260965	0.207661	-1568.204066	**0.63**	-1567.943101	**0.29**	-1567.996405	**0**	**1.55**
De-H^{+}-Allene Rad.	**1a$^{•}$**	-1107.103002		0.260254	0.208013	-1108.052955		-1107.792701		-1107.844942		
**Cl* + 1* *vs* ^{3}3**		-1567.088649	**15.34**	0.260254	0.192336	-1568.182860	**13.94**	-1567.922605	**13.15**	-1567.990523	**1**	**7.13**
Toluene		-271.153085		0.126607	0.095949	-271.445817		-271.319210		-271.349868		
**Cpl [Cl* * 1*] + Toluene**		-1838.263449	**1.71**	0.387572	0.303610	-1839.649883	**0.63**	-1839.262311	**0.29**	-1839.346273	**0**	**1.55**
Cpl ^{2}[Cl* * Toluene]		-731.164442		0.127239	0.092613	-731.598662		-731.471423		-731.506049		
**Cpl ^{2}[Cl* * Toluene] + 1***		-1838.267444	**-0.79**	0.387493	0.300626	-1839.651617	**-0.45**	-1839.264124	**-0.84**	-1839.350991	**0**	**-2.96**
**1* + Cl-T vs ^{1}3* + Toluene**		-0.036874	**- 23.14**			-0.035018	**- 21.97**					

^{a} M06/def2-SVP; ^{b} M06/def2-TZVP; ^{c} M06/def2-TZVP enegies combined with thermal corrections M06/def2-SVP.

§ 3 The radical reaction mechanism from the *N*-Centere Radical 1a•.

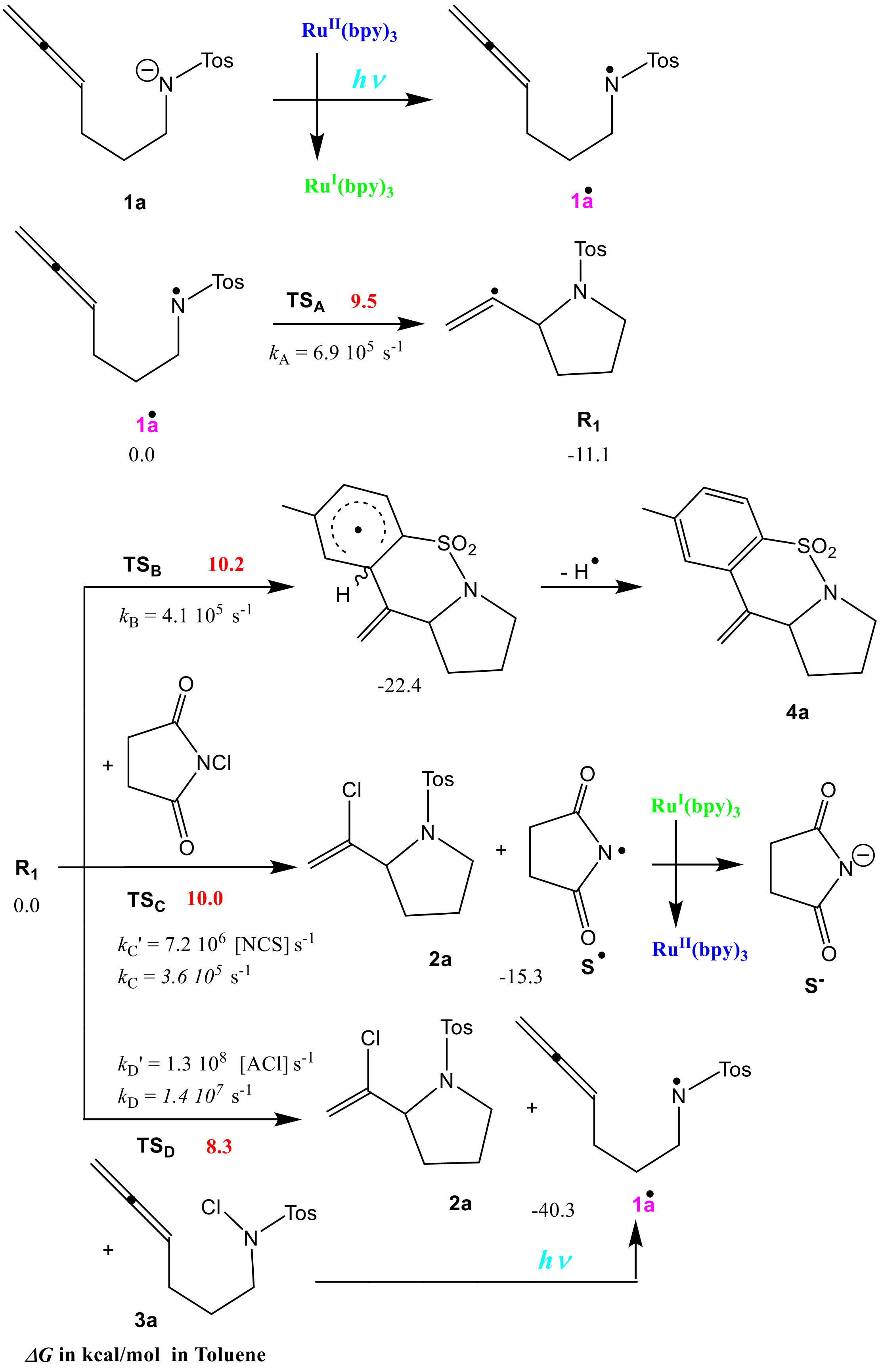

Scheme S-2. The radical reaction mechanism.

The starting radical is the NCR from the allene **1a˙**, generated by the oxidation of the deprotonated allene **1a⁻** by the photoexcited $^{3}Ru^{II}(bpy)_3$ or by the photodissociation of **3a**. The first step (Scheme S-2, Table S-3) is the formation of the pyrrole ring through an intramolecular radical addition (**TS$_A$**, Figure S-3(a) of the *N*-centered radical to the allene moiety generating the highly reactive vinyl radical **R$_1$**. The step is fast ($\Delta G^{\ddagger}$ = 9.5 kcal mol^{-1}, $k_A = 6.9 \cdot 10^5$ s^{-1}) and thermodynamically favored (ΔG = -11.1 kcal mol^{-1}).

From the radical intermediate **R$_1$** three pathways open: the intramolecular cyclization (**TS$_B$**, Figure S-3(b), $\Delta G^{\ddagger}$ = 10.2 kcal mol^{-1}, $k_B = 4.1 \cdot 10^5$ s^{-1}, ΔG = -22.4 kcal mol^{-1}) towards to the tolyl moiety yielding the precursor of the product **4a**; the radical Chlorine-Atom-Transfer (CAT) from the NCS (**TS$_C$**, Figure S-3(c), $\Delta G^{\ddagger}$ = 10.0 kcal mol^{-1}, $k_C' = 7.2 \cdot 10^6 \cdot$ [NCS] s^{-1}, ΔG = -15.3 kcal mol^{-1}) yielding the main product **2a** and the succinimin-*N*-yl **S˙** (the latter will be reduced to the anion by $^{2}Ru^{I}(bpy)_3$ regenerating the photocatalyzer); the CAT from the *N*-chloro allene **3aa** (**TS$_D$**, Figure S-3(d),, $\Delta G^{\ddagger}$ = 8.3 kcal mol^{-1}, $k_D' = 1.3 \cdot 10^8 \cdot$ [ACl] s^{-1}, ΔG = -40.3 kcal mol^{-1}) also yielding the main product **2a** and a new NCR **1a˙**. Both CAT are bimolecular processes that require the presence of the chlorine donor. A part for the obvious choice of NCS, the experimental and computational study suggested the this role can also be assumed by **3a** easily formed in the reaction environment (see above). On the basis of the experimental data (Table S7, entry 6) we can estimate the initial concentrations of NCS and **3** as 0.18 • 3 • 0.5 M = 0.05 M and 0.82 • 3 • 0.5 M = 0.123 M, respectively. Multiplying the corresponding bimolecular rate constants for these concentrations we obtain the rate constants $k_C = 1.9 \cdot 10^5$ and $k_D = 1.7 \cdot 10^7$ s^{-1}. From the sum of these two rate constants and k_B we can estimate a yield of the main product **2** of 98%. The formation of the tricyclic product **4a** is estimated to be marginal (2%) in agreement with the experimental findings (Table S8, entry 1, by-product).

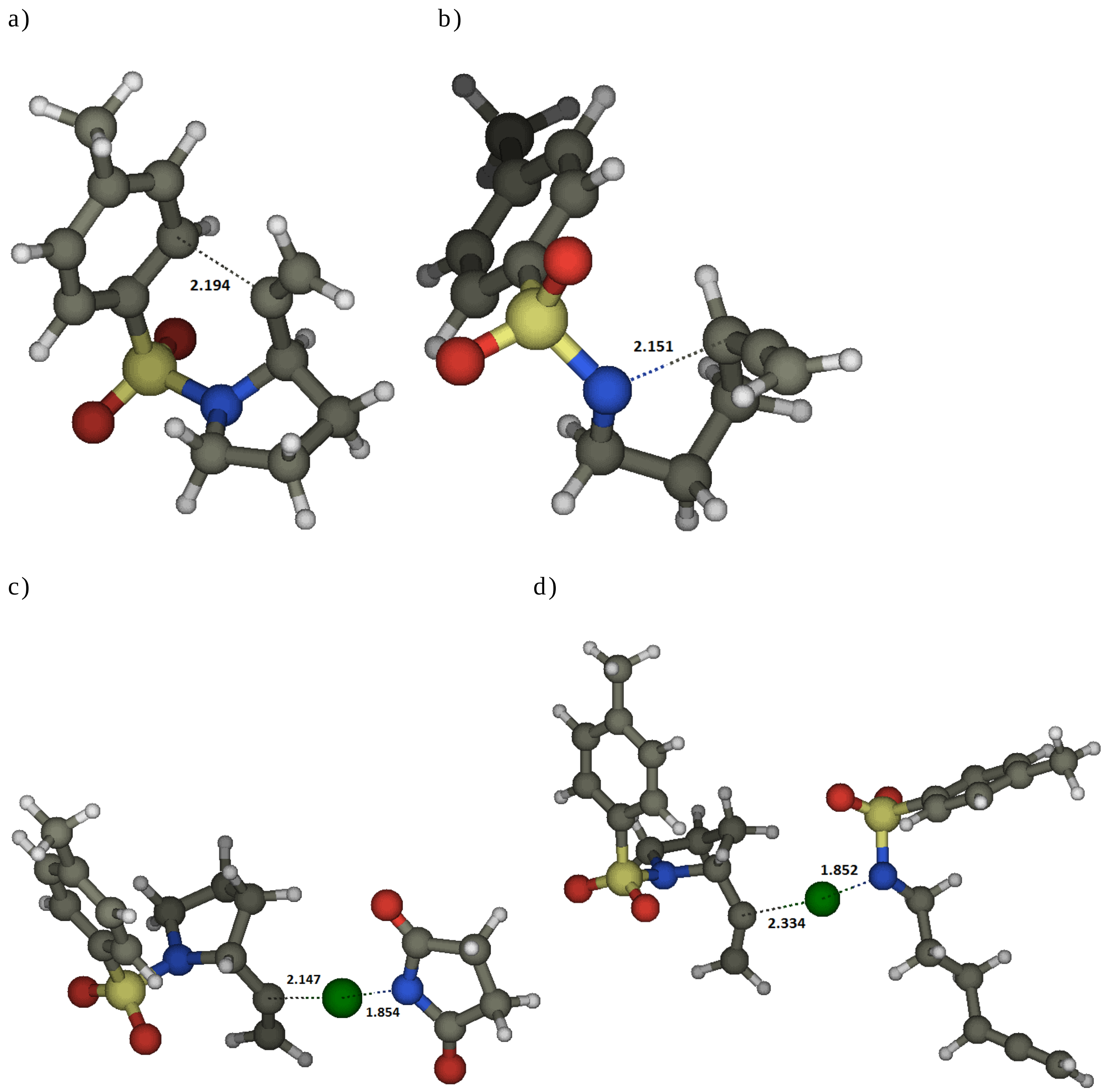

Figure S-3. Transition structures: (a) $\mathbf{TS_A}$ for the intramolecular cyclization of $\mathbf{1a}^{\cdot}$ yielding the 1-(*N*-tosyl-pyrrol-1-yl)vinyl radical $\mathbf{R_1}$; (b) $\mathbf{TS_B}$ for the intramolecular cyclization of $\mathbf{R_1}$ yielding the precursor of the product **2a**.; (c) $\mathbf{TS_C}$ for the CAT from NCS to $\mathbf{R_1}$; (d) $\mathbf{TS_D}$ for the CAT from **3a** to $\mathbf{R_1}$.

Table S-3. Calculated absolute and relative (in kcal mol^{-1}) energies for the radical reaction mechanism from the *N*-Centere Radical 1a$^{\bullet}$.

Table S-3		E /au[a]	ΔE	ZPE / au[a]	δG / au[a]	E TZ(D)/ au[b]	ΔE	E^{0K} /au[c]	ΔE^{0K}	G TZ(D) / au[c]	Δn	ΔG^{298K}
De-H^{+}-Allene Rad.	**1a$^{\bullet}$**	-1107.103002		0.260254	0.208013	-1108.052955		-1107.792701		-1107.844942		
N-ChloroSuccinimmide	**NCS**	-819.625317		0.081360	0.048204	-820.161245		-820.079885		-820.113041		
**Reacts 1a$^{\bullet}$ + NCS**		-1926.728319	**0.00**	0.341614	0.256217	-1928.214200	**0.00**	-1927.872586	**0.00**	-1927.957983	**0**	**0.00**
TS_A to R_1	**TS_A**	-1107.096344		0.261053	0.214031	-1108.043854		-1107.782801		-1107.829823		
**TS_A to R_1 + NCS**		-1926.721661	**4.18**	0.342413	0.262235	-1928.205099	**5.71**	-1927.862687	**6.21**	-1927.942865	**0**	**9.49**
R_1	**R_1**	-1107.136071		0.264639	0.217696	-1108.080292		-1107.815654		-1107.862597		
**R_1 + NCS**		-1926.761387	**-20.75**	0.345998	0.265899	-1928.241537	**-17.15**	-1927.895539	**-14.40**	-1927.975638	**0**	**-11.08**
TS_B to Rad. **4a'**	**TS_B**	-1107.124927	**6.99**	0.264682	0.221541	-1108.067868	**7.80**	-1107.803186	**767.50**	-1107.846327		**10.21**
**TS_B to Rad. 4a' + NCS**		-1926.750244	**-13.76**	0.346042	0.269745	-1928.229113	**-9.36**	-1927.883071	**-6.58**	-1927.959368	**0**	**-0.87**
Rad. 4' *Vinyl on Tol*	**4a'**	-1107.165672	**-18.58**	0.267701	0.225428	-1108.106012	**-16.14**	-1107.838311		-1107.880584		
**Rad. 4a' + NCS**		-1926.790989	**-39.33**	0.349061	0.273632	-1928.267257	**-33.29**	-1927.918196	**-28.62**	-1927.993625	**0**	**-22.37**
**Cpl [R_1 NCS]**		-1926.769555	**-25.88**	0.346952	0.284250	-1928.247417	**-20.84**	-1927.900465	**-17.49**	-1927.963167	**-1**	**-5.15**
**TS_C R_1 + NCS**	**TS_C**	-1926.763535	**-22.10**	0.345977	0.284442	-1928.241138	**-16.90**	-1927.895162	**-14.17**	-1927.956697	**-1**	**-1.09**
**Cpl [2 S$^{\bullet}$]**		-1926.800262	**-45.15**	0.347683	0.288386	-1928.275956	**-38.75**	-1927.928273	**-34.94**	-1927.987570	**-1**	**-20.46**
Product	**2a**	-1567.271724		0.269461	0.221962	-1568.361033		-1568.091572		-1568.139071		
De-H^{+}-Succinim. Rad. **S$^{\bullet}$**		-359.515755		0.076249	0.045197	-359.906132		-359.829883		-359.860935		
**2a + S$^{\bullet}$**		-1926.787480	**-37.12**	0.345711	0.267160	-1928.267165	**-33.24**	-1927.921455	**-30.67**	-1928.000006	**0**	**-26.37**
N-ChloroAllene	**3a**	-1567.173516		0.264483	0.213056	-1568.270206		-1568.005722		-1568.057149		
**R_1 + 3a**		-2674.309587	**0.00**	0.529122	0.430752	-2676.350498	**0.00**	-2675.821376	**0.00**	-2675.919746	**0**	**0.00**
**Cpl [R_1 3a]**		-2674.319521	**-6.23**	0.530613	0.452878	-2676.357901	**-4.65**	-2673.788908	**-4.65**	-2675.905023	**-1**	**7.34**
**TS_D R_1 + 3a**	**TS_D**	-2674.316791	**-4.52**	0.529530	0.451780	-2676.355352	**-3.05**	-2673.787261	**-3.05**	-2675.903571	**-1**	**8.26**

**Cpl [2 $1^{\bullet}$]**	-2674.382561	**-45.79**	0.531456	0.453750	-2676.420184	**-43.73**	-2673.851105	**-43.73**	-2675.966434	**-1**	**-31.19**
**2 + $1^{\bullet}$**	-2674.374727	**-40.88**	0.529716	0.429976	-2676.413988	**-39.84**	-2675.884272	**-39.84**	-2675.984012	**0**	**-40.33**

[a] M06/def2-SVP; [b] M06/def2-TZVP; [c] M06/def2-TZVP enegies combined with thermal corrections M06/def2-SVP.

§ 4 **Pictures** and **Cartesian coordinates** of optimized structures M06/def2-TZVP.

1a (Table S-1a)

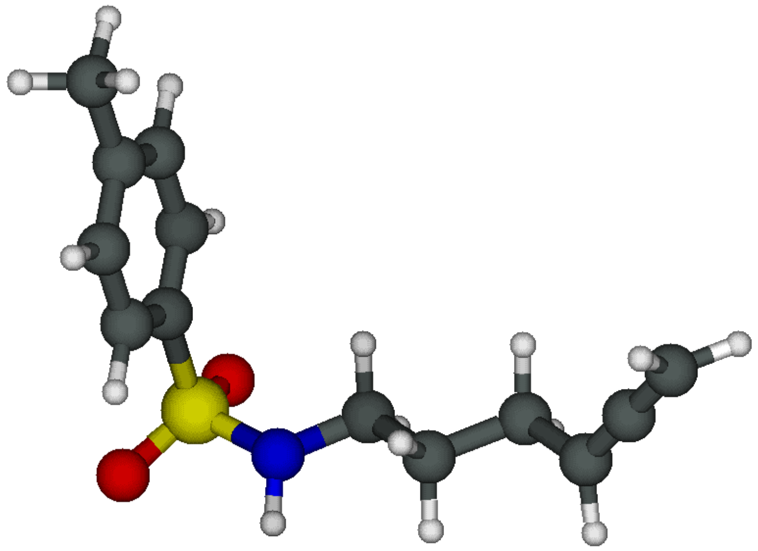

1	7	0	-0.031275	0.001176	0.001970
2	6	0	-0.016092	0.011104	1.457830
3	6	0	1.406896	-0.009253	1.964589
4	1	0	-0.555906	0.877611	1.860446
5	1	0	-0.548162	-0.881964	1.805196
6	6	0	1.466531	0.001879	3.482572
7	1	0	1.921183	-0.893833	1.575346
8	1	0	1.946218	0.861694	1.570792
9	6	0	2.874625	0.037106	3.990162
10	1	0	0.925699	0.880641	3.855468
11	1	0	0.951437	-0.876180	3.883699
12	6	0	3.415345	-0.866007	4.751902
13	1	0	3.485653	0.883761	3.676673
14	6	0	3.947876	-1.768459	5.517278
15	1	0	3.908319	-1.691058	6.599991
16	1	0	4.456625	-2.635514	5.106048
17	16	0	-1.452465	-0.226334	-0.783432
18	1	0	0.516685	0.722717	-0.453454
19	6	0	-1.659836	-1.974227	-0.680925
20	8	0	-2.537216	0.376696	-0.067099
21	8	0	-1.196759	0.145854	-2.142427
22	6	0	-2.691167	-2.509391	0.067815
23	6	0	-2.847280	-3.884951	0.122749
24	6	0	-1.986380	-4.730789	-0.561672
25	6	0	-0.952933	-4.166360	-1.310105
26	6	0	-0.783948	-2.800944	-1.373216
27	1	0	-3.367467	-1.849614	0.597464
28	1	0	-3.656704	-4.308760	0.707838
29	6	0	-2.148318	-6.213069	-0.511593
30	1	0	-0.274498	-4.818786	-1.850970
31	1	0	0.020342	-2.368026	-1.957390
32	1	0	-2.990135	-6.506614	0.116357
33	1	0	-2.313595	-6.622972	-1.511447
34	1	0	-1.248672	-6.693408	-0.118043

K_2CO_3 (Table S-1a)

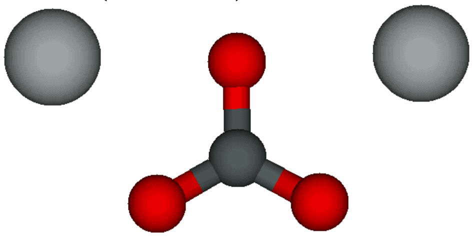

1	6	0	-0.000305	-0.000281	0.008538
2	8	0	-0.000746	-0.000507	1.329558
3	8	0	1.114533	-0.018181	-0.606474
4	8	0	-1.114684	0.018117	-0.607145
5	19	0	2.519308	-0.067382	1.418695
6	19	0	-2.519761	0.066271	1.416953

$KHCO_3$ (Table S-1a)

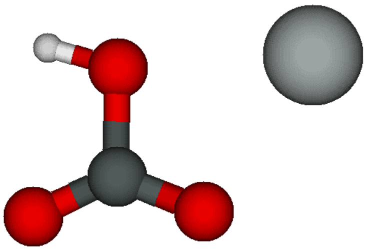

1	6	0	0.000156	-0.000177	0.001881
2	8	0	-0.001972	-0.000679	1.442384
3	8	0	1.155818	0.000585	-0.451329
4	8	0	-1.099491	-0.000524	-0.521021
5	19	0	2.593795	0.002842	1.608746
6	1	0	-0.931444	-0.001376	1.694805

K^+1^- (Table S-1a)

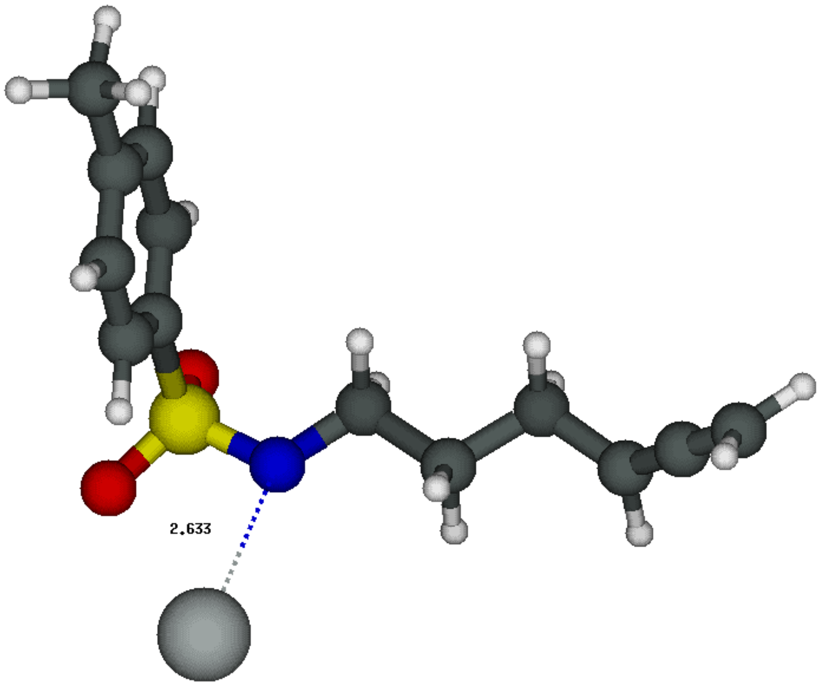

1	7	0	0.002114	-0.072333	-0.004618
2	6	0	-0.000616	-0.063369	1.443339
3	6	0	1.420425	-0.032387	1.962228
4	1	0	-0.546783	0.803684	1.847036
5	1	0	-0.504241	-0.956500	1.856008
6	6	0	1.491232	-0.053993	3.479716
7	1	0	1.973717	-0.888409	1.557049
8	1	0	1.919541	0.868756	1.582365
9	6	0	2.897310	0.050713	3.983740
10	1	0	0.903888	0.784135	3.875558
11	1	0	1.028712	-0.968237	3.863736
12	6	0	3.502444	-0.841015	4.709755
13	1	0	3.452433	0.944336	3.696718
14	6	0	4.097808	-1.733807	5.440483
15	1	0	4.062935	-1.696312	6.525606
16	1	0	4.654848	-2.554291	4.997213
17	16	0	-1.391706	0.005865	-0.694429
18	6	0	-2.229667	-1.539354	-0.381519
19	8	0	-2.283027	1.025803	-0.190385
20	8	0	-1.143754	0.034175	-2.136282
21	6	0	-3.314561	-1.592314	0.473872
22	6	0	-3.918985	-2.809647	0.751724
23	6	0	-3.455443	-3.986651	0.183852
24	6	0	-2.358331	-3.916349	-0.673936
25	6	0	-1.747896	-2.711226	-0.950881
26	1	0	-3.686408	-0.674066	0.912627
27	1	0	-4.772062	-2.843576	1.422386
28	6	0	-4.104750	-5.301085	0.468448
29	1	0	-1.983705	-4.829237	-1.128344
30	1	0	-0.894619	-2.670867	-1.619719
31	1	0	-4.514780	-5.744815	-0.443104
32	1	0	-3.386673	-6.018698	0.874378
33	1	0	-4.920153	-5.199577	1.185996
34	19	0	1.436742	0.195969	-2.196508

NCS (Table S-1b)

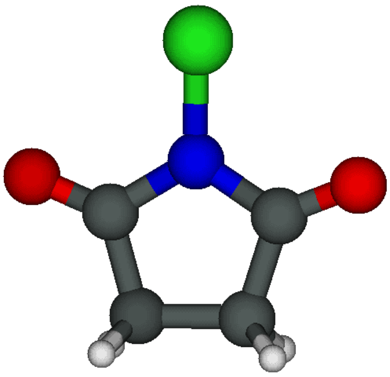

1	6	0	0.001667	0.000008	0.001497
2	6	0	0.001755	0.000027	1.522887
3	6	0	1.445787	0.000068	-0.421518
4	1	0	-0.481486	-0.876412	-0.433597
5	1	0	-0.481479	0.876452	-0.433567
6	6	0	1.445927	0.000464	1.945737
7	1	0	-0.481650	0.876279	1.958039

8	1	0	-0.481037	-0.876583	1.958007
9	8	0	1.906584	-0.000134	-1.522401
10	7	0	2.192534	0.000478	0.762064
11	8	0	1.906855	0.000724	3.046564
12	17	0	3.868903	0.000717	0.761964

TS$_{Cl-Exc}$ (Table S-1b) Imaginary frequency: 173 cm^{-1}.

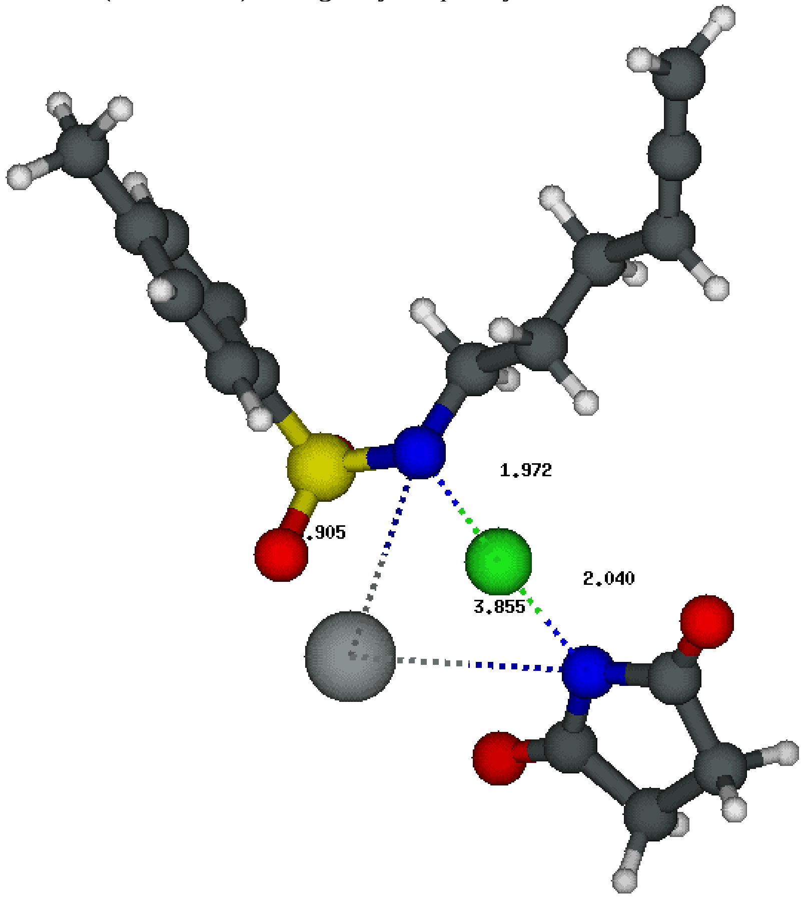

1	7	0	-0.081678	0.027397	-0.004905
2	6	0	-0.059048	0.024995	1.456249
3	6	0	1.365839	-0.004553	1.960024
4	1	0	-0.591058	0.902815	1.851211
5	1	0	-0.593382	-0.858381	1.831129
6	6	0	1.426039	0.042584	3.477098
7	1	0	1.856574	-0.911867	1.590829
8	1	0	1.920454	0.842278	1.541357
9	6	0	2.834493	0.048230	3.984655
10	1	0	0.916473	0.950610	3.824057
11	1	0	0.882486	-0.805189	3.906556
12	6	0	3.337542	-0.827677	4.801581
13	1	0	3.479151	0.847354	3.618361
14	6	0	3.832976	-1.703658	5.621850
15	1	0	3.798546	-1.558978	6.697973
16	1	0	4.306039	-2.614166	5.265235
17	16	0	-1.455580	-0.462833	-0.679864
18	6	0	-1.561331	-2.165349	-0.237796
19	8	0	-2.629367	0.213203	-0.156456
20	8	0	-1.302506	-0.360362	-2.113375
21	6	0	-2.617830	-2.628150	0.523970

22	6	0	-2.680586	-3.973695	0.847044
23	6	0	-1.699307	-4.858790	0.422364
24	6	0	-0.641001	-4.365027	-0.340391
25	6	0	-0.563625	-3.029721	-0.671167
26	1	0	-3.381642	-1.936892	0.858892
27	1	0	-3.508241	-4.343213	1.443471
28	6	0	-1.758201	-6.308911	0.768301
29	1	0	0.133101	-5.048031	-0.676057
30	1	0	0.262977	-2.649012	-1.260527
31	1	0	-0.876292	-6.612079	1.338698
32	1	0	-2.641783	-6.546921	1.361367
33	1	0	-1.778994	-6.926809	-0.133201
34	17	0	0.343154	1.846821	-0.635973
35	7	0	0.834567	3.743104	-1.204316
36	6	0	2.003074	4.358535	-0.813864
37	8	0	2.845505	3.877257	-0.099195
38	6	0	2.050908	5.745056	-1.430912
39	6	0	0.757619	5.857594	-2.213705
40	1	0	2.150264	6.482087	-0.632370
41	1	0	2.948043	5.822550	-2.047364
42	6	0	0.070484	4.528875	-1.990049
43	1	0	0.098607	6.657354	-1.870240
44	1	0	0.898756	6.003851	-3.286350
45	8	0	-1.019991	4.228603	-2.458841
46	19	0	-2.501174	2.116624	-2.247007

3a (Table S-1b)

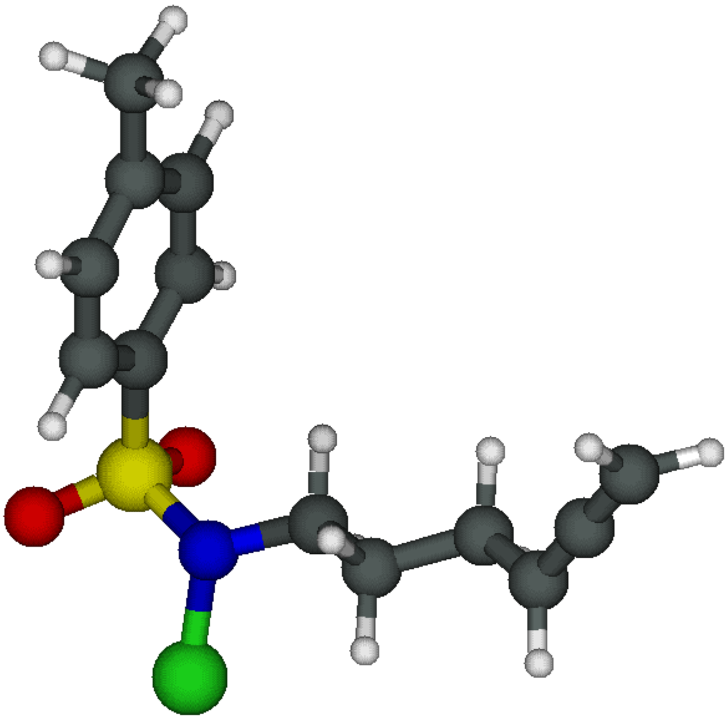

1	7	0	-0.042645	0.009383	-0.002868
2	6	0	-0.023043	0.010057	1.465541
3	6	0	1.388597	-0.014399	2.000237
4	1	0	-0.591031	0.863158	1.858118
5	1	0	-0.546941	-0.899202	1.778892
6	6	0	1.398301	-0.079778	3.519046

7	1	0	1.916700	-0.878100	1.582996
8	1	0	1.930349	0.877257	1.667969
9	6	0	2.789537	-0.063509	4.071712
10	1	0	0.841283	0.777644	3.917039
11	1	0	0.876377	-0.978604	3.861509
12	6	0	3.309280	-1.001012	4.805951
13	1	0	3.405644	0.799203	3.818222
14	6	0	3.820593	-1.938504	5.543218
15	1	0	3.746229	-1.914299	6.626604
16	1	0	4.346664	-2.782540	5.106637
17	16	0	-1.530463	-0.476513	-0.688978
18	17	0	0.405931	1.558660	-0.628758
19	6	0	-1.460171	-2.190664	-0.309529
20	8	0	-2.624544	0.119455	0.014119
21	8	0	-1.393396	-0.287849	-2.095170
22	6	0	-2.385527	-2.741557	0.558969
23	6	0	-2.344487	-4.101640	0.810968
24	6	0	-1.389226	-4.912515	0.211968
25	6	0	-0.465636	-4.329586	-0.655901
26	6	0	-0.494458	-2.978732	-0.923234
27	1	0	-3.130889	-2.108476	1.025225
28	1	0	-3.069655	-4.543204	1.486082
29	6	0	-1.340340	-6.379203	0.476830
30	1	0	0.283774	-4.955660	-1.129572
31	1	0	0.220315	-2.529898	-1.603225
32	1	0	-2.108343	-6.688092	1.186356
33	1	0	-1.484362	-6.946713	-0.446284
34	1	0	-0.367420	-6.672520	0.879327

K^+S^- (Table S-1b)

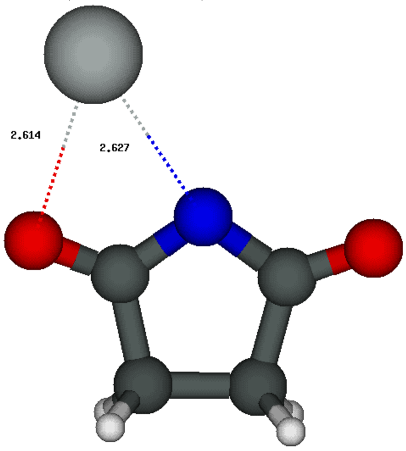

1	6	0	-0.001117	-0.001046	0.001794
2	6	0	0.001776	0.000525	1.514616
3	6	0	1.479848	0.001364	-0.365625
4	1	0	-0.477590	-0.876948	-0.442947
5	1	0	-0.477808	0.875002	-0.442772
6	6	0	1.478916	0.013997	1.854187
7	1	0	-0.479481	0.871577	1.964894
8	1	0	-0.461687	-0.880247	1.964881
9	8	0	1.885908	-0.005160	-1.510076
10	7	0	2.259610	0.012403	0.757545
11	8	0	1.910174	0.025487	3.013721
12	19	0	4.413527	0.055315	2.261291

TS$_{Cl\text{-}Exc}$ (Table S-1c) Imaginary frequency: 374 cm^{-1}.

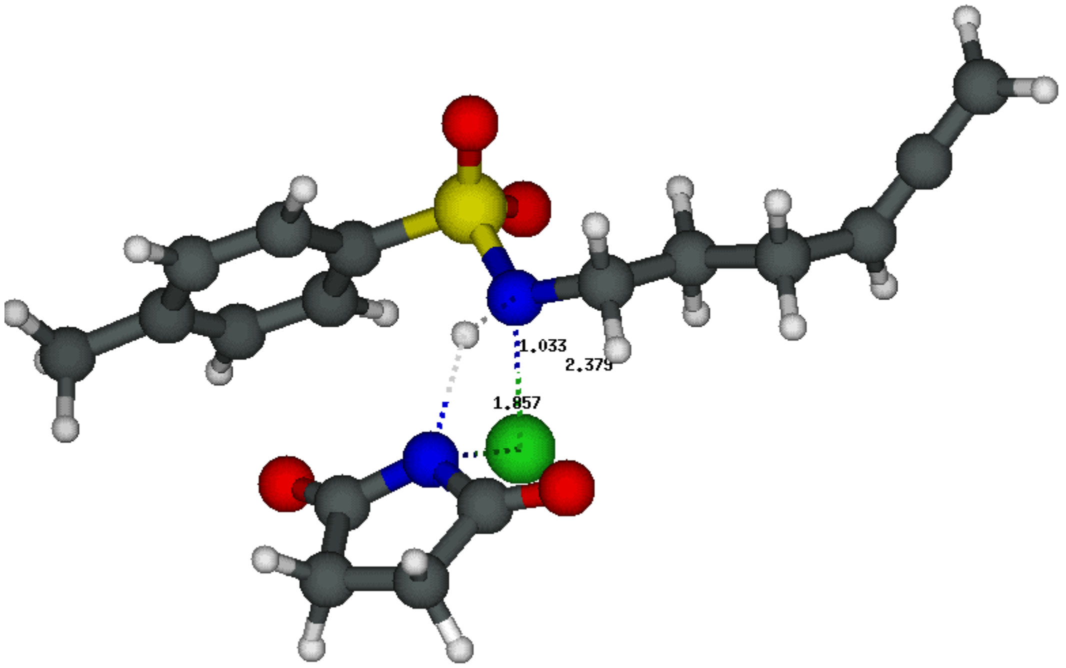

1	7	0	0.021296	0.010535	-0.019039
2	6	0	0.025852	-0.005661	1.363615
3	6	0	1.465404	-0.009485	1.847090
4	6	0	2.296415	-0.120549	0.583101
5	6	0	1.280333	-0.175475	-0.543513
6	8	0	-0.948100	-0.001892	2.071573
7	1	0	1.647123	0.903960	2.415850
8	1	0	1.592470	-0.844501	2.538426
9	1	0	2.963079	0.726417	0.413154
10	1	0	2.909207	-1.022400	0.530201
11	8	0	1.548156	-0.338280	-1.709829
12	1	0	-1.286157	0.746815	-1.113257
13	7	0	-1.909308	0.472670	-1.890405
14	16	0	-3.521157	1.029238	-1.723234
15	6	0	-3.630567	1.187535	0.007322
16	8	0	-3.541759	2.315589	-2.348388
17	8	0	-4.367617	-0.013767	-2.194226
18	6	0	-1.271842	0.498658	-3.193246
19	6	0	-1.996330	-0.295233	-4.258235
20	1	0	-1.168625	1.549174	-3.504595
21	1	0	-0.255752	0.118365	-3.022135
22	17	0	-1.563574	-1.655651	-0.885527
23	6	0	-3.155730	2.343769	0.612194
24	6	0	-3.232403	2.456523	1.983664
25	6	0	-3.774527	1.434035	2.757512
26	6	0	-4.259986	0.296008	2.122365
27	6	0	-4.185326	0.158161	0.752736
28	1	0	-2.743810	3.145426	0.010184
29	1	0	-2.864381	3.353514	2.470122
30	6	0	-3.792891	1.536459	4.243403
31	1	0	-4.690736	-0.501626	2.717778
32	1	0	-4.552153	-0.732907	0.259120
33	1	0	-4.637579	0.997087	4.674799

34	1	0	-2.880886	1.095452	4.657996
35	1	0	-3.833821	2.574294	4.577503
36	6	0	-1.170482	-0.338761	-5.534277
37	1	0	-2.178263	-1.310028	-3.890585
38	1	0	-2.975639	0.141869	-4.472130
39	6	0	-1.841768	-1.146837	-6.600847
40	1	0	-0.988852	0.676755	-5.899496
41	1	0	-0.189407	-0.775072	-5.310435
42	6	0	-2.211012	-0.688334	-7.759062
43	1	0	-2.044713	-2.191721	-6.366509
44	6	0	-2.573606	-0.225463	-8.915787
45	1	0	-3.556383	0.210366	-9.070632
46	1	0	-1.910013	-0.257813	-9.775113

SH (Table S-1c)

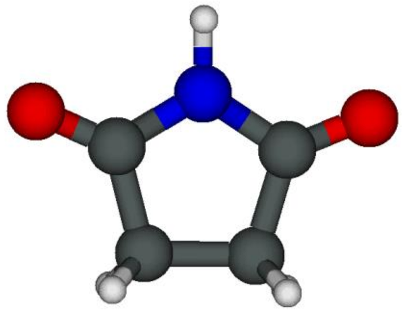

1	6	0	0.001099	0.000365	0.001378
2	6	0	0.001083	0.000399	1.521857
3	6	0	1.455587	0.000165	-0.403126
4	1	0	-0.480242	-0.875219	-0.436828
5	1	0	-0.479958	0.876034	-0.436944
6	6	0	1.455564	0.000313	1.926395
7	1	0	-0.480049	0.876043	1.960144
8	1	0	-0.480197	-0.875210	1.960085
9	8	0	1.910120	-0.000727	-1.513130
10	7	0	2.200837	-0.000358	0.761643
11	8	0	1.910068	-0.000496	3.036411
12	1	0	3.211651	-0.001047	0.761642

1a• (Table S-2a)

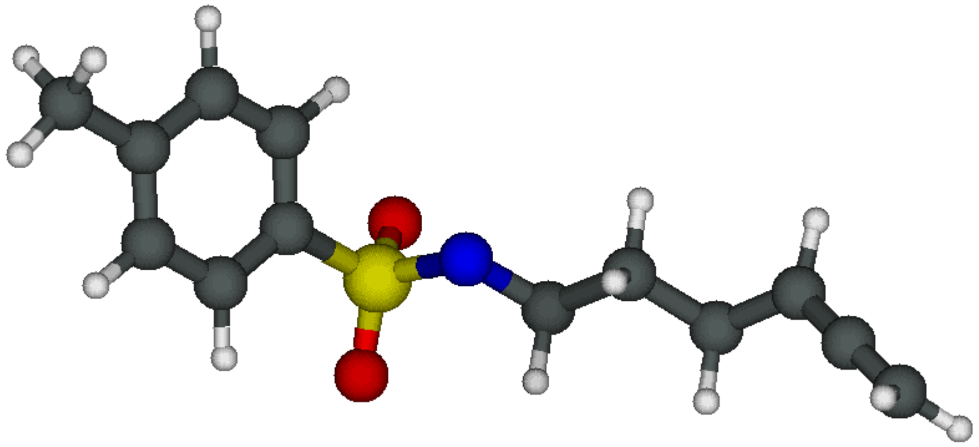

1	7	0	-0.024462	0.083148	-0.005101
2	6	0	-0.025060	0.086567	1.426113
3	6	0	1.380230	0.021096	1.977585
4	1	0	-0.536992	1.001976	1.769375
5	1	0	-0.644540	-0.739128	1.814454
6	6	0	1.388675	0.058387	3.496009
7	1	0	1.862812	-0.895466	1.622363
8	1	0	1.965112	0.855159	1.577463
9	6	0	2.772595	-0.064515	4.053647
10	1	0	0.927646	0.984586	3.852844
11	1	0	0.771223	-0.765090	3.877283
12	16	0	-1.521216	-0.107775	-0.687259
13	6	0	-1.185070	0.273802	-2.365545
14	8	0	-1.822984	-1.505880	-0.566602
15	8	0	-2.432505	0.863936	-0.159299
16	6	0	3.350796	0.816786	4.813353
17	1	0	3.330801	-0.960216	3.780813
18	6	0	3.920910	1.698575	5.575721
19	1	0	4.504414	2.516139	5.162376
20	1	0	3.839259	1.652517	6.657868
21	6	0	-0.429870	-0.611261	-3.124029
22	6	0	-0.173123	-0.313127	-4.443897
23	6	0	-0.662104	0.856087	-5.026672
24	6	0	-1.415798	1.722577	-4.246831
25	6	0	-1.679364	1.440996	-2.917546
26	1	0	-0.051390	-1.523473	-2.677392
27	1	0	0.416506	-0.997667	-5.045436
28	6	0	-0.376947	1.150736	-6.460825
29	1	0	-1.804242	2.634488	-4.687667
30	1	0	-2.269000	2.117031	-2.310921
31	1	0	0.698773	1.157370	-6.653971
32	1	0	-0.810030	0.385241	-7.110259
33	1	0	-0.781912	2.116865	-6.763353

^{1}**3a*** (Table S-2b)

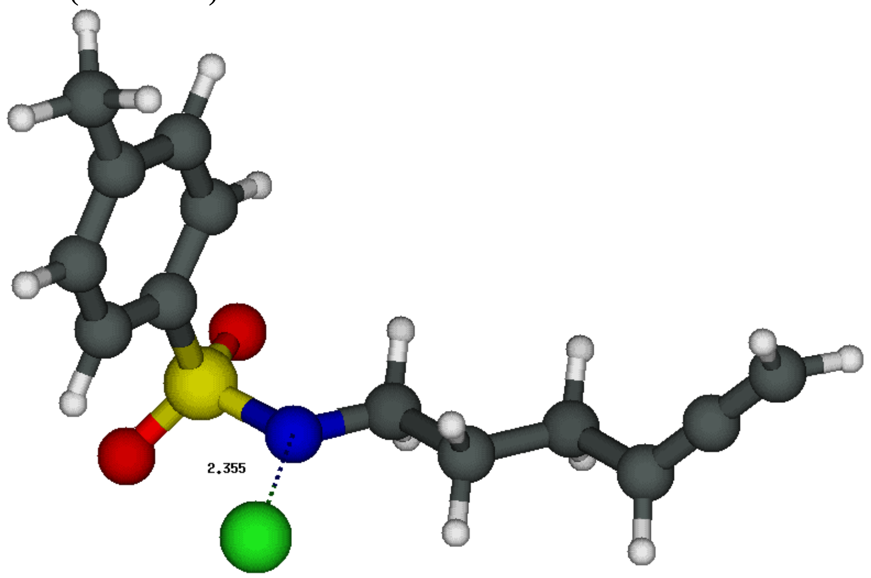

1	7	0	-0.020562	0.025347	-0.007424
2	6	0	-0.012151	0.018175	1.417290

3	6	0	1.370582	-0.004786	2.017170
4	1	0	-0.574034	0.906090	1.765581
5	1	0	-0.620352	-0.823531	1.789006
6	6	0	1.304851	0.023904	3.535325
7	1	0	1.903725	-0.898173	1.678349
8	1	0	1.942866	0.850910	1.647845
9	6	0	2.668182	0.033822	4.153530
10	1	0	0.758417	0.921370	3.852742
11	1	0	0.737204	-0.834322	3.908682
12	6	0	3.106592	-0.845479	5.003363
13	1	0	3.337508	0.836842	3.845244
14	6	0	3.542880	-1.725073	5.851663
15	1	0	3.423520	-1.590142	6.922776
16	1	0	4.039882	-2.633667	5.524422
17	16	0	-1.415789	-0.133879	-0.856027
18	17	0	1.996113	0.130919	-1.219709
19	6	0	-1.169567	-1.793573	-1.333995
20	8	0	-2.529296	-0.057082	0.041716
21	8	0	-1.349729	0.712238	-1.998742
22	6	0	-1.669160	-2.823512	-0.533749
23	6	0	-1.465491	-4.126061	-0.916428
24	6	0	-0.752092	-4.430613	-2.081017
25	6	0	-0.254057	-3.384565	-2.860900
26	6	0	-0.444385	-2.074547	-2.493121
27	1	0	-2.232605	-2.584977	0.360174
28	1	0	-1.865424	-4.935568	-0.315600
29	6	0	-0.506952	-5.845405	-2.460591
30	1	0	0.290491	-3.615127	-3.769619
31	1	0	-0.054979	-1.261034	-3.091814
32	1	0	-0.289450	-5.947254	-3.524105
33	1	0	0.355554	-6.238662	-1.912235
34	1	0	-1.358291	-6.480361	-2.209057

---

^{3}**3a*** (Table S-2c)

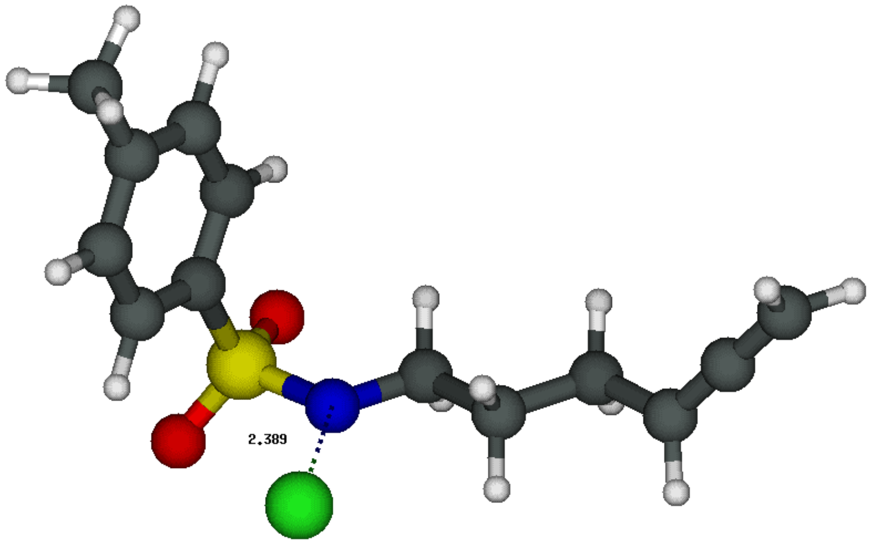

1	7	0	-0.115724	0.336617	-0.123170
2	6	0	-0.111902	0.108808	1.279432
3	6	0	1.274139	0.056046	1.879031
4	1	0	-0.718246	0.899713	1.754276
5	1	0	-0.669761	-0.820700	1.488401

6	6	0	1.205968	-0.166925	3.380615
7	1	0	1.848615	-0.743657	1.403224
8	1	0	1.800697	0.989052	1.657108
9	6	0	2.567109	-0.184159	4.003517
10	1	0	0.609780	0.634497	3.835520
11	1	0	0.687757	-1.105485	3.601071
12	6	0	3.065636	-1.179934	4.672832
13	1	0	3.179470	0.706410	3.862845
14	6	0	3.556946	-2.176073	5.343455
15	1	0	3.428538	-2.256001	6.419057
16	1	0	4.120061	-2.966850	4.856362
17	16	0	-1.582454	0.306318	-0.968980
18	17	0	1.892808	0.617615	-1.386093
19	6	0	-1.418510	-1.269996	-1.697849
20	8	0	-2.651732	0.295848	-0.019263
21	8	0	-1.490573	1.330405	-1.951959
22	6	0	-1.975289	-2.375022	-1.068012
23	6	0	-1.828697	-3.619584	-1.646635
24	6	0	-1.130348	-3.776855	-2.839880
25	6	0	-0.577249	-2.650242	-3.449007
26	6	0	-0.710405	-1.400224	-2.888702
27	1	0	-2.530878	-2.251832	-0.146003
28	1	0	-2.266971	-4.488720	-1.168368
29	6	0	-0.974078	-5.116767	-3.472147
30	1	0	-0.032512	-2.765802	-4.380253
31	1	0	-0.276994	-0.525962	-3.358548
32	1	0	-1.471889	-5.145777	-4.445230
33	1	0	0.079811	-5.343773	-3.651071
34	1	0	-1.395191	-5.909455	-2.853507

TS$_{Cl\text{-}Det}$ (Table S-2c) Imaginary frequency: 169 cm^{-1}.

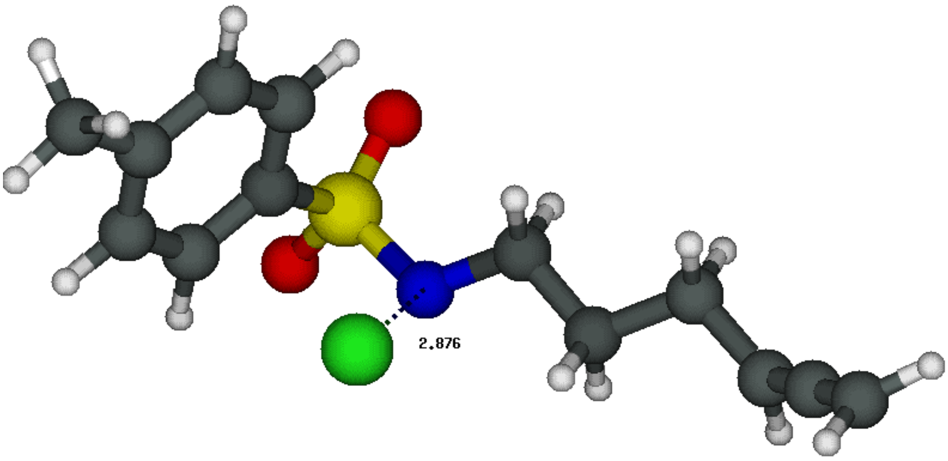

1	7	0	-0.019533	0.026390	-0.029552
2	6	0	-0.025128	-0.033164	1.394528
3	6	0	1.378728	-0.009008	1.956110
4	1	0	-0.640508	0.783119	1.804280
5	1	0	-0.548785	-0.956437	1.706718
6	6	0	1.370412	-0.081864	3.473487
7	1	0	1.950093	-0.843409	1.539247
8	1	0	1.877786	0.907980	1.625907
9	6	0	2.750854	0.006219	4.045797
10	1	0	0.761888	0.741271	3.869638

11	1	0	0.893352	-1.010233	3.802709
12	6	0	3.310075	-0.904724	4.784432
13	1	0	3.324068	0.900731	3.802030
14	6	0	3.861587	-1.816783	5.524527
15	1	0	3.772698	-1.801682	6.606985
16	1	0	4.436601	-2.629763	5.090735
17	16	0	-1.483052	0.271969	-0.788484
18	17	0	0.972480	-2.600799	-0.651372
19	6	0	-1.782092	-1.366044	-1.350462
20	8	0	-2.504125	0.637694	0.148060
21	8	0	-1.211005	1.109544	-1.912313
22	6	0	-2.600570	-2.215539	-0.612439
23	6	0	-2.872515	-3.468125	-1.106547
24	6	0	-2.348598	-3.896967	-2.335702
25	6	0	-1.531850	-3.035616	-3.053772
26	6	0	-1.228347	-1.780037	-2.565938
27	1	0	-3.028956	-1.876293	0.322879
28	1	0	-3.515376	-4.140445	-0.547760
29	6	0	-2.674521	-5.258101	-2.838097
30	1	0	-1.117389	-3.357532	-4.002271
31	1	0	-0.593799	-1.100586	-3.122217
32	1	0	-2.217642	-5.453574	-3.807833
33	1	0	-2.326856	-6.020828	-2.135855
34	1	0	-3.755871	-5.389116	-2.933024

Cpl [Cl• 1a•] (Table S-2c)

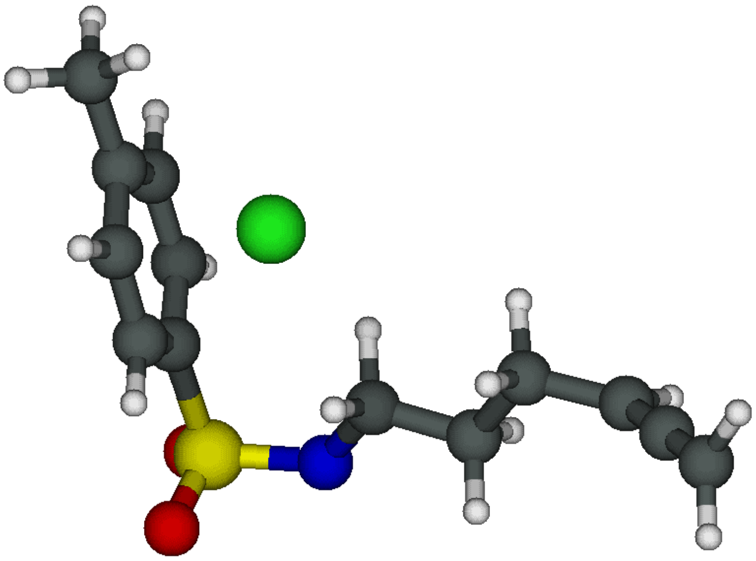

1	7	0	-0.074068	0.407971	0.165901
2	6	0	-0.038016	-0.017604	1.526127
3	6	0	1.333346	0.164688	2.132162
4	1	0	-0.794799	0.542159	2.105945
5	1	0	-0.371196	-1.067935	1.609557
6	6	0	1.359814	-0.279962	3.584641
7	1	0	2.064431	-0.404587	1.550240
8	1	0	1.624619	1.217373	2.052226
9	6	0	2.696888	-0.050192	4.217238
10	1	0	0.593528	0.274512	4.142312
11	1	0	1.090448	-1.338063	3.659744
12	6	0	3.442221	-0.976556	4.741268

13	1	0	3.067396	0.975032	4.217190
14	6	0	4.179744	-1.905757	5.266739
15	1	0	4.098711	-2.169837	6.317221
16	1	0	4.908163	-2.455251	4.677526
17	16	0	-1.383437	0.065976	-0.767372
18	17	0	-2.652874	-1.937485	3.557906
19	6	0	-2.756276	-0.223799	0.304977
20	8	0	-1.657563	1.248472	-1.518924
21	8	0	-1.062724	-1.164527	-1.425383
22	6	0	-3.478345	0.862157	0.799139
23	6	0	-4.543794	0.656364	1.659234
24	6	0	-4.905353	-0.620476	2.051429
25	6	0	-4.147356	-1.711354	1.565432
26	6	0	-3.086053	-1.507454	0.669513
27	1	0	-3.207291	1.866607	0.493410
28	1	0	-5.107667	1.505875	2.027280
29	6	0	-6.037740	-0.874987	2.974504
30	1	0	-4.492504	-2.719634	1.761675
31	1	0	-2.532587	-2.353146	0.279523
32	1	0	-6.745001	-1.585849	2.539146
33	1	0	-5.670501	-1.323534	3.902655
34	1	0	-6.572879	0.041093	3.222254

Cpl [Cl• Toluene] (Table S-2c)

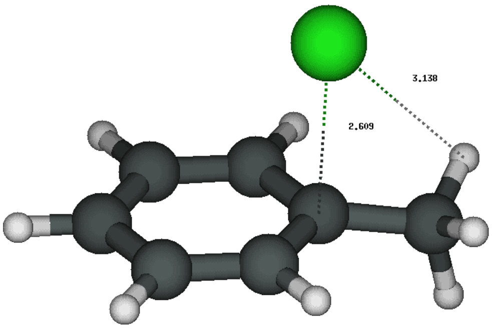

1	6	0	0.002373	0.001643	0.009380
2	6	0	-0.000012	0.004717	1.495526
3	1	0	1.017691	-0.006334	-0.388201
4	1	0	-0.538729	-0.856335	-0.390364
5	1	0	-0.491451	0.907802	-0.358567
6	6	0	-1.107991	-0.495534	2.215052
7	6	0	-1.215080	-0.299253	3.571554
8	6	0	-0.222877	0.396301	4.251334
9	6	0	0.884341	0.891742	3.568404
10	6	0	0.997169	0.700034	2.213572
11	1	0	-1.874416	-1.037345	1.671328
12	1	0	-2.068660	-0.691947	4.110402
13	1	0	-0.307162	0.547804	5.321225
14	1	0	1.658818	1.424052	4.107097
15	1	0	1.854864	1.080661	1.669473
16	17	0	1.224114	-2.284221	1.759501

TS$_A$ (Table S-3) Imaginary frequency: 416 cm^{-1}.

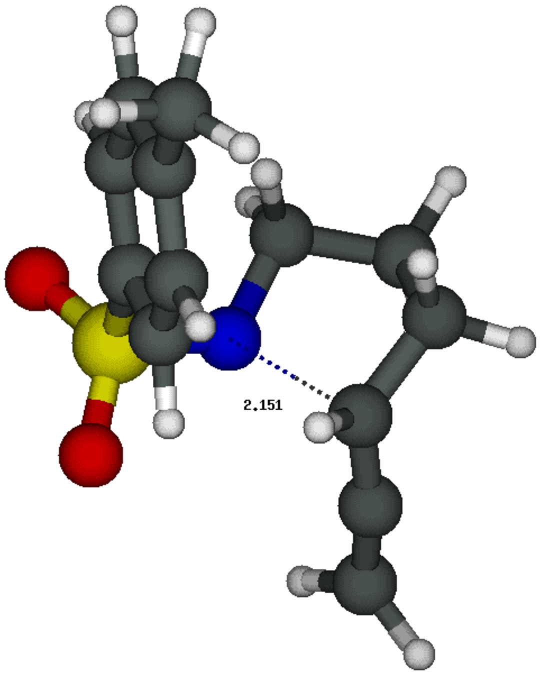

1	7	0	-0.003136	-0.028234	0.013634
2	6	0	0.055020	-0.012460	1.455135
3	6	0	1.521023	-0.060673	1.831904
4	1	0	-0.416804	0.907504	1.819325
5	1	0	-0.464238	-0.864310	1.918522
6	6	0	2.111022	-1.254324	1.100916
7	1	0	2.016318	0.858617	1.503632
8	1	0	1.648982	-0.143806	2.912705
9	6	0	1.800644	-1.137372	-0.365717
10	1	0	3.190248	-1.320241	1.257608
11	1	0	1.667651	-2.178937	1.483409
12	6	0	2.398528	-0.240355	-1.161509
13	1	0	1.348772	-1.994941	-0.858012
14	6	0	2.769147	0.750986	-1.897647
15	1	0	3.731592	0.787952	-2.400750
16	1	0	2.095050	1.595292	-2.046178
17	16	0	-1.293717	-0.650608	-0.744779
18	6	0	-1.519809	-2.314574	-0.179365
19	8	0	-2.435875	0.101906	-0.302000
20	8	0	-0.972970	-0.711115	-2.138593
21	6	0	-2.259665	-2.567135	0.966928
22	6	0	-2.392795	-3.865763	1.424126
23	6	0	-1.804469	-4.929611	0.750136
24	6	0	-1.079455	-4.658245	-0.406653
25	6	0	-0.936786	-3.366080	-0.873517
26	1	0	-2.746222	-1.748894	1.484447
27	1	0	-2.973129	-4.060890	2.320281
28	6	0	-1.980131	-6.332930	1.226545
29	1	0	-0.628462	-5.479339	-0.954986
30	1	0	-0.395698	-3.168595	-1.791631
31	1	0	-1.093187	-6.937304	1.027231
32	1	0	-2.189905	-6.371053	2.296638
33	1	0	-2.818125	-6.814473	0.713730

R$_1$ (Table S-3)

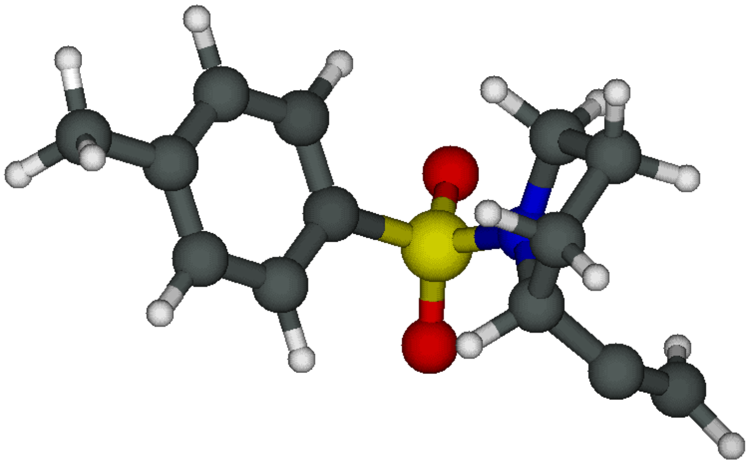

1	7	0	-0.019686	-0.024766	-0.013281
2	6	0	-0.009177	-0.021852	1.456673
3	6	0	1.469750	-0.022994	1.790758
4	1	0	-0.549319	0.842752	1.842554
5	1	0	-0.488410	-0.929499	1.847949
6	6	0	2.061380	-0.868561	0.678292
7	1	0	1.868210	0.994370	1.743183
8	1	0	1.665761	-0.417574	2.787497
9	6	0	1.318451	-0.366425	-0.559573
10	1	0	3.142394	-0.774596	0.574702
11	1	0	1.817057	-1.924309	0.836040
12	6	0	1.945109	0.815840	-1.163918
13	1	0	1.227616	-1.154737	-1.313412
14	6	0	1.681126	2.067519	-1.395292
15	1	0	2.390674	2.735082	-1.877052
16	1	0	0.714683	2.500944	-1.125346
17	16	0	-1.370957	-0.470408	-0.815360
18	6	0	-1.486728	-2.220429	-0.602356
19	8	0	-2.468993	0.127899	-0.121997
20	8	0	-1.136517	-0.213837	-2.201592
21	6	0	-2.146663	-2.740988	0.500654
22	6	0	-2.181997	-4.111176	0.691167
23	6	0	-1.569545	-4.977271	-0.205606
24	6	0	-0.921750	-4.434598	-1.312821
25	6	0	-0.877360	-3.070598	-1.517603
26	1	0	-2.650988	-2.075998	1.191407
27	1	0	-2.705764	-4.518103	1.549906
28	6	0	-1.610429	-6.456077	-0.010743
29	1	0	-0.455285	-5.100661	-2.032150
30	1	0	-0.396053	-2.660147	-2.397291
31	1	0	-2.143820	-6.728863	0.900473
32	1	0	-2.105888	-6.948782	-0.851411
33	1	0	-0.601697	-6.872798	0.049948

TS$_B$ (Table S-3) Imaginary frequency: 471 cm^{-1}.

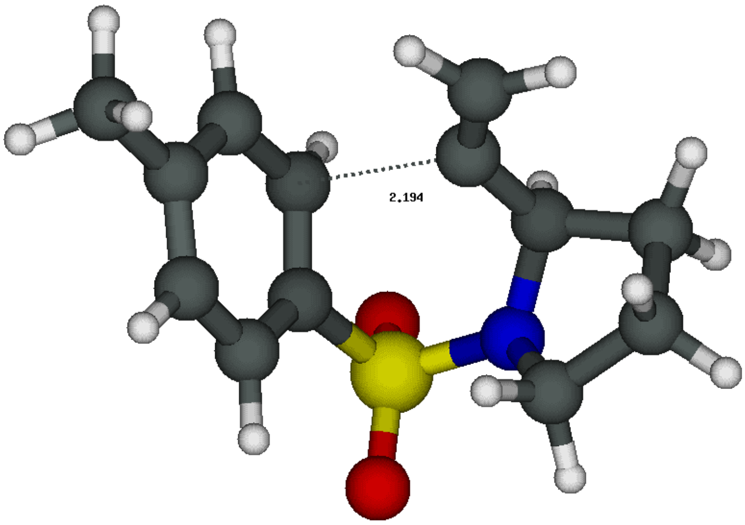

1	7	0	-0.009460	0.021414	0.000071
2	6	0	0.000800	0.067861	1.468041
3	6	0	1.489135	-0.016784	1.827881
4	1	0	-0.440637	1.002450	1.839109
5	1	0	-0.590115	-0.759310	1.858793
6	6	0	2.232459	-0.071544	0.493134
7	1	0	1.785063	0.862282	2.402027
8	1	0	1.705696	-0.890804	2.441807
9	6	0	1.264047	0.574483	-0.494416
10	1	0	3.201505	0.427312	0.515950
11	1	0	2.394115	-1.105040	0.180792
12	6	0	1.292819	2.059733	-0.454302
13	1	0	1.421631	0.229385	-1.520030
14	6	0	2.061209	2.969188	0.078947
15	1	0	1.859602	4.032289	-0.033586
16	1	0	2.950717	2.713185	0.658033
17	16	0	-1.392797	0.370952	-0.812883
18	6	0	-1.435868	2.119529	-0.744453
19	8	0	-1.181804	-0.019416	-2.172101
20	8	0	-2.473327	-0.170272	-0.050730
21	6	0	-0.474072	2.807065	-1.519842
22	6	0	-0.438638	4.209509	-1.423883
23	6	0	-1.198904	4.884490	-0.489045
24	6	0	-2.065899	4.156683	0.334039
25	6	0	-2.187069	2.782265	0.203865
26	1	0	-0.069528	2.325037	-2.403342
27	1	0	0.218709	4.767256	-2.083232
28	6	0	-1.129336	6.369804	-0.358863
29	1	0	-2.669704	4.684358	1.065350
30	1	0	-2.876488	2.223318	0.825954
31	1	0	-0.431066	6.805841	-1.074226
32	1	0	-2.109664	6.825210	-0.521374
33	1	0	-0.809632	6.659393	0.645855

4a’ (Table S-3)

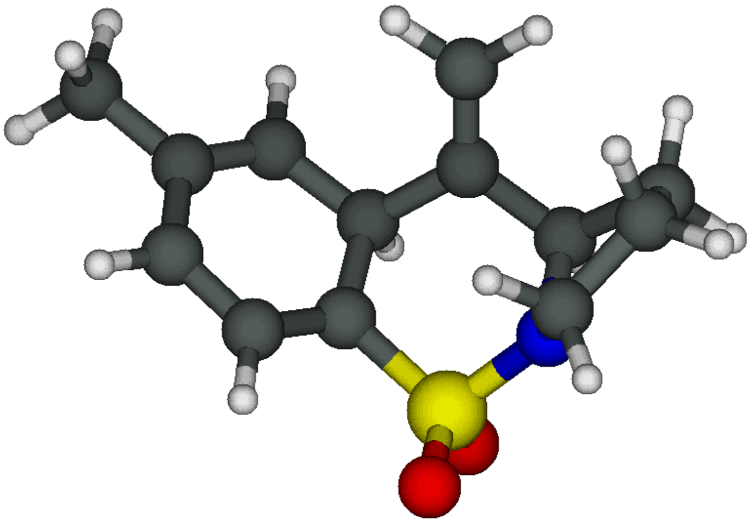

1	7	0	-0.009629	0.022359	0.001438
2	6	0	0.000295	0.064734	1.470195
3	6	0	1.489158	-0.024778	1.822831
4	1	0	-0.439587	0.998958	1.843935
5	1	0	-0.592208	-0.761748	1.859330
6	6	0	2.231918	0.039566	0.484413
7	1	0	1.770943	0.802388	2.475622
8	1	0	1.717991	-0.946932	2.356876
9	6	0	1.237996	0.661598	-0.479112
10	1	0	3.176009	0.581049	0.536024
11	1	0	2.454241	-0.967793	0.128498
12	6	0	1.119892	2.171603	-0.442816
13	1	0	1.408023	0.341863	-1.512077
14	6	0	1.899966	2.960118	0.279386
15	1	0	1.766199	4.035450	0.294845
16	1	0	2.710454	2.571705	0.884187
17	16	0	-1.387662	0.398189	-0.803048
18	6	0	-1.326187	2.140343	-0.788720
19	8	0	-1.208093	-0.030682	-2.156958
20	8	0	-2.494450	-0.075764	-0.033537
21	6	0	-0.033659	2.716251	-1.292493
22	6	0	-0.086535	4.202054	-1.363405
23	6	0	-1.121591	4.944119	-0.898155
24	6	0	-2.247567	4.299487	-0.322360
25	6	0	-2.333739	2.903521	-0.291248
26	1	0	0.141957	2.322424	-2.309554
27	1	0	0.769141	4.698667	-1.812218
28	6	0	-1.119818	6.434930	-0.982863
29	1	0	-3.062378	4.900363	0.067184
30	1	0	-3.215763	2.417283	0.111440
31	1	0	-0.206152	6.812438	-1.443337
32	1	0	-1.969786	6.794554	-1.569142
33	1	0	-1.210235	6.882042	0.010953

Cpl [1a• NCS] (Table S-3)

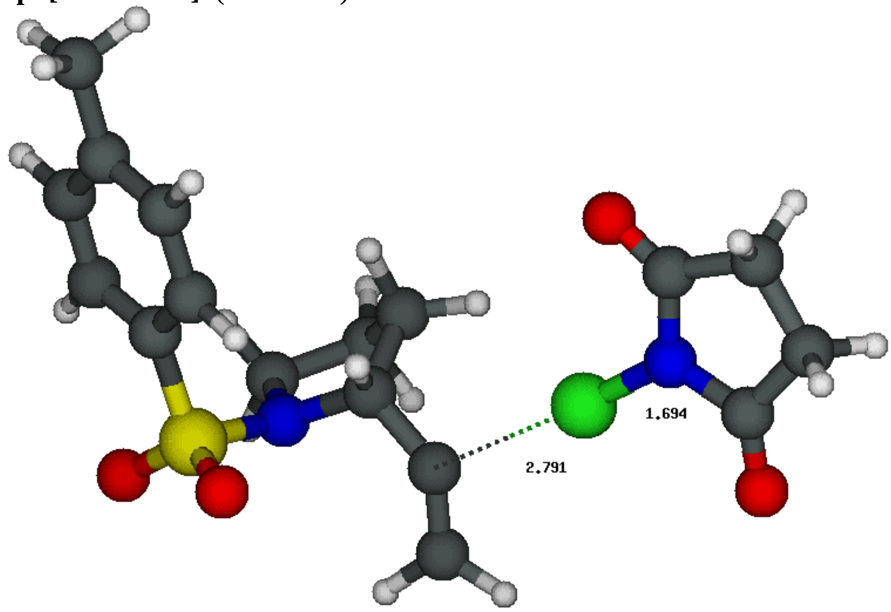

1	7	0	0.003601	-0.056992	-0.034622
2	6	0	0.026325	-0.041782	1.435637
3	6	0	1.507696	-0.069883	1.755718
4	1	0	-0.493432	0.836960	1.817172
5	1	0	-0.467755	-0.936343	1.838642
6	6	0	2.069249	-0.947381	0.652421
7	1	0	1.927622	0.937737	1.687124
8	1	0	1.705229	-0.452020	2.756951
9	6	0	1.321965	-0.452481	-0.585911
10	1	0	3.151023	-0.880960	0.534121
11	1	0	1.805008	-1.994923	0.829357
12	6	0	1.971842	0.698851	-1.224742
13	1	0	1.207190	-1.253949	-1.323440
14	6	0	1.784272	1.958108	-1.482224
15	1	0	2.524674	2.561231	-2.001375
16	1	0	0.856032	2.458675	-1.197519
17	16	0	-1.367666	-0.475470	-0.821541
18	6	0	-1.519370	-2.220153	-0.593751
19	8	0	-2.442526	0.154516	-0.120260
20	8	0	-1.140205	-0.231146	-2.210893
21	6	0	-2.190172	-2.716144	0.513936
22	6	0	-2.250814	-4.083235	0.719327
23	6	0	-1.651466	-4.969998	-0.166029
24	6	0	-0.992108	-4.451569	-1.277992
25	6	0	-0.923541	-3.091131	-1.498479
26	1	0	-2.682490	-2.034119	1.196679
27	1	0	-2.783261	-4.470995	1.581581
28	6	0	-1.715612	-6.445323	0.047679
29	1	0	-0.533782	-5.133433	-1.987564
30	1	0	-0.431816	-2.700520	-2.381377
31	1	0	-2.202404	-6.943226	-0.794917
32	1	0	-0.713381	-6.873596	0.131855
33	1	0	-2.267926	-6.698488	0.953285
34	17	0	4.283461	-0.310138	-2.418665
35	7	0	5.647421	-1.009286	-3.140023
36	6	0	6.563636	-0.266254	-3.889183
37	8	0	6.491422	0.907304	-4.097000
38	6	0	7.615265	-1.241545	-4.348420

39	6	0	5.932436	-2.370674	-3.025465
40	8	0	5.272542	-3.164044	-2.422865
41	6	0	7.203164	-2.599262	-3.800246
42	1	0	7.662671	-1.207278	-5.437768
43	1	0	8.582659	-0.898989	-3.978179
44	1	0	7.000551	-3.337979	-4.577243
45	1	0	7.940378	-3.040754	-3.128135

TS$_C$ (Table S-3) Imaginary frequency: 318 cm^{-1}.

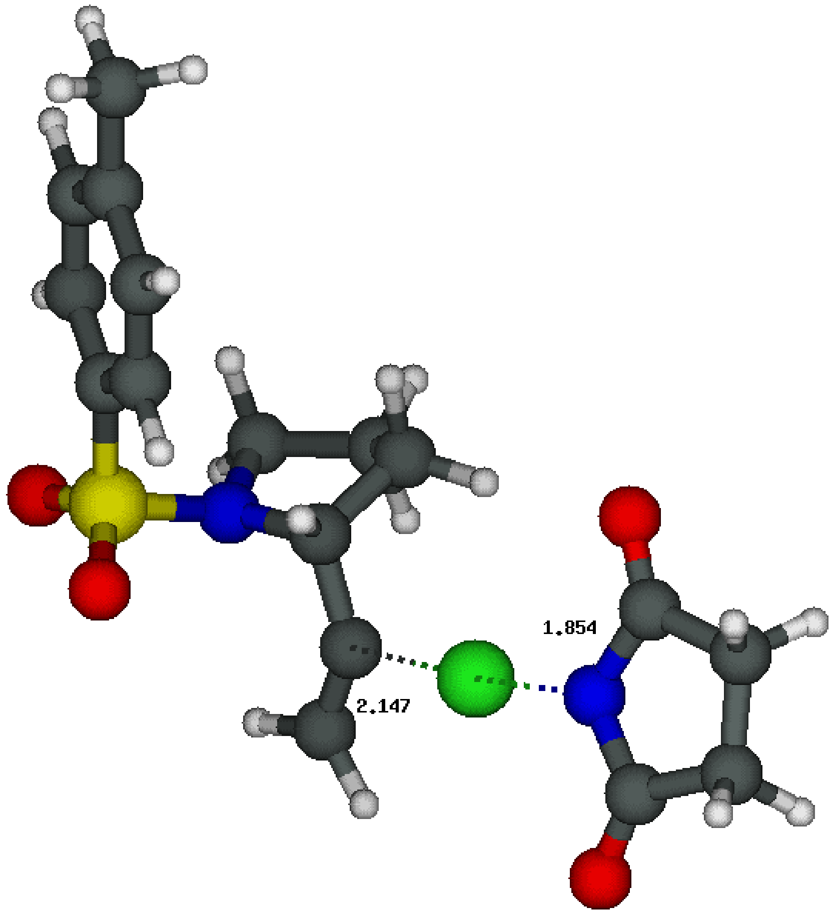

1	7	0	0.052195	-0.097799	-0.021904
2	6	0	0.159530	-0.142698	1.445013
3	6	0	1.656030	-0.210523	1.676478
4	1	0	-0.320914	0.728154	1.890720
5	1	0	-0.329925	-1.044744	1.836230
6	6	0	2.139671	-1.036557	0.498873
7	1	0	2.090220	0.792628	1.639841
8	1	0	1.903492	-0.649880	2.642319
9	6	0	1.320034	-0.479207	-0.663760
10	1	0	3.212482	-0.981852	0.318134
11	1	0	1.869883	-2.088296	0.636785
12	6	0	1.920679	0.726477	-1.281723
13	1	0	1.163032	-1.234220	-1.440200
14	6	0	1.688340	2.002535	-1.369717
15	1	0	2.352215	2.682690	-1.891853
16	1	0	0.783553	2.419528	-0.928652
17	16	0	-1.375682	-0.446285	-0.749533
18	6	0	-1.568284	-2.190664	-0.570419

19	8	0	-2.386588	0.192816	0.032496
20	8	0	-1.210152	-0.156340	-2.138369
21	6	0	-2.210316	-2.702030	0.547173
22	6	0	-2.305687	-4.072627	0.710448
23	6	0	-1.769147	-4.946579	-0.226563
24	6	0	-1.136982	-4.411538	-1.346602
25	6	0	-1.034676	-3.047425	-1.526239
26	1	0	-2.652576	-2.028163	1.270969
27	1	0	-2.815415	-4.473164	1.580472
28	6	0	-1.873256	-6.425447	-0.059595
29	1	0	-0.727681	-5.083106	-2.094845
30	1	0	-0.565457	-2.642801	-2.415100
31	1	0	-2.429096	-6.874807	-0.886680
32	1	0	-0.884178	-6.890651	-0.053994
33	1	0	-2.378110	-6.692438	0.869357
34	17	0	3.670537	0.083791	-2.346061
35	7	0	5.279786	-0.502047	-3.057224
36	6	0	6.027226	0.266919	-3.933455
37	8	0	5.741805	1.356206	-4.340157
38	6	0	7.259146	-0.547998	-4.262221
39	6	0	5.833939	-1.714689	-2.693681
40	8	0	5.364846	-2.505302	-1.923945
41	6	0	7.134352	-1.832436	-3.457235
42	1	0	7.283540	-0.708908	-5.341114
43	1	0	8.138983	0.045016	-4.008165
44	1	0	7.090066	-2.732268	-4.072712
45	1	0	7.941384	-1.978387	-2.737605

Cpl [2a S$^\bullet$] (Table S-3)

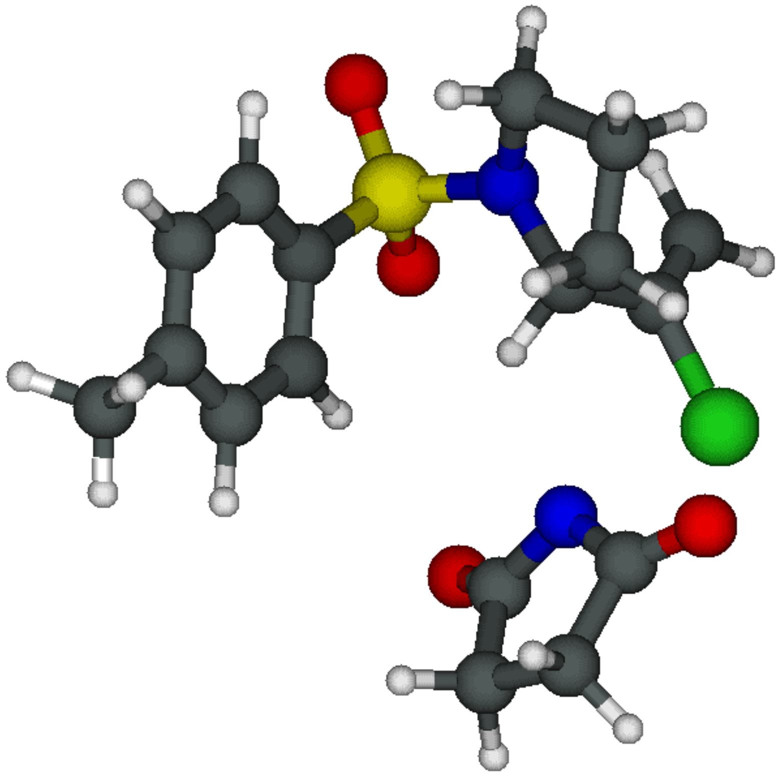

1	7	0	-0.032416	-0.012627	-0.023143
2	6	0	-0.059679	-0.000686	1.448915

3	6	0	1.410478	-0.002193	1.811386
4	1	0	-0.609417	0.867198	1.812742
5	1	0	-0.549606	-0.904172	1.835690
6	6	0	2.013522	-0.877068	0.728739
7	1	0	1.816656	1.011578	1.750092
8	1	0	1.587043	-0.376495	2.819481
9	6	0	1.283179	-0.410040	-0.531195
10	1	0	3.096934	-0.803775	0.638719
11	1	0	1.760468	-1.928480	0.905441
12	6	0	1.973194	0.725325	-1.231313
13	1	0	1.198365	-1.231401	-1.253588
14	6	0	1.582333	1.979693	-1.320003
15	1	0	2.155891	2.725638	-1.853454
16	1	0	0.647451	2.275959	-0.860046
17	16	0	-1.379533	-0.458012	-0.848344
18	6	0	-1.434314	-2.217113	-0.711722
19	8	0	-2.485099	0.082905	-0.120958
20	8	0	-1.158684	-0.130852	-2.220358
21	6	0	-2.054477	-2.810134	0.381717
22	6	0	-2.031993	-4.184137	0.511586
23	6	0	-1.399322	-4.987972	-0.433822
24	6	0	-0.795457	-4.374693	-1.524485
25	6	0	-0.810220	-2.998824	-1.672542
26	1	0	-2.571843	-2.195627	1.108729
27	1	0	-2.525009	-4.651393	1.358515
28	6	0	-1.392661	-6.471573	-0.273151
29	1	0	-0.318719	-4.986246	-2.284453
30	1	0	-0.353937	-2.536379	-2.539844
31	1	0	-2.410602	-6.864615	-0.211112
32	1	0	-0.891539	-6.965311	-1.106559
33	1	0	-0.883950	-6.765062	0.649043
34	17	0	3.474309	0.221902	-1.985036
35	7	0	3.199354	-2.633547	-1.717193
36	6	0	4.436114	-3.089425	-1.285747
37	8	0	5.161716	-2.583228	-0.482245
38	6	0	4.650157	-4.406782	-2.018198
39	6	0	2.761896	-3.181553	-2.910934
40	8	0	1.911347	-2.756185	-3.637306
41	6	0	3.548386	-4.473443	-3.069170
42	1	0	5.658338	-4.390938	-2.435544
43	1	0	4.613511	-5.218478	-1.290591
44	1	0	3.913490	-4.521145	-4.096131
45	1	0	2.862536	-5.310531	-2.929176

2a (Table S-3)

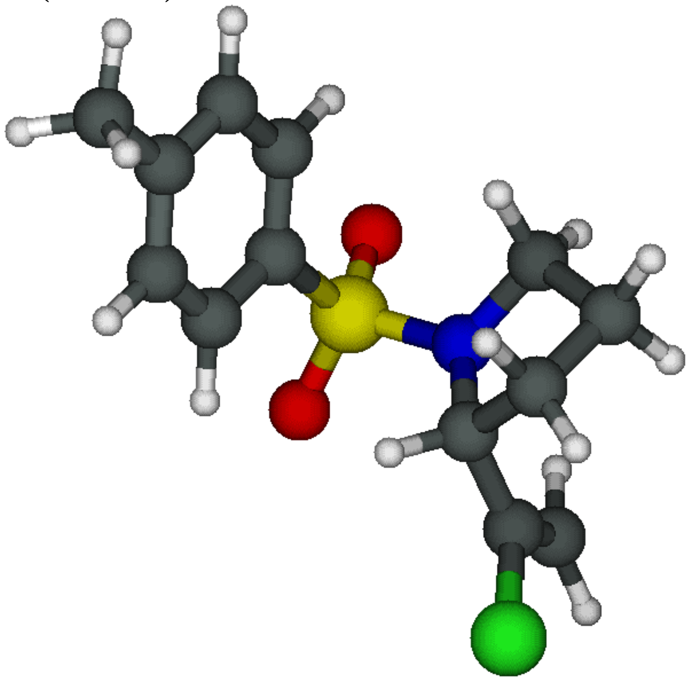

1	7	0	-0.026898	-0.016020	-0.009872
2	6	0	-0.016631	0.000829	1.461533
3	6	0	1.462386	-0.017485	1.790957
4	1	0	-0.544682	0.877273	1.837547
5	1	0	-0.509856	-0.894956	1.861875
6	6	0	2.038568	-0.876245	0.680322
7	1	0	1.874406	0.994219	1.740056
8	1	0	1.657591	-0.411282	2.788093
9	6	0	1.283342	-0.384050	-0.555624
10	1	0	3.119855	-0.798930	0.570513
11	1	0	1.786272	-1.928311	0.848765
12	6	0	1.948056	0.787559	-1.222576
13	1	0	1.187636	-1.182834	-1.298856
14	6	0	1.541971	2.040559	-1.238217
15	1	0	2.099545	2.816178	-1.745919
16	1	0	0.613663	2.307572	-0.748178
17	16	0	-1.393185	-0.448691	-0.801441
18	6	0	-1.529536	-2.194639	-0.576603
19	8	0	-2.473402	0.170933	-0.099769
20	8	0	-1.166086	-0.201511	-2.190119
21	6	0	-2.194909	-2.699472	0.530437
22	6	0	-2.250666	-4.067946	0.727345
23	6	0	-1.652995	-4.947318	-0.166550
24	6	0	-0.998778	-4.419904	-1.277363
25	6	0	-0.934389	-3.057875	-1.489137
26	1	0	-2.688066	-2.023684	1.218724
27	1	0	-2.779211	-4.462805	1.588735
28	6	0	-1.715652	-6.424260	0.035681
29	1	0	-0.543376	-5.096239	-1.994102
30	1	0	-0.448669	-2.659623	-2.371932
31	1	0	-2.260107	-6.684836	0.943893
32	1	0	-2.210375	-6.914694	-0.806684
33	1	0	-0.713051	-6.853922	0.107090
34	17	0	3.428508	0.328594	-2.038966

S$^{\bullet}$ (Table S-3)

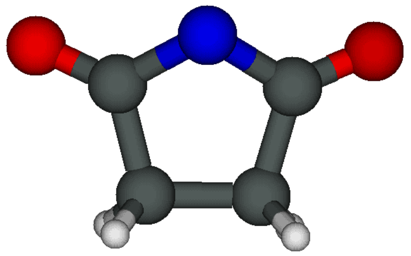

1	6	0	0.000445	-0.001282	0.001740
2	6	0	0.000632	0.002698	1.527933
3	6	0	1.467939	0.005775	-0.397602
4	1	0	-0.473026	-0.883664	-0.430889
5	1	0	-0.471812	0.874478	-0.445912
6	6	0	1.468139	0.009382	1.927054
7	1	0	-0.469815	0.881805	1.970927
8	1	0	-0.474503	-0.876343	1.965483
9	8	0	1.935528	0.219584	-1.474727
10	7	0	2.154083	-0.340745	0.765211
11	8	0	1.935964	0.225401	3.003653

Cpl [1a$^{\bullet}$ 3a] (Table S-3)

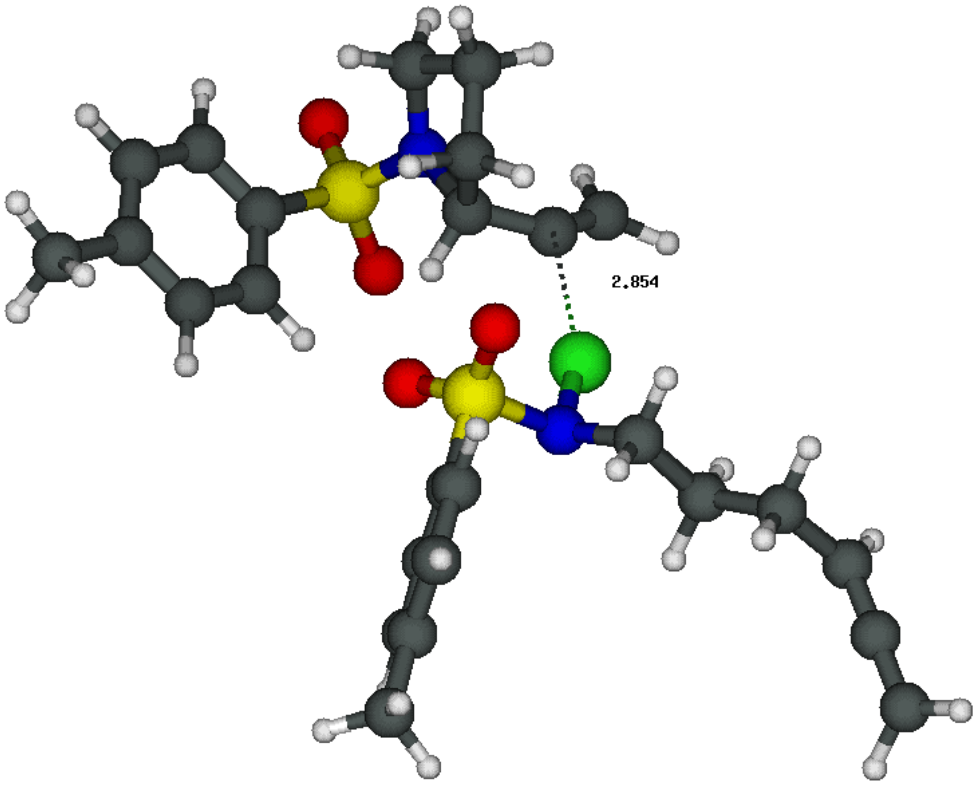

1	7	0	-0.006642	-0.037309	-0.011329
2	6	0	-0.060672	-0.026246	1.458339
3	6	0	1.402112	-0.007513	1.855724
4	1	0	-0.628641	0.833496	1.814024
5	1	0	-0.545016	-0.938080	1.833531
6	6	0	2.049448	-0.862613	0.782380
7	1	0	1.791559	1.013816	1.811231

8	1	0	1.558763	-0.386355	2.865504
9	6	0	1.351085	-0.390588	-0.493081
10	1	0	3.133112	-0.760633	0.717998
11	1	0	1.814838	-1.920023	0.942069
12	6	0	1.999743	0.778486	-1.100679
13	1	0	1.305630	-1.197585	-1.231815
14	6	0	1.785414	2.036495	-1.346487
15	1	0	2.520768	2.670486	-1.834256
16	1	0	0.834915	2.506498	-1.082557
17	16	0	-1.319659	-0.510686	-0.863180
18	6	0	-1.424941	-2.257058	-0.621844
19	8	0	-2.451553	0.088775	-0.226610
20	8	0	-1.030479	-0.271030	-2.242052
21	6	0	-2.168129	-2.763610	0.433513
22	6	0	-2.190328	-4.128634	0.659472
23	6	0	-1.478317	-5.002261	-0.152364
24	6	0	-0.745653	-4.473726	-1.212184
25	6	0	-0.716028	-3.115702	-1.454235
26	1	0	-2.742044	-2.091066	1.059558
27	1	0	-2.777751	-4.525250	1.481141
28	6	0	-1.500093	-6.475617	0.083300
29	1	0	-0.192153	-5.144806	-1.861373
30	1	0	-0.159830	-2.720940	-2.296161
31	1	0	-1.952304	-7.000954	-0.762258
32	1	0	-0.487211	-6.870349	0.196098
33	1	0	-2.066289	-6.733006	0.979227
34	17	0	4.140529	-0.753376	-2.203000
35	7	0	5.140020	-2.120468	-2.621867
36	6	0	6.565099	-1.784344	-2.519234
37	6	0	7.009426	-0.925798	-3.679168
38	1	0	6.780345	-1.306397	-1.554780
39	1	0	7.110915	-2.734105	-2.537748
40	6	0	8.496679	-0.621016	-3.602247
41	1	0	6.778968	-1.441949	-4.616951
42	1	0	6.442576	0.010992	-3.688398
43	6	0	8.945194	0.263232	-4.724034
44	1	0	8.711389	-0.128480	-2.645493
45	1	0	9.073718	-1.550830	-3.612554
46	6	0	9.847842	-0.051182	-5.604056
47	1	0	8.456917	1.234359	-4.805389
48	6	0	10.753570	-0.369378	-6.477162
49	1	0	11.802800	-0.137316	-6.318622
50	1	0	10.498089	-0.876335	-7.403189
51	16	0	4.658170	-3.455930	-1.682909
52	6	0	5.421257	-4.740242	-2.608416
53	8	0	5.247320	-3.398097	-0.379315
54	8	0	3.239664	-3.544197	-1.817198
55	6	0	6.422535	-5.500538	-2.031336
56	6	0	6.986930	-6.533860	-2.758712
57	6	0	6.567843	-6.810089	-4.053643
58	6	0	5.559077	-6.024868	-4.612213
59	6	0	4.981687	-4.995875	-3.901426
60	1	0	6.750179	-5.282982	-1.021941
61	1	0	7.769812	-7.137363	-2.311963
62	6	0	7.169617	-7.922297	-4.843913
63	1	0	5.225529	-6.233010	-5.623829
64	1	0	4.195664	-4.390094	-4.337376
65	1	0	6.417129	-8.675707	-5.091495
66	1	0	7.573239	-7.556366	-5.791315

67	1	0	7.974717	-8.416520	-4.299692

TS$_D$ (Table S-3) Imaginary frequency: 269 cm^{-1}.

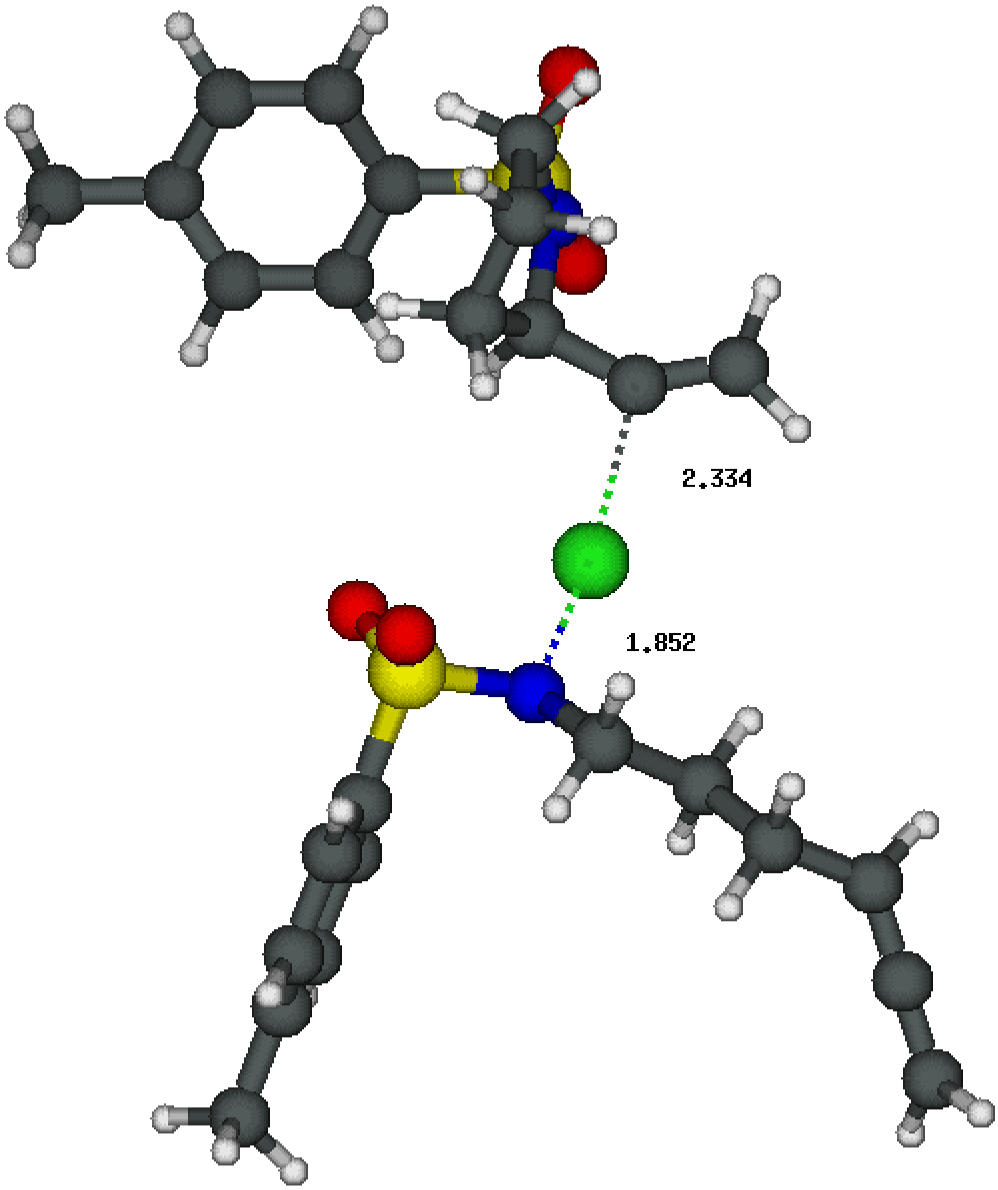

1	7	0	0.068217	-0.074631	0.032705
2	6	0	0.112477	-0.081572	1.503354
3	6	0	1.594161	-0.197227	1.799908
4	1	0	-0.353057	0.818839	1.903882
5	1	0	-0.425425	-0.953443	1.899630
6	6	0	2.091320	-1.087458	0.676157
7	1	0	2.068840	0.786587	1.741609
8	1	0	1.784958	-0.606082	2.791783
9	6	0	1.346171	-0.542385	-0.542305
10	1	0	3.172368	-1.079734	0.539749
11	1	0	1.776012	-2.122482	0.841628
12	6	0	2.037881	0.587903	-1.188009
13	1	0	1.181984	-1.327792	-1.286947
14	6	0	1.919102	1.868390	-1.369415
15	1	0	2.656870	2.454075	-1.908973
16	1	0	1.042968	2.398844	-0.992680

17	16	0	-1.339540	-0.403721	-0.738160
18	6	0	-1.591592	-2.136793	-0.518677
19	8	0	-2.361884	0.286343	-0.015384
20	8	0	-1.116622	-0.160125	-2.128160
21	6	0	-2.293005	-2.597247	0.585329
22	6	0	-2.432208	-3.959348	0.785046
23	6	0	-1.879769	-4.875364	-0.101106
24	6	0	-1.188224	-4.391519	-1.209095
25	6	0	-1.042910	-3.036647	-1.425304
26	1	0	-2.746052	-1.890210	1.269784
27	1	0	-2.988413	-4.319527	1.644252
28	6	0	-2.024264	-6.345358	0.109368
29	1	0	-0.763957	-5.095340	-1.918271
30	1	0	-0.525028	-2.673248	-2.304824
31	1	0	-1.046811	-6.824121	0.211383
32	1	0	-2.604968	-6.569967	1.004673
33	1	0	-2.520053	-6.817430	-0.742739
34	17	0	3.869309	-0.395943	-2.248318
35	7	0	5.325723	-1.363836	-2.857897
36	6	0	6.533058	-0.544897	-2.747170
37	6	0	6.587409	0.498586	-3.837923
38	1	0	6.609082	-0.090165	-1.750678
39	1	0	7.389387	-1.222842	-2.850590
40	6	0	7.867912	1.313683	-3.759242
41	1	0	6.514543	0.005110	-4.812485
42	1	0	5.721590	1.164182	-3.758852
43	6	0	7.919011	2.382790	-4.805929
44	1	0	7.935623	1.776851	-2.766658
45	1	0	8.739135	0.658797	-3.858383
46	6	0	8.841624	2.493591	-5.713988
47	1	0	7.105410	3.108302	-4.802389
48	6	0	9.768051	2.602045	-6.616291
49	1	0	10.663381	3.192493	-6.444080
50	1	0	9.683377	2.109792	-7.580807
51	16	0	5.372278	-2.794211	-1.960809
52	6	0	6.496765	-3.716288	-2.953558
53	8	0	5.958729	-2.579677	-0.670326
54	8	0	4.072093	-3.378613	-2.054980
55	6	0	7.735800	-4.066425	-2.447802
56	6	0	8.595336	-4.818998	-3.229284
57	6	0	8.234555	-5.218839	-4.509301
58	6	0	6.981435	-4.847600	-4.996798
59	6	0	6.111334	-4.102869	-4.231060
60	1	0	8.016835	-3.754276	-1.449142
61	1	0	9.566369	-5.101872	-2.836945
62	6	0	9.152739	-6.034472	-5.355707
63	1	0	6.690658	-5.154247	-5.996624
64	1	0	5.137473	-3.818359	-4.612392
65	1	0	9.325502	-5.555448	-6.322659
66	1	0	10.118974	-6.186859	-4.873880
67	1	0	8.720999	-7.017430	-5.562788

Cpl [2a 1a•] (Table S-3)

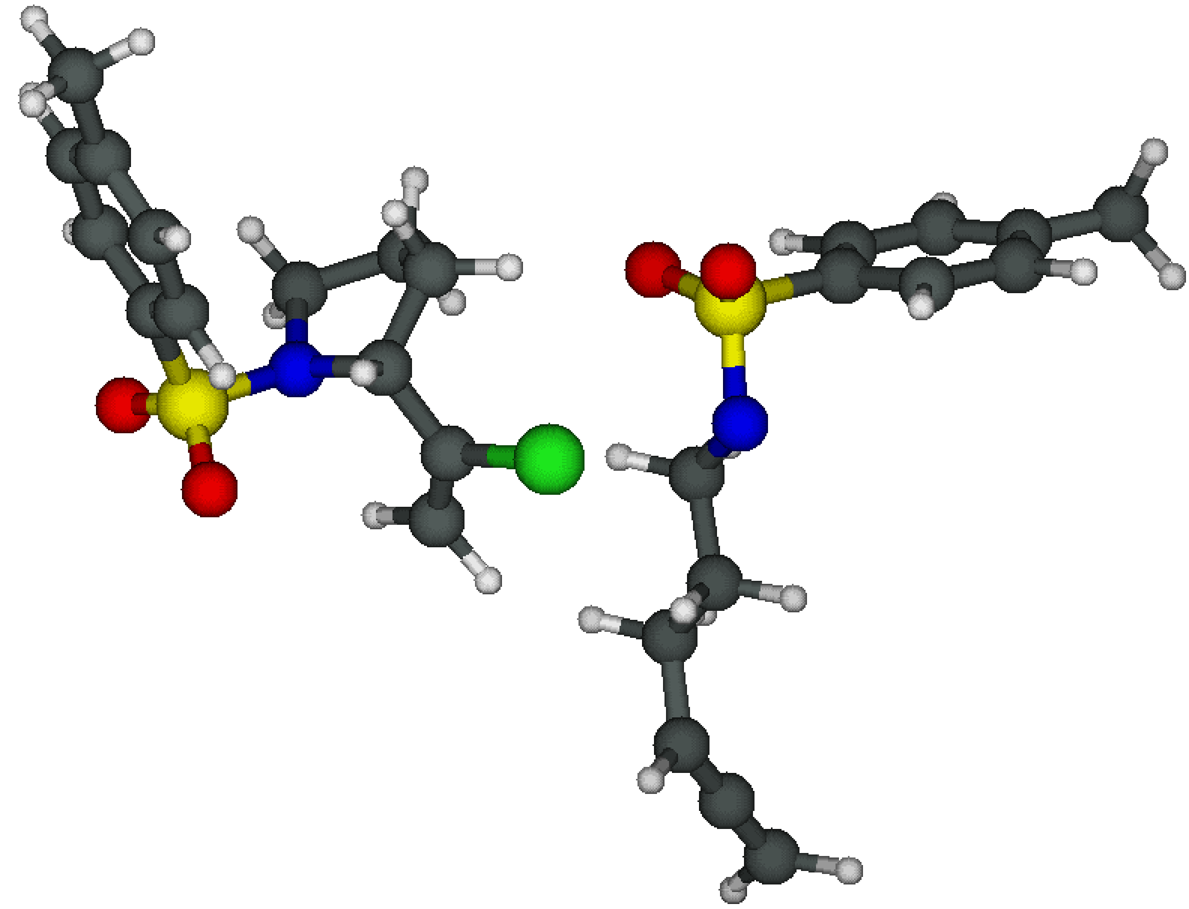

1	7	0	-0.018872	0.008175	-0.074490
2	6	0	-0.031080	0.061333	1.397476
3	6	0	1.442871	0.049665	1.749415
4	1	0	-0.565461	0.946793	1.742450
5	1	0	-0.531826	-0.824514	1.810086
6	6	0	2.035192	-0.835135	0.669776
7	1	0	1.863647	1.056639	1.679880
8	1	0	1.626875	-0.318785	2.758177
9	6	0	1.304260	-0.364095	-0.587118
10	1	0	3.118425	-0.762362	0.585795
11	1	0	1.772368	-1.882337	0.852345
12	6	0	1.972811	0.801730	-1.261648
13	1	0	1.227015	-1.172432	-1.322220
14	6	0	1.531463	2.042562	-1.341303
15	1	0	2.079434	2.811031	-1.870923
16	1	0	0.575718	2.300793	-0.902243
17	16	0	-1.368191	-0.451882	-0.876642
18	6	0	-1.498481	-2.192682	-0.612420
19	8	0	-2.464759	0.177344	-0.208977
20	8	0	-1.121403	-0.236128	-2.267697
21	6	0	-2.179236	-2.674461	0.495484
22	6	0	-2.225656	-4.037646	0.728020
23	6	0	-1.602817	-4.934537	-0.130515
24	6	0	-0.934354	-4.430531	-1.243621
25	6	0	-0.879388	-3.074072	-1.490941
26	1	0	-2.689998	-1.985253	1.157091
27	1	0	-2.765321	-4.414549	1.590563
28	6	0	-1.652044	-6.405822	0.112879

29	1	0	-0.458330	-5.121000	-1.932895
30	1	0	-0.380514	-2.695066	-2.374839
31	1	0	-2.120768	-6.926968	-0.725885
32	1	0	-0.646101	-6.819841	0.220264
33	1	0	-2.214131	-6.646882	1.015753
34	17	0	3.501398	0.364632	-1.987830
35	7	0	5.780672	1.926969	-0.407139
36	6	0	4.920488	3.053481	-0.235929
37	6	0	4.977966	3.975810	-1.431080
38	1	0	3.887697	2.708347	-0.047347
39	1	0	5.195296	3.591181	0.689190
40	6	0	4.070737	5.181637	-1.255386
41	1	0	6.010130	4.302962	-1.590152
42	1	0	4.692205	3.414040	-2.327827
43	6	0	4.069817	6.066955	-2.462520
44	1	0	3.047060	4.834755	-1.060864
45	1	0	4.374439	5.757421	-0.375481
46	6	0	4.413813	7.319831	-2.467720
47	1	0	3.769453	5.607255	-3.404267
48	6	0	4.754002	8.572082	-2.464807
49	1	0	4.024098	9.359130	-2.298566
50	1	0	5.780875	8.883379	-2.633190
51	16	0	5.840311	0.855026	0.866423
52	6	0	7.263789	1.511006	1.656913
53	8	0	4.709393	0.994368	1.740818
54	8	0	6.144814	-0.424842	0.309426
55	6	0	7.130435	2.275882	2.804992
56	6	0	8.263426	2.791886	3.405530
57	6	0	9.526108	2.564377	2.869297
58	6	0	9.632656	1.800289	1.707458
59	6	0	8.515421	1.274359	1.097563
60	1	0	6.147404	2.446435	3.227611
61	1	0	8.169363	3.381291	4.311364
62	6	0	10.751974	3.111757	3.517685
63	1	0	10.614269	1.619182	1.281361
64	1	0	8.598845	0.677795	0.196687
65	1	0	11.389793	2.303704	3.886469
66	1	0	11.350292	3.685787	2.805930
67	1	0	10.509432	3.759356	4.360587
