# Supplementary material for: Photoinduced Chloroamination Cyclization Cascade with N-Chlorosuccinimide: From N-(Allenyl)sulfonylamides to 2-(1-Chlorovinyl)pyrrolidines
Source: J Org Chem. 2022 Oct 26;88(10):6420–33. doi: 10.1021/acs.joc.2c01963 (PMC10204091; doi:10.1021/acs.joc.2c01963)

SUPPORTING INFORMATION AND EXPERIMENTAL PROCEDURES

Photoinduced Chloroamination Cyclization Cascade with *N*-Chlorosuccinimide: from *N*-(Allenyl)sulfonylamides to 2-(1-Chlorovinyl)pyrrolidines

Emanuele Azzi, Giovanni Ghigo, Lorenzo Sarasino, Stefano Parisotto, Riccardo Moro, Polyssena Renzi* and Annamaria Deagostino

[a]Department of Chemistry, University of Torino, Via P. Giuria 7, 10125, Turin, Italy

Table of Contents

S1. Materials and methods S1

S1.1. Characterization of photochemical setup S2

S2. Screening of the reaction conditions S3

S2.1. Optimization of the reaction conditions S3

S2.2. Thermal Experiments S7

S2.3. Control experiments S8

S2.4. Influence of the halogenating agent S8

S3. Mechanism investigation S10

S3.1. NMR monitoring S10

S3.2. ON/OFF Experiments S11

S3.3. Fluorescence quenching and Stern-Volmer experiments S11

S3.4. TEMPO trapping Experiments S12

S3.5. Determination of the Quantum Yield S13

S3.6. Empirical Identification of the reacting allene intermediate S15

S3.7. NMR-Determination of the isomers obtained in product 2k, 2l, 2m S16

S4. Experimental Procedures S19

S4.1 Procedures for the synthesis of allenes 1a-u S19

S4.1.1 General Procedure A: synthesis of allenes 1a, 1j S20

S4.1.2 General Procedure B: synthesis of internal allenes 1k, 1l, 1m, 1n, 1o, 1p, 1q S22

S4.1.3 Synthesis of alkoxy-allene 1r S24

S4.1.4 General Procedure C: synthesis of allenes 1b, 1c, 1d, 1e, 1f, 1g, 1h, 1i from nitrile S17. .... S26

S4.1.5 Synthesis of alkoxy allenes 1s, 1t. S29

S4.1.6 Synthesis of phenylthioallene 1u S30

S4.1.7 Synthesis of allenes for mechanistic studies: 3a and $1a^{-}Na^{+}$ S31

S4.2 ^{1}H-NMR Characterization of product 4a and 4b S32

S5 NMR characterization of new compounds S33

S1. Materials and methods

Flasks and all equipment used for the generation and reaction of moisture-sensitive compounds were dried by electric heat gun under vacuum and back filled with N_2, then used under N_2 atmosphere.

All commercially available reagents and solvents were used as received. Anhydrous solvents were purchased by Sigma-Aldrich or distilled as indicated by Armarego.[1] ZnI, NaI, aldehydes and NCS were purified as reported by Armarego.[1] All sulfonyl chlorides, aldehydes, ketones employed and compounds **5**, **10**, **14** were commercially available.

Products were purified by preparative column chromatography on Sigma-Aldrich silica-gel for flash chromatography, 0.04–0.063 mm/230–400 mesh. Reactions were monitored by TLC using silica-gel on TLC-PET foils Sigma-Aldrich, 2–25 μm, layer thickness 0.2 mm, medium pore diameter 60 Å.

NMR spectra were recorded employing a Jeol ECZR instrument. ^{1}H NMR spectra were recorded in $CDCl_3$ or DMSO-d_6 at 600 MHz. $^{13}C\{^{1}H\}$ NMR spectra were recorded in $CDCl_3$ at 150 MHz. Chemical shifts were reported in ppm relative to the resonance of $CHCl_3$ (δ= 7.26) for ^{1}H NMR, or referred to the central peak of $CDCl_3$ (δ= 77.0) for ^{13}C NMR. ^{13}C NMR spectra were measured with complete proton decoupling, thus ^{13}C NMR implies $^{13}C\{^{1}H\}$ NMR in the NMR characterization of new products. DEPT experiments were carried out with a DEPT-135 sequence. ^{1}H NMR coupling constants (*J*) were reported in Hertz (Hz) and multiplicities are indicated as follows s (singlet), bs (broad singlet), d (doublet), t (triplet), q (quartet), m (multiplet), dd (doublet of doublets), ddd (doublet of doublets of doublets), dm (doublets of multiplet), td (triplet of doublets), tm (triplet of multiplets). Structural assignments were made with additional information from gCOSY, gNOESY experiments.

UV–vis spectra were carried out with a Varian Cary “100 Scan” spectrophotometer. The extinctions were measured on freshly prepared and previously N_2 purged solutions. Optical path length: 1 cm. The solutions were stable during the timescales necessary for the measurements, and the results of repeated measures were reproducible.

Fluorescence emission spectra were collected with a Cary Eclipse Fluorescence Spectrophotometer, with excitation at 415 and 450 nm. Excitation and emission slits set both at 5 nm. Spectra were taken in a fluorescence fused silica cuvette with 1 cm optical path length.

IR spectra were recorded on a BrukerVertex 70 FT-IR.

HRMS spectra were obtained on a mass selective detector Agilent 5970 B operating at an ionizing voltage of 70 eV connected to a HP 5890 GC equipped with a HP-1 MS capillary column (25 m length, 0.25 mm I.D., 0.33 μm film thickness). The MS flow-injection analyses were run on a high resolving power hybrid mass spectrometer (HRMS) Orbitrap Fusion (Thermo Scientific, Rodano, Italy) and a Bruker Daltonics microTOF Mass Spectrometer equipped with an h-ESI ion source. The samples were analysed in methanol or acetonitrile solution using a syringe pump at a flow rate of 10 μL/min. The tuning parameters adopted for the ESI source were as follows: source voltage 3.5 kV, RF lens 60% (positive ion mode MH^+, MNa^+); source voltage 2.5 kV, RF lens 60%. The ion transfer tube was maintained at 270 °C. The mass accuracy of the recorded ions (vs the calculated ones) was <5 ppm. Analyses were run using full MS (50-500 m/z range) acquisition, at 240 000 resolution (200 m/z).

New compounds were fully characterized. For known compounds obtained with different procedures, the reference with full characterization was cited and only ^{1}H NMR characterization observed without experiments was reported.

Yields related to product **2a-u** obtained with the photochemical reaction describe herein are reported on the isolated product after silica gel chromatography purification.

For NMR yield determination, a solution containing nitromethane and 1,2-dichloroethane in $CDCl_3$ was used: a known volume of this standard solution was added to the crude of the photochemical reaction obtained after the workup, the resulting mixture was completely dissolved in $CDCl_3$. An aliquot of this solution was then transferred to the NMR tube for the ^{1}H NMR analysis. The proton signal related to the desired products and those of the standards (singlet at 3.71 ppm for 1,2-dichloroethane, 4H; and the singlet at 4.30 ppm for nitromethane, 3H) were integrated and the rate between these integration value was used to determine the yield of the desired product.

---

[1] Armarego, W. L. F., *Purification of laboratory chemicals*. Butterworth-Heinemann: **2017.**

For product **2a** the proton signals related to the vinyl proton at 5.57 (s, 1H) and at 5.33 (s, 1H) were considered. The yield values obtained considering these two different protons (typically not differing of more than 1-2%) were mediated for the final yield value. For allene **1a** the proton signal related to the allene system at 4.64 (dt, 2H) was considered. For allene **3a** the proton signal related to the allene system at 4.69 (dt, 2H) was considered. For by-product of the photochemical reaction the proton signal related to the vinyl proton at 5.23 was considered. Since the signals considered for the products and starting materials were distinguished and well separated. Thus, when a mixture of products and starting material was found, for each one of the molecule considered (**2a**, **3a**, byproduct, **1a** or starting material) the integral of the corresponding characteristic signal was compared to the integral of the standards, treating each molecule as the desired product (also **1a**, the starting material).

S1.1. Characterization of photochemical setup

Photochemical reactions: Photochemical reactions were carried out in a 10 mL Schlenk tube (cylindric shape, 11 cm heigt, 13 mm diameter). A Kessil Blue Lamp was used as irradiation source, which emits a band centered at 450 nm and of about 55 nm width to half height. The spectral irradiance of the source is reported (Figure S1) The irradiation source was located at 3 cm from the glass wall of the Schlenk tube. The reaction setup was continuously cooled with a fan.

Spectral irradiance of the 40W Kessil Tuna Blue LED (456 nm) was recorded with an Ocean Optics USB2000 spectrophotometer equipped with a cosine corrected probe and calibrated with a NIST traceable certified DH-2000 source (Ocean Optics). The data were acquired with the SpectraSuite software (Ocean Optics). The lamp employed has two emission bands, one centered at 450 nm and one at 396 nm (Figure S.1).

Quantum Yield Experiment: The experiment to determine the quantum yield was carried out in a cylinder-shaped photochemical reactor (4.4 cm as ID and 2.5 cm height; area of the photoreactor 15.21 cm^2). A Kessil Blue Lamp was used as irradiation source. The irradiation source was located at 9 cm from the surface of the reaction solution. The reaction setup was continuously cooled with a fan.

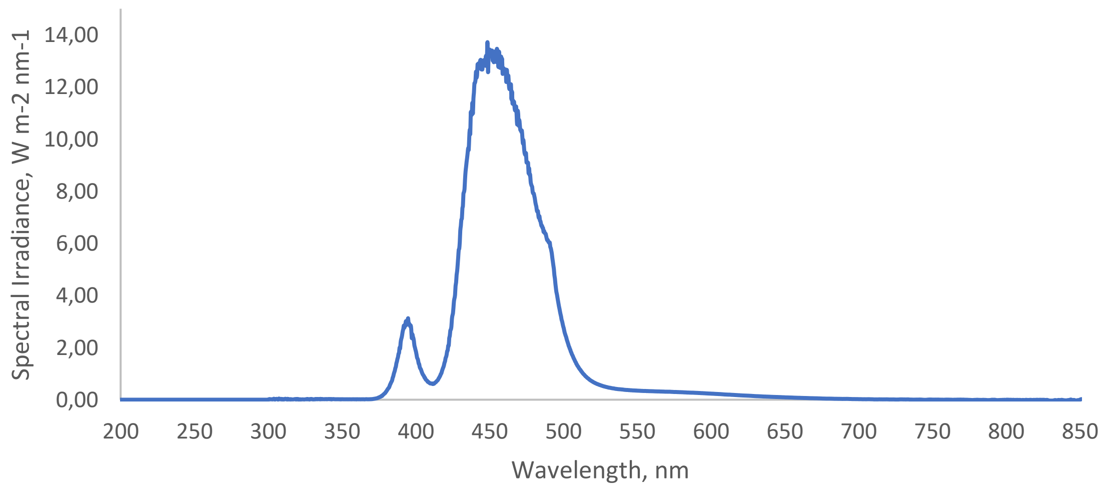

Figure S1 Measured emission for the 40W Kessil Tuna Blue LED (456 nm**) recorded** with the Ocean Optics USB2000 spectrophotometer.

S2. Screening of the reaction conditions

S2.1. Optimization of the reaction conditions

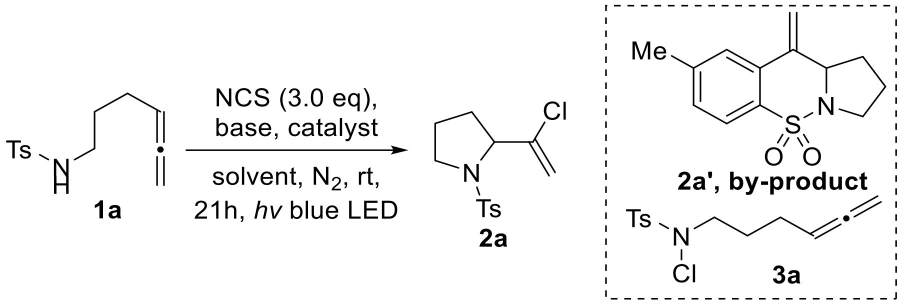

General procedure: A 10 mL Schlenk tube containing a magnetic stirring bar was dried with a heat gun under vacuum then the tube was backfilled with N_2. 4 mL of solvent were added *via* syringe and degassed with N_2 bubbling for 20 minutes. Then NCS, the catalyst and the base (when solid) were added in one portion, otherwise, the base was subsequently added *via* syringe under N_2 atmosphere. The resulting mixture was stirred and degassed with N_2 for additional 2 minutes and the allene **1a** (50 mg, 0.2 mmol) was added *via* syringe under N_2 atmosphere. The mixture was stirred and degassed with N_2 for additional 2 minutes. Finally, the tube was sealed and placed under irradiation with Kessil A160PR Blue LED (456 nm) placed at 3 cm distance for 21 h with continuous stirring. In order to analyze the crude mixture by ^{1}H NMR, the reaction was filtered through a thin pad of silica and eluted with EtOAc. In order to isolate the product **2a**, the solvent was removed and the crude mixture was purified by flash chromatography on silica gel chromatography (Eluent: EP 9/1 Acetone).

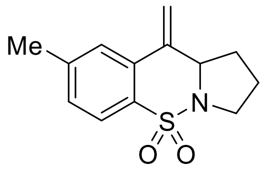

By-product **2a'** (8-methyl-10-methylene-2,3,10,10a-tetrahydro-1H-benzo [e]pyrrolo[1,2-b][1,2]thiazine 5,5-dioxide) was recovered in traces as a yellowish oil together with grease impurities (eluent: EP 92/8 Acetone), and preparative TLC (eluent EP 7:1 AcOEt) was necessary for characterization.

^{1}H NMR (600 MHz, $CDCl_3$, Me_4Si) δ 7.71 (d, *J* = 7.8 Hz, 1H, Ar-*H*), 7.32 (s, *J* = 1.7, 0.9 Hz, 1H, Ar-*H*), 7.27 – 7.23 (m, 1H, Ar-*H*), 5.57 (d, *J* = 1.3 Hz, 1H, C=CH_aH_b), 5.22 (d, *J* = 1.3 Hz, 1H, C=CH_aH_b), 4.75 (bt, *J* = 7.2, 1H, C-C*H*(CH_2)-N), 3.53 (m, 1H, N(CH)-CH_aH_b-CH_2), 2.95 (m, 1H, m, 1H, N(CH)-CH_aH_b-CH_2), 2.42 (s, 3H, Ar-CH_3), 2.39 – 2.29 (m, 1H, C-CH(CH_bH_a)-N), 1.92 – 1.80 (m, 1H, C-CH(CH_bH_a)-N), 1.77 – 1.63 (m, 2H, CH_2-CH_2-CH_2).

^{13}C{^{1}H} NMR (151 MHz, $CDCl_3$, Me_4Si) δ 143.4 (Cq), 141.2 (Cq), 140.8 (Cq), 135.5 (Cq), 129.2 (CH), 127.5 (CH), 124.6 (CH), 115.2 (CH_2), 64.9 (CH), 50.3 (CH_2), 35.5 (CH_2), 24.2 (CH_2), 21.8 (CH_3).

HRMS (ESI) *m/z*: $[M + H]^+$ Calcd for $C_{13}H_{16}NO_2S$ 250.0896, found 250.0897.

IR v max (neat)/ cm^{-1}: 2964, 1655, 1328, 1178, 1132

Table S1 Optimization of the catalyst

Entry	photocatalyst	1a [%][b]	2a [%][a]	by-product [%][b]	3a [%][b]
1	Mes-(Acr)-Me$^+$ BF_4^-	6	29	1	14
2	$[Ru(bpy)_3]Cl_2$	0	33	2	0
3	**$[Ru(bpy)_3](PF_6)_2$**	**0**	**43**	**4**	**0**
4	$[Ir(dtbbpy)(ppy)_2]PF_6$	0	15	<1	0
5	$[Ir(dFCF_3ppy)_2(bpy)]PF_6$	0	9	<1	0
6	Rhodamine 6G	17	17	3	26
7	Eosin Y (Green Light used)	18	12	4	12
8	Rhodamine B (Green Light used)	15	30	2	37
9	4-CzIPN	0	24	1	0

Reactions conditions: **1a** (1.0 eq, 0.20 mmol, 50 mg), photocatalyst (0.05 eq., as indicated), K_2CO_3 (1.0 eq, 0.20 mmol, 28 mg) as indicated, in anhydrous CH_3CN 4 mL under irradiation with 456 nm light source – blue light. *a)* Yield determined on isolated products. *b)* Yield determined by ^1H NMR analysis, using dichloroethane and nitromethane as internal standards

Table S2 Optimization of the base

Entry	base	1a [%][b]	2a [%][a]	by-product [%][b]	3a [%][b]
1	**K_2CO_3**	**0**	**43**	**4**	**0**
2	Cs_2CO_3	25	27	3	0
3	Na_2CO_3	15	23	1	0
4	$NaHCO_3$	13	25	<1	0
5	Li_2CO_3	4	27	2	0
6	KOH	0	29	1	0
7	NaOH	0	17	<1	0
8	K_3PO_4	0	27	0	0
9	Na_2HPO_4	0	16	0	0
10	NaH_2PO_4	0	8	0	0
11	2,6-Lutidine	10	10	<1	0
12	Et_3N	0	12	<1	0

Reactions conditions: **1a** (1.0 eq, 0.20 mmol, 50 mg), $[Ru(bpy)_3](PF_6)_2$ (0.05 eq., 0.01 mmol, 8 mg), base (1.0 eq, 0.20 mmol) as indicated, in anhydrous CH_3CN 4 mL under irradiation with 456 nm light source – blue light. *a)* Yield determined on isolated products. *b)* Yield determined by ^1H NMR analysis, using dichloroethane and nitromethane as internal standards

Table S3 Optimization of the solvent and solvent mixtures

Entry	solvent	1a [%][b]	2a [%][a]	by-product 2a' [%][b]	3a [%][b]
1	CH_3CN	0	43	4	0
2	$CHCl_3$	0	28	2	0
3	CH_2Cl_2	0	34	3	0
4	PhCl	0	34	3	0
5	1,2-Dichloroethane	0	26	1	0
6	DMF	0	28	Not obs	0
7	DMA	0	20	Not obs	0
8	DMSO	100	0	0	0
9	1,4-Dioxane	0	5	6	0
10	THF	0	15	6	0
11	CH_3OH	0	14	1	0
12	$PhCH_3$	0	47	5	0
13	HCO_2CH_3	0	31	4	0
14	Acetone	0	43	2	0

Entry	Solvent mixture [solvent 1/ solvent 2] ratio [1:2]	2a [%][a]	by-product [%][b]
M-1	$PhCH_3/CH_3CN$ 3:1	46	4
M-2	$PhCH_3$DMSO 3:1	0	0
M-3	$PhCH_3$/PhCl 3:1	49	2
M-4	**$PhCH_3$/ HCO_2Me 3:1**	**56**	**5**
M-5	$PhCH_3$/ DMF 3:1	32	0
M-6	$PhCH_3$/Acetone 3:1	45	3
M-7	$PhCH_3$/Acetone 2:2	42	3

Reactions conditions: **1a** (1.0 eq, 0.20 mmol, 50 mg), $[Ru(bpy)_3](PF_6)_2$ (0.05 eq., 0.01 mmol, 8 mg), K_2CO_3 (1.0 eq, 0.20 mmol, 28 mg) in anhydrous solvent (4 mL in total) as indicated under irradiation with 456 nm light source – blue light. *a)* Yield determined on isolated products. *b)* Yield determined by ^{1}H NMR analysis, using dichloroethane and nitromethane as internal standards

Table S4 Optimization of loading of the base and of the catalyst

Entry	$[Ru(bpy)_3](PF_6)_2$	K_2CO_3	2a [%][a]	by-product [%][b]
1	5% mol	1.0 eq.	56%	5
2	5% mol	0.5 eq.	52%	4
3	5% mol	0.3 eq.	53%	4
4	**5% mol**	**0.2 eq.**	**56%**	**5**
5	5% mol	0.1 eq.	51%	4
6	8% mol	0.2 eq.	56%	4
7	4% mol	0.2 eq.	49%	4
8	3% mol	0.2 eq.	49%	3
9	2% mol	0.2 eq.	49%	4
10	1% mol	0.2 eq.	51%	4
11	0.5% mol	0.2 eq.	39%	3

Reactions conditions: **1a** (1.0 eq, 0.20 mmol, 50 mg), $Ru(bpy)_3](PF_6)_2$ and K_2CO_3 as indicated in anhydrous solvent ($PhCH_3$ 3 mL and HCO_2Me 1mL) under irradiation with 456 nm light source – blue light. *a)* Yield determined on isolated products.

Table S5 Optimization of Catalyst/Base Combination (less efficient catalysts)

Base	1a [%][b]	2a [%][a]	by-product [%][b]	3a [%][b]
		Catalyst: $[Ru(bpy)_3]Cl_2$		
K_2CO_3	0	**33**	2	0
Cs_2CO_3	20	**22**	1	0
KOH	0	**12**	<1	0
NaOH	0	**18**	<1	0
2,6-Lutidine	10	**10**	1	0
		Catalyst: Mes-(Acr)-Me^+ BF_4^-		
K_2CO_3	6	**29**	<1	14
Cs_2CO_3	17	**11**	<1	0
KOH	0	**0**	0	0
NaOH	0	**14**	0	0
2,6-Lutidine	0	**13**	1	0
		Catalyst: Rhodamine B (using green lamp)		
K_2CO_3	15	**30**	2	37
Cs_2CO_3	20	**5**	5	0
2,6-Lutidine	80	**9**	traces	0
		Catalyst: $[Ir(dtbbpy)(ppy)_2][PF_6]$		
K_2CO_3	0	15	<1	0
K_3PO_4	0	13	<1	0
Na_2HPO_4	0	traces	0	0
NaH_2PO_4	0	traces	0	0
		Catalyst: potassium 5-bromo-1H-indole-1-carbodithioate		
2,6-lutidine	0	**18**	0	0
		Catalyst: 4Cz-IPN		
K_2CO_3	0	**18**	0	0
K_3PO_4	0	**traces**	0	0

*Note: With Na_2CO_3 $NaHCO_3$ and Li_2CO_3 degradation of the starting material **1a** was observed and not even traces of product recovered*

Reactions conditions: **1a** (1.0 eq, 0.20 mmol, 50 mg), catalyst (0.05 eq., 0.01 mmol) and base (1.0 eq, 0.20 mmol) as indicated in anhydrous CH_3CN 4 mL under irradiation with 456 nm light source – blue light. *a)* Yield determined on isolated products. *b)* Yield determined by ^1H NMR analysis, using dichloroethane and nitromethane as internal standards

S2.2. Thermal Experiments

The possibility of a thermal and polar pathway for the reaction studied was tested repeating the model reaction with all the reagents and catalyst in the absence of light irradiation at different temperatures. Computational calculations suggested a possible polar pathway in a total a polar media, thus experiments in $PhCH_3$ as the solvent with catalytic and stoichiometric amount of base were also repeated at different temperatures.

Table S6 Thermal experiments

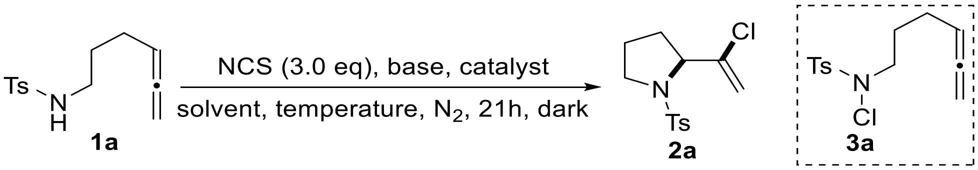

	1a [%]	solvent	T	catalyst	base	2a [%]	3a [%]
1	20	$PhCH_3/HCO_2CH_3$ 3:1	45°C	$[Ru(bpy)_3](PF_6)_2$ (5% mol)	K_2CO_3 (0.20 eq.)	0	80
2	19	$PhCH_3/HCO_2CH_3$ 3:1	60°C	$[Ru(bpy)_3](PF_6)_2$ (5% mol)	K_2CO_3 (0.20 eq.)	0	81
3	13	$PhCH_3/HCO_2CH_3$ 3:1	90°C	$[Ru(bpy)_3](PF_6)_2$ (5% mol)	K_2CO_3 (0.20 eq.)	3	78
4	9	$PhCH_3$ (4 mL)	45°C	/	K_2CO_3 (0.20 eq.)	0	91
5	9	$PhCH_3$ (4 mL)	60°C	/	K_2CO_3 (0.20 eq.)	0	91
6	20	$PhCH_3$ (4 mL)	90°C	/	K_2CO_3 (0.20 eq.)	7	25
7	9	$PhCH_3$ (4 mL)	45°C	/	K_2CO_3 (1.0 eq.)	0	91
8	14	$PhCH_3$ (4 mL)	60°C	/	K_2CO_3 (1.0 eq.)	0	86
9	27	$PhCH_3$ (4 mL)	90°C	/	K_2CO_3 (1.0 eq.)	2	65

Standard reaction conditions: **1a** (0.20 mmol), K_2CO_3 (as indicated), NCS (3.0 eq., 0.60 mmol) $[Ru(bpy)_3](PF_6)_2$ (5 mol%, 0.01 mmol, when indicated) and 4 mL of solvent (anhydrous $PhCH_3$ (3 mL), anhydrous HCO_2Me (1 mL); or 4 mL anhydrous $PhCH_3$) under irradiation with 456 nm light source – blue light. Yields determined after 2 repetitions with ^1H NMR using dichloroethane and nitromethane as internal standards.

S2.3. Control experiments

Table S7 Control experiments

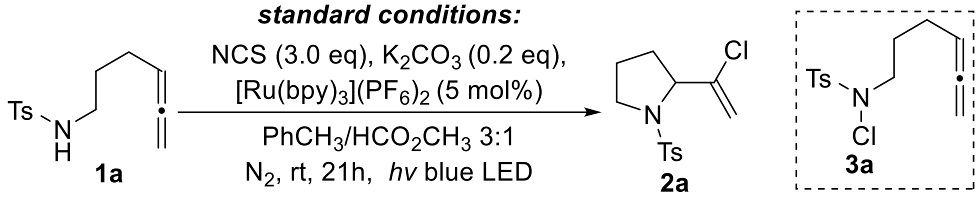

	1a [%]	Deviation from standard conditions	2a [%]	3a [%]	by-product [%]
1	*0*	*None*	*56*	*0*	*0*
2	100	no Base	0	0	0
3	100	No NCS	0	0	0
4	13	nocatalyst (PC)	11	66	<1
5	0	Presence of 1.00 eq of H_2O	29	0	<1
6	18	no irradiation (in the dark)	traces	82	0
7	0	White LED instead of Blue LED	49	0	2

Standard reaction conditions: **1a** (0.20 mmol), K_2CO_3 (0.20 eq., 0.04 mmol), NCS (3.0 eq., 0.60 mmol) $[Ru(bpy)_3](PF_6)_2$ (5 mol%, 0.01 mmol), anhydrous $PhCH_3$ (3 mL), anhydrous HCO_2Me (1 mL) under irradiation with 456 nm light source – blue light. Yields determined after 2 repetitions with ^{1}H NMR using dichloroethane and nitromethane as internal standards.

S2.4. Influence of the halogenating agent

The influence of the chlorinating agent was evaluated testing the effects of different loading of NCS and other commercially available chlorine donors.

Table S8 Evaluation of the influence of NCS

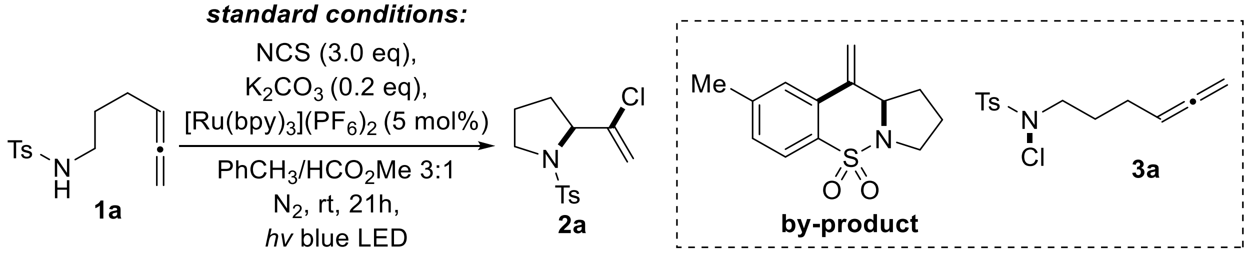

Entry	1a [%]	Deviation from standard conditions	2a [%]	3a [%]	by-product [%]
1	*0*	*None*	*56*	*0*	*4*
2	90	0.25 eq. NCS	5	0	7
3	72	0.50 eq. NCS	10	0	4
4	30	1.00 eq. NCS	41	8	8
5	29	2.00 eq. NCS	45	5	6

Standard reaction conditions: **1a** (0.20 mmol), K_2CO_3 (0.20 eq., 0.04 mmol), NCS (3.0 eq., 0.60 mmol) $[Ru(bpy)_3](PF_6)_2$ (5 mol%, 0.01 mmol), anhydrous $PhCH_3$ (3 mL), anhydrous HCO_2Me (1 mL) under irradiation with 456 nm light source – blue light. Yields determined after 2 repetitions with ^{1}H NMR using dichloroethane and nitromethane as internal standards.

Table S9 Different chlorine donors tested

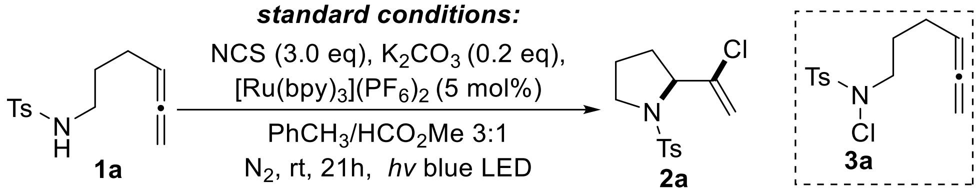

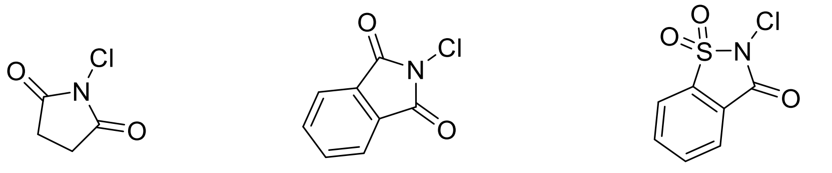

NCS *N-Chlorophthalimide* *N-Chlorosaccharin*

	1a [%]	Deviation from standard conditions	2a [%]	3a [%]	by-product [%]
1	*0*	*None*	*56*	*0*	*4*
2	0	3.00 eq. of *N*-chlorophtalimide	41	0	7
3	0	3.00 eq. of *N*-chlorosaccharin	30	0	4
4	92	3.00 eq. of NaClO (11-15% aq)	4	3	nd

Standard reaction conditions: **1a** (0.20 mmol), K_2CO_3 (0.20 eq., 0.04 mmol), NCS (3.0 eq., 0.60 mmol) $[Ru(bpy)_3](PF_6)_2$ (5 mol%, 0.01 mmol), anhydrous $PhCH_3$ (3 mL), anhydrous HCO_2Me (1 mL) under irradiation with 456 nm light source – blue light. Yields determined after 2 repetitions with ^{1}H NMR using dichloroethane and nitromethane as internal standards.

The model reaction was also repeated with NBS and NIS with related control experiments, confirming that for such halosuccinimides agent, the halocyclization is a completely polar pathway. Product **4a** and **4b** were isolated and NMR spectral data are coherent with those reported in literature, see paragraph S4.2.

Table S10 Reactions with other *N*-halosuccinimides

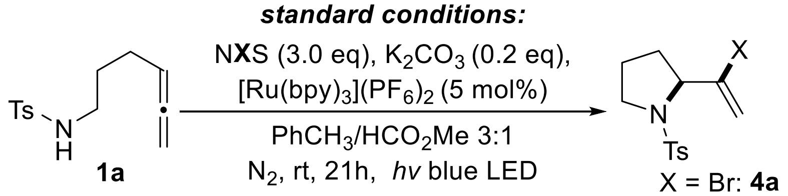

X = Br: **4a**
X = I: **4b**

	X =	Deviation from standard conditions	1a [%]	4 [%]
1	*Cl*	*None*	*0*	*56*
2	Br	3.00 eq. of NBS	0	58
3	Br	3.00 eq. of NBS, no base	0	57
4	Br	3.00 eq. of NBS, no catalyst	0	55
5	Br	3.00 eq. of NBS, no irradiation (in the dark)	0	58
6	I	3.00 eq. of NIS	0	66
7	I	3.00 eq. of NIS, no base	0	65
8	I	3.00 eq. of NIS, no catalyst	0	64
9	I	3.00 eq. of NIS, no irradiation (in the dark)	0	66

Standard reaction conditions: **1a** (0.20 mmol), K_2CO_3 (0.20 eq., 0.04 mmol), NXS (3.0 eq., 0.60 mmol) $[Ru(bpy)_3](PF_6)_2$ (5 mol%, 0.01 mmol), anhydrous $PhCH_3$ (3 mL), anhydrous HCO_2Me (1 mL) under irradiation with 456 nm light source – blue light. Yields determined after on isolated product

S3. Mechanism investigation

S3.1. NMR monitoring

The thermal evolution of the starting material, in the absence of the catalyst, was studied recording ^{1}H NMR spectra adding a component at a time to the allene **1a** in the NMR tube. Four different solutions were prepared in NMR tubes with the same molar concentration of the model reaction, in $CDCl_3$ as the solvent. The ^{1}H NMR spectra were recorded every 60 minutes for each solution.

- Solution A: allene **1a** (9.4 mg, 0.037 mmol) in 0.750 mL of $CDCl_3$ (**1a** molarity=0.05 M);

- Solution B: allene **1a** (9.4 mg, 0.037 mmol), NCS (15.0 mg, 0.112 mmol) in 0.750 mL of $CDCl_3$ (**1a** molarity=0.05 M, NCS molarity=0.15 M);

- Solution C: allene **1a** (9.4 mg, 0.037 mmol), NCS (15.0 mg, 0.112 mmol) and K_2CO_3 (10 mg, 0.072 mmol, an excess was poured in the tube due to poor solubility in $CDCl_3$) in 0.750 mL of $CDCl_3$ (**1a** molarity=0.05 M, NCS molarity=0.15 M, for K_2CO_3 not determinable);

- Solution D: allene **1a** (9.4 mg, 0.037 mmol), NBS (20.0 mg, 0.112 mmol) in 0.750 mL of $CDCl_3$ (**1a** molarity=0.05 M, NCS molarity=0.15 M);

Once the solutions were prepared, the tube was sealed, and the NMR experiment immediately recorded (time zero). See the Figure 1 (top) in the manuscript.

After 60 minutes from the time zero experiment, another experiment was repeated for each solution and so on every 60 minutes. No changes were observed in solutions A, B and C after 60 minutes. Comparison and stacking of spectra of solution A, B and C confirmed that NCS alone does not influence allene **1a** whereas the presence of the base and NCS combined immediately triggered the formation of **3a** species. For solution D, the presence of NBS alone is able to start the reaction of bromocyclization to product **4a** with no base or irradiation required. See the Figure 1 (bottom) in the manuscript

Experiment repeated after 60 minutes: For solution D the reaction achieved complete conversion of **1a** with bromocyclization to product **4a** after 1 h, thus confirming that for NBS the mechanism is polar and does not require neither the presence of a base nor irradiation. For solutions A and B no changes were observed. Also, for solution C no changes were observed, the species **3a** seemed not to be able to evolve to the desired product **2a** in the absence of light and catalyst after 60 minutes.

Experiment repeated after 120, 180, 240, 300, 360 minutes: No changes were observed in solutions A, B, C, D in further experiments repeated after 120, 180, 240 and 300 minutes since time zero.

S3.2. ON/OFF Experiments

To confirm the fundamental role of the light in promoting the chlorocyclization reaction, we set a light/dark experiment on the model reaction between allene **1a** and K_2CO_3 (0.20 eq., 0.04 mmol), NCS (3.0 eq., 0.60 mmol) $[Ru(bpy)_3](PF_6)_2$. The model reaction was set up according to the general procedure reported in "Experimental Section and/or Computational Methods" paragraph of the manuscript the reaction mixture under stirring was submitted to light/dark cycles of variable time length according to Table S11.

Table S11 Results in term of yield obtained for the light/dark experiment.

Irradiation ON/OFF cycle	Total reaction time [min]	2a [%]	3a [%]	1a [%]
Start	0	***0***	70	30
Light ON for 30 min	30	***8***	66	22
Light OFF for 30 min	60	***8***	64	22
Light ON for 30 min	90	***12***	64	16
Light OFF for 30 min	120	***12***	65	14
Light ON for 60 min	180	***24***	54	12
Light ON for 60 min	240	***25***	54	10
Light ON for 60 min	300	***37***	35	9

For each point, 0.2 mL of crude reaction mixture were withdrawn under N_2 with a syringe. The solution was filtered over a short pad of silica (5 mm), eluted with AcOEt (5 mL), and evaporated. After solvent removal, the crude was analysed by ^1H NMR dissolving the crude in 0.700 mL of a standard solution containing 1,2-dichloroethane as internal standard in $CDCl_3$ (0.02 M solution of DCE in $CDCl_3$)

S3.3. Fluorescence quenching and Stern-Volmer experiments

To investigate the reaction mechanism, some quenching experiments were carried out. The three species identified as possible reactant **1a**, **3a** and **1a$^-$Na$^+$** and NCS **155 were tested** as the quenchers of the photoexcited state of $[Ru(bpy)_3](PF_6)_2$.

Given the difficulty of replication of our reaction conditions in the fluorimeter cell, due to uncomplete solubility, the analyses were carried out in acetonitrile, since complete conversion and modest yield of the product were recovered also with this more polar solvent, as reported in Table 1 of the manuscript and in Table S1

The luminescence emission spectra of a 5×10^{-5} M solution of $[Ru(bpy)_3](PF_6)_2$ in acetonitrile and in the presence of one of the possible quenchers were collected after sparging the solutions with N_2 (5 min, directly in cuvette) in order to remove oxygen.

The decrease of $[Ru(bpy)_3]^{2+}$ luminescence was not observed in the presence of different concentrations of **1**, NCS and **3a.** Conversely, a significant decrease of $[Ru(bpy)_3]^{2+}$ luminescence was reported in the presence of **1a$^-$Na$^+$** salt (Figure 2b left, manuscript). Luminescence quenching experiments were repeated with different concentration of **1a$^-$Na$^+$** salt and a Stern–Volmer plot for the $[Ru(bpy)_3]^{2+}$ luminescence quenching could be extrapolated by the results (Figure 2b right, manuscript, where R is the ratio [quencher]/[$[Ru(bpy)_3]^{2+}$]).

Also. to replicate the reaction conditions the luminescence emission spectra of a 5×10^{-5} M solution of $[Ru(bpy)_3](PF_6)_2$ in acetonitrile was collected:

- in the presence of allene **1a**, [**1a**] = $1x10^{-3}$;
- in the presence of allene **1a** and 3.0 eq. of NCS, [**1a**] = $1x10^{-3}$; [NCS] = $3x10^{-3}$;
- in the presence of allene **1a** and 3.0 eq. of NCS and 0.20 eq. of K_2CO_3,
  [**1a**] = $1x10^{-3}$; [NCS] = $3x10^{-3}$; $[K_2CO_3]$= $2x10^{-4}$

In none of three cases above luminescence quenching was observed.

S3.4. TEMPO trapping Experiments

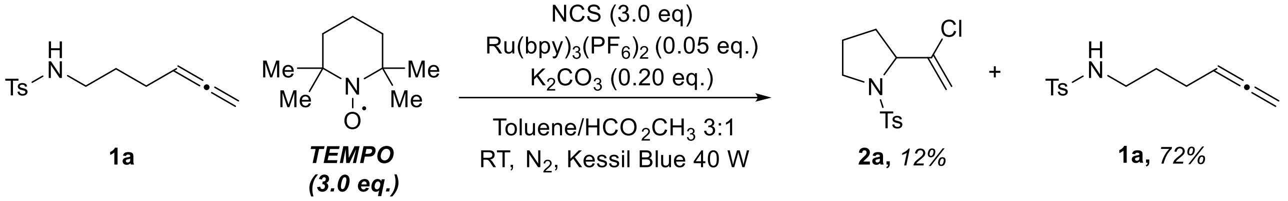

A 10 mL Schlenk tube containing a magnetic stirring bar was dried with a heat gun under vacuum then the tube was backfilled with N_2. 4 mL of a 3:1 mixture of $PhCH_3$ and HCO_2CH_3 were added *via* syringe and degassed with N_2 bubbling for 20 minutes. Then NCS (3.0 eq., 80.0 mg, 0.60 mmol), $[Ru(bpy)_3](PF)_6$ (0.05 eq., 8.6 mg, 0.01 mmol), K_2CO_3 (0.20 eq., 5.4 mg, 0.04 mmol) and TEMPO (3.0 eq., 94.0 mg, 0.60 mmol) were added in one portion.The resulting mixture was stirred and degassed with N_2 for additional 2 minutes and the allene **1a** (50 mg, 0.2 mmol) was added *via* syringe under N_2 atmosphere. The mixture was stirred and degassed with N_2 for additional 2 minutes. Finally, the tube was sealed and placed under irradiation with Kessil A160PR Blue LED (456 nm) placed at 3 cm distance for 21 h with continuous stirring. In order to analyze the crude mixture by ^{1}H NMR, the reaction was filtered through a thin pad of silica and eluted with EtOAc. ^{1}H NMR analysis with DCE and CH_3NO_2 as internal standard determined a yield of 12% of the product **2a**, with 72% of **1a** recovered and no formation of **3a** observed. To isolate the product **2a**, the solvent was removed and the crude mixture was purified by flash chromatography on silica gel chromatography (Eluent: EP 9/1 Acetone, yield 8%).

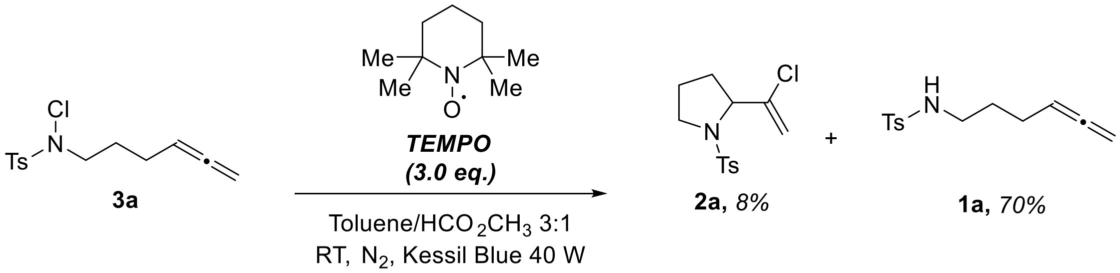

Once determined the possibility of a photolytic pathway starting from the chlorinate sulfonamide **3a**, a similar experiment was repeated. A 10 mL Schlenk tube containing a magnetic stirring bar was dried with a heat gun under vacuum then the tube was backfilled with N_2. 4 mL of a 3:1 mixture of $PhCH_3$ and HCO_2CH_3 were added *via* syringe and degassed with N_2 bubbling for 20 minutes. Then TEMPO (3.0 eq., 94.0 mg, 0.60 mmol) was added. The resulting mixture was stirred and degassed with N_2 for additional 2 minutes and the chlorinated species **1a** (50 mg, 0.2 mmol) was added *via* syringe under N_2 atmosphere. The mixture was stirred and degassed with N_2 for additional 2 minutes. Finally, the tube was sealed and placed under irradiation with Kessil A160PR Blue LED (456 nm) placed at 3 cm distance for 21 h with continuous stirring. In order to analyze the crude mixture by ^{1}H-NMR, the reaction was filtered through a thin pad of silica and eluted with EtOAc. ^{1}H NMR analysis with DCE and CH_3NO_2 as internal standard determined a yield of 8% of the product **2a**, with 70% of **1a** recovered.

S3.5. Determination of the Quantum Yield

The experiment to determine the quantum yield was carried out in a cylinder-shaped photochemical reactor (4.4 cm as ID and 2.5 cm height; area of the photoreactor 15.21 cm^2). The spectral irradiance was measured at the surface of the irradiated solution at the center of the cylindrical photoreactor. For Spectral irradiance of the 40 W Kessil Tuna Blue LED (456 nm) Lamp, see paragraph S 1.1. The irradiation source was located at 9 cm from the reaction solution surface. The total irradiance from 410 to 875 nm striking the reaction media surface, able to excite the $[Ru(bpy)_3](PF_6)_2$ is 344.55 W m^{-2}. The irradiance in the UV range (from 178 to 410 nm) is 16.54 W m^{-2}. Accordingly, the photon flow entering the surface of reaction milieu in the VIS range is $1.23x10^{18}$ photons s^{-1} (410-875 nm), while in the UV range is $5.01x10^{16}$ photons s^{-1} (178-410 nm). The moles of product were determined by NMR sampling the reaction after 63, 126, 199 and 401 minutes from its starting (for sampling procedure see the ON/OFF experiment). The reaction rate was evaluated with a linear regression of the time (min) vs the product amount (mmol) (Figure S2). The calculated rate is **$2.12x10^{-4}$ mmol min^{-1}**.

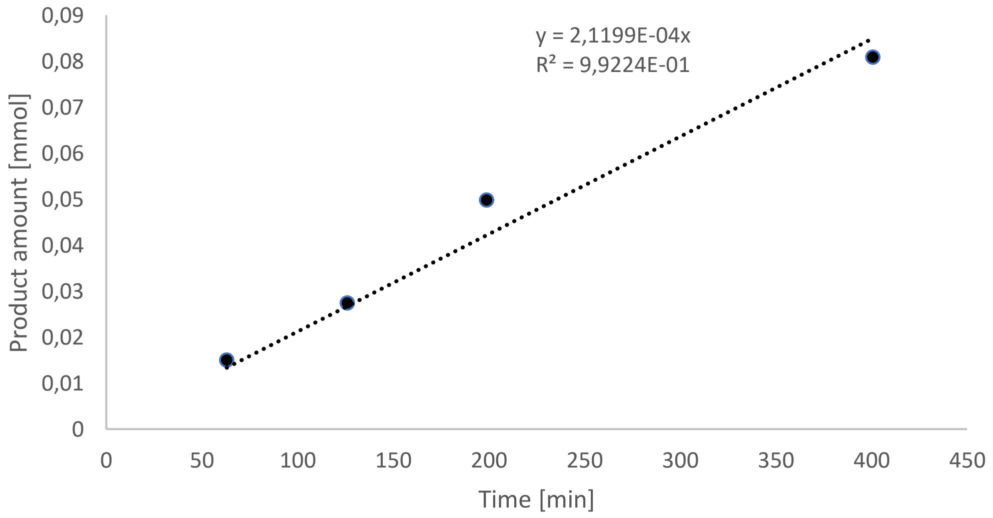

Figure S2 Determination of the reaction rate

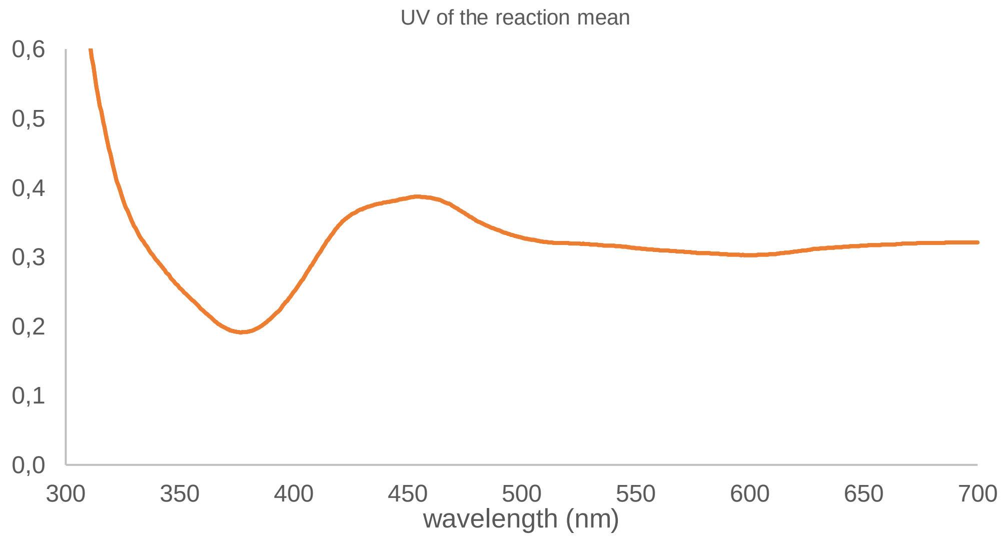

Figure S3 UV-Vis spectra of the reaction mixture

The moles of product per unit of time are related to the number of photons absorbed. The photons absorbed are correlated to the number of incident photons. From the UV-Vis spectra of the reaction mixture (Figure S3), we calculated the transmittance and then the absorbed fraction of the reaction solution.

Note 1: we approximate the irradiation as if all photons had a wavelength of 450 nm, where the catalyst absorb.

Note 2: the solution thickness in the reactor is 0.5 cm.

Absorbance at 450 nm: A_{1cm} = 0.336 a.u. (optical path 1 cm); $A_{0.5cm}$ = 0.336/2 = 0.168 a.u. (optical path 0.5 cm)

$$T = 10^{-A} = 10^{-0.168} = 0.68$$

$$Absorbed\ Fraction = 1 - T = 1 - 0.68 = 0.32$$

Therefore, only the 32% of the photons that beat the reactor are absorbed. In our case, in the VIS range the total number of photons that are absorbed is: **$3.92\text{x}10^{-2}$ mmol min^{-1}**.

The quantum yield (QY) of the reaction was calculated as follow:

$$QY = \frac{reaction\ rate\ (mmol\ min^{-1})}{absorbed\ photons\ (mmol\ min^{-1})} \times\ 100 = \frac{2.12 \times 10^{-4}}{3.92 \times 10^{-2}} \times 100 = \mathbf{0.54}\%$$

S3.6. Empirical Identification of the reacting allene intermediate

Table S12 Analysis of reactivity of **1a**$^-$**Na**$^+$

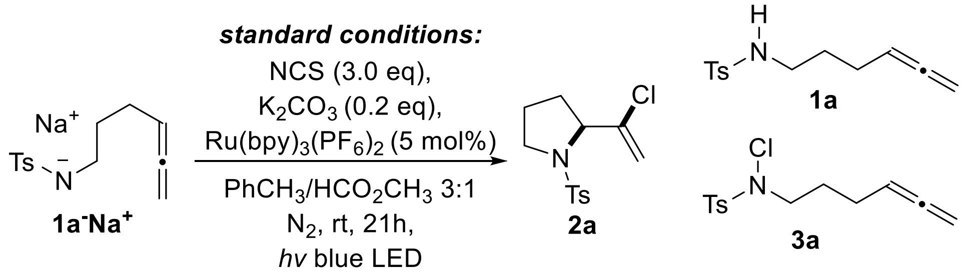

	**1a**$^-$**Na**$^+$ **[%]**	**Deviation from standard conditions**	**2a [%]**	**3a [%]**	**1a [%]**
1	0	None	56	0	0
2	0	no catalyst	13	72	8
3	0	no base, no catalyst	13	70	6
4	0	no base, no NCS	0	0	100
5	0	no base	45	10	0
6	0	no base, no irradiation(in the dark)	0	100	0

Standard reaction conditions:**1a** (0.20 mmol), K_2CO_3 (0.20 eq., 0.04 mmol), NCS (3.0 eq., 0.60 mmol) $[Ru(bpy)_3](PF_6)_2$ (5 mol%, 0.01 mmol), anhydrous $PhCH_3$ (3 mL), anhydrous HCO_2Me (1 mL) under irradiation with 456 nm light source – blue light. Yields determined after 2 repetitions with ^1H NMR using dichloroethane and nitromethane as internal standards.

Table S13 Analysis of reactivity of **3a**

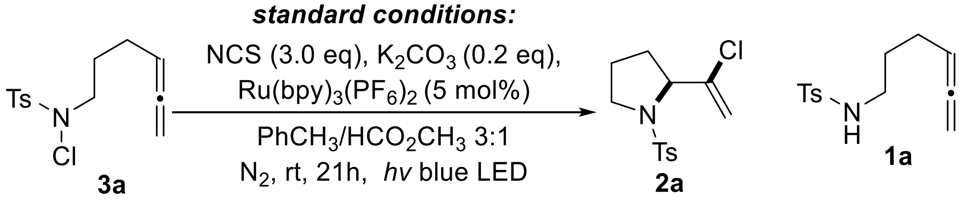

	**3a [%]**	**Deviation from standard conditions**	**2a [%]**	**1a [%]**
1	0	None	56	0
2	0	no catalyst	55	0
3	25	no base, no NCS	25	0
4	19	no base, no NCS, no catalyst	45	0
5	0	irradiation with Kessil Purple Light no base, no NCS, no catalyst	32	0
6	24	irradiation with Kessil Blue 440 nm Light (NO UV tail), no base, no NCS, no catalyst	39	0
7	100	no base, no NCS, no irradiation (in the dark)	0	0
8	0	NCS (2.0 eq), no base, no catalyst	40	0
9	23	no NCS, no catalyst	26	16

Standard reaction conditions: **1a** (0.20 mmol), K_2CO_3 (0.20 eq., 0.04 mmol), NCS (3.0 eq., 0.60 mmol) $[Ru(bpy)_3](PF_6)_2$ (5 mol%, 0.01 mmol), anhydrous $PhCH_3$ (3 mL), anhydrous HCO_2Me (1 mL) under irradiation with 456 nm light source – blue light. Yields determined after 2 repetitions with ^1H NMR using dichloroethane and nitromethane as internal standards.

S3.7. NMR-Determination of the isomers obtained in product 2k, 2l, 2m

A 1D-NOE experiment was conducted on the single isomer product **2m**. Signal at 4.7468 ppm was irradiated

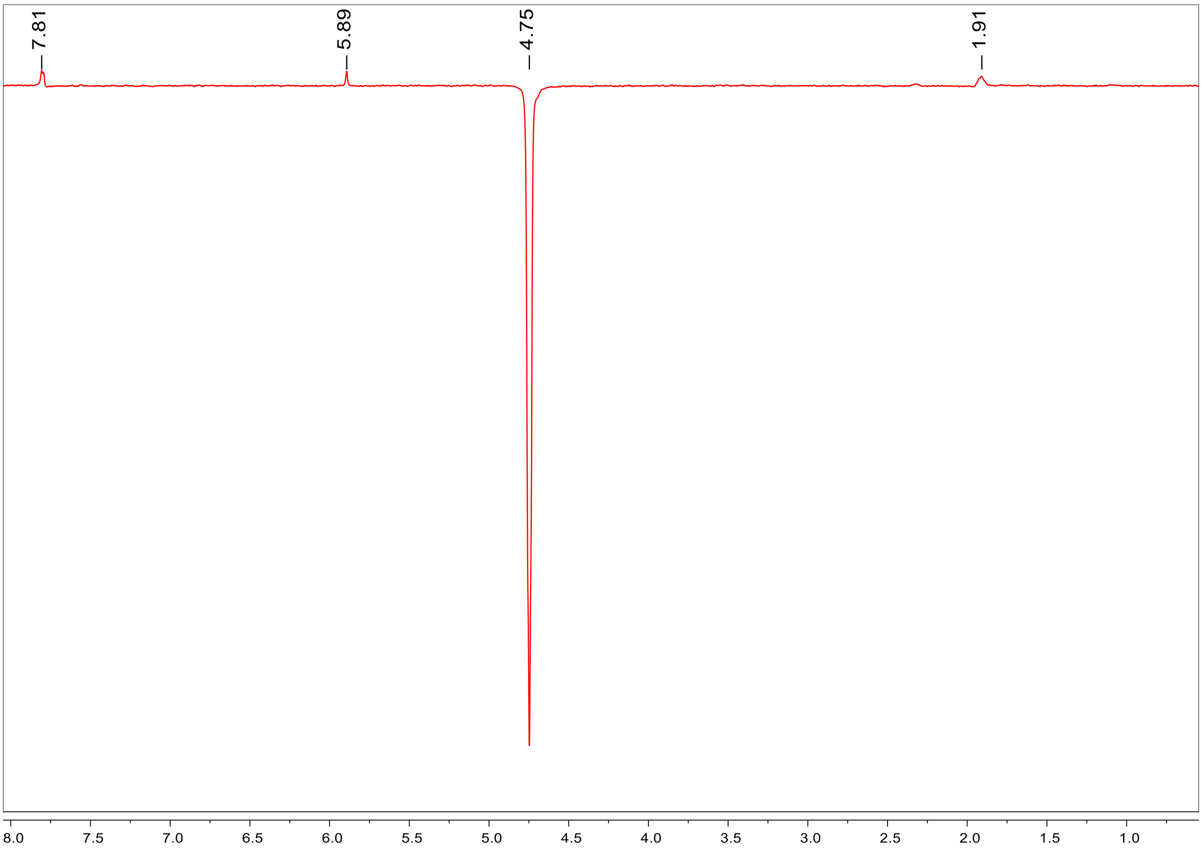

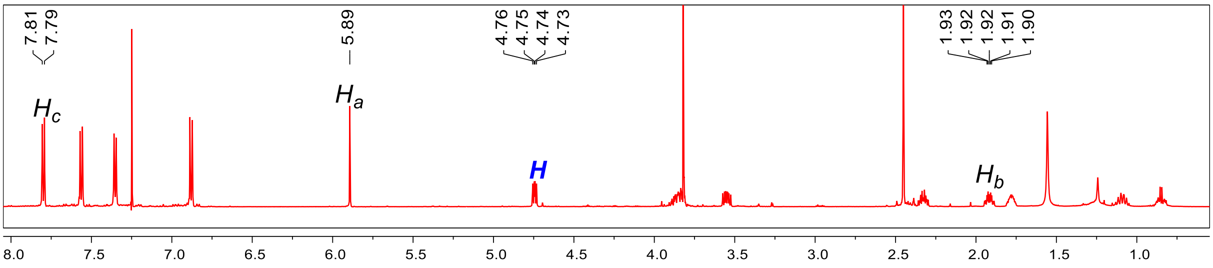

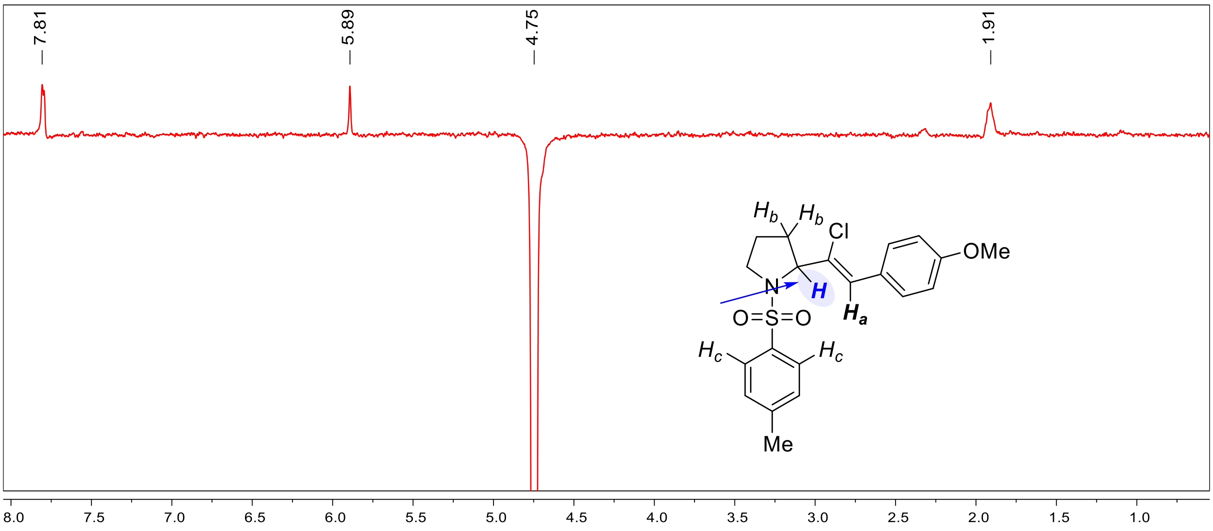

1D NOE findings were confirmed by 2D-NOESY experiments for compound **2m**

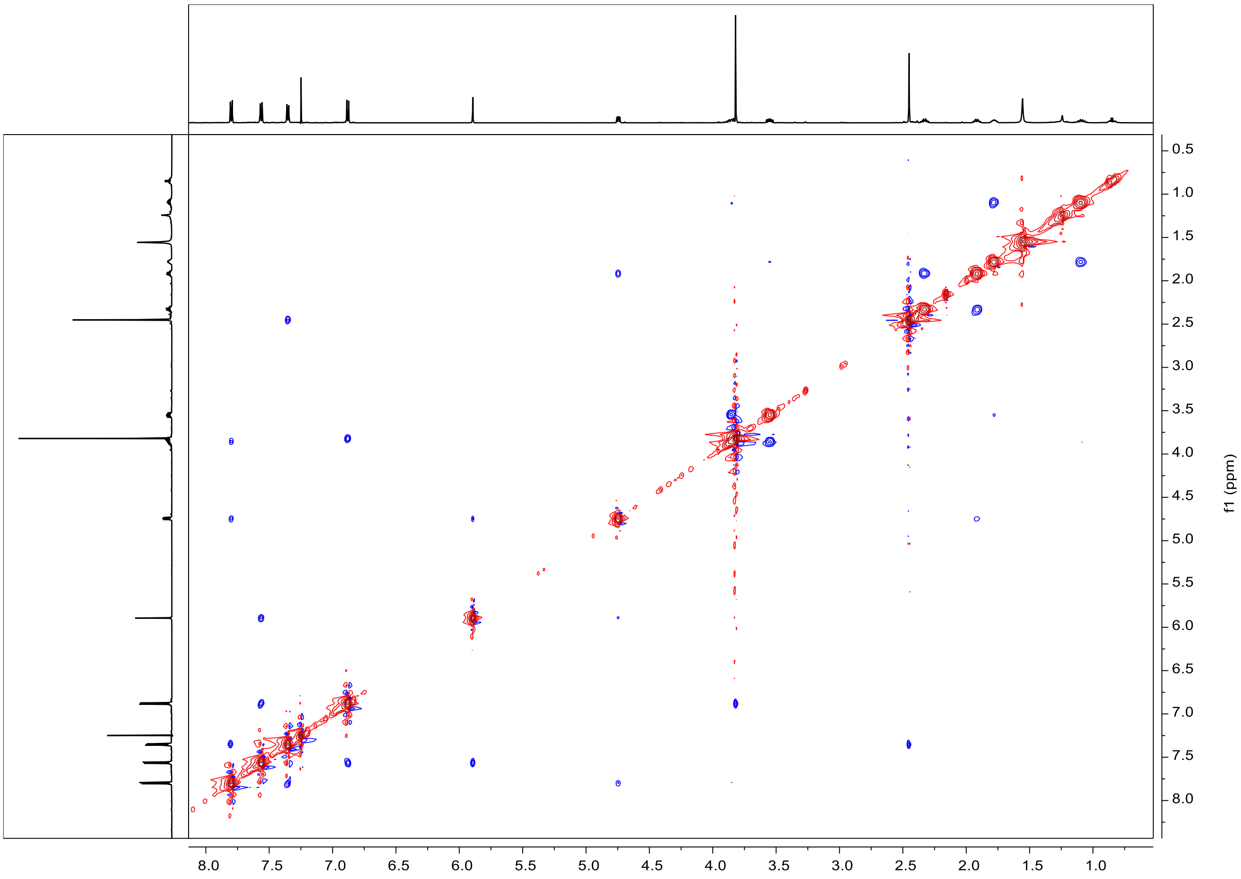

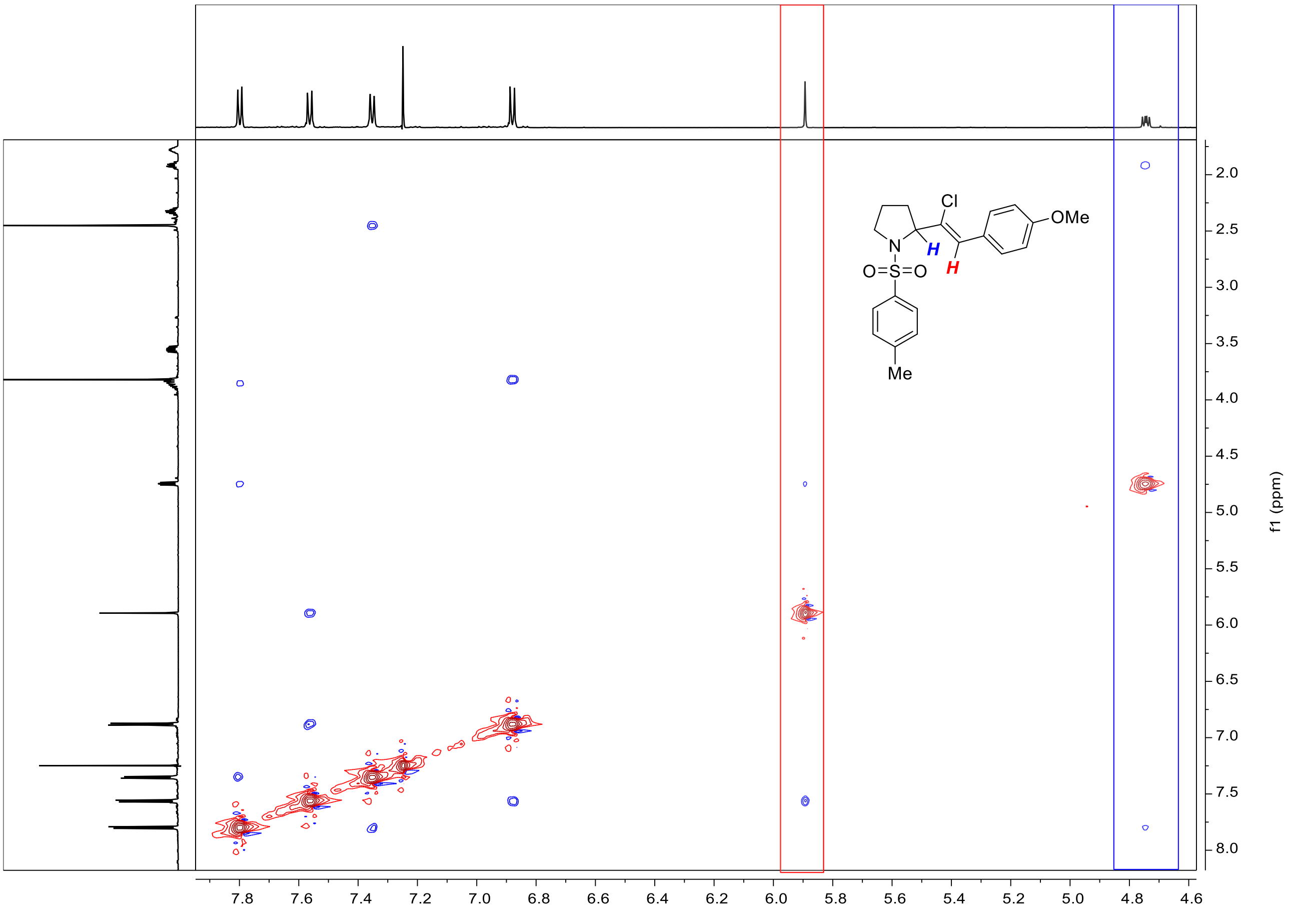

The NOESY experiment was repeated also for product **2k**, a mixture of isomers. The major isomer is the *Z* isomer, showing the same correlation observed for **2m**. The same deduction was extended also to product **2l**

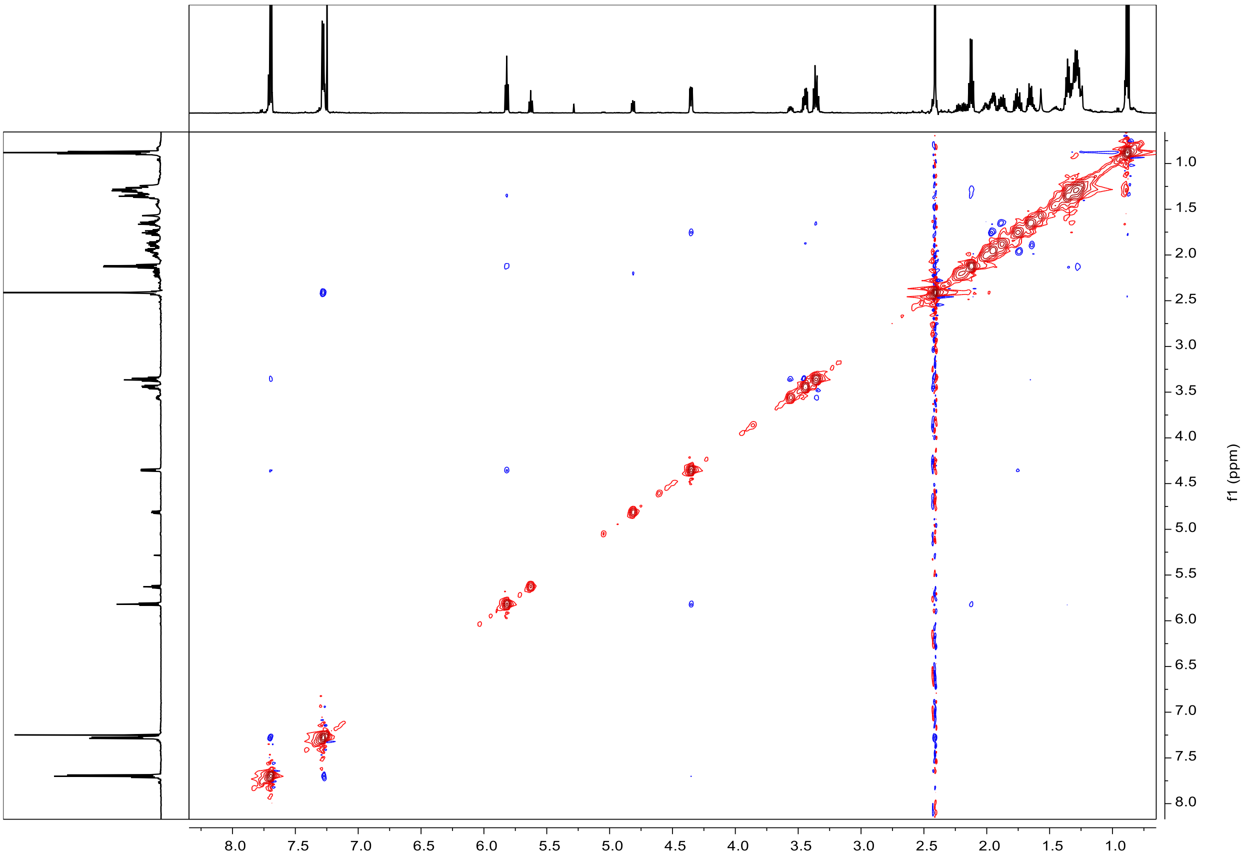

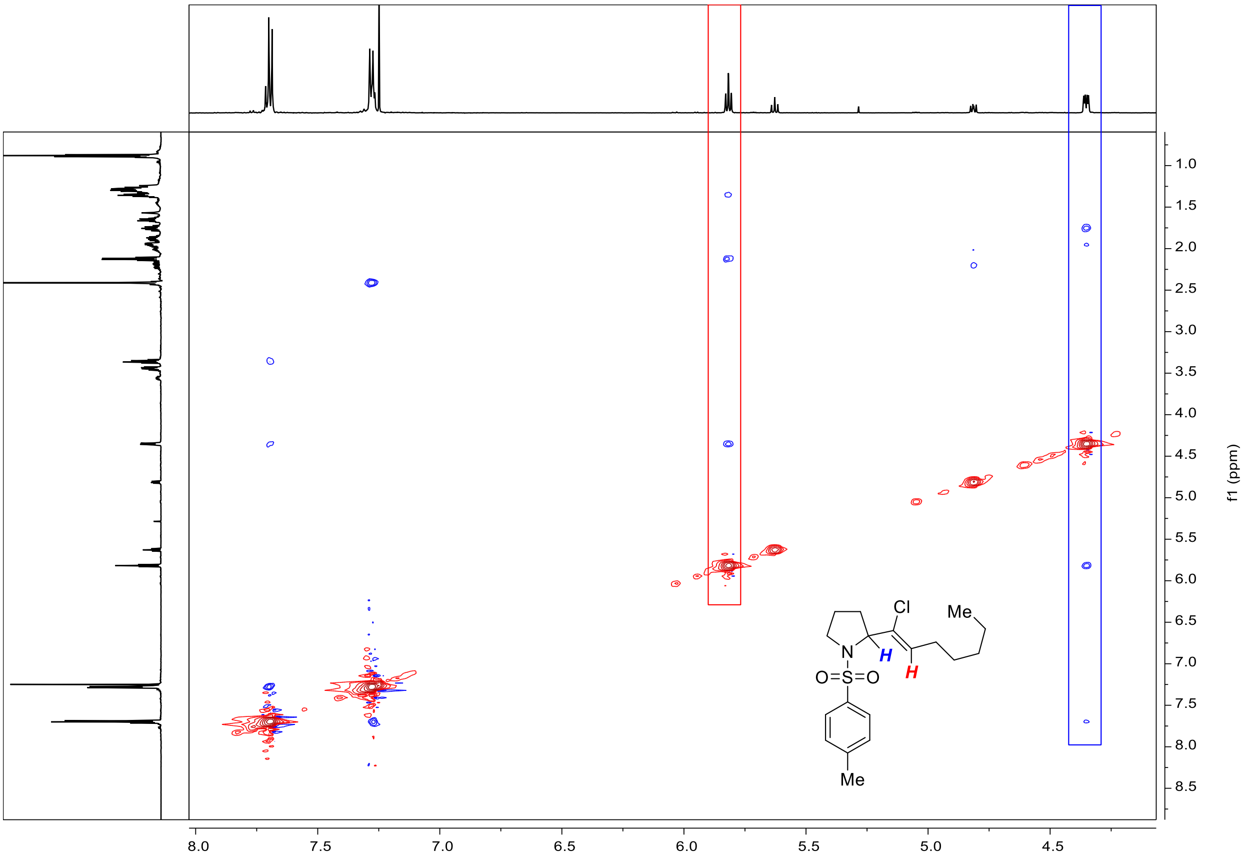

S4. Experimental Procedures

S4.1 Procedures for the synthesis of allenes 1a-u

Summary of allenes synthesized

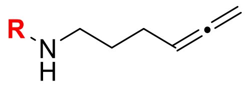

R = Tosyl, **1a**
R = benzenesulfonyl, **1b**
R = 2,4,6-trimethylbenzenesulfonyl, **1c**
R = 4-Chlorobenzenesulfonyl, **1d**
R = 4-Acetyl-benzenesulfonyl **1e**
R = 4-Methoxybenzenesulfonyl **1f**
R = 2,3-dihydrobenzo[b][1,4]dioxine-6-sulfonyl chloride **1g**
R = Methanesulfonyl **1h**
R = (1*S*)-(+)-10-Camphorsulfonyl chloride **1i**

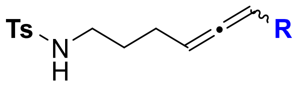

R = cyclohexyl **1k**
R = *n*-hexyl **1l**
R = methoxyphenyl **1m**

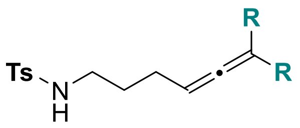

R = -Me **1n**
R = $-CH_2-(CH_2)_2-CH_2-$ **1o**
R = $-CH_2-(CH_2)_3-CH_2-$ **1p**
R = $-CH_2-CH_2-C($*t*$-Bu)H-CH_2-CH_2-$ **1q**

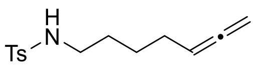

1j

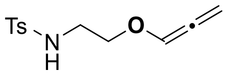

1r

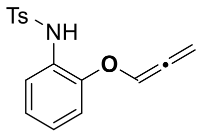

1s

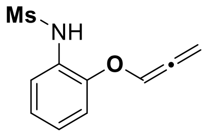

1t

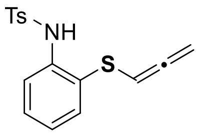

1u

Allenes synthesized for mechanistic studies

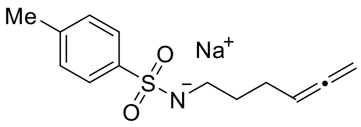

$1a^{-}Na^{+}$

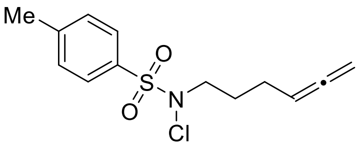

3a

S4.1.1 General Procedure A: synthesis of allenes 1a, 1j

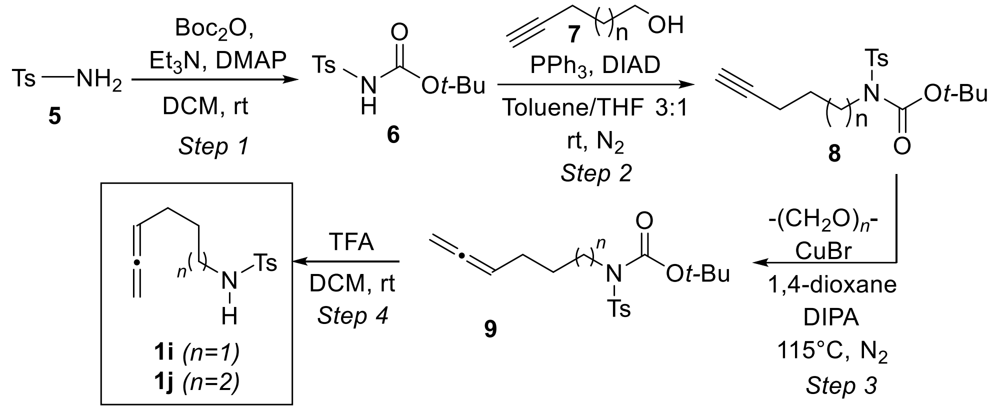

Allenes **1a** and **1j** were synthesized according to our previously reported procedure[2] from the commercially available alkynols **7i** and **7j**. Slight modifications to our previously described procedure were applied to achieve a larger scale preparation:

Step 1: ***t*-Butyl *N*-tosylcarbamate (6)**

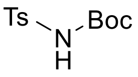

Following a reported procedure, *t*-Butyl *N*-tosylcarbamate **6** was obtained from tosylamide **5** as a white solid (yield 91%). Spectral data are coherent with those reported in literature.[3]
^{1}H NMR (600 MHz, $CDCl_3$, Me_4Si) δ: 7.89 (d, J= 8.3 Hz, 2H, Ar-*H*), 7.33 (d, *J* = 8.3 Hz, 2H, Ar-*H*), 2.44 (s, 3H; Ar-CH_3), 1.38 (s, 9H; C(CH_3)$_3$).

Step 2: Reaction was scaled to 30 mmol (1.0 eq. of alkyne **7**, 30.00 mmol; 1.05 eq. of *t*-butyl tosylcarbamate **6**, 8.80 g, 32 mmol; 1.22 eq of DIAD, 7.30 g, 36 mmol; 1.30 eq. of PPh_3, 10.45 g, 40 mmol; in 60 mL of a 3:1 mixture of anhydrous $PhCH_3$/THF). The crude white solid was purified by crystallization from ethanol (3 mL/mmol) to obtain the desired product in 92% yield from pent-4-yn-1-ol **7a** and in 90% yield from hex-5-yn-1-ol **7j**.

t-Butyl pent-4-yn-1-yl(*N*-tosyl)carbamate (8a)

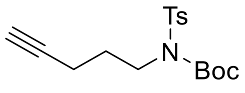

Following the described procedure, 2.60 g (30 mmol) of pent-4-yn-1-ol **7a** were reacted to afford 9.30 g of *t*-butyl pent-4-yn-1-l(*N*-tosyl)carbamate **8a** as a white solid after crystallization. Spectral data are coherent with those reported in literature.[4]
^{1}H NMR (600 MHz, $CDCl_3$, Me_4Si) δ: 7.78 (dm, *J* = 8.2 Hz, 2H, Ar-*H*), 7.30 (dm, *J* = 8.2, 2H, Ar-*H*), 3.92 (m, 2H, N-CH_2-CH$_2$), 2.44 (s, 3H, Ar-CH_3), 2.28 (td, *J* = 7.1, 2.7 Hz, 2H, CH_2-C≡CH), 2.02-1.96 (m, 3H, CH$_2$-CH$_2$-CH$_2$ and CH$_2$-C≡C*H*), 1.35 (s, 9H, C(CH_3)$_3$).

t-Butyl hex-5-yn-1-yl(*N*-tosyl)carbamate (8b)

Following the described procedure, 3.0 g (30 mmol) of hex-5-yn-1-ol **7b** were reacted to afford 9.5 g of *t*-Butyl hex-5-yn-1-yl(*N*-tosyl)carbamate **8b** as a white solid. Spectral data are coherent with those reported in literature.[4]

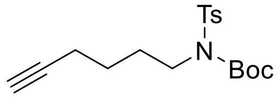

^{1}H NMR (600 MHz, $CDCl_3$, Me_4Si) δ: 7.78 (d, *J* = 8.3 Hz, 2H,Ar-*H*), 7.30 (dm, *J* = 8.3 Hz, 2H, Ar-*H*), 3.85 (m, 2H, N-CH_2-CH$_2$), 2.43 (s, 3H; Ar-CH_3), 2.26 (td, *J* = 7.0, 2.6 Hz, 2H,CH$_2$-CH_2-C≡), 1.97

[2] Renzi, P.; Azzi, E.; Bessone, E.; Ghigo, G.; Parisotto, S.; Pellegrino, F.; Deagostino, A. *Org. Chem. Front.* **2022,** *9*, 906-916.
[3] Shaw, P.; Hassell-Hart, S. J.; Douglas, G. E.; Malcolm, A. G.; Kennedy, A. R.; White, G. V.; Paterson, L. C.; Kerr, W. J. *Org. Lett.* **2022,** *24*, 2750-2755.
[4] Campbell, C. D.; Greenaway, R. L.; Holton , O. T.; Walker, P. R.; Chapman, H. A.; Russell, C. A.; Carr, G.; Thomson, A. L.; Anderson, E. A. *Chem. Eur. J.* **2015,** *21*, 12627-12639.

(t, *J* =2.7 Hz, 1H, C≡C*H*), 1.90-1.85 (m, 2H, CH_2-$C\mathit{H}_2$-CH_2), 1.59 (quin, *J* = 7.1 Hz, 2H, CH_2-$C\mathit{H}_2$-CH_2-C≡), 1.34 (s, 9H, $C(C\mathit{H}_3)_3$).

<u>*Step 3:*</u> reaction was scaled to 1.70 mmol of alkyne for each Pyrex tube (1.0 eq. of alkyne, 1.70 mmol; 2.0 eq. of paraformaldehyde, 100 mg, 3.35 mmol; 0.70 eq. of CuBr, 168 mg, 1.17 mmol; 2.0 eq. of DIPA, 340 mg, 0.470 mL, 3.35 mmol; in 17 mL of anhydrous 1,4-dioxane). The crudes were gathered, and the crude red oil obtained as product was used without further purification in the next step.

_t_-Butyl hexa-4,5-dien-1-yl(_N_-tosyl)carbamate (9a)

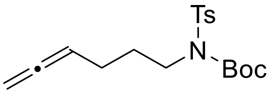

Following the described procedure, 1.90 g (5.64 mmol) of *t*-Butyl pent-4-yn-1-yl(*N*-tosyl)carbamate **8a** were reacted to afford *t*-butyl hexa-4,5-dien-1-yl(tosyl)carbamate **9a** as a red oil (yield 91% on the crude). Spectral data are coherent with those reported in literature.[2]

^{1}H NMR (600 MHz, $CDCl_3$, Me_4Si) δ: 7.77 (d, *J* = 8.3 Hz, 2H, Ar-*H*), 7.30 (d, *J* = 8.3 Hz, 2H, Ar-*H*), 5.14 (quin, *J* = 6.4 Hz, 1H, CH_2-C*H*=C=CH_2), 4.70 (dt, *J* = 6.3, 3.1 Hz, 2H, CH=C=$C\mathit{H}_2$), 3.87-3.83 (m, 2H, N-$C\mathit{H}_2$-CH_2), 2.43 (s, 3H, Ar-$C\mathit{H}_3$), 2.07 (m, 2H, CH_2-$C\mathit{H}_2$-CH), 1.88 (quin, *J* = 7.9 Hz, 2H,CH_2-$C\mathit{H}_2$-CH_2), 1.33 (s, 9H, $C(CH_3)_3$).

_t_-Butyl hepta-5,6-dien-1-yl(_N_-tosyl)carbamate (9b)

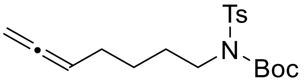

Following the described procedure, 1.98 g (5.64 mmol) of *t*-butyl hexa-4,5-dien-1-yl(tosyl)carbamate **8b** were reacted with paraformaldehyde to afford *t*-butyl hepta-5,6-dien-1-yl(*N*-tosyl)carbamate **9b** as a red oil which was used without further purification in step 4 (yield 63% on the crude). Spectral data are coherent with those reported in literature. [2]

^{1}H NMR (600 MHz, $CDCl_3$, Me_4Si) crude reaction mixture; δ: 7.77 (d, *J* = 8.4 Hz, 2H, Ar-*H*), 7.29 (d, *J* = 8.4 Hz, 2H, Ar-*H*), 5.10 (quin, *J* = 6.7 Hz, 1H, CH_2-C*H*=C=CH_2), 4.67 (dt, *J* = 6.7, 3.3 Hz, 2H, =C=$C\mathit{H}_2$), 3.82 (m, 2H, N-$C\mathit{H}_2$-CH_2), 3.70 (residue of dioxane), 2.44 (s, 3H, Ar-$C\mathit{H}_3$), 2.08-2.03 (m, 2H, CH_2-$C\mathit{H}_2$-CH), 1.82-1.77 (m, 2H, N-$C\mathit{H}_2$-CH_2), 1.50-1.45 (m, 2H, CH_2-$C\mathit{H}_2$-CH_2), 1.33 (s, 9H, $C(CH_3)_3$).

<u>*Step 4:*</u>

_N_-tosylhexa-4,5-dien-1-ylamine (1a)

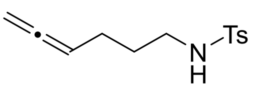

Following the described procedure, 1.80 g (5.11 mmol) of *t*-butyl hexa-4,5-dien-1-yl(tosyl)carbamate **9a** were reacted with TFA to afford 1.09 g of *N*-tosylhexa-4,5-dien1-ylamine **1a** as a yellowish oil after flash silica gel chromatography purification with EP/Acetone 85/15 (yield 94% on the crude, yield 85% over the two steps 3-4). Spectral data are coherent with those reported in literature.[2]

^{1}H NMR (600 MHz, $CDCl_3$, Me_4Si) δ: 7.74 (d, *J* = 8.3 Hz, 2H, Ar-*H*), 7.31 (d, *J* = 8.3 Hz, 2H, Ar-*H*), 5.02 (quin, *J* = 6.7 Hz, 1H, CH_2-C*H*=C=CH_2), 4.64 (dt, *J* = 6.7, 3.2 Hz, 2H, CH=C=$C\mathit{H}_2$), 4.30 (s broad, 1H, N*H*), 2.99 (q, *J* = 7.0 Hz, 2H, NH-$C\mathit{H}_2$-CH_2), 2.43 (s, 3H, Ar-$C\mathit{H}_3$), 1.99 (m, 2H, CH_2-$C\mathit{H}_2$-CH), 1.59 (quin, *J* = 7.1 Hz, 2H, CH_2-$C\mathit{H}_2$-CH_2).

_N_-tosylhepta-5,6-dien-1-ylamine (1j)

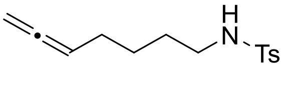

Following the described procedure, 1.86 g (5.11 mmol) of *t*-butyl hepta-5,6-dien-1-yl(tosyl)carbamate **9j** were reacted with TFA to afford 0.771 mg of *N*-tosylhepta-5,6-dien-1-ylamine **1j** as a yellow oil after chromatography purification with EP/Acetone 85/15 (yield 93% on the crude, yield 57% over the two steps 3-4). Spectral data are coherent with those reported in literature. [2]

^{1}H NMR (600 MHz, $CDCl_3$, Me_4Si) δ: 7.74 (d, *J* = 8.3 Hz, 2H, Ar-*H*), 7.31 (d, *J* = 8.3 Hz, 2H, Ar-*H*), 5.02 (quin, *J* = 6.7 Hz, 1H, CH_2-C*H*=C), 4.64 (dt, 2H, *J* = 6.7, 3.3 Hz, =C=$C\mathit{H}_2$), 4.27 (t, *J* = 6.2 Hz, 1H, NH), 2.95 (q, *J* = 6.8 Hz, 2H, NH-$C\mathit{H}_2$-CH_2), 2.43 (s, 3H, Ar-$C\mathit{H}_3$), 1.94 (m, 2H, CH_2-$C\mathit{H}_2$-CH), 1.50 (quin, *J* = 7.1 Hz, 2H, NH-CH_2-$C\mathit{H}_2$-CH_2), 1.39 (quin, *J* = 7.1 Hz, 2H, CH_2-$C\mathit{H}_2$-CH_2).

S4.1.2 General Procedure B: synthesis of internal allenes 1k, 1l, 1m, 1n, 1o, 1p, 1q

A procedure reported by Ma and coworkers was adapted.[5]

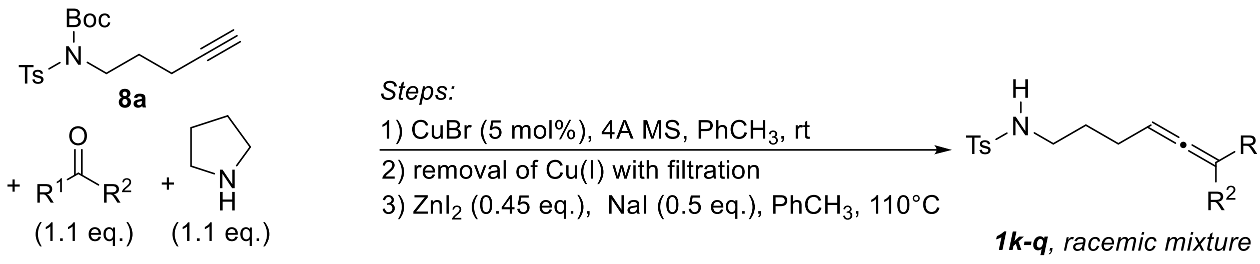

R^1 = alkyl, aryl; R^2= H or alkyl

The reaction seemed to be sensitive to scale up, so it was repeated on maximum 1 mmol of substrate (larger scale preparation performed by parallel repetitions).

Step 1: A 10 mL Schlenk tube (with screwing cap) containing a magnetic stirring bar was dried with a heat gun under vacuum then the tube was backfilled with N_2. CuBr (0.05 eq., 7.4 mg, 0.05 mmol), activated 4 Å molecular sieves (305 mg), and the alkyne **8a** (1.0 eq, 340 mg, 1.0 mmol) were added and subjected to two vacuum/N_2 cycles. Anhydrous $PhCH_3$ (2 mL), the appropriate carbonyl compound (ketone or aldehyde, 1.1 eq., 1.1 mmol) and the pyrrolidine (1.1 eq., 78 mg, 1.1 mmol) were added sequentially under N_2 atmosphere. The solution was then stirred at room temperature until completion (24h for aldehydes, 72 h for ketones).

Step 2: The crude reaction mixture was then filtered through a short pad of silica and eluted with AcOEt (40 mL). Then the solvent was evaporated, and the crude yellowish oil obtained subjected to the following step.

Step 3: A 10 mL Schlenk tube (with screwing cap) containing a magnetic stirring bar was dried with a heat gun under vacuum then the tube was backfilled with N_2. Anhydrous ground ZnI_2 (0.45 eq., 147.0 mg, 0.45 mmol) and NaI (0.5 eq., 75.2 mg, 0.5 mmol) were added. Subsequently the Schlenk tube was heated with a heating gun (350°C) and subjected to two vacuum/N_2 cycles. The crude oil product (or the isolated propargylamine intermediate) was then dissolved in $PhCH_3$ (5 mL) and added *via* syringe to the Schlenk tube and the tube sealed to be immerged in a pre-heated oil bath at 110 °C with vigorous stirring for 6 h. The resulting reaction mixture was then filtered through a short pad of silica gel and eluted with AcOEt (40 mL). The crude obtained after evaporation of the solvent was finally purified with flash silica gel chromatography to isolate the racemic mixture of not terminal allene.

_N_-(6-cyclohexylhexa-4,5-dien-1-yl)-4-methylbenzenesulfonamide (1k)

Following the described procedure, 0.360 g of *t*-butyl pent-4-yn-1- yl(tosyl)carbamate **8a** were reacted with 110 mg of hexanal to afford 135 mg of *N*-(6-cyclohexylhexa-4,5-dien-1-yl)-4-methylbenzenesulfonamide **1k** as a yellow oil (EP/Acetone 9/1, 40%).

^{1}H NMR (600 MHz, $CDCl_3$, Me_4Si) δ: 7.73 (d, *J* = 8.3 Hz, 2H, Ar-*H*), 7.29 (d, *J* = 8.3 Hz, 2H, Ar-*H*), 5.02 (m, 2H, CH_2-C*H*=C=C*H*-Cy), 4.50 (t, *J* = 6.2 Hz, 1H, N*H*), 2.97 (dq, *J* = 7.1, 1.6 Hz, 2H, NCH_2-CH_2-CH_2), 2.41 (s, 3H, Ar-CH_3), 1.99-1.92 (m, 2H, Cy=C=CH-CH_2), 1.92-1.85 (m, 1H, CH=C=CH-C_a(C_bH_2)H_bH_a), 1.71-1.65 (m, 5H, $(CH_2)_2$-CH_2-$(CH_2)_2$, $C_bH_bH_a$-CH_2-CH_2-CH_2-C_aH_2 $C_aH_bH_a$-CH_2-CH_2-CH_2-C_bH_2), 1.56 (quin, *J* = 7.1 Hz, 2H, NCH_2-CH_2-CH_2), 1.28-1.19 (m, 2H, CH=C=CH-C_a(C_bH_2)H_bH_a, $C_aH_bH_a$-CH_2-CH_2-CH_2-C_bH_2), 1.18-1.09 (m, 1H, CH=C=CH-C_b(C_aH_2)H_bH_a), 1.06-0.96 (m, 2H, CH=C=CH-C_b(C_aH_2)H_bH_a, $C_bH_bH_a$-CH_2-CH_2-CH_2-C_aH_2).

---

[5] Ye, J.; Li, S.; Chen, B.; Fan, W.; Kuang, J.; Liu, J.; Liu, Y.; Miao, B.; Wan, B.; Wang, Y.; Xie, X.; Yu, Q.; Yuan, W.; Ma, S. *Org. Lett.* **2012,** *14*, 1346-1349.

$^{13}C\{^1H\}$ NMR (151 MHz, $CDCl_3$, Me_4Si) δ: 202.8 (Cq), 143.4 (Cq), 137.1 (Cq), 129.8 (2 x CH), 127.2 (2 x CH), 98.0 (CH), 90.5 (CH), 42.8 (CH_2), 37.2 (CH), 33.2 (CH_2), 33.1 (CH_2), 28.9 (CH_2), 28.9 (CH_2), 26.2 (CH_2), 26.1 (CH_2), 26.1 (CH_2), 26.0 (CH_2), 21.6 (CH_3).
HRMS (ESI) *m/z*: $[M + H]^+$ Calcd for $C_{19}H_{28}NO_2S$ 334.1835, found 334.1828
IR ν max (neat)/ cm^{-1}: 3278, 2934, 2836, 1321, 1169.

4-methyl-*N*-(undeca-4,5-dien-1-yl)benzenesulfonamide (1l)

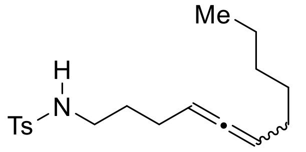

Following the described procedure, 0.360 g of *t*-butyl pent-4-yn-1-yl(tosyl)carbamate **8a** were reacted with 100 mg of hexanal to afford 100 mg of 4-methyl-*N*-(undeca-4,5-dien-1-yl)benzenesulfonamide **1l** as a yellow oil (EP/Acetone 9/1, 30%). Spectral data are coherent with those reported in literature.[6]

^1H NMR (600 MHz, $CDCl_3$, Me_4Si) δ: 7.73 (d, *J* = 8.3 Hz, 2H, Ar-*H*), 7.28 (d, *J* = 8.3 Hz, 2H, Ar-*H*), 5.01 (m, 1H, CH_2-CH_a=C), 4.95 (m, 1H, C=CH_b-CH_2), 4.83 (t, *J* = 6.2 Hz, 1H, N*H*), 2.94 (td, *J* = 7.0, 6.0 Hz, 1H, NH-CH_2-CH_2), 2.40 (s, 3H, Ar-CH_3), 2.02 – 1.84 (m, 4H, CH_2-CH_2-CH_2 and CH_2-CH_3), 1.60-1.49 (m, 2H, CH_2-CH_2-CH_2), 1.35-1.30 (m, 2H, CH_2-CH_2-CH_2), 1.30-1.20 (m, 4H, CH_2-CH_2-CH_2), 0.85 (t, *J* = 7.0 Hz, 3H, CH_2-CH_3).

***N*-(6-(4-methoxyphenyl)hexa-4,5-dien-1-yl)-4-methylbenzenesulfonamide (1m).**

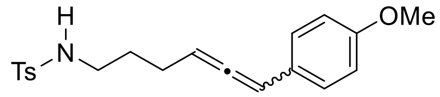

Following the described procedure, 0.360 g of *t*-butyl pent-4-yn-1-yl(tosyl)carbamate **8a** was reacted with 150 mg of 4-methoxybenzaldehyde to afford 147 mg of *N*-(6-(4-methoxyphenyl)hexa-4,5-dien-1-yl)-4-methylbenzenesulfonamide **1m** as a yellow oil (EP/Acetone 9/1, yield 40%). Spectral data are coherent with our previous report.[2]

^1H NMR (600 MHz, $CDCl_3$, Me_4Si) δ: 7.71 (d, *J* = 8.3 Hz, 2H,Ts-*H*), 7.25 (d, *J* = 8.3 Hz, 2H, Ts-*H*), 7.14 (d, *J* = 8.8 Hz, 2H, Ar-*H*), 6.82 (d, *J* = 8.8 Hz, 2H, Ar-*H*), 6.05 (dt, *J* = 6.4, 3.1 Hz, 1H, C=C*H*-Ph), 5.46 (q, *J* = 6.4 Hz, 1H, CH_2-C*H*=C), 4.69 (t, *J* = 6.3 Hz, 1H, N*H*), 3.01-2.93 (m, 2H; NH-CH_2-CH_2), 3.78 (s, 3H; OCH_3), 2.39 (s, 3H; Ar-CH_3), 2.20-2.05 (m, 2H; CH_2-CH_2-CH), 1.66-1.58 (m, 2H; CH_2-CH_2-CH_2).

4-methyl-*N*-(6-methylhepta-4,5-dien-1-yl)benzenesulfonamide (1n)

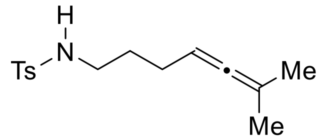

Following the described procedure, 340 mg of *t*-butyl pent-4-yn-1-yl(tosyl)carbamate **8a** was reacted with 175 mg an excess of anhydrous acetone (3.0 eq, 3.0 mmol) to afford 98 mg of 4-methyl-*N*-(6-methylhepta-4,5-dien-1-yl)benzenesulfonamide **1n** as colorless oil (EP/Acetone 9/1, yield 35%). Spectral data are coherent with those reported in literature.[6]

^1H NMR (600 MHz, $CDCl_3$, Me_4Si) δ: 7.74 (d, *J* = 8.3 Hz, 2H, Ar-*H*), 7.29 (d, *J* = 8.3 Hz, 2H, Ar-*H*), 4.83 (m, 1H, C*H*=C), 4.75 (t, *J* = 6.2 Hz, 1H, N*H*), 2.98 (m, 2H; NH-CH_2-CH_2), 2.40 (s, 3H, Ar-CH_3), 1.95 – 1.85 (m, 2H, CH_2-CH_2-CH), 1.60 (s, 3H, =C(CH_3)$_2$), 1.59 (S, 3H, =C(CH_3)$_2$) 1.52 (quint, *J* = 7.2 Hz, 2H, CH_2-CH_2-CH_2).

***N*-(5-cyclopentylidenepent-4-en-1-yl)-4-methylbenzenesulfonamide (1o)**

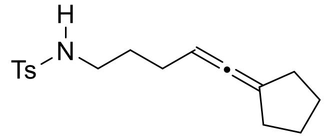

Following the described procedure, 340 mg of *t*-butyl pent-4-yn-1-yl(tosyl)carbamate **8a** was reacted with 90 mg of cyclopentanone to afford 35.2 mg of *N*-(5-cyclopentylidenepent-4-en-1-yl)-4-methylbenzenesulfonamide $$ **1o** as a yellow oil (EP/Acetone 9/1, yield 11%). Spectral data are coherent with those reported in literature.[7]

^1H NMR (600 MHz, $CDCl_3$, Me_4Si) δ: 7.73 (d, 2H, *J* = 8.4 Hz, Ar-*H*), 7.28 (d, 2H, *J* = 8.0 Hz, Ar-*H*), 4.98-4.92 (m, 1H, *J* = 6.3, 2.2 Hz, C=C=C-*H*), 4.78 (t, 1H, J = 6.0 Hz,), 4.72 (m, 1H, N-*H*),) 2.94 (q, 2H, *J* = 6.8 Hz, N-CH_2-CH_2), 2.40 (s, 3H, Ar-CH_3), 2.28-2.20 (m, 4H, =C=C-(CH_2)$_2$), 1.96-1.89 (m, 2H, N-CH_2-CH_2-CH_2), 1.64-1.58 (m, 4H, =C=C-(CH_2)$_2$)-(CH_2)$_2$), 1.53 (quint, 2H, *J* = 6.4 Hz, N-CH_2-CH_2-CH_2).

[6] Jonasson, C.; Horváth, A.; Bäckvall, J.-E. *J. Am. Chem. Soc.* **2000,** *122*, 9600-9609.
[7] LaLonde, R. L.; Sherry, B. D.; Kang, E. J.; Toste, F. D. *J. Am. Chem. Soc.* **2007,** *129*, 2452-2453.

N-(5-cyclohexylidenepent-4-en-1-yl)-4-methylbenzenesulfonamide (1p)

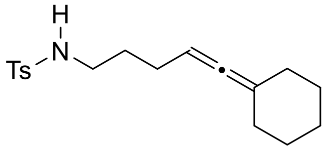

Following the described procedure, 340 mg of *t*-butyl pent-4-yn-1-yl(tosyl)carbamate **8a** was reacted with 100 mg of cyclohexanone to afford 103 mg of *N*-(5-cyclohexylidenepent-4-en-1-yl)-4-methylbenzenesulfonamide **1p** as a yellowish oil (EP/Acetone 9/1, 32%). Spectral data are coherent with those reported in literature.[7]

^{1}H NMR (600 MHz, $CDCl_3$, Me_4Si) δ: 7.73 (d, 2H, *J* = 8.4 Hz, Ar-*H*), 7.29 (d, 2H, *J* = 8.6 Hz, Ar-*H*), 4.86 (tt, 1H, *J* = 6.3, 2.2 Hz, C=C=C-*H*), 4.34 (bt, 1H, *J* = 6.0 Hz, N-*H*), 2.97 (q, 2H, *J* = 6.4 Hz, N-CH_2-CH_2), 2.41 (s, 3H, Ar-CH_3), 2.02-2.00 (m, 4H, =C=C-(CH_2)$_2$), 1.93 (q, 2H, *J* = 6.4 Hz, N-CH_2-CH_2-CH_2), 1.61-1.43 (m, 8H, N-CH_2-CH_2-CH_2, =C=C-(CH_2)$_2$-(CH_2)$_2$-CH_2).

N-(5-cyclohexylidenepent-4-en-1-yl)-4-methylbenzenesulfonamide (1q)

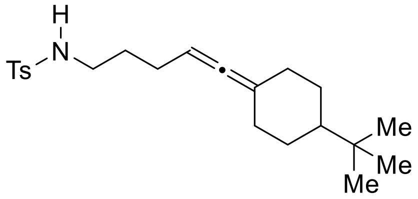

Following the described procedure, 340 mg of *t*-butyl pent-4-yn-1-yl(tosyl)carbamate **8a** was reacted with 170 mg of cyclohexanone to afford 61 mg of *N*-(5-cyclohexylidenepent-4-en-1-yl)-4-methylbenzenesulfonamide **1q** as a yellowish oil (EP/Acetone 9/1, 17%).

^{1}H NMR (600 MHz, $CDCl_3$, Me_4Si) δ: 7.73 (dm, 2H, *J* = 8.4 Hz, Ar-*H*), 7.29 (d, 2H, *J* = 8.0 Hz, Ar-*H*), 4.87 (tt, 1H, *J* = 6.4, 3.3 Hz, C=C=C-*H*), 4.51 (bt, 1H, *J* = 6.2 Hz, N-*H*), 2.95 (q, 2H, *J* = 6.4 Hz, N-CH_2-CH_2), 2.41 (s, 3H, Ar-CH_3), 2.19 (m, 2H, C=C-(C(H)*H*)$_2$), 1.97 – 1.86 (m, 2H, =C=C-(C(H)*H*)$_2$), 1.91 (q, 2H, *J* = 6.4 Hz, N-CH_2-CH_2-CH_2), 1.84 – 1.79 (m, 2H, C=C-(CH_2)$_2$-(C(H)*H*)$_2$), 1.55 – 1.50 (m, 2H, C=C-(CH_2)$_2$-(C(H)*H*)$_2$), 1.11 – 0.97 (m, 3H, C(*t*-Bu)*H*, (CH_2)$_2$-C(*t*-Bu)*H*), 0.83 (s, 9H, C-(CH_3)$_3$).

^{13}C{^{1}H} NMR (151 MHz, Chloroform-*d*) δ: 198.2 (Cq), 143.4 (Cq), 137.1 (Cq), 129.8 (2 x CH), 127.2 (2 x CH), 103.0 (Cq), 87.5 (CH), 47.8 (CH), 42.7 (CH_2), 32.6 (Cq), 31.8 (2 x CH_2), 28.8 (CH_2), 28.1 (2 x CH_2), 27.7 (3 x CH_3), 26.3 (CH_2), 21.6 (CH_3)

HRMS (ESI) *m/z*: $[M + H]^+$ Calcd for $C_{22}H_{34}NO_2S$ 376.2305, found 376.2296.

IR v max (neat)/ cm^{-1}: 3279, 2939, 2838, 1183, 1019.

S4.1.3 Synthesis of alkoxy-allene 1r

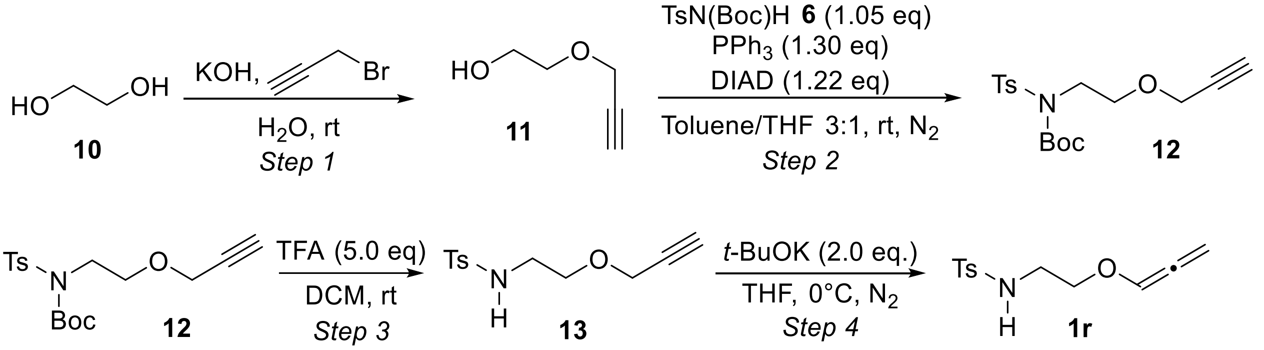

<u>*Step 1:*</u> **2-(prop-2-yn-1-yloxy)ethanol (11)**

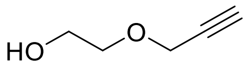

Following a procedure by Finn[8] ethylene glycol **10** (6.98 mL, 7.76 g, 125 mmol, 5.0 eq.) was reacted with propargyl bromide (2.79 mL of 80% w/w solution in $PhCH_3$, 25 mmol, 1.0 Eq.) and KOH (2.81 g, 50 mmol, 2.0 eq.) in water (4.40 mL) to obtain 2.90 g of 2-(prop-2-yn-1-yloxy)ethan-1-ol **11** (yield 58%). Spectral data are coherent with those reported in literature.[8]

^{1}H NMR (600 MHz, $CDCl_3$, Me_4Si) δ: 4.20 (d, *J* = 2.4 Hz, 2H, CH_2C≡CH), 3.77 (m, 2H, CH_2), 3.65 (m, 2H, CH_2), 2.45 (t, *j* = 2.4 Hz, 1H, C≡C*H*), 2.08 (s, 1H, O*H*).

[8] Kislukhin, A. A.; Higginson, C. J.; Hong, V. P.; Finn, M. G. *J. Am. Chem. Soc.* **2012,** *134*, 6491-6497.

Step 2: ***t*-butyl (2-(prop-2-yn-1-yloxy)ethyl)(*N*-tosyl)carbamate (12)**

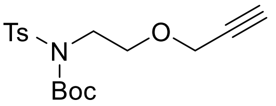

Employing our conditions for Mitsunobu reaction (see above, general procedure A, step 2) alcohol **11** (1.0 eq., 8.0 mmol, 0.80 g) was reacted with 1 *t*-butyl tosylcarbamate **6** (1.05 eq, 9.6 mmol, 2.60 g), DIAD (1.22 eq, 9.76 mmol, 1.98 g) and PPh_3 (1.30 eq., 10.4 mmol, 2.73 g); in 24 mL of a 3:1 mixture of anhydrous $PhCH_3$/THF. The crude mixture was purified by column chromatography (EP/EE 9:1) to obtain *t*-butyl (2-(prop-2-yn-1-yloxy)ethyl)(*N*-tosyl)carbamate **12** as a colorless oil.

^{1}H NMR (600 MHz, $CDCl_3$, Me_4Si) δ: 7.85 (d, *J* = 8.1 Hz, 2H, Ar-*H*), 7.28 (d, *J* = 8.1 Hz, 2H, Ar-*H*), 4.19 (d, *J* = 2.4 Hz, 2H, O-C*H*$_2$-C≡), 4.06 (t, *J* = 5.8 Hz, 2H, O-(C*H*$_2$)$_2$-N), 3.78 (t, *J* = 5.8 Hz, O-(C*H*$_2$)$_2$-N), 2.44-2.39 (m, 4H, Ar-C*H*$_3$, CH_2-C≡C*H*), 1.32 (s, 9H, (C*H*$_3$)$_3$).

^{13}C{^{1}H} NMR (150 MHz, $CDCl_3$, Me_4Si) δ: 151.0 (Cq), 144.1 (Cq), 137.6 (Cq), 129.3 (2 x CH), 128.2 (2 x CH), 84.5 (Cq), 79.5 (CH), 74.7 (CH), 68.0 (CH_2), 58.1 (CH_2), 45.4 (CH_2), 27.9 (3 x CH_3), 21.7 (CH_3).

HRMS (ESI) *m/z*: $[M + H]^+$ Calcd for $C_{17}H_{24}NO_5S$ 354.1370, found 354.1373.

IR ν max (neat)/ cm^{-1}: 2967, 2800, 1729, 1320, 1141, 1091.

Step 3: **4-methyl-*N*-(2-(prop-2-yn-1-yloxy)ethyl)benzenesulfonamide (13)**

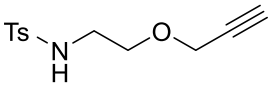

Following the general procedure A, step 3, carbamate **12** (1.0 eq., 2.5 mmol, 0.88g) was deprotected using TFA (5.0 eq, 12.5 mmol, 1.40 g) to afford 0.60 g of 4-methyl-*N*-(2-(prop-2-yn-1-yloxy)ethyl)benzenesulfonamide **13** (yield 95%) as a colorless oil that was used without further purification in the following step. Spectral data are coherent with those reported in literature.[9]

^{1}H NMR (600 MHz, $CDCl_3$, Me_4Si) δ: 7.73 (d, *J* = 8.3, Hz, 2H, Ar-*H*), 7.31 (d, *J* = 8.1, Hz, 2H, Ar-*H*), 4.85 (bs, 1H, N*H*), 4.06 (t, *J* = 2.5 Hz, 2H, O-C*H*$_2$-C≡), 3.54 (t, *J* = 5.2, Hz, 2H, O-C*H*$_2$-CH_2-N), 3.18 – 3.07 (m, 2H, m, 2H, O-CH_2-C*H*$_2$-N), 2.41 (s, 3H, Ar-C*H*$_3$).

Step 4: **4-methyl-*N*-(2-(propa-1,2-dien-1-yloxy)ethyl)benzenesulfonamide (1r)**

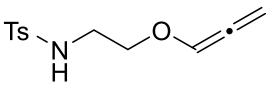

A 10 mL Schlenk tube (with screwing cap) containing a magnetic stirring bar was dried with a heat gun under vacuum then the tube was backfilled with N_2. The alkyne **13** (1.0 eq., 1 mmol, 250 mg) and 4 mL of dry THF were poured into the flask under N_2 atmosphere and the solution was cooled to -10 °C. *t*-BuOK commercial solution 1M in THF was added dropwise (2.1 eq., 2.1 mmol, 2 mL) under N_2 atmosphere with continuous stirring. The reaction was sealed and kept a -10°C for 35 minutes, then quenched with 3mL of water. The water phase was extracted with Et_2O (3x10 mL), then the organic phases were collected, dried with Na_2SO_4, filtered and the solvent evaporated. The crude yellow oil obtained (a mixture containing 60:40 ratio of **1r**/**13**) was purified by column chromatography on silica gel flash (SiO_2 deactivated with 1% w/w of Et_3N, eluent EP:EE 9:1) to obtain 125 g of **1r** (50% yield) as a colorless oil (solidification as a white solid observed when stored below -20° C).

^{1}H NMR (600 MHz, $CDCl_3$, Me_4Si) δ: 7.73 (d, *J* = 8.1 Hz, 2H, Ar-*H*), 7.30 (d, *J* = 8.1 Hz, 2H, Ar-*H*), 6.62 (t, *J* = 6.0 Hz, 1H, O-C*H*=C=), 5.40 (d, *J* = 6.0 Hz, 2H, O-CH=C=C*H*$_2$), 4,76 (m, 1H, N*H*), 3.56 (t, *J* = 5.1 Hz, 2H, C*H*$_2$-O), 3.19 (m, 2H, C*H*$_2$-NH), 2.42 (s, 3H, Ar-C*H*$_3$).

^{13}C{^{1}H} NMR (150 MHz, $CDCl_3$, Me_4Si) 200.8 (Cq), 143.8 (Cq), 137.0 (Cq), 129.9 (2 x CH), 127.2 (2 x CH), 121.2 (CH), 91.6 (CH_2), 66.8 (CH_2), 42.5 (CH_2), 21.6 (CH_3).

HRMS (ESI) *m/z*: $[M + H]^+$ Calcd for $C_{12}H_{16}NO_3S$ 254.0845, found 254.0850.

IR ν max (neat)/ cm^{-1}: 3294, 19511576, 1372, 1149, 1080, 906.

[9] Aubineau, T.; Dupas, A.; Zeng, T.; Cossy, J. *Synlett* **2021,** *32*, 525-531.

S4.1.4 General Procedure C: synthesis of allenes 1b, 1c, 1d, 1e, 1f, 1g, 1h, 1i from nitrile 17.

For allenes **1b-1i** the general synthetic route was adapted from two different previous reports[10,11] defining 4 steps:

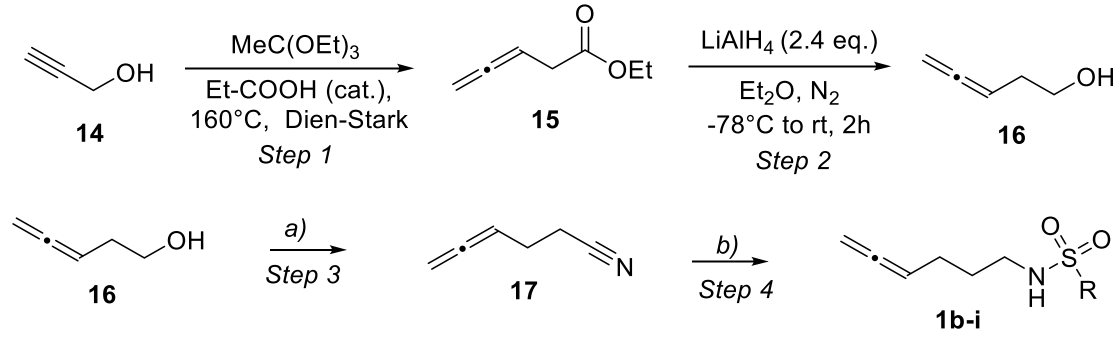

a) i) Et_3N, MsCl, N_2, Et_2O, 0°C to rt, 1h
ii) NaCN, DMSO, 50°C, 72h

b) i) $LiAH_4$ (1.5 eq), Et_2O, 0 °C to rt, 12 h
ii) RSO_2Cl **18** (1.10 eq), KOH (5.00 eq), Et_2O, rt, 24h

Step 1: **ethyl penta-3,4-dienoate (15)**

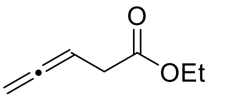

A procedure by Breit[12] from propargyl alcohol **14** was followed and the obtained product **15** was purified by distillation (bp: 46 mbar, 75 °C, yield 80%).
^{1}H NMR (600 MHz, $CDCl_3$, Me_4Si) δ: 5.26 (tt, J = 7.4, 6.7 Hz, 1H, C*H*=C=CH_2), 4.75 (dt, *J* = 6.7, 2.9 Hz, 2H, CH=C=C*H*$_2$), 4.15 (q, *J* = 7.1 Hz, 2H, O-C*H*$_2$), 3.04 (dt, *J* = 7.4, 2.9 Hz, 2H, CH-C*H*$_2$-C(O)), 1.26 (t, *J* = 7.1 Hz, 3H, CH_2-C*H*$_3$).

Step 2: **penta-3,4-dien-1-ol (16)**

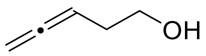

A reported procedure of reduction with $LiAlH_4$ was adapted to ethyl penta-3,4-dienoate **15** (instead of ethyl 3-(((tert-butyldiphenylsilyl)oxy)methyl)penta-3,4-dienoate)[13]. Penta-3,4-dienoate **15** (24 mmol, 1.0 eq, 3.03 g), $LiAlH_4$ (33.6 mmol, 1.4 eq, 1.30 gr) and dry Et_2O (70 mL distilled from sodium benzophenone) were used. The reaction was monitored by GC-MS. The product penta-3,4-dien-1-ol **16** was obtained in 84% yield and it was used without further purification in the next step. Spectral data are coherent with those reported in literature.[10]
^{1}H NMR (600 MHz, $CDCl_3$, Me_4Si) δ: 5.11 (q, *J* = 6.7 Hz, 1H, CH_2-C*H*=C=CH_2), 4.72 (td, *J* = 3.0, 6.7 Hz, 2H, CH=C=C*H*$_2$), 3.70 (t, *J* = 6.4 Hz, 2H, O-C*H*$_2$), 2.26 (tq, *J* = 3.0, 6.4 Hz, 2H, O-CH_2-C*H*$_2$), 1.60 (bs, 1H, O-*H*)

Step 3: **hexadi-4,5-enenitrile (17)**

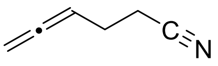

Following the procedure reported by Díez-González,[10] **16** was reacted with NaCN. The mixture was heated in a DrySyn at 50°C for 68 hours in order to obtain the total conversion of penta-3,4-dien-1-ol **16.** The product hexadi-4,5-enenitrile **17** obtained in 60% yield was used without further purification in the next step. Spectral data are coherent with those reported in literature.[10]
^{1}H NMR (600 MHz, $CDCl_3$) δ: 5.19 (quint, *J* = 6.6 Hz, 1H, CH_2-C*H*=C=CH_2), 4.84 (td, *J* = 6.6, 3.3 Hz, 2H, CH=C=C*H*$_2$), 2.45 (t, *J* = 6.7 Hz, 2H, NC-C*H*$_2$), 2.34 (m, 2H, CH_2-C*H*$_2$).

Step 4: Adapting the procedure by Breit[11], a round-bottomed flask dried under vacuum was filled with a suspension of $LiAlH_4$ (0.65 g, 17.2 mmol) in Et_2O (40 mL) under N_2 atmosphere. Then the solution was cooled at -78°C and a solution **17** (0.80 g, 8.6 mmol) in Et_2O (10 mL) was slowly added. The reaction mixture was stirred for 2 h, while temperature was allowed to reach -20°C. Monitoring of the reaction by GC-Ms confirmed its completion thus the reaction was quenched at this temperature by a dropwise addition of aqueous 5M KOH until H_2 evolution stopped and a white precipitate formed. After stirring at 0°C for 30 min, the formed precipitate

[10] Zelenay, B.; Munton, P.; Tian, X.; Díez-González, S. *Eur. J. Org. Chem.* **2019,** *2019*, 4725-4730.
[11] Berthold, D.; Geissler, A. G. A.; Giofré, S.; Breit, B. *Angew. Chem. Int. Ed.* **2019,** *58*, 9994-9997.
[12] Schmidt, J. P.; Breit, B. *Angew. Chem. Int. Ed.* **2020,** *59*, 23485-23490.
[13] Joseph E. Burchick. Jr., S. M. W., Kay M. Brummond. *Org. Synth.* **2017**, 109-122.

was filtered off and washed with Et_2O (10 mL). The aqueous layer was separated and extracted with Et_2O (3×10 mL). The combined organic phases were dried over Na_2SO_4, filtered, and concentrated under reduced pressure. The crude amine (typically 2 mmol) and 5.0 eq. KOH were suspended in Et_2O (5 mL) in a screwing cap vial to obtain a 0.4 M solution of the amine. 1.10 eq of the appropriate sulfonyl chloride R-SO_2Cl **18** was added in small portions (1 mmol every 30 minutes) at 0 °C. The suspension was continuously stirred and allowed to warm to rt overnight, then the reaction was carefully quenched with H_2O (5 mL) and the phases were separated. The aqueous phase was extracted with CH_2Cl_2 (3 × 5 mL). The organic phases were dried over Na_2SO_4 and concentrated under reduced pressure then the crude was purified on silica gel chromatography.

***N*-(penta-3,4-dien-1-yl)benzenesulfonamide (1b)**

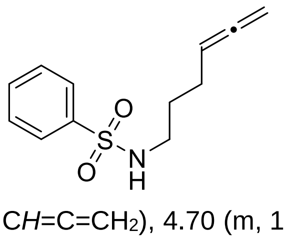

Following the described procedure, nitrile **17** (2.0 mmol, 190 mg) was reacted with benzenesulfonyl chloride **18b** (2.2 mmol, 390 mg) to obtain 260 mg of sulfonylamide **1b** as a colorless oil (Eluent: EP 95/5 Acetone. 55% yield). Spectral data match previous report.[14]

^{1}H-NMR (600 MHz, $CDCl_3$, Me_4Si) δ: 7.87 (dm, *J* = 7.6 Hz, 2H, Ar-*H*), 7.59 – 7.55 (m, 1H, *p-H*-Ar), 7.50 (tm, J = 7.6 Hz, 2H, Ar-*H*), 4.97 (quin, J = 6.6 Hz, 1H, CH_2-C*H*=C=CH_2), 4.70 (m, 1H, N-*H*), 4.58 (dt, *J* = 6.7, 3.3 Hz, 2H, CH=C=C*H*$_2$), 2.94 (q, *J* = 6.7 Hz, 2H, NH-C*H*$_2$-CH_2), 2.00 – 1.92 (m, 2H; CH_2-C*H*$_2$-CH), 1.63 – 1.55 (m, 2H, CH_2-C*H*$_2$-CH_2).

2,4,6-Trimethyl-*N*-(penta-3,4-dien-1-yl)benzenesulfonamide (1c)

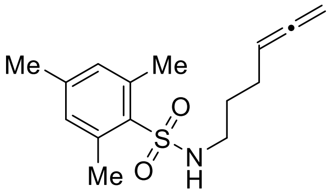

Following the described procedure, nitrile **17** (2.0 mmol, 190 mg) was reacted with 2,4,6-trimethylbenzenesulfonyl chloride **18c** (2.2 mmol, 480 mg), to obtain 346 mg of sulfonylamide **1c** as a colorless oil (Eluent: EP 92/8 Acetone, yield 62%).

^{1}H NMR (600 MHz, $CDCl_3$, Me_4Si) δ: 6.95 (s, 2H, Ar-*H*), 4.99 (q, *J* = 6.6 Hz, 1H, CH_2-C*H*=C=CH_2) 4.62 (dt, *J* = 6.7, 3.3 Hz, 2H, CH=C=C*H*$_2$), 4.47 (bt, *J* = 6.6 Hz, 1H, N-*H*) 2.93 (q, *J* = 6.8 Hz, 2H, NH-C*H*$_2$-CH_2), 2.62 (s, 6H, *o*-C*H*$_3$-*Ar*), 2.29 (s, 3H, *p*-C*H*$_3$-*Ar*), 1.96 (m, 2H; CH_2-C*H*$_2$-CH), 1.60 – 1.54 (m, 2H, CH_2-C*H*$_2$-CH_2).

^{13}C{^{1}H} NMR (151 MHz, $CDCl_3$, Me_4Si) δ: 208.8 (Cq), 142.2 (Cq), 139.2 (Cq), 133.7 (Cq), 132.1 (2 x CH), 88.8 (CH), 75.5 (CH_2), 42.0 (CH_2), 28.8 (CH_2), 25.2 (CH_2), 23.0 (2 x CH_3), 21.0 (CH_3).

HRMS (ESI) *m/z*: $[M + H]^+$ Calcd for $C_{15}H_{22}NO_2S$ 280.1366, found 280.1355.

IR ν max (neat)/ cm^{-1}: 3039, 2939, 1953, 1603, 1422, 1314, 1145, 1084, 849, 654.

4-Chloro-*N*-(penta-3,4-dien-1-yl)benzenesulfonamide (1d)

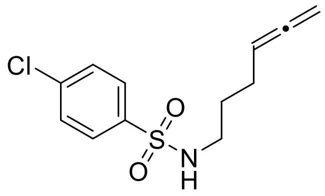

Following the described procedure, nitrile **17** (2.0 mmol, 190 mg) was reacted with 4-chlorolbenzenesulfonyl chloride **18d** (2.2 mmol, 464 mg), to obtain 245 mg of sulfonylamide **1d** as a colorless oil (Eluent: EP 92/8 Acetone, 45% yield).

^{1}H NMR (600 MHz, $CDCl_3$, Me_4Si) δ: 7.80 (dm, *J* = 7.6 Hz, 2H, Ar-*H*), 7.50 (dm, *J* = 7.6 Hz, 2H, Ar-*H*), 5.03 (quin, *J* = 6.7 Hz, 1H, CH_2-C*H*=C=CH_2), 4.66 (dt, *J* = 6.7, 3.3 Hz, 2H, CH=C=C*H*$_2$), 4.39 (t, *J* = 6.6 Hz, 1H, N-*H*), 3.02 (q, *J* = 6.8 Hz, 2H, NH-C*H*$_2$-CH_2), 2.00 (m, 2H; CH_2-C*H*$_2$-CH), 1.61 (quin, *J* = 7.1 Hz, 2H, CH_2-C*H*$_2$-CH_2).

^{13}C{^{1}H} NMR (151 MHz, $CDCl_3$, Me_4Si) δ: 208.8 (Cq), 139.2 (Cq), 138.7 (Cq), 129.5 (2 x CH), 128.6 (2 x CH), 88.7 (CH), 75.8 (CH_2), 42.7 (CH_2), 28.8 (CH_2), 25.1 (CH_2).

HRMS (ESI) *m/z*: $[M + H]^+$ Calcd for $C_{12}H_{15}ClNO_2S$ 272.0507, found 272.0504.

IR ν max (neat)/ cm^{-1}: 3265, 2949, 1952, 1414, 1323, 1161, 1084, 840, 753.

[14] Yang, C.-H.; Han, M.; Li, W.; Zhu, N.; Sun, Z.; Wang, J.; Yang, Z.; Li, Y.-M. *Org. Lett.* **2020,** *22*, 5090-5093.

4-acetyl-*N*-(hexa-4,5-dien-1-yl)benzenesulfonamide (1e)

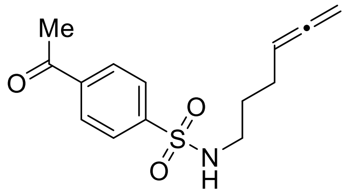

Following the described procedure, nitrile **17** (2.0 mmol, 190 mg) was reacted with 4-acetylbenzenesulfonyl chloride **18e** (2.2 mmol, 482 mg), to obtain 296 mg of sulfonylamide **1e** as a colorless oil (Eluent: EP/EE 1:1 to EP/EE 1:3, 53% yield).

^{1}H NMR (600 MHz, $CDCl_3$, Me_4Si) δ: ^{1}H NMR (600 MHz, Chloroform-*d*) δ: 8.06 (dm, 2H, *J* = 8.2 Hz, 2H, Ar-*H*), 7.95 (d, *J* = 8.3 Hz, 2H, Ar-*H*), 5.04 – 4.97 (m, 1H, CH_2-C*H*=C=CH_2), 4.62 (dt, *J* = 6.5, 3.3 Hz, 2H, CH=C=C*H*$_2$), 3.02 (q, *J* = 7.1 Hz, 2H, NH-C*H*$_2$-CH_2), 2.64 (s, 3H, C(=O)-C*H*$_3$), 1.97 (m, 2H; CH_2-C*H*$_2$-CH), 1.59 (quin, *J* = 7.1 Hz, 2H, CH_2-C*H*$_2$-CH_2).

^{13}C{^{1}H} NMR (151 MHz, $CDCl_3$, Me_4Si) δ: 208.6 (Cq), 197.0 (Cq), 144.1 (Cq), 140.1 (Cq), 129.1 (2 x CH), 127.4 (2 x CH), 88.7 (CH), 75.6 (CH_2), 42.7 (CH_2), 28.8 (CH_2), 27.0 (CH_3), 25.0 (CH_2).

HRMS (ESI) *m/z*: $[M + H]^+$ Calcd for $C_{14}H_{17}NO_3S$ 280.1002, found 280.1005.

IR ν max (neat)/ cm^{-1}: 1958, 1686, 1397, 1326, 913, 837.

4-methoxy-*N*-(hexa-4,5-dien-1-yl)benzenesulfonamide (1f)

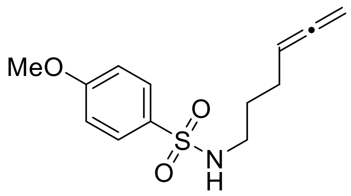

Following the described procedure, nitrile **17** (2.0 mmol, 190 mg) was reacted with 4-methoxybenzenesulfonyl chloride **18f** (2.2 mmol, 455 mg), to obtain 320 mg of sulfonylamide **1f** as a colorless oil (Eluent: Eluent: EP/EE 1:1, 55% yield). Spectral data match previous report.[11]

^{1}H NMR (600 MHz, $CDCl_3$, Me_4Si) δ: 7.79 (dm, 2H, *J* = 8.2 Hz, 2H, Ar-*H*), 6.97 (d, *J* = 8.3 Hz, 2H, Ar-*H*), 5.01 (p, *J* = 6.7 Hz, 1H, CH_2-C*H*=C=CH_2), 4.63 (dt, *J* = 6.6, 3.3 Hz, 2H, CH=C=C*H*$_2$), 4.33 (bt, *J* = 6.3 Hz, 1H, N*H*), 3.86 (s, 3H, OC*H*$_3$), 2.97 (q, *J* = 6.8 Hz, 2H NH-C*H*$_2$-CH_2), 1.97 (m, 2H; CH_2-C*H*$_2$-CH), 1.59 (quin, *J* = 7.1 Hz, 2H, CH_2-C*H*$_2$-CH_2).

***N*-(hexa-4,5-dien-1-yl)-2,3-dihydrobenzo[b][1,4]dioxine-6-sulfonamide (1g)**

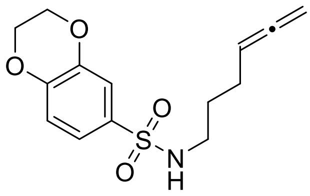

Following the described procedure, nitrile **17** (2.0 mmol, 190 mg) was reacted with 1,4-Benzodioxan-6-sulfonyl chloride **18g** (2.2 mmol, 516 mg), to obtain 167 mg of sulfonylamide **1g** as a colorless oil (Eluent: EP 70/30 EtOAc, 30% yield).

^{1}H NMR (600 MHz, $CDCl_3$, Me_4Si) δ: 7.38 (d, *J* = 2.2 Hz, 1H, Ar-*H*), 7.34 (dd, *J* = 8.5, 2.2 Hz, 1H, Ar-*H*), 6.95 (d, *J* = 8.5 Hz, 1H, Ar-*H*), 5.03 (quin, *J* = 6.5 Hz, 1H, CH_2-C*H*=C=CH_2), 4.66 (dt, *J* = 6.7, 3.3 Hz, 2H, CH=C=C*H*$_2$), 4.31 (m, 5H, N-*H*, O-(C*H*$_2$)$_2$-O), 2.98 (q, *J* = 6.3 Hz, 2H, NH-C*H*$_2$-CH_2), 2.00 (m, 2H; CH_2-C*H*$_2$-CH), 1.60 (m, 2H, CH_2-C*H*$_2$-CH_2).

^{13}C{^{1}H} NMR (151 MHz, $CDCl_3$, Me_4Si) δ: 208.5 (Cq), 147.3 (Cq), 147.5 (Cq), 132.4 (Cq), 120.7 (CH), 117.7 (CH), 116.7 (CH), 88.7 (CH), 75.4 (CH_2), 64.5 (CH_2), 64.2 (CH_2), 42.6 (CH_2), 28.7 (CH_2), 25.0 (CH_2).

HRMS (ESI) *m/z*: $[M + H]^+$ Calcd for $C_{14}H_{18}ClNO_4S$ 296.0951, found 296.0948.

IR ν max (neat)/ cm–1: 3277, 1952, 1583, 1320, 1151, 816, 698.

4-acetyl-*N*-(hexa-4,5-dien-1-yl)benzenesulfonamide (1h)

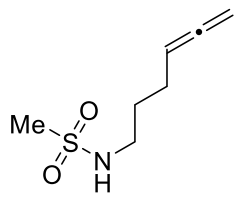

Following the described procedure, nitrile **17** (2.0 mmol, 190 mg) was reacted with mesylchloride **18h** (2.2 mmol, 482 mg), to obtain 296 mg of sulfonylamide **1h** as a colorless oil (Eluent: EP/EE 1:1 to EP/EE 1:3, 53% yield).

^{1}H NMR (600 MHz, $CDCl_3$, Me_4Si) δ: 5.09 (quint, *J* = 6.6 Hz, 1H, CH_2-C*H*=C=CH_2), 4.70 (dt, *J* = 6.6, 3.3 Hz, 2H, CH=C=C*H*$_2$), 4.20 (s, 1H, N*H*), 3.18 (q, *J* = 6.9 Hz, 2H, NH-C*H*$_2$-CH_2), 2.95 (s, 3H, C*H*$_3$$SO_2$), 2.08 (m, 2H; CH_2-C*H*$_2$-CH), 1.70 (quin, *J* = 7.2 Hz, 2H, CH_2-C*H*$_2$-CH_2).

^{13}C{^{1}H} NMR (151 MHz, $CDCl_3$, Me_4Si) δ: 208.7 (Cq), 88.8 (CH), 75.7 (CH_2), 42.7 (CH_2), 40.5 (CH_3), 29.3 (CH_2), 25.10 (CH_2).

HRMS (ESI) *m/z*: $[M + H]^+$ Calcd for $C_7H_{14}NO_2S$ 176.0740, found 176.0743.

IR ν max (neat)/ cm^{-1}: 3282, 2931, 1953, 1410, 1310, 1143, 845.

1-((1*S*,4*S*)-7,7-dimethyl-2-oxobicyclo[2.2.1]heptan-1-yl)-*N*-(hexa-4,5-dien-1-yl)methanesulfonamide (1i)

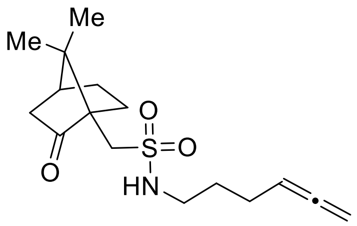

Following the described procedure, nitrile **17**(2.0 mmol, 190 mg) was reacted with (1*S*)-(+)-10-Camphorsulfonyl chloride **18i** (2.2 mmol, 550 mg), to obtain 181 mg of sulfonylamide **1i** as a colorless oil (Eluent: EP 92/8 Acetone, 29% yield).

^{1}H NMR (600 MHz, $CDCl_3$, Me_4Si) δ: 5.15 (bt, *J* = 6.6 Hz, 1H, N*H*), 5.10 (quint, *J* = 6.7 Hz, 1H,, CH_2-C*H*=C=CH_2), 4.69 (dt, *J* = 6.6, 3.3 Hz, 2H, CH=C=C*H*$_2$), 3.38 (d, *J* = 15.1 Hz, 1H, SO_2-C(H)*H*), 3.27 – 3.14 (m, 2H, N-C*H*$_2$-CH_2-C*H*$_2$), 2.90 (d, *J* = 15.1 Hz, 1H, SO_2-C(H)*H*), 2.39 (ddd, *J* = 18.6, 4.9, 3.2 Hz, 1H, C(=O)-C(H)*H*), 2.26 – 2.17 (m, 1H, CH_2-C(C)*H*-CH_2), 2.12 – 2.07 (m, 1H, C-(C(*H*)H)$_2$-CH-C(=O)), 2.12 (t, *J* = 4.5 Hz, 2H, N-CH_2-CH_2-C*H*$_2$), 2.06-1.99 (m, 1H, C-(C(*H*)H)$_2$-CH-C(=O)), 1.96-1.89 (dm, *J* =18.6 Hz, 1H, C(=O)-C(H)*H*), 1.99 – 1.93 (m, 1H, C-(C(*H*)H)$_2$-CH-C(=O)), 1.72 (q, *J* =7.2 Hz, 2H, N-CH_2-C*H*$_2$-CH_2), 1.48 – 1.40 (m, 1H, C-(C(*H*)H)$_2$-CH-C(=O)), 1.01 (s, 3H, C(CH_3)C*H*$_3$), 0.91 (s, 3H, C(CH_3)C*H*$_3$).

^{13}C{^{1}H} NMR (151 MHz, $CDCl_3$, Me_4Si) δ: 217.2 (Cq), 208.7 (Cq), 89.0 (CH), 75.5 (CH_2), 59.4 (Cq), 49.5 (CH_2) 48.9 (Cq), 43.2 (CH_2), 43.1 (CH_2), 42.9 (CH), 27.1 (CH_2), 26.9 (CH_2), 25.2 (CH_2), 20.0 (CH_3), 19.6 (2 x CH_3).

HRMS (ESI) *m/z*: [M + H]$^+$ Calcd for C $C_{16}H_{26}NO_3S$ 312.1628, found 312.1625.

IR ν max (neat)/ cm^{-1}: 3276, 2952, 1736, 1200, 1026.

S4.1.5 Synthesis of alkoxy allenes 1s, 1t.

4-methyl-*N*-(2-(prop-2-yn-1-yloxy)phenyl)benzenesulfonamide **19** and *N*-(2-(prop-2-yn-1-yloxy)phenyl)methanesulfonamide **20** were synthetized from commercially available *ortho*-aminophenol in three steps according to a procedure reported by Karpoormath, Bera[15] and co-workers. Isomerization to the corresponding alkoxy allenes **1s**, **1t** was conducted with *t*-BuOK according to an adapted procedure.[16]

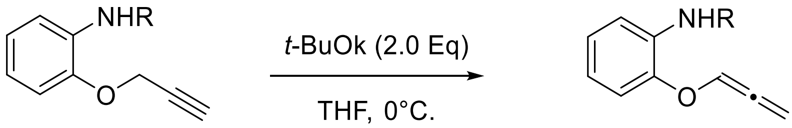

19, R= Ts
20, R= Ms

1s, R= Ts
1t, R= Ms

In a Schlenk bottle under N_2, a solution of sulfonamide **19** or **20** (3.0 mmol) in 10 ml anhydrous THF was chilled in an ice-water bath and treated with *t*-BuOK (2.0 eq., 6.0 mmol, 6.0 ml 1M in THF). The solution was stirred 25 minutes at room temperature before being quenched with 10 ml of crushed ice. The mixture was extracted with ethyl acetate (3x25 ml). The collected organic phases were washed with brine (1x50 ml) and dried with Na_2SO_4. The crude mixture was purified with silica gel chromatography (EP/AcOEt 5:1, Et_3N 1%).

4-methyl-*N*-(2-(propa-1,2-dien-1-yloxy)phenyl)benzenesulfonamide (1s)

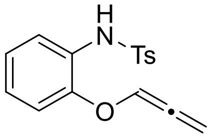

Following the reported procedure, 0.910 gr (3.0 mmol) of 4-methyl-*N*-(2-(prop-2-yn-1-yloxy)phenyl)benzenesulfonamide **19** were reacted to afford 0.65 g of 2-(1-chlorovinyl)-3-tosyl-2,3-dihydrobenzo[d]oxazole **21** as a pale yellow solid (70% yield).

^{1}H NMR (600 MHz, $CDCl_3$, Me_4Si) δ: 7.69 – 7.62 (m, 2H, Ar-*H*), 7.60 – 7.54 (m, 1H, Ar-*H*), 7.22 – 7.15 (m, 2H, Ar-*H*), 7.04– 6.99 (m, 2H, Ar-*H*), 6.98 (bs, 1H, N*H*), 6.95 – 6.90 (m, 1H, Ar-*H*), 6.51 (t, *J* = 5.9 Hz, 1H, OC*H*=CH_2), 5.38 (d, *J* = 5.9 Hz, 2H, OCH=C*H*$_2$), 2.36 (s, 3H, C*H*$_3$).

^{13}C{^{1}H} NMR (151 MHz, $CDCl_3$, Me_4Si) δ: 202.2 (Cq), 147.2 (Cq), 143.9 (Cq), 136.2 (Cq), 129.6 (2 x CH), 127.4 (2 x CH), 127.3 (Cq), 125.3 (CH), 123.7 (CH), 121.7 (CH), 117.9 (CH), 116.0 (CH), 90.7 (CH_2), 21.7 (CH_3).

HRMS (ESI) *m/z*: [M + H]$^+$ Calcd for $C_{16}H_{16}NO_3S$ 302.0845, found 302.0837.

IR ν max (neat)/ cm^{-1}: 3242, 1966, 1596, 1495, 1149.

m.p.: 72.8 –74.6 °C.

[15] Karunanidhi, S.; Karpoormath, R.; Bera, M.; Rane, R. A.; Palkar, M. B. *J. Heterocyclic Chem.* **2016,** *53*, 1611-1616.

N-(2-(propa-1,2-dien-1-yloxy)phenyl)methanesulfonamide (1t)

Following the reported procedure, 0.680 gr (3.0 mmol) of *N*-(2-(prop-2-yn-1-yloxy)phenyl)methanesulfonamide **20** were reacted to afford 0.428 g of *N*-(2-(propa-1,2-dien-1-yloxy)phenyl)methanesulfonamide **1t** as a pale yellow solid (63% yield). Spectral data match previous report.[16]

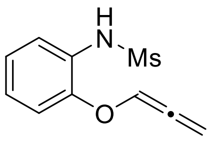

^{1}H NMR (600 MHz, $CDCl_3$, Me_4Si) δ*:* 7.59–7.55 (m, 1H, Ar-*H*), 7.19–7.15 (m, 1H, Ar-*H*), 7.14-7.05 (m, 2H, Ar-*H*), 6.85 (bt, *J* = 5.9 Hz, 1H, N*H*), 6.78 (s, 1H, OC*H*=CH_2), 5.49 (d, *J* = 5.9 Hz, 2H, OCH=C*H_2*), 2.99 (s, 3H, CH_3SO_2).

S4.1.6 Synthesis of phenylthioallene 1u.

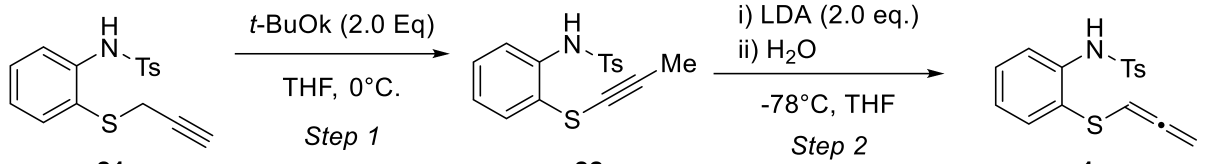

21 **22** **1u**

<u>Step 1:</u> **4-methyl-*N*-(2-(prop-1-yn-1-ylthio)phenyl)benzenesulfonamide (22)**

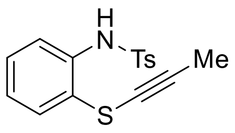

In a Schlenk bottle under N_2, a solution of sulfonamide **21** (3.0 mmol) in 10 ml anhydrous THF was chilled in an ice-water bath and treated with *t*-BuOK (2.0 eq., 6.0 mmol, 6.0 ml 1M in THF). The solution was stirred 15 minutes at room temperature before being quenched with 10 ml of crushed ice. The mixture was extracted with ethyl acetate (3x25 ml). The collected organic phases were washed with brine (1x50 ml) and dried with Na_2SO_4. The crude yellow solid obtained resulted to be product **22**, obtained in 91% yield and used without further purification in the next step. The sulfonamide **21** was prepared according to the procedure reported by Kundu *et al*. [17]

^{1}H NMR (600 MHz, $CDCl_3$, Me_4Si) δ: 7.60 (m, *J* = 7.0 Hz, 2H, Ar*H*), 7.45-7.39 (m, 1H, Ar-*H*), 7.31-7.13 (m, 2H, Ar-*H*, N*H*), 7.05-6.78 (m, 4H, Ar-*H*), 2.24 (s, 3H, Ar-C*H_3*), 2.01 (s, 3H, C≡C-C*H_3*).

^{13}C{^{1}H} NMR (151 MHz, $CDCl_3$, Me_4Si) δ: 141.8 (Cq), 138.6 (Cq), 132.7 (Cq), 129.3 (2 x CH), 129.2 (Cq), 127.2 (2 x CH), 127.1 (CH), 126.1 (CH), 123.7 (CH), 121.7 (CH), 93.8 (Cq), 65.2 (Cq), 21.5 (CH_3), 5.3 (CH_3).

HRMS (ESI) *m/z*: $[M + H]^+$ Calcd for $C_{16}H_{16}NO_2S_2$ 318.0617, found 318.0610.

IR v max (neat)/ cm^{-1}: 3250, 2913, 1578, 1464, 1280, 1118, 1085 cm^{-1}

mp: degradation 150°.

<u>Step 2</u>**: 4-methyl-*N*-(2-(propa-1,2-dien-1-ylthio)phenyl)benzenesulfonamide (1u)**

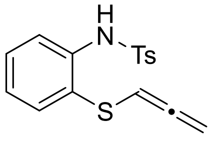

A procedure by Pearson was adapted to the synthesis of a phenylthioallene.[18] In a Schlenk bottle under N_2 a solution of diisopropylamine (2.2 eq., 0.440 mL, 320 mg, 3.30 mmol) in THF (15 mL) was added and cooled to -50 °C. Then *n*-BuLi (2.1 eq., 1.2 mL of the 2.5 M solution in hexanes, 3.15 mmol) was added dropwise over 1.5 min under stirring. After 10 min, the solution was cooled to -75 °C, and a solution of the crude phenylthiopropyne **22** (480 mg, 1.5 mmol) in THF (10 mL) was added dropwise in a 30 second period under continuous stirring. After 8 min, H_2O (2 mL) was added within a 10 s period *via* syringe with the stainless needle immersed in the reaction solution, quenching the reaction. After 1 minute, the reaction mixture was removed from the cooled bath and diluted with AcOEt (10 mL) and water (10 mL). The solution was allowed to warm to room temperature. The aqueous phase was extracted with AcOEt (3x10 mL), the organic phase was gathered, washed with brine (1 X 15 mL), dried (Na_2SO_4), and concentrated in vacuo. The resulting crude was purified with flash silica gel chromatography (SiO_2 treated with 1% w/w of Et_3N, eluent EP/Acetone 5:1) to give 228 mg of **1u** as a colorless oil (yield 48%).

---

[16] Choi, J.; Kim, G. *Tetrahedron Lett.* **2017,** *58*, 4436-4439.
[17] Kundu, N. G.; Nandi, B. *Tetrahedron* **2001,** *57*, 5885-5895.
[18] Pearson, W. H.; Lin, K. C.; Poon, Y. F. *J. Org. Chem.* **1989,** *54*, 5814-5819.

^{1}H NMR (600 MHz, $CDCl_3$, Me_4Si) δ: 7.71 – 7.67 (m, 2H, Ar-*H*), 7.64 (dd, *J* = 8.2, 1.3 Hz, 1H, Ar-*H*), 7.56 (s, 1H, N*H*), 7.41 (dd, *J* = 7.7, 1.6 Hz, 1H, Ar-*H*), 7.32 – 7.26 (m, 1H, Ar-*H*), 7.23 – 7.19 (m, 2H, Ar-*H*), δ: 7.03 (td, *J* = 7.7, 1.3 Hz, 1H, Ar-*H*), 5.49 (t, *J* = 6.3 Hz, 1H, S-C*H*=CH_2), 4.81 (d, *J* = 6.3 Hz, 2H, S-CH=C*H*$_2$), 2.36 (s, 3H, Ar-C*H*$_3$).
^{13}C{^{1}H} NMR (151 MHz, $CDCl_3$, Me_4Si) δ: 207.0 (Cq), 144.1 (Cq), 138.6 (Cq), 136.1 (Cq), 135.8 (CH), 130.5 (CH), 129.7 (2 x CH), 127.4 (2 x CH), 124.9 (CH), 122.7 (CH), 120.2 (CH), 87.0 (CH), 80.8 (CH_2), 21.6 (CH_3).
HRMS (ESI) *m/z*: $[M + H]^+$ Calcd for $C_{16}H_{16}NO_2S_2$ 318.0617, found 318.0613.
IR ν max (neat)/ cm^{-1}: 3269, 1586, 1475, 1388, 1334, 1158, 1089, 915.

S4.1.7 Synthesis of allenes for mechanistic studies: 3a and 1a$^-$Na$^+$

***N*-chloro-*N*-(hexa-4,5-dien-1-yl)-tosylamide (3a)**

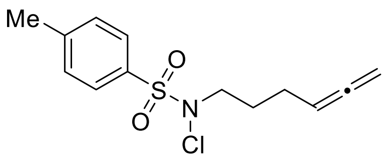

According to a modified procedure,[19] in a 100 mL round-bottomed flask, allene **1a** (1.0 eq., 400 mg, 1.6 mmol) was dissolved in 30 mL of $CHCl_3$, then 30 mL of aqueous NaClO (11-15% available chlorine) was added and vigorously stirred until reaction completion (carefully monitored every 30 minutes by TLC in eluent Hexanes/AcOEt 3:1. The reaction mixture was then stopped, the aqueous phase removed and washed with $CHCl_3$ (3x30 mL) then the organic phase was collected, dried over Na_2SO_4, filtered and solvent evaporated under reduced pressure to obtain 458 mg of a colorless oil that resulted to be the pure product **3a** (98%), used without further purification, and stored under N_2 at -20°C (as a white solid).
^{1}H NMR (600 MHz, $CDCl_3$, Me_4Si) δ: 7.81 (dm, *J* = 7.9 Hz, 2H, Ar-*H*), 7.38 (dm, *J* = 7.9 Hz, 2H, Ar-*H*), 5.10 (quin, *J* = 6.9, 6.9 Hz, 1H, CH_2-C*H*=C=CH_2), 4.69 (m, 2H, CH=C=C*H*$_2$), 3.27 (br t, *J* = 6.9 Hz, 2H, N-C*H*$_2$), 2.46 (s, 3H, Ar-C*H*$_3$), 2.08 (m, 2H, C*H*$_2$-CH=C), 1.80 (quin, *J* = 6.9 Hz, 2H, CH_2-C*H*$_2$-CH_2).
^{13}C{^{1}H} NMR (151 MHz, $CDCl_3$, Me_4Si) δ: 208.7 (Cq), 145.5 (Cq), 130.0 (Cq), 129.8 (2xCH), 129.7 (2xCH), 88.8 (CH), 75.7 (CH_2), 56.0 (CH_2), 26.3 (CH_2), 24.6 (CH_2), 21.8 (CH_3).
HRMS (ESI) *m/z*: $[M + H]^+$ Calcd for $C_{13}H_{17}NO_2S$ 286.0663, found 286.0663.
IR ν max (neat)/ cm^{-1}: 2919, 2852, 1954, 1595, 1359, 1167, 1088, 666.

Sodium hexa-4,5-dien-1-yl(tosyl)amide (1a$^-$Na$^+$)

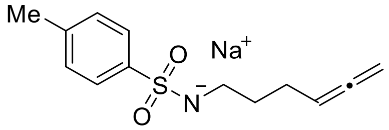

A 10 mL Schlenk tube (with screwing cap) containing a magnetic stirring bar was dried with a heat gun under vacuum then the tube was backfilled with N_2. NaH in mineral oil suspension (1.0 eq., 24 mg (40 mg of the suspension), 1.0 mmol), was added under N_2 to the flask and subjected to two vacuum/N_2 cycles. Anhydrous THF (3 mL/mmol of NaH) was added *via* syringe under N_2 atmosphere, then the allene **1a** (1.1 eq, 1.1 mmol, 275 mg) was added dropwise at RT to this mixture. The solution was stirred for 2h, then upon completion verified by TLC, hexanes were added (5 ml/mmol of NaH) and a white precipitate formed. The precipitate was filtered and washed with hexanes and cold THF (1mL/mmol of NaH) to remove the unreacted amide **1a**. 205 mg of white solid were recovered and resulted to be the desired sodium salt **1a$^-$Na$^+$**.
^{1}H NMR (600 MHz, **$CDCl_3$**, Me_4Si) δ: 7.57 (d, *J* = 7.9 Hz, 2H, Ar-*H*), 6.99 (d, *J* = 7.9 Hz, 2H, Ar-*H*), 4.87 (quin, *J* = 6.7 Hz, 1H, CH_2=C=C*H*), 4.51 (dt, *J* = 6.7, 3.2 Hz, 2H, CH=C=C*H*$_2$), 2.64 (t, *J* = 6.9 Hz, 2H, N-C*H*$_2$), 2.31 (s, 3H, Ar-C*H*$_3$), 1.79 (m, 2H, C*H*$_2$-CH=C), 1.36 (quin, *J* = 6.9 Hz, 2H, CH_2-C*H*$_2$-CH_2).
^{1}H NMR (600 MHz, **DMSO-d_6**, Me_4Si) δ: 7.44 (d, *J* = 7.9 Hz, 2H, Ar-*H*), 7.08 (d, *J* = 7.9 Hz, 2H, Ar-*H*), 5.09 (quin, *J* = 6.7, 1H, CH_2=C=C*H*), 4.65 (dt, *J* = 6.6, 3.3 Hz, 2H, CH=C=C*H*$_2$), 2.57 (t, *J* = 6.9 Hz, 2H, N-C*H*$_2$), 2.27 (s, 3H, Ar-C*H*$_3$), 1.89 (m, 2H, C*H*$_2$-CH=C), 1.33 (quin, *J* = 6.9, 2H, CH_2-C*H*$_2$-CH_2).
^{13}C{^{1}H} NMR (151 MHz, DMSO-d_6, Me_4Si) δ: 208.3 (Cq), 141.5 (Cq), 129.7 (2 x CH), 127.0 (2 x CH), 90.0 (CH), 79.7 (Cq) 75.9 (CH_2), 43.5 (CH_2), 30.1 (CH_2), 25.5 (CH_2), 21.4 (CH_3).
HRMS (ESI) *m/z* $[M]^-$ Calcd for $C_{13}H_{16}NO_2S$ 250.0896, found 250.0897.

---

[19] Dzandzi, J. P. K.; Beckford Vera, D. R.; Genady, A. R.; Albu, S. A.; Eltringham-Smith, L. J.; Capretta, A.; Sheffield, W. P.; Valliant, J. F. *J. Org. Chem.* **2015,** *80*, 7117-7125.

IR ν max (neat)/ cm^{-1}: 1953, 1651, 1372, 1156, 1093, 863, 813.   **Mp**: degradation 150°C

S4.2 ^{1}H-NMR Characterization of product 4a and 4b

Product **4a** and **4b** were obtained studying the influence of the halogenating agent, see paragraph S2.4. At the end of the experiments, the crude obtained was purified by silica gel chromatography (EP/Acetone 9:1).

2-(1-Bromovinyl)-1-tosylpyrrolidine (4a)

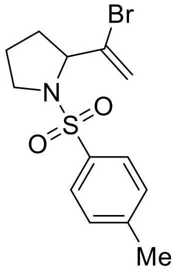

42 mg, colorless solid, 58% yield. Spectral data match previous report.[6]

^{1}H NMR (600 MHz, $CDCl_3$, Me_4Si): δ 7.72 (d, *J* = 8.3 Hz, 2H, Ar-*H*), 7.31 (d, *J* = 8.3 Hz, 1H, Ar-*H*), 5.99 (dd, *J* = 2.0, 1.2 Hz, CBr=CH_aH_b), 5.56 (dd, *J* = 2.0, 0.7 Hz, 1H, CBr=CH_aH_b), 4.35 (dd, 1H, *J* = 8.3, 3.5 Hz, N-C*H*-CBr), 3.49 (m, 1H, N-CH_aH_b-CH_2), 3.28 (m, 1H, N-CH_aH_b-CH_2), 2.42 (s, 3H, CH_3), 1.99 (m, 2H, N-CH_2-$(CH_2)_2$), 1.72 (m, 1H, N-CH_2-$(CH_2)_2$), 1.64 (m, 1H, N-CH_2-$(CH_2)_2$).

2-(1-Iodovinyl)-1-tosylpyrrolidine (4b)

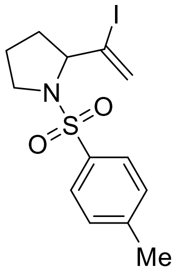

50 mg colorless solid, 58% yield. Spectral data match previous report.[20]

^{1}H NMR (600 MHz, $CDCl_3$, Me_4Si): δ 7.72 (d, *J* = 8.3 Hz, 2H, Ar-*H*), 7.31 (d, *J* = 8.3 Hz, 1H, Ar-*H*), 6.43 (dd, *J* = 1.9, 1.3 Hz, CI=CH_aH_b), 5.83 (dd, *J* = 2.0, 0.7 Hz, 1H, CI=CH_aH_b), 4.16 (dd, 1H, *J* = 8.3, 3.8 Hz, N-C*H*-CBr), 3.48 (m, 1H, N-CH_aH_b-CH_2), 3.31 (m, 1H, N-CH_aH_b-CH_2), 2.42 (s, 3H, CH_3), 1.89 (m, 2H, N-CH_2-$(CH_2)_2$), 1.74 (m, 1H, N-CH_2-$(CH_2)_2$), 1.62 (m, 1H, N-CH_2-$(CH_2)_2$).

[20] Shaw, R. W.; Gallagher, T. *J. Chem. Soc., Perkin Trans. 1* **1994**, 3549-3555.

S5 NMR characterization of new compounds

^{1}H NMR *spectra* were recorded at NMR Jeol ECZR 600 MHz. $^{13}C\{^{1}H\}$ NMR spectra at 150 MHz, in $CDCl_3$ or DMSO-d^{6}-. ^{13}C NMR *spectra* were measured with complete proton decoupling (notation $^{13}C\{1H\}$). DEPT experiments were carried out with a DEPT-135 sequence.

For each new compound, 4 spectra are reported ^{1}H-NMR, 2d-COSY, $^{13}C\{^{1}H\}$ NMR, DEPT-135.

Data were reported as follows: chemical shifts in ppm from Me_4Si as an internal standard, multiplicity, coupling constants (Hz), integration and assignment. Chemical shifts were reported in ppm from the residual solvent peak as an internal standard. Consequently, *spectra* are referenced to the residual solvent peak.

Annotations in spectra:

- Unknow impurities are indicated
- Residual "grease" impurities are indicated highlining the corresponding peak in grey.
- The signal of residual $CHCl_3$ (77.3 ppm) is notated in DEPT-135 *spectra* to differentiate it from the diagnostic signals related to the compounds **1c-u** which typically possess a signal at 75-76 ppm.
- In the case of isomer mixtures:
  When a mixture enriched on one major isomer was found:
  * annotations indicate a peak of the major isomer;
  @ annotations indicate a peak of the minor isomer;
  When peaks could not be clearly assigned to a specific isomer in a mixture, no annotations on *spectra* were given.

^{1}H-NMR (600 MHz, $CDCl_3$)

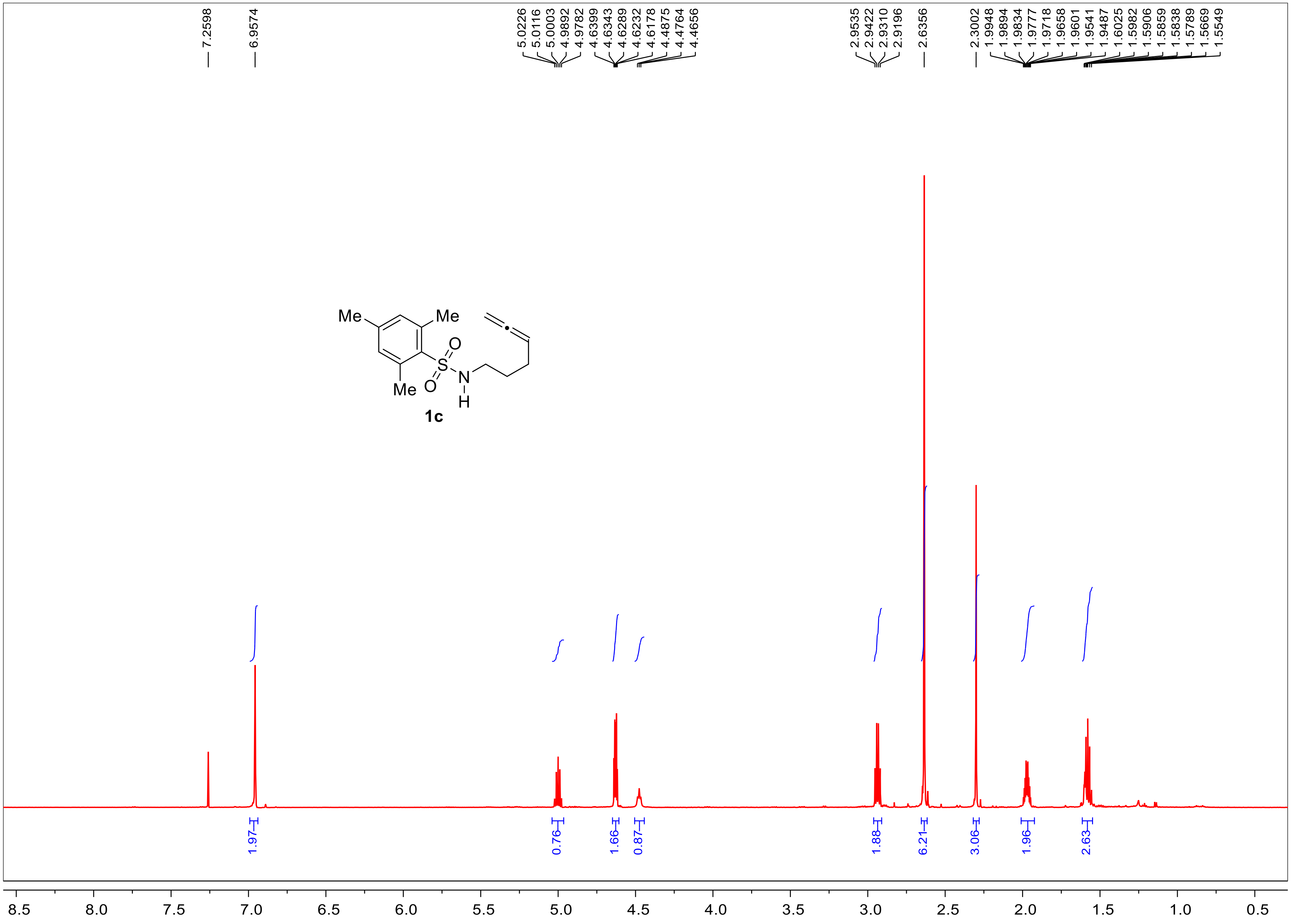

2D - COSY (600 MHz, $CDCl_3$)

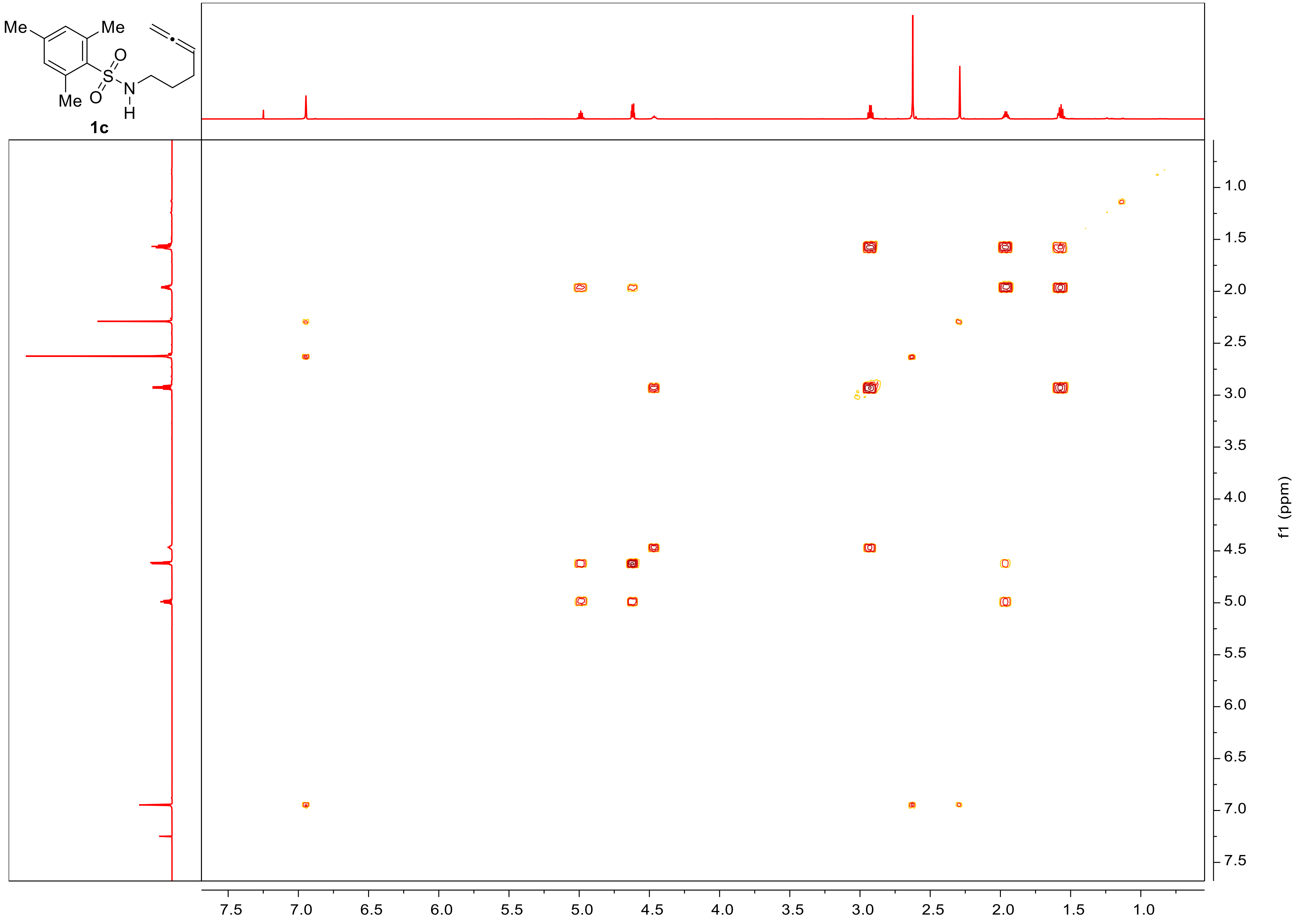

$^{13}C\{^{1}H\}$ NMR (150 MHz, $CDCl_3$)

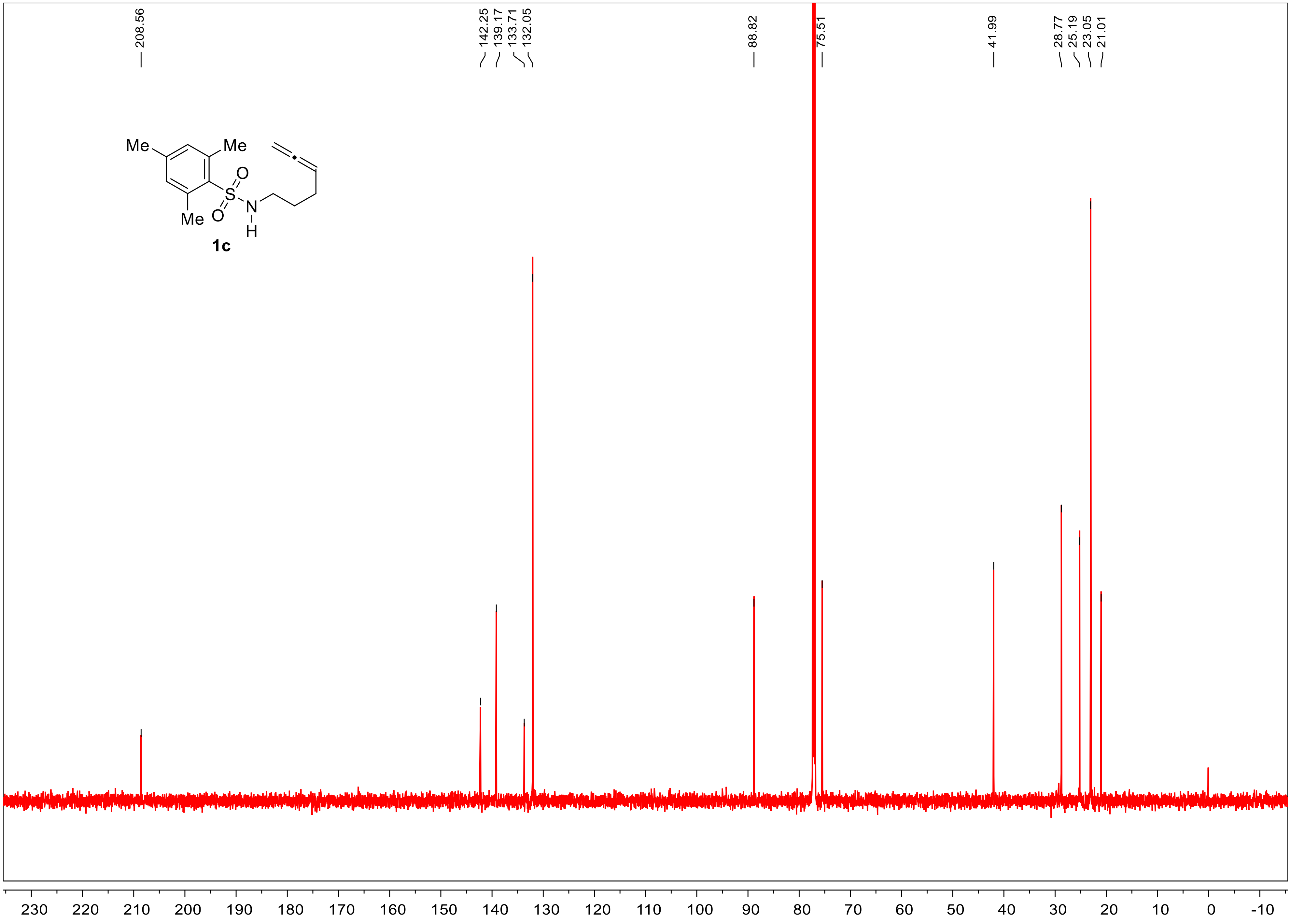

DEPT 135 (150 MHz, $CDCl_3$)

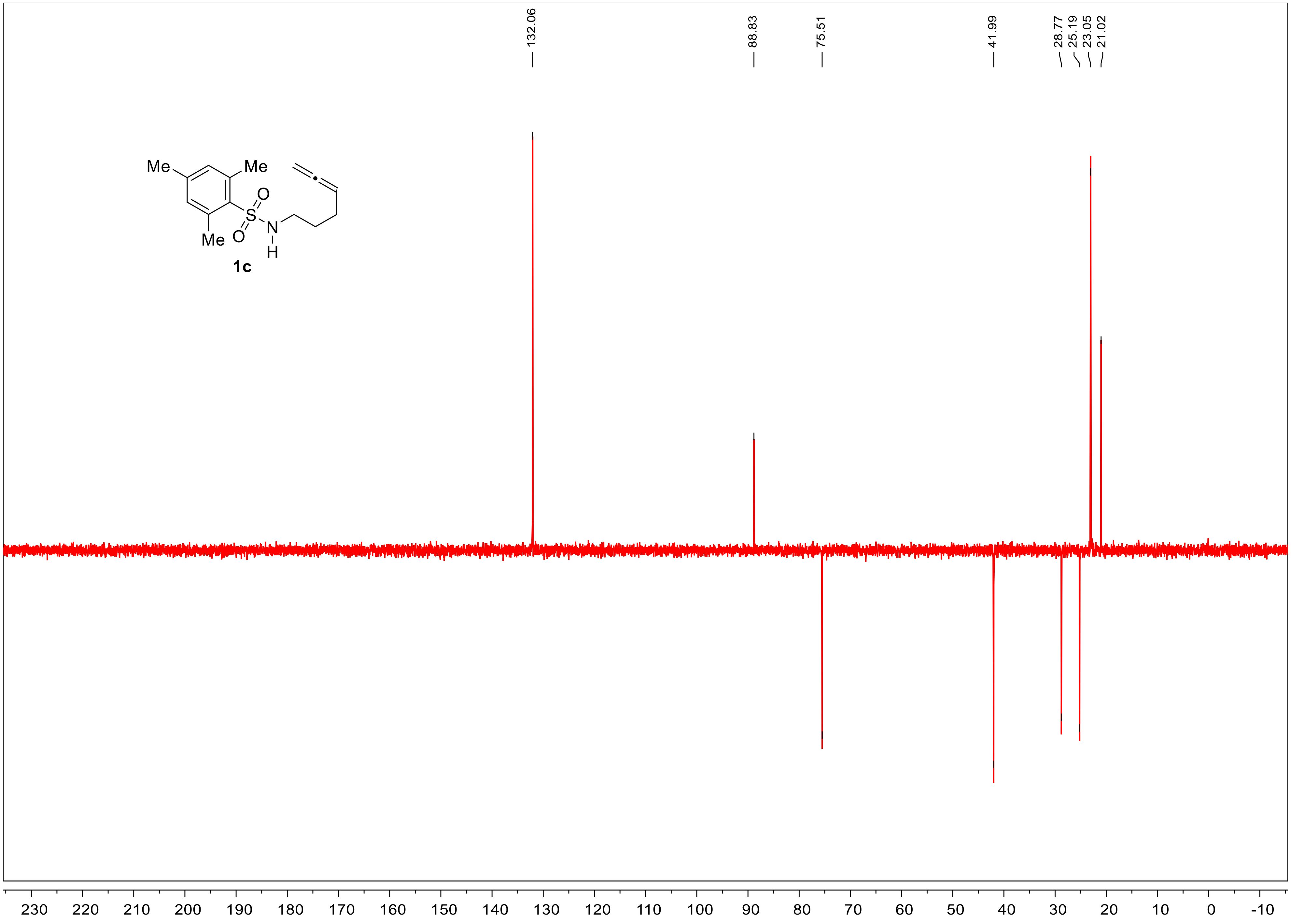

^{1}H-NMR (600 MHz, $CDCl_3$)

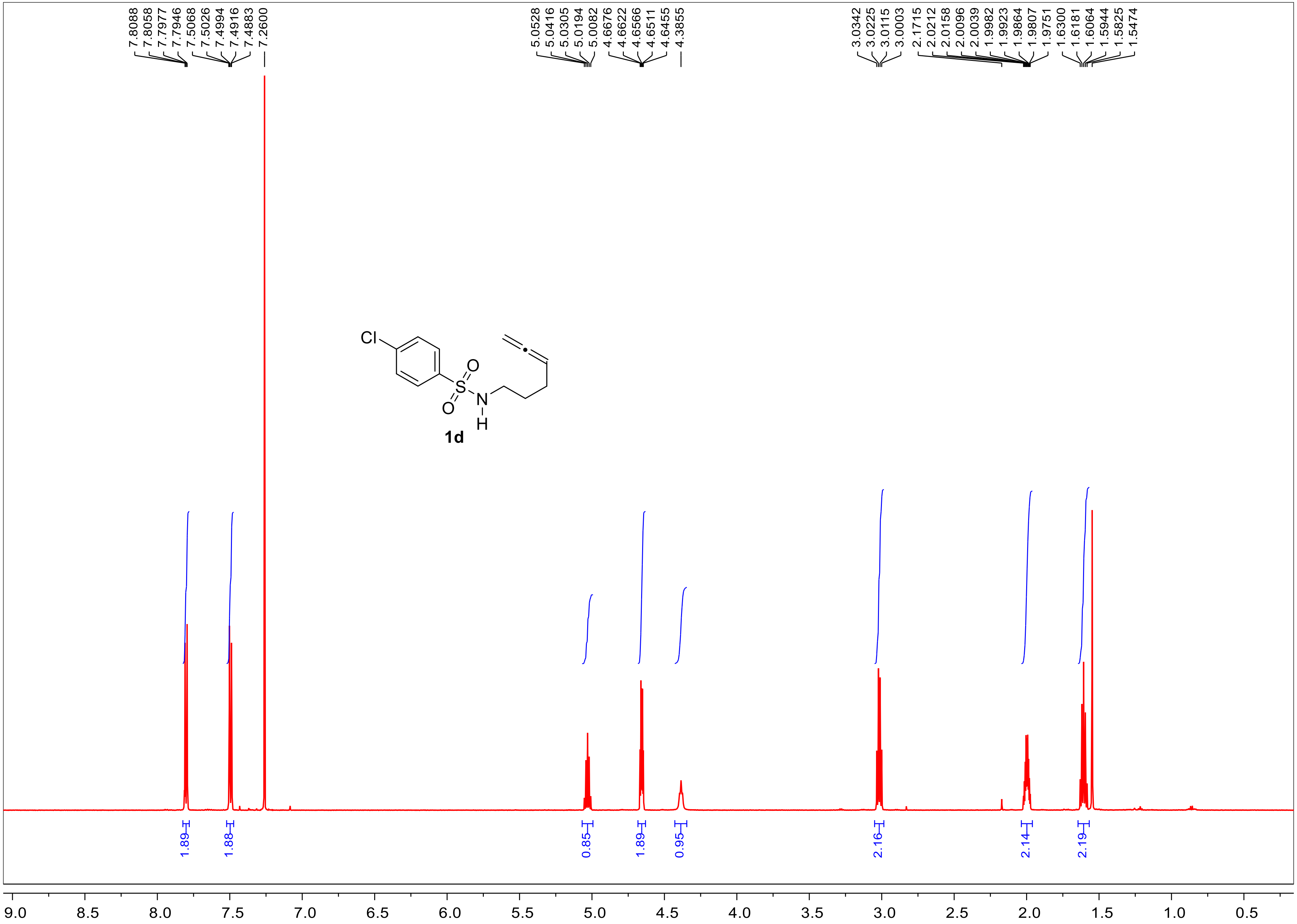

2D - COSY (600 MHz, $CDCl_3$)

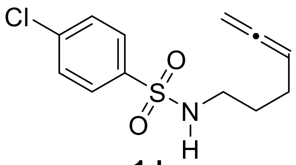

1d

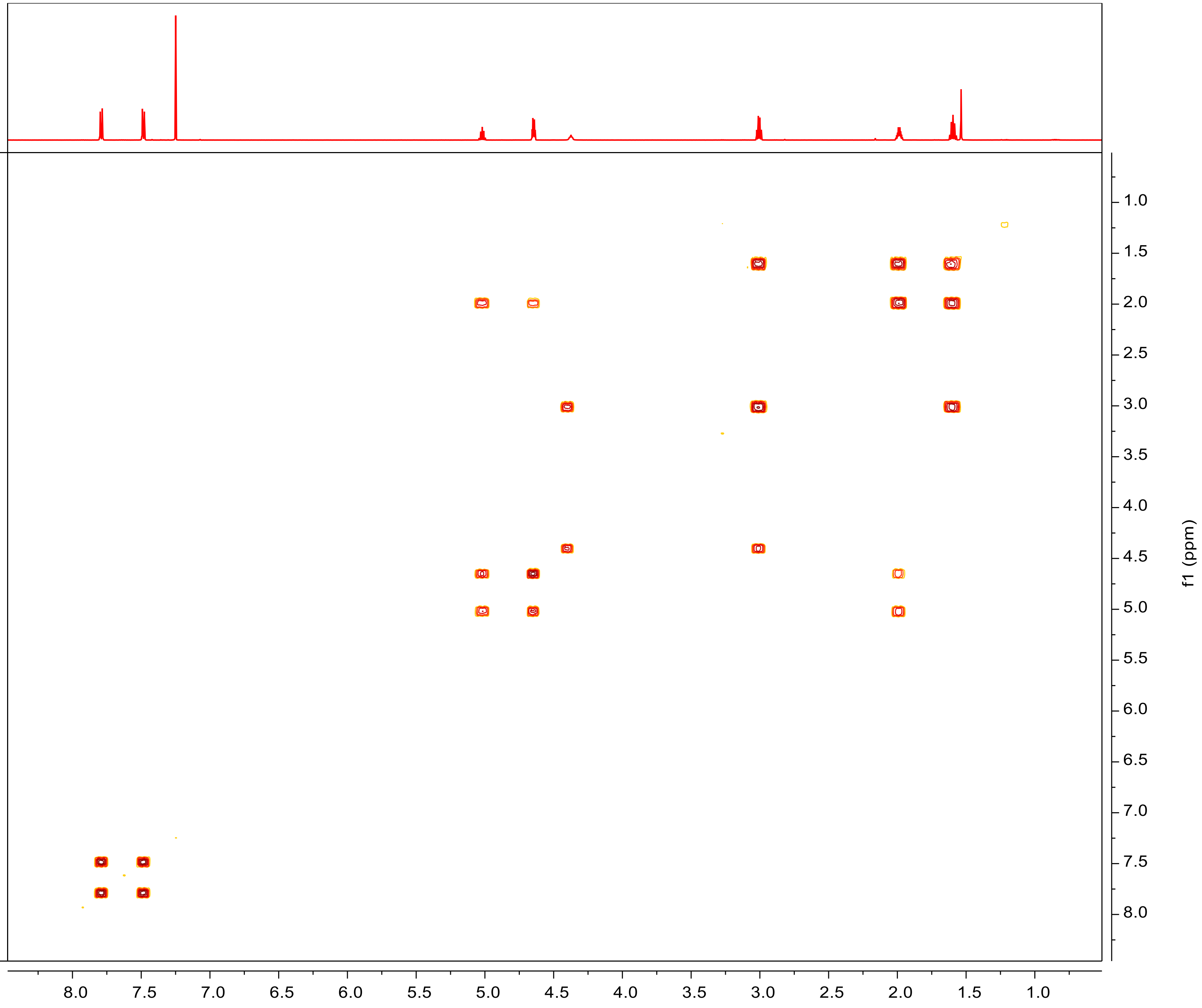

^{13}C-NMR (150 MHz, $CDCl_3$)

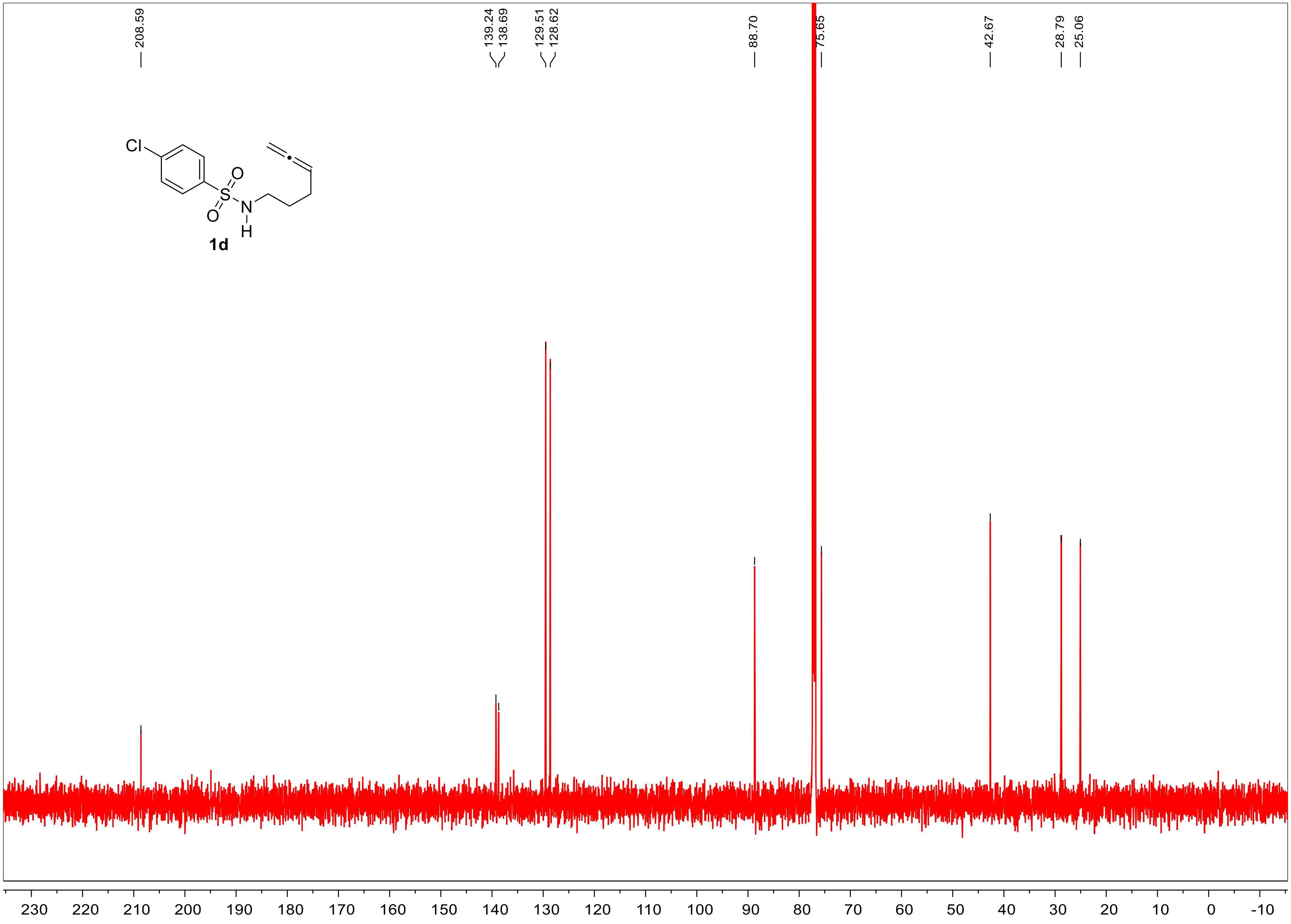

DEPT 135 (150 MHz, $CDCl_3$)

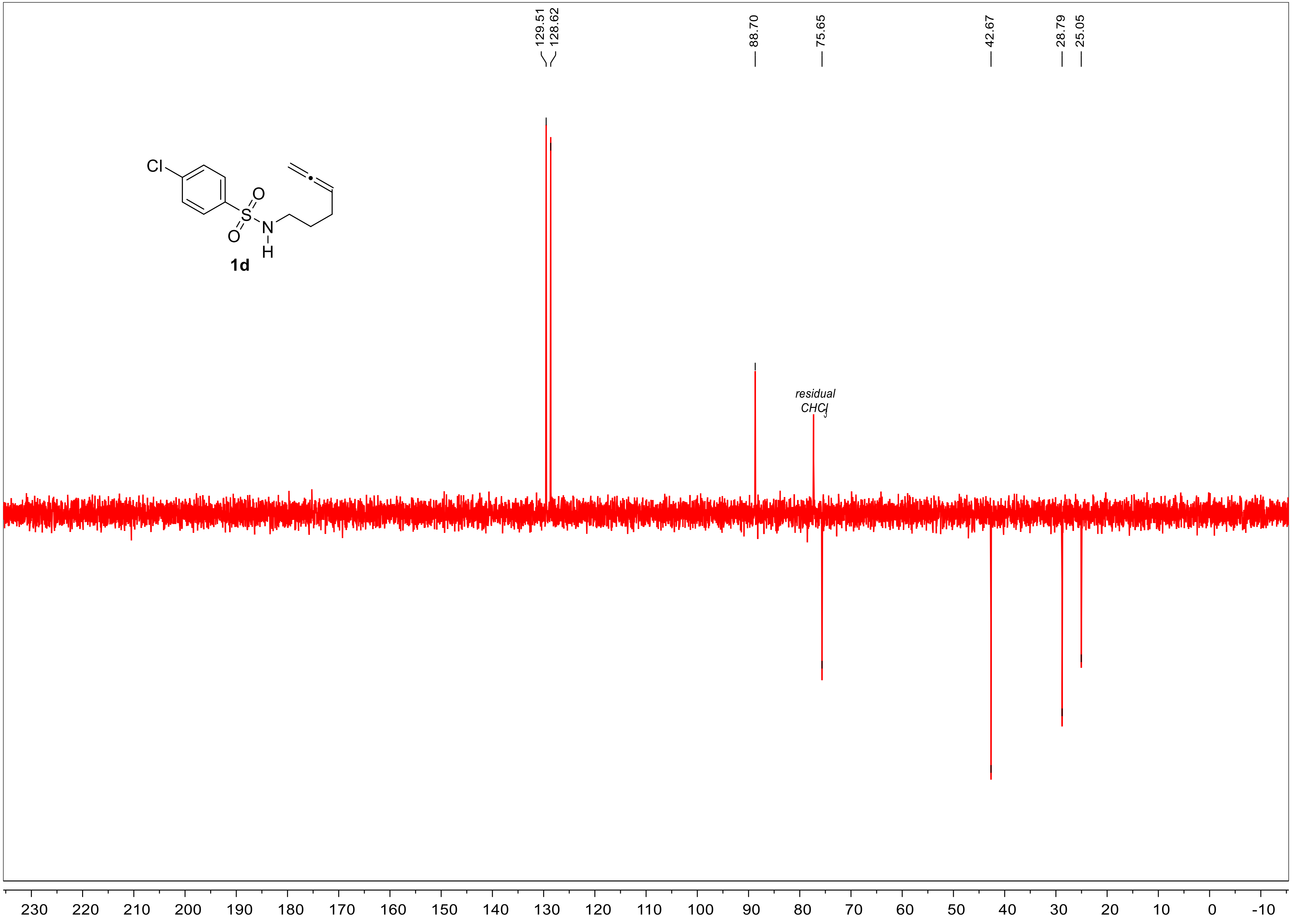

^{1}**H-NMR** (600 MHz, $CDCl_3$)

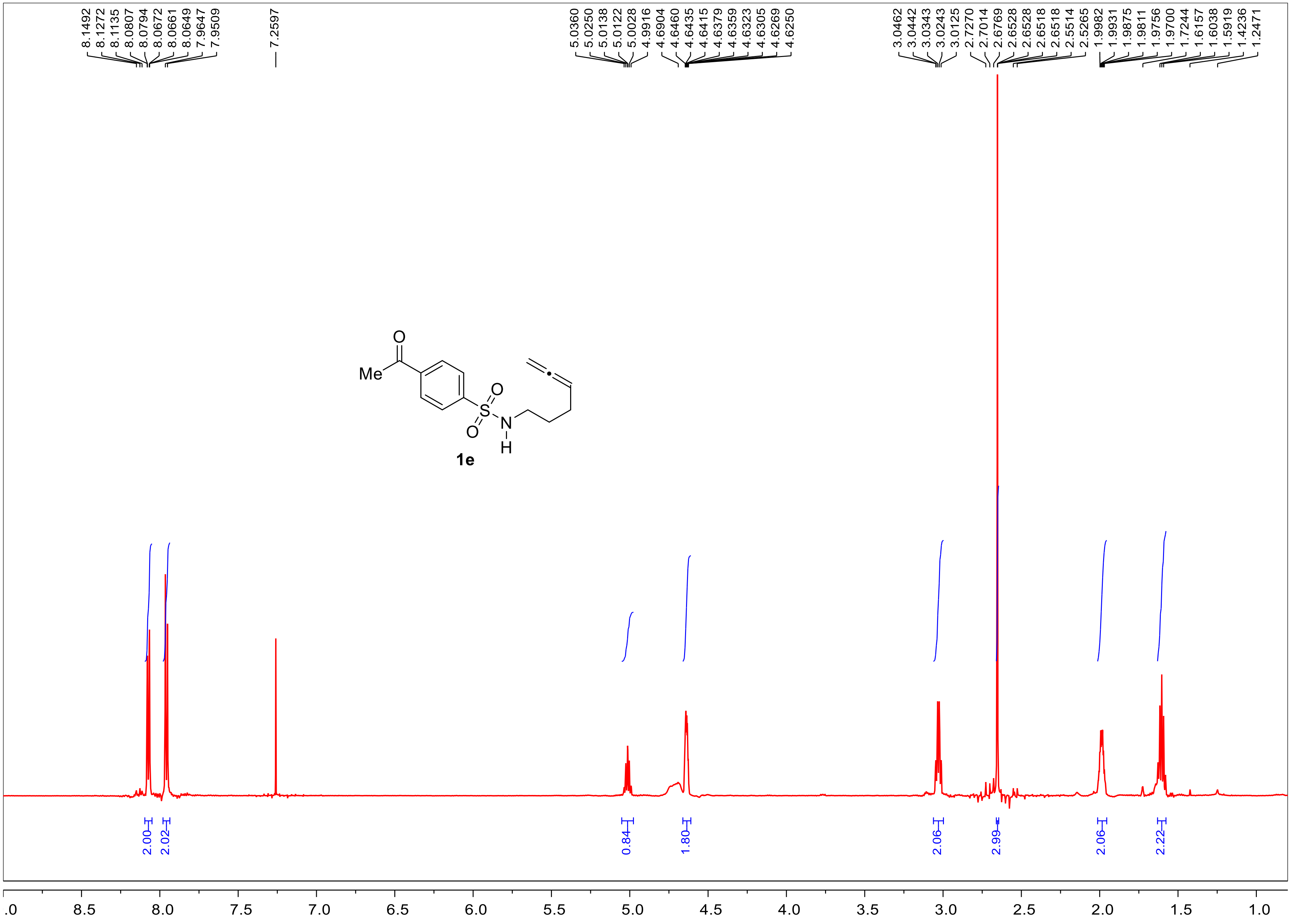

2D - COSY (600 MHz, $CDCl_3$)

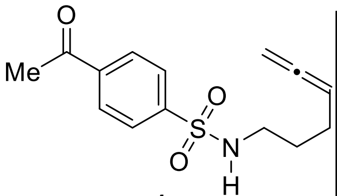

1e

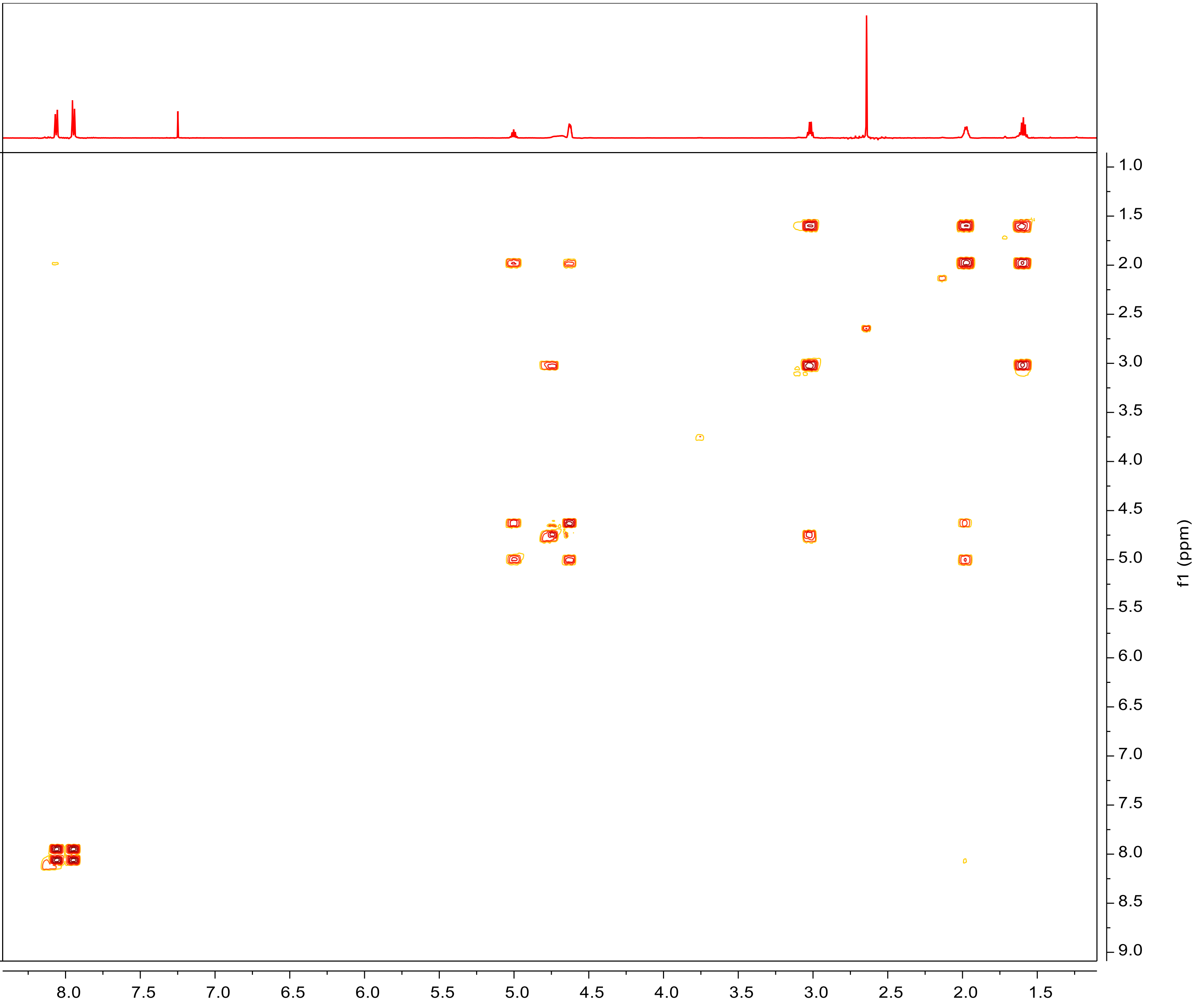

$^{13}C\{^{1}H\}$ NMR (150 MHz, $CDCl_3$)

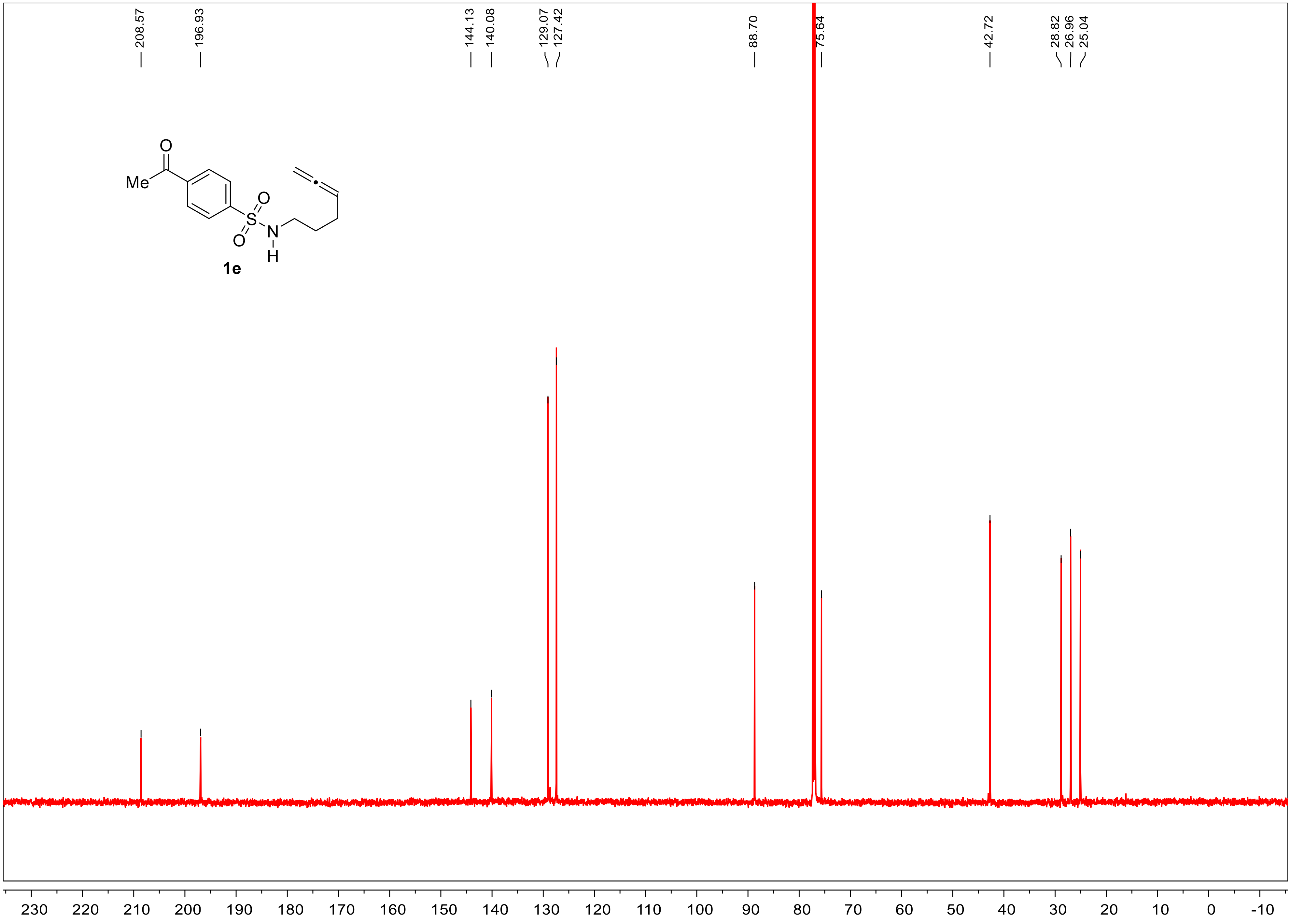

DEPT 135 (150 MHz, $CDCl_3$)

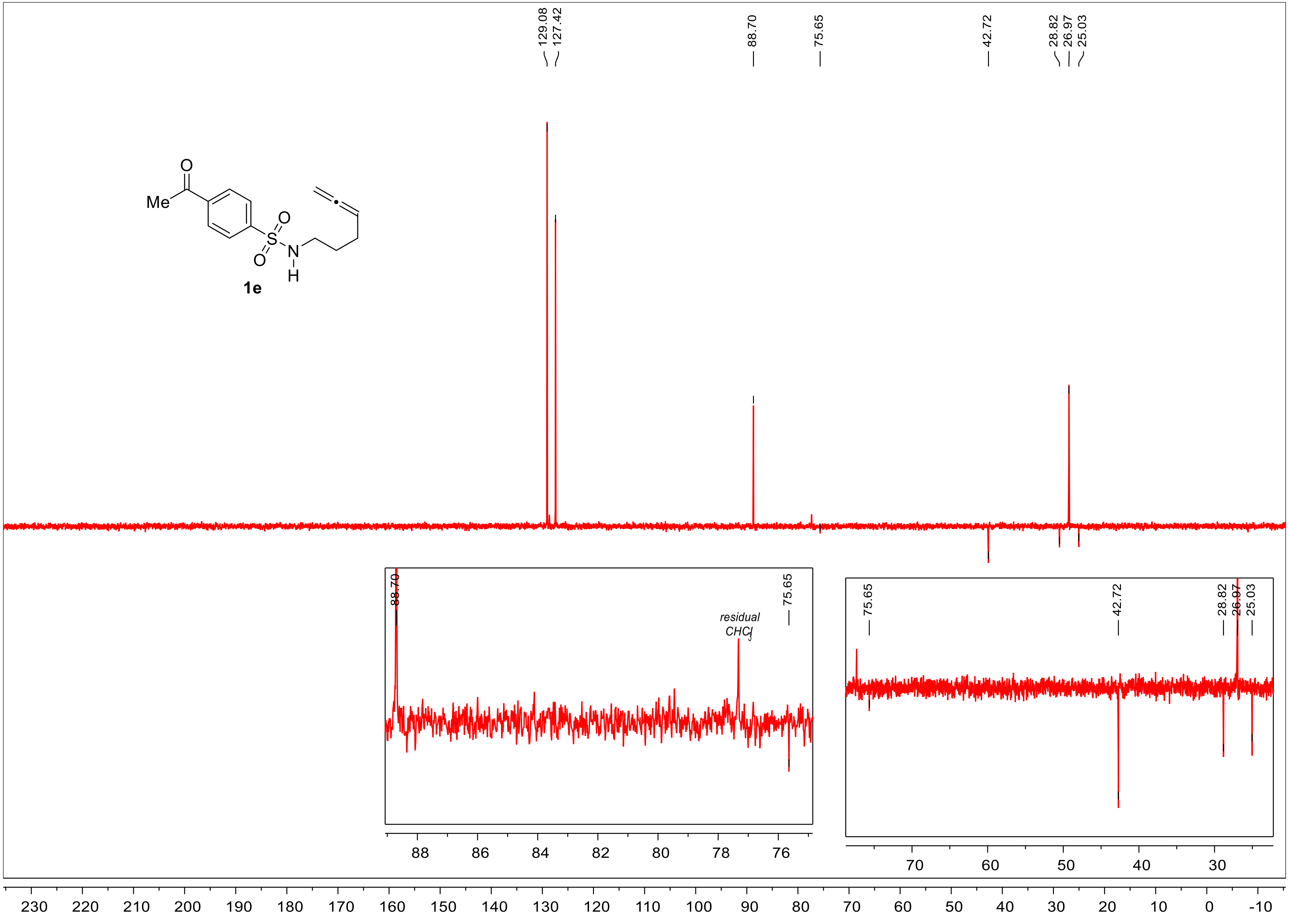

^{1}H-NMR (600 MHz, $CDCl_3$)

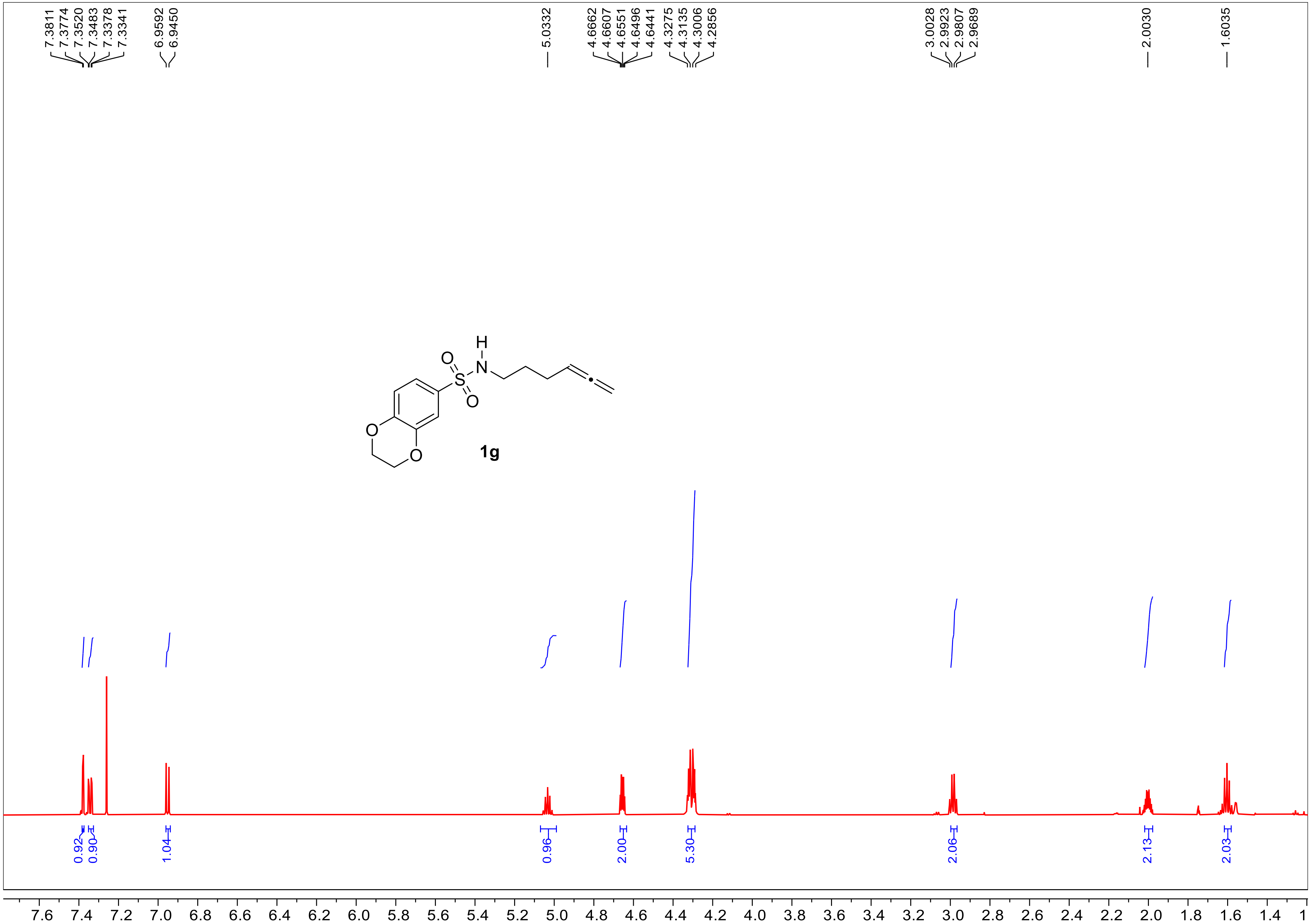

2D - COSY (600 MHz, $CDCl_3$)

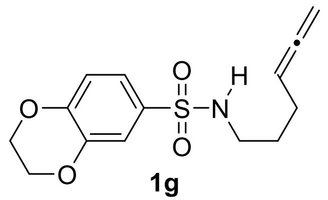

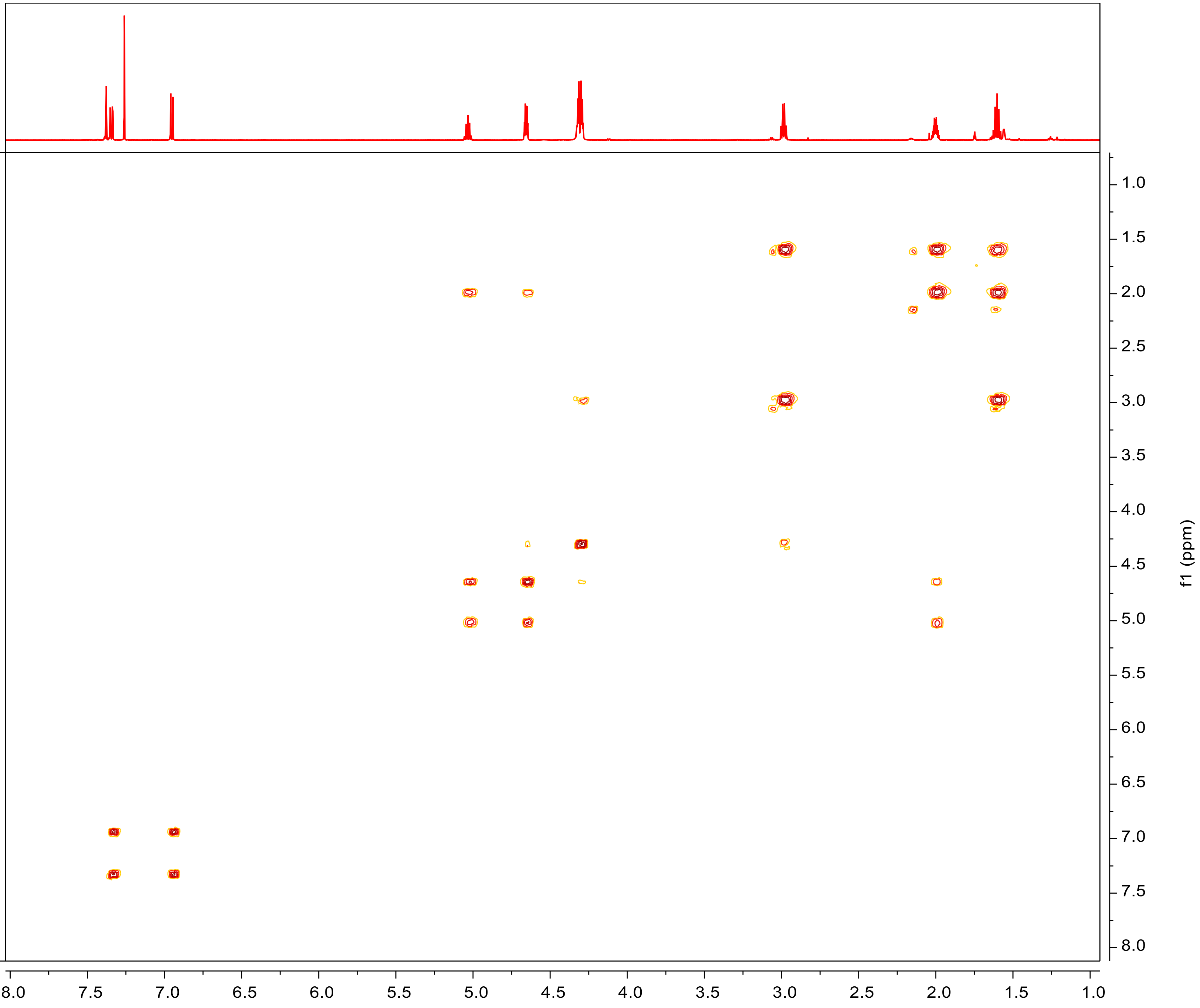

$^{13}C\{^1H\}$ NMR (150 MHz, $CDCl_3$)

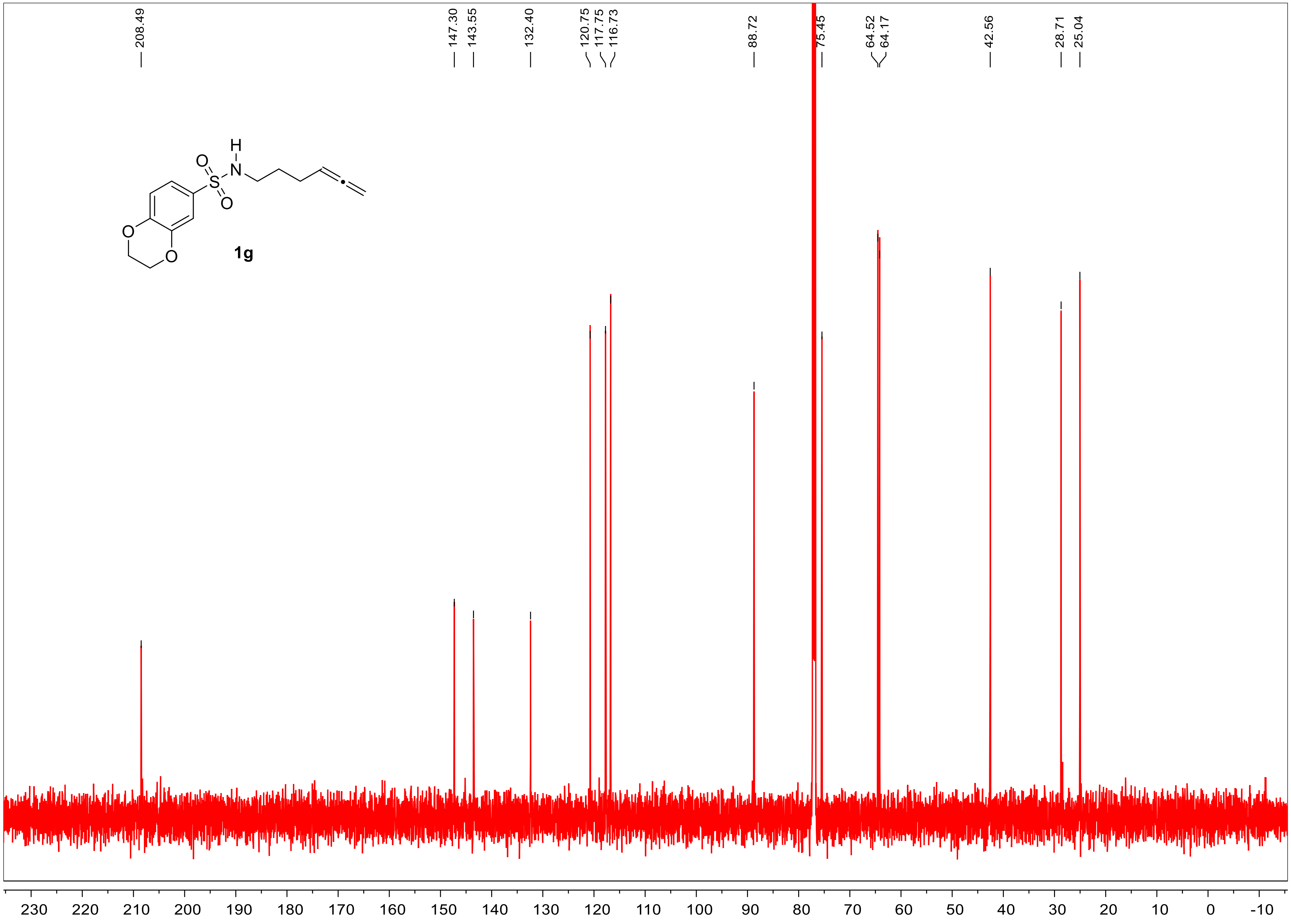

DEPT 135 (150 MHz, $CDCl_3$)

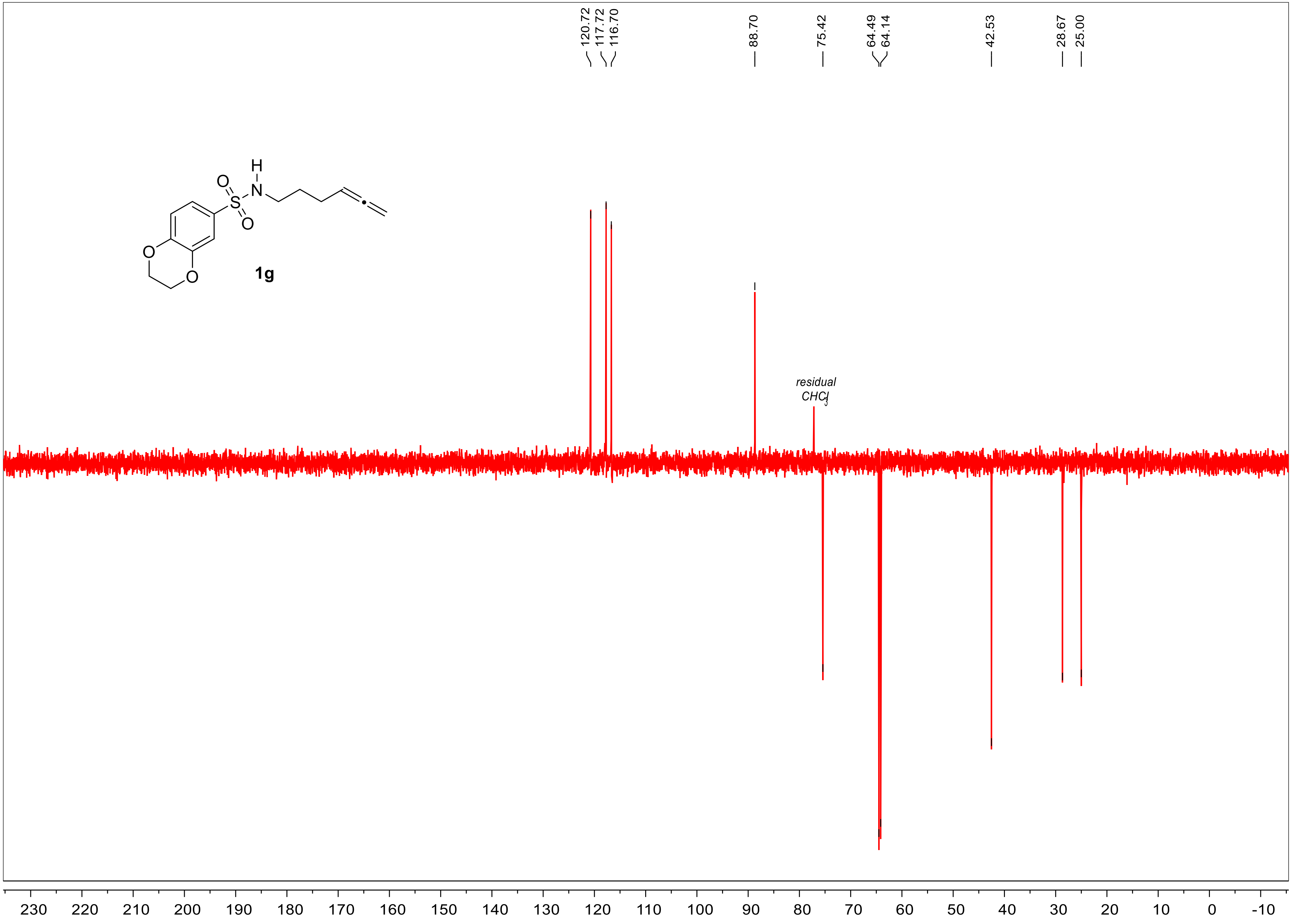

^{1}H-NMR (600 MHz, $CDCl_3$)

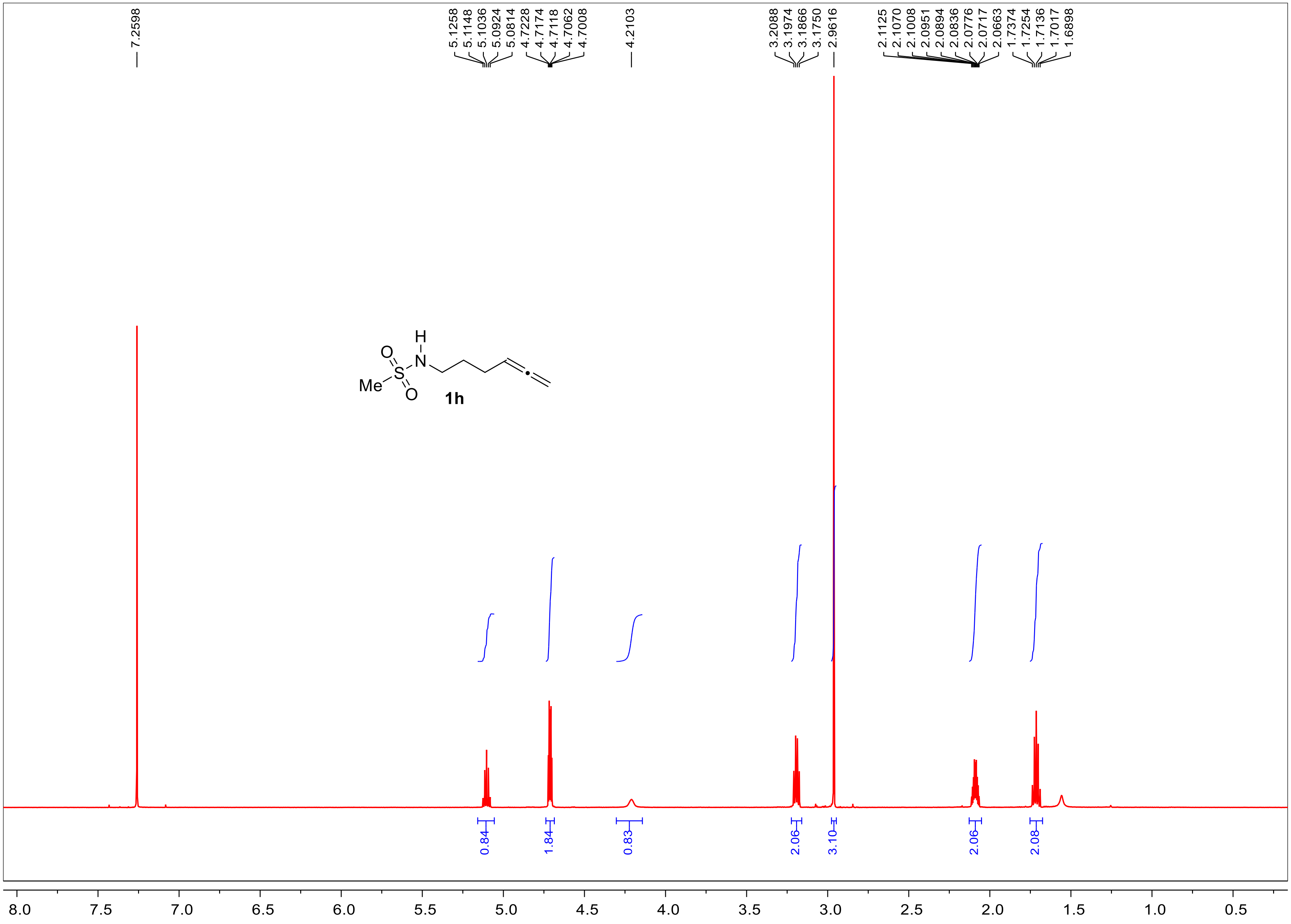

2D - COSY (600 MHz, $CDCl_3$)

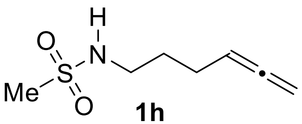

1h

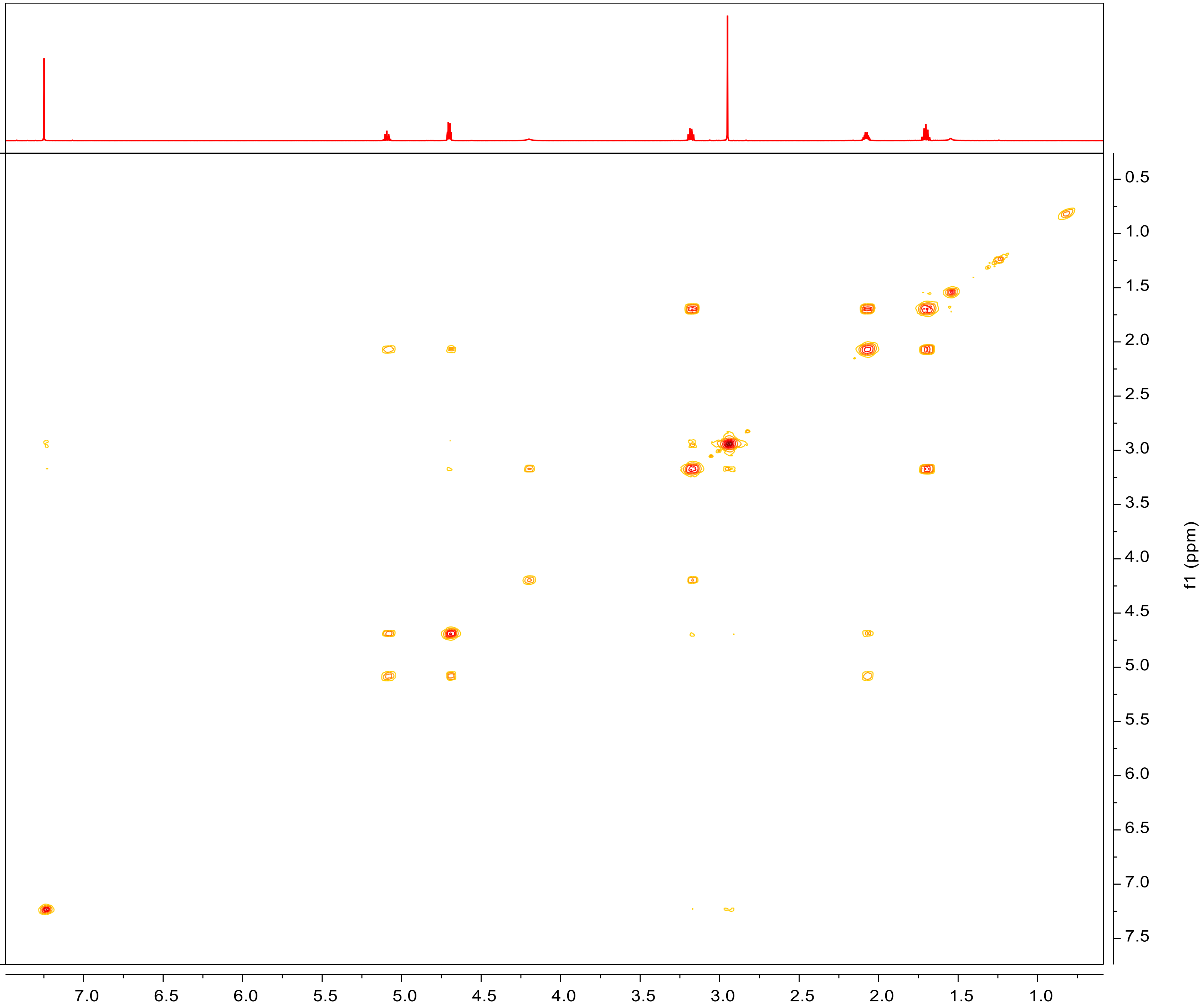

^{13}C{^{1}H} NMR (150 MHz, $CDCl_3$)

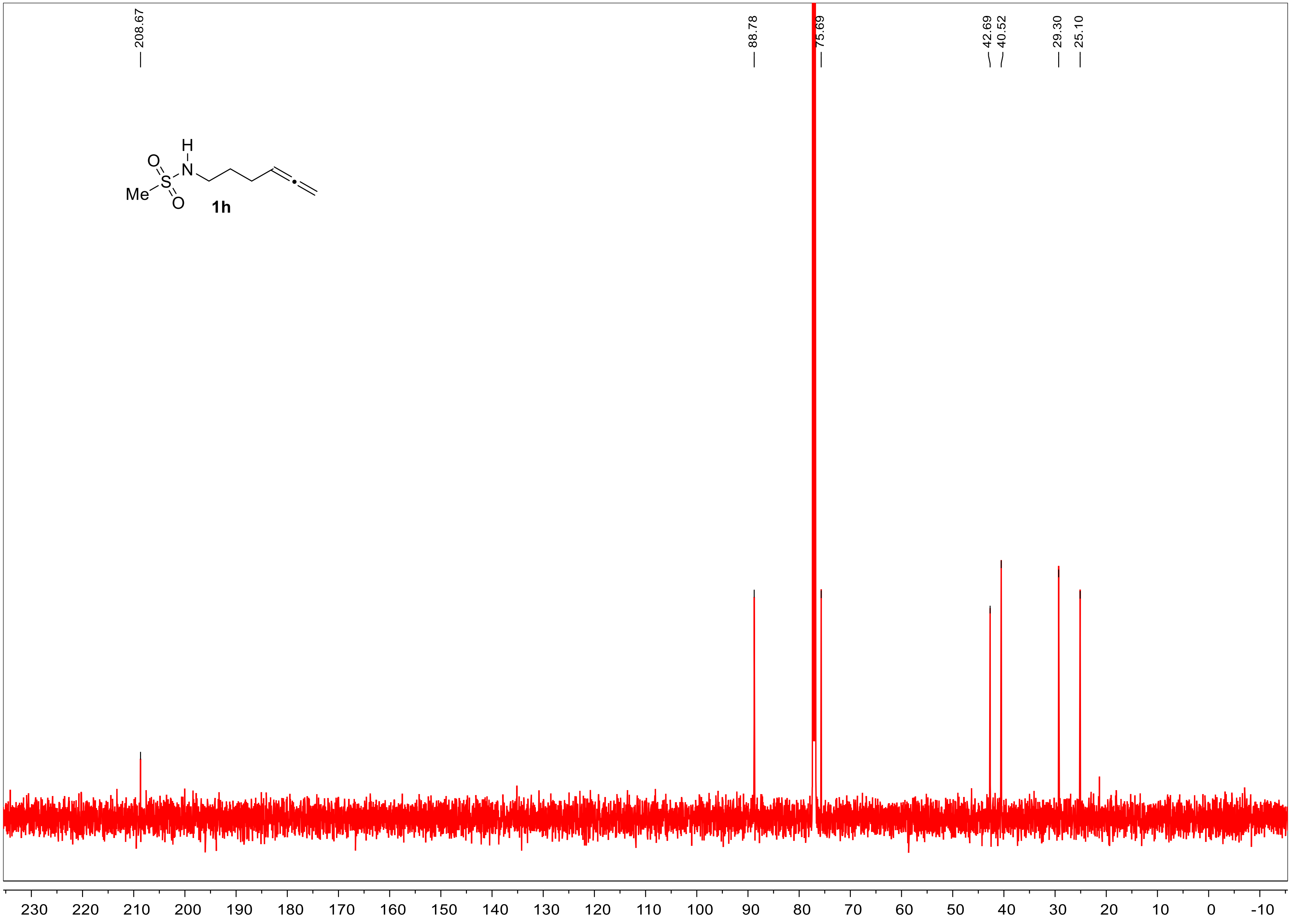

DEPT 135 (150 MHz, $CDCl_3$)

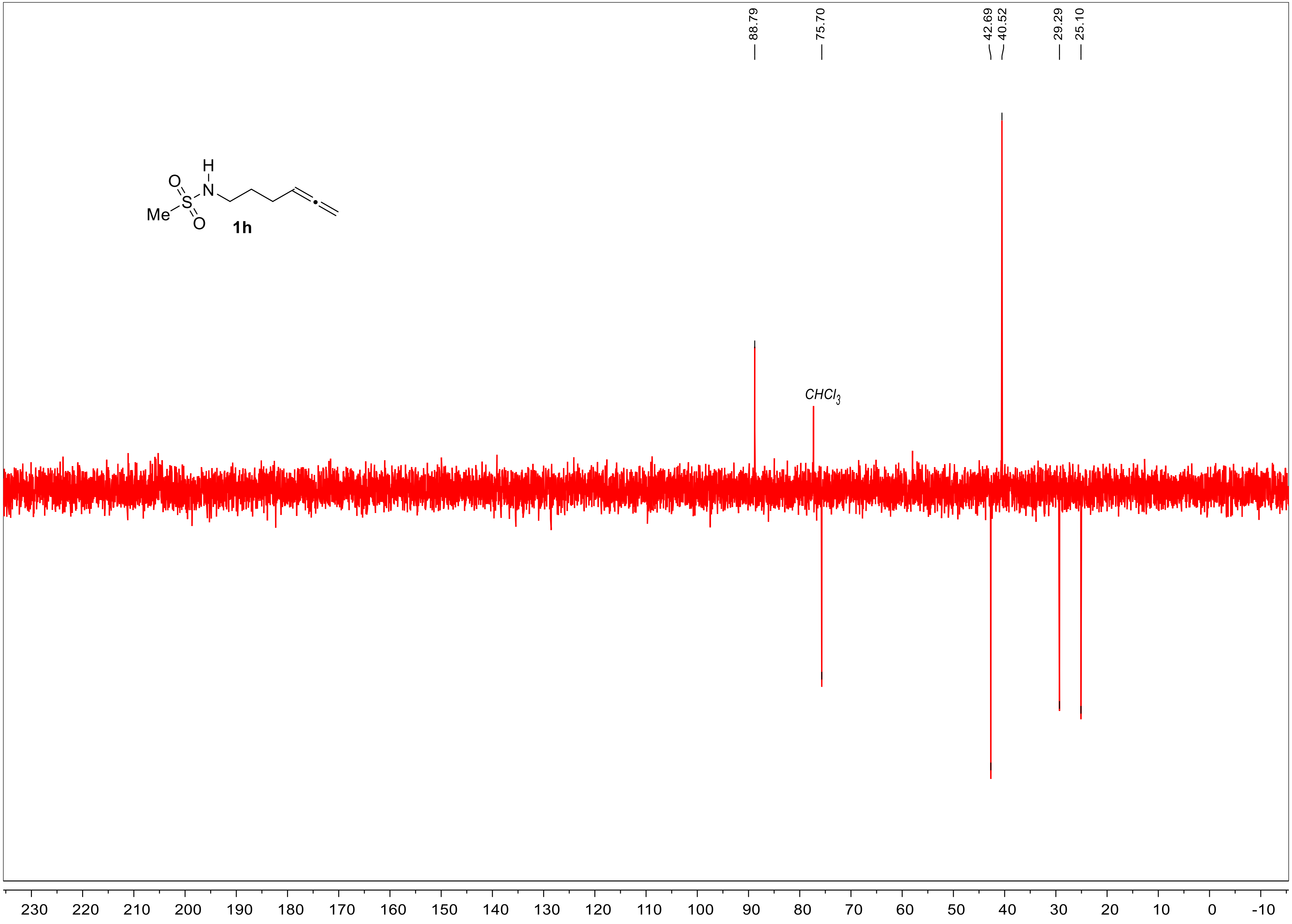

^{1}H-NMR (600 MHz, $CDCl_3$)

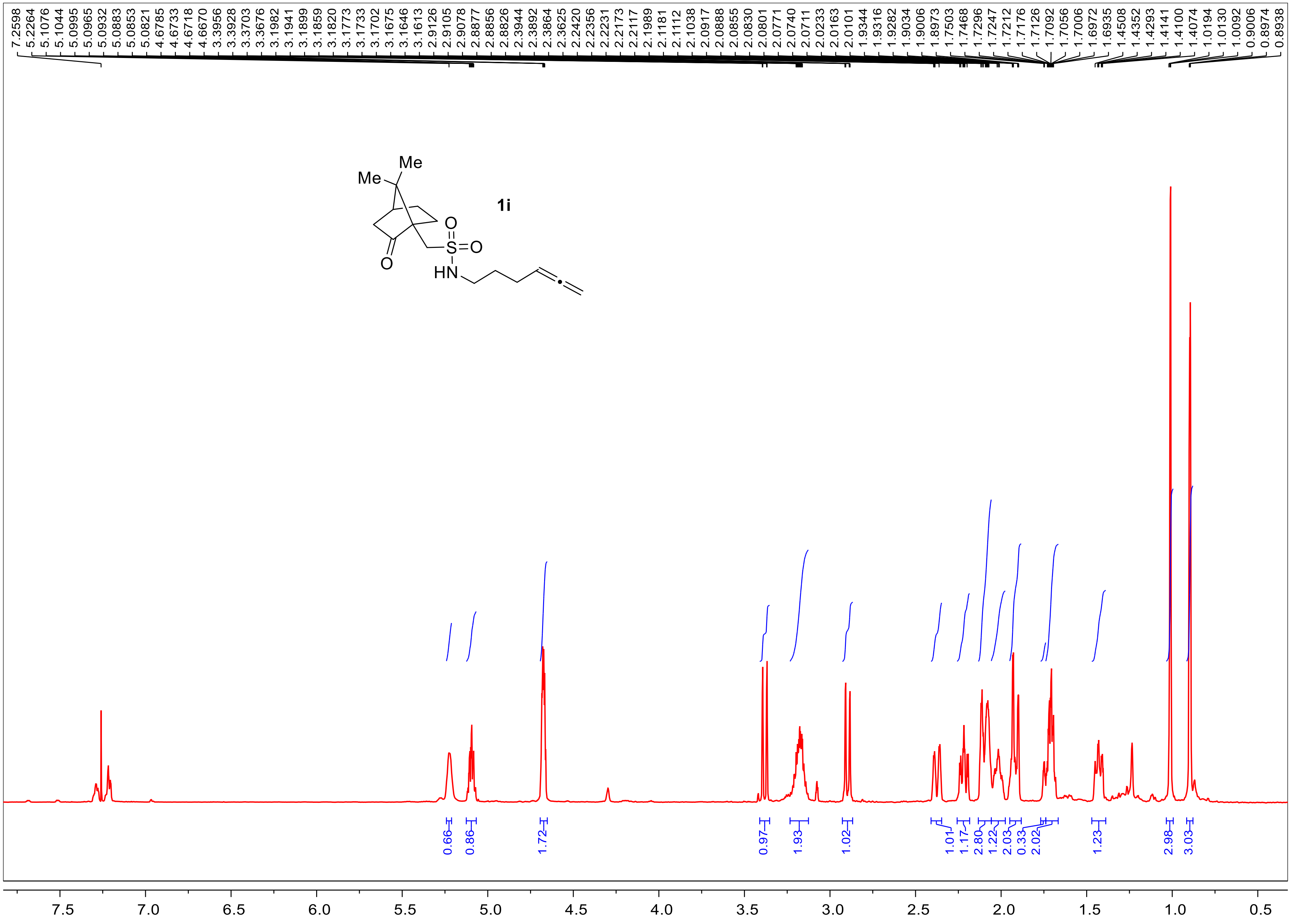

2D - COSY (600 MHz, $CDCl_3$)

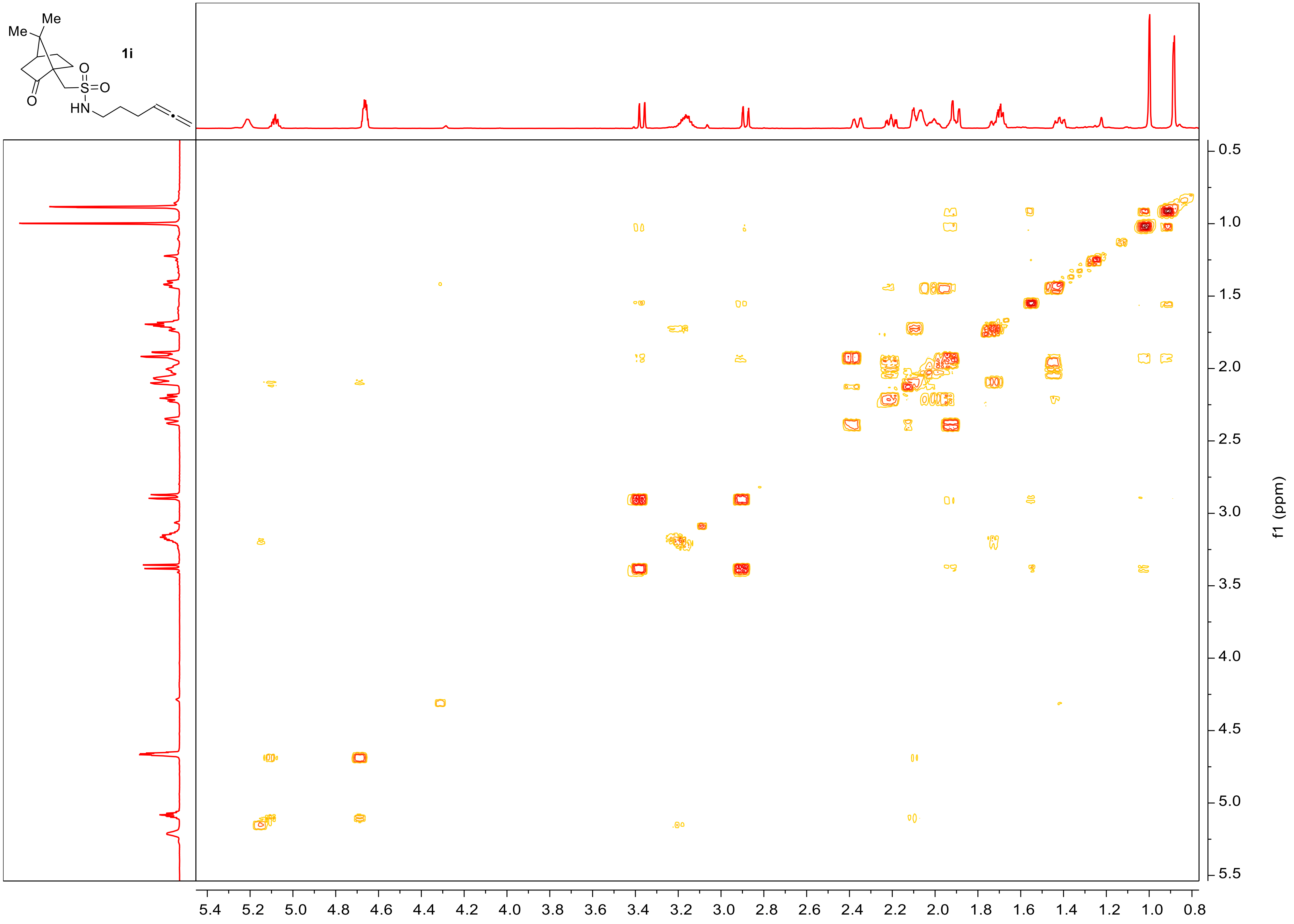

$^{13}C\{^{1}H\}$ NMR (150 MHz, $CDCl_3$)

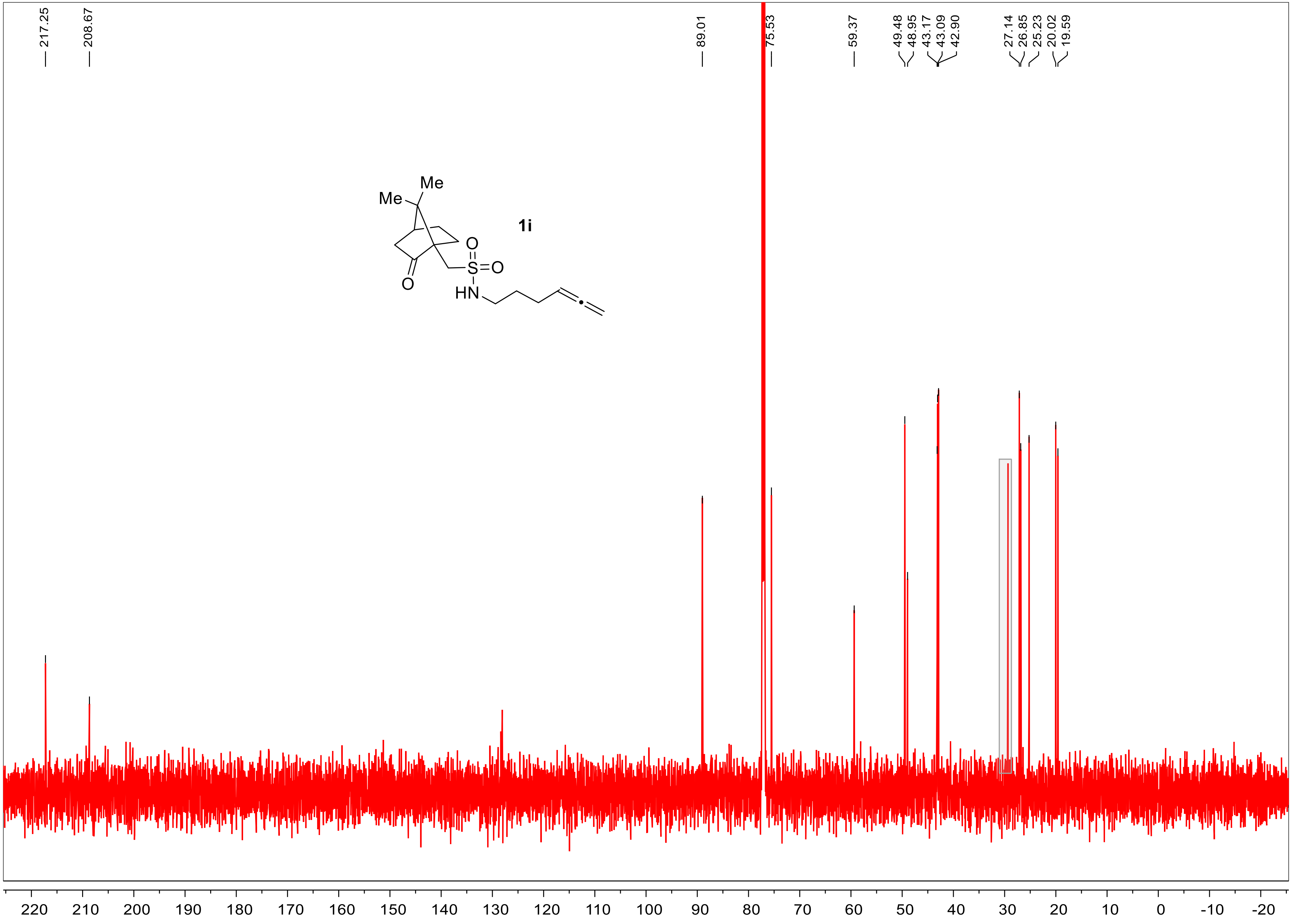

DEPT 135 (150 MHz, $CDCl_3$)

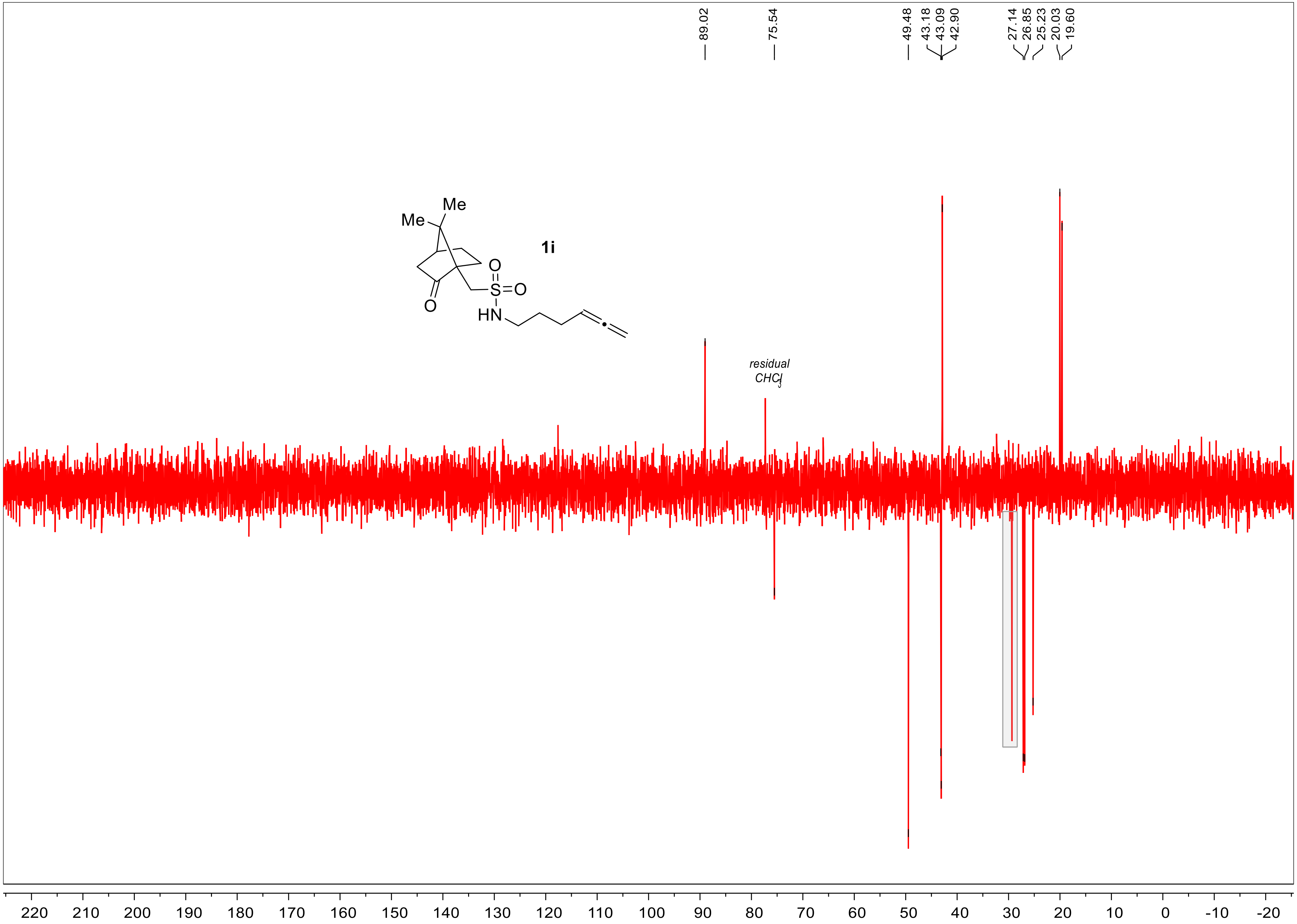

^{1}H-NMR (600 MHz, $CDCl_3$)

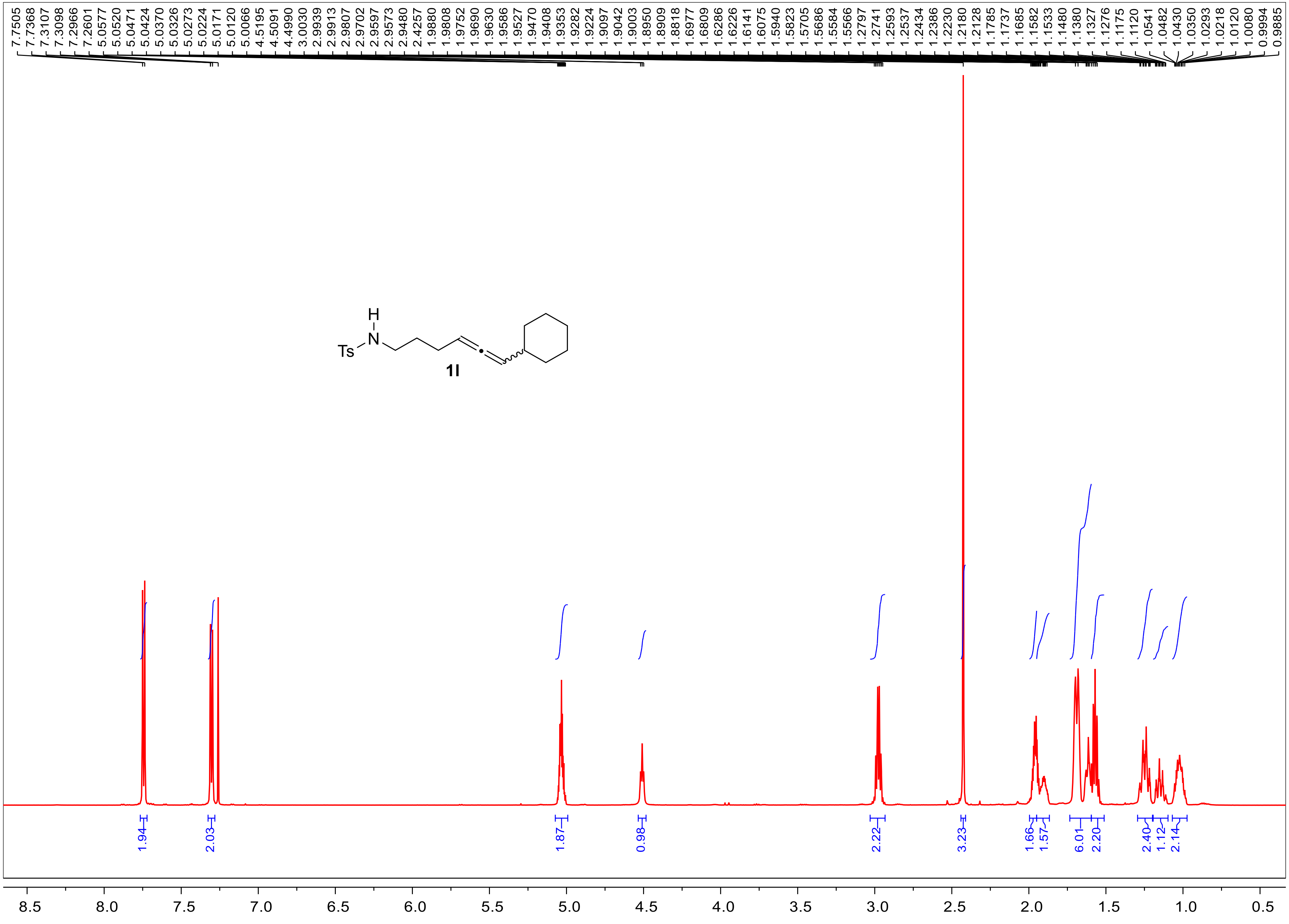

2D - COSY (600 MHz, $CDCl_3$)

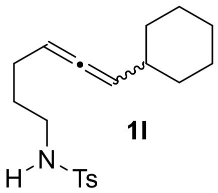

1l

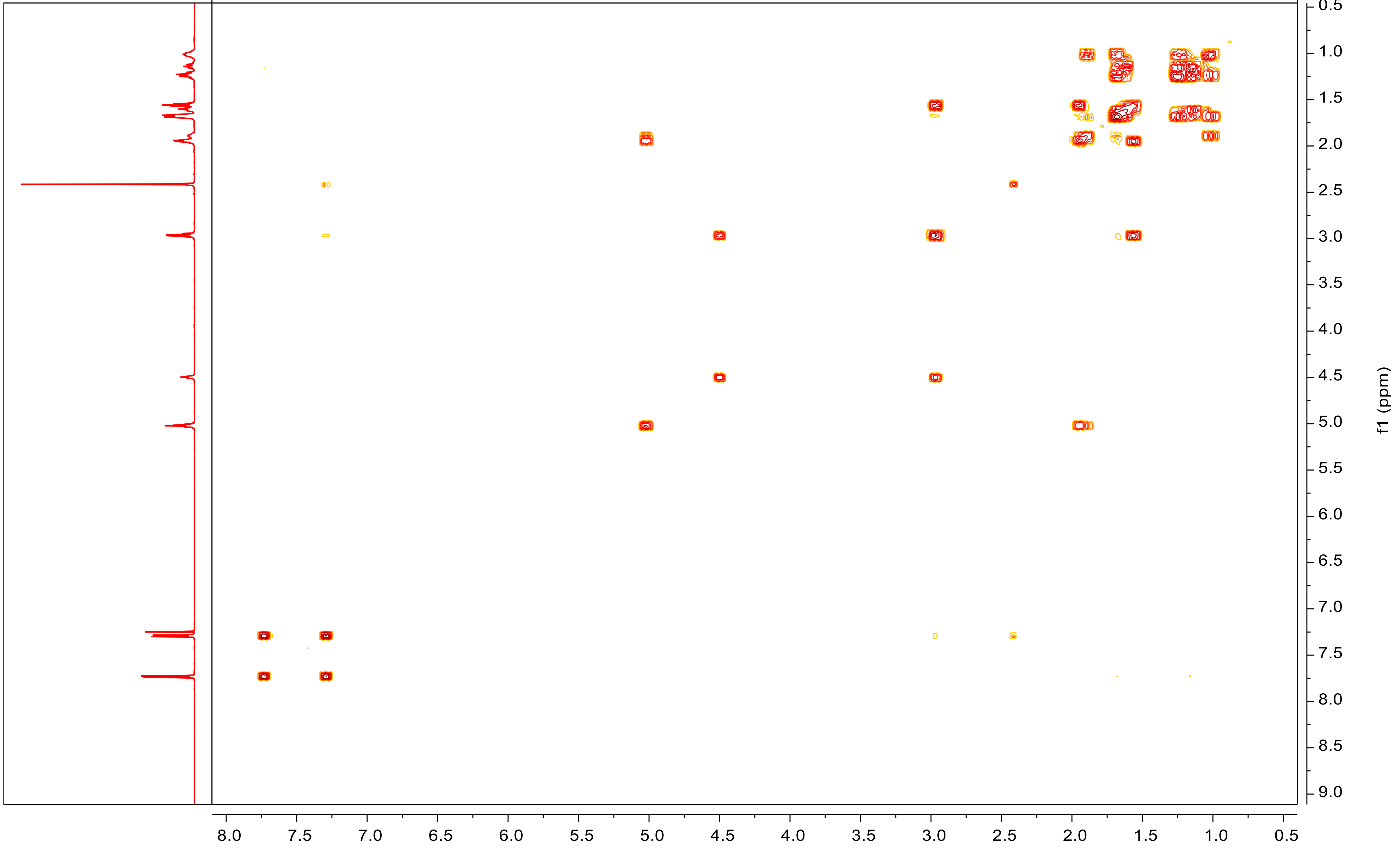

$^{13}C\{^{1}H\}$ **NMR** (150 MHz, $CDCl_3$)

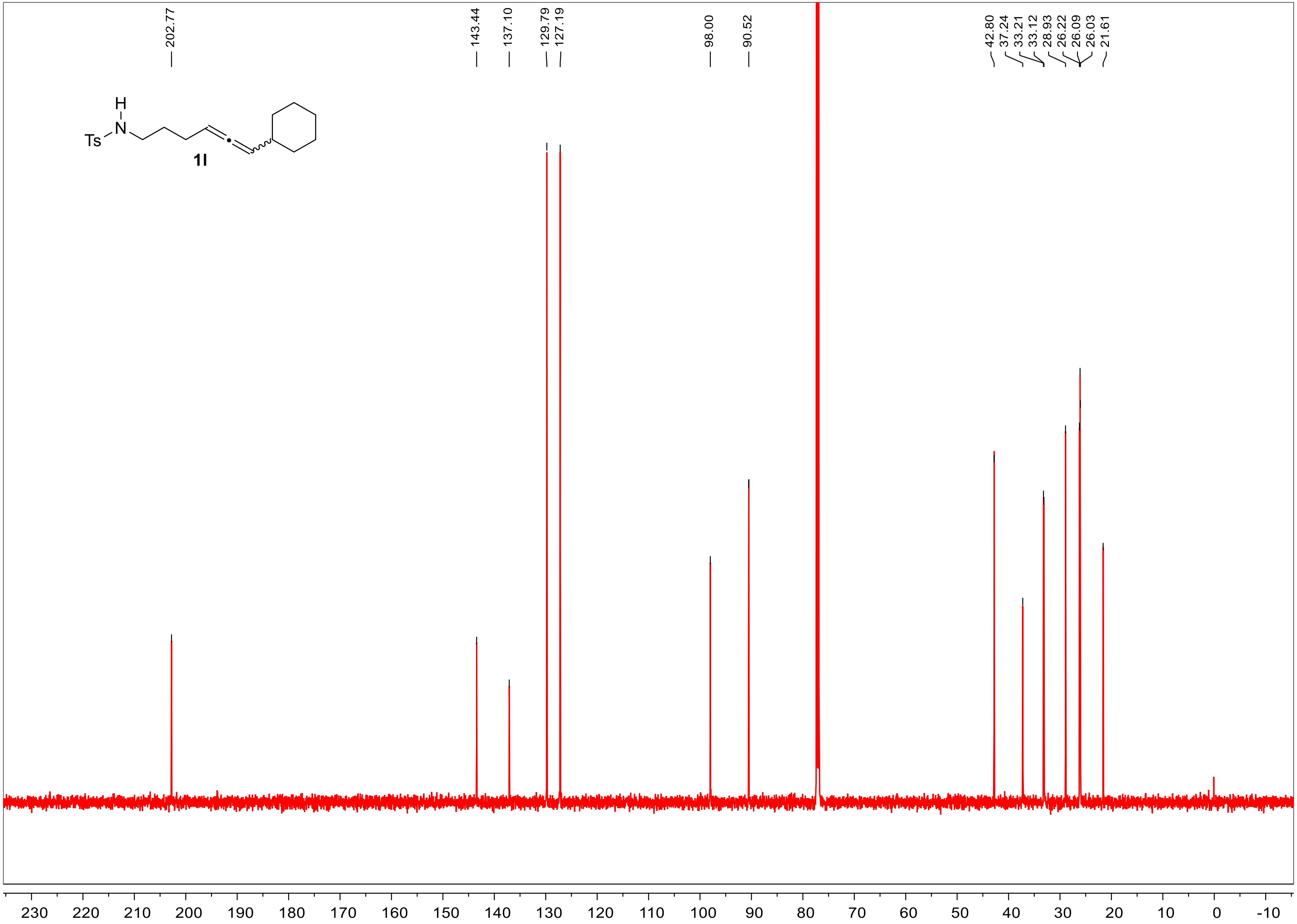

DEPT 135 (150 MHz, $CDCl_3$)

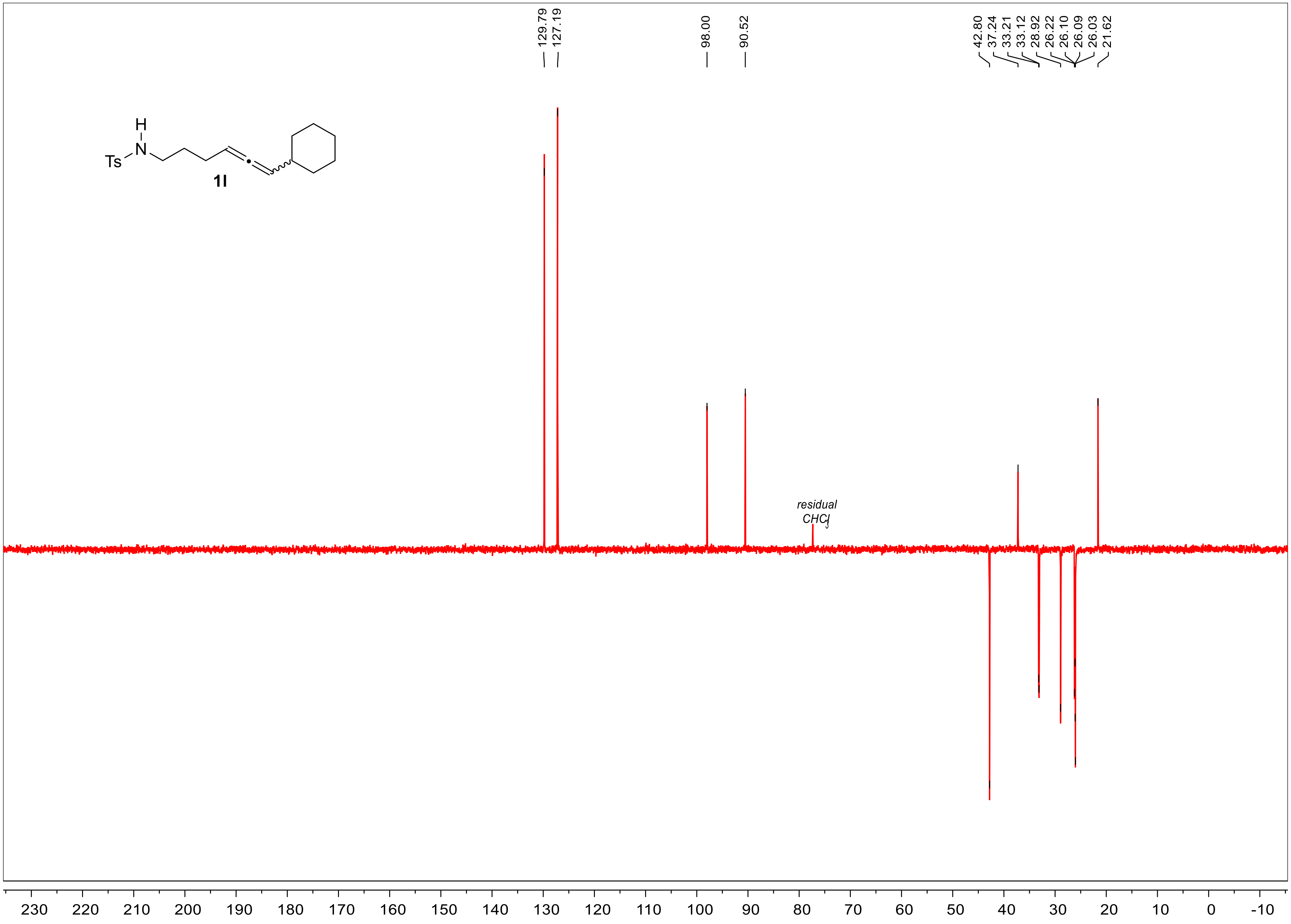

^{1}H-NMR (600 MHz, $CDCl_3$)

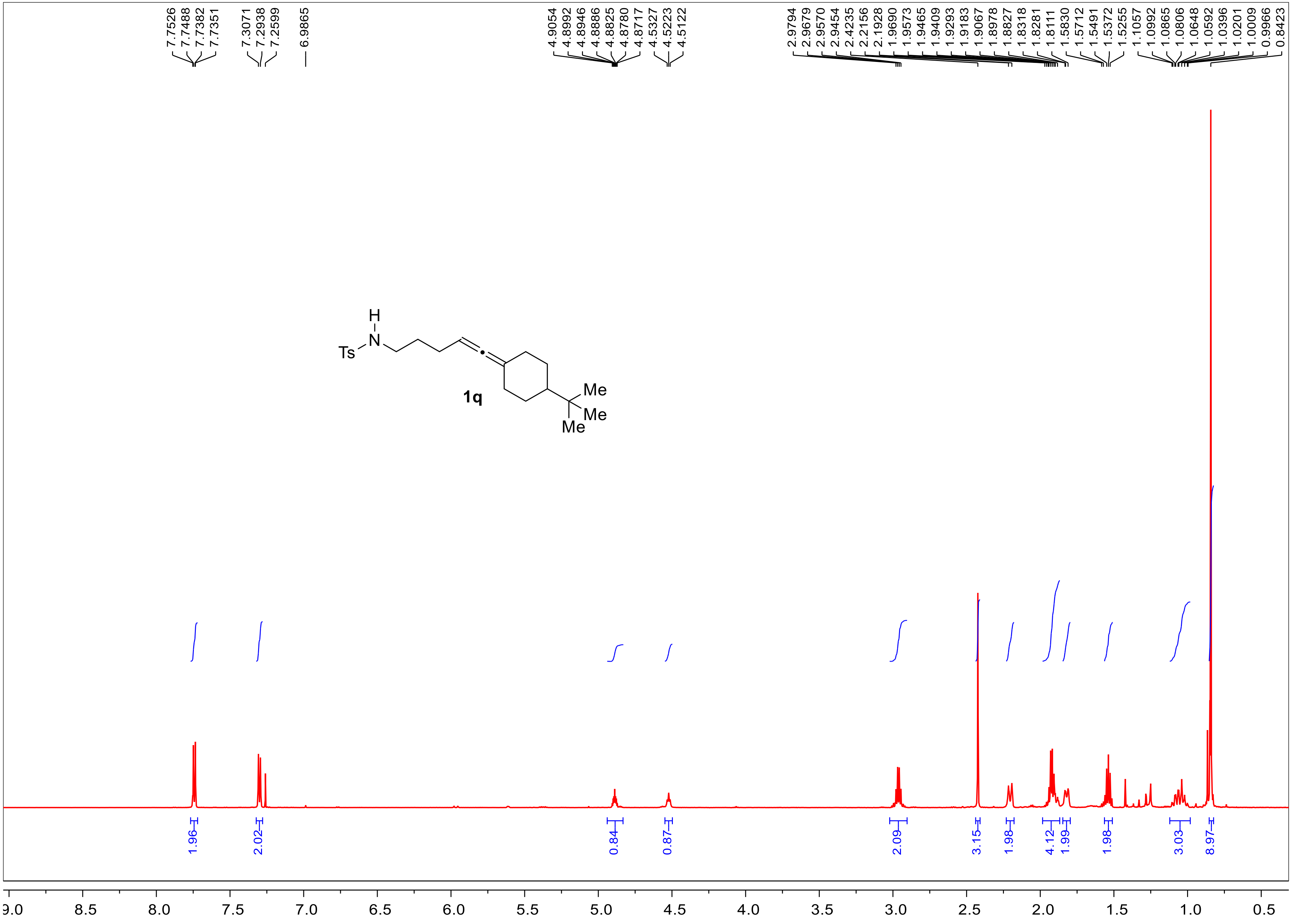

2D - COSY (600 MHz, $CDCl_3$)

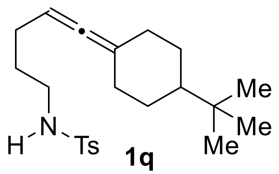

1q

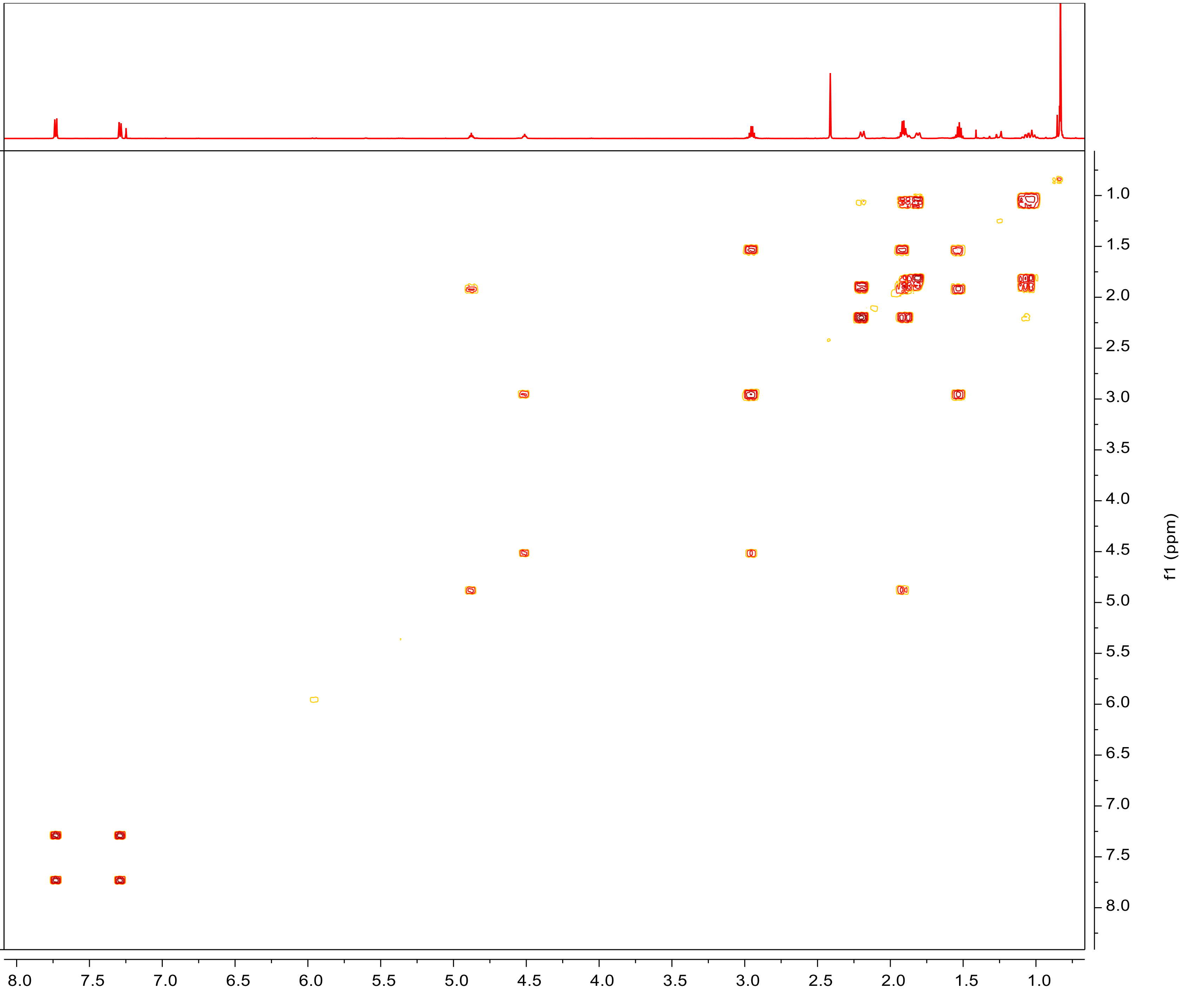

^{13}C{^{1}H} NMR (150 MHz, $CDCl_3$)

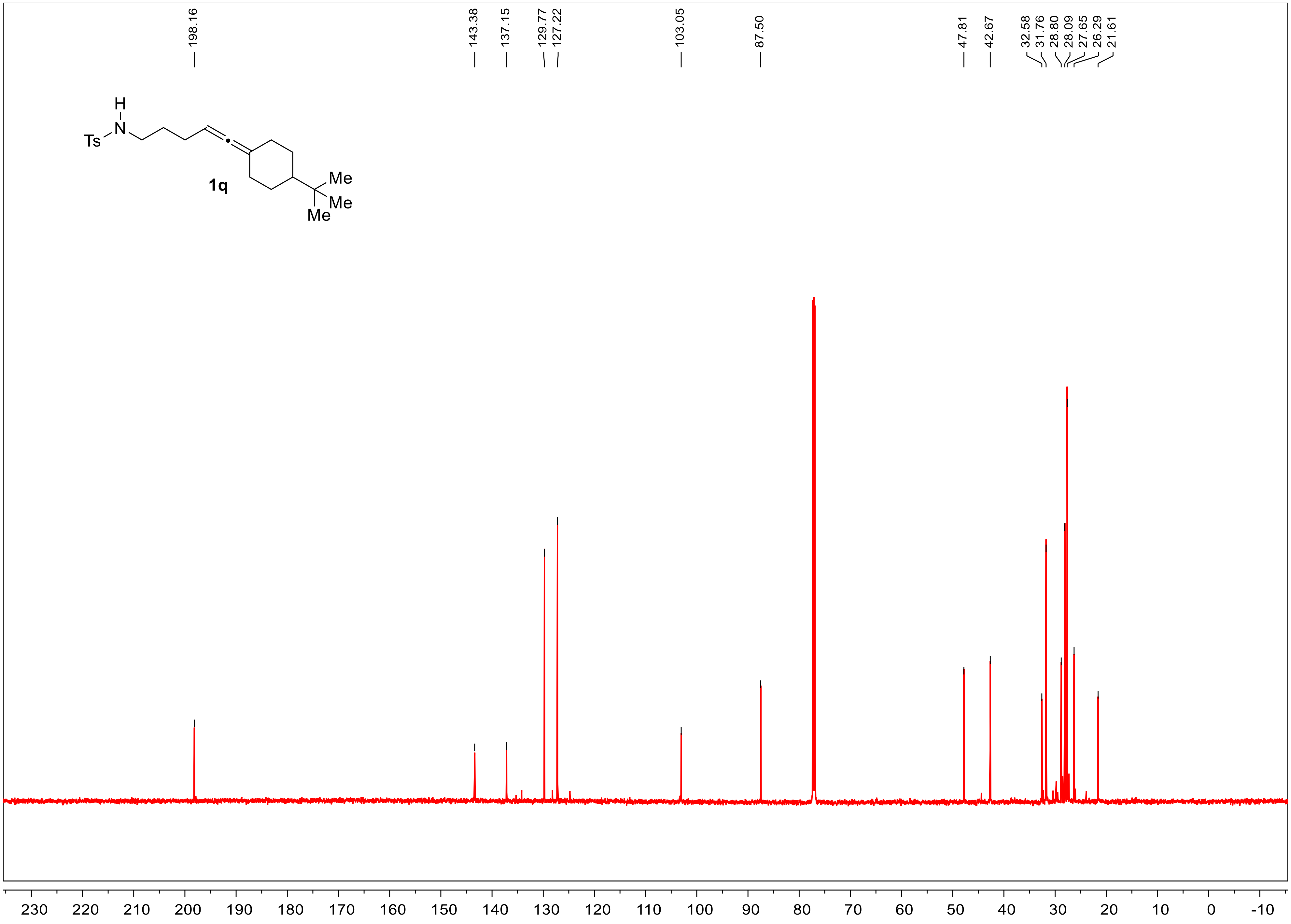

DEPT 135 (150 MHz, $CDCl_3$)

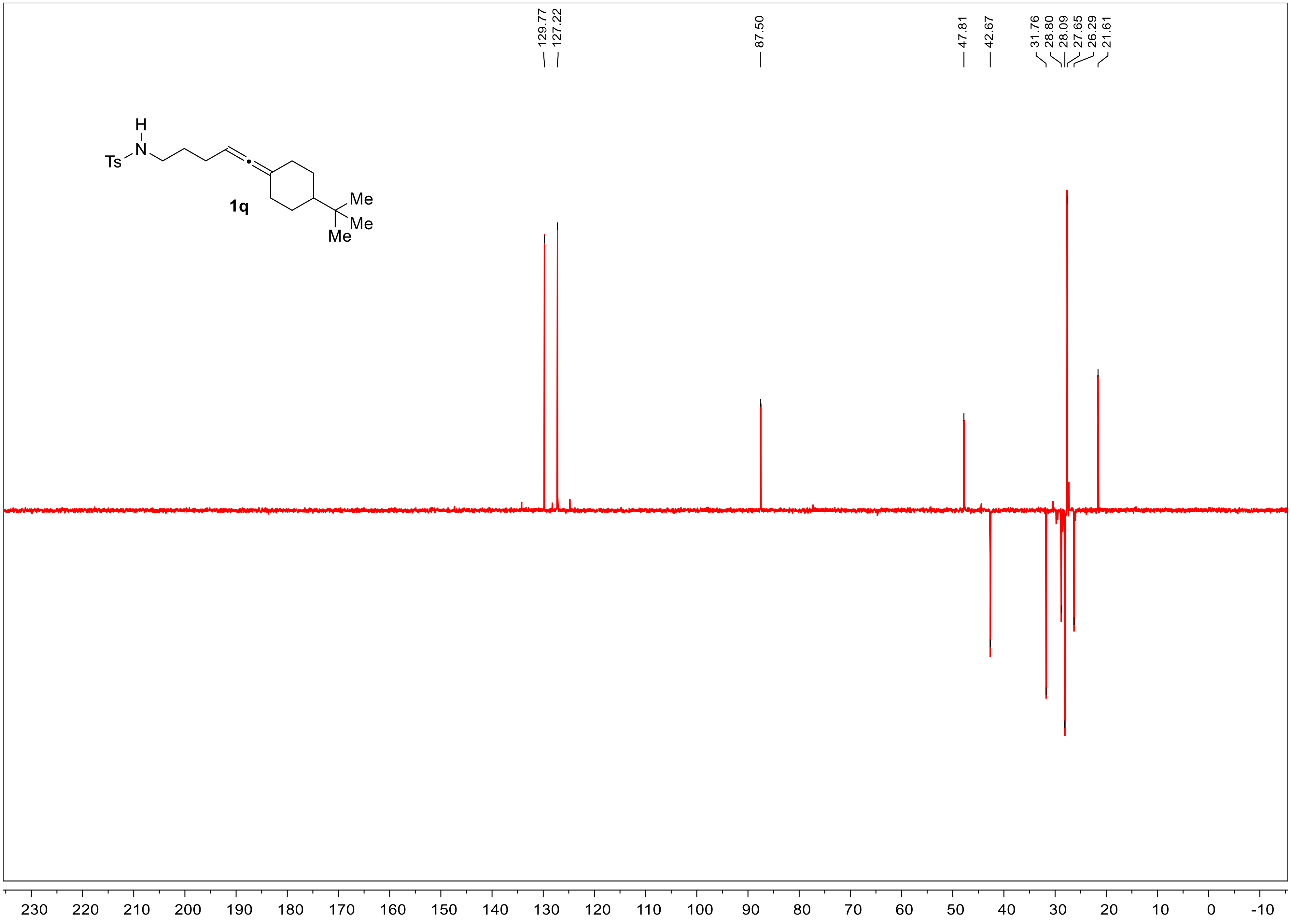

^{1}H-NMR (600 MHz, $CDCl_3$)

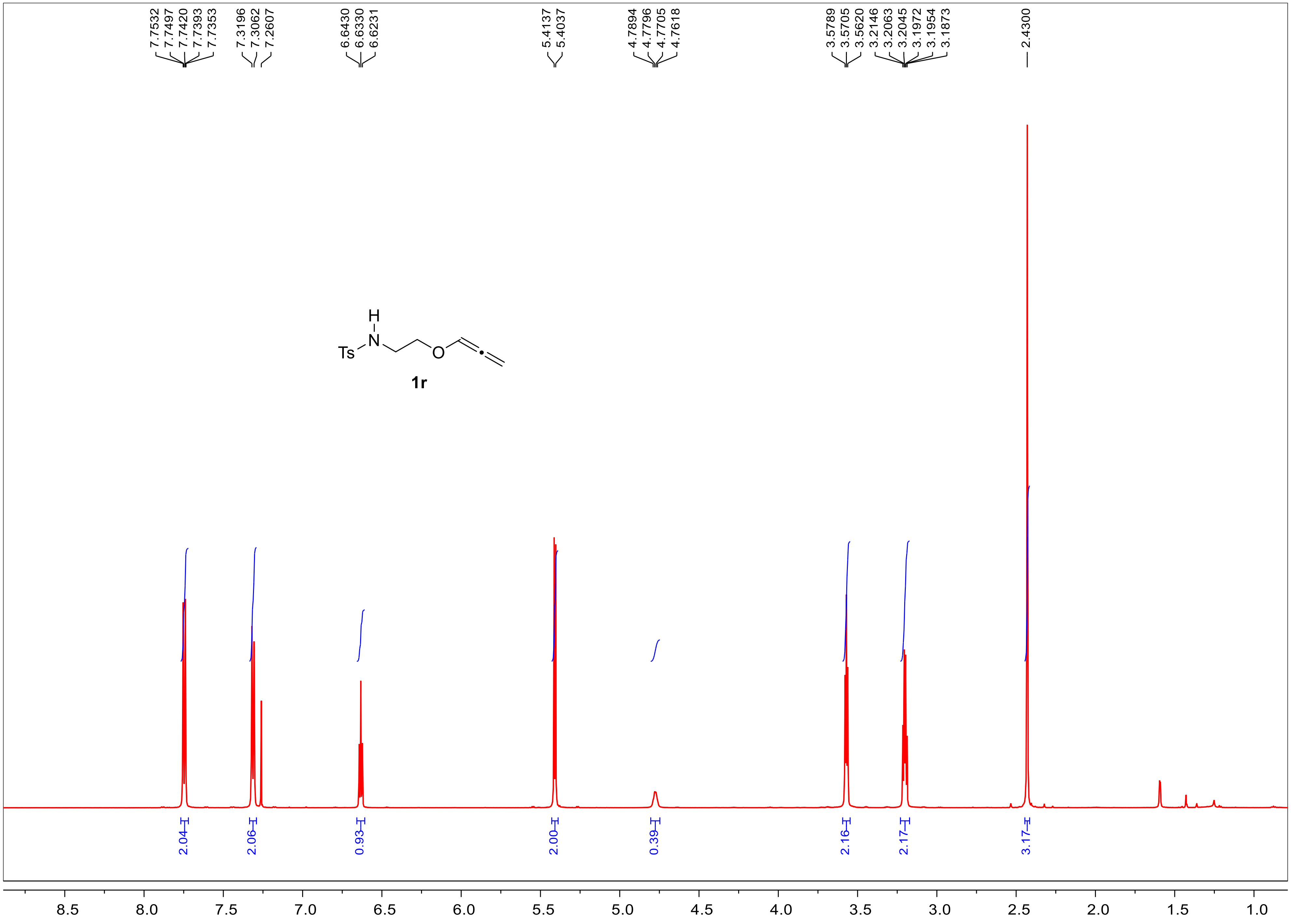

2D - COSY (600 MHz, $CDCl_3$)

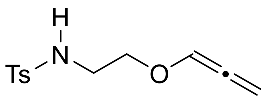

1r

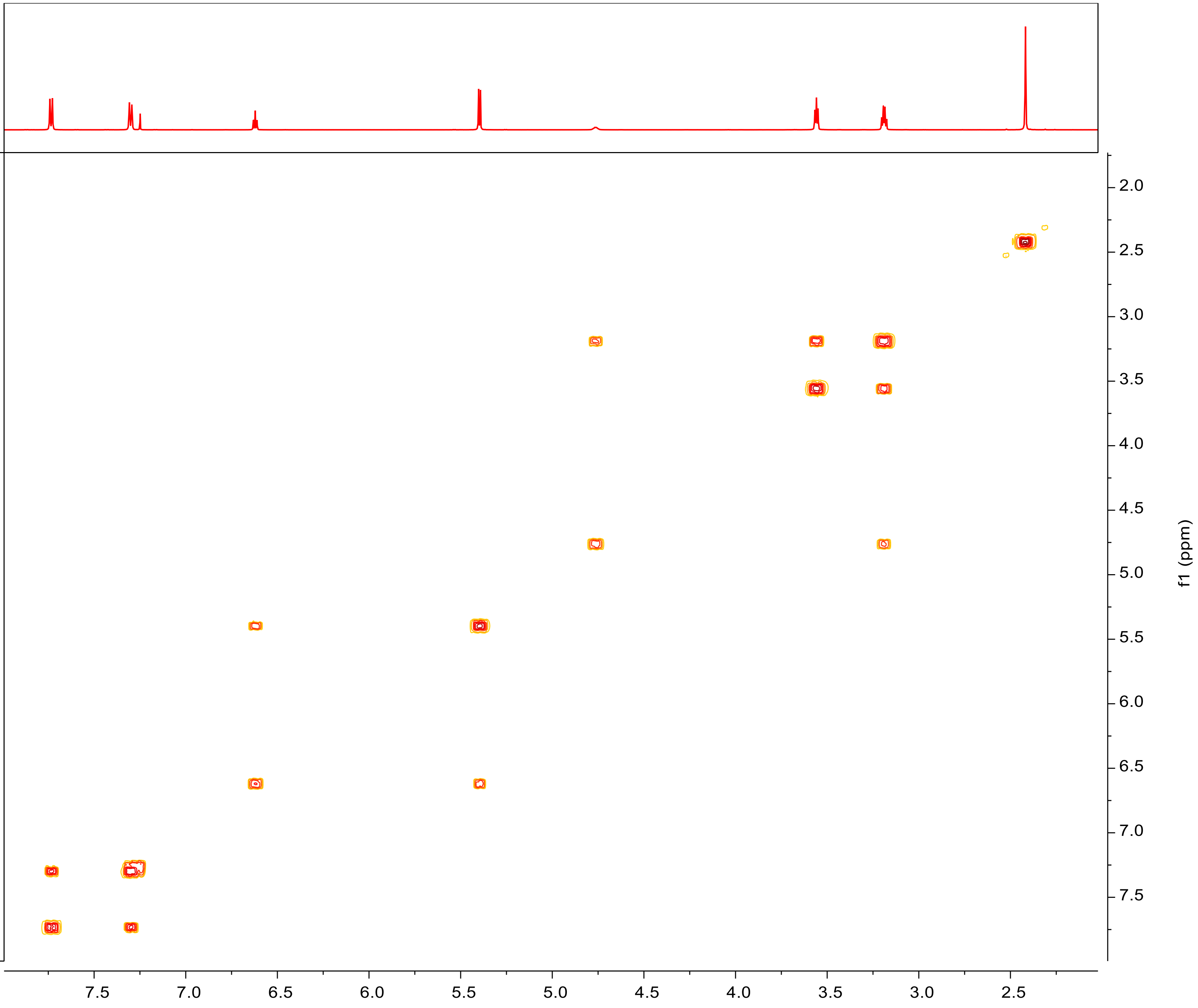

$^{13}C\{^{1}H\}$ **NMR** (150 MHz, $CDCl_3$)

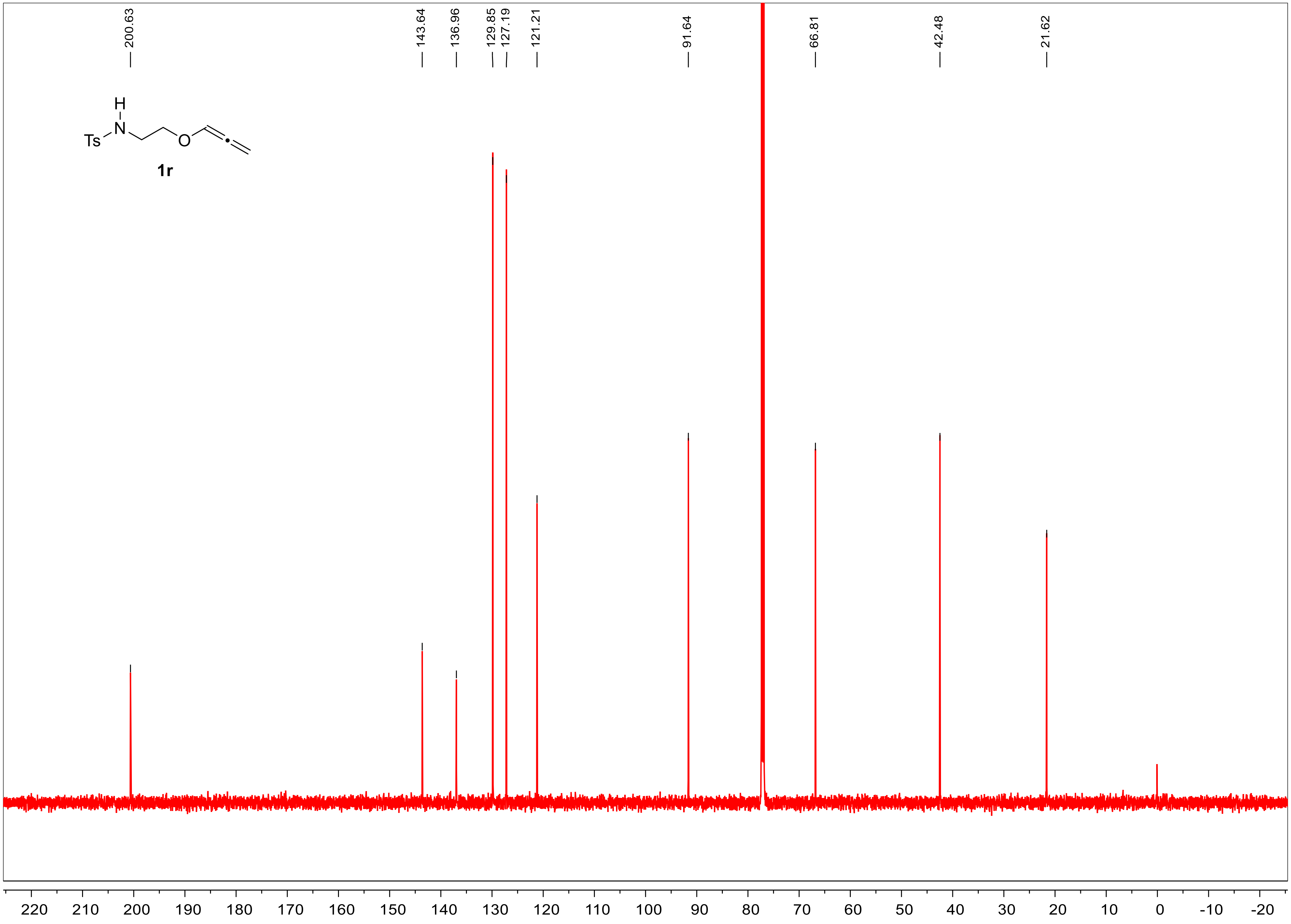

DEPT 135 (150 MHz, $CDCl_3$)

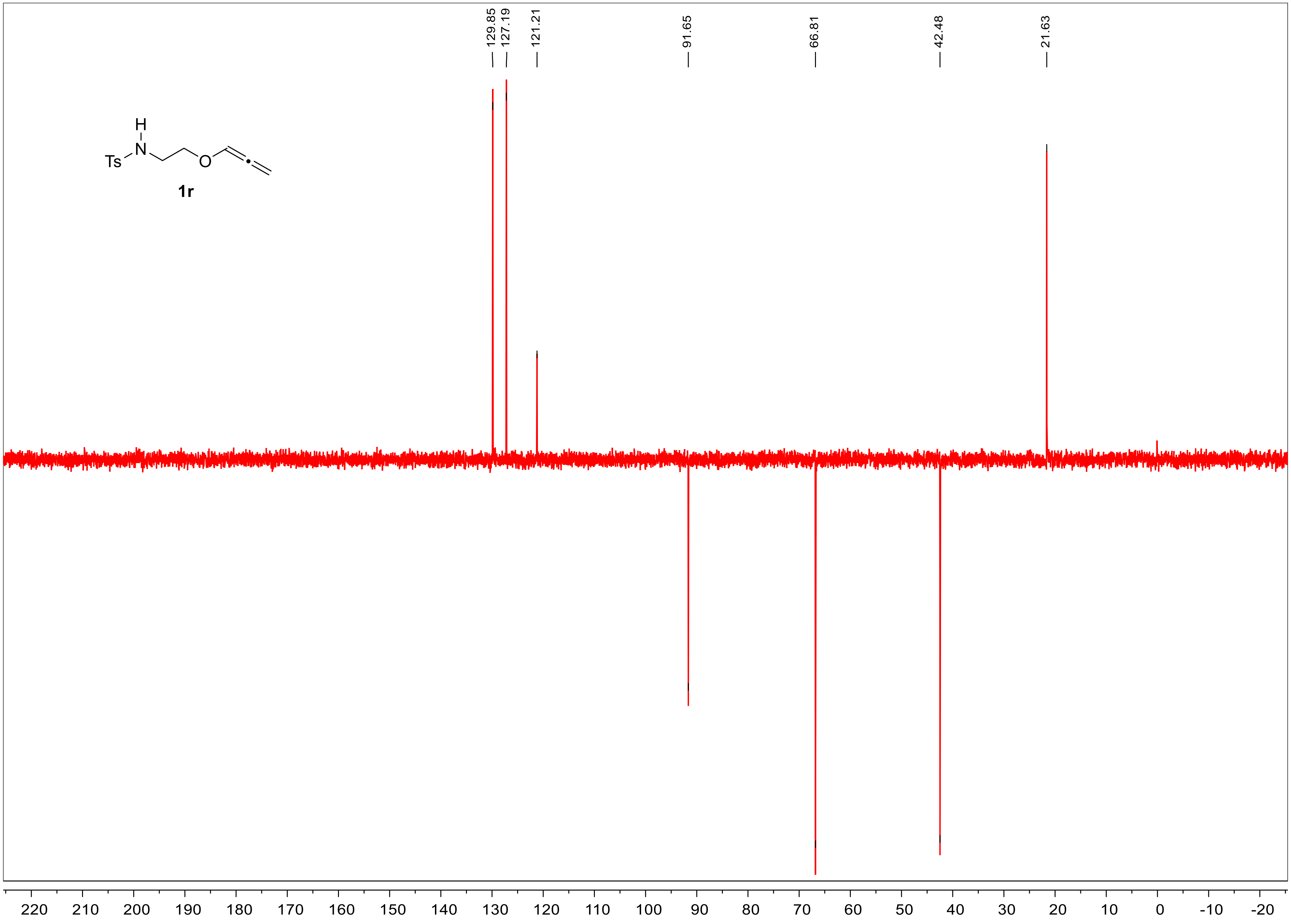

^{1}H-NMR (600 MHz, $CDCl_3$)

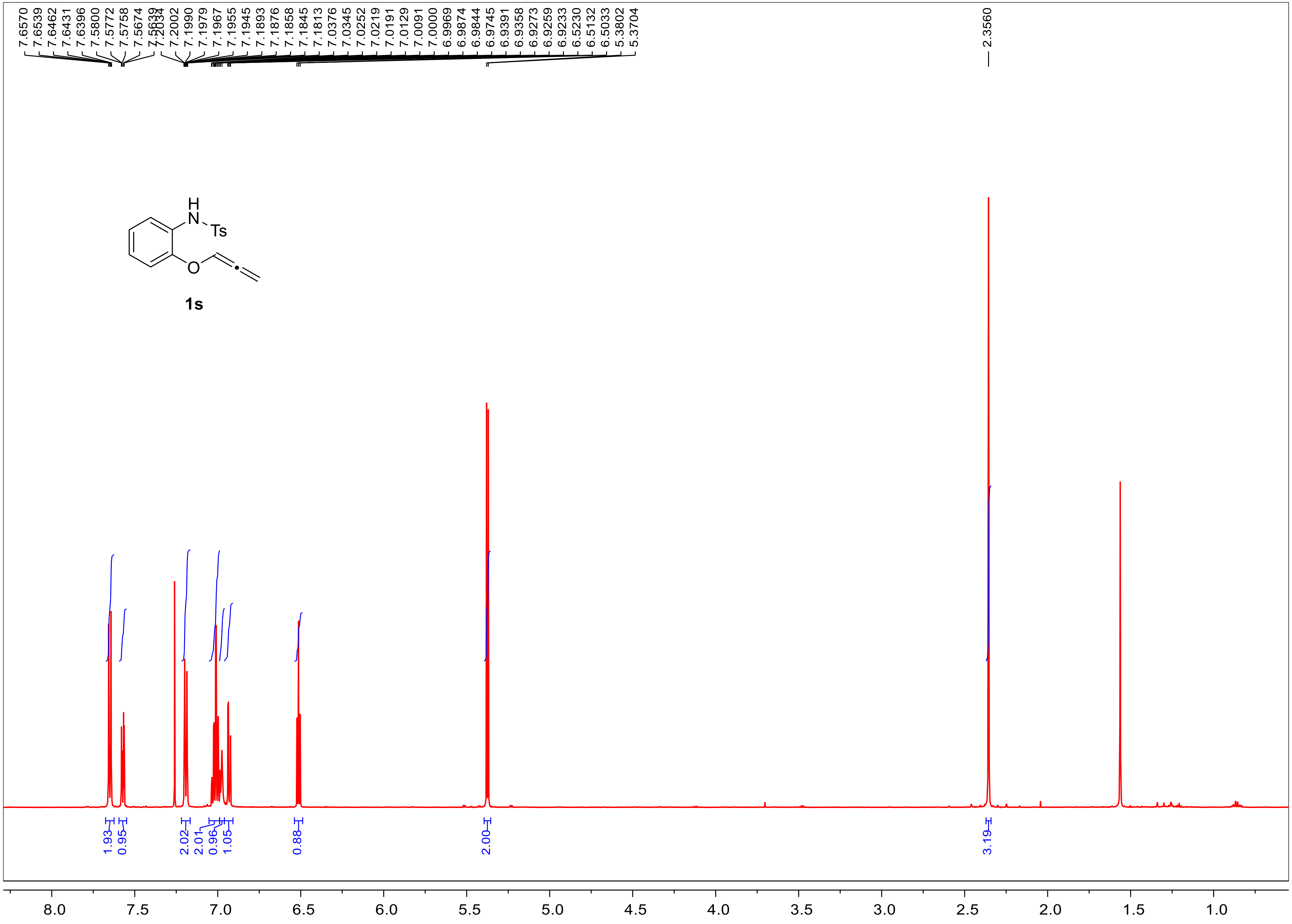

2D - COSY (600 MHz, $CDCl_3$)

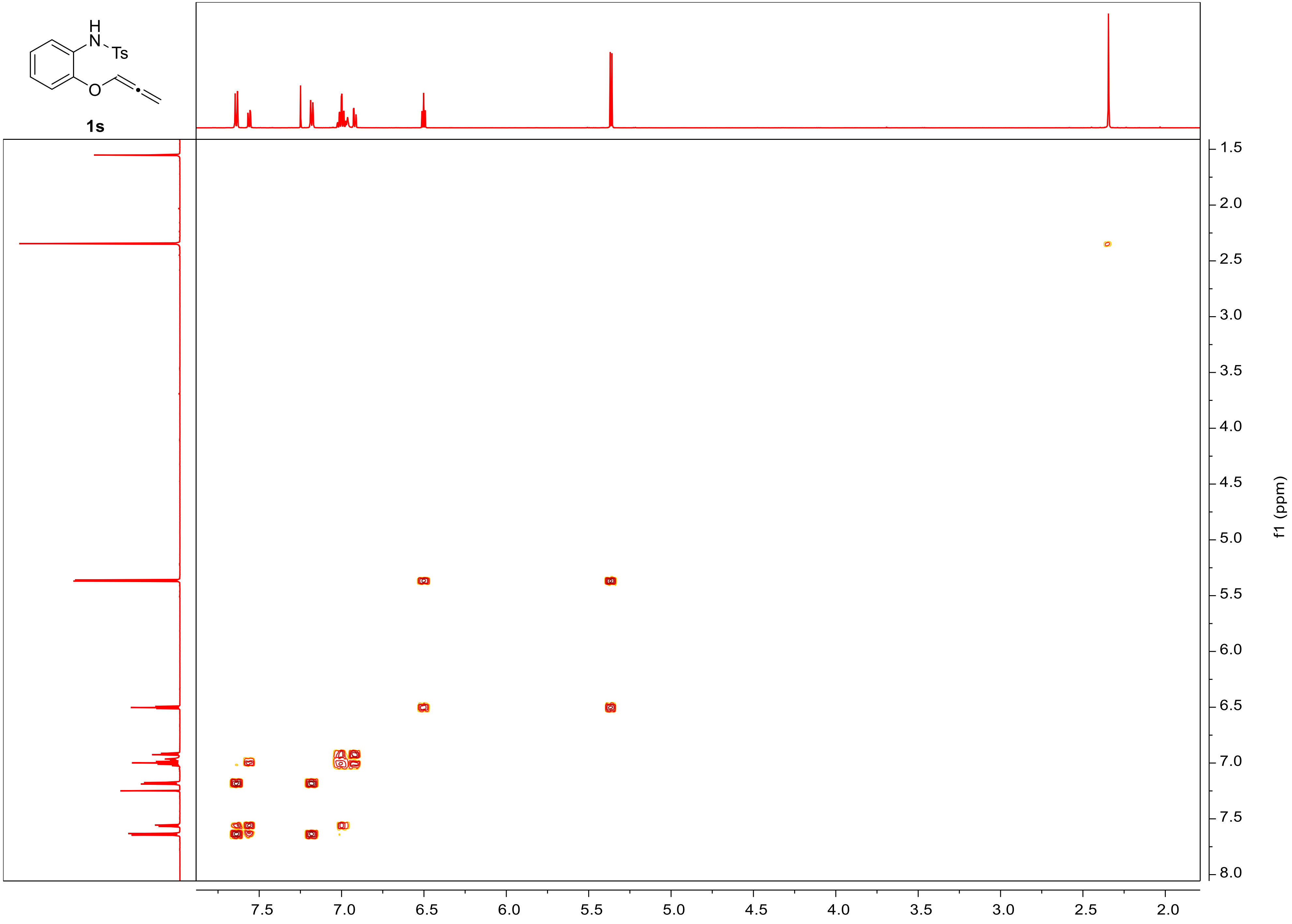

$^{13}C\{^{1}H\}$ NMR (150 MHz, $CDCl_3$)

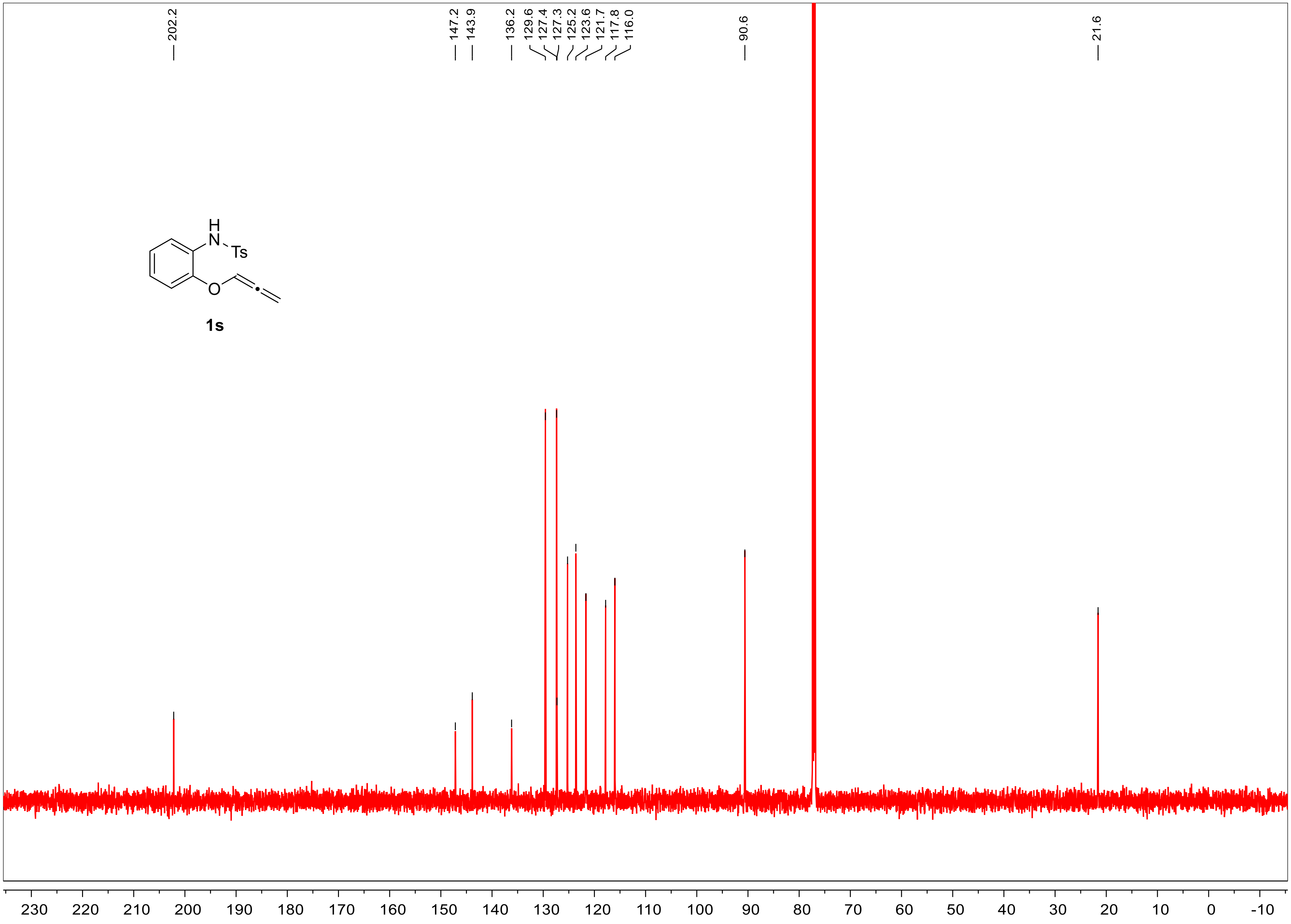

DEPT 135 (150 MHz, $CDCl_3$)

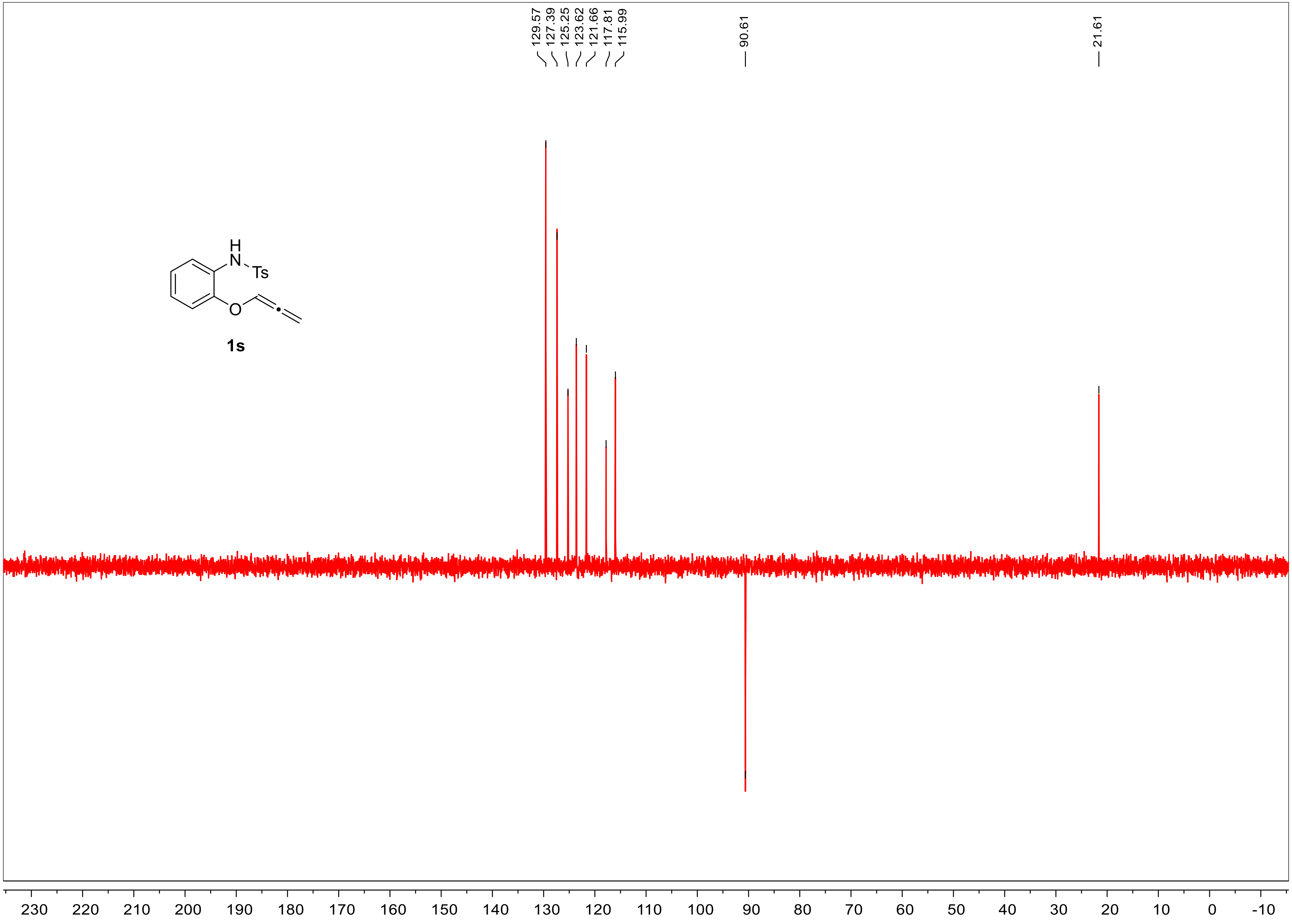

1s

^{1}H-NMR (600 MHz, $CDCl_3$)

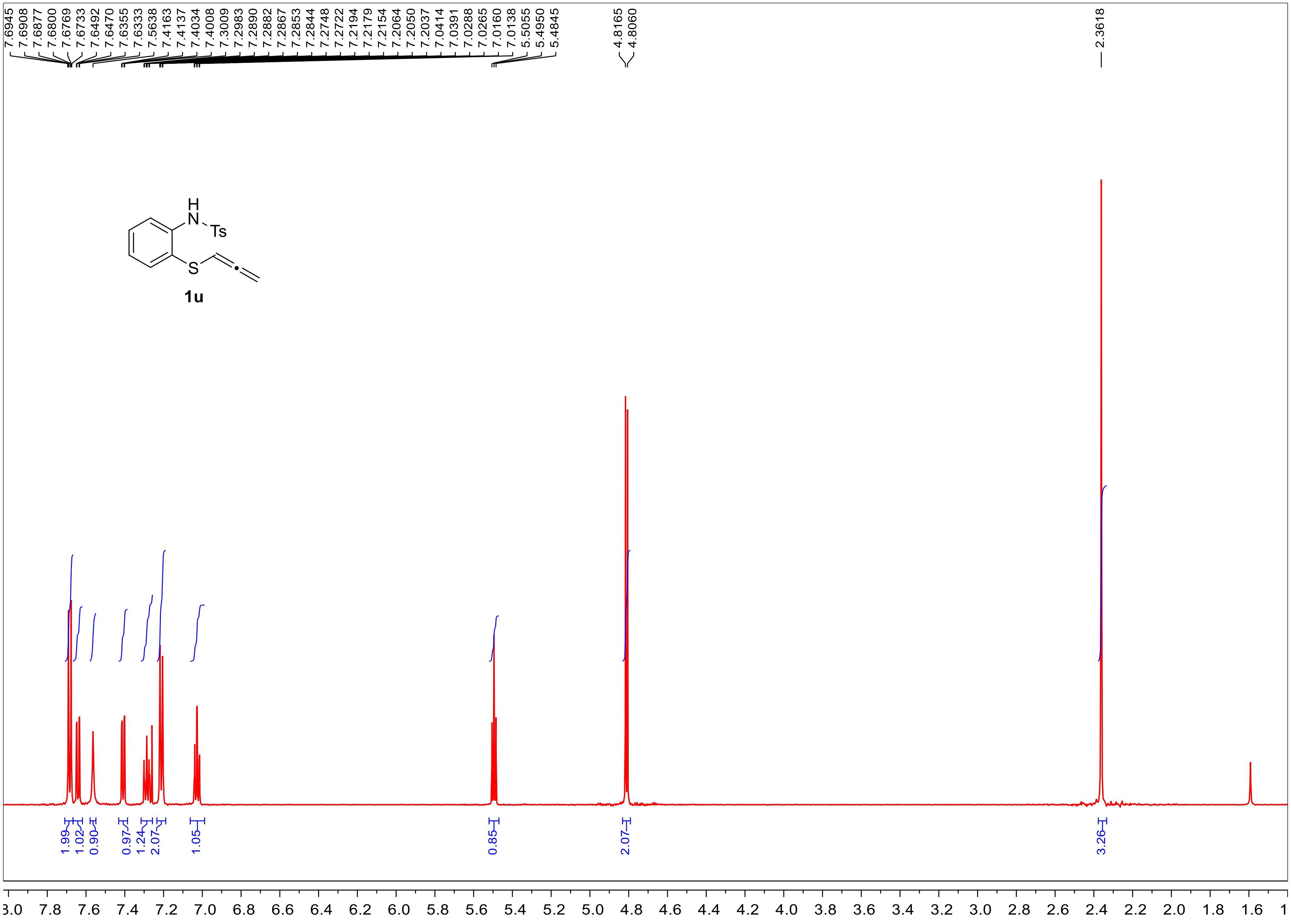

2D - COSY (600 MHz, $CDCl_3$)

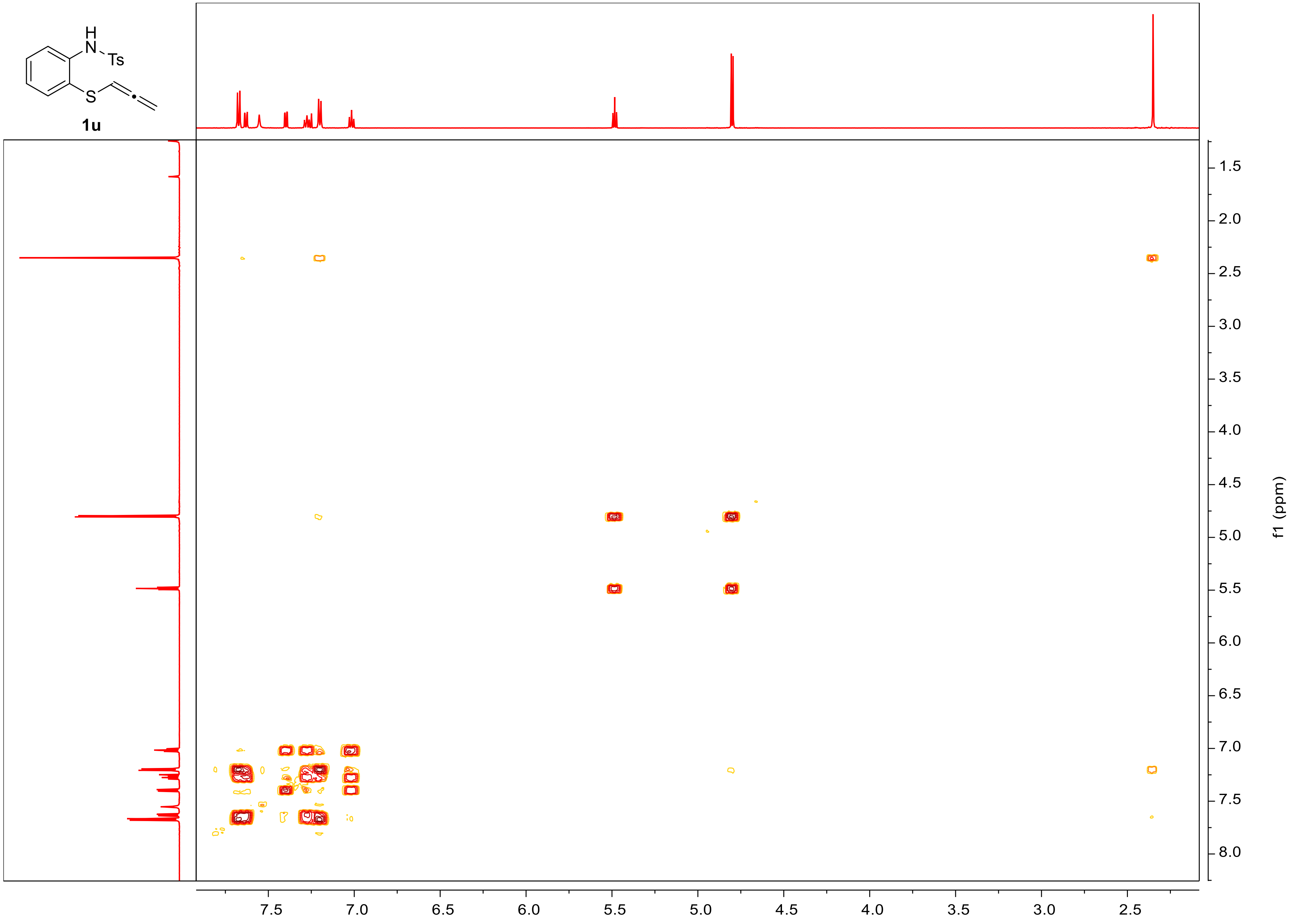

$^{13}C\{^{1}H\}$ NMR (150 MHz, $CDCl_3$)

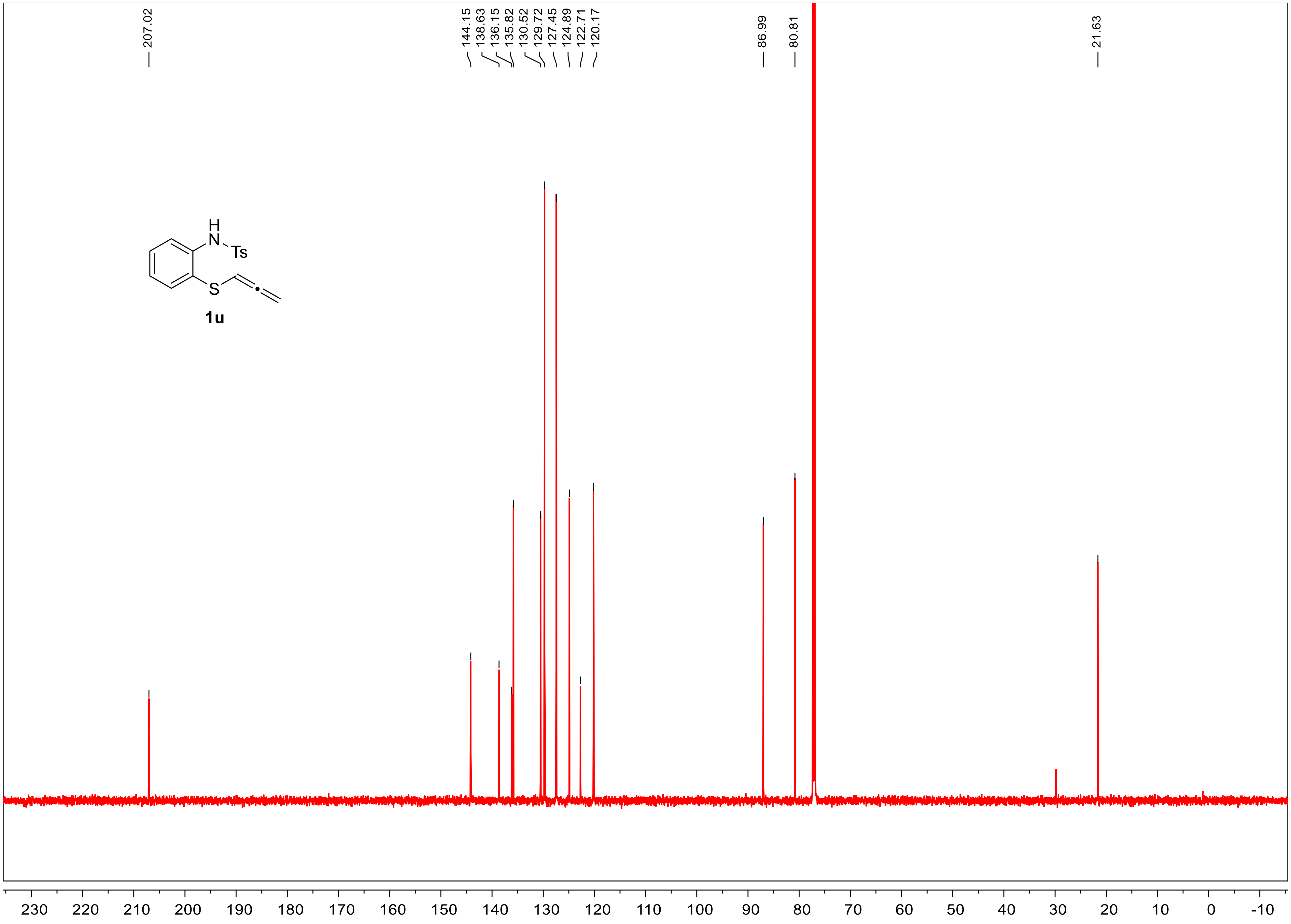

DEPT 135 (150 MHz, $CDCl_3$)

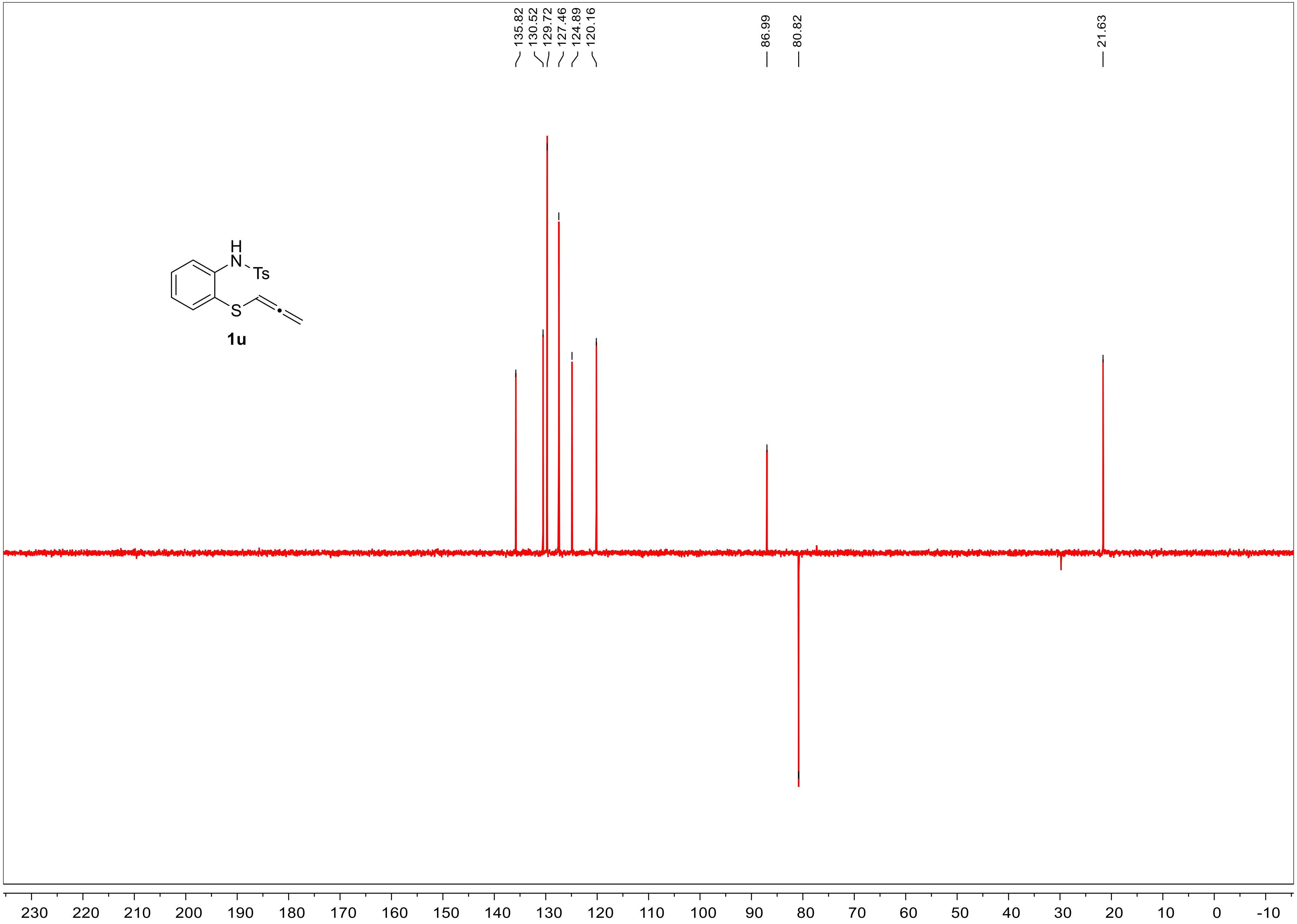

¹H-NMR (600 MHz, $CDCl_3$)

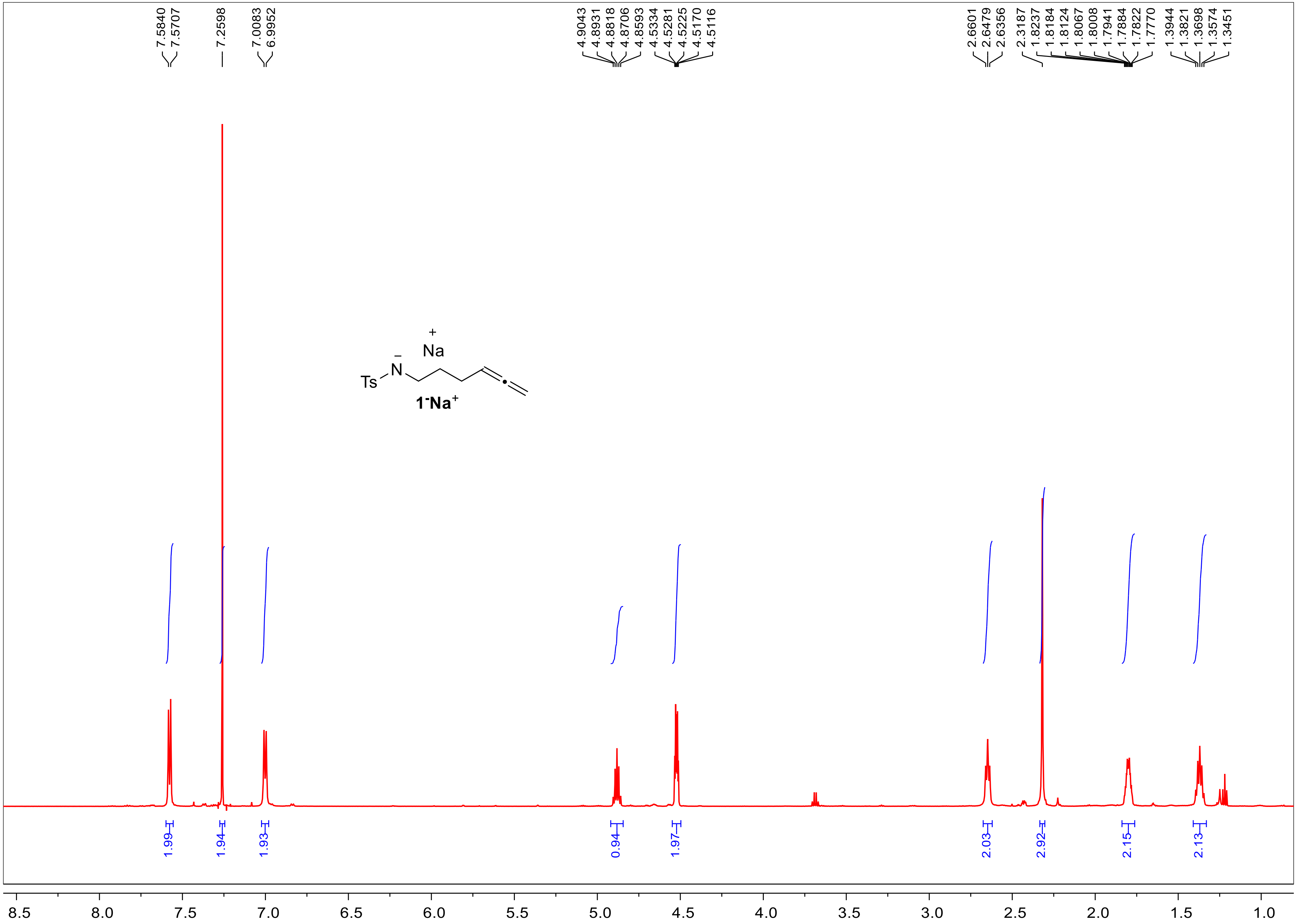

^{1}H-NMR (600 MHz, DMSO-d^6)

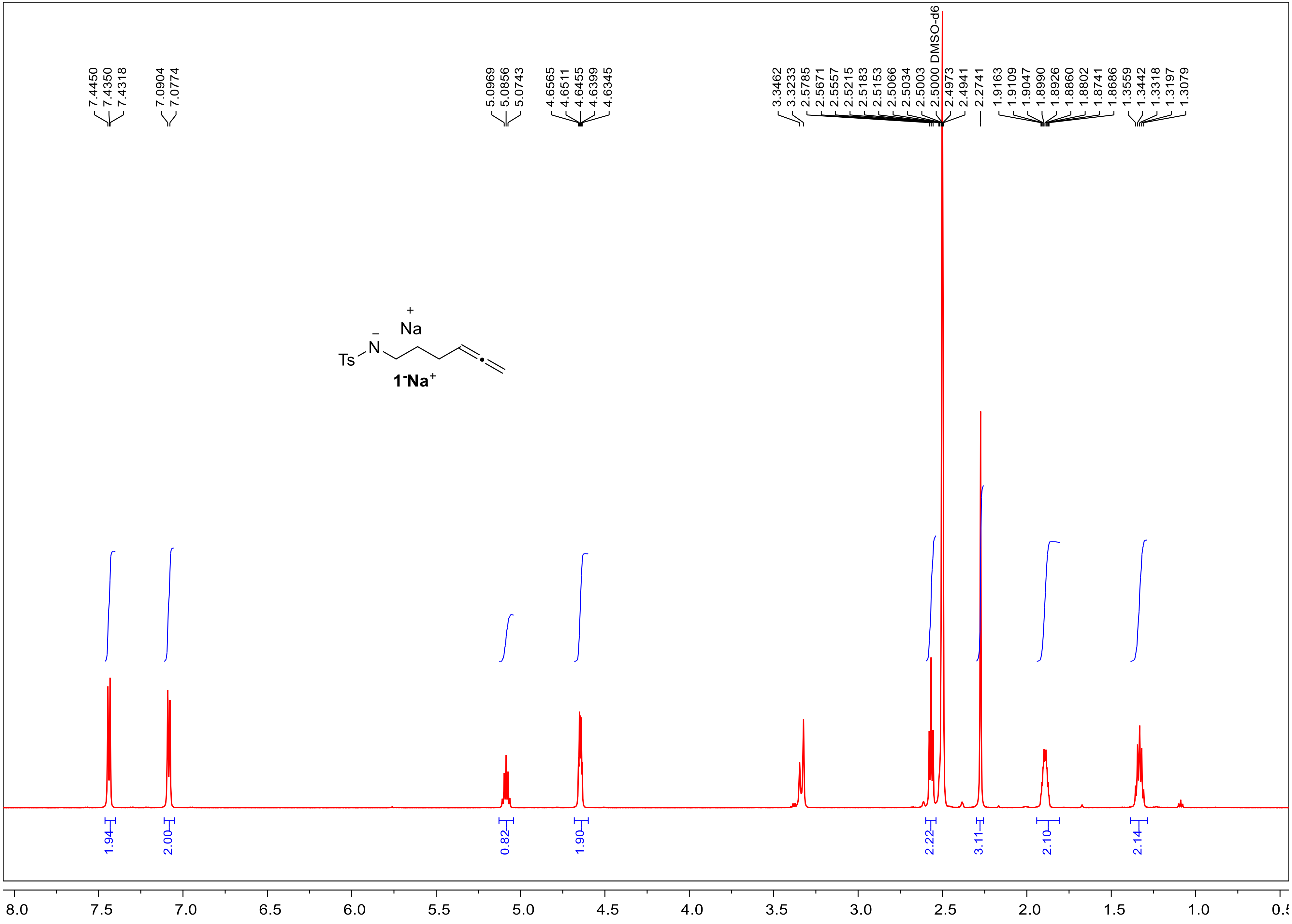

2D - COSY (600 MHz, DMSO-d^6)

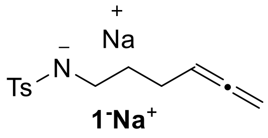

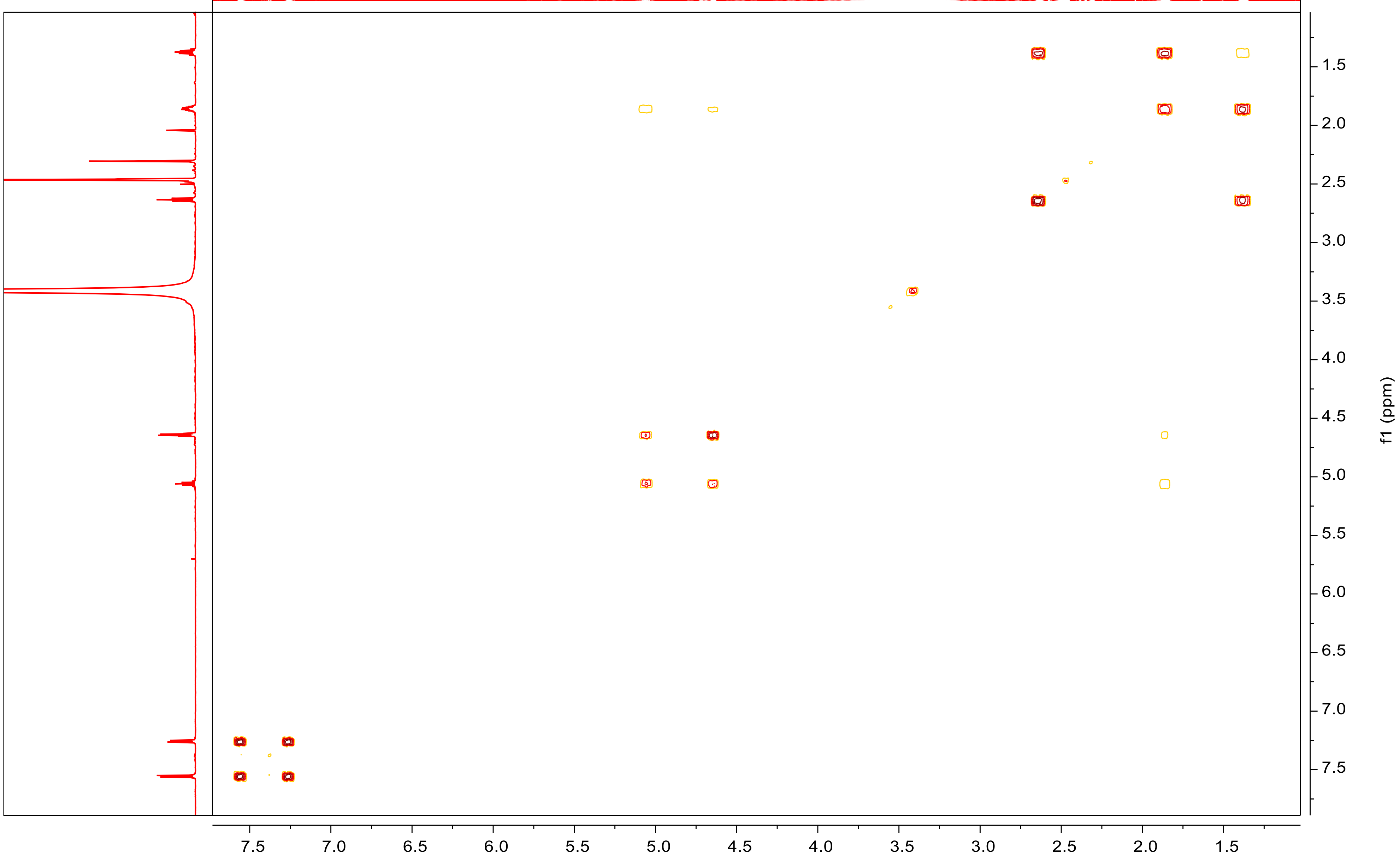

$^{13}C\{^1H\}$ NMR (150 MHz, DMSO-d^6)

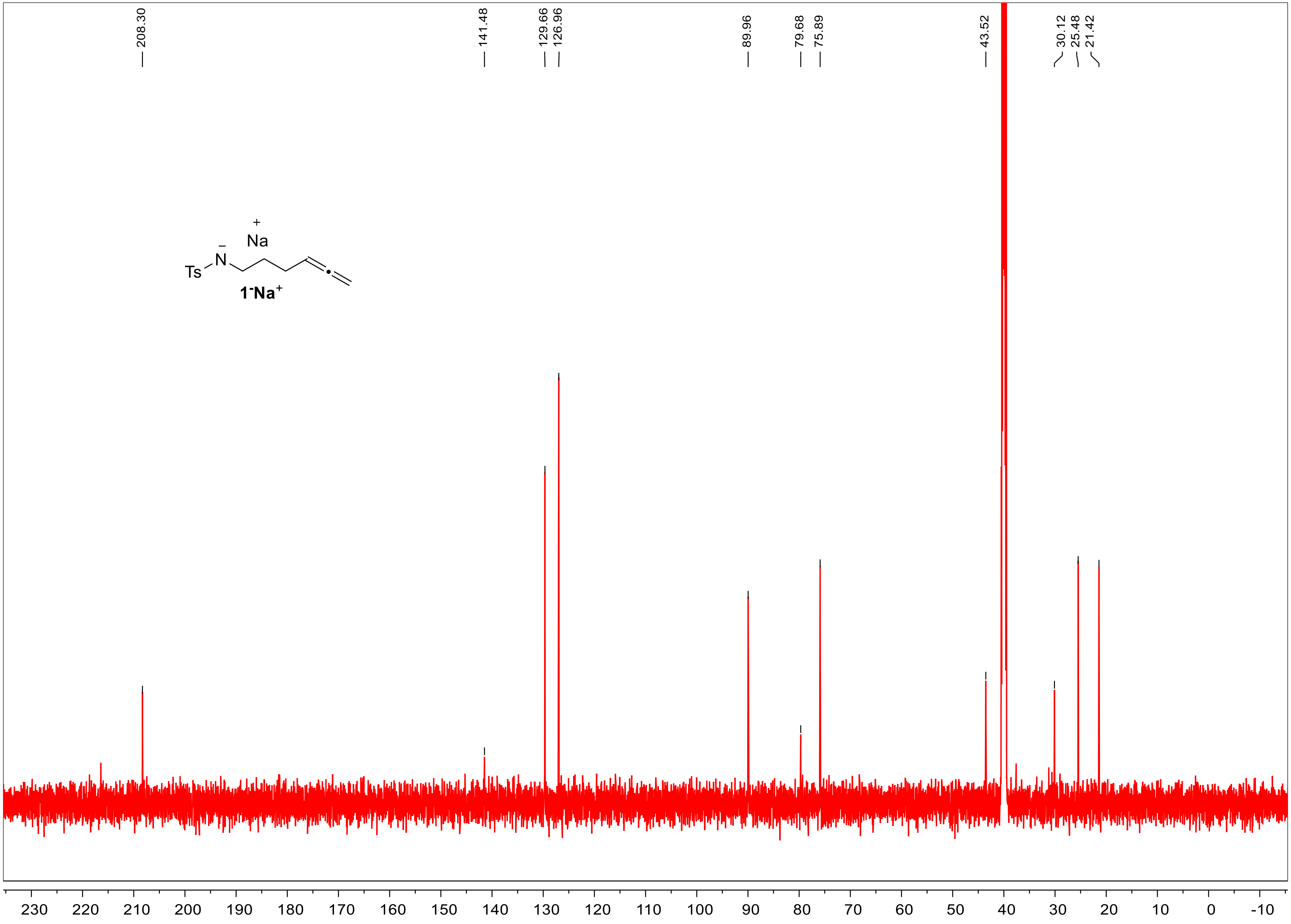

DEPT 135 (150 MHz, DMSO-d^6)

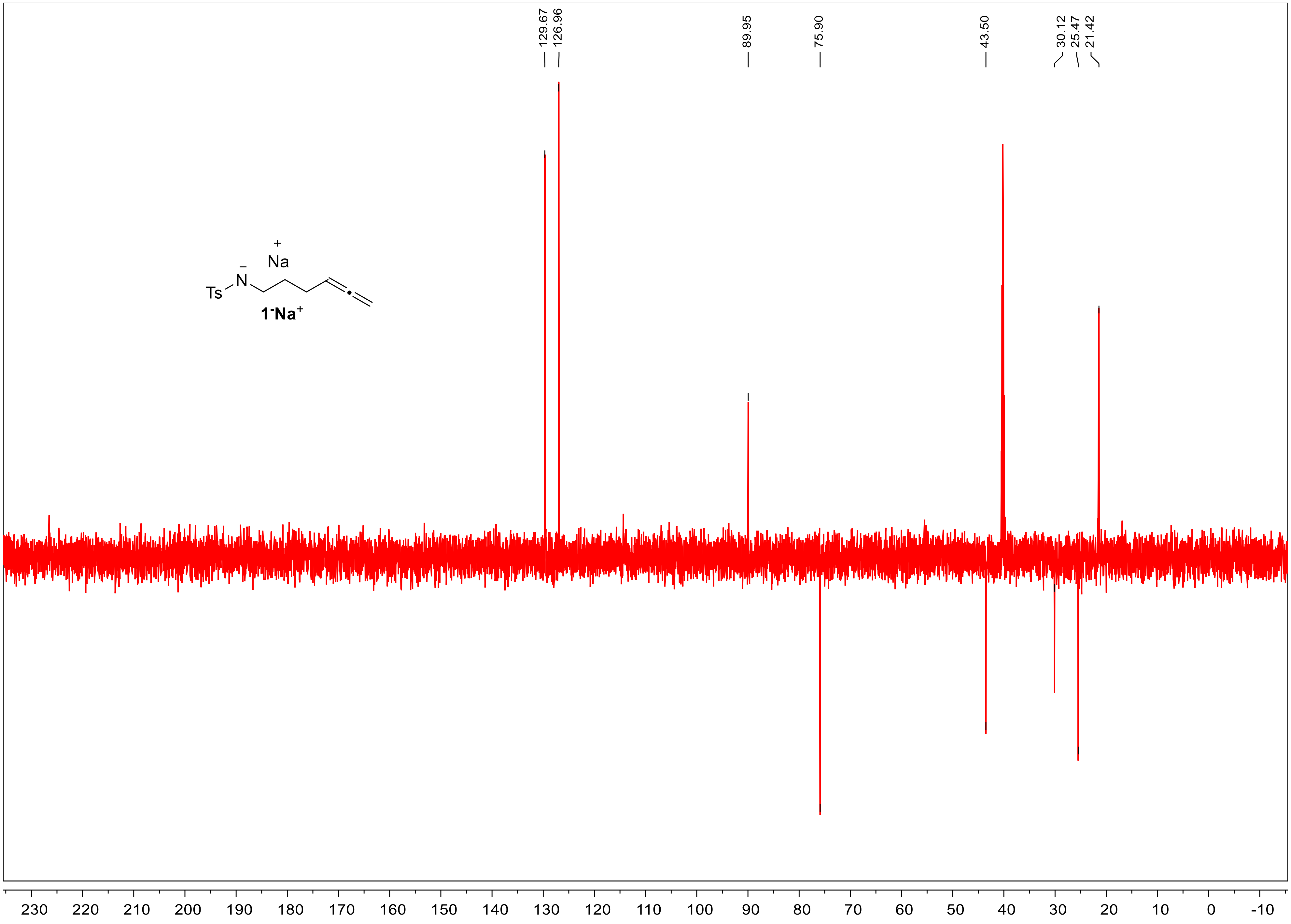

¹H-NMR (600 MHz, $CDCl_3$)

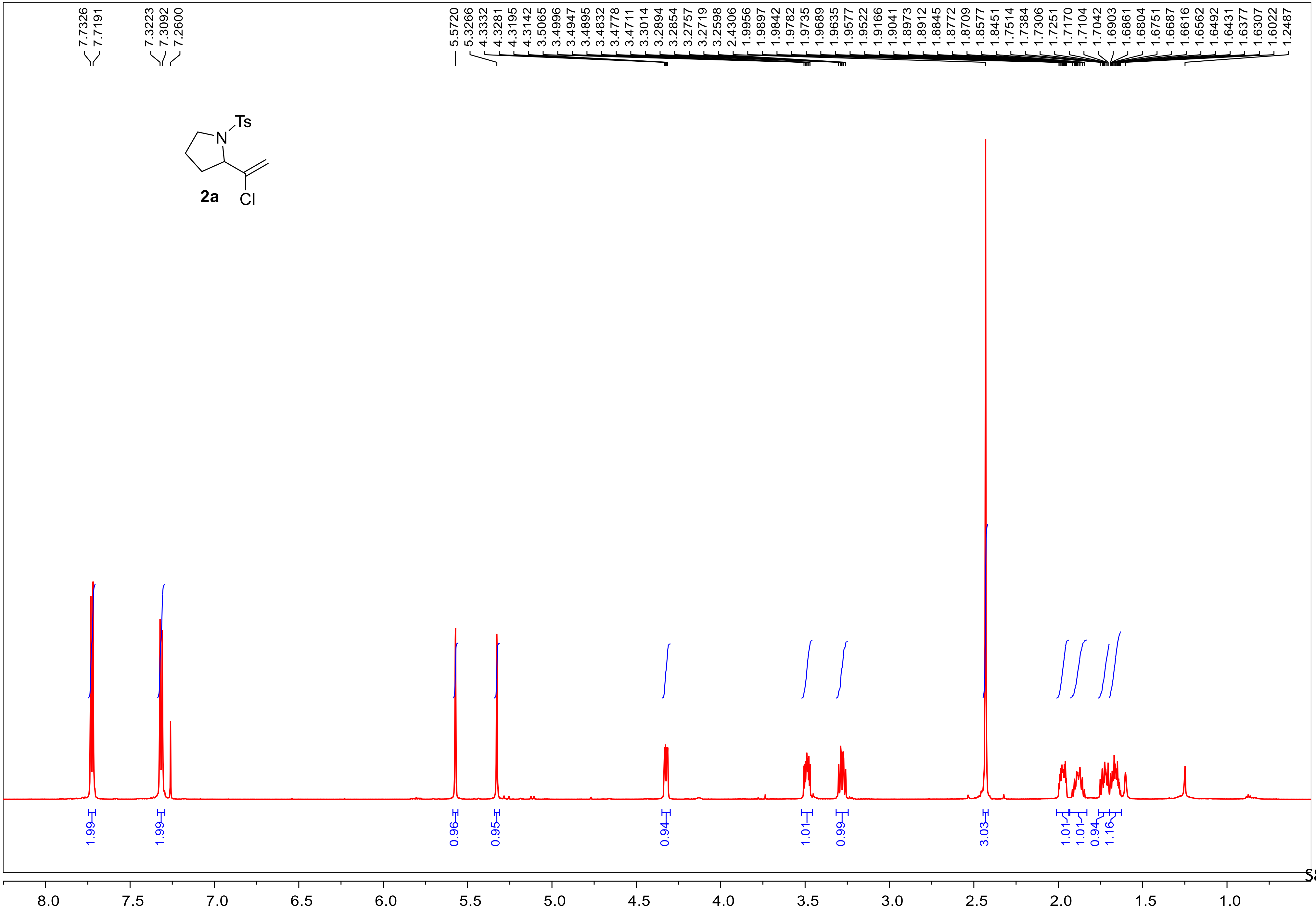

2D - COSY (600 MHz, $CDCl_3$)

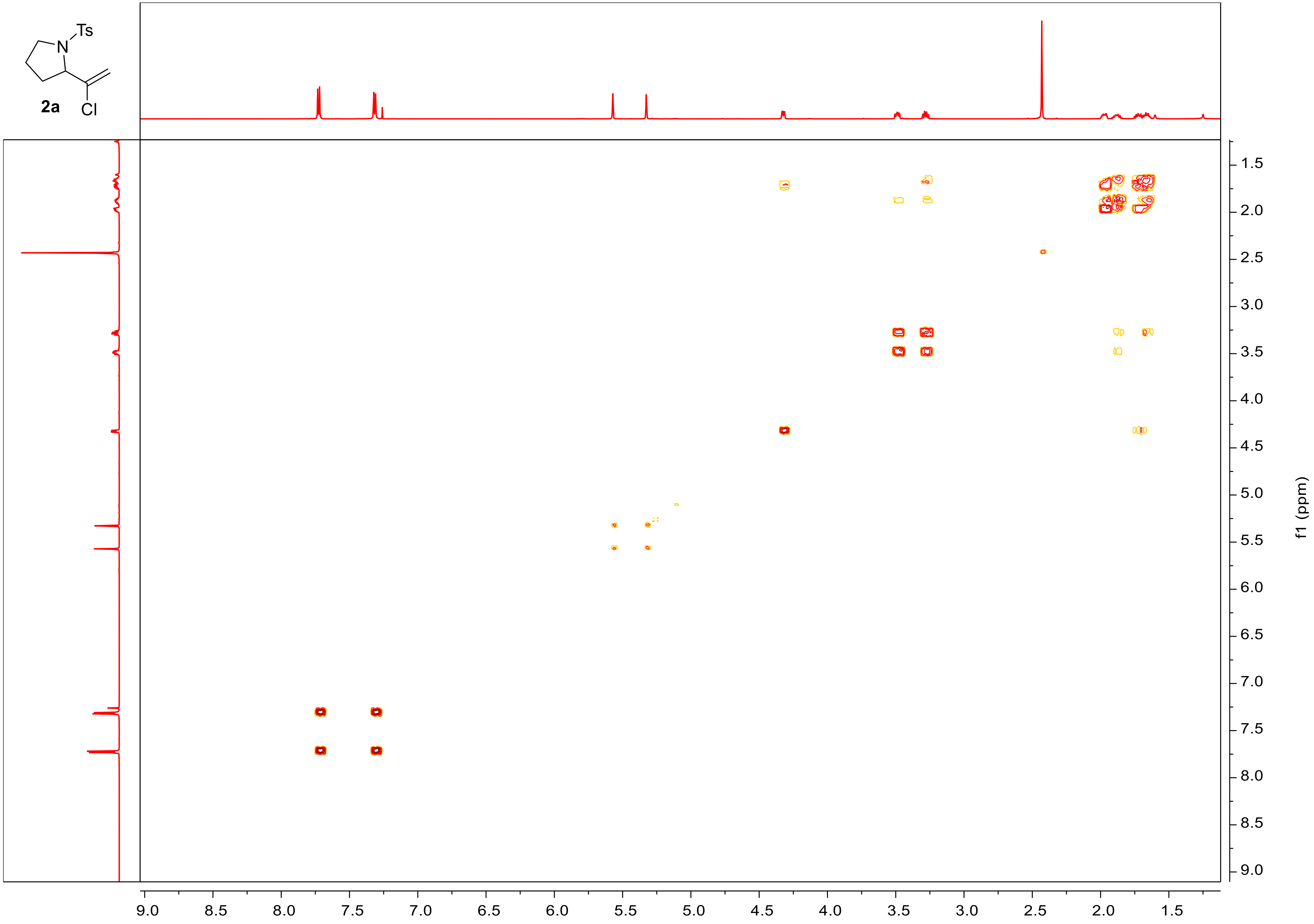

$^{13}C\{^{1}H\}$ NMR (150 MHz, $CDCl_3$)

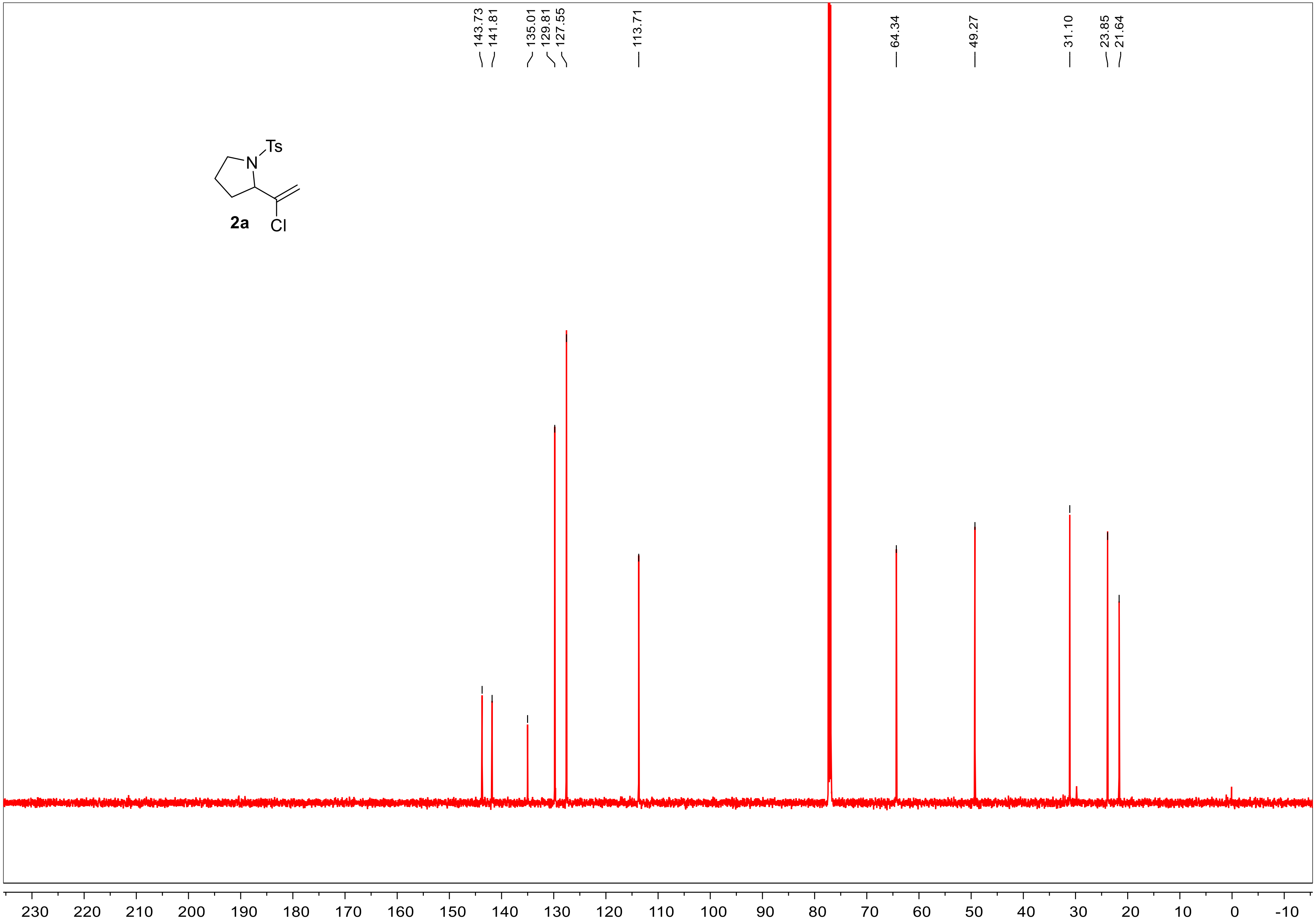

DEPT 135 (150 MHz, $CDCl_3$)

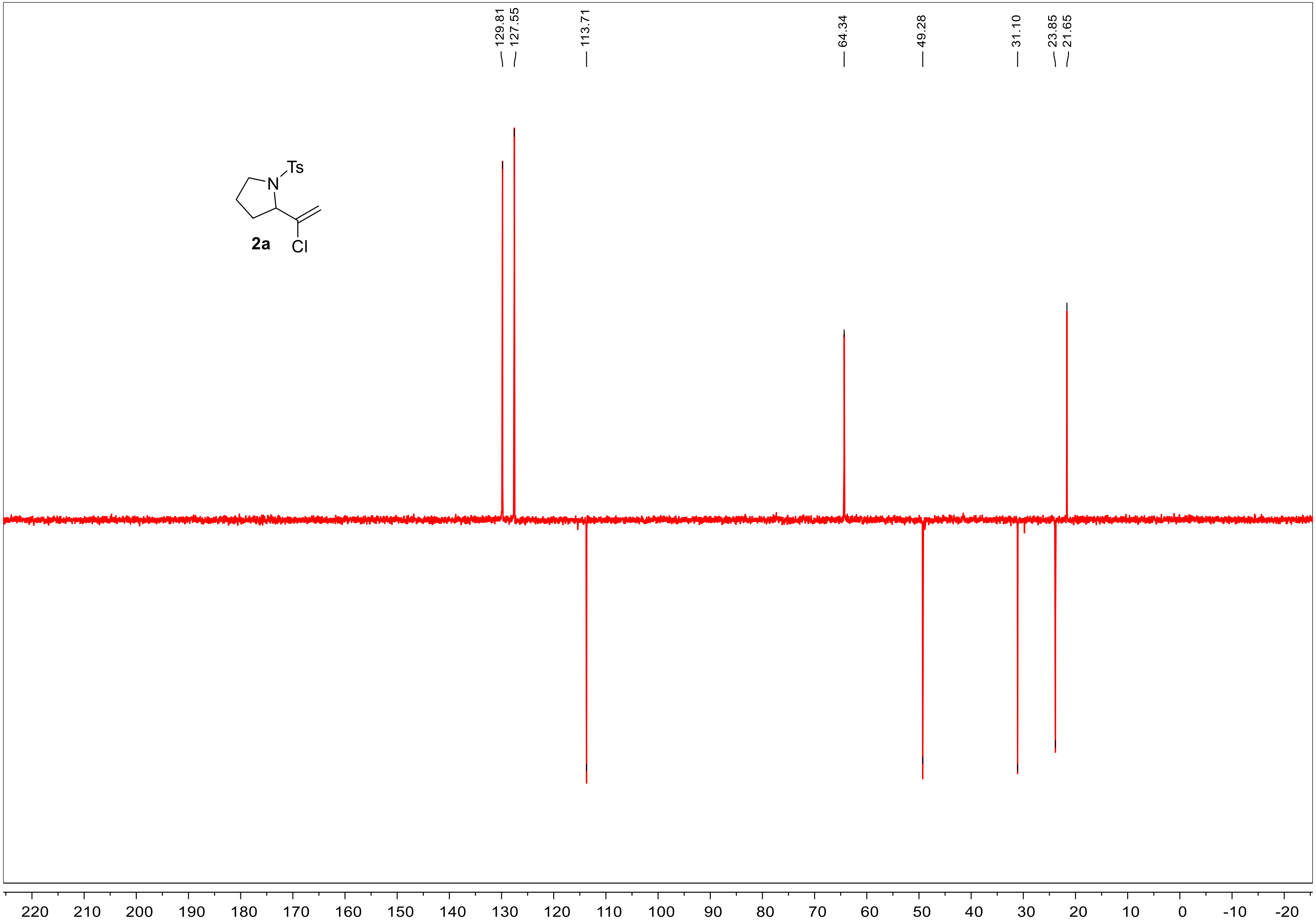

^{1}H-NMR (600 MHz, $CDCl_3$)

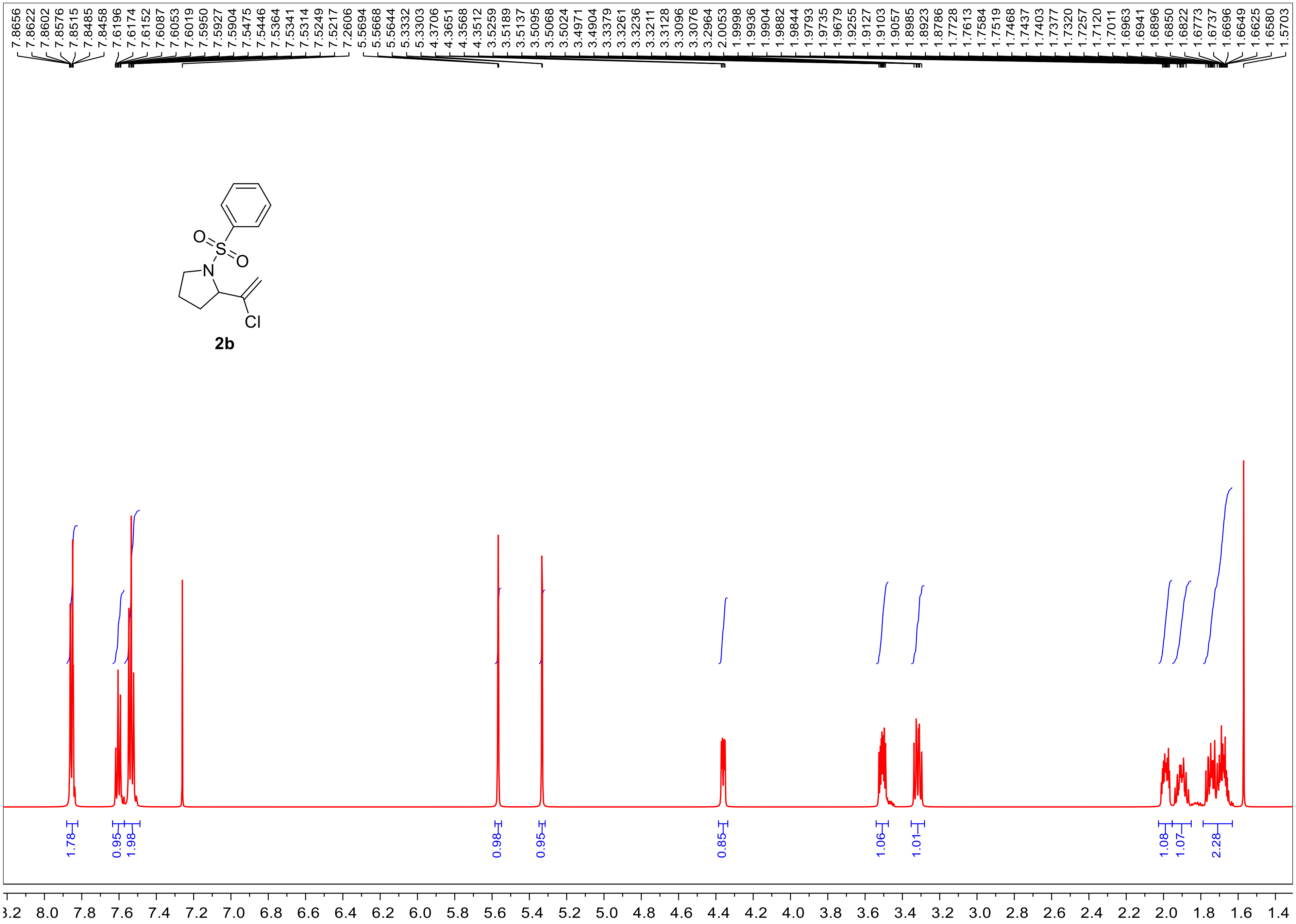

2D - COSY (600 MHz, $CDCl_3$)

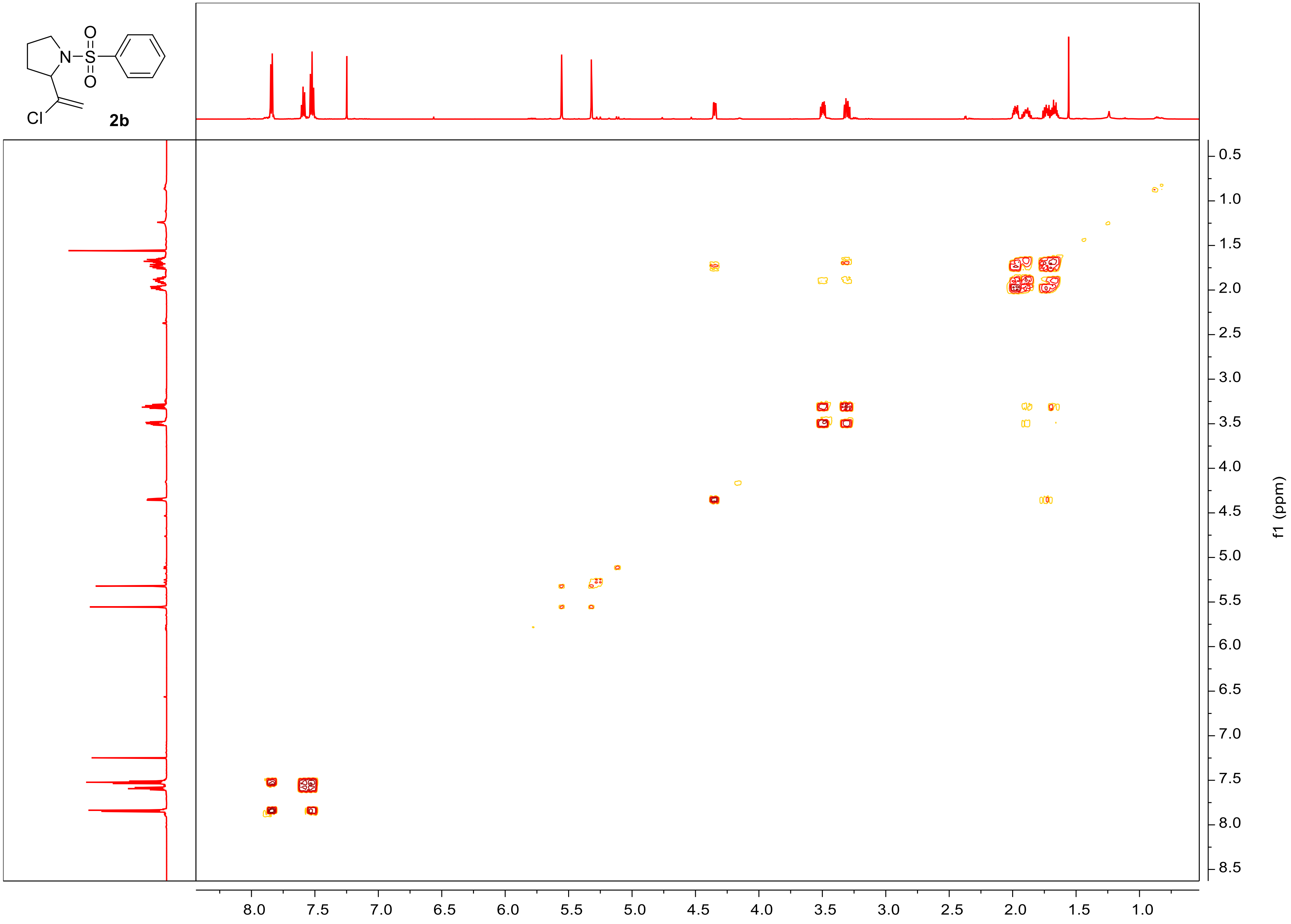

^{13}C{^{1}H} NMR (150 MHz, $CDCl_3$)

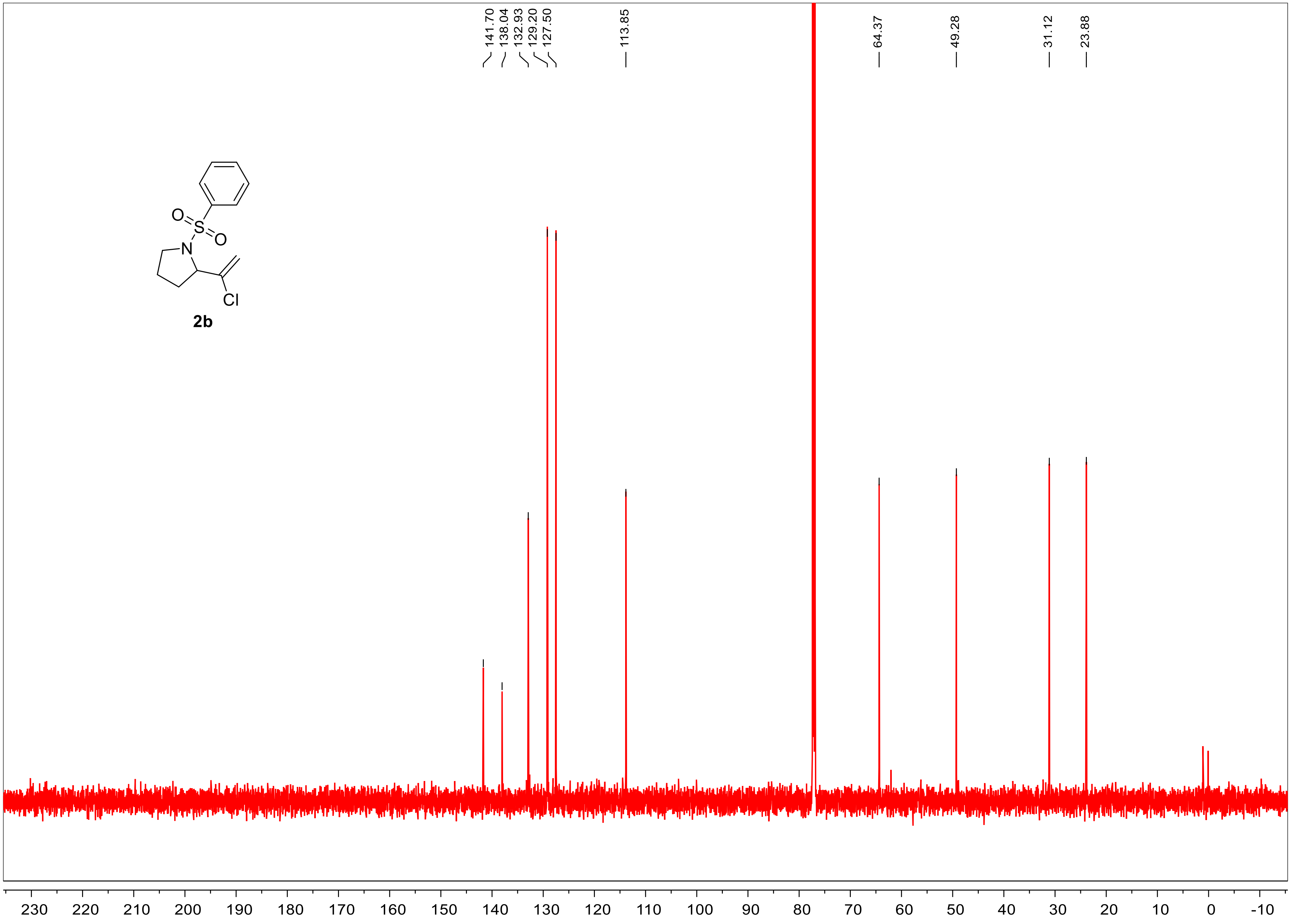

DEPT 135 (150 MHz, $CDCl_3$)

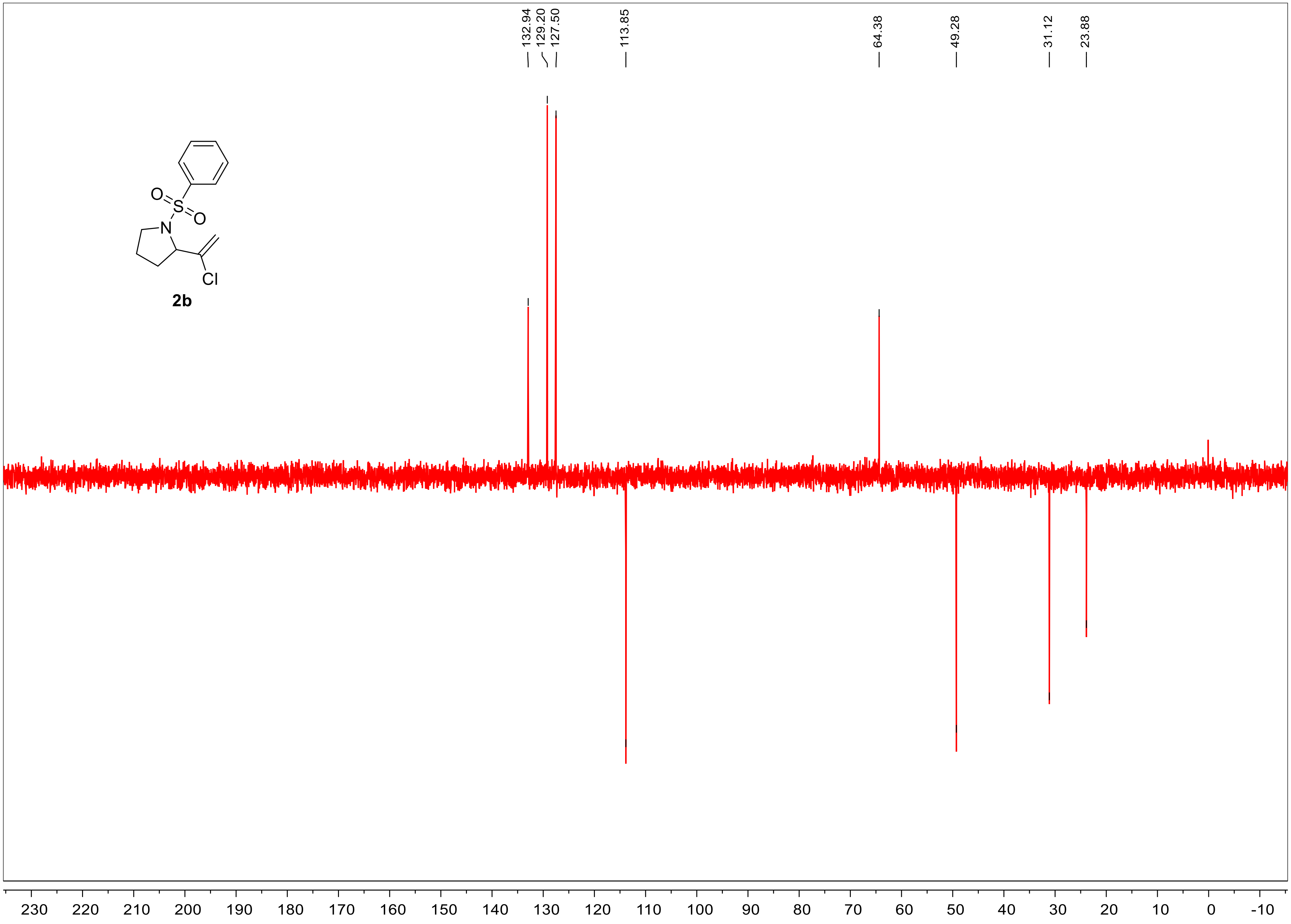

^{1}H-NMR (600 MHz, $CDCl_3$)

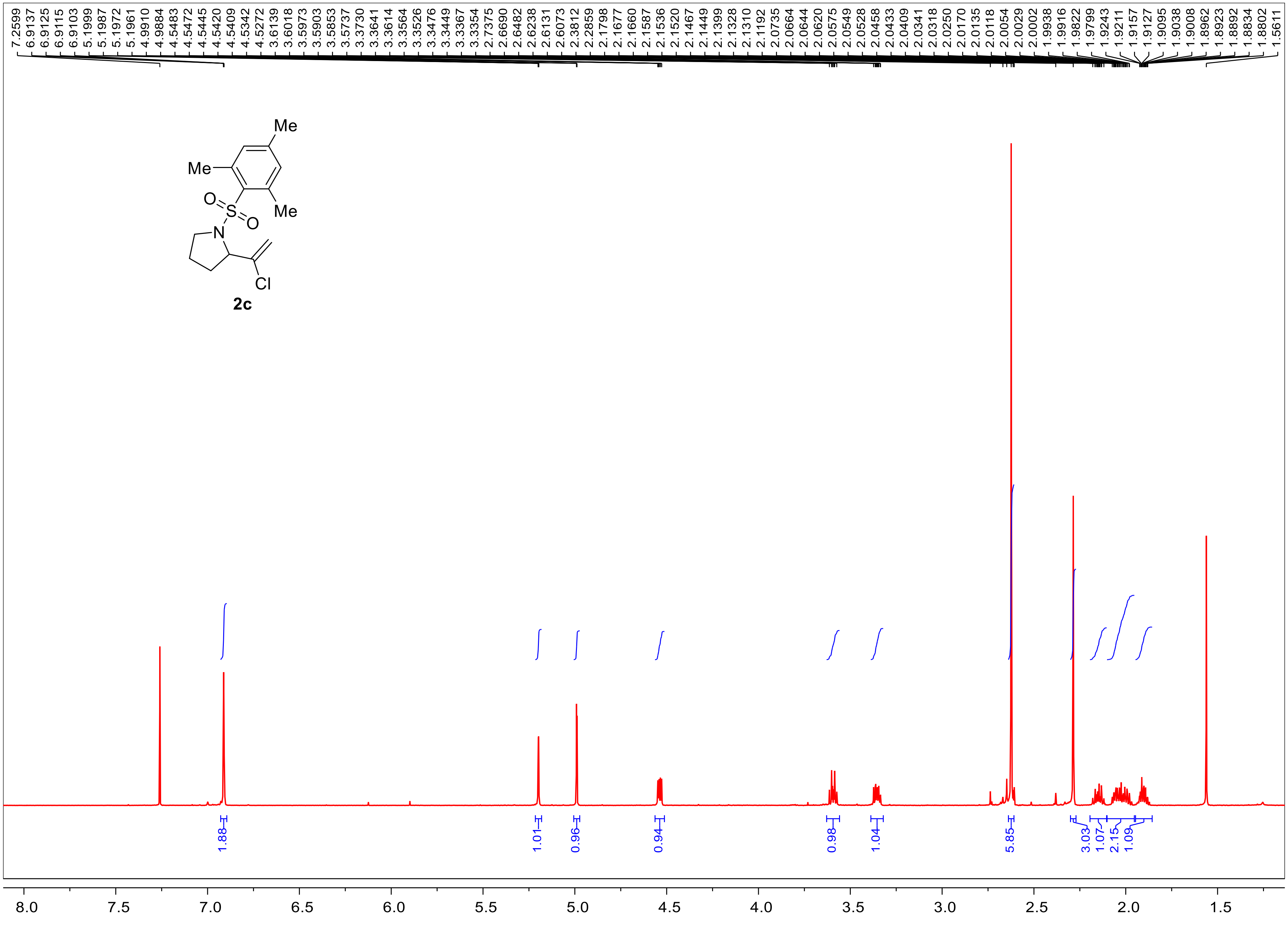

2D - COSY (600 MHz, $CDCl_3$)

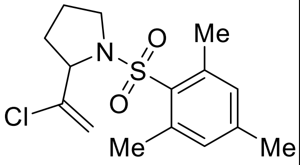

2c

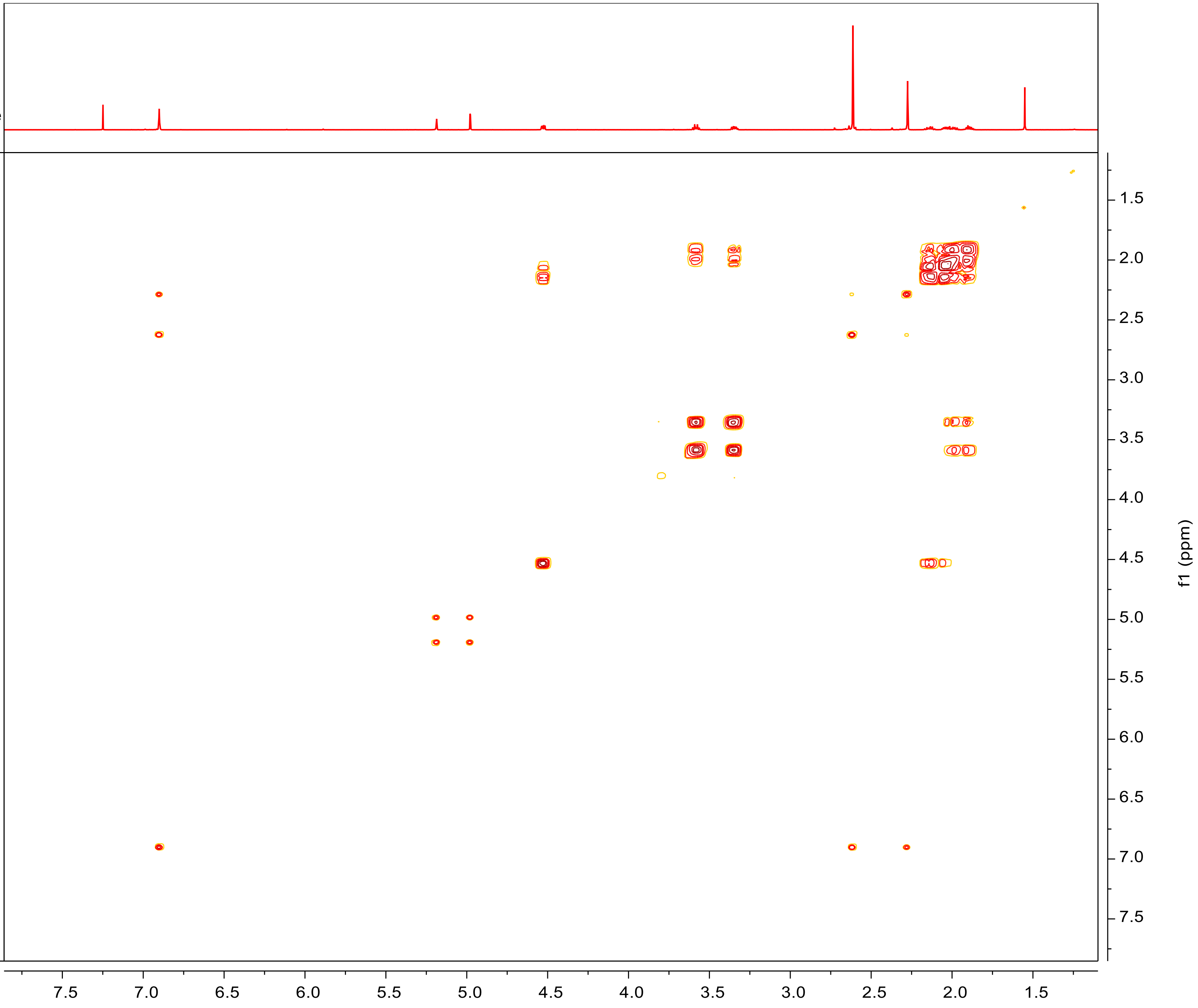

^{13}C{^{1}H} NMR (150 MHz, $CDCl_3$)

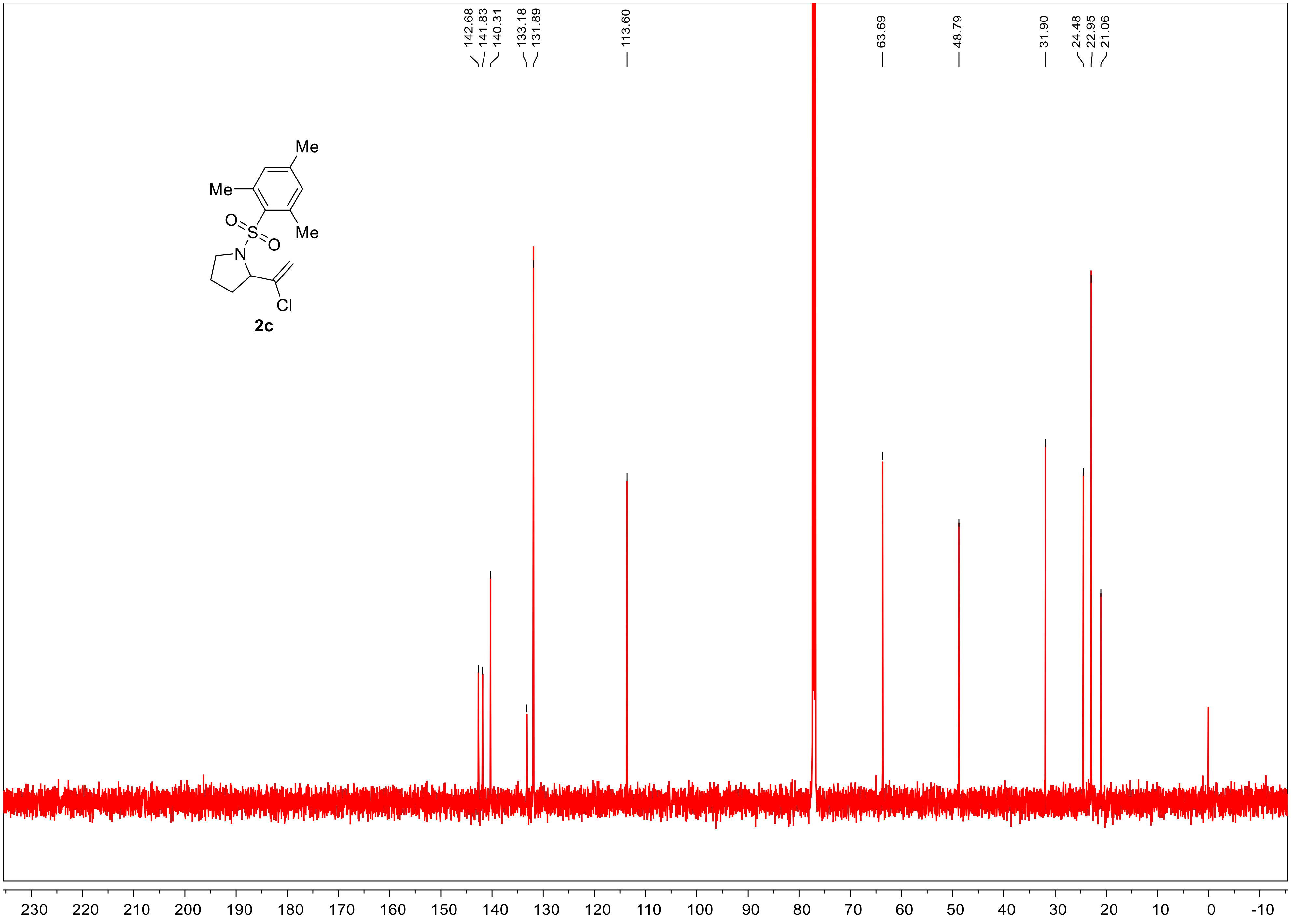

DEPT 135 (150 MHz, $CDCl_3$)

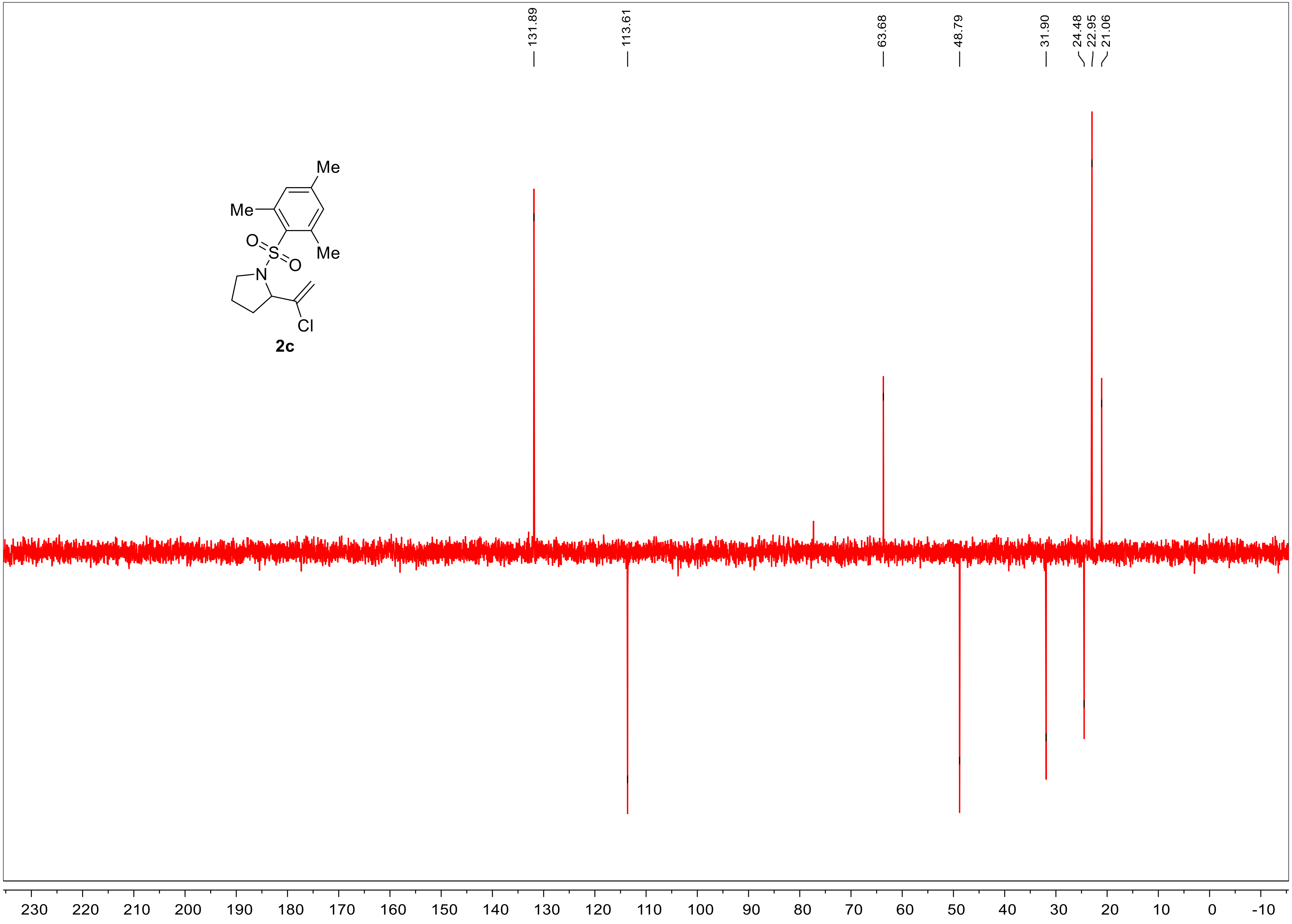

^{1}H-NMR (600 MHz, $CDCl_3$)

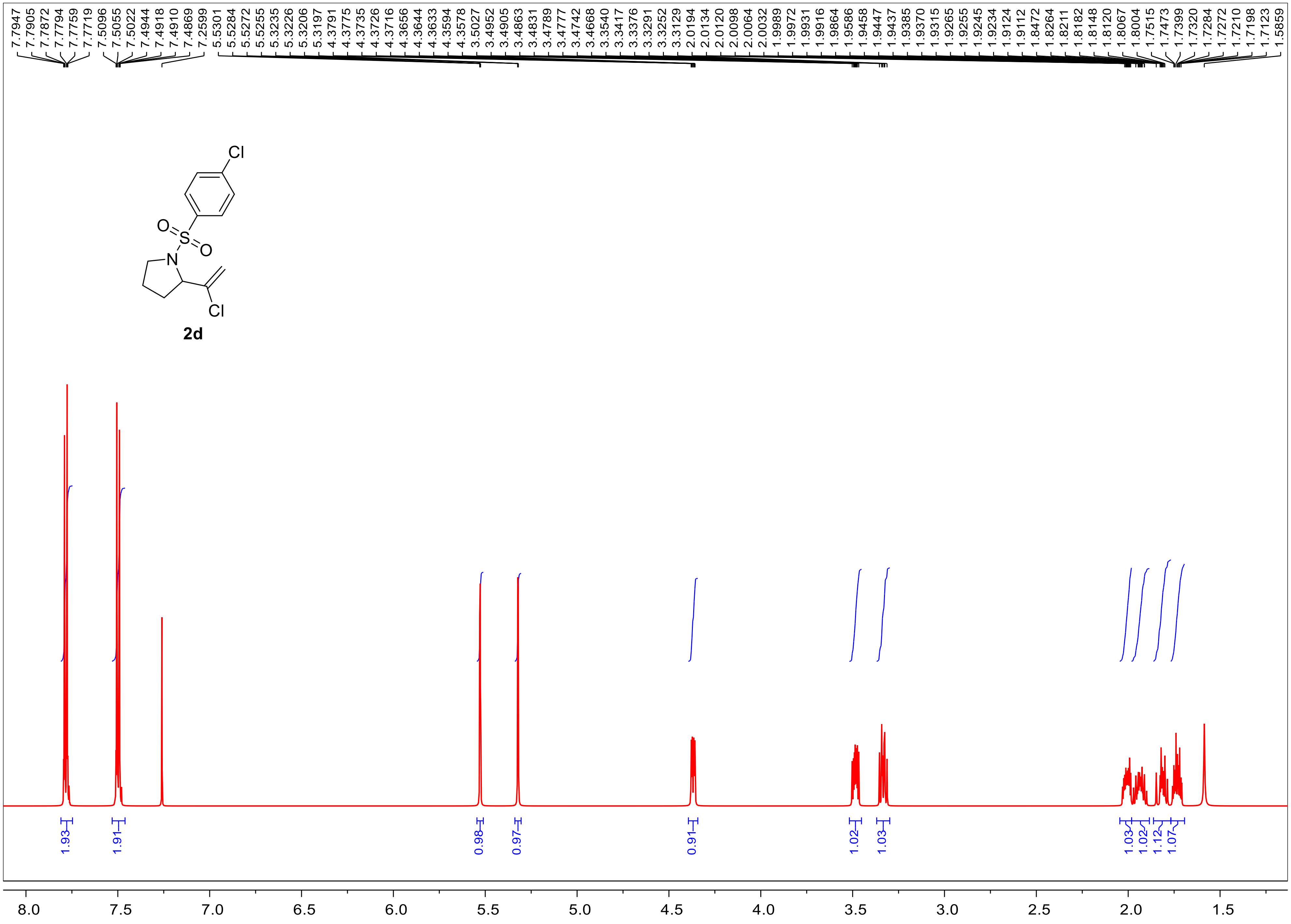

2D - COSY (600 MHz, $CDCl_3$)

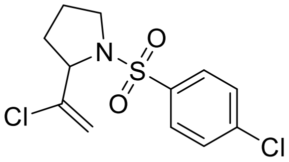

2d

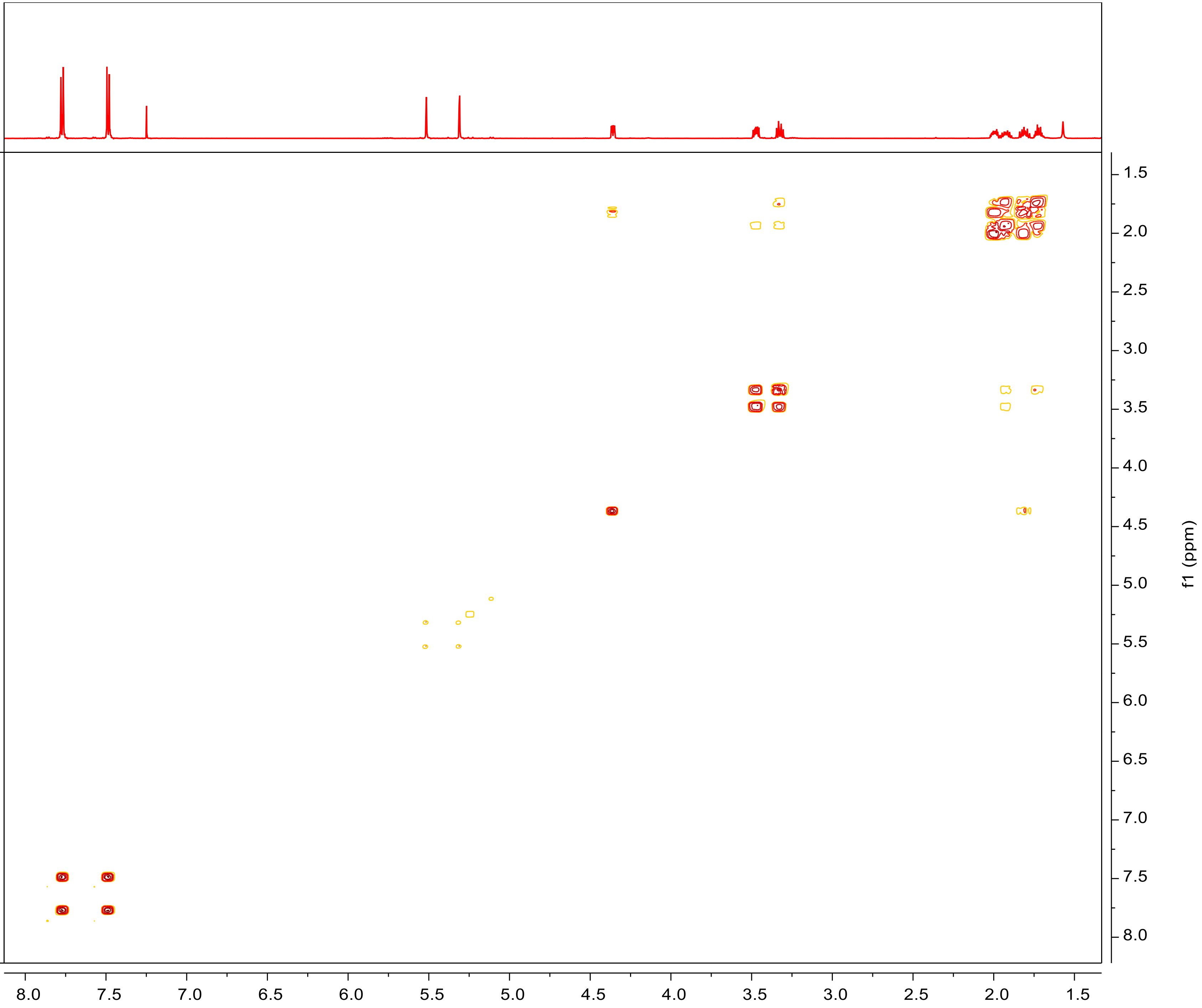

$^{13}C\{^{1}H\}$ **NMR** (150 MHz, $CDCl_3$)

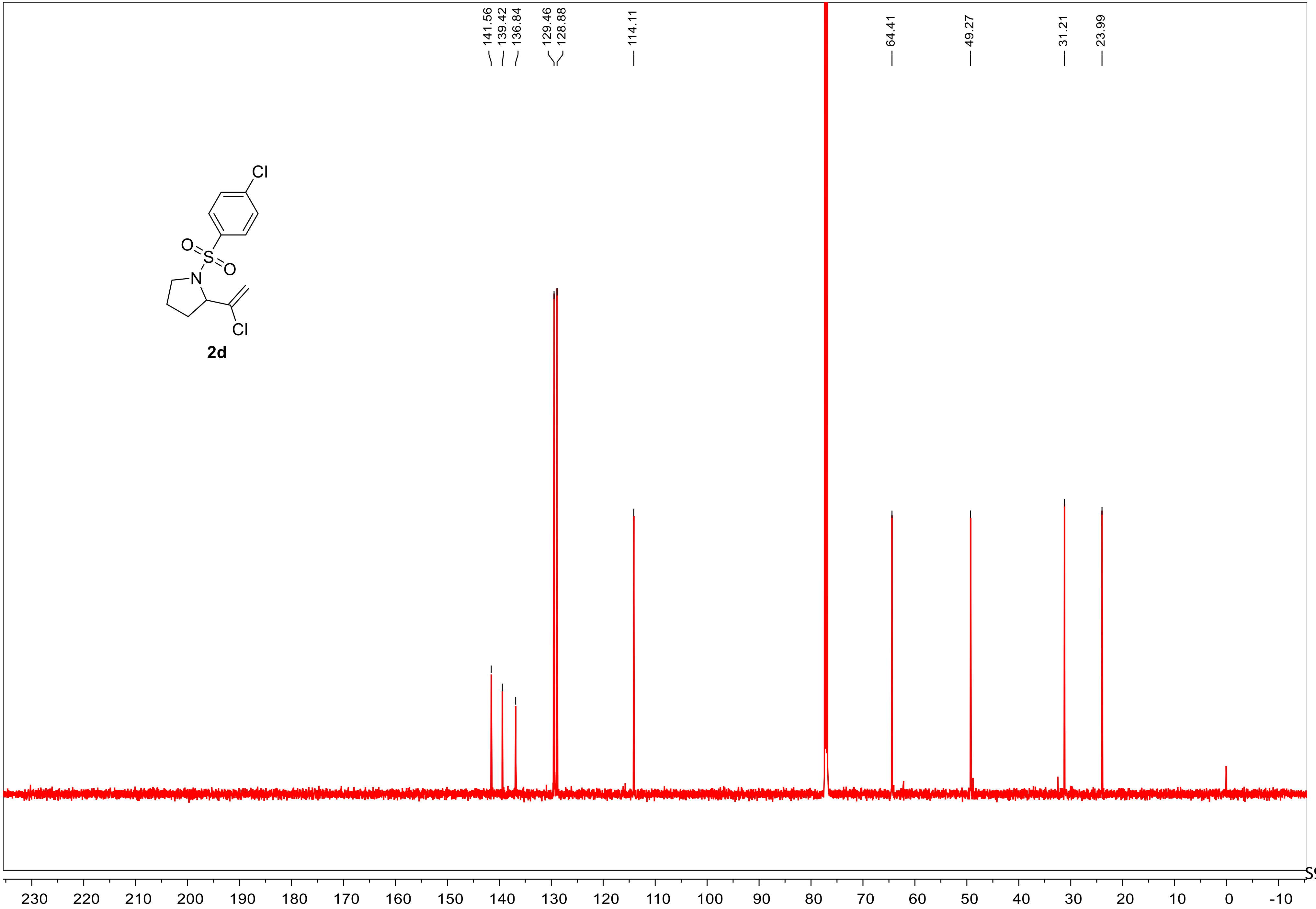

2d

DEPT 135 (150 MHz, $CDCl_3$)

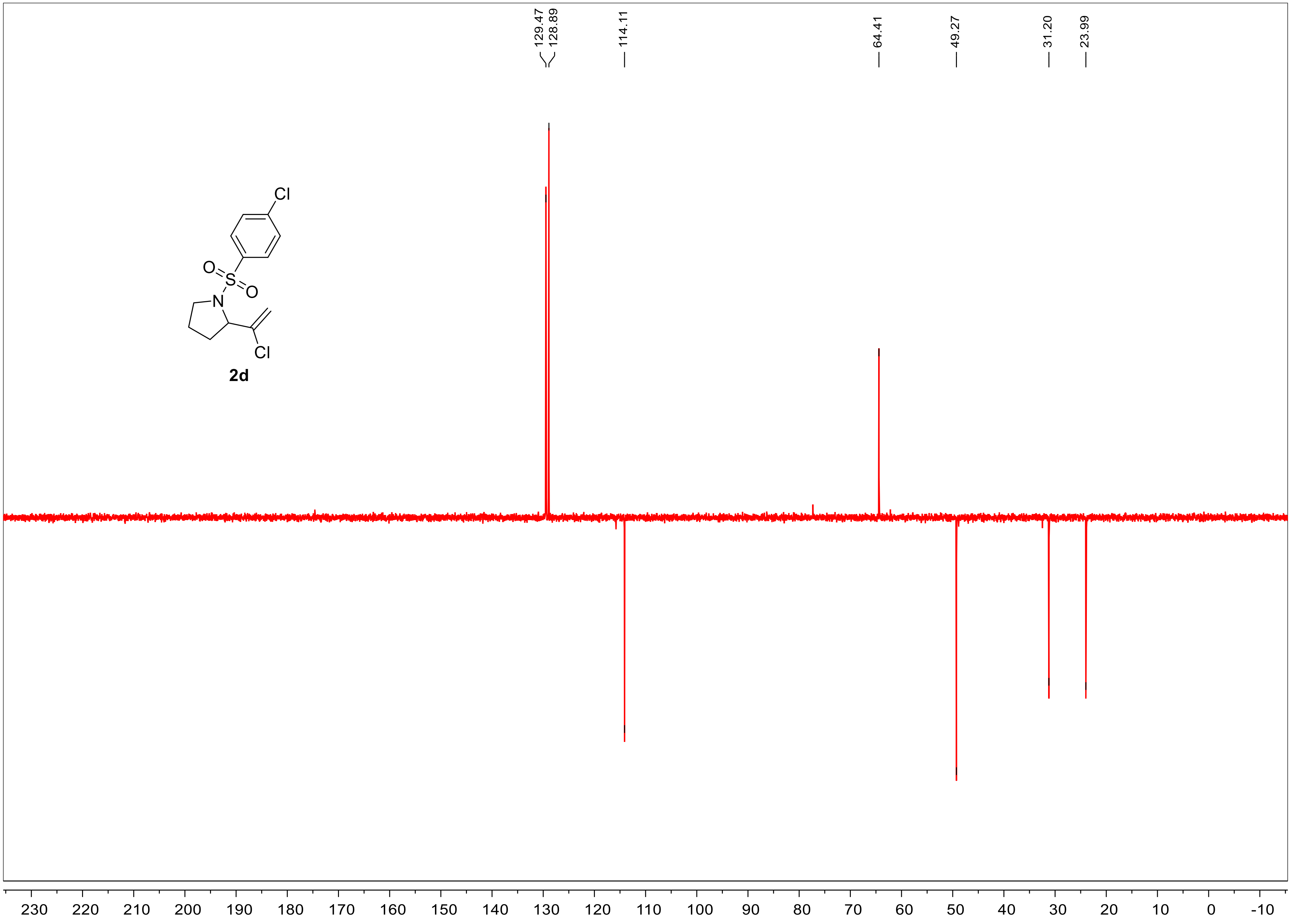

^{1}H-NMR (600 MHz, $CDCl_3$)

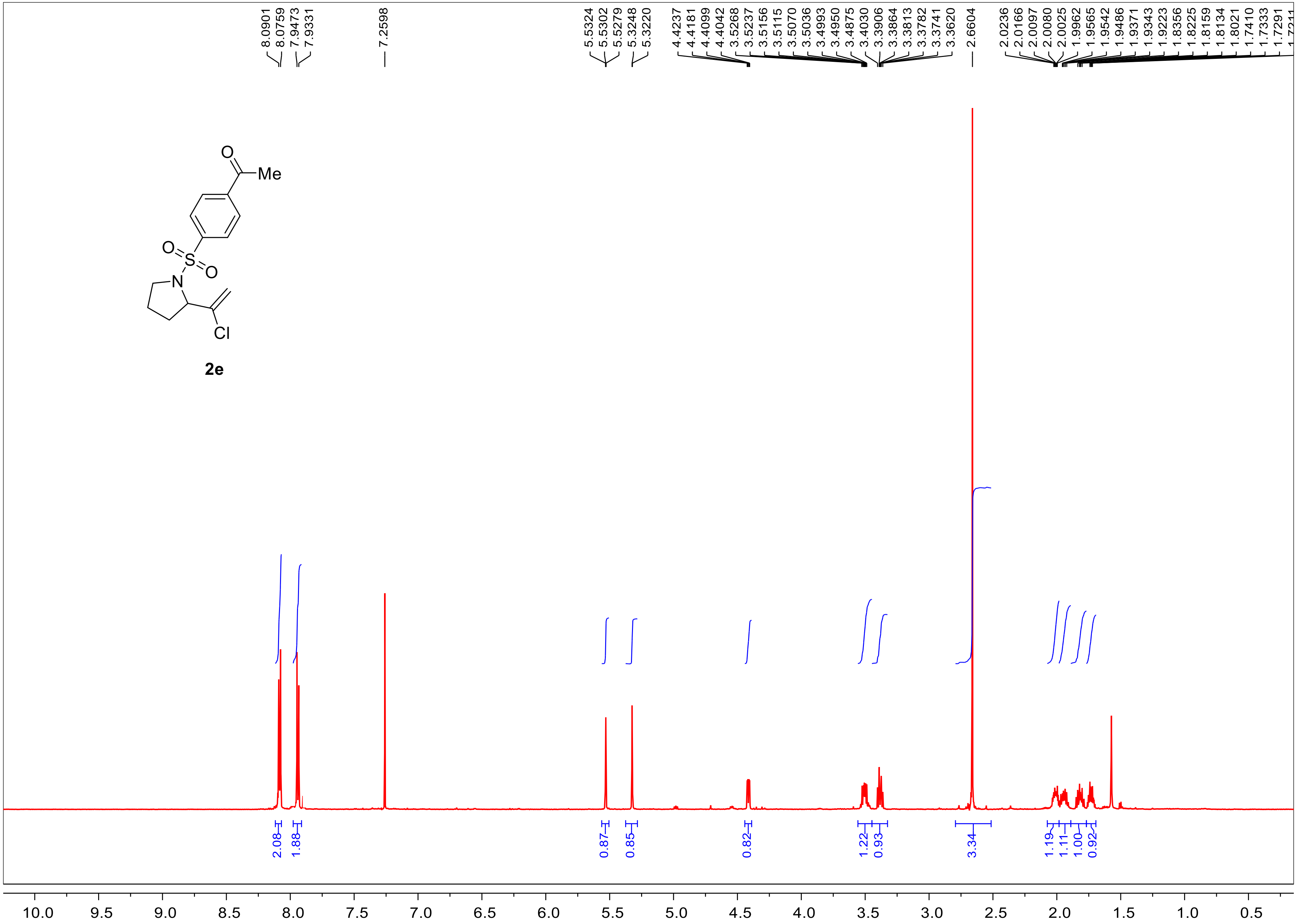

2D - COSY (600 MHz, $CDCl_3$)

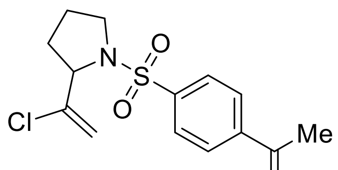

2e

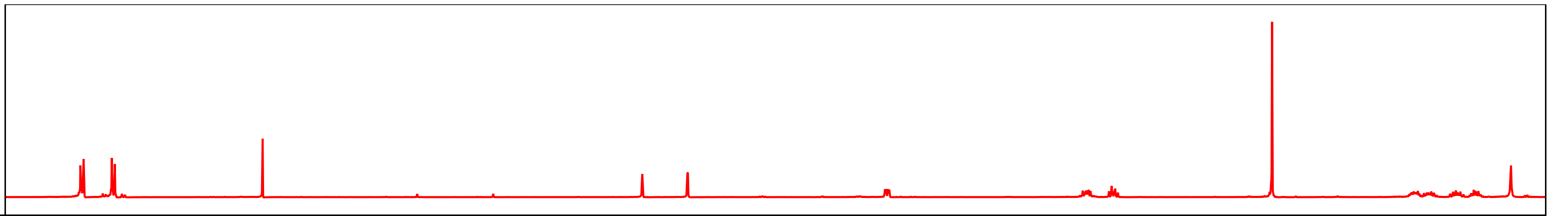

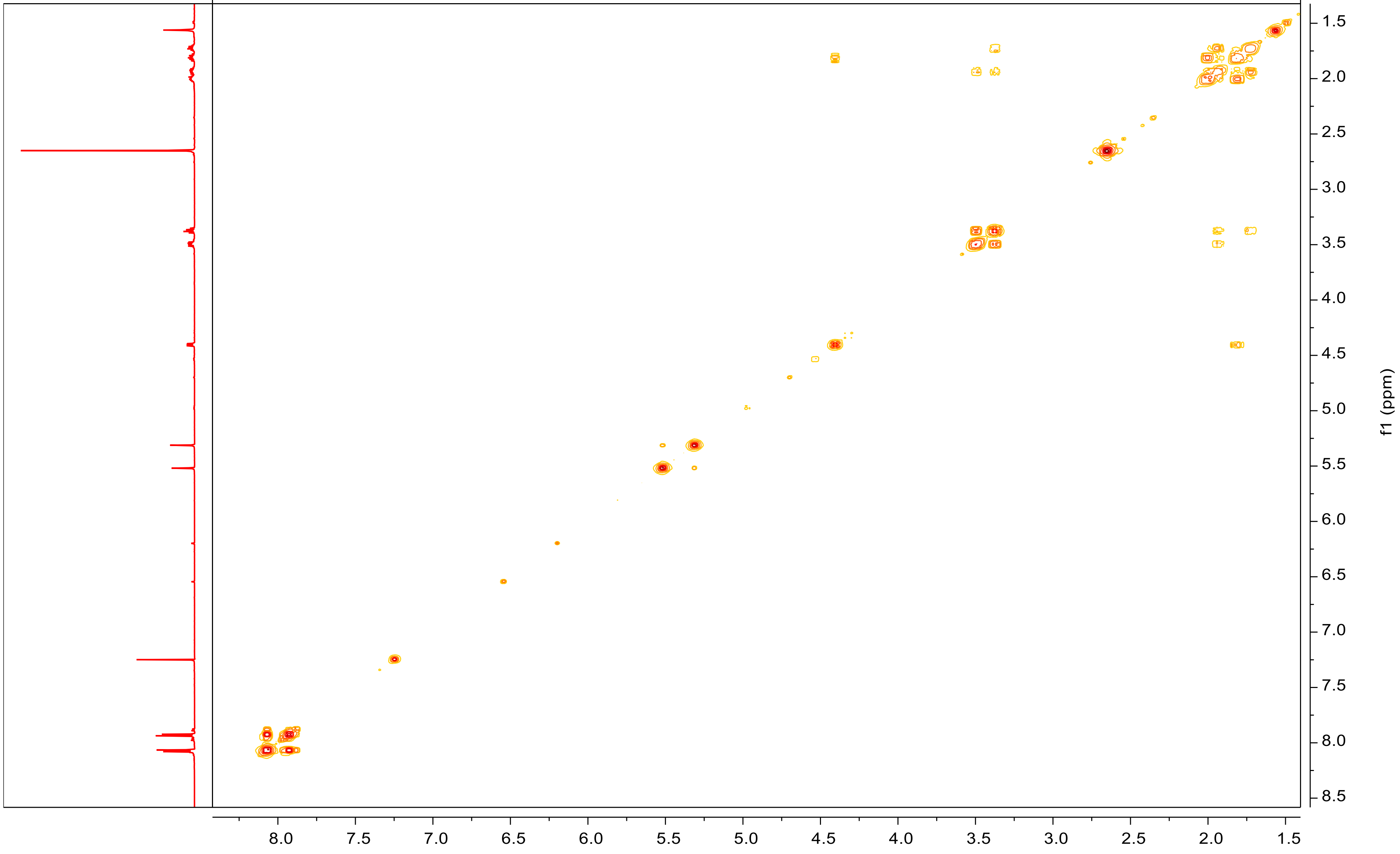

$^{13}C\{^1H\}$ **NMR** (150 MHz, $CDCl_3$)

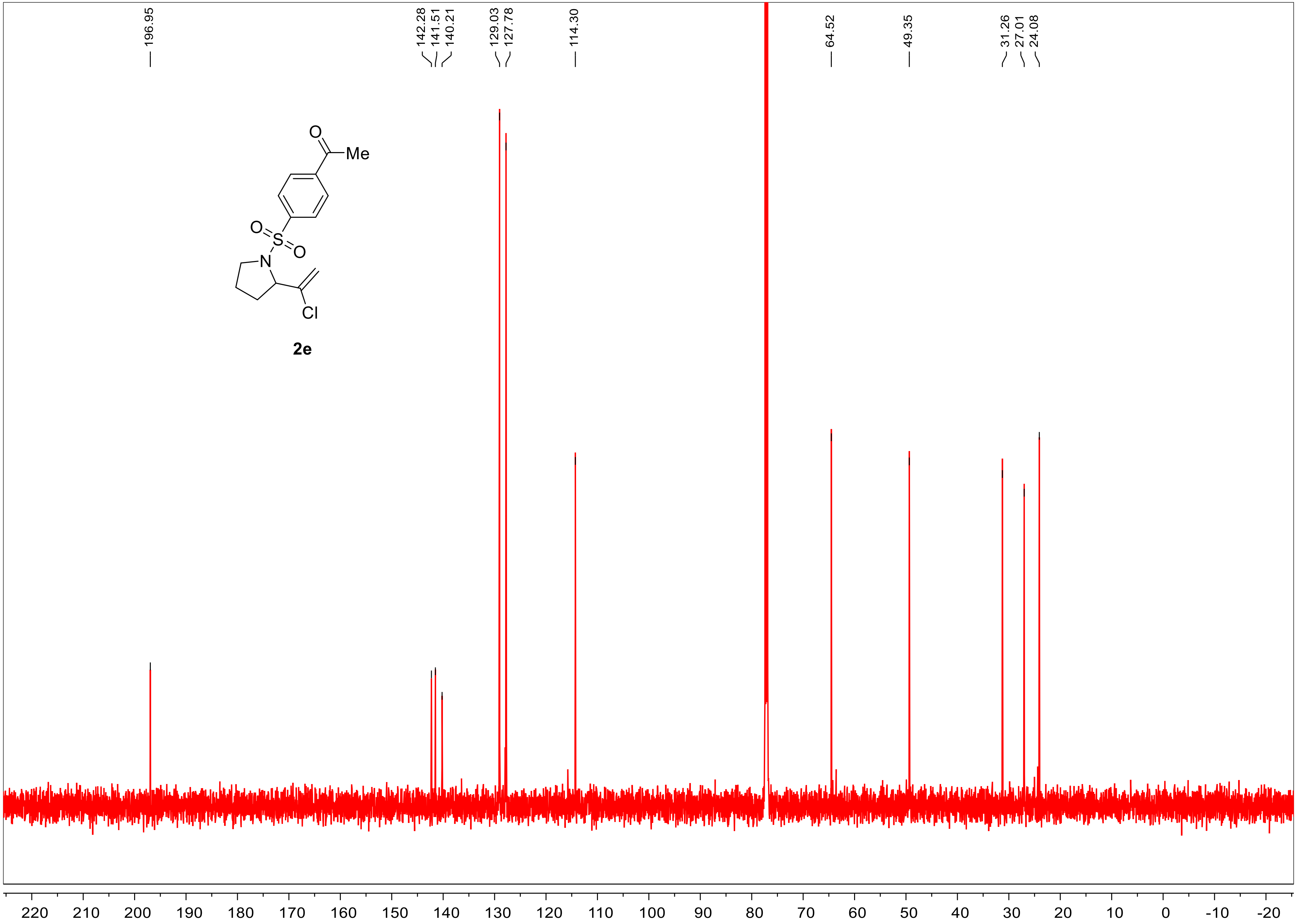

DEPT 135 (150 MHz, $CDCl_3$)

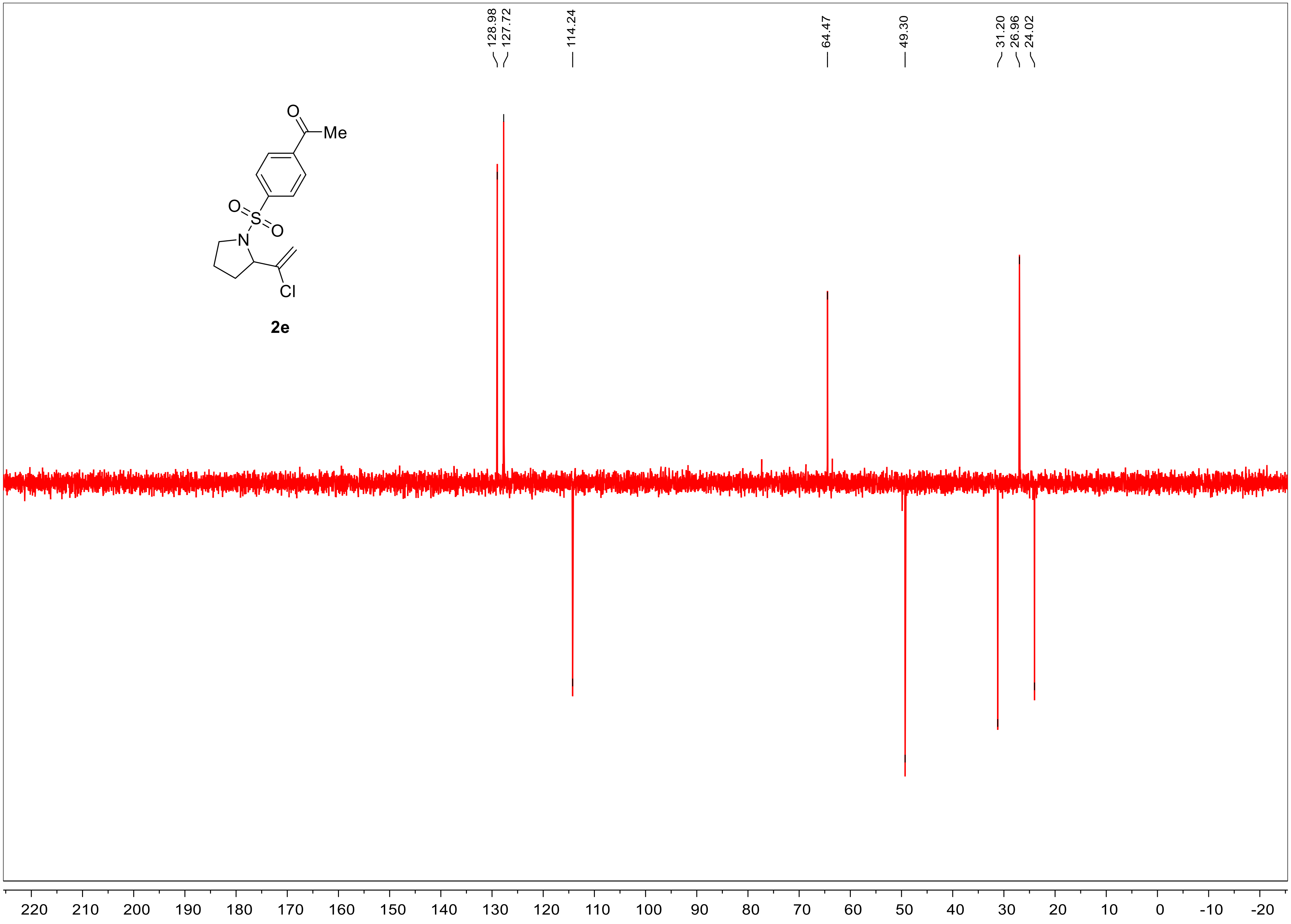

¹H-NMR (600 MHz, $CDCl_3$)

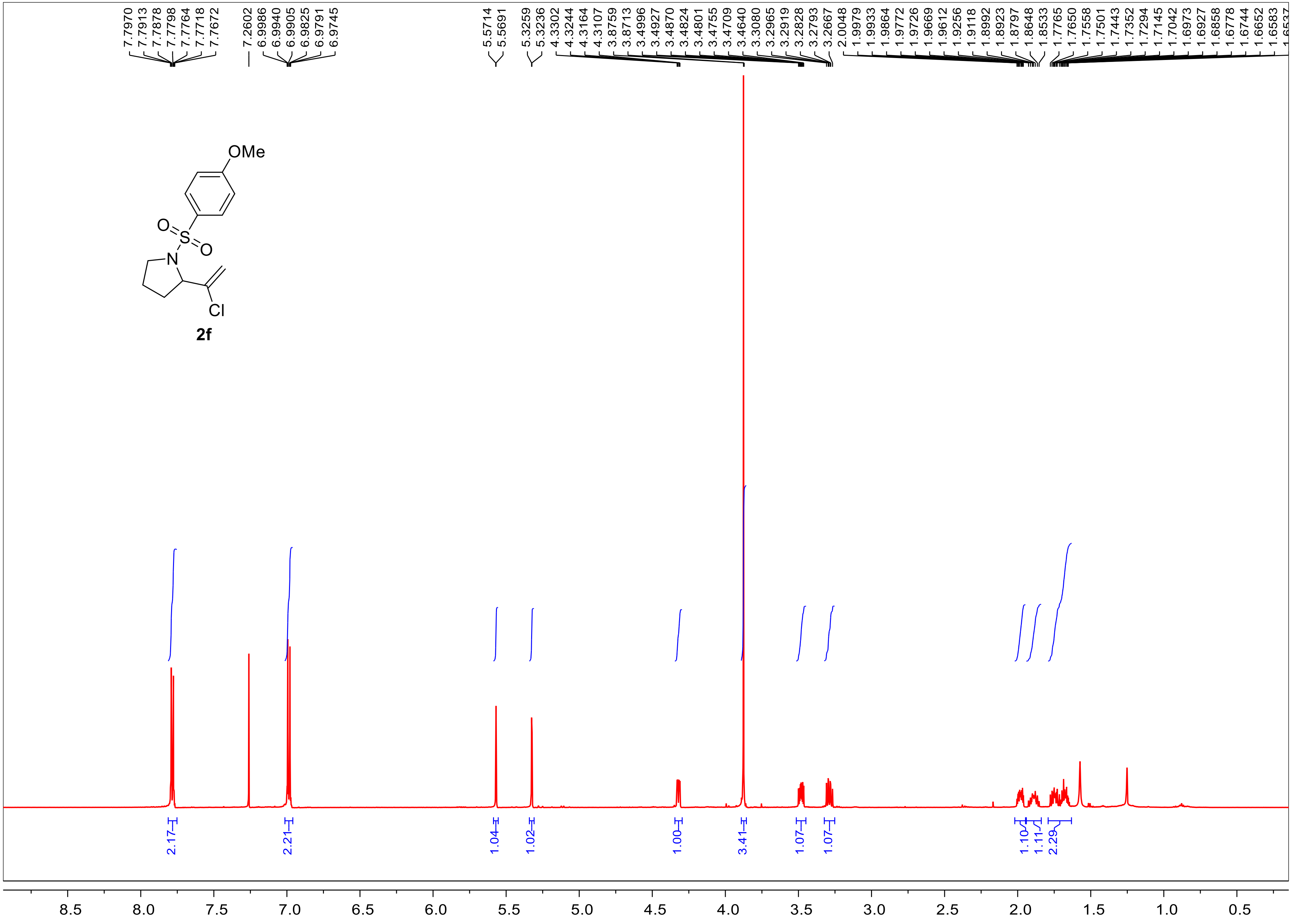

2D - COSY (600 MHz, $CDCl_3$)

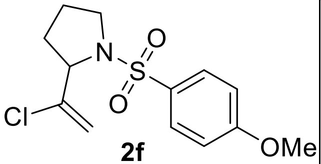

2f

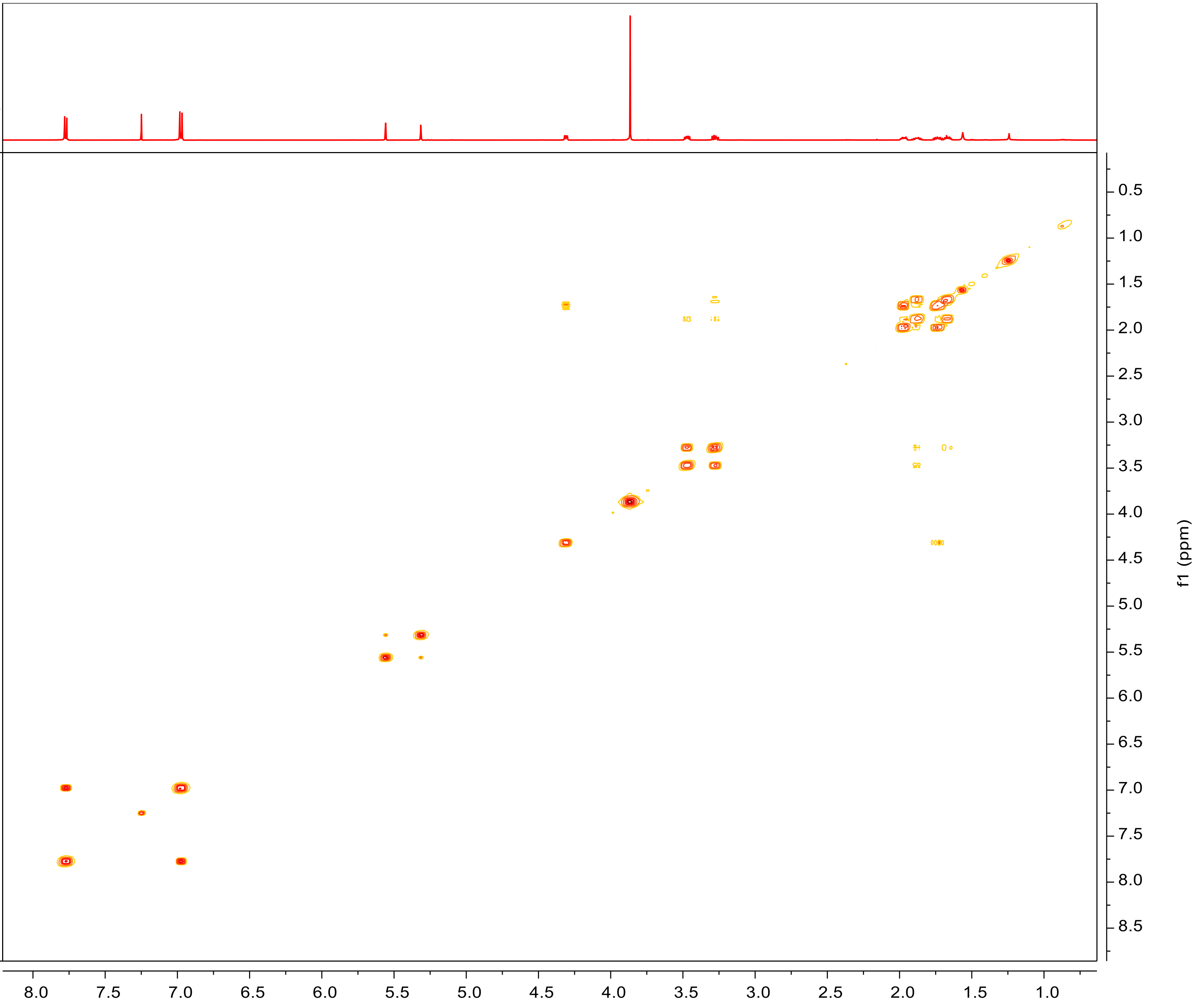

$^{13}C\{^1H\}$ **NMR** (150 MHz, $CDCl_3$)

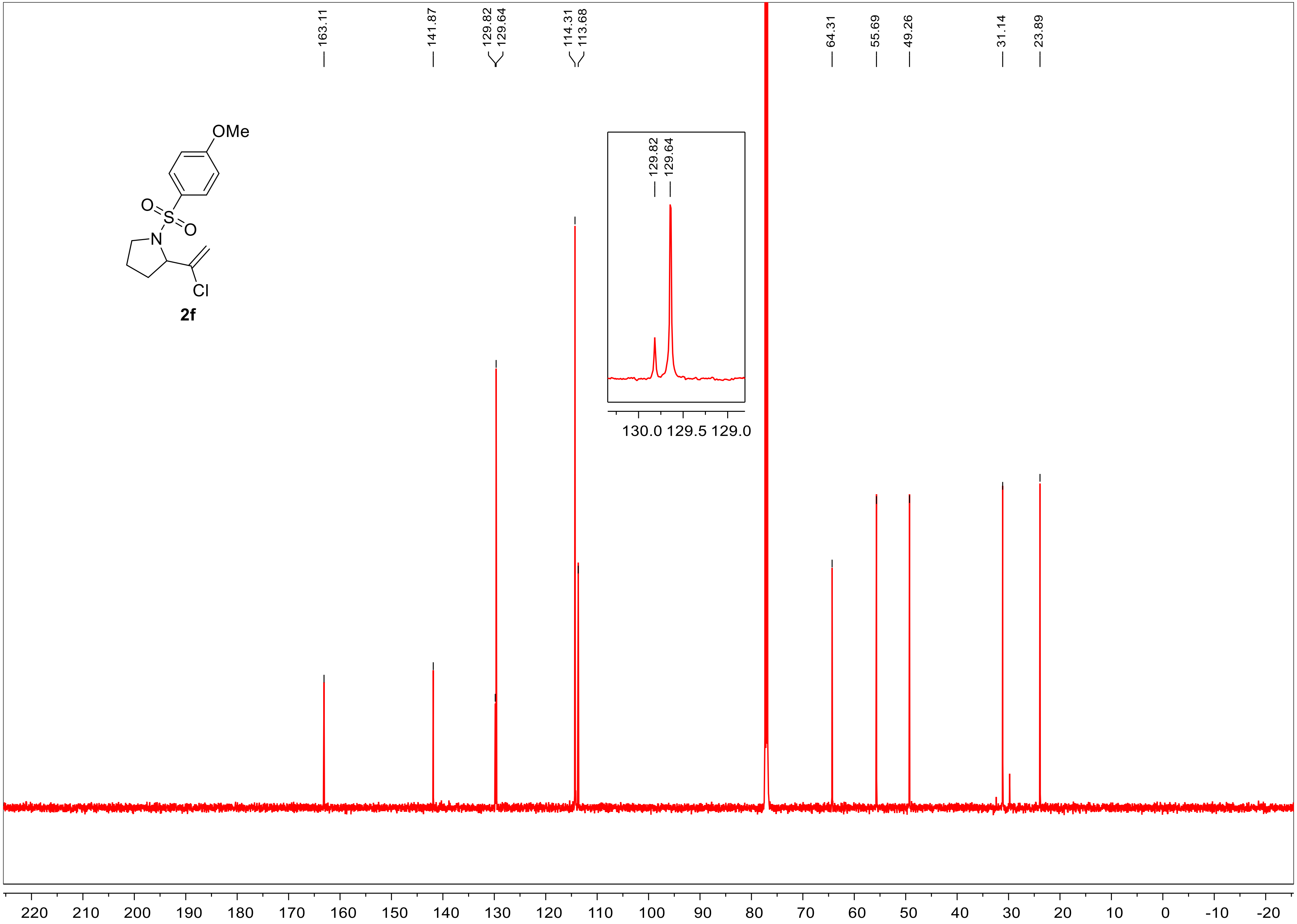

DEPT 135 (150 MHz, $CDCl_3$)

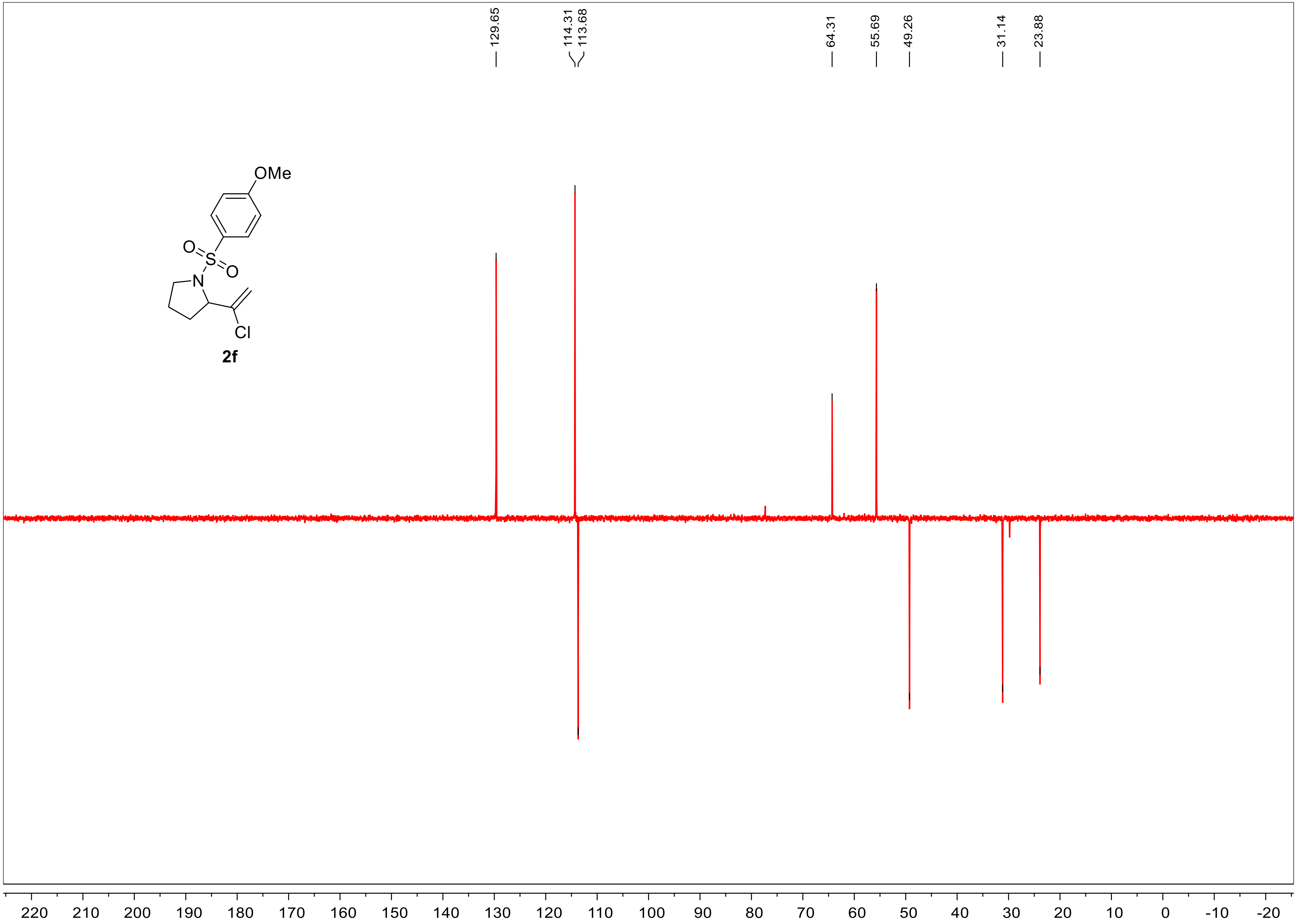

^{1}H-NMR (600 MHz, $CDCl_3$)

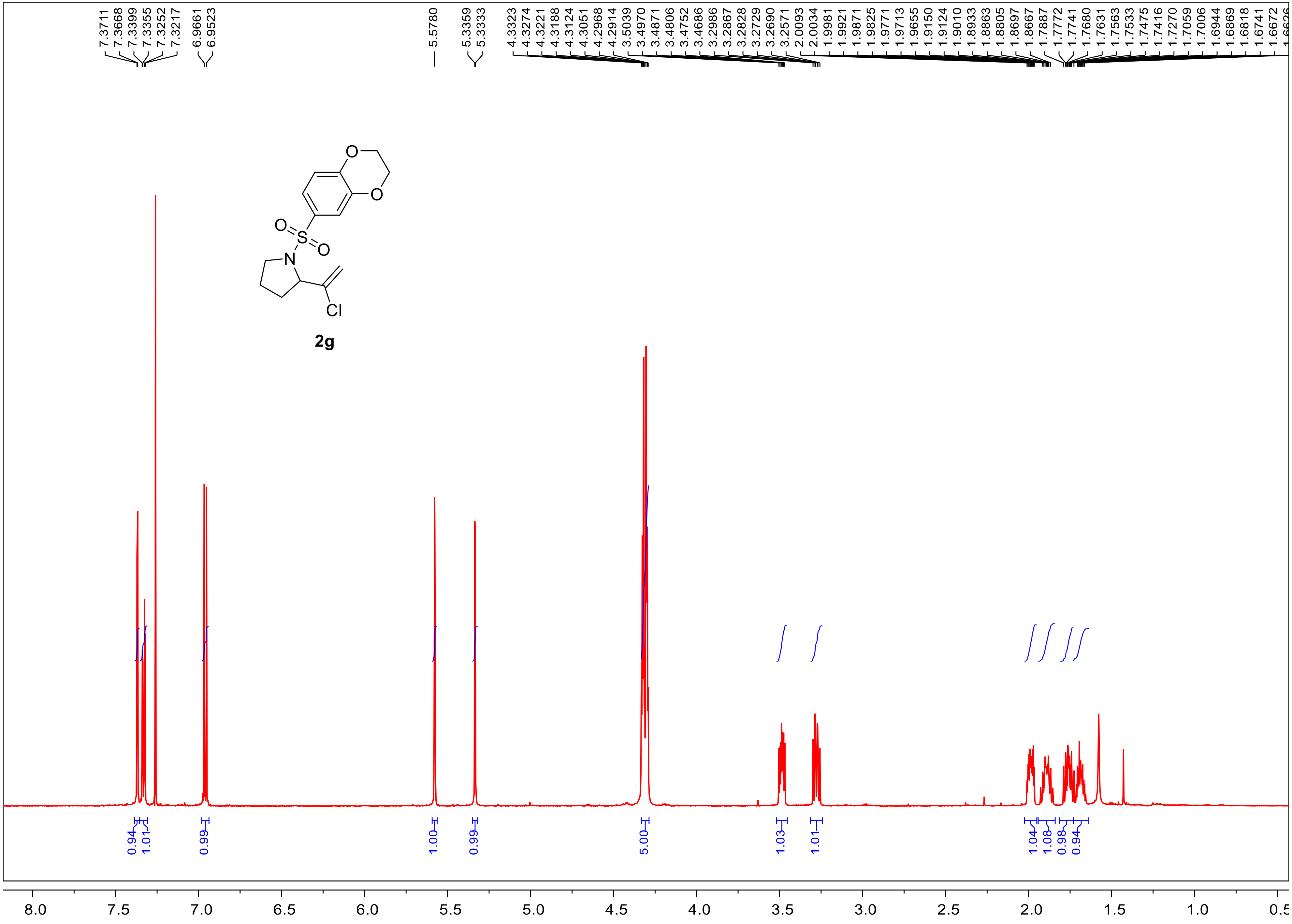

2D - COSY (600 MHz, $CDCl_3$)

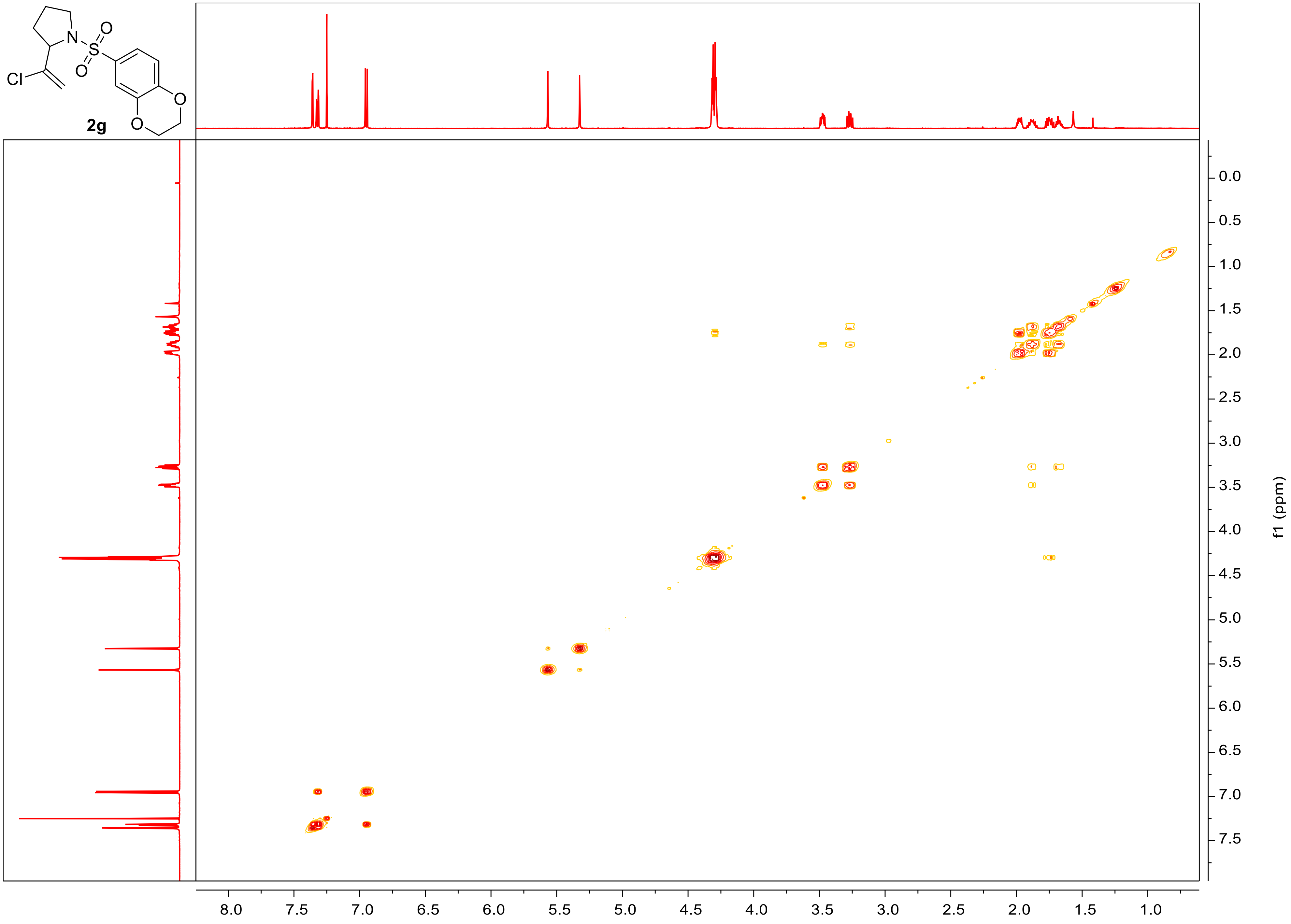

$^{13}C\{^{1}H\}$ **NMR** (150 MHz, $CDCl_3$)

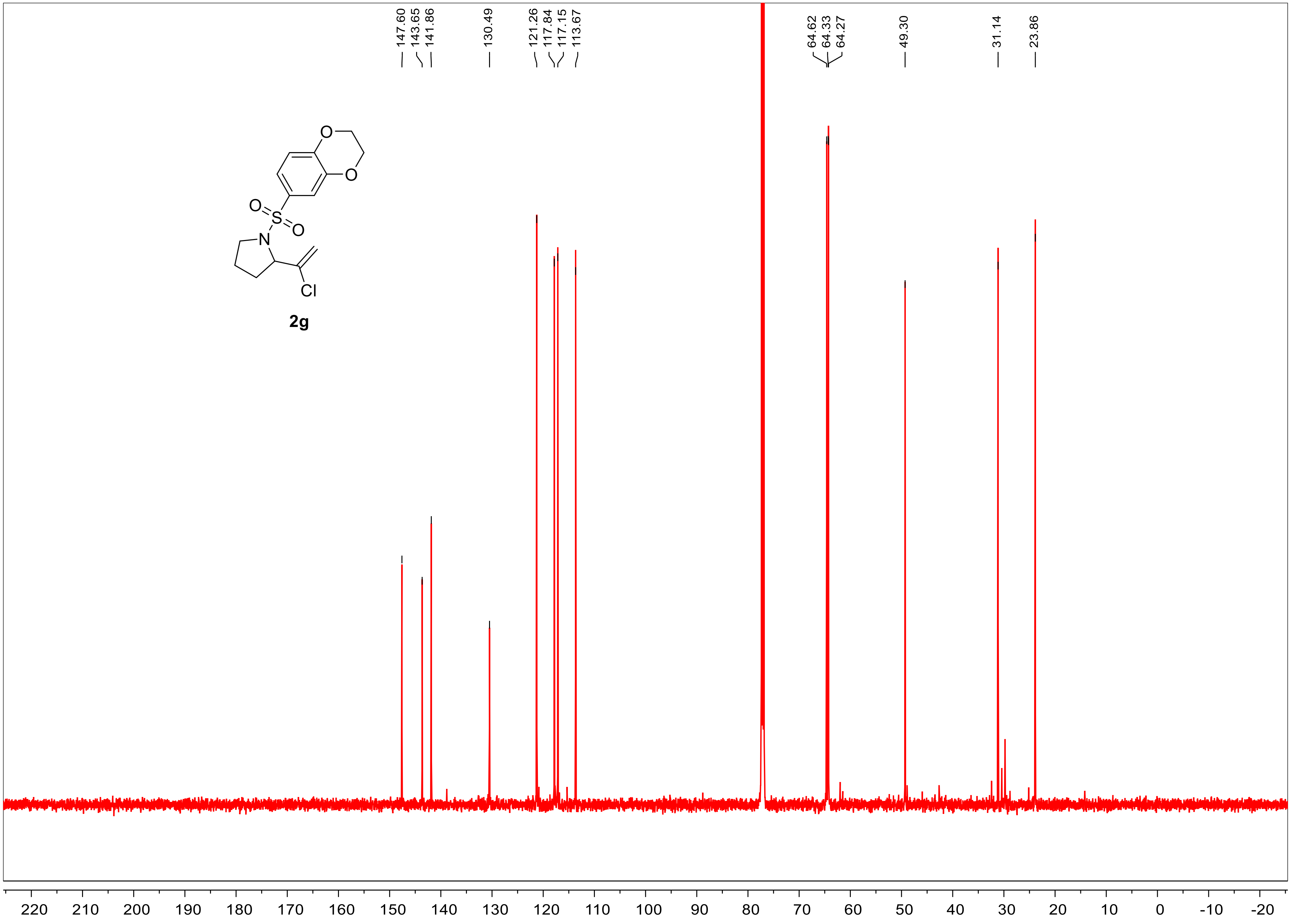

DEPT 135 (150 MHz, $CDCl_3$)

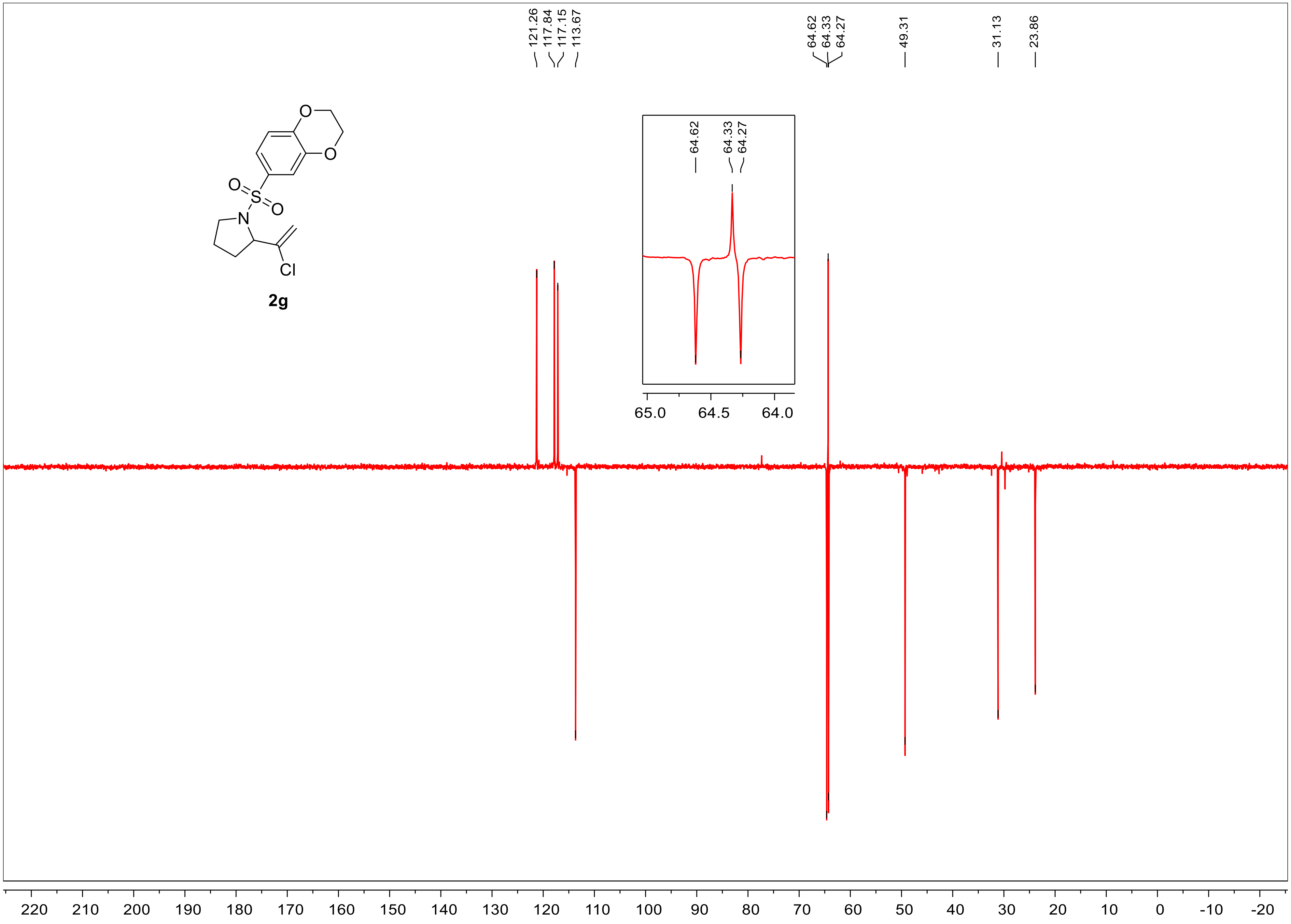

^{1}H-NMR (600 MHz, $CDCl_3$)

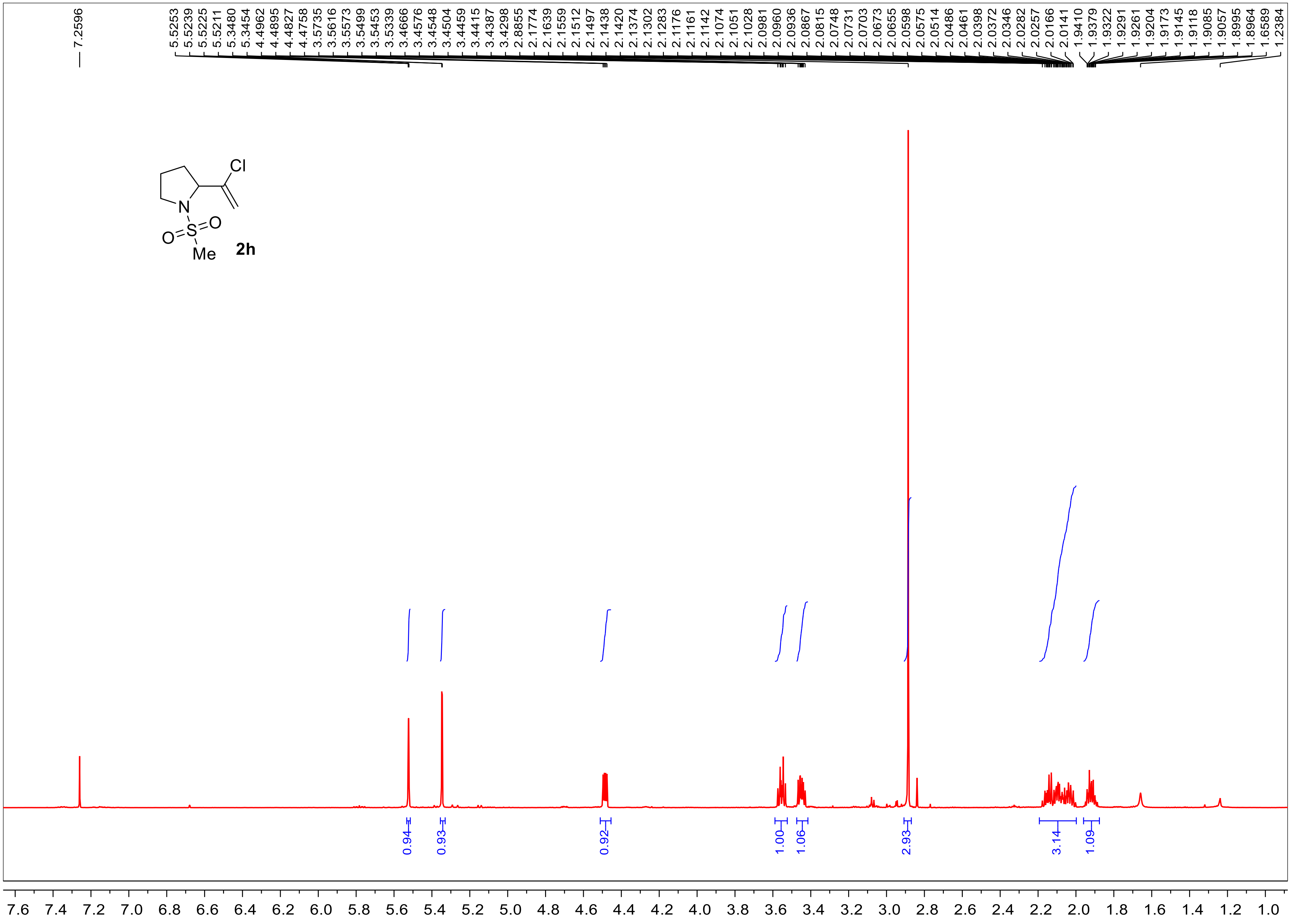

2D - COSY (600 MHz, $CDCl_3$)

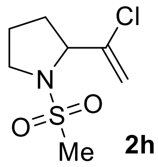

2h

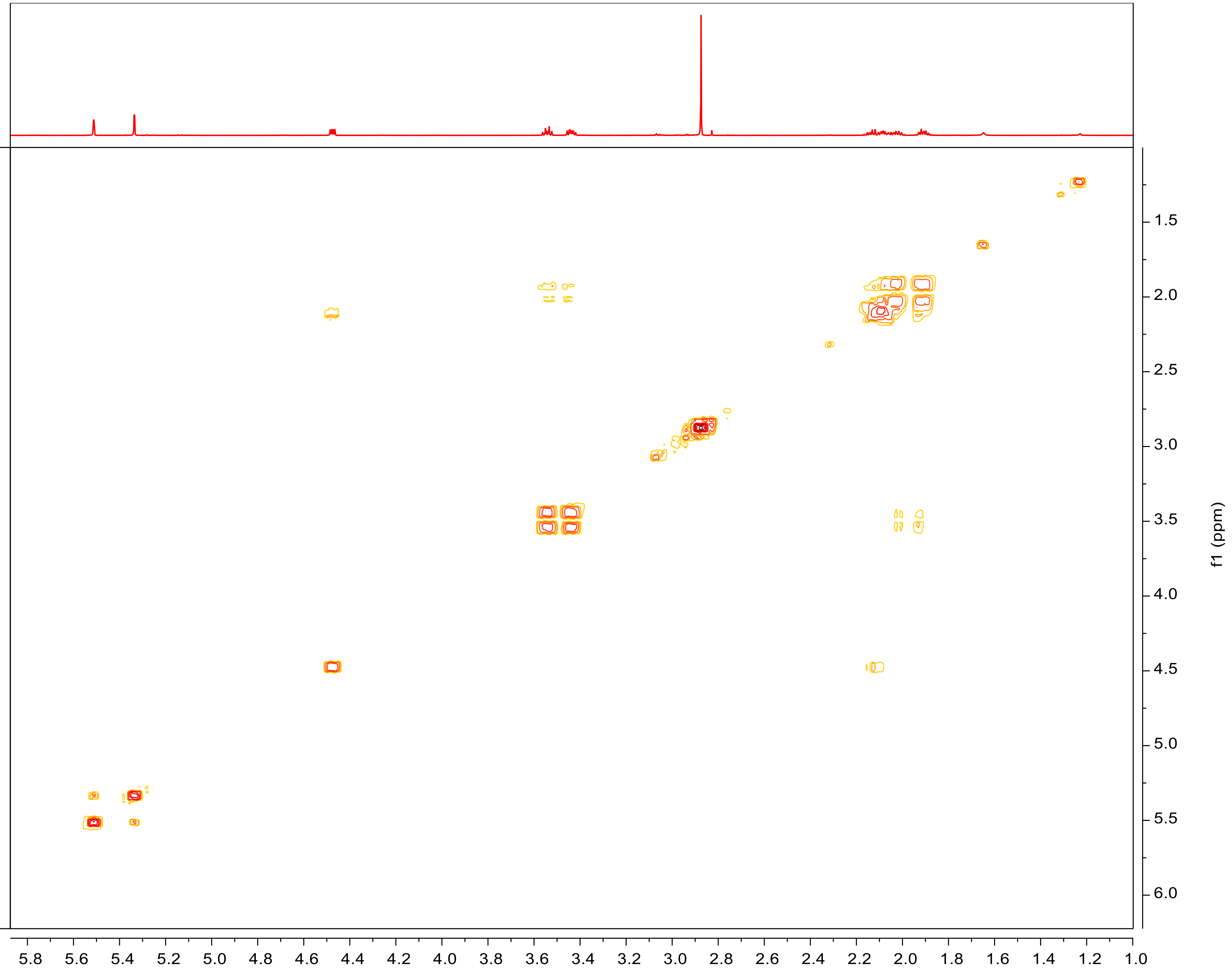

$^{13}C\{^1H\}$ NMR (150 MHz, $CDCl_3$)

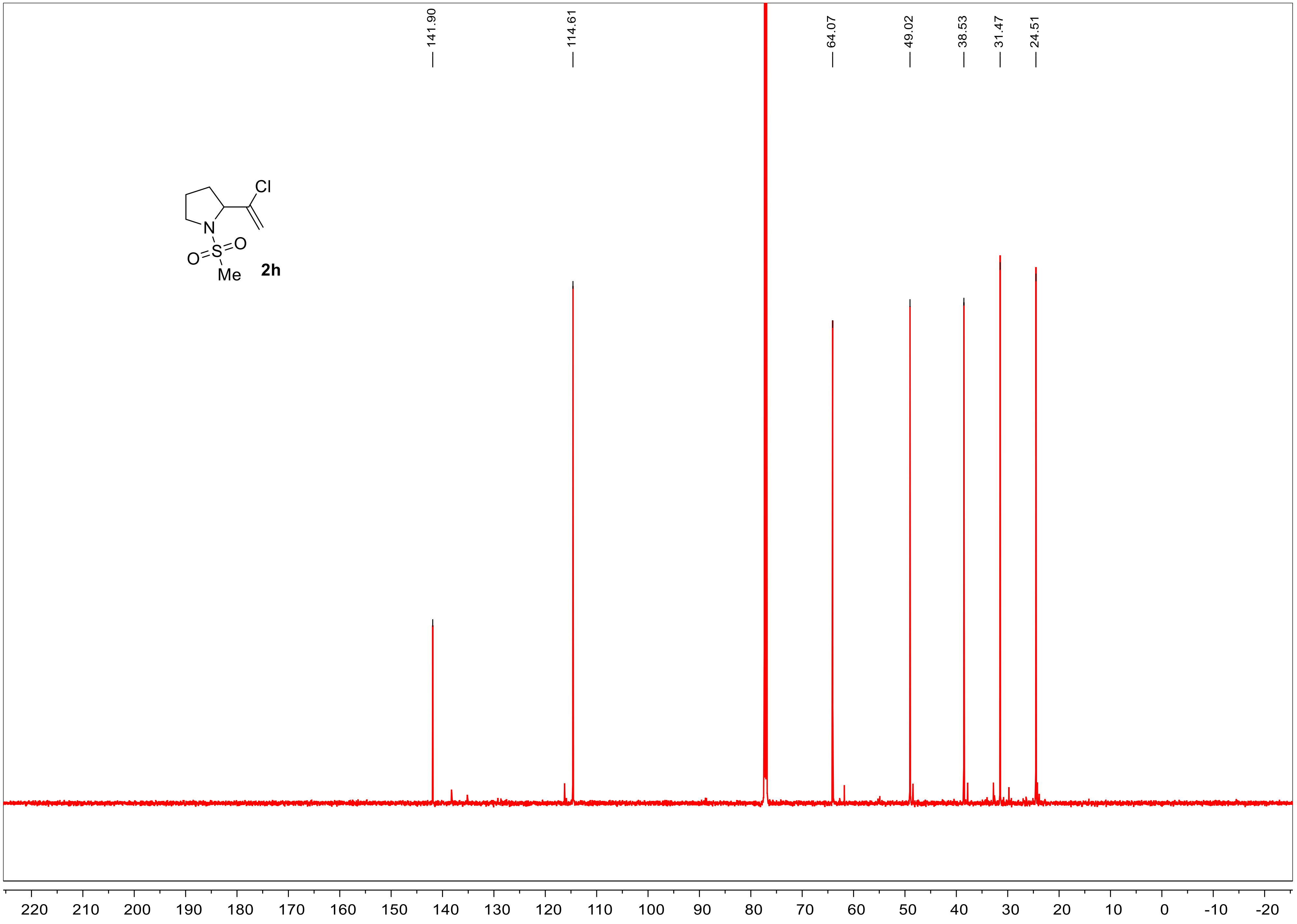

DEPT 135 (150 MHz, $CDCl_3$)

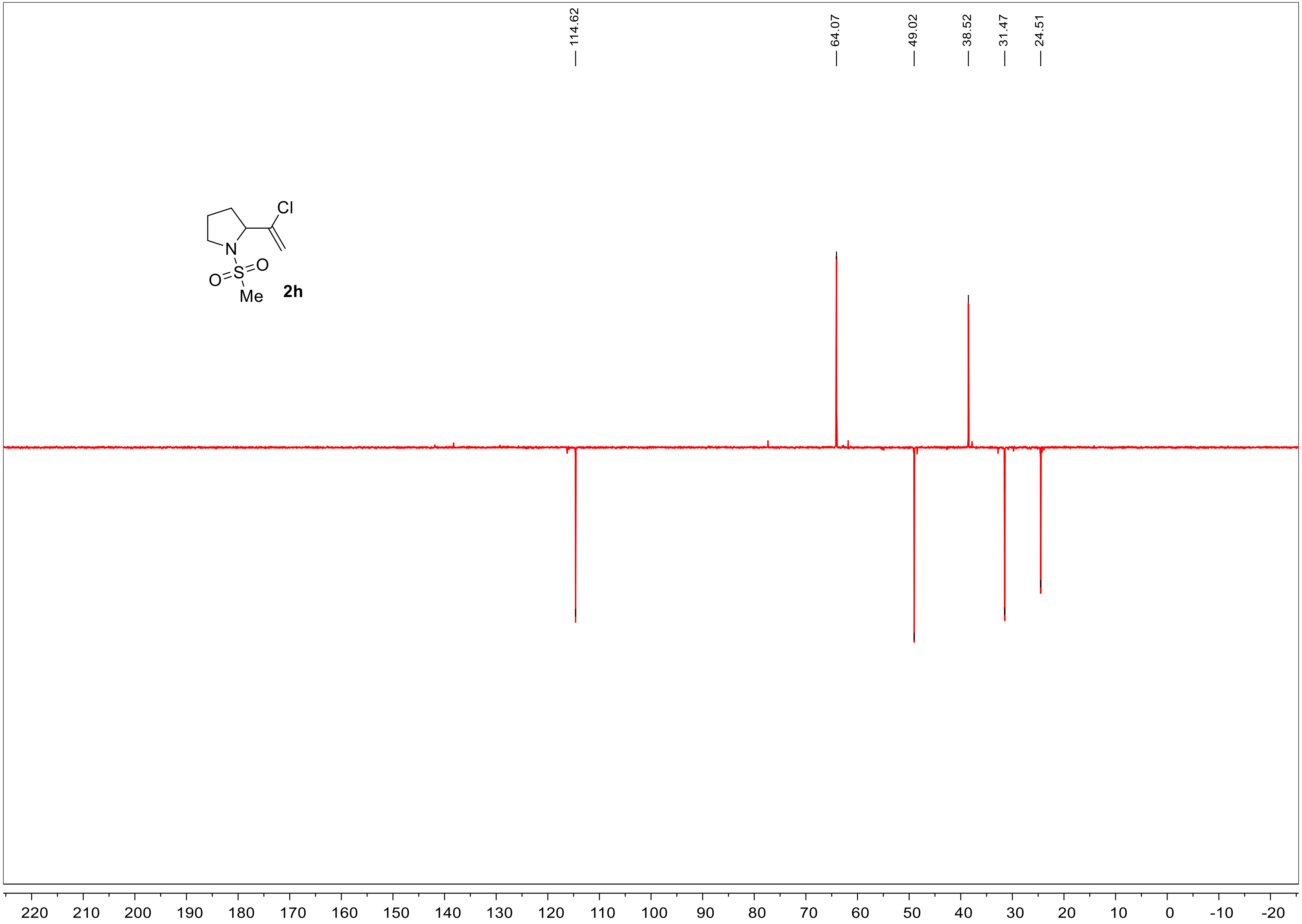

¹H-NMR (600 MHz, $CDCl_3$)

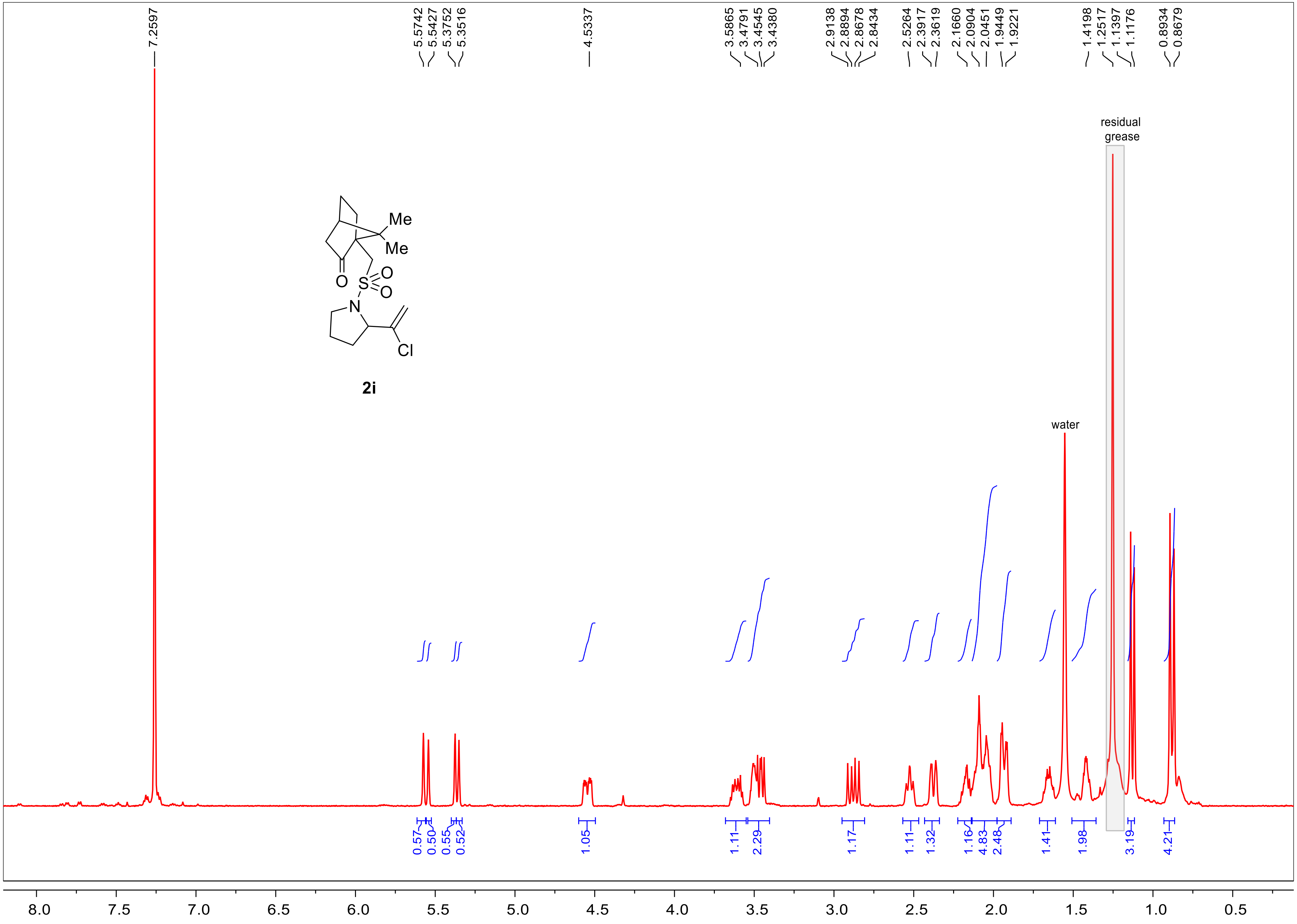

2D - COSY (600 MHz, $CDCl_3$)

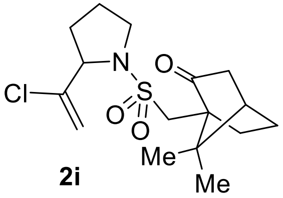

2i

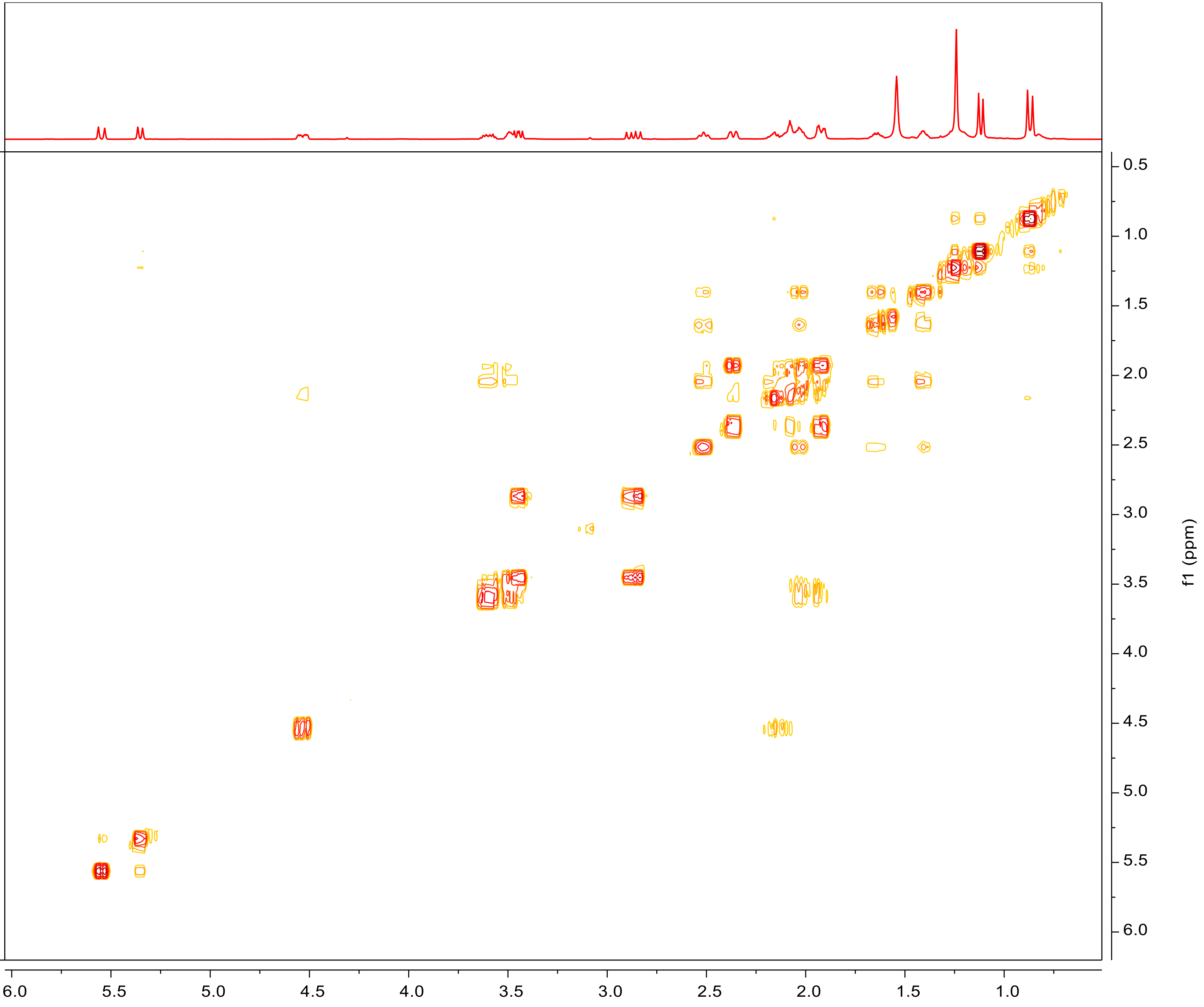

$^{13}C\{^{1}H\}$ **NMR** (150 MHz, $CDCl_3$)

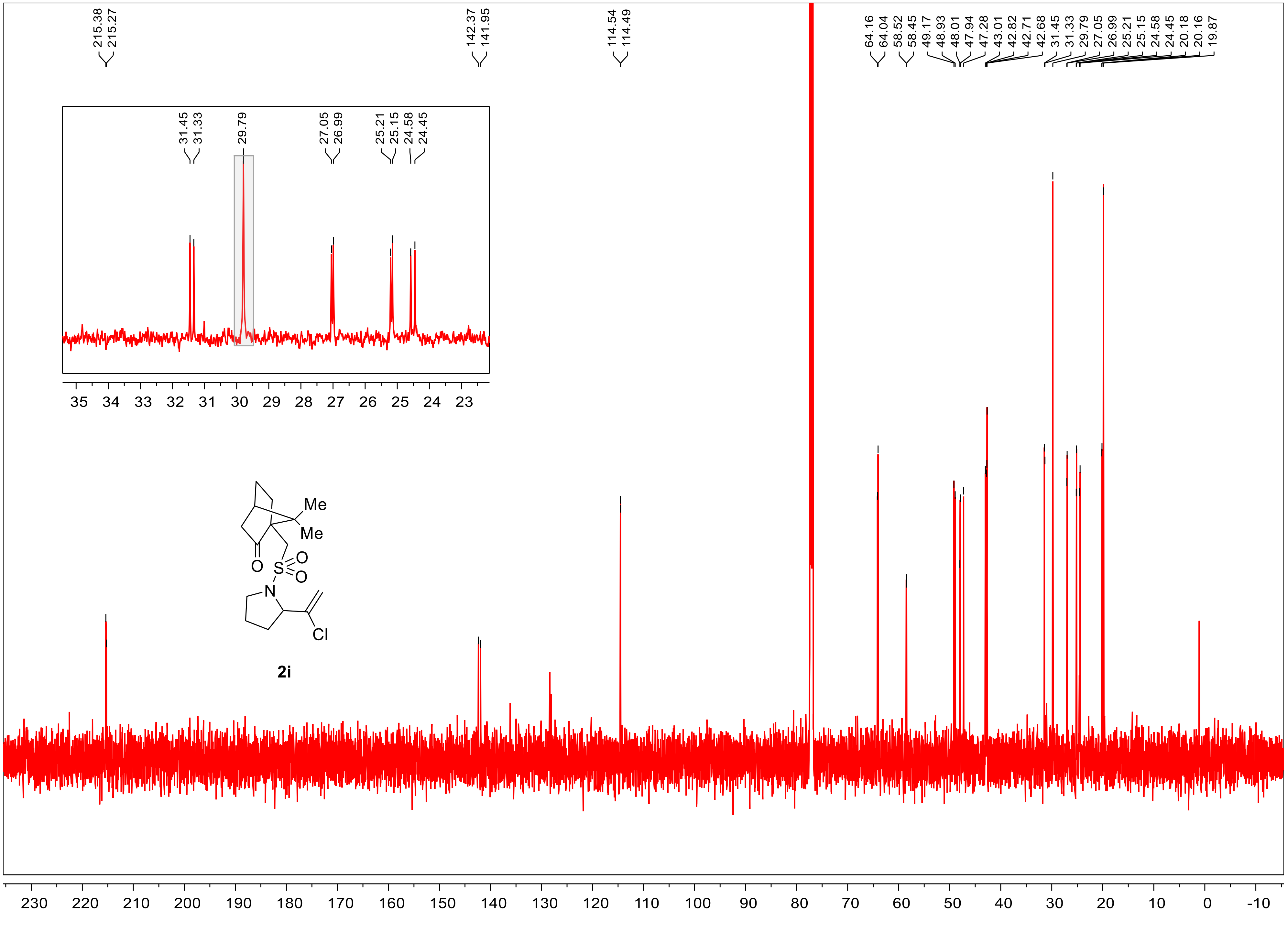

DEPT 135 (150 MHz, $CDCl_3$)

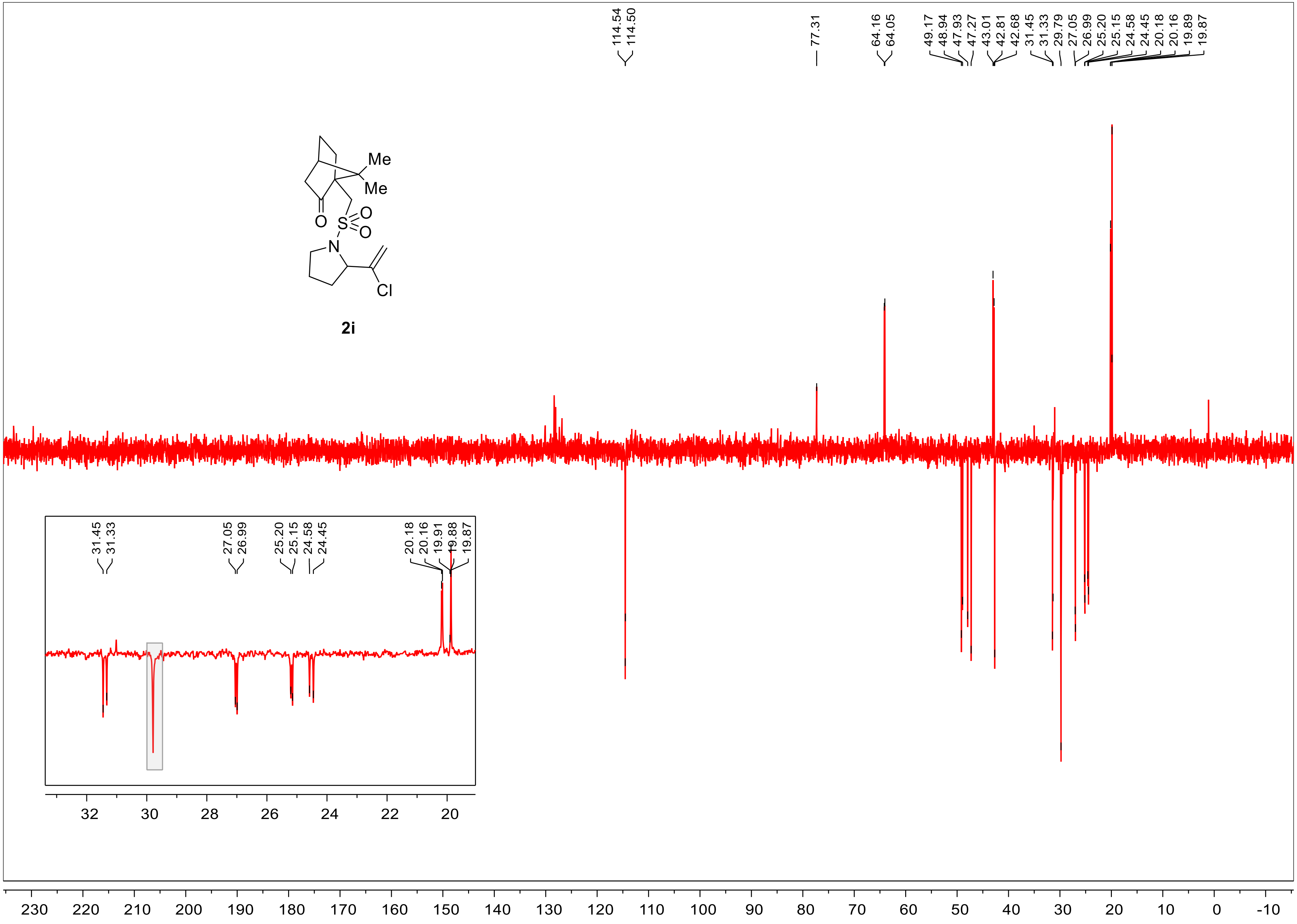

¹H-NMR (600 MHz, $CDCl_3$)

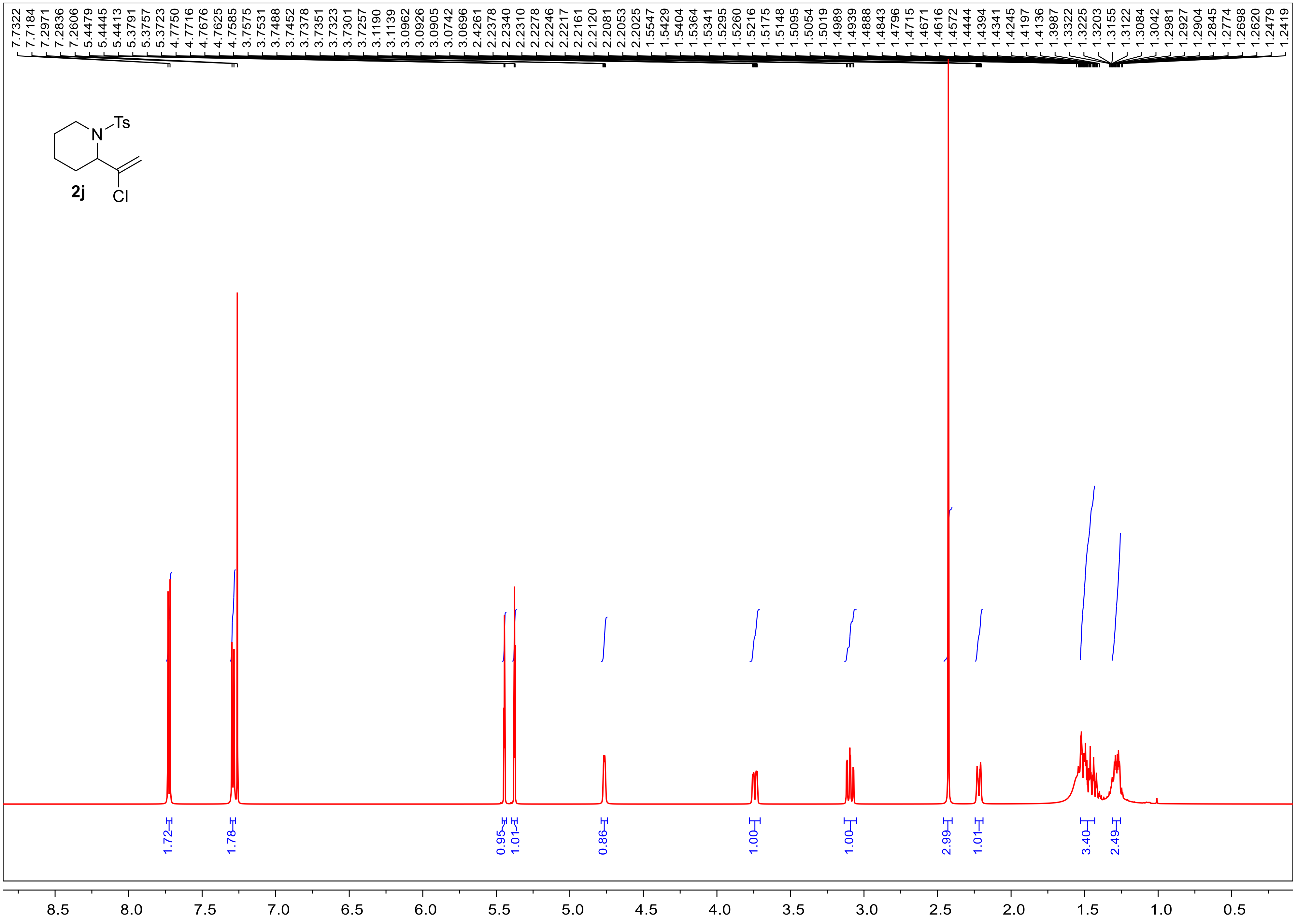

2D - COSY (600 MHz, $CDCl_3$)

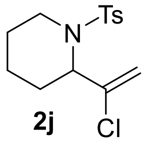

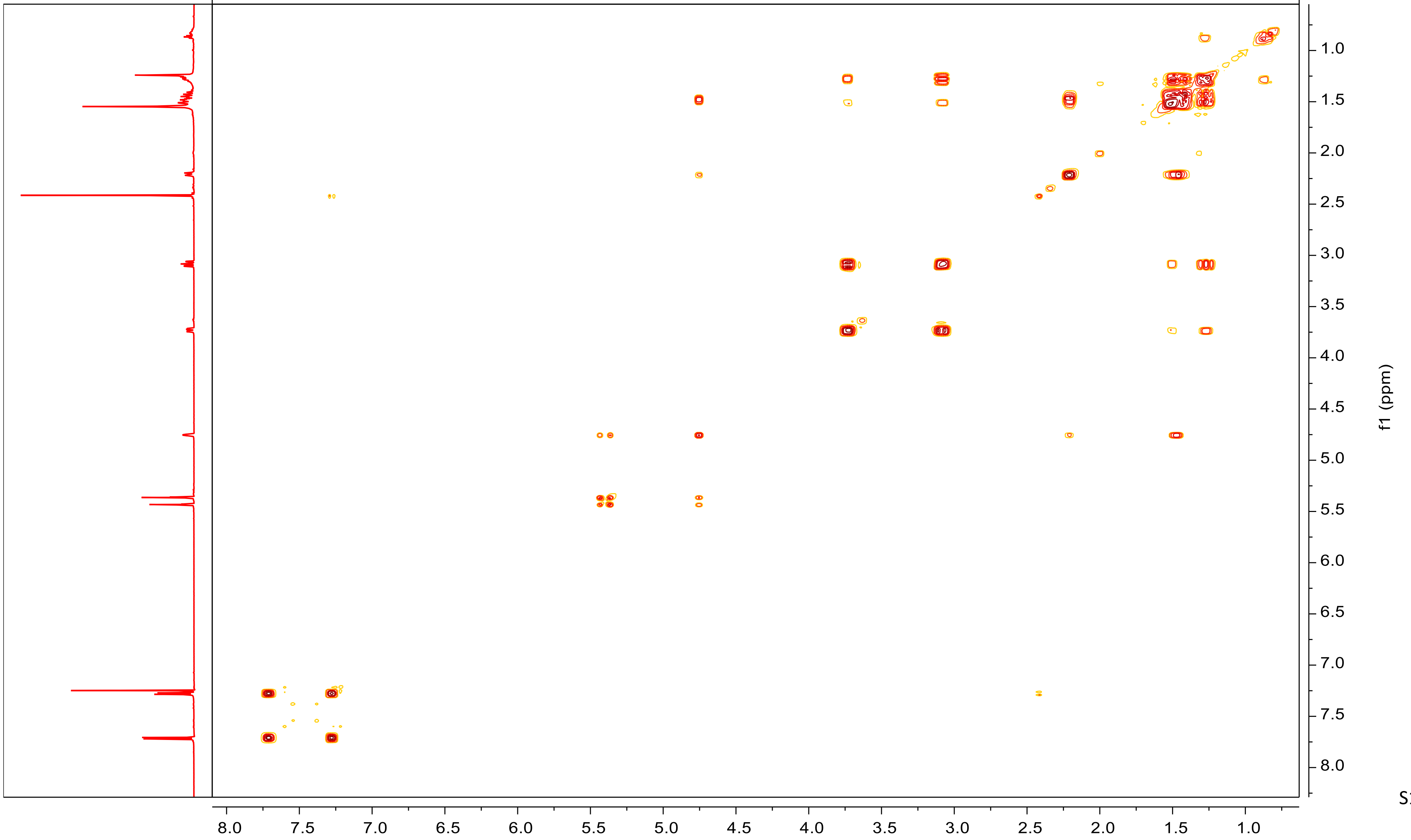

$^{13}C\{^{1}H\}$ NMR (150 MHz, $CDCl_3$)

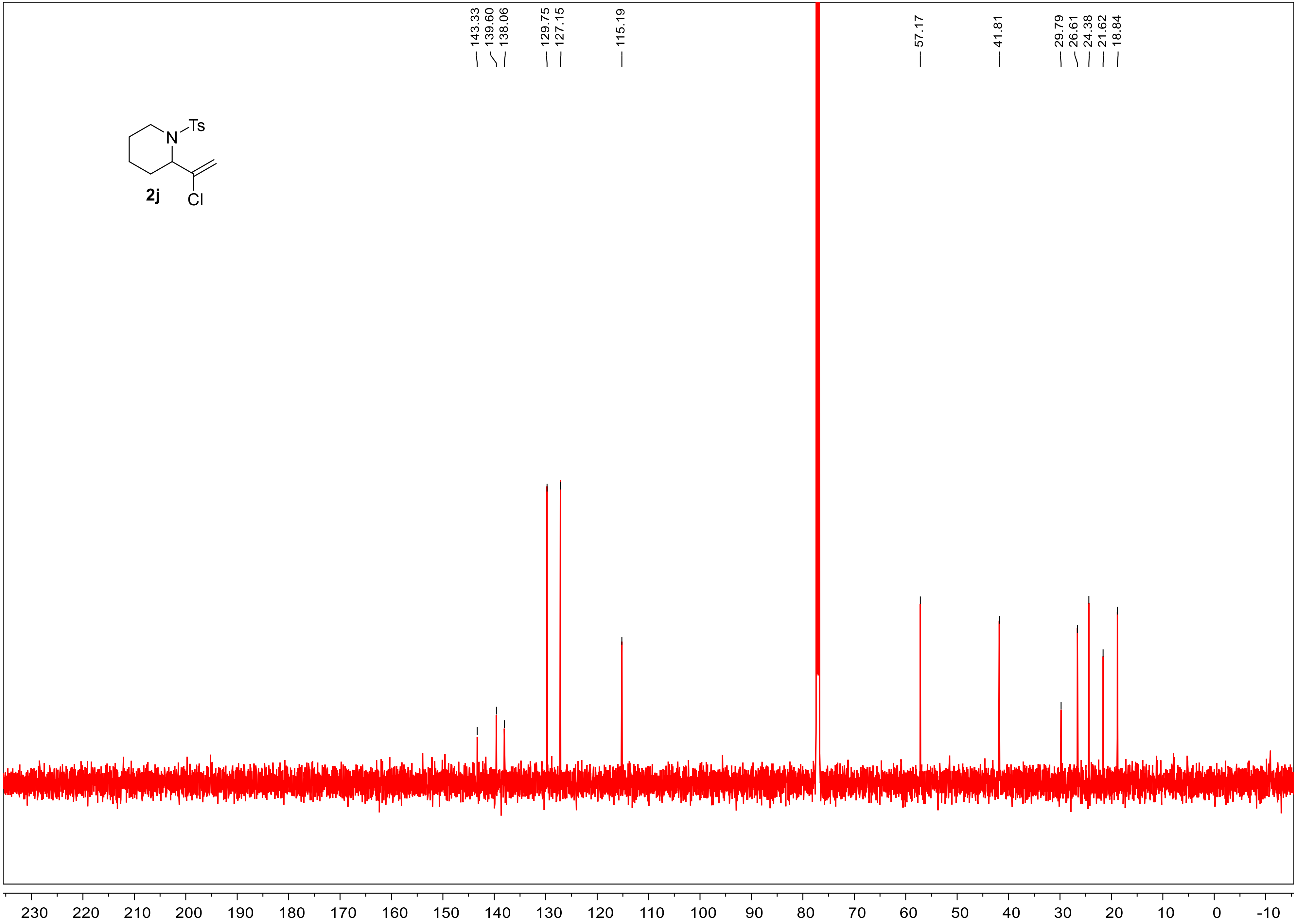

DEPT 135 (150 MHz, $CDCl_3$)

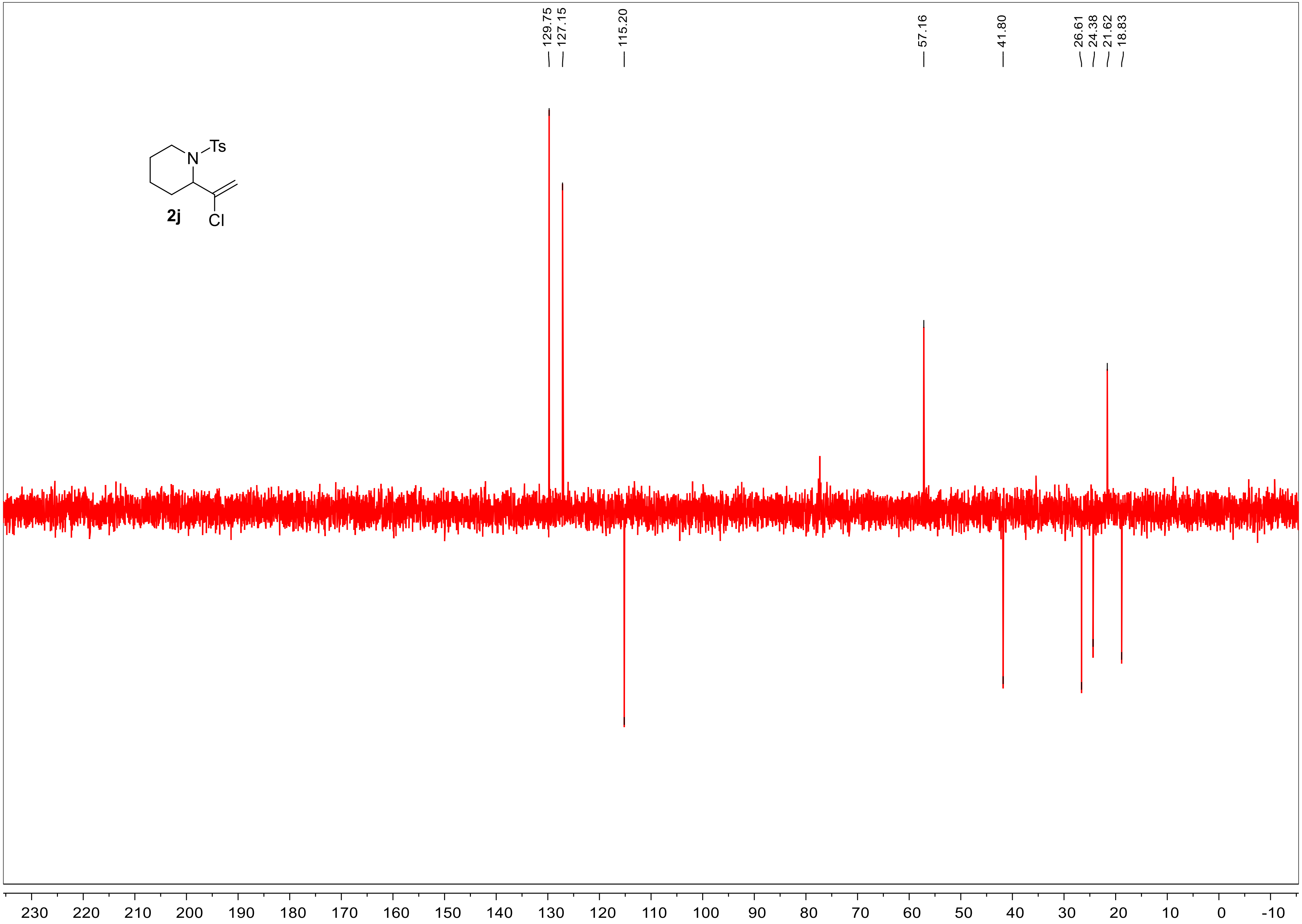

^{1}**H-NMR** (600 MHz, $CDCl_3$)

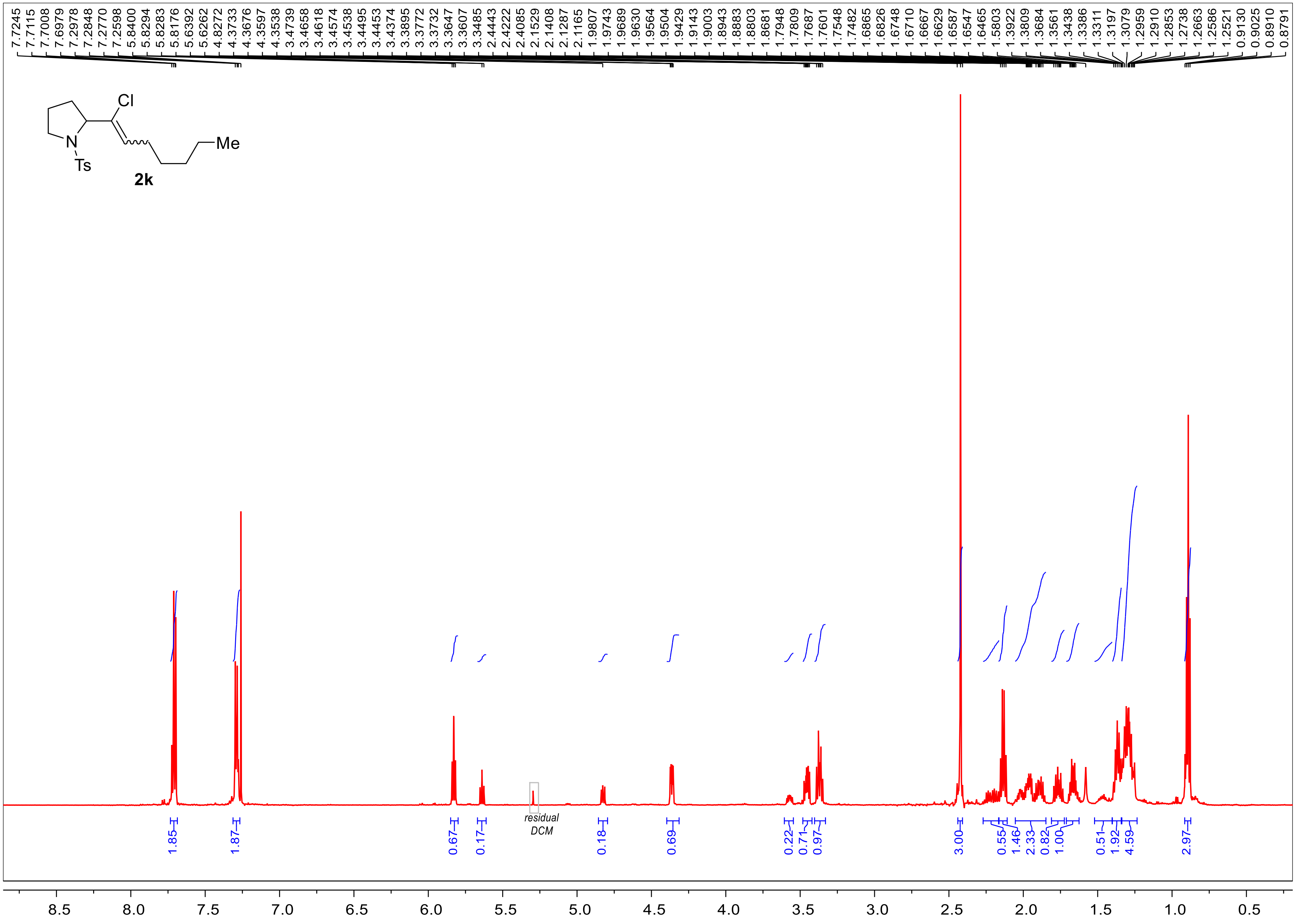

2D - COSY (600 MHz, $CDCl_3$)

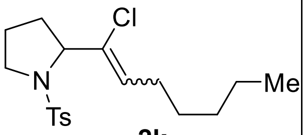

2k

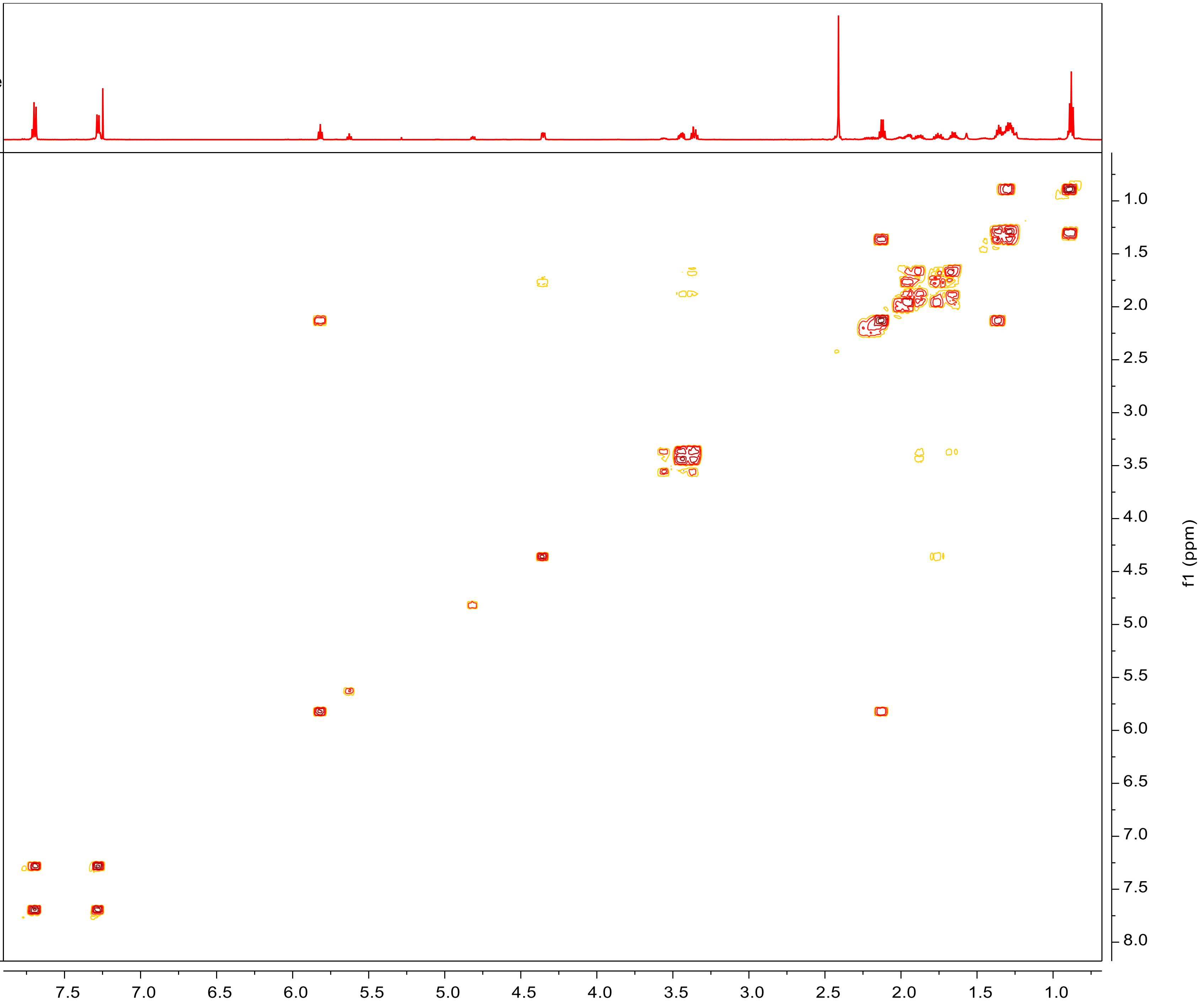

$^{13}C\{^1H\}$ NMR (150 MHz, $CDCl_3$)

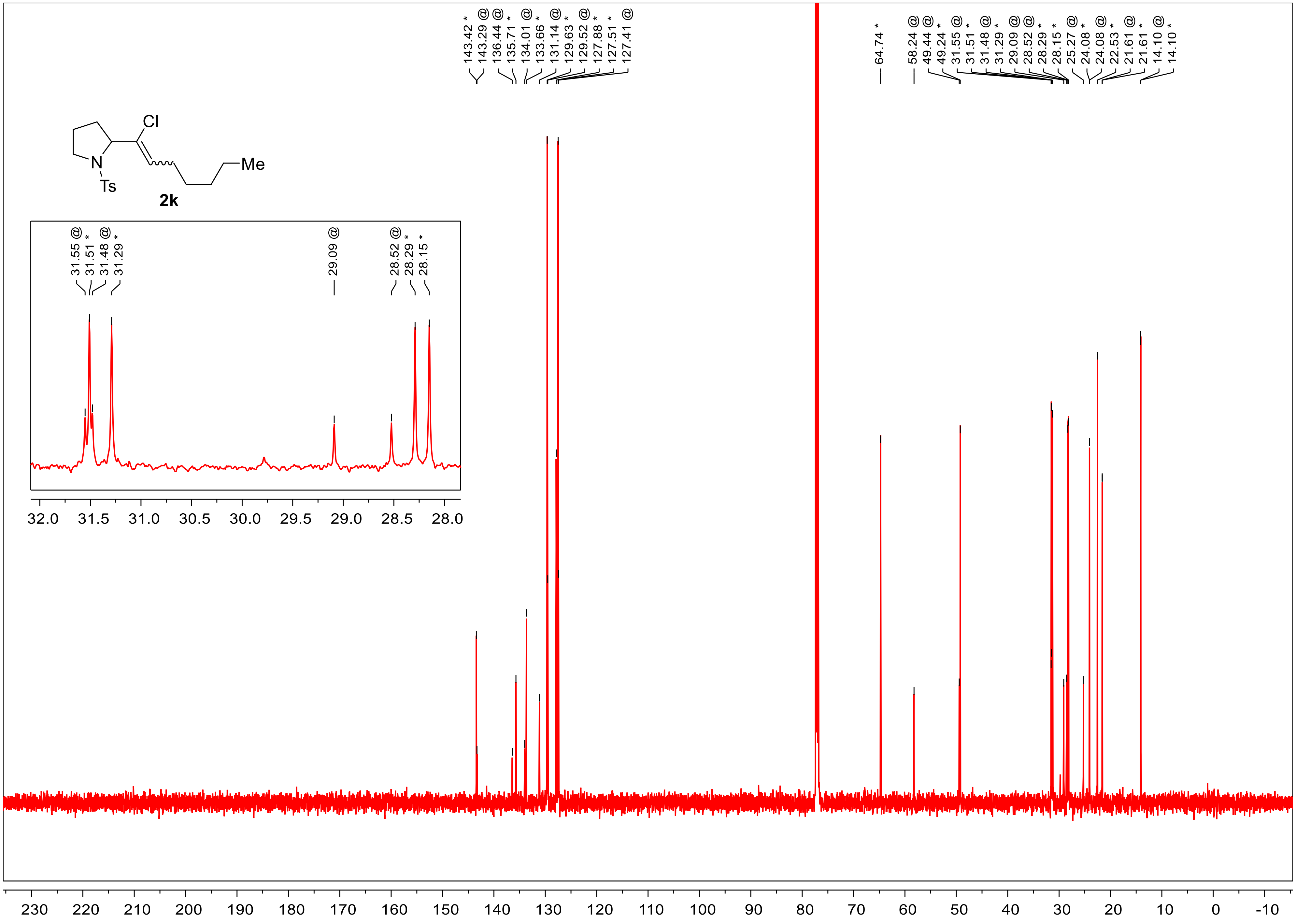

DEPT 135 (150 MHz, $CDCl_3$)

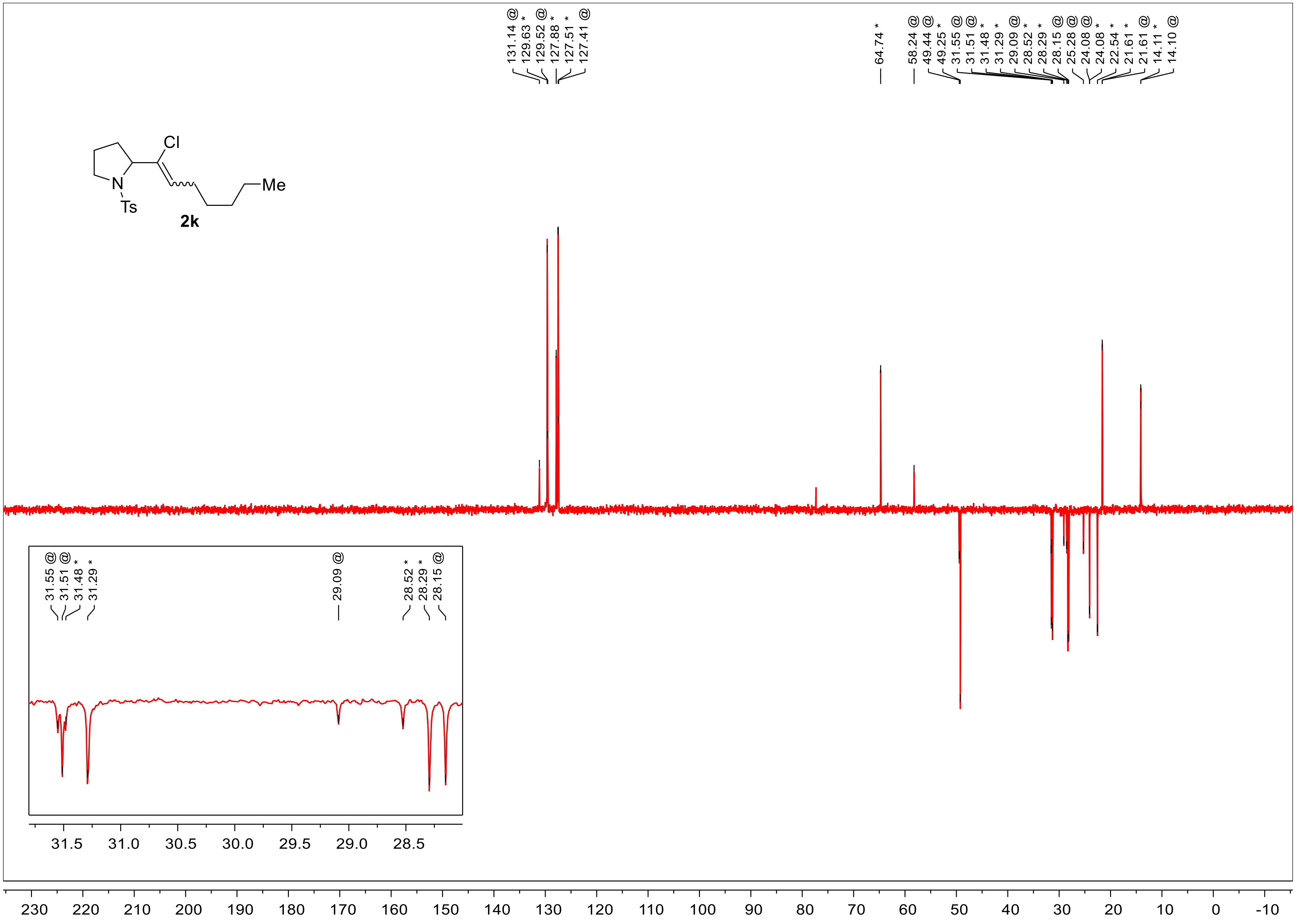

^{1}H-NMR (600 MHz, $CDCl_3$)

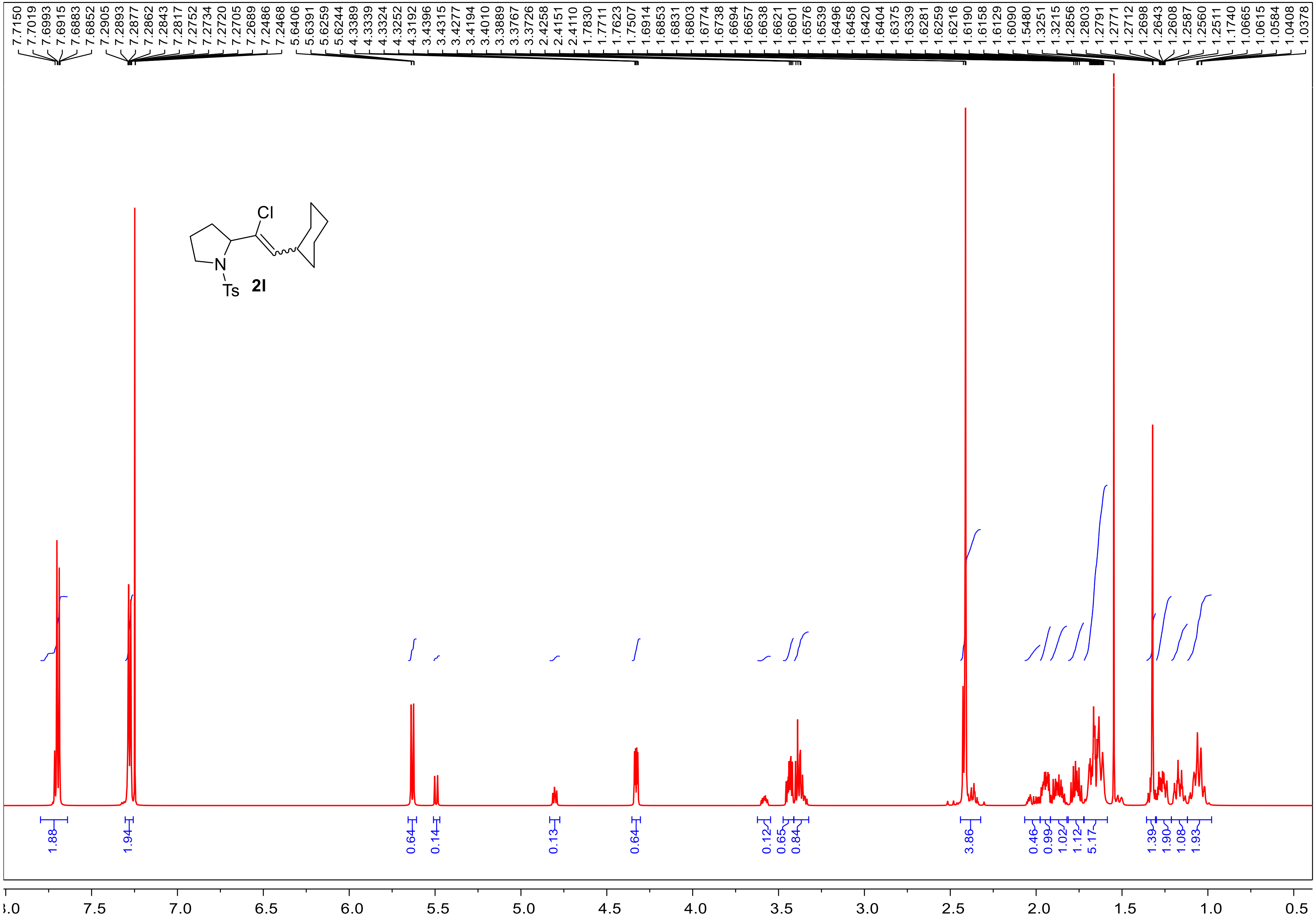

2D - COSY (600 MHz, $CDCl_3$)

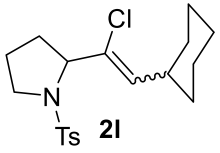

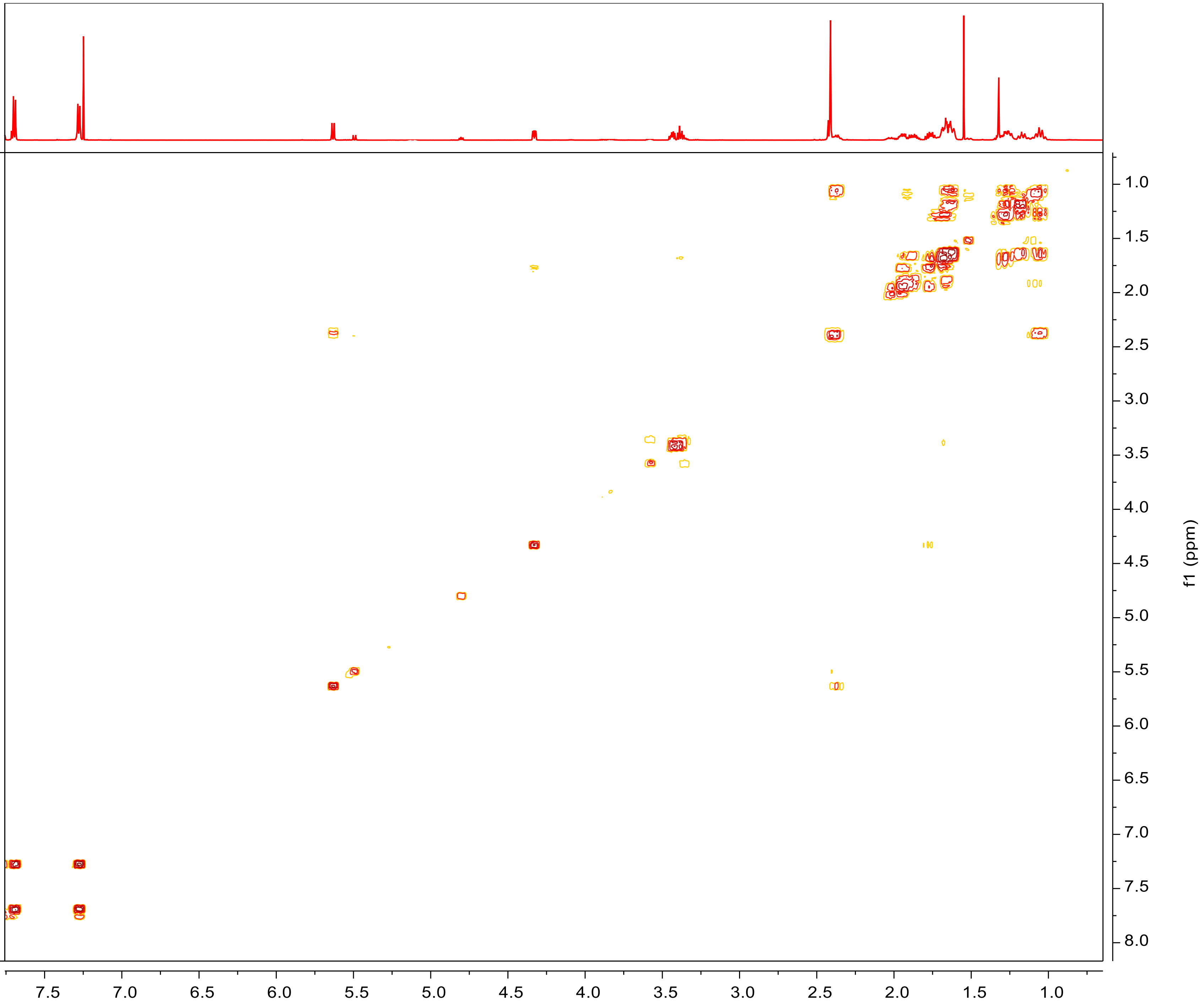

$^{13}C\{^1H\}$ **NMR** (150 MHz, $CDCl_3$)

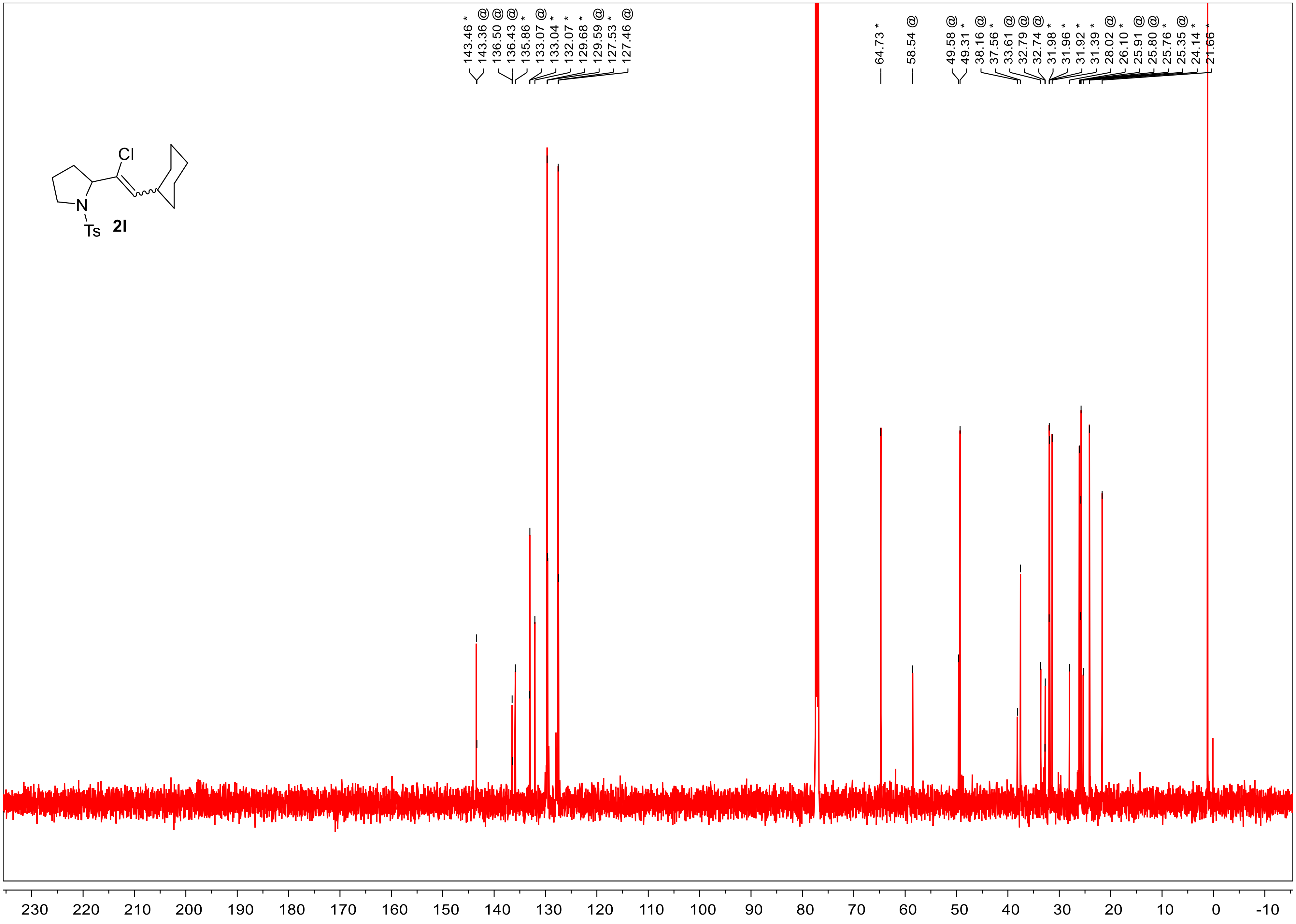

2l

DEPT 135 (150 MHz, $CDCl_3$)

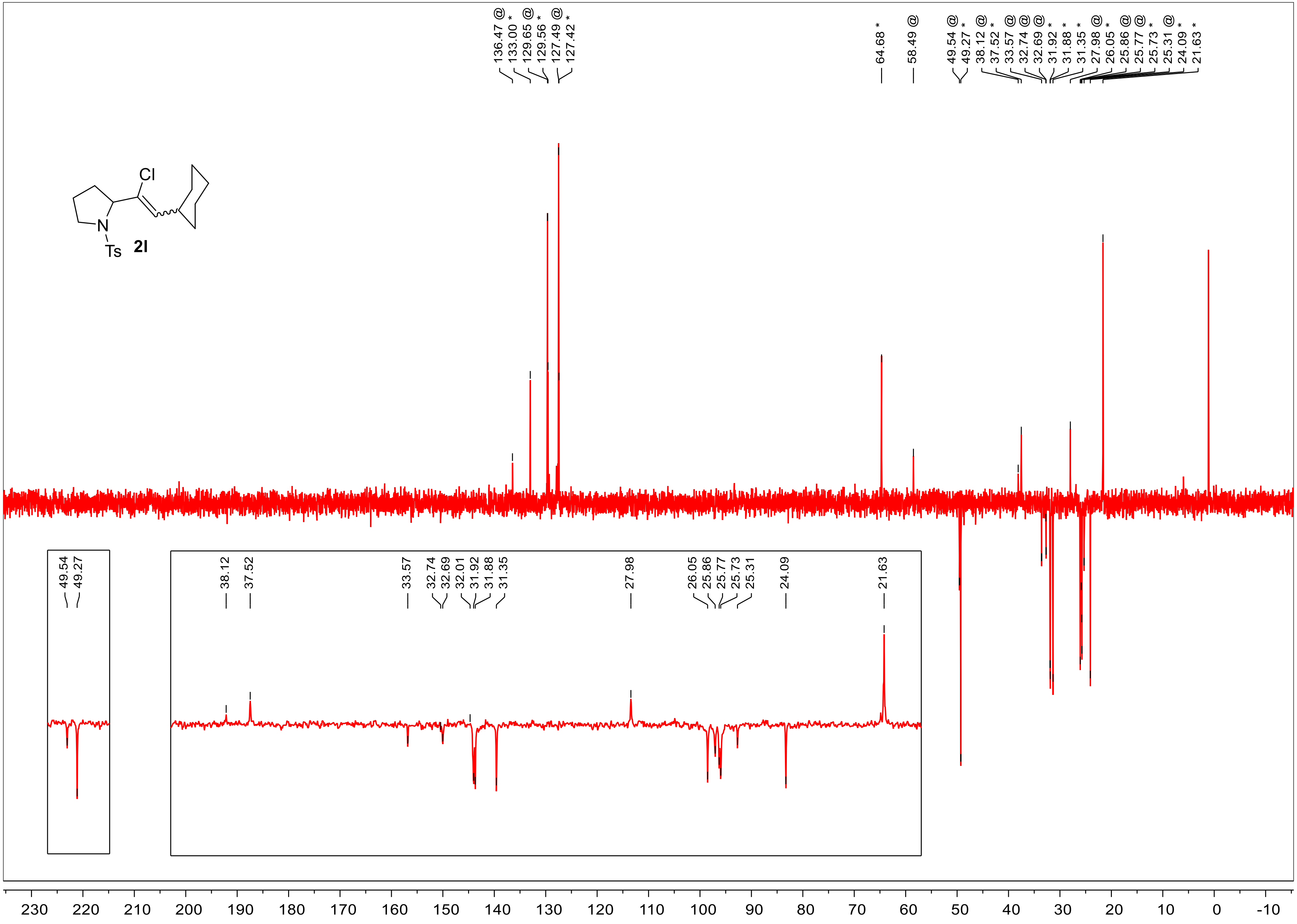

^{1}H-NMR (600 MHz, $CDCl_3$)

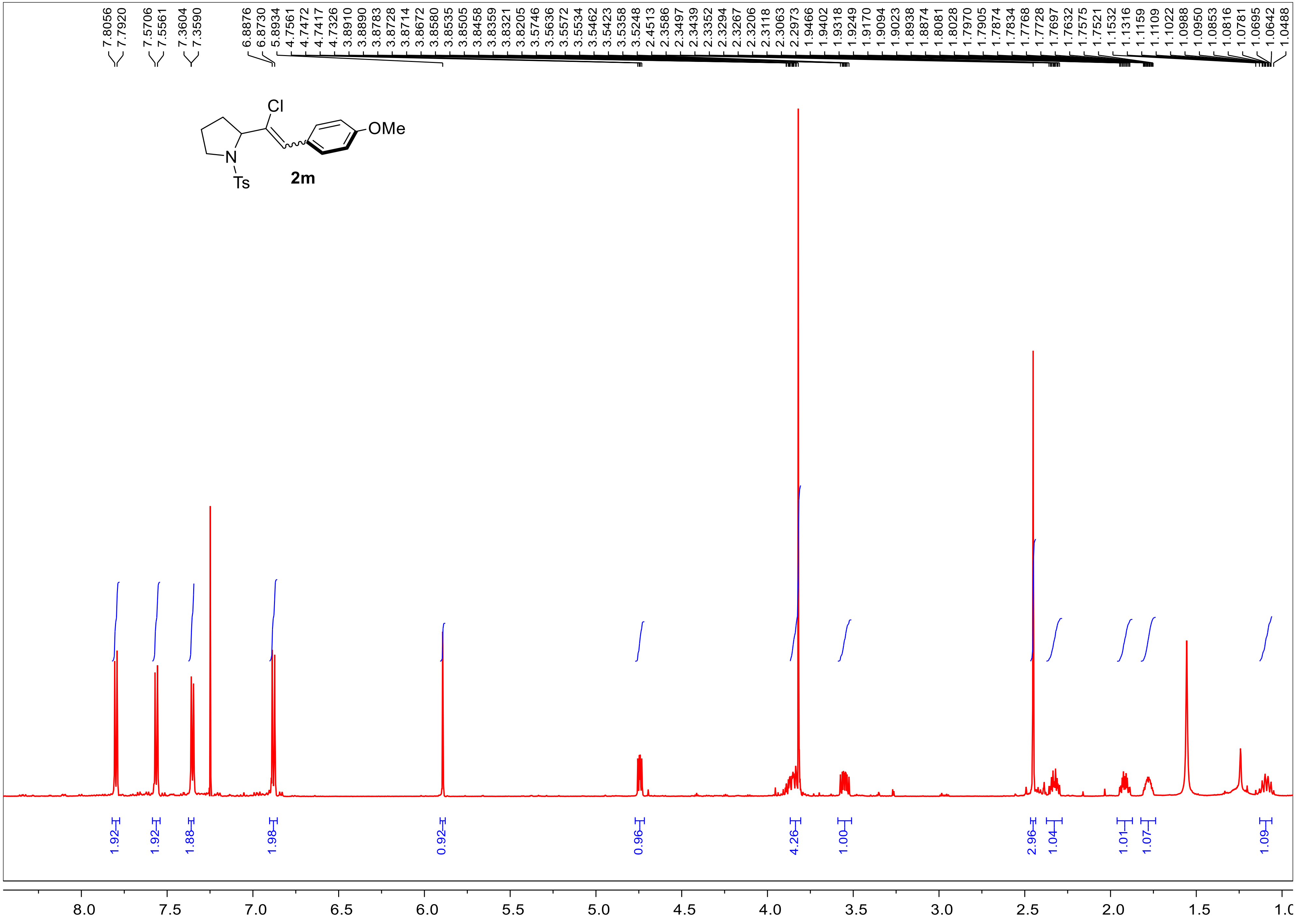

2D - COSY (600 MHz, $CDCl_3$)

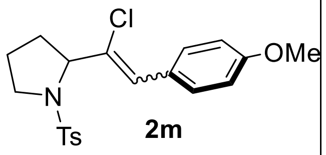

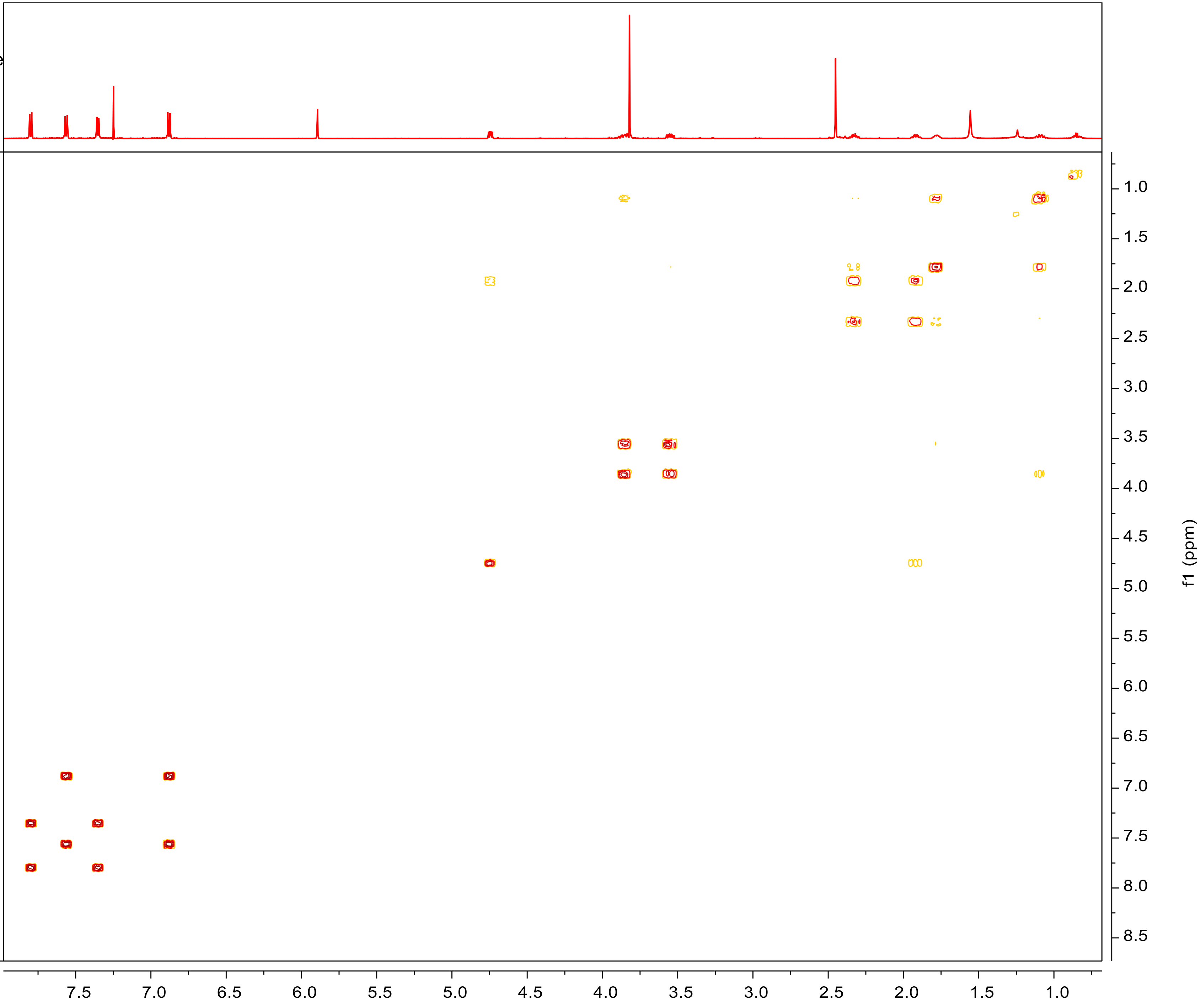

^{13}C{^{1}H} NMR (150 MHz, CDCl$_3$)

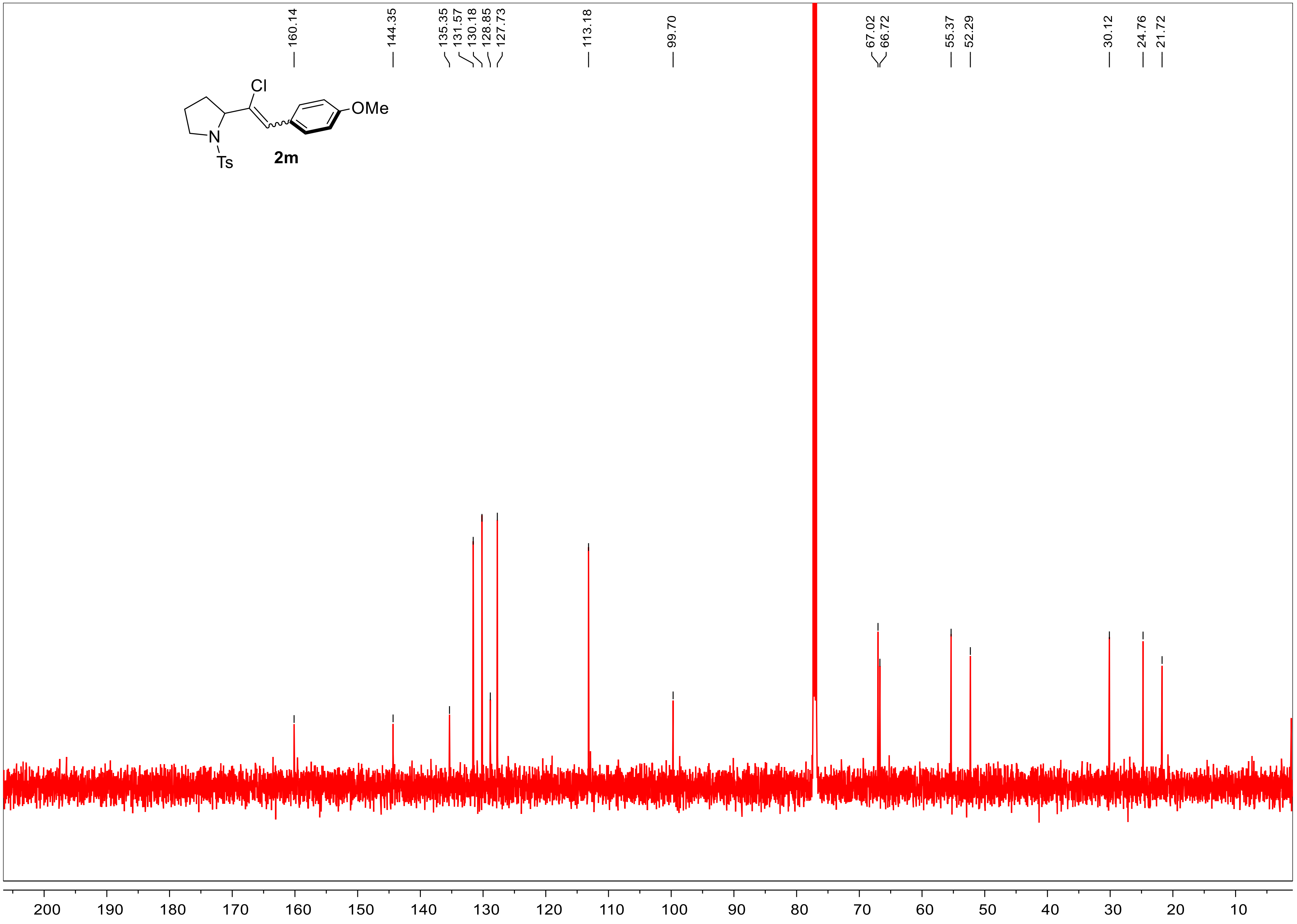

DEPT 135 (150 MHz, $CDCl_3$)

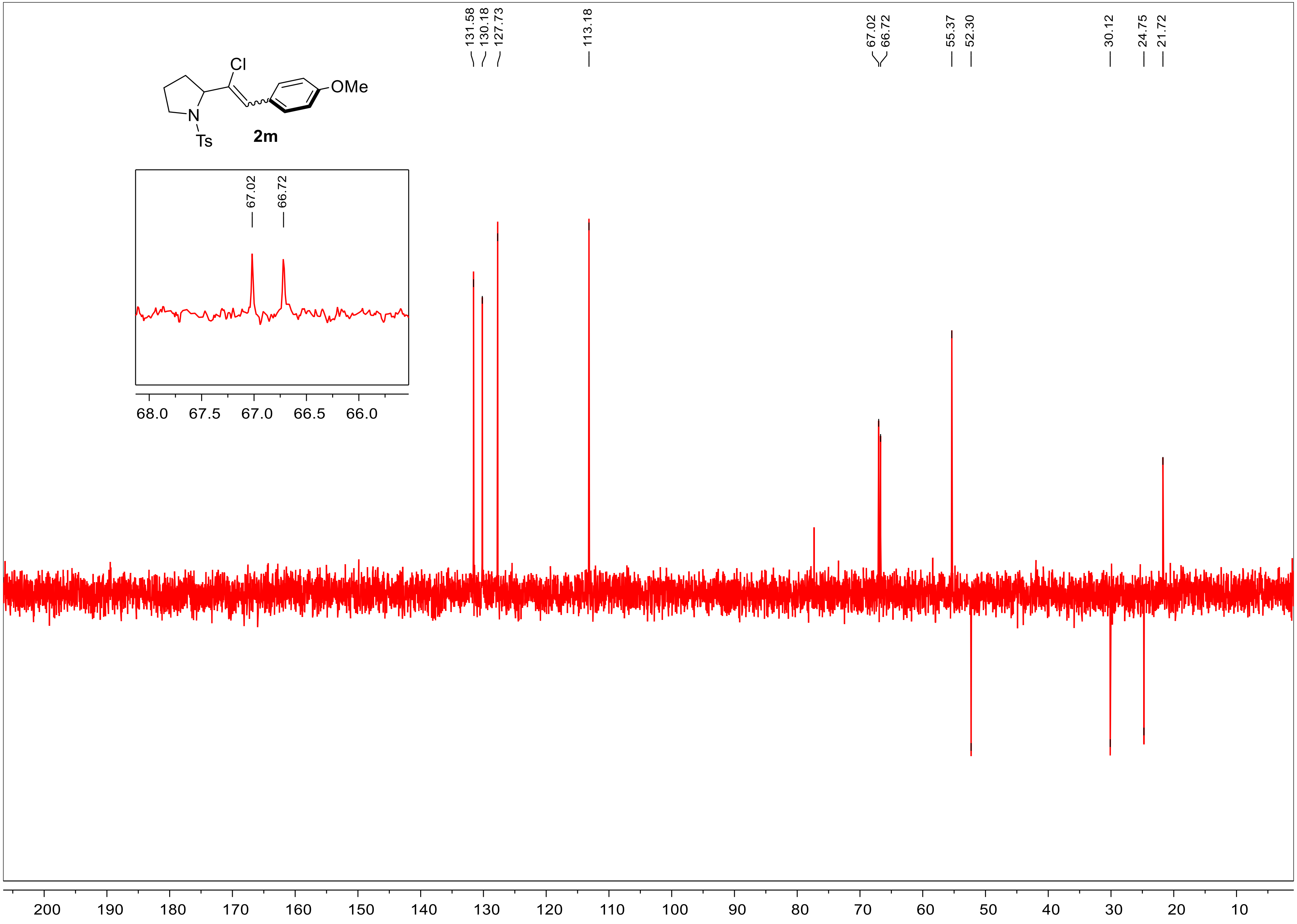

^{1}H-NMR (600 MHz, $CDCl_3$)

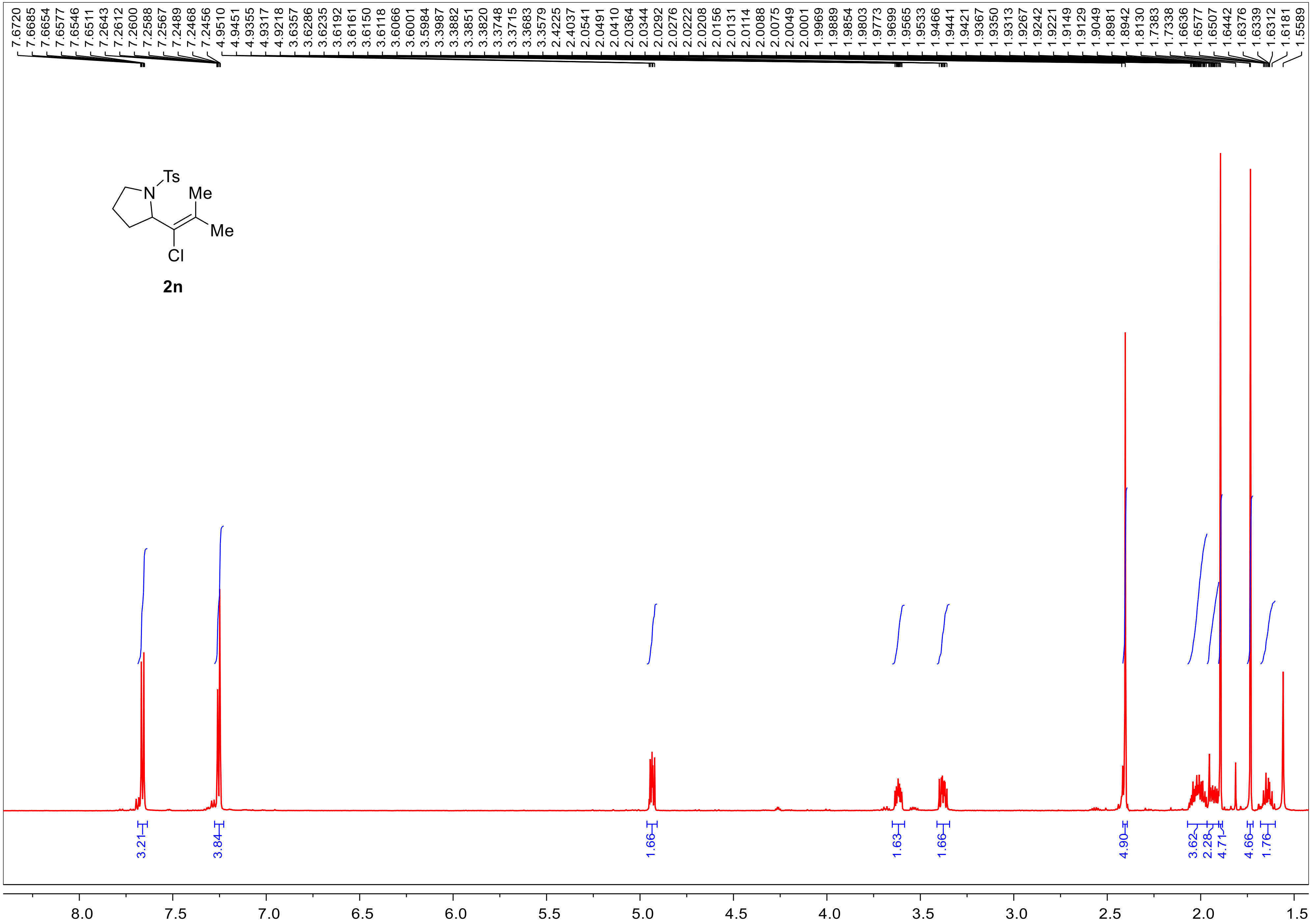

2D - COSY (600 MHz, $CDCl_3$)

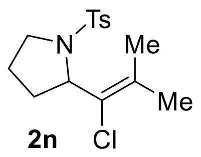

2n

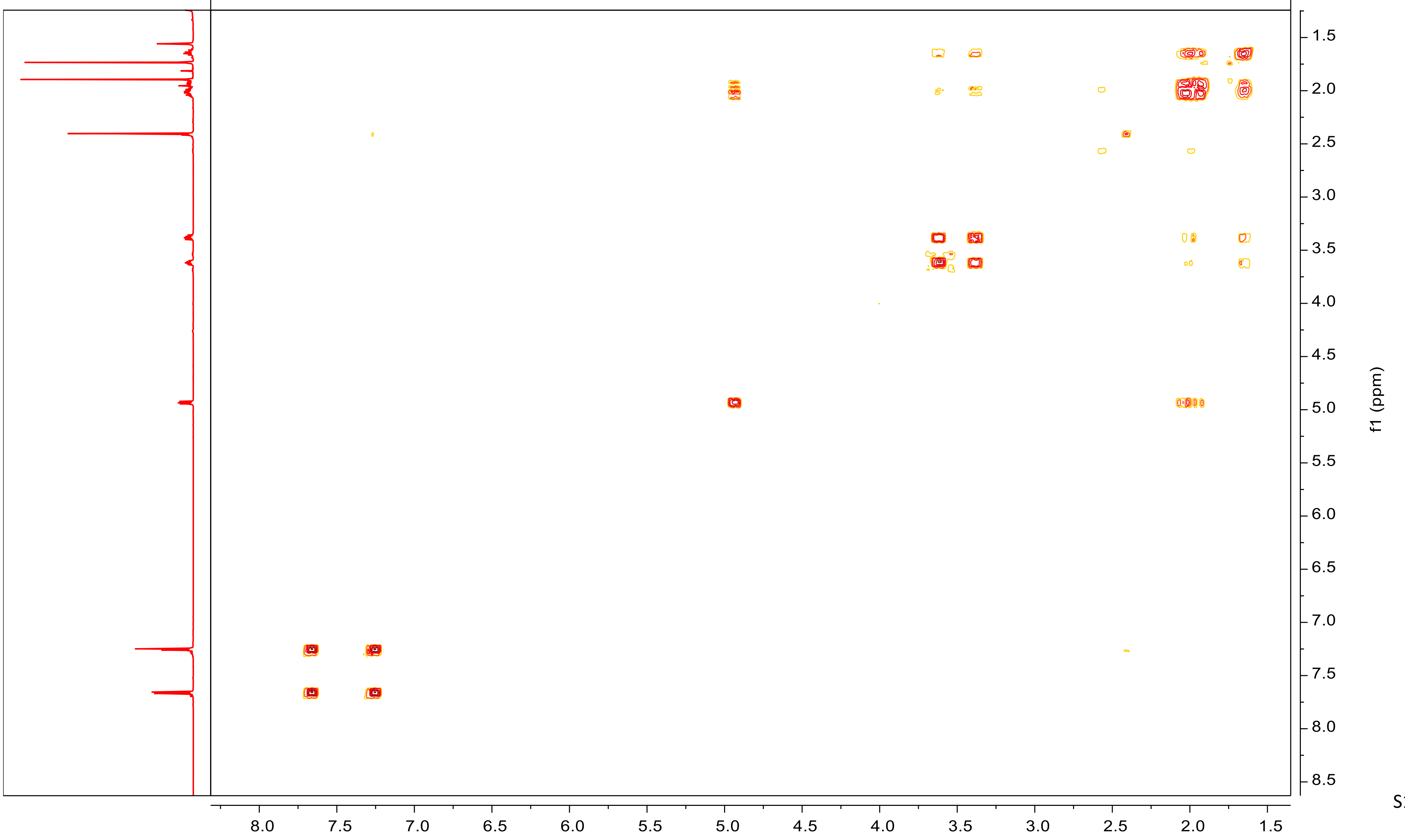

$^{13}C\{^{1}H\}$ NMR (150 MHz, $CDCl_3$)

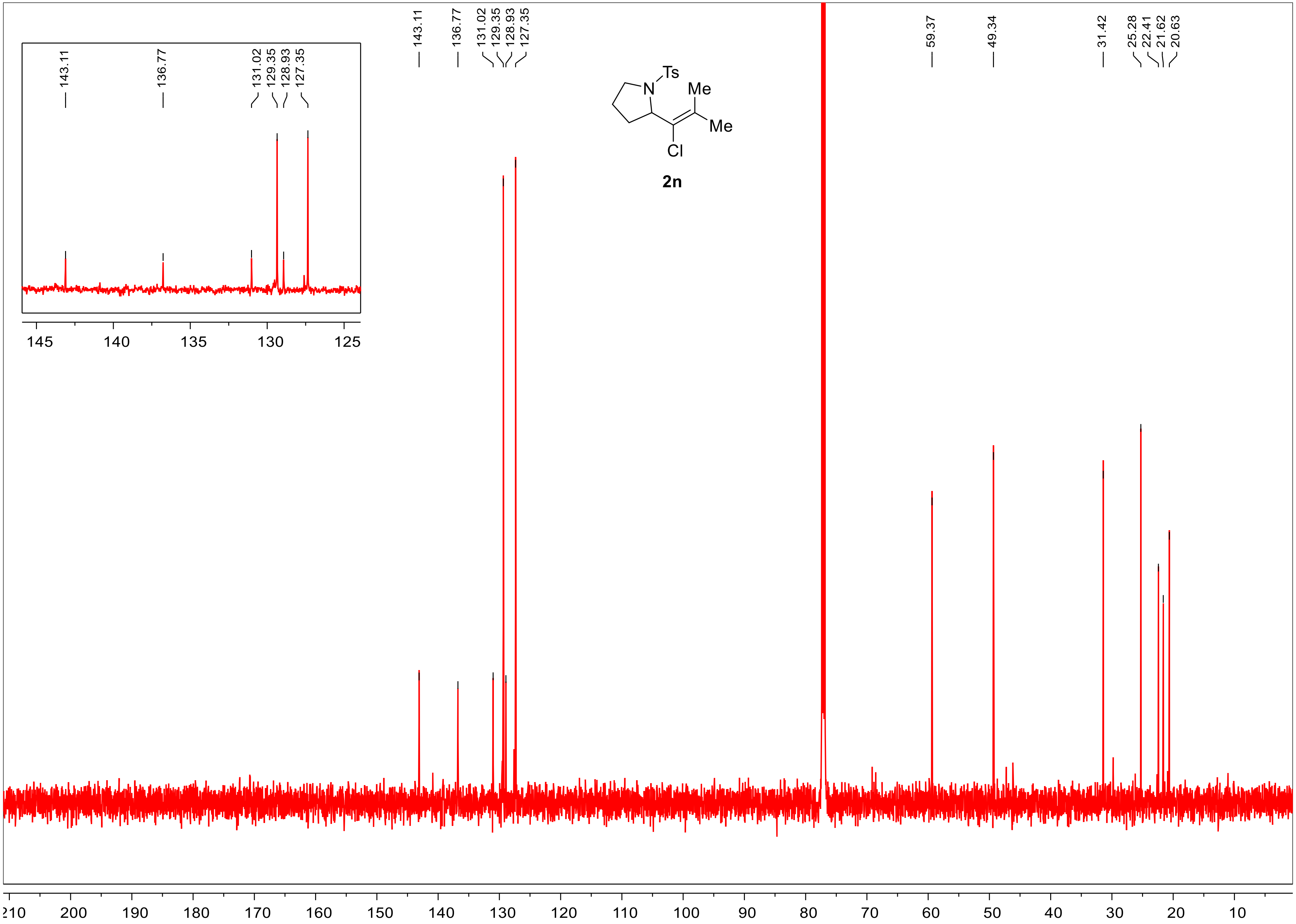

DEPT 135 (150 MHz, $CDCl_3$)

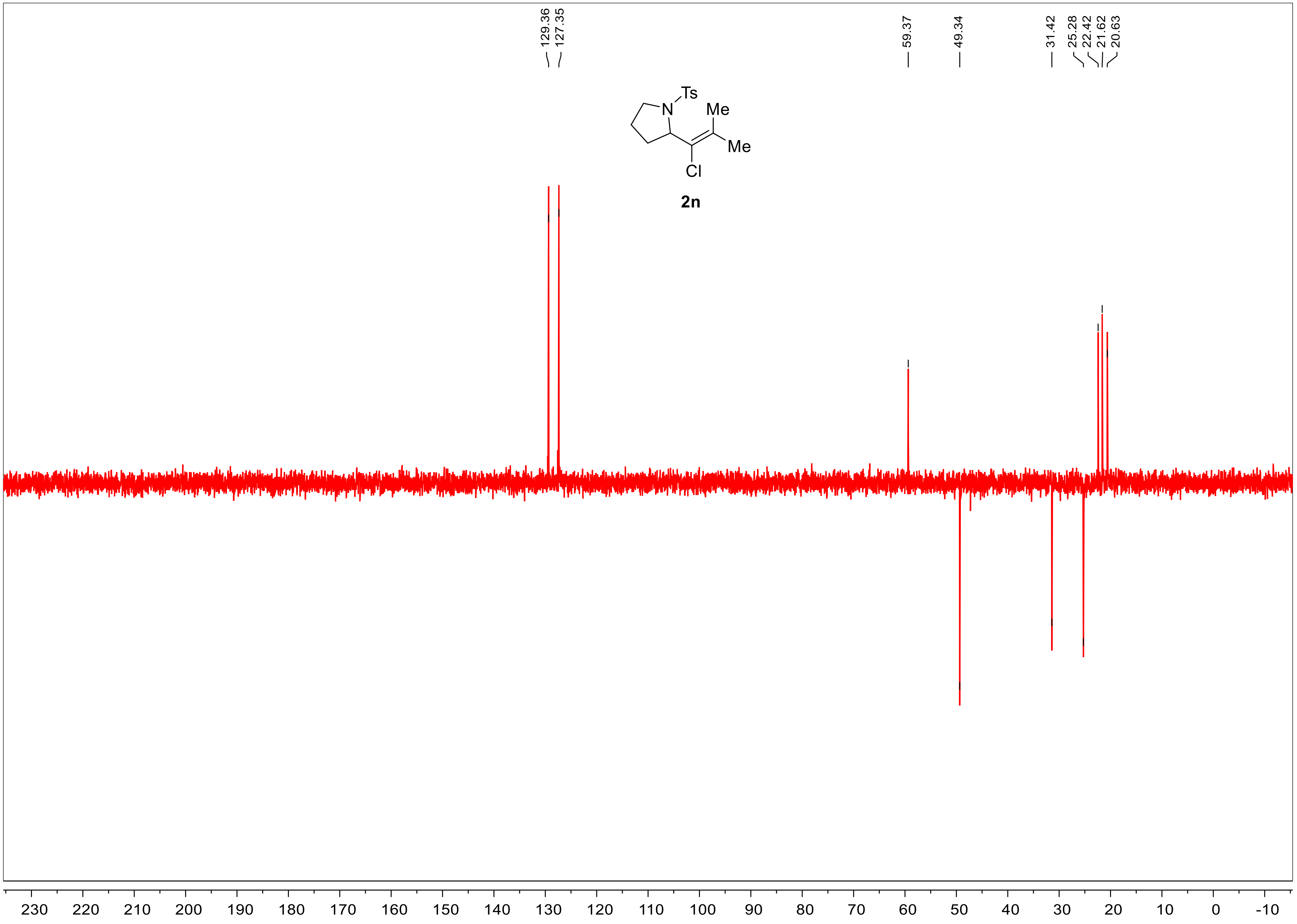

^{1}H-NMR (600 MHz, $CDCl_3$)

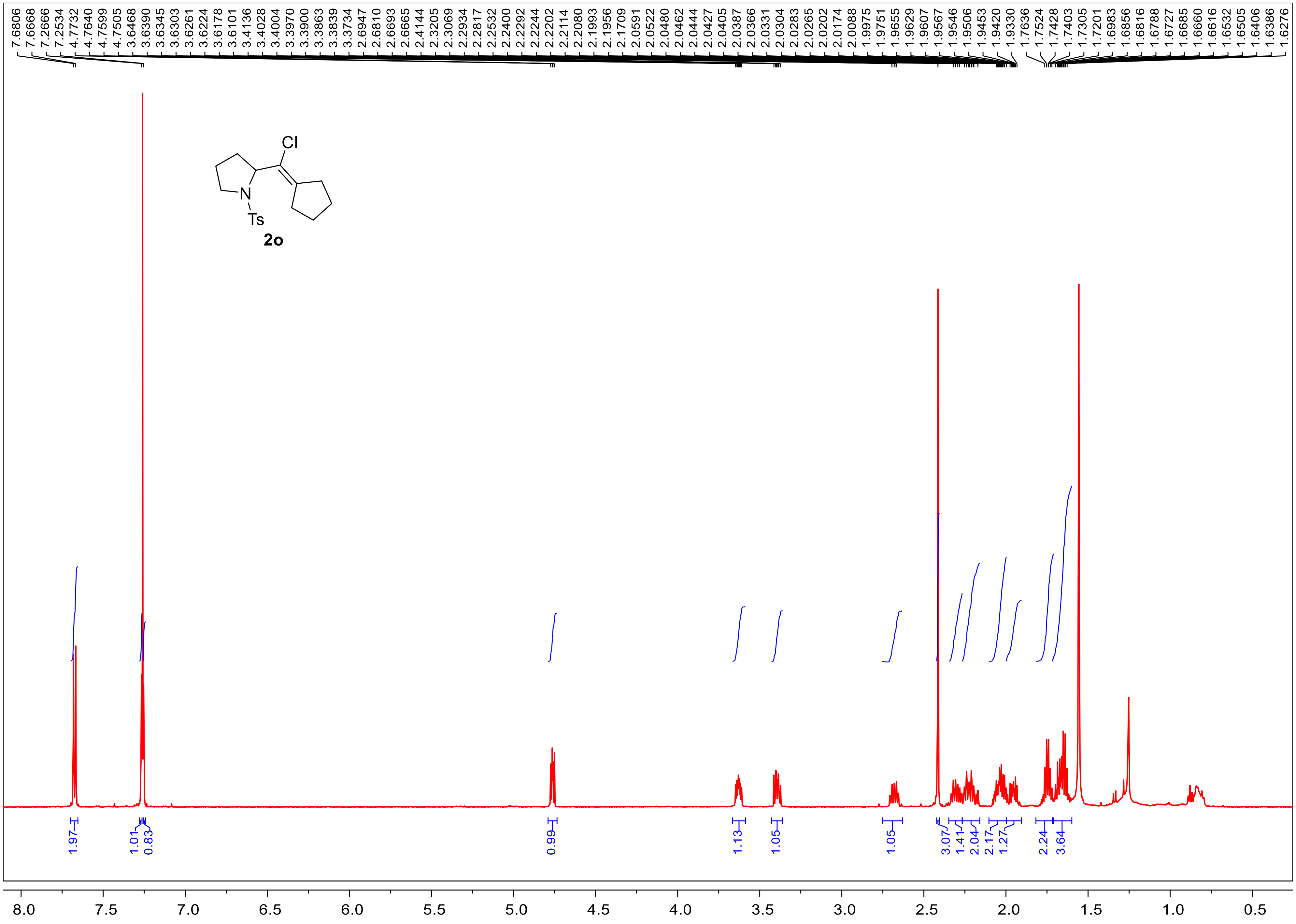

2D - COSY (600 MHz, $CDCl_3$)

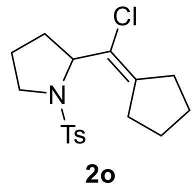

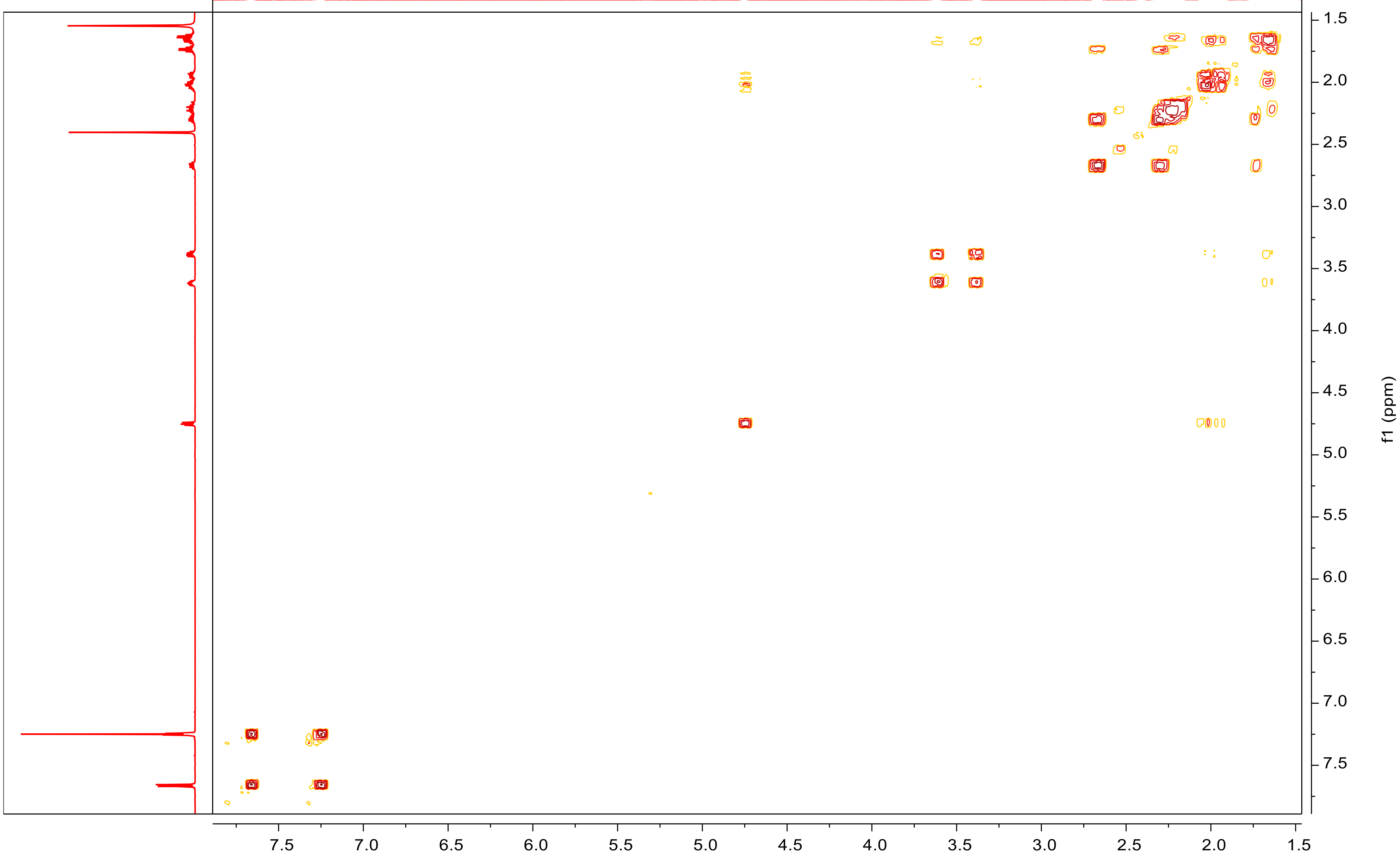

$^{13}C\{^{1}H\}$ **NMR** (150 MHz, $CDCl_3$)

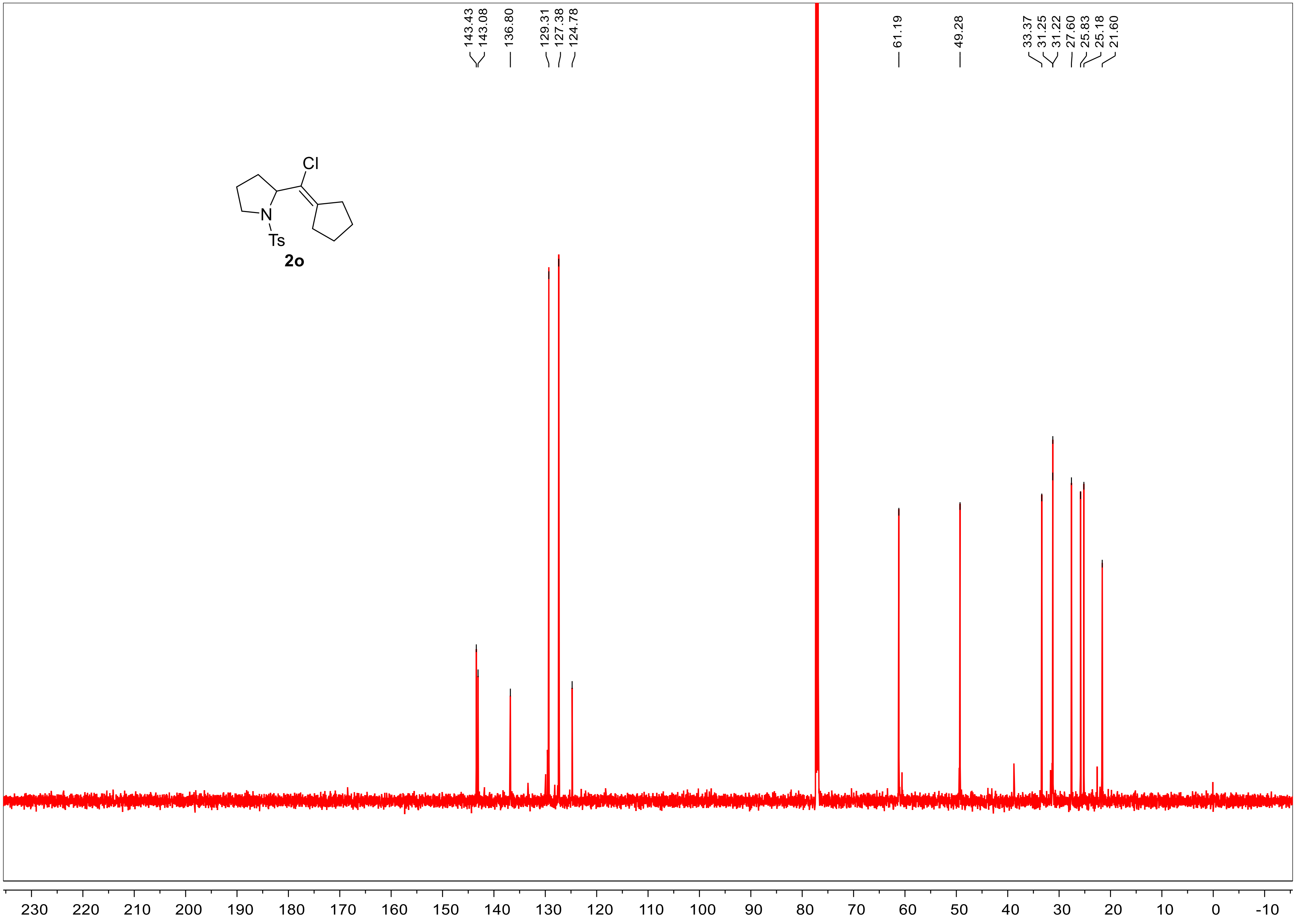

DEPT 135 (150 MHz, $CDCl_3$)

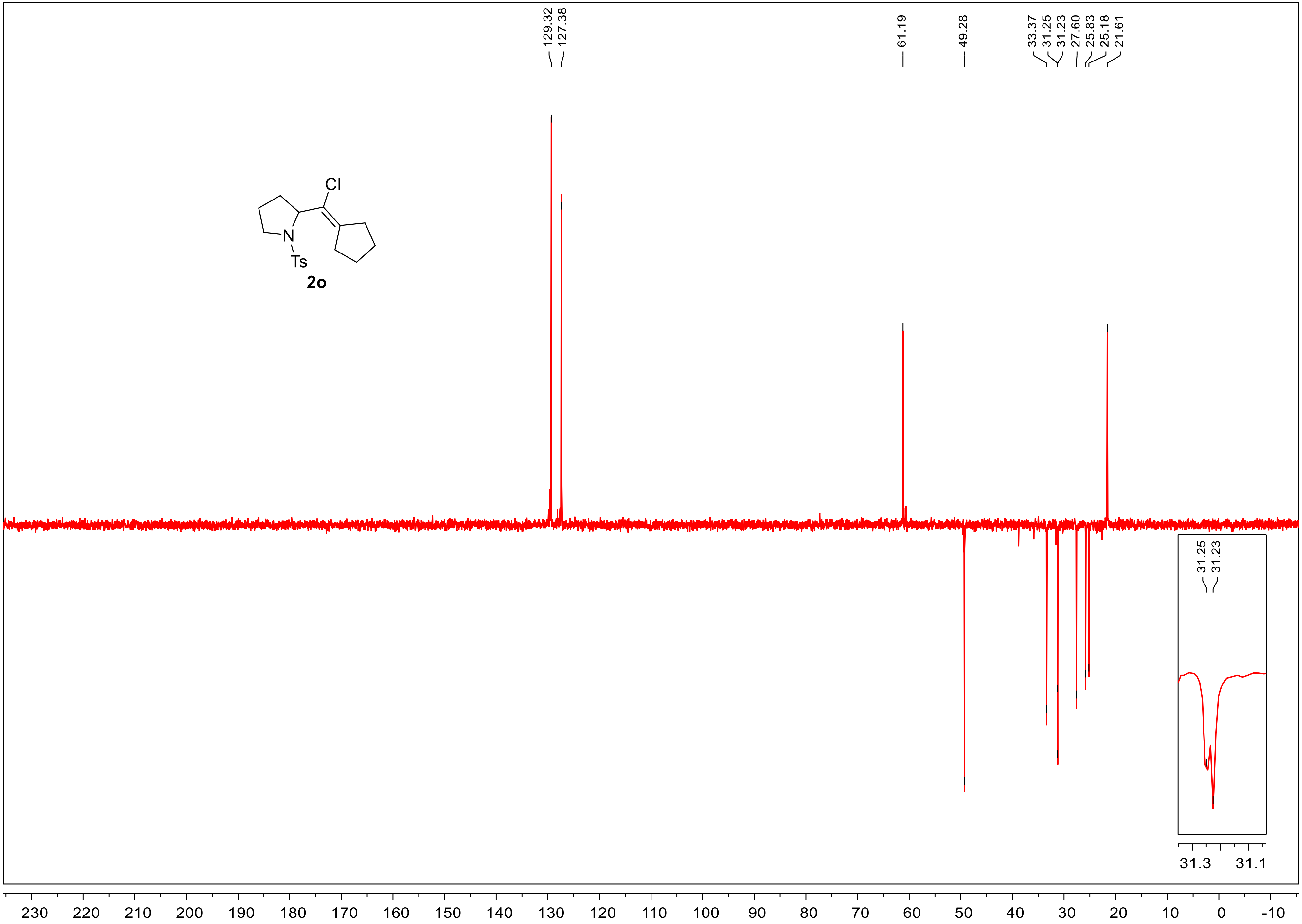

^{1}H-NMR (600 MHz, $CDCl_3$)

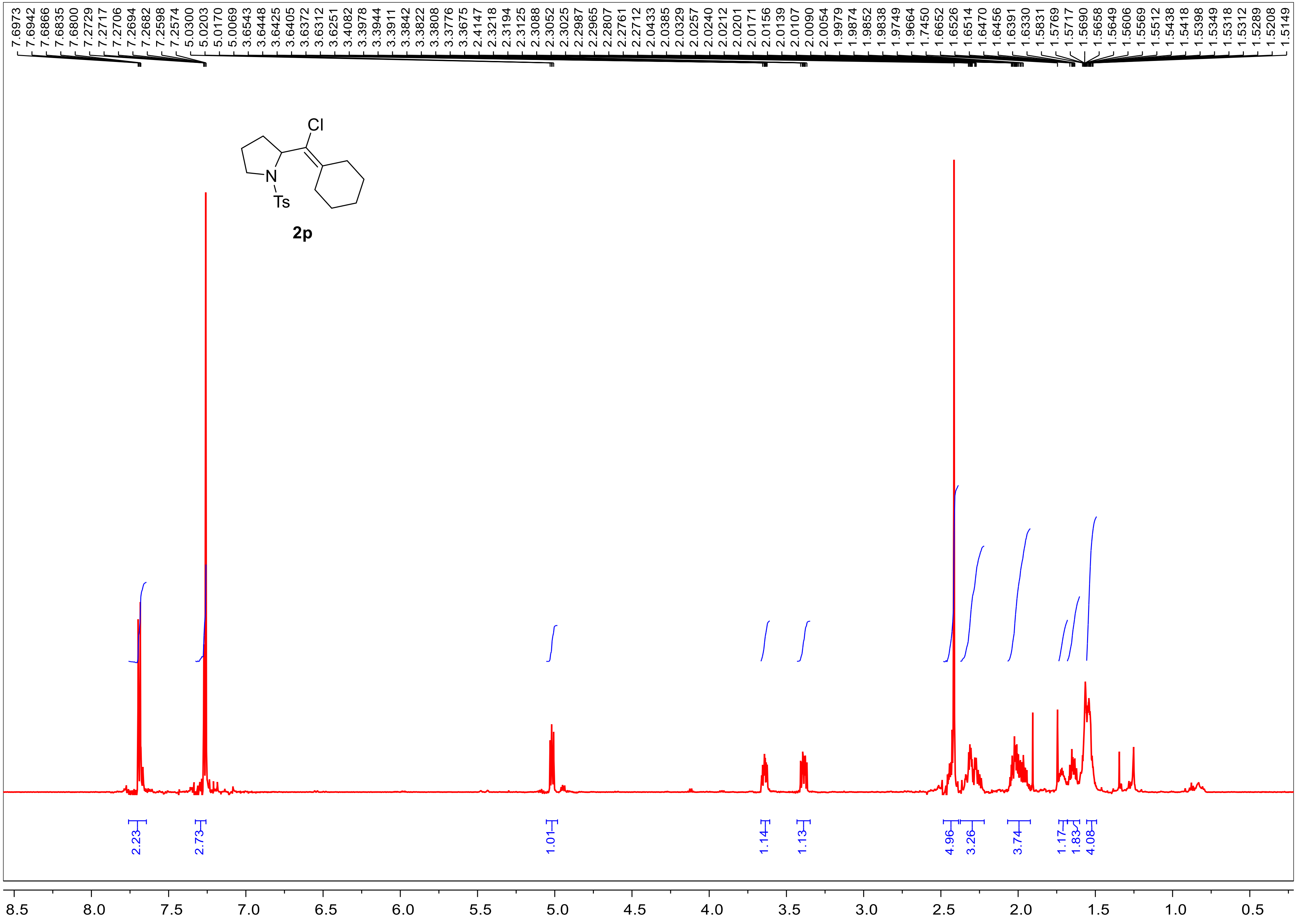

2D - COSY (600 MHz, $CDCl_3$)

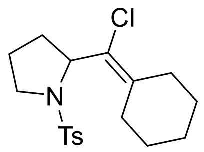

2p

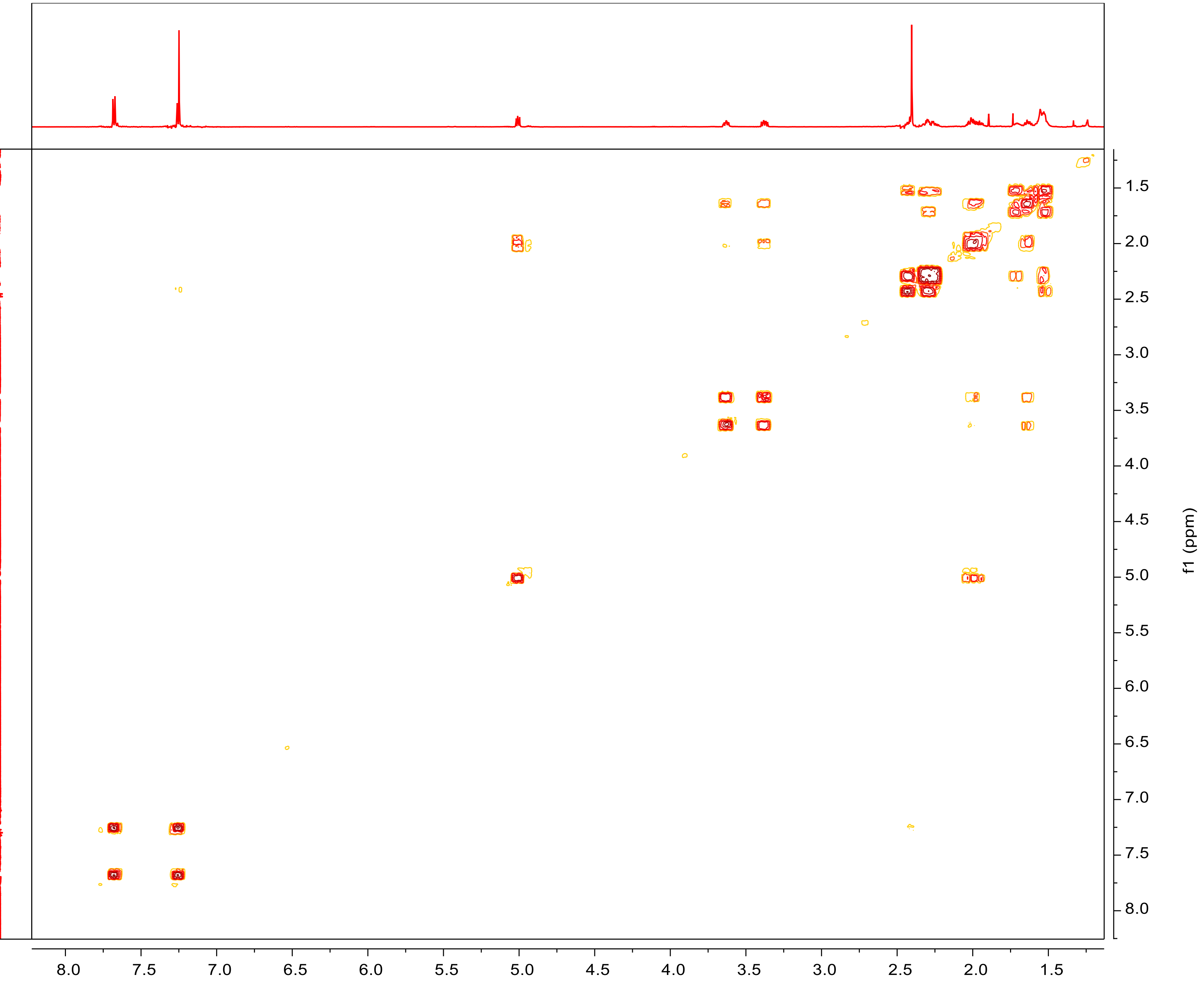

$^{13}C\{^{1}H\}$ NMR (150 MHz, $CDCl_3$)

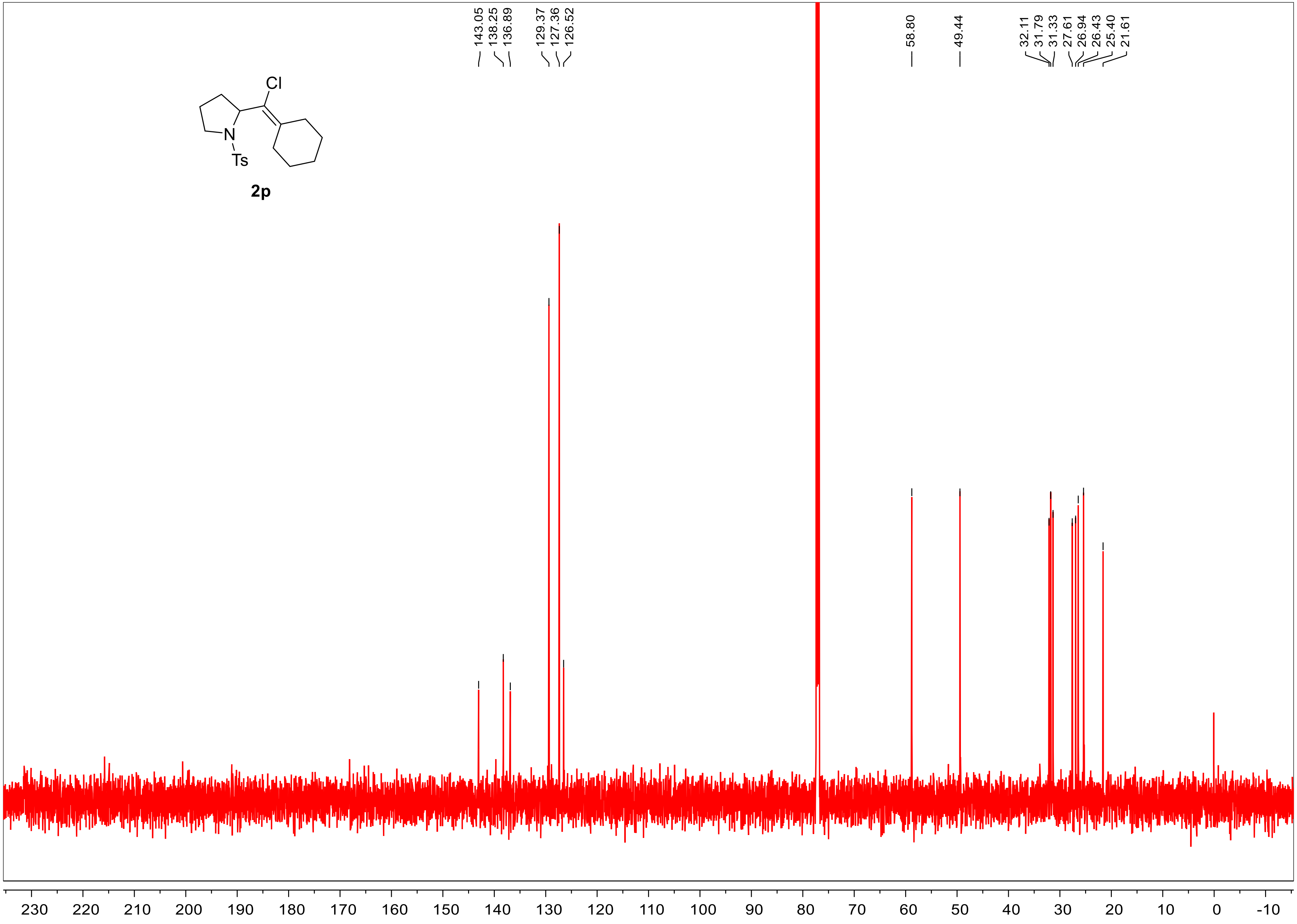

2p

DEPT 135 (150 MHz, $CDCl_3$)

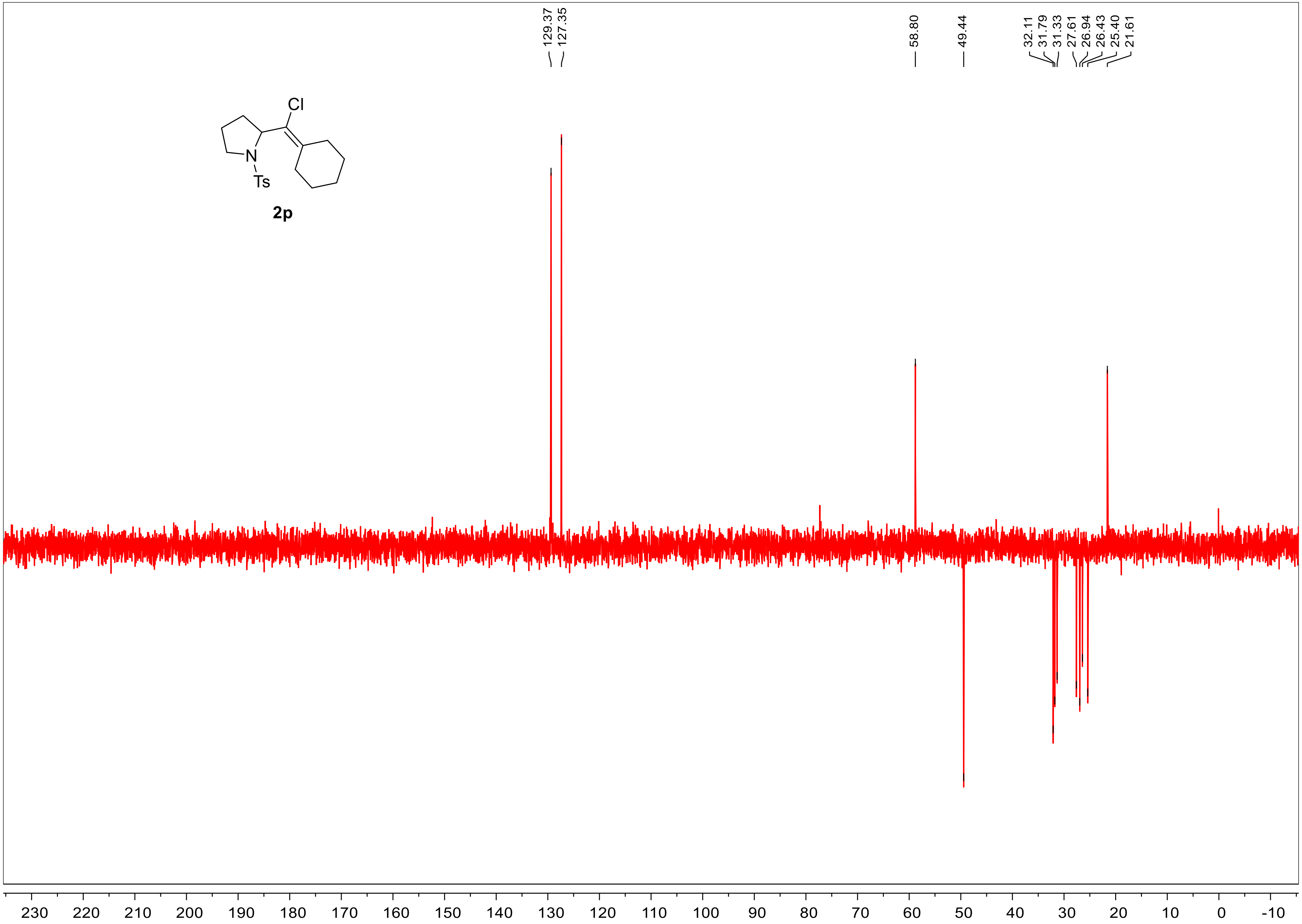

2p

^{1}H-NMR (600 MHz, $CDCl_3$)

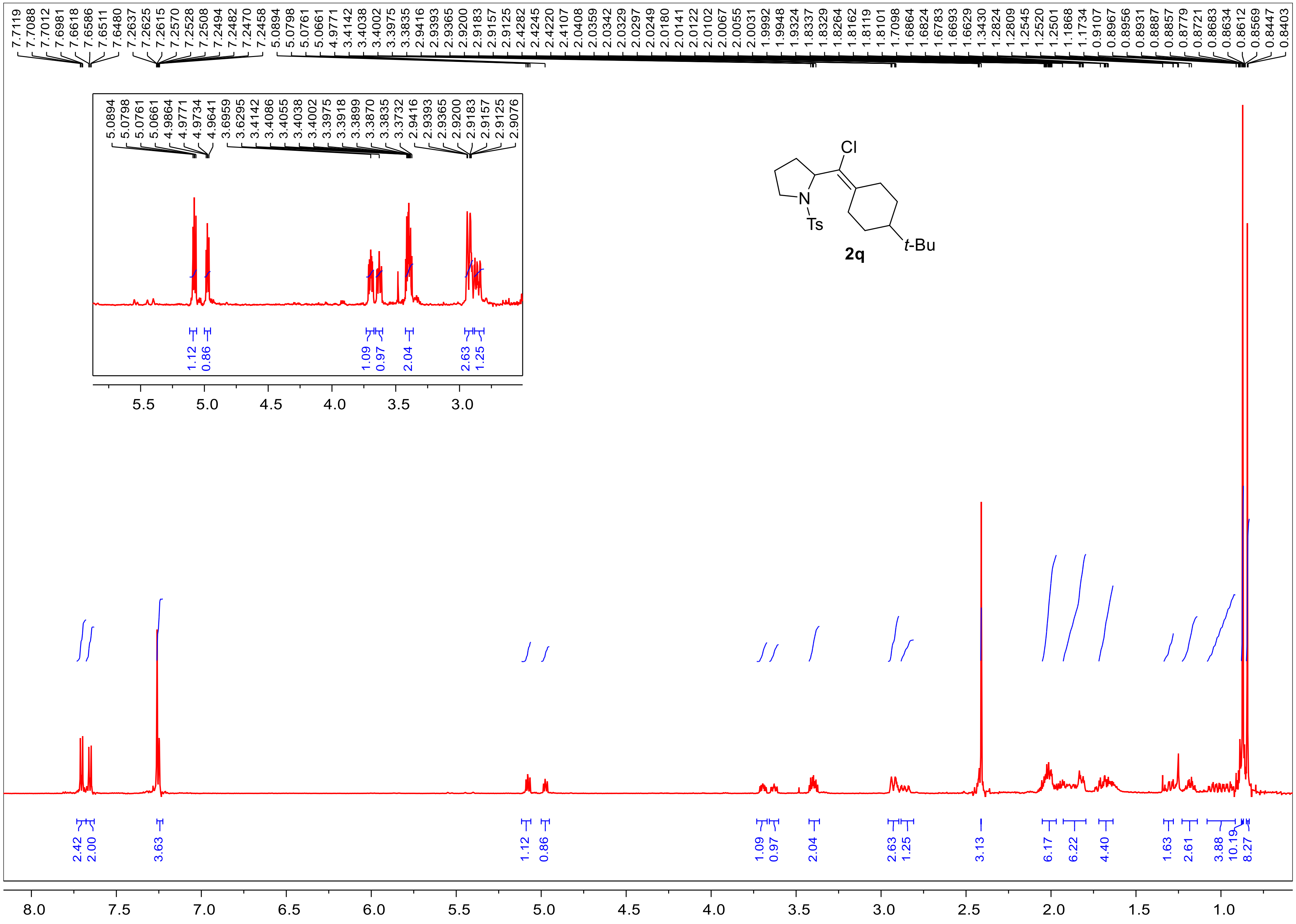

2D - COSY (600 MHz, $CDCl_3$)

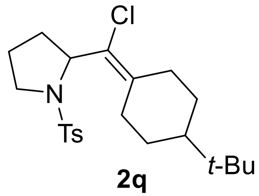

2q

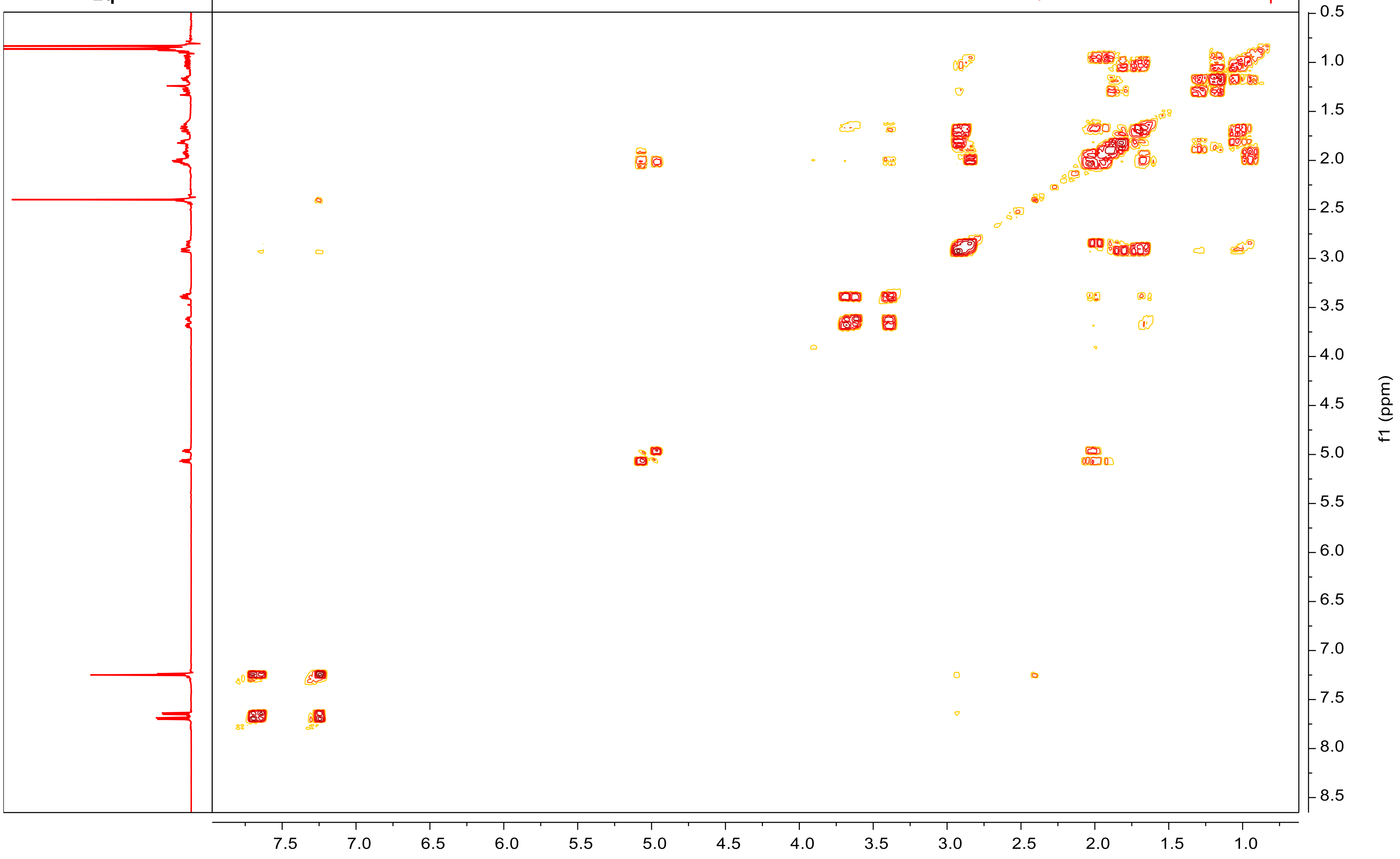

$^{13}C\{^1H\}$ NMR (150 MHz, $CDCl_3$)

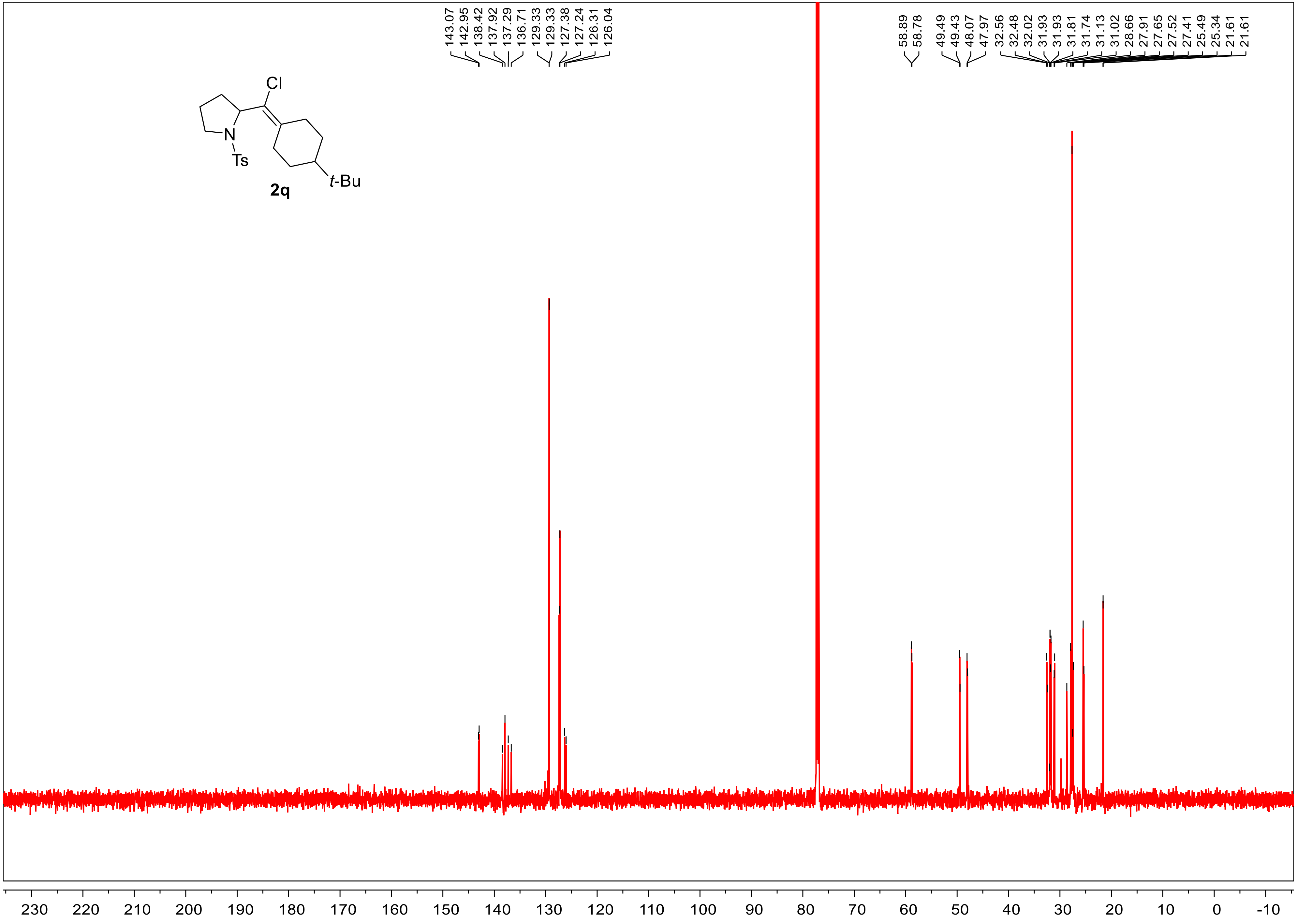

DEPT 135 (150 MHz, $CDCl_3$)

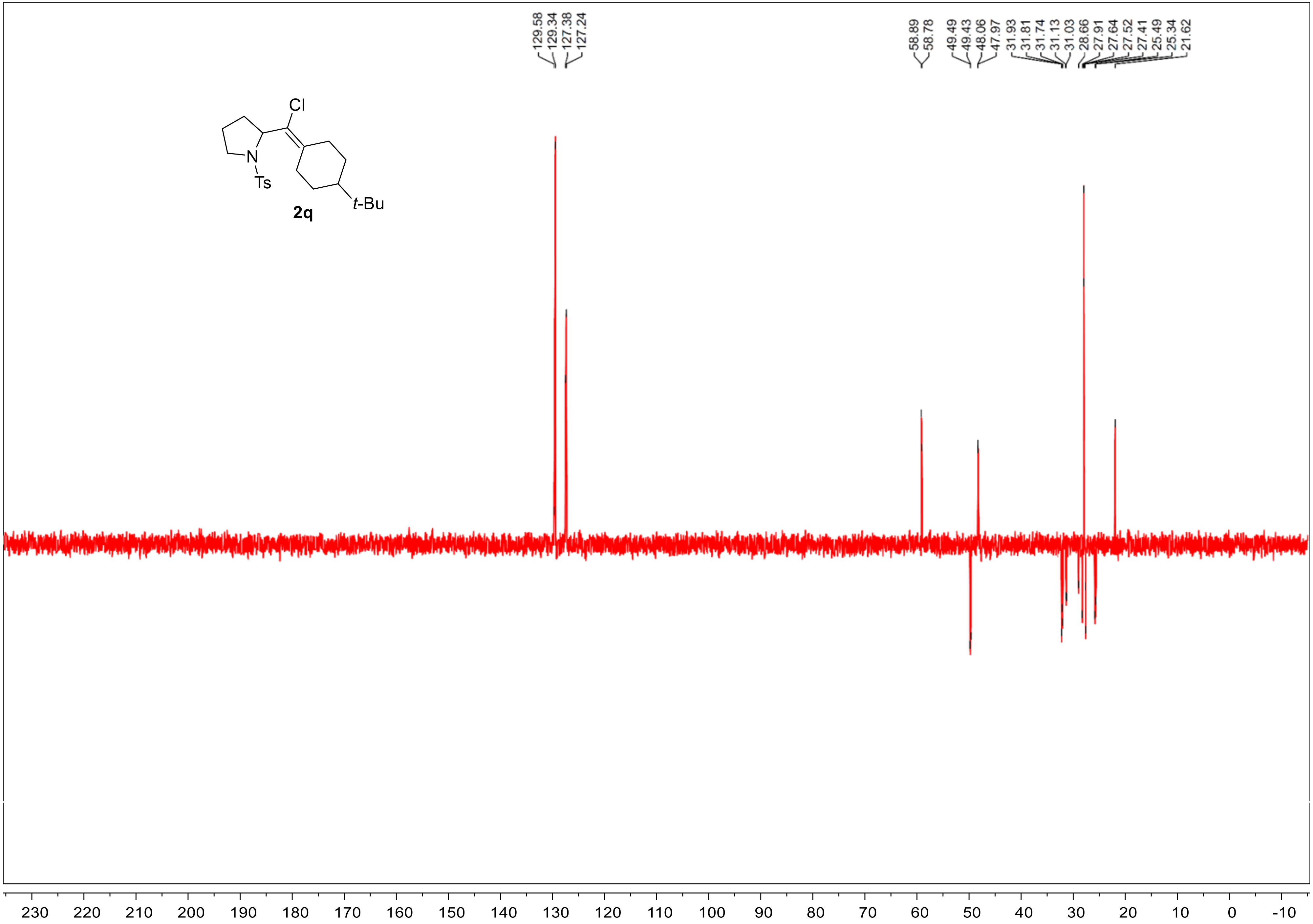

^{1}H-NMR (600 MHz, $CDCl_3$)

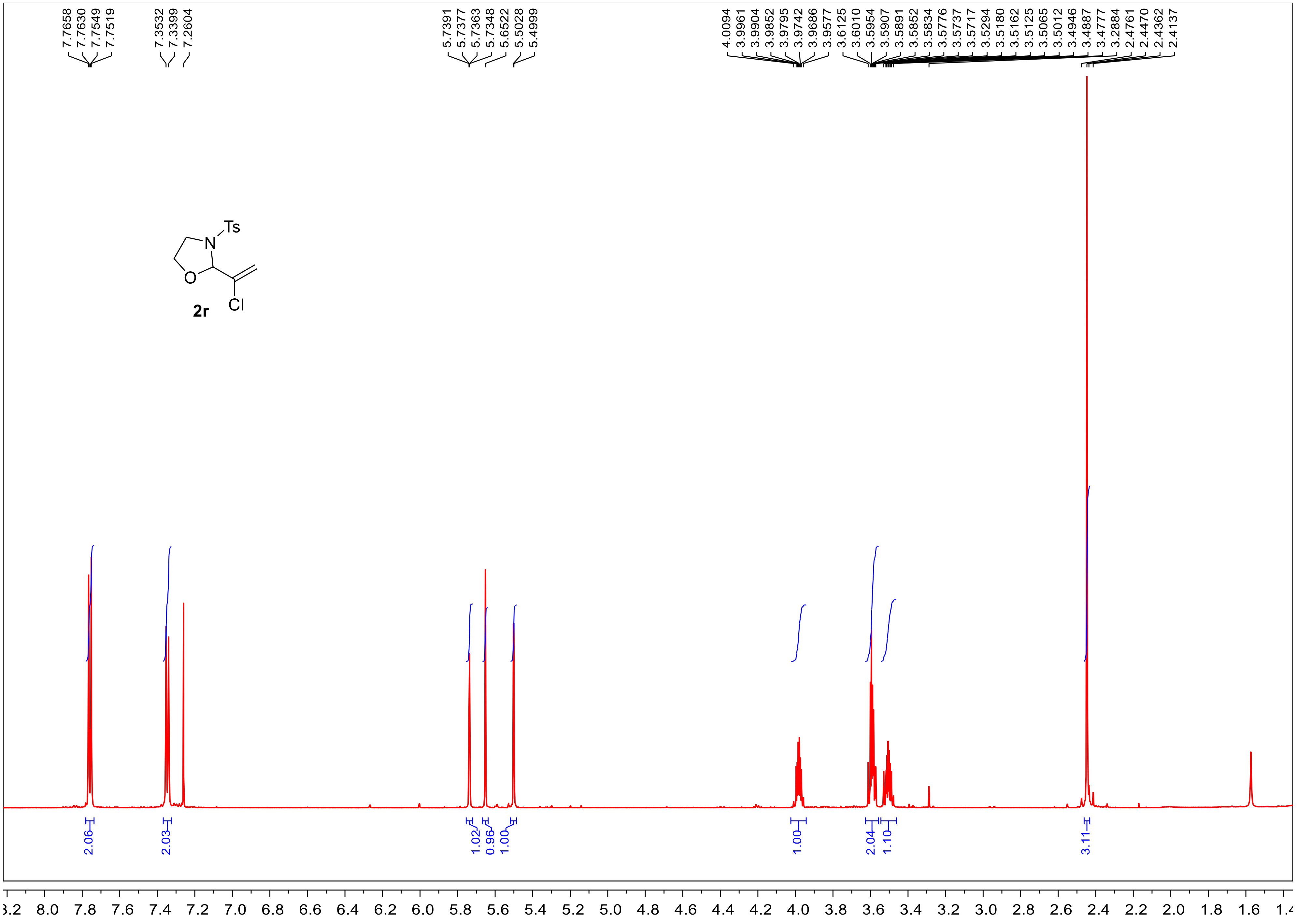

2D - COSY (600 MHz, $CDCl_3$)

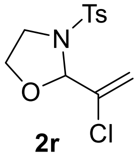

2r

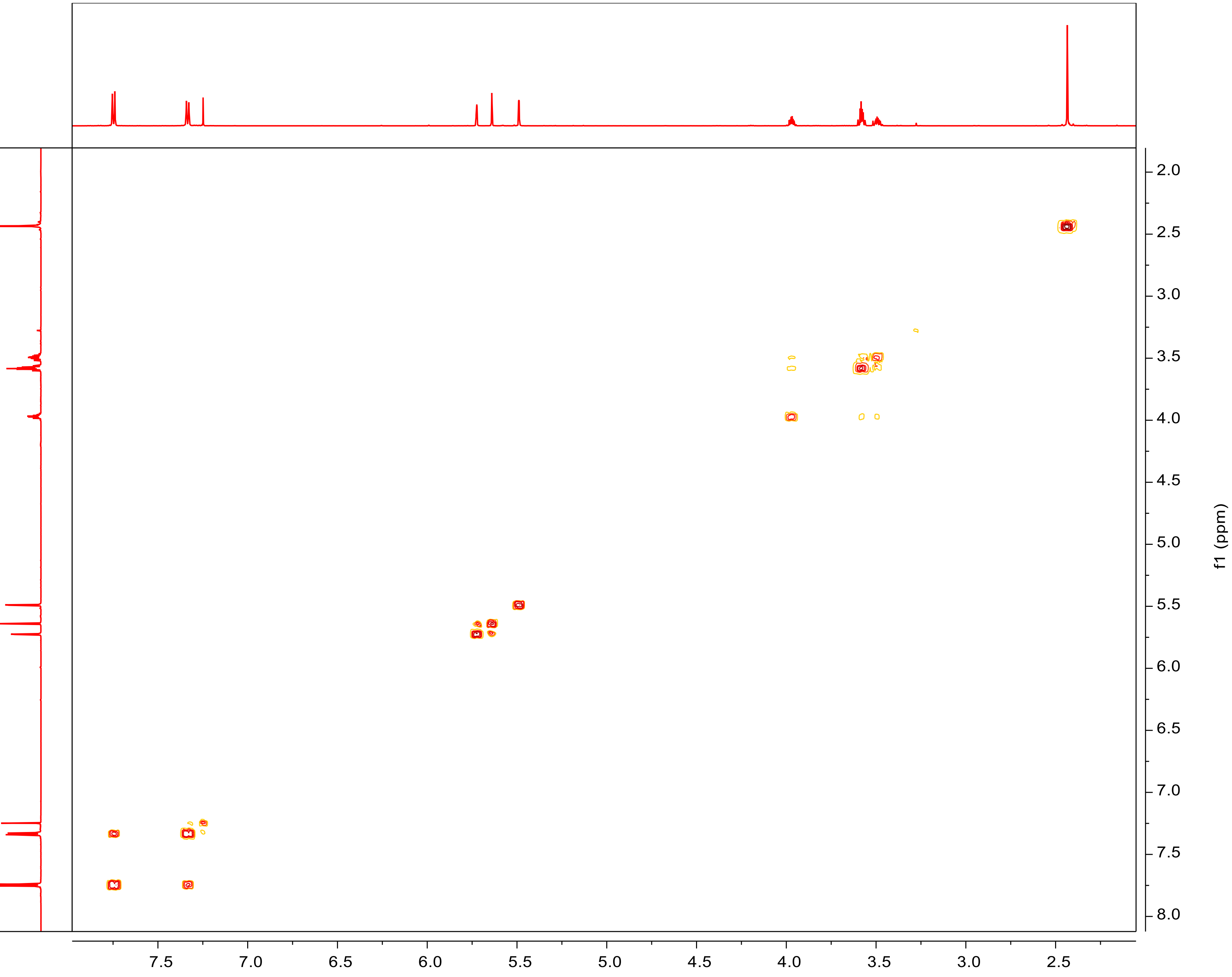

$^{13}C\{^1H\}$ NMR (150 MHz, $CDCl_3$)

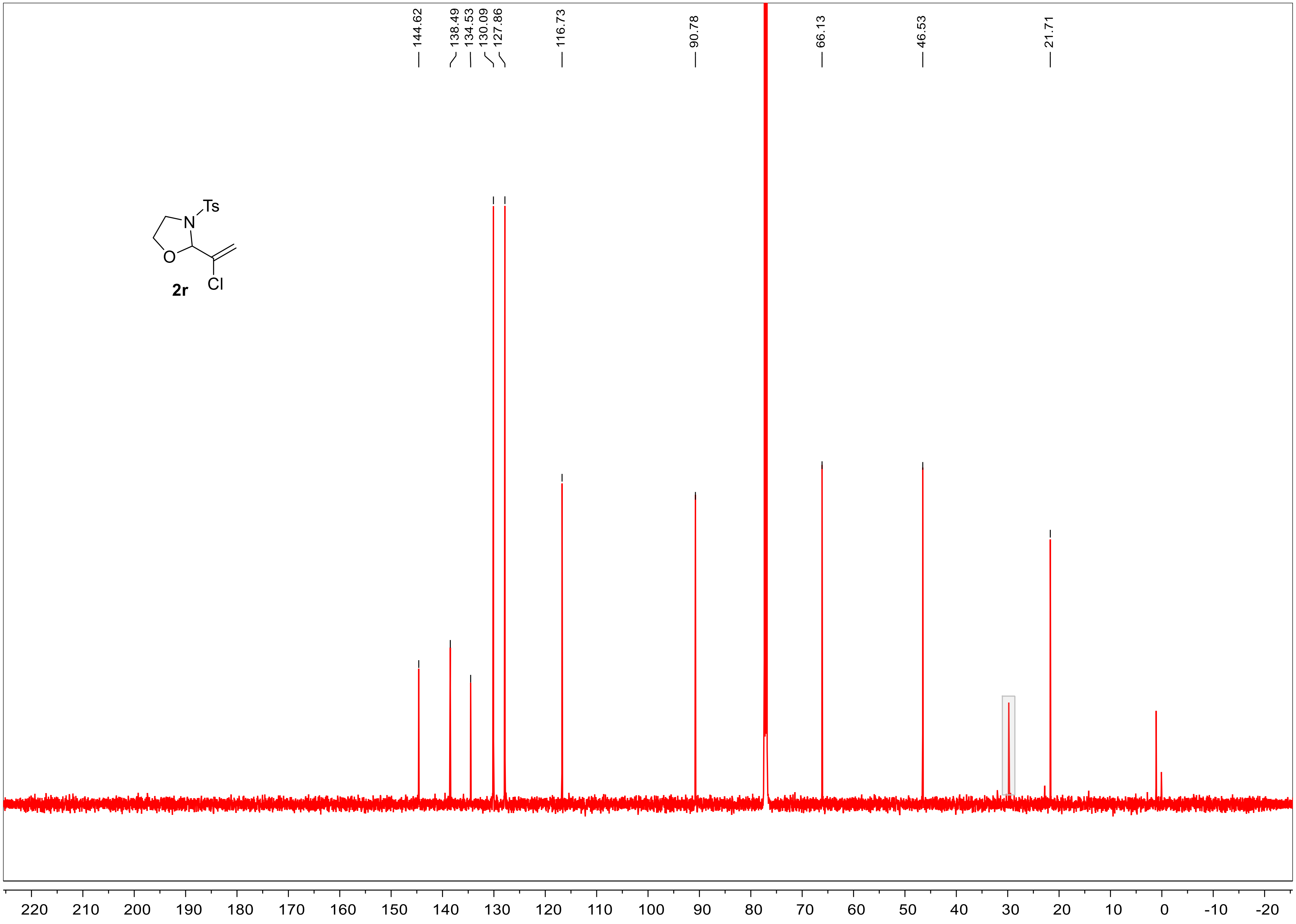

DEPT 135 (150 MHz, $CDCl_3$)

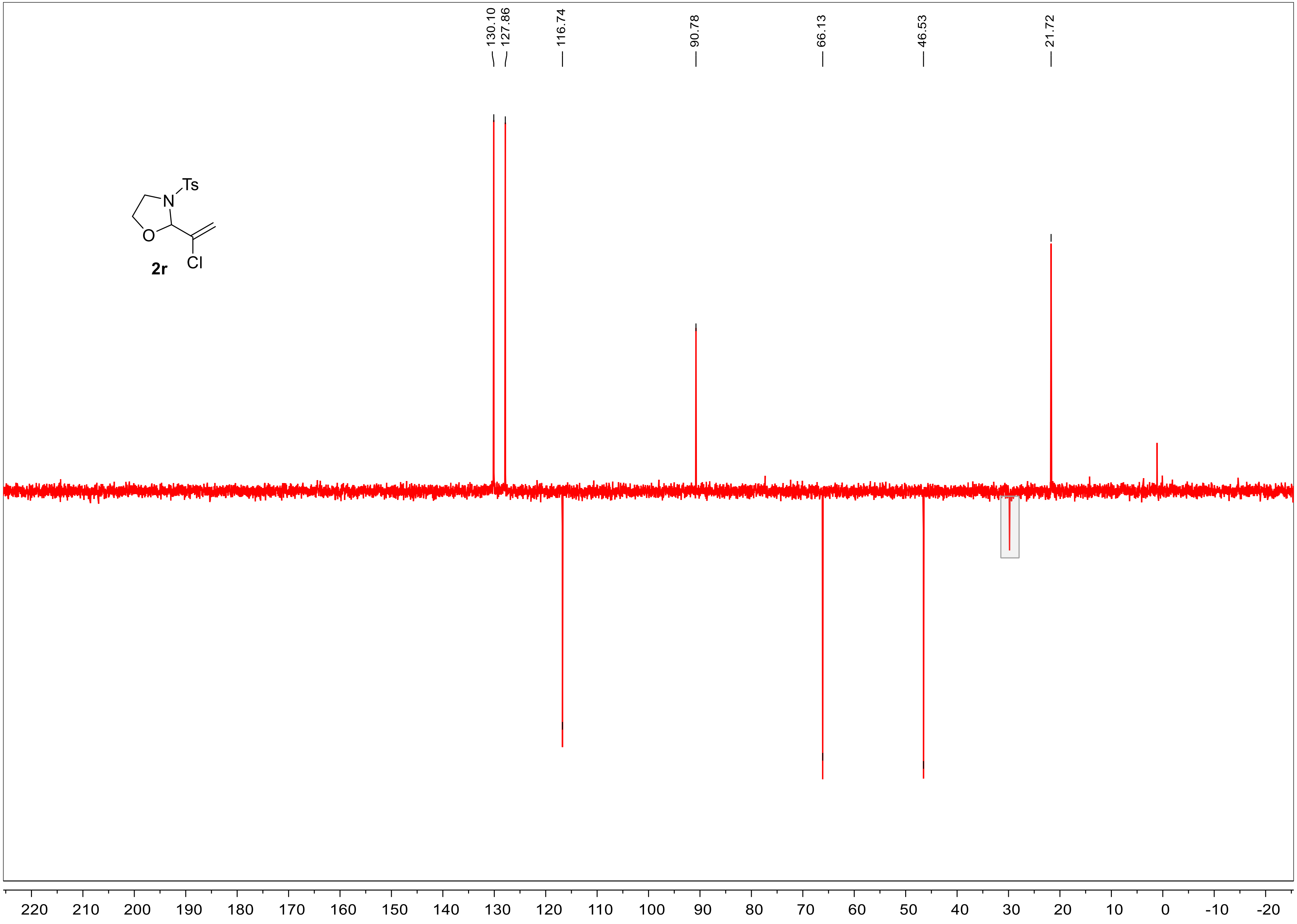

2r

^{1}H-NMR (600 MHz, $CDCl_3$)

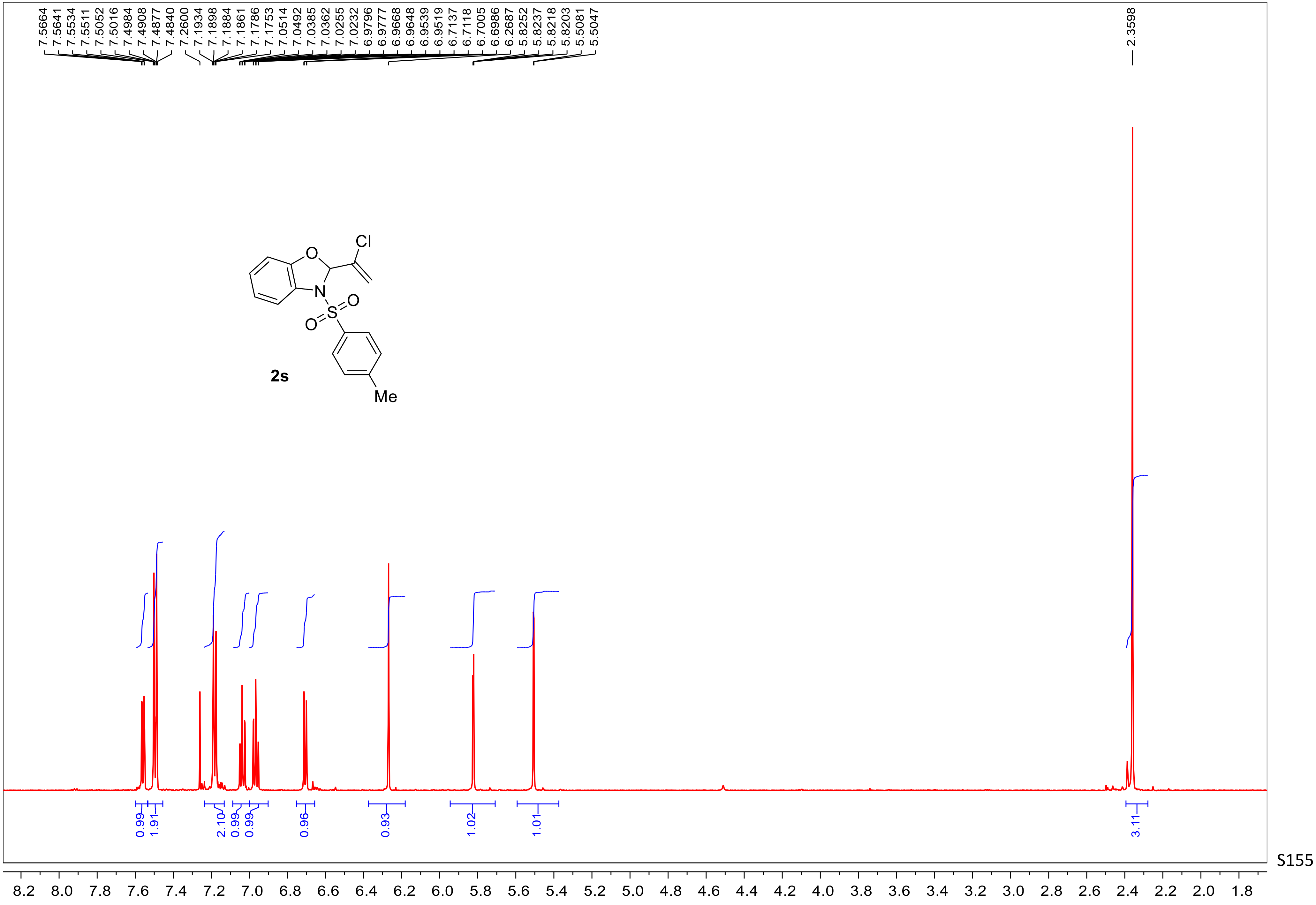

2D - COSY (600 MHz, $CDCl_3$)

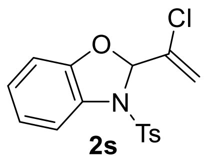

2s

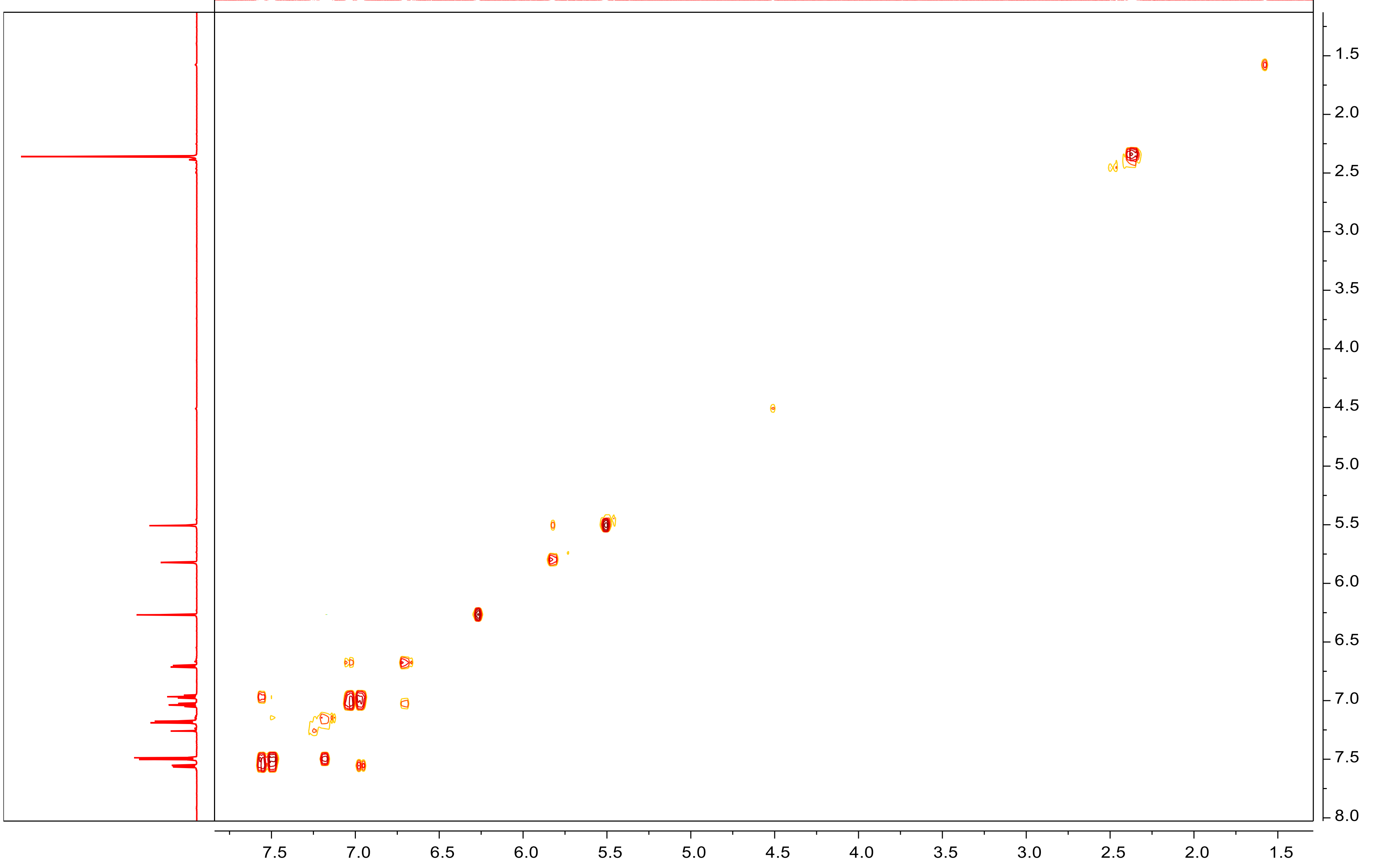

$^{13}C\{^{1}H\}$ NMR (150 MHz, $CDCl_3$)

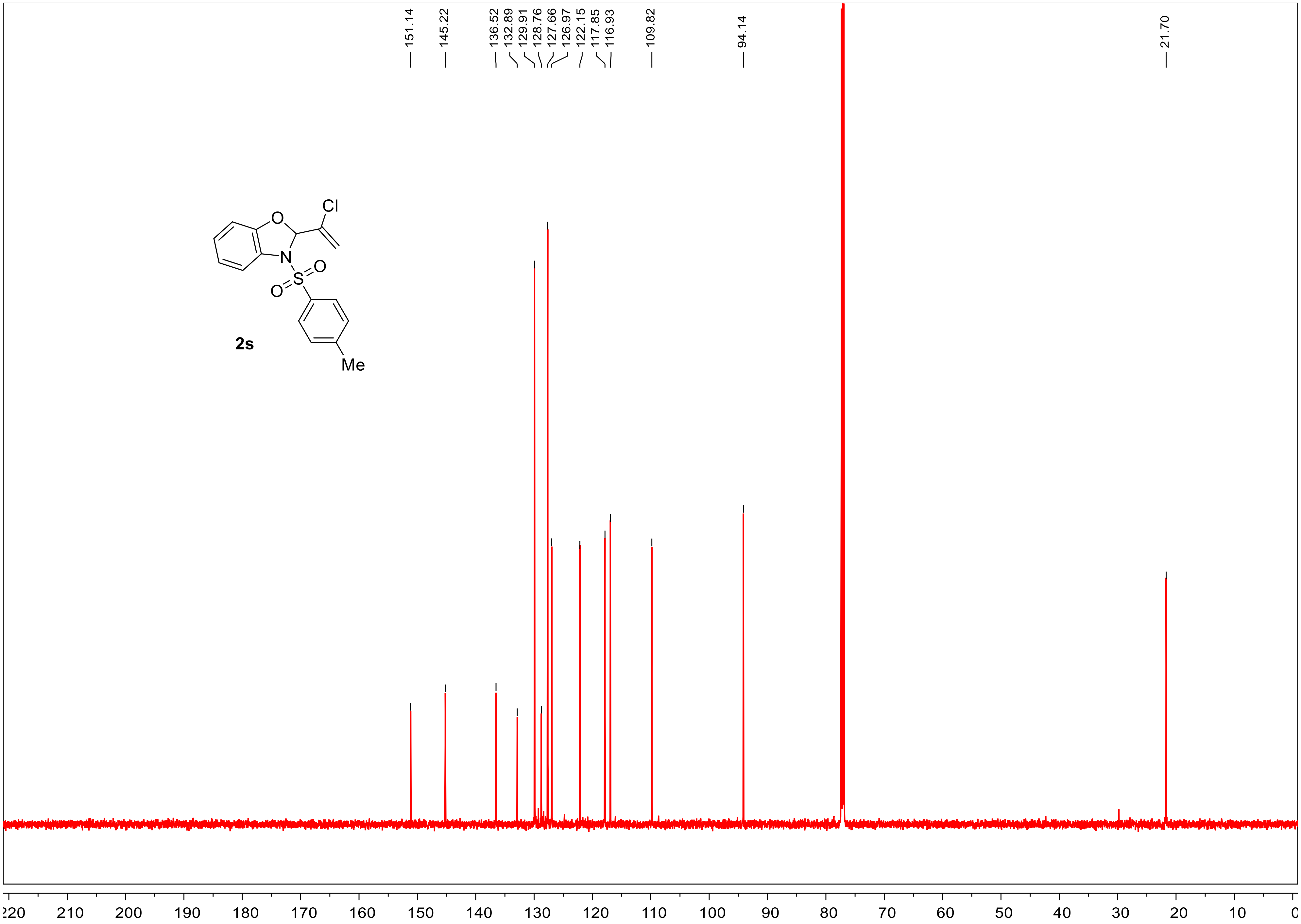

DEPT 135 (150 MHz, $CDCl_3$)

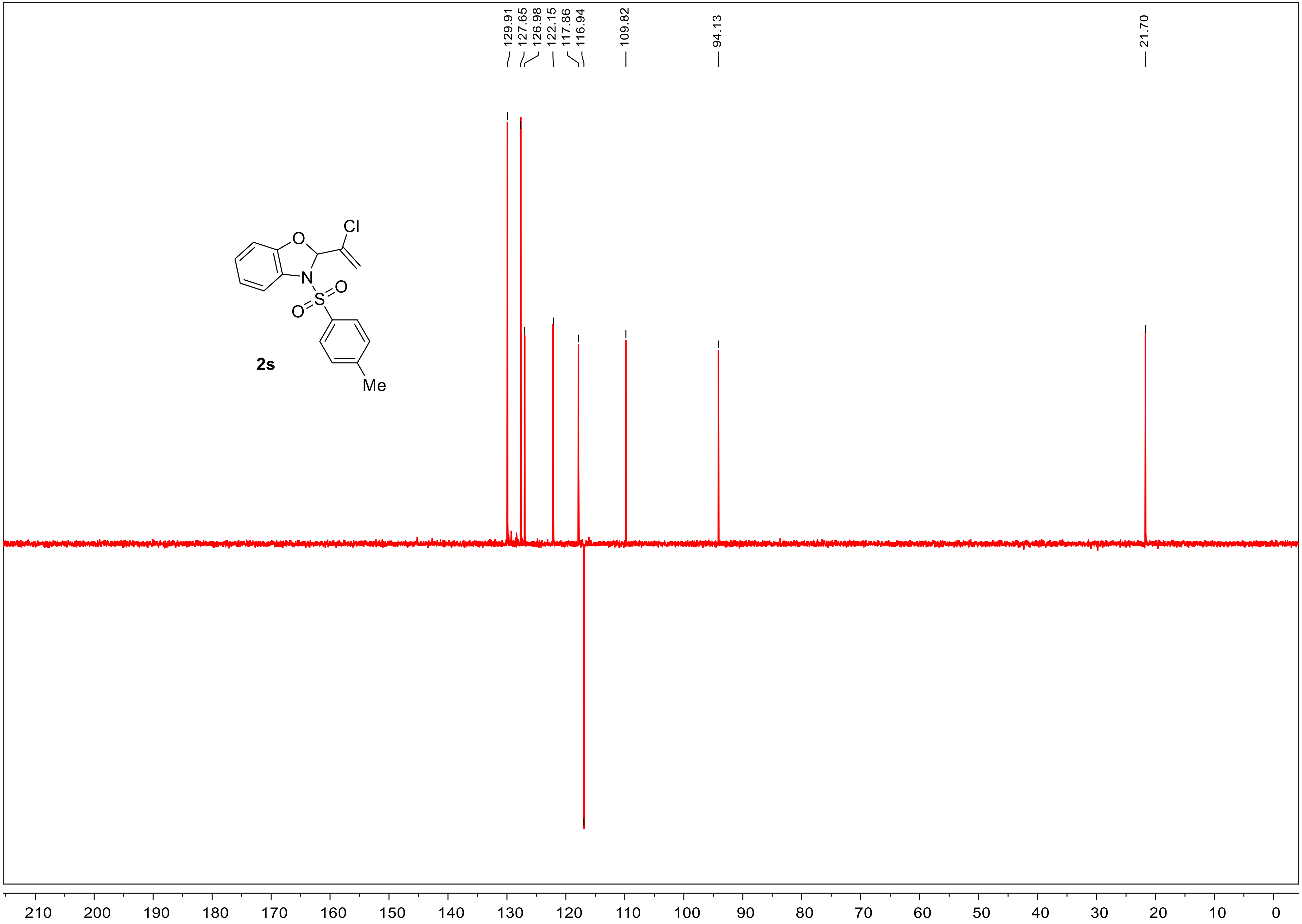

^{1}H-NMR (600 MHz, $CDCl_3$)

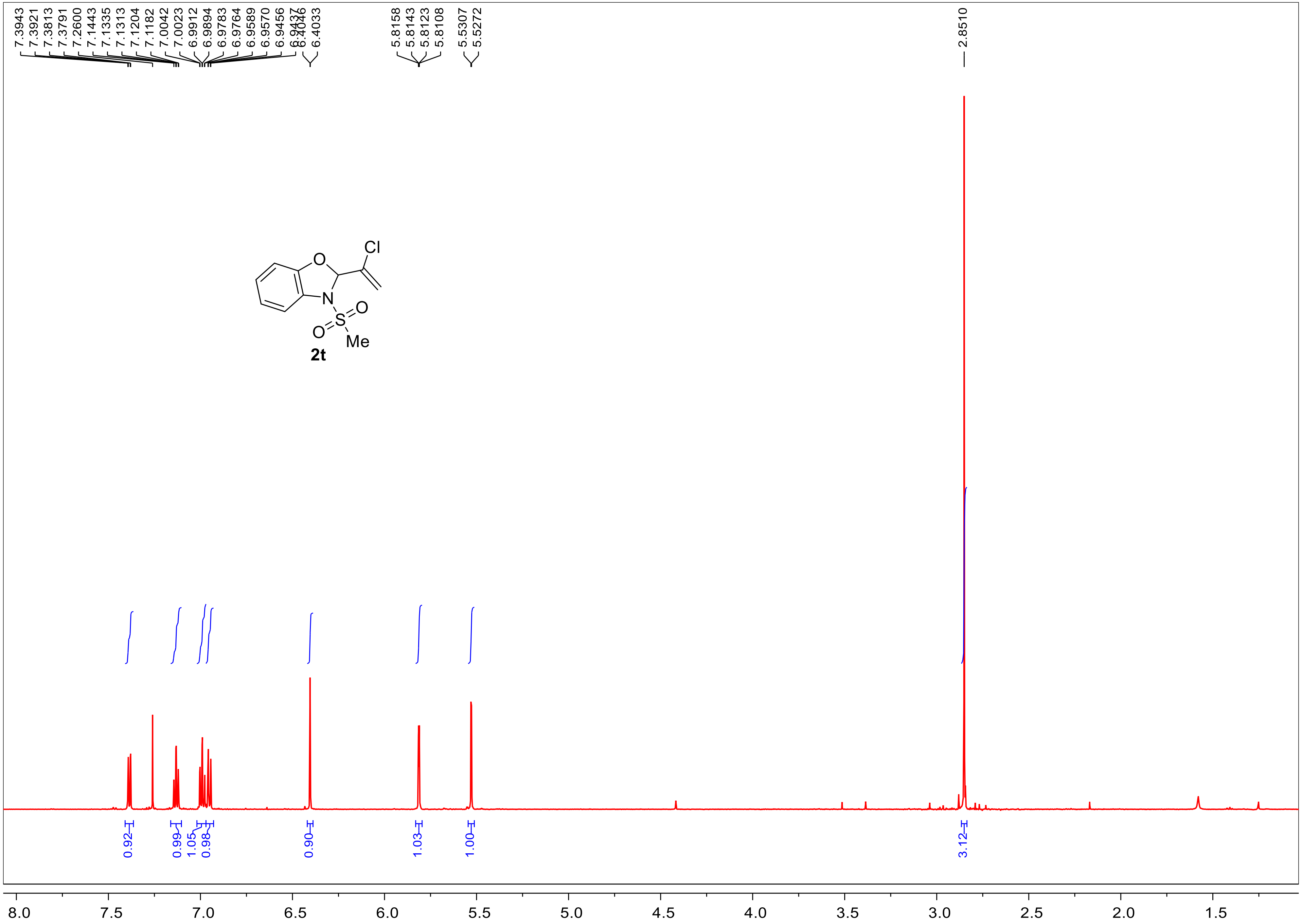

2D - COSY (600 MHz, $CDCl_3$)

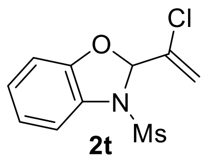

2t

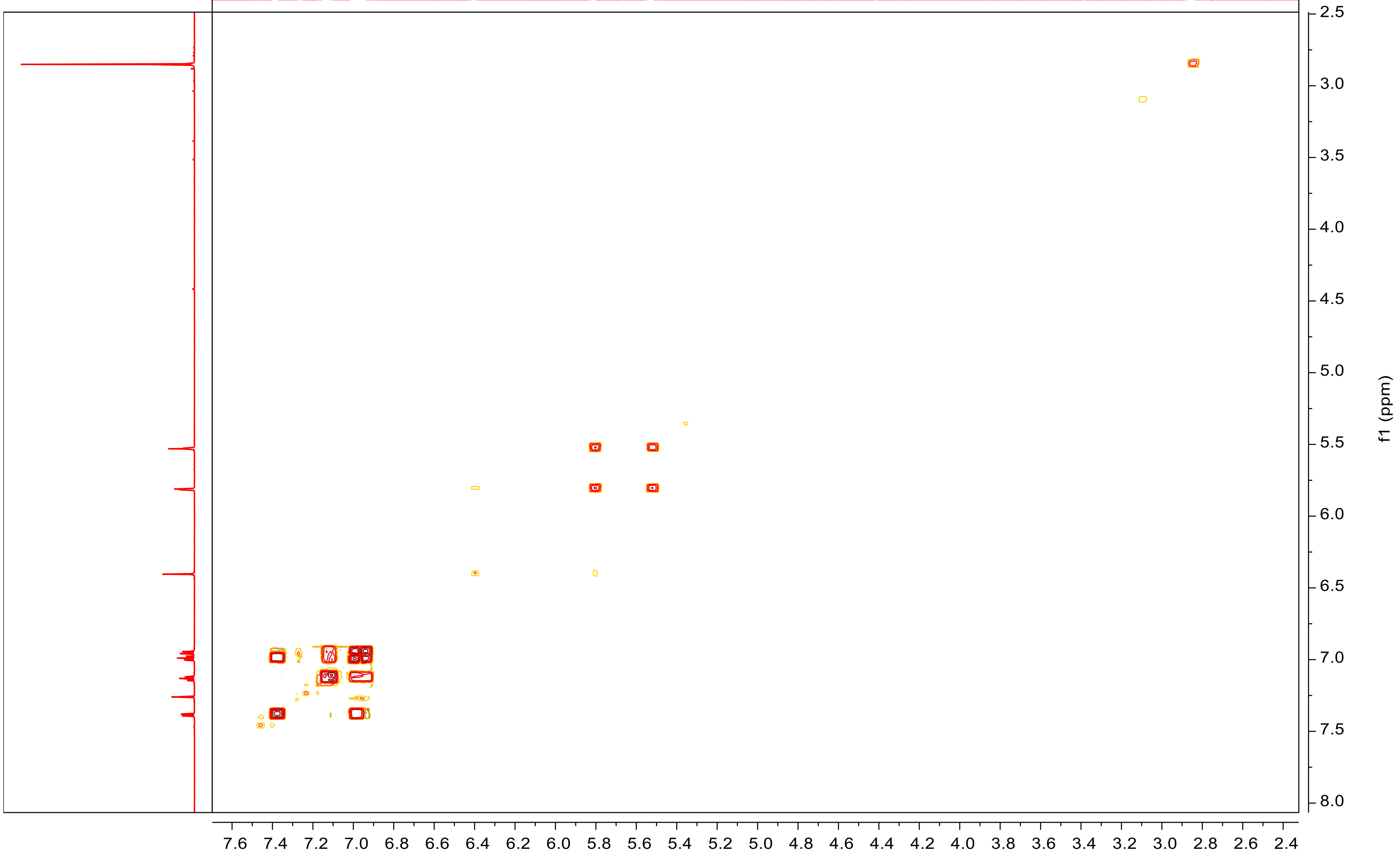

$^{13}C\{^{1}H\}$ **NMR** (150 MHz, $CDCl_3$)

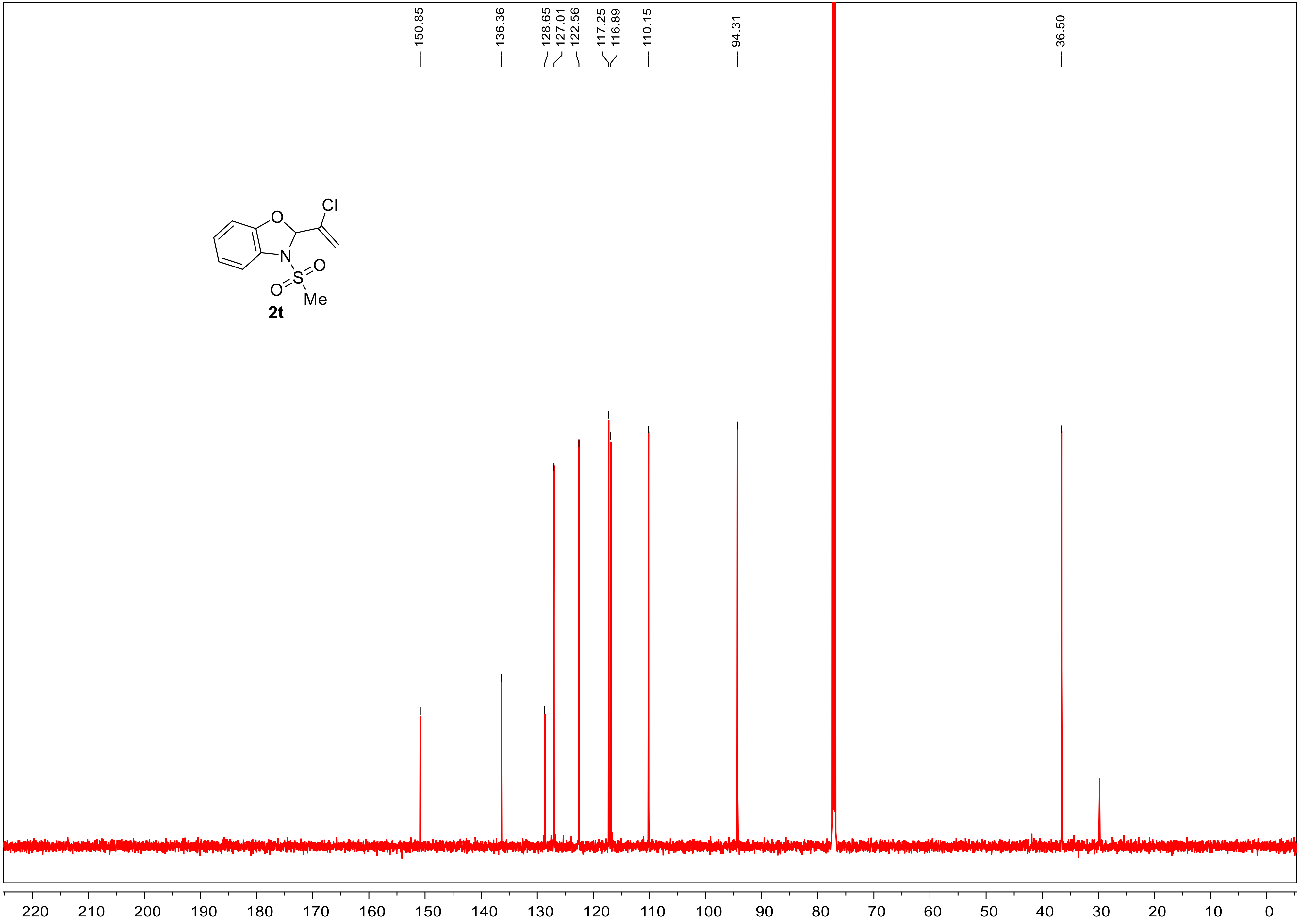

DEPT 135 (150 MHz, $CDCl_3$)

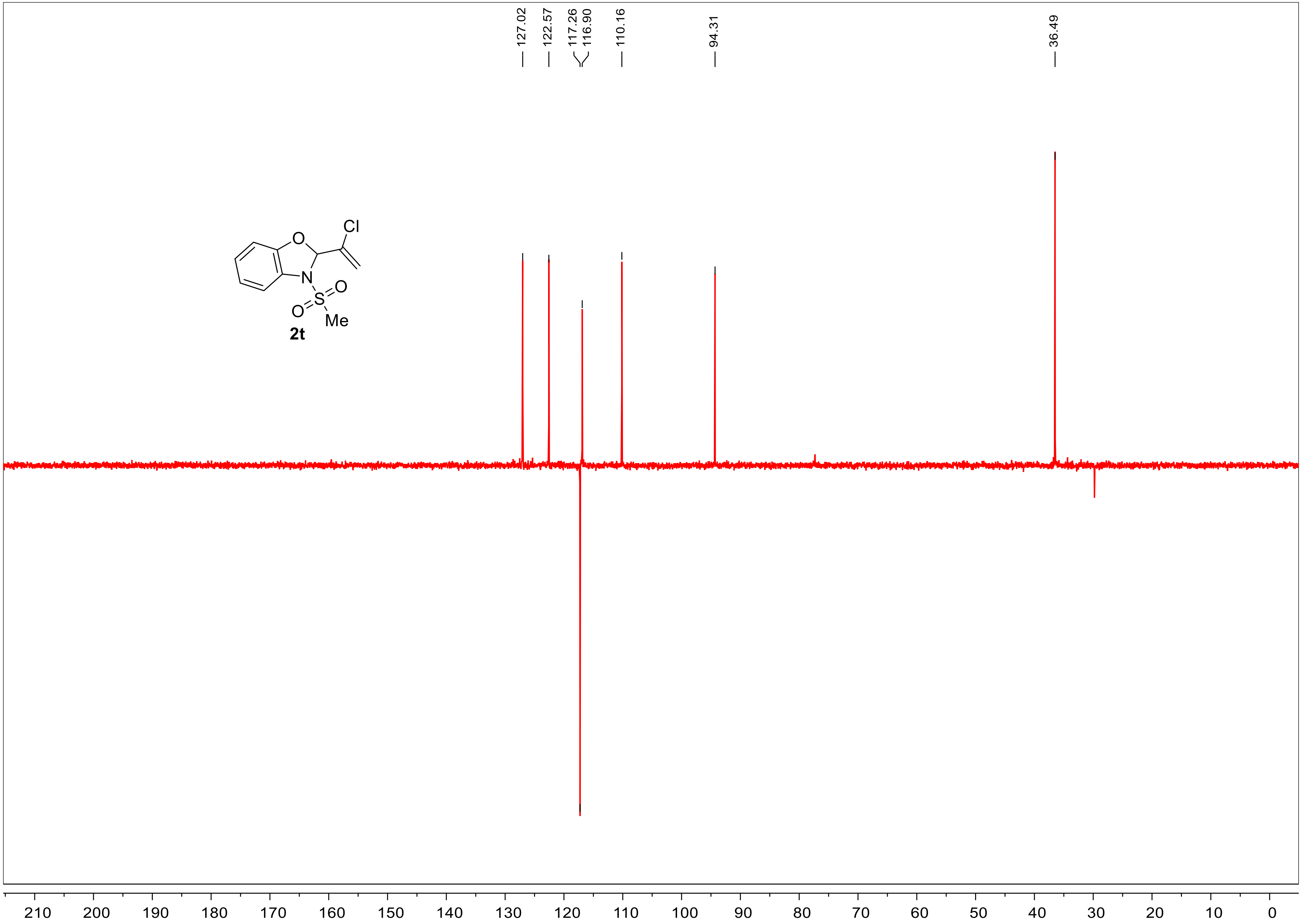

2t

^{1}H-NMR (600 MHz, $CDCl_3$)

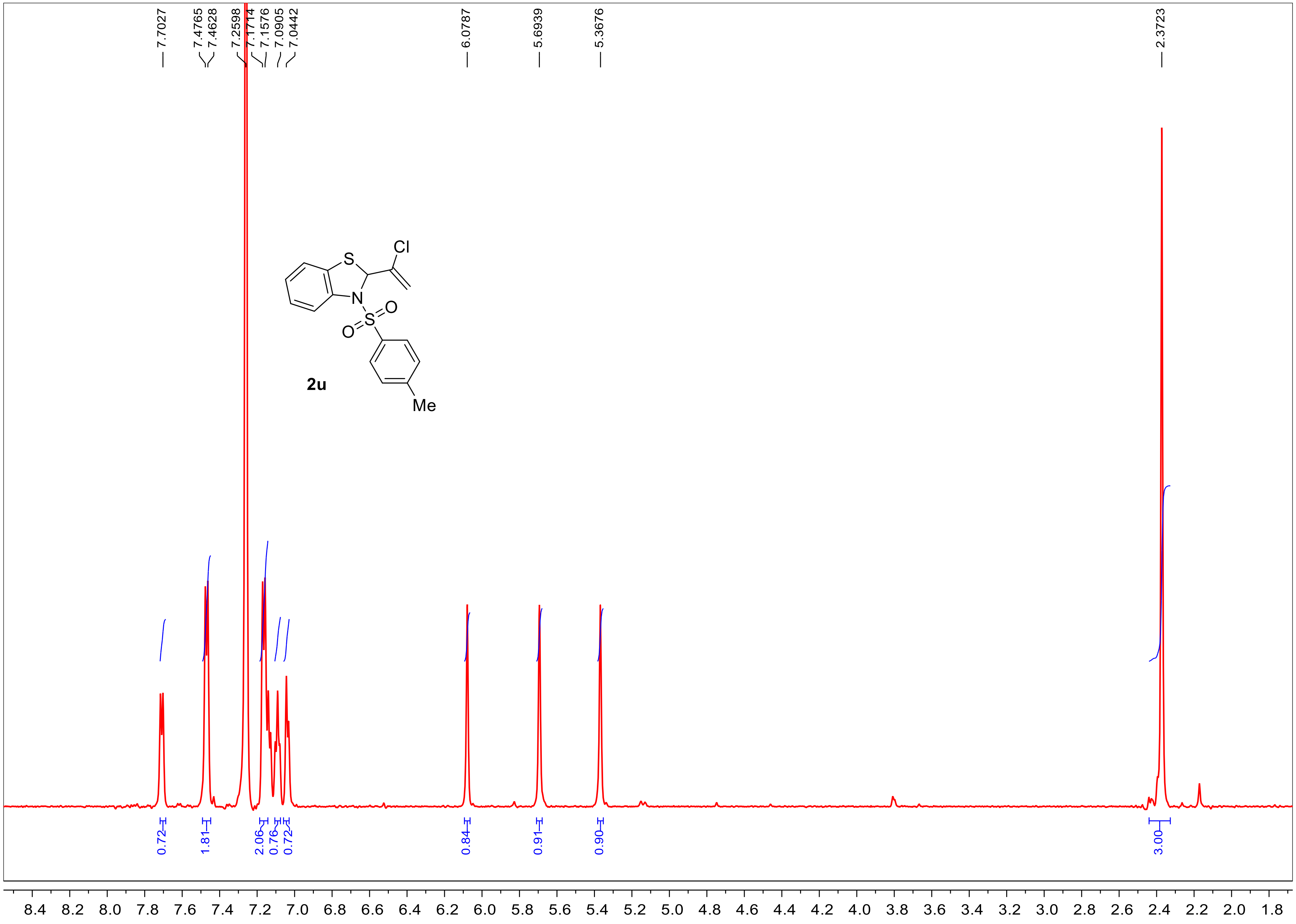

2D - COSY (600 MHz, $CDCl_3$)

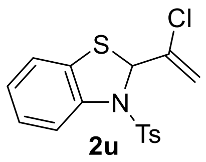

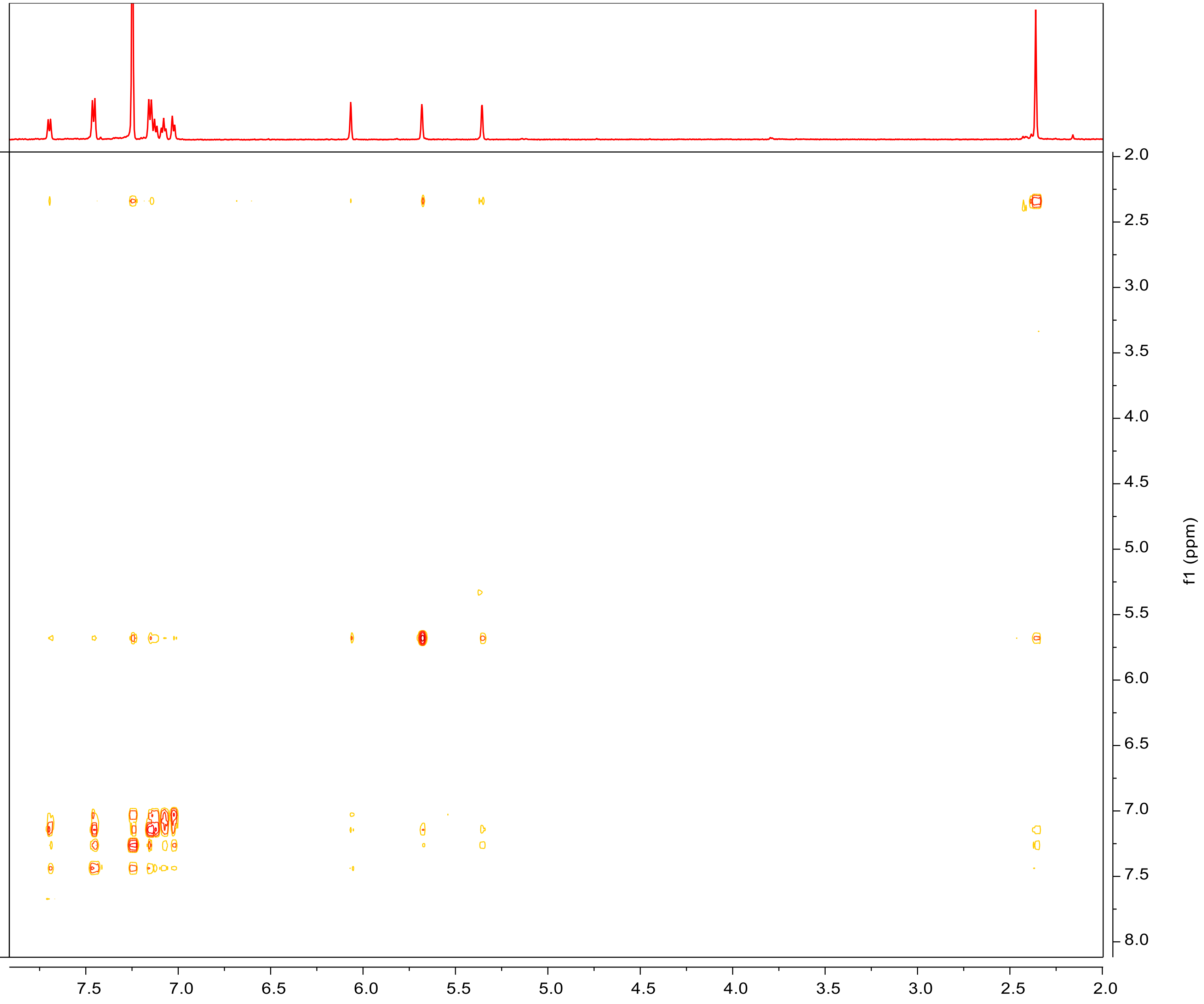

$^{13}C\{^1H\}$ NMR (150 MHz, $CDCl_3$)

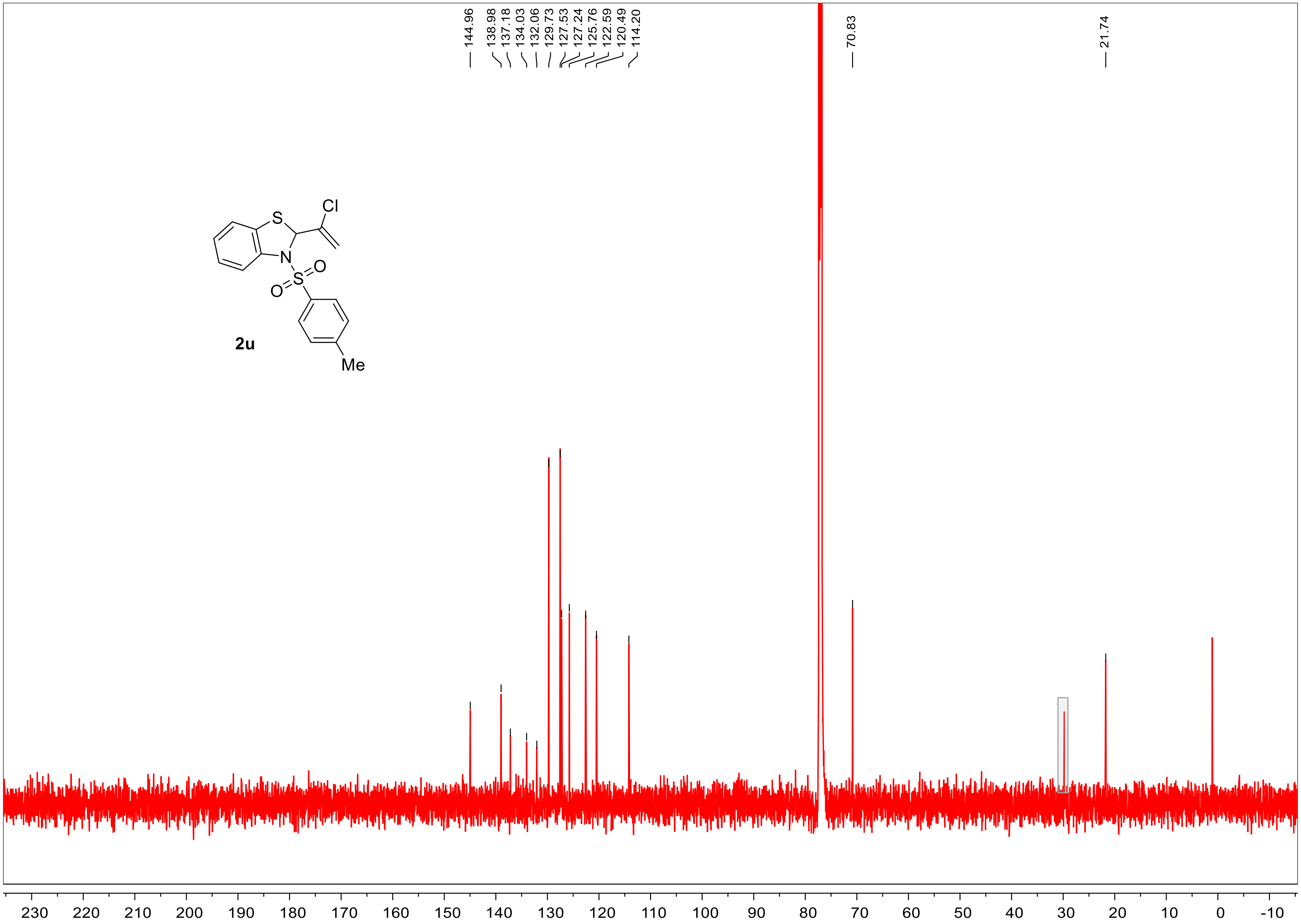

DEPT 135 (150 MHz, $CDCl_3$)

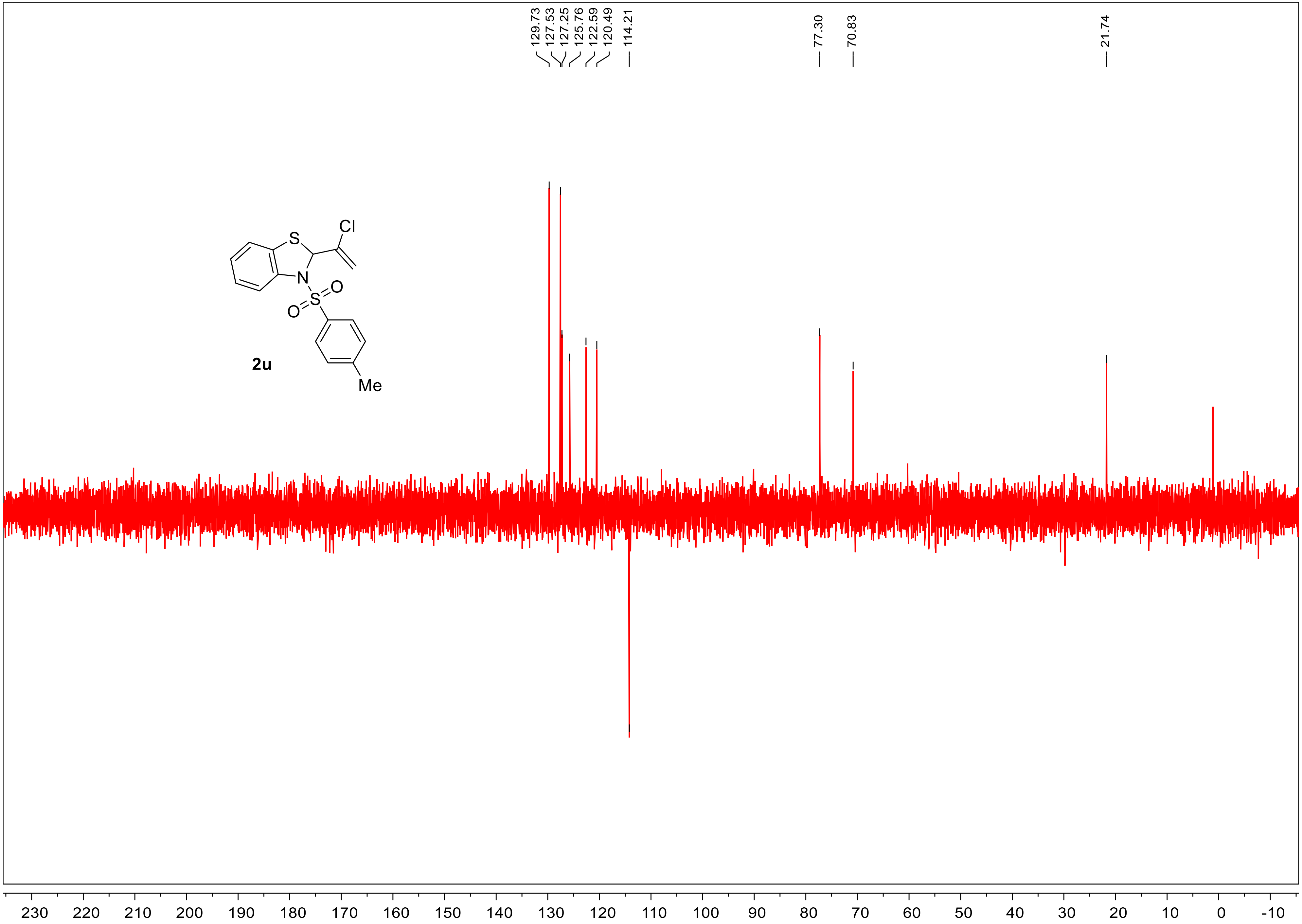

^{1}H-NMR (600 MHz, $CDCl_3$)

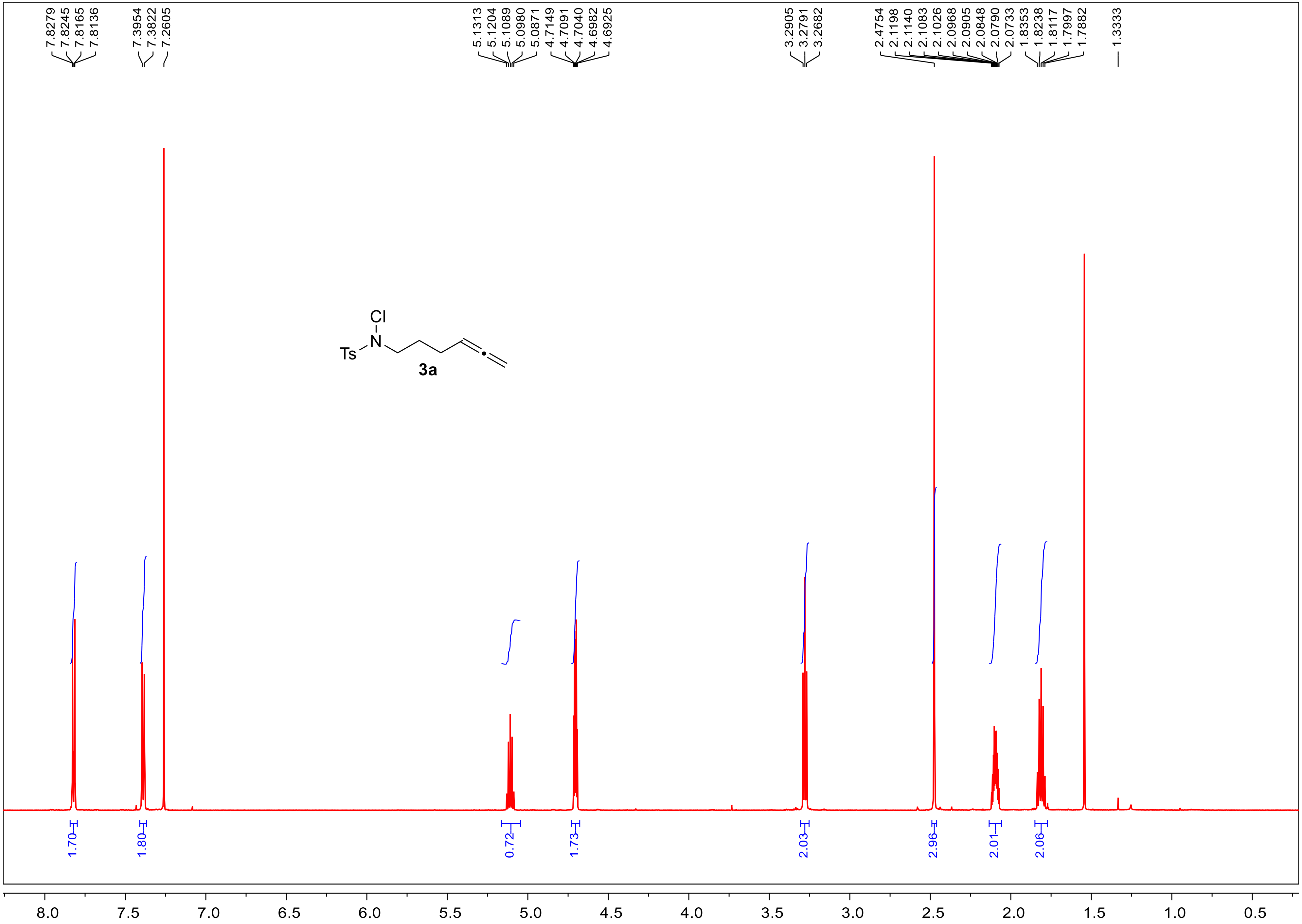

2D - COSY (600 MHz, $CDCl_3$)

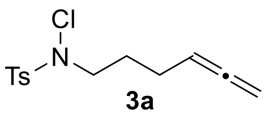

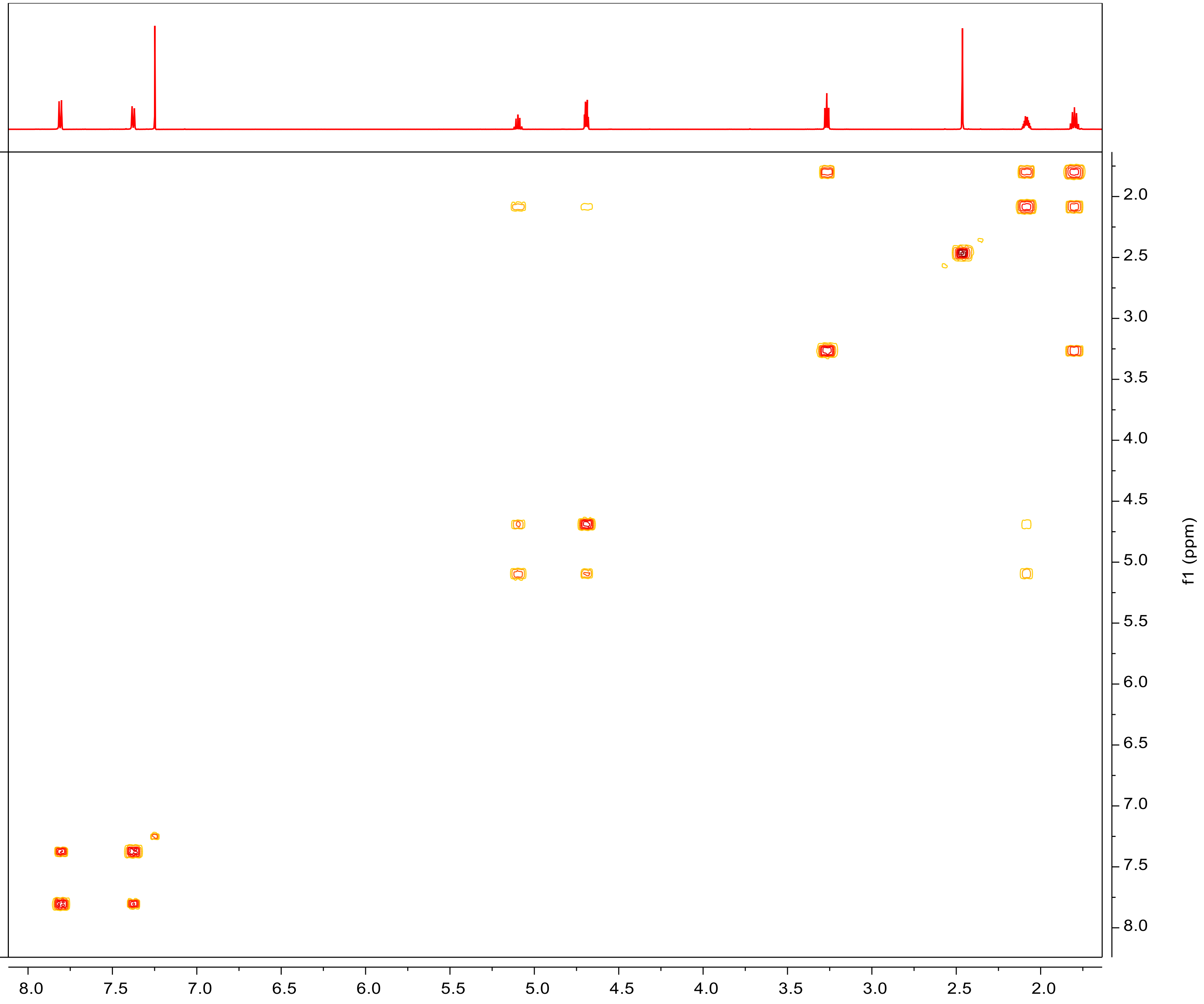

$^{13}C\{^1H\}$ **NMR** (150 MHz, $CDCl_3$)

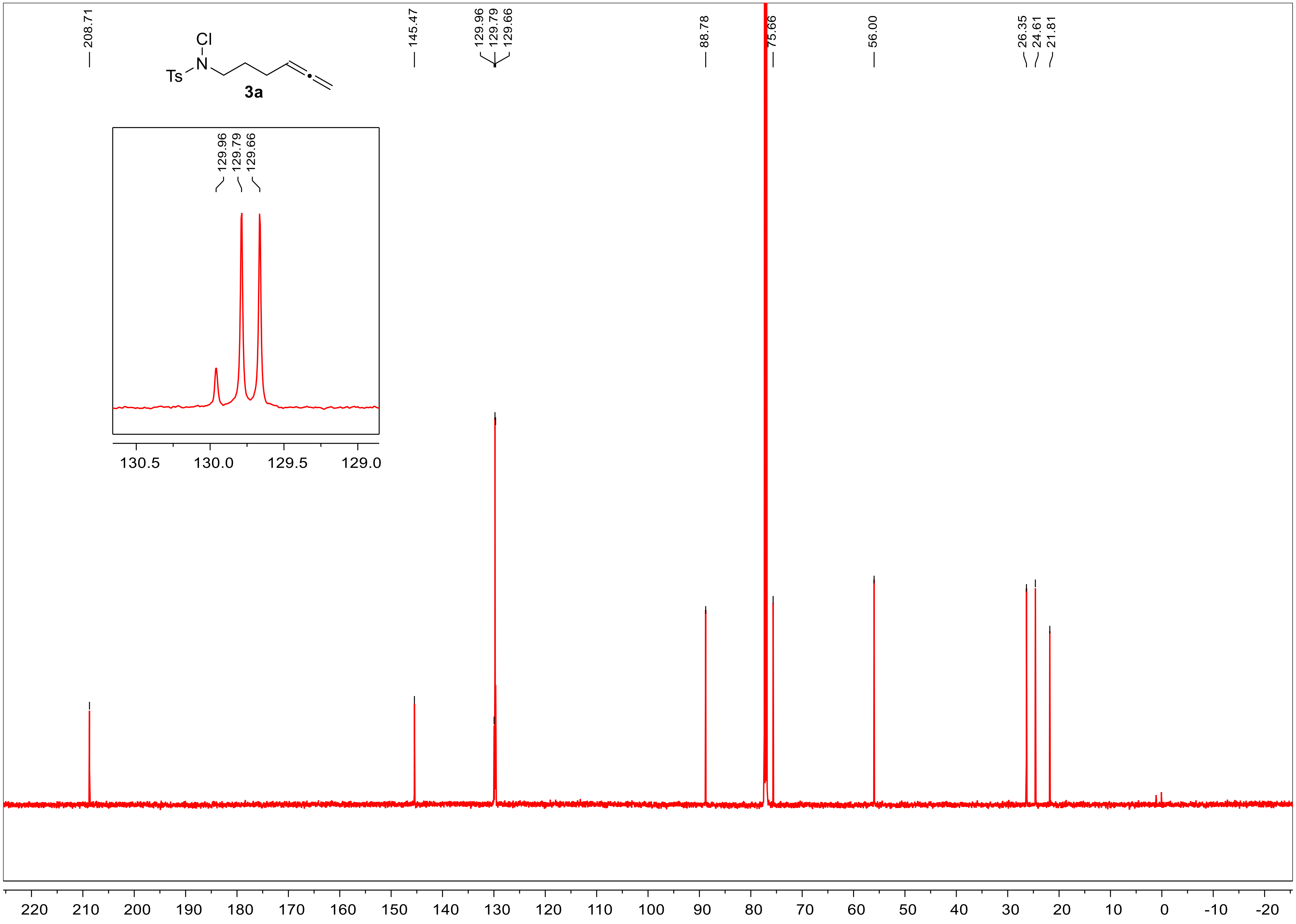

DEPT 135 (150 MHz, $CDCl_3$)

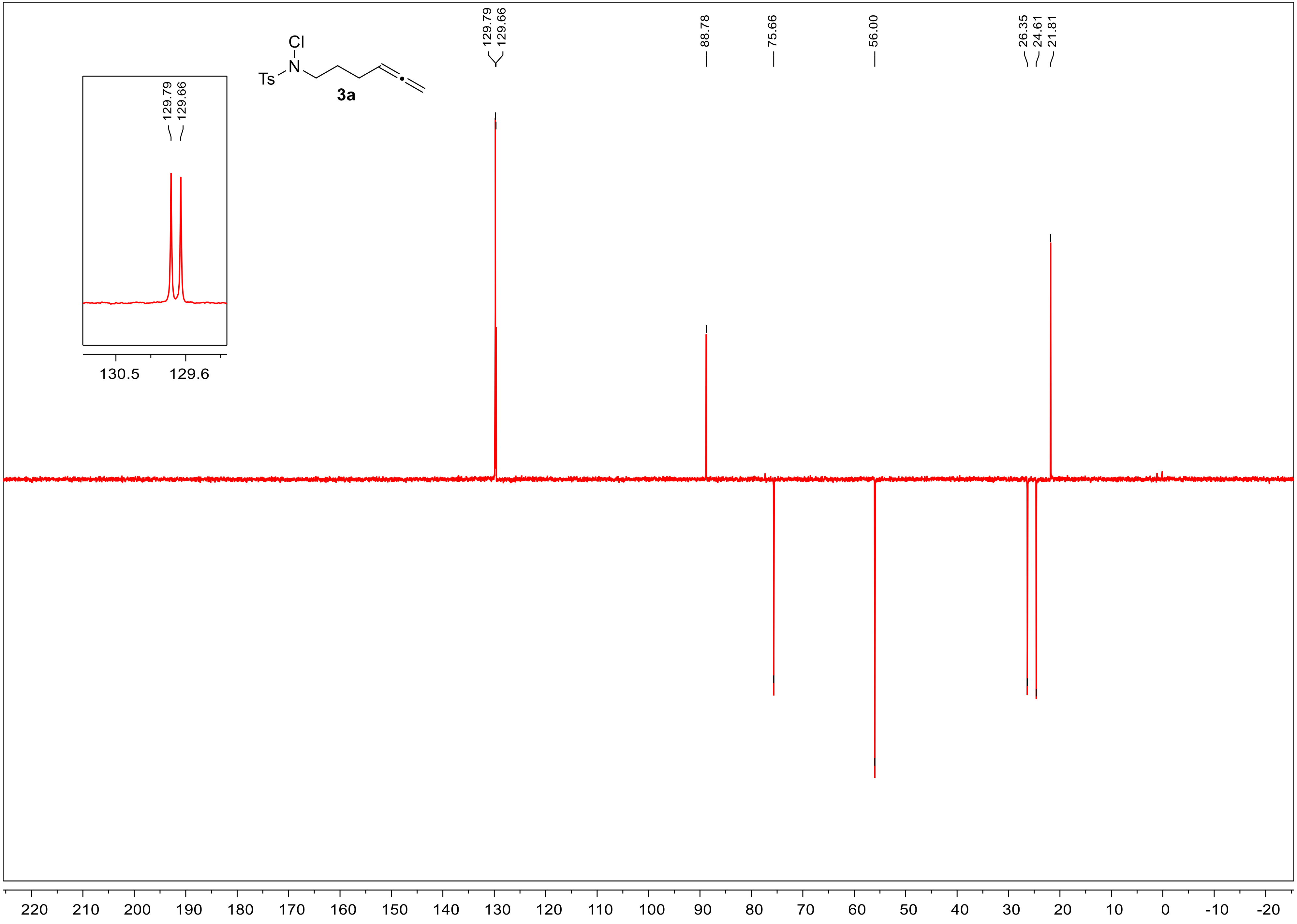

¹H-NMR (600 MHz, $CDCl_3$)

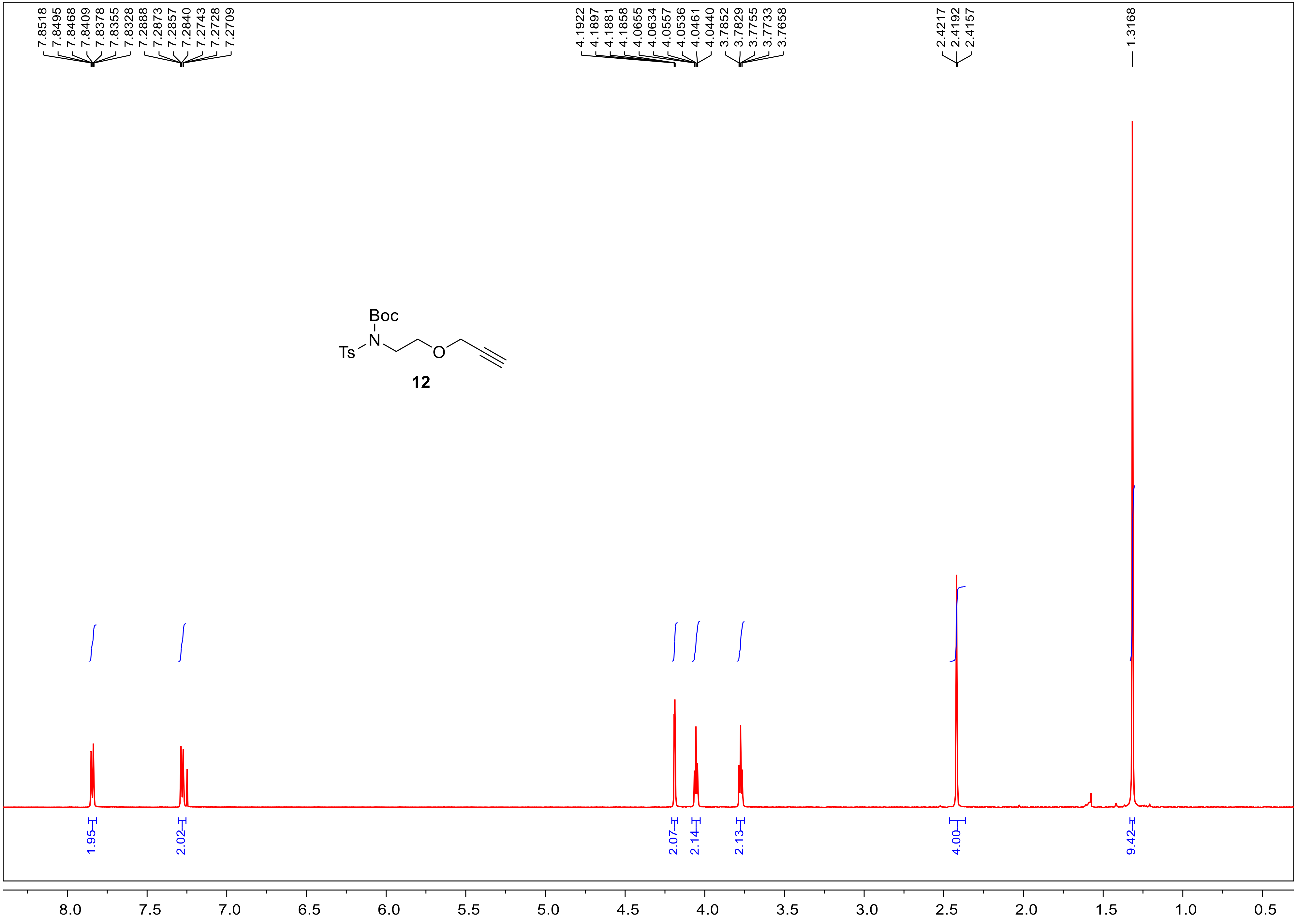

2D - COSY (600 MHz, $CDCl_3$)

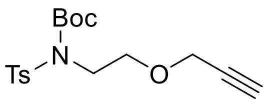

12

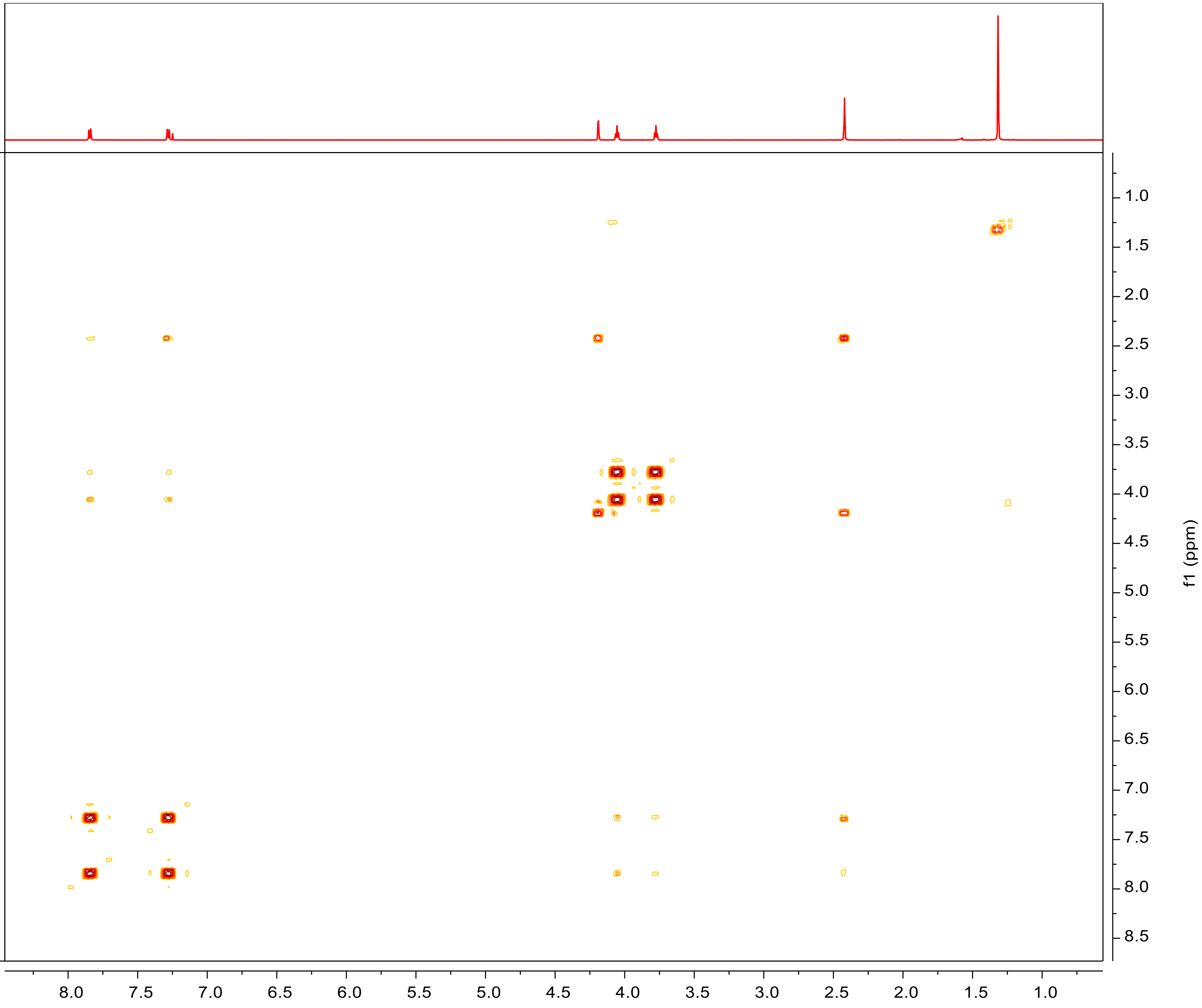

13C{1H} NMR (150 MHz, $CDCl_3$)

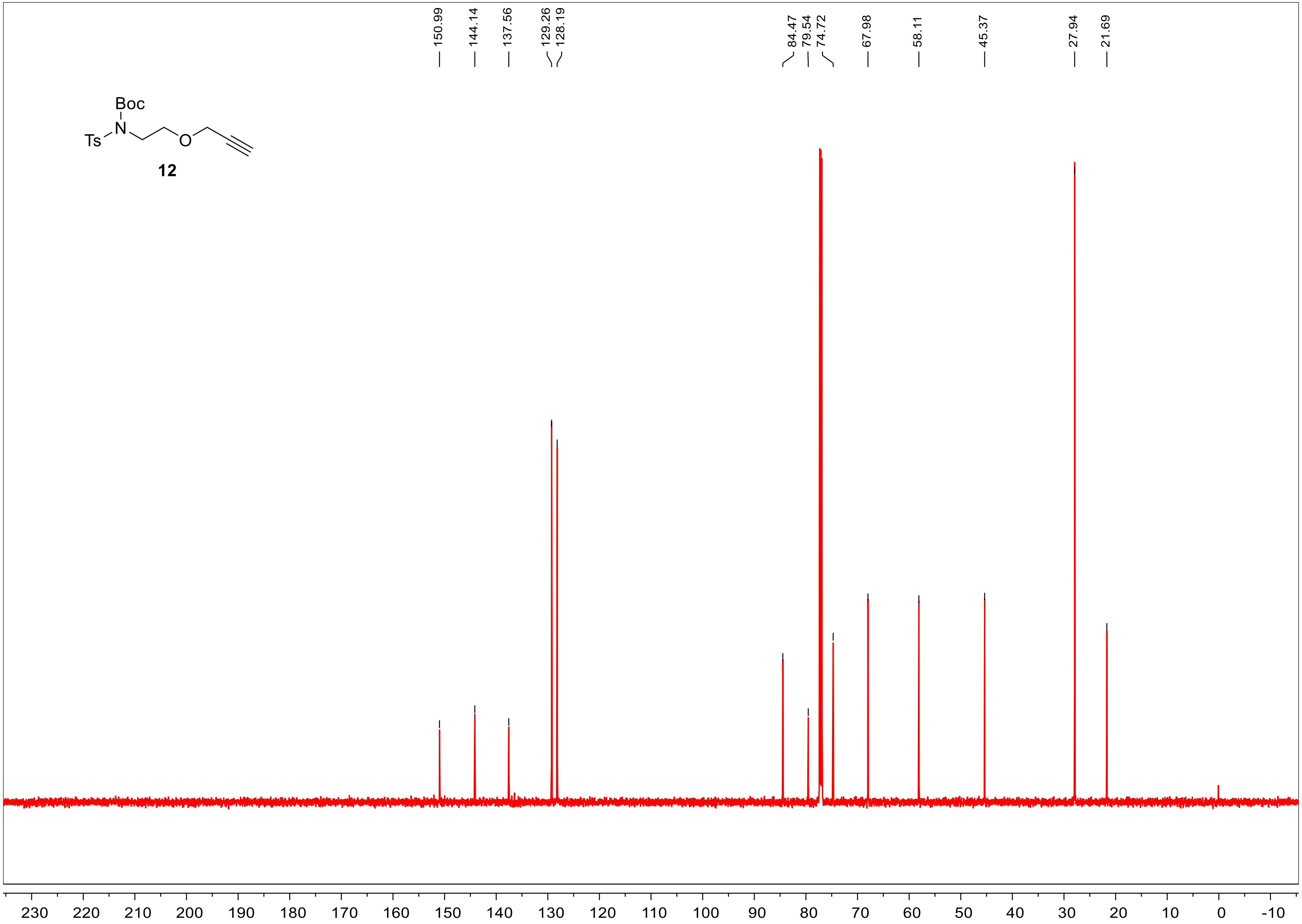

DEPT 135 (150 MHz, $CDCl_3$)

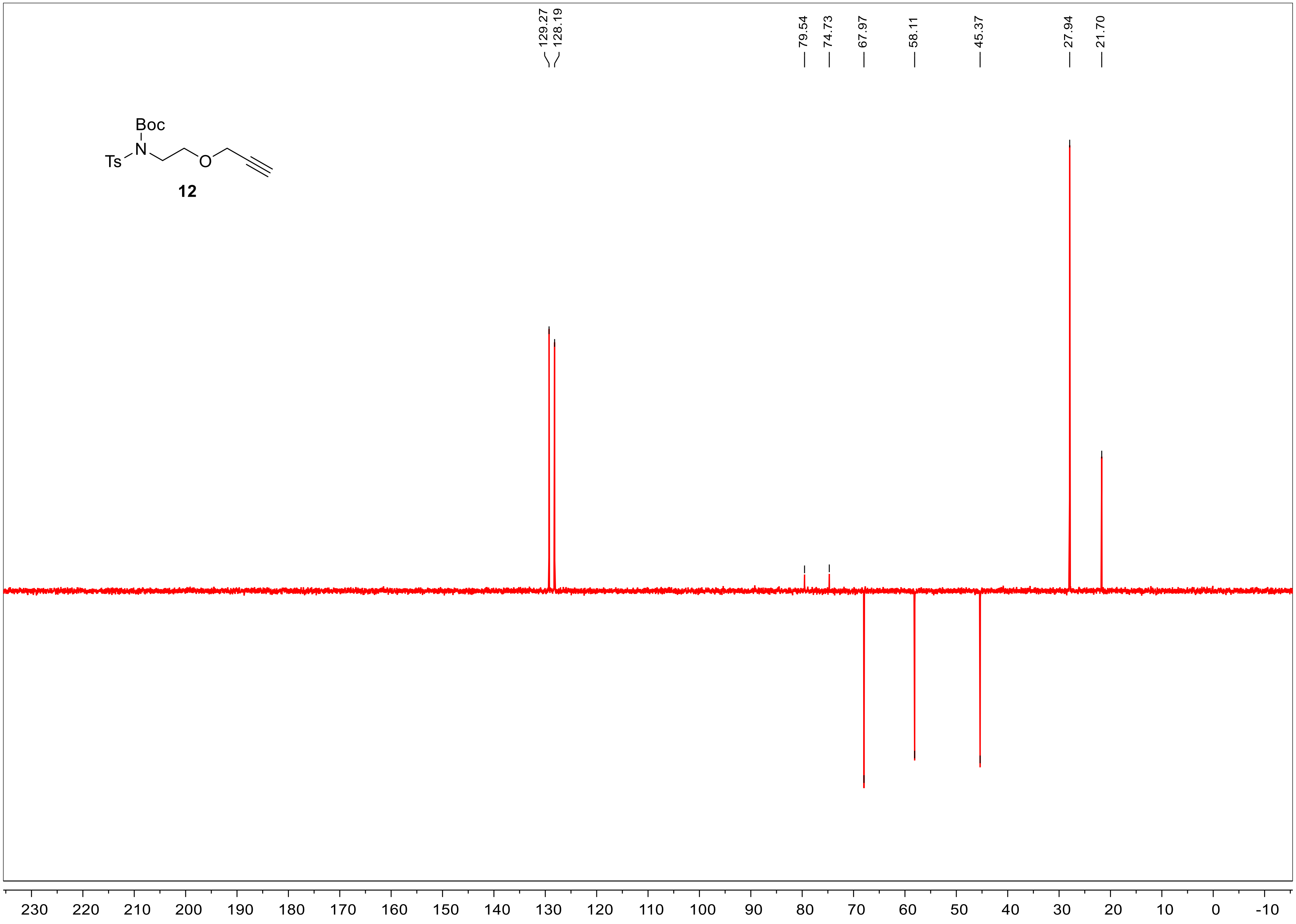

^{1}H-NMR (600 MHz, $CDCl_3$)

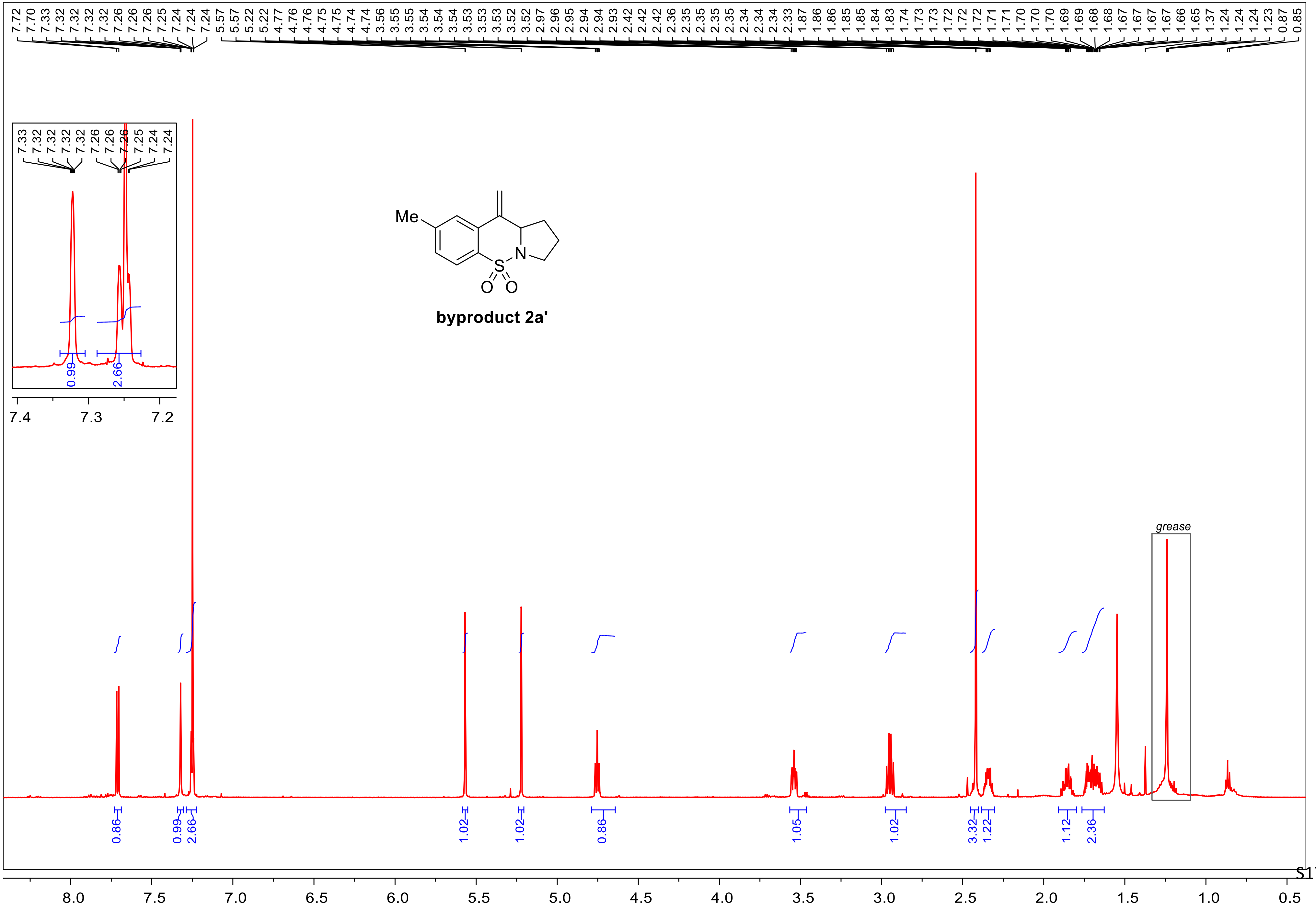

2D - COSY (600 MHz, $CDCl_3$)

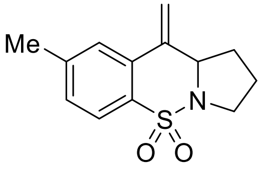

byproduct 2a'

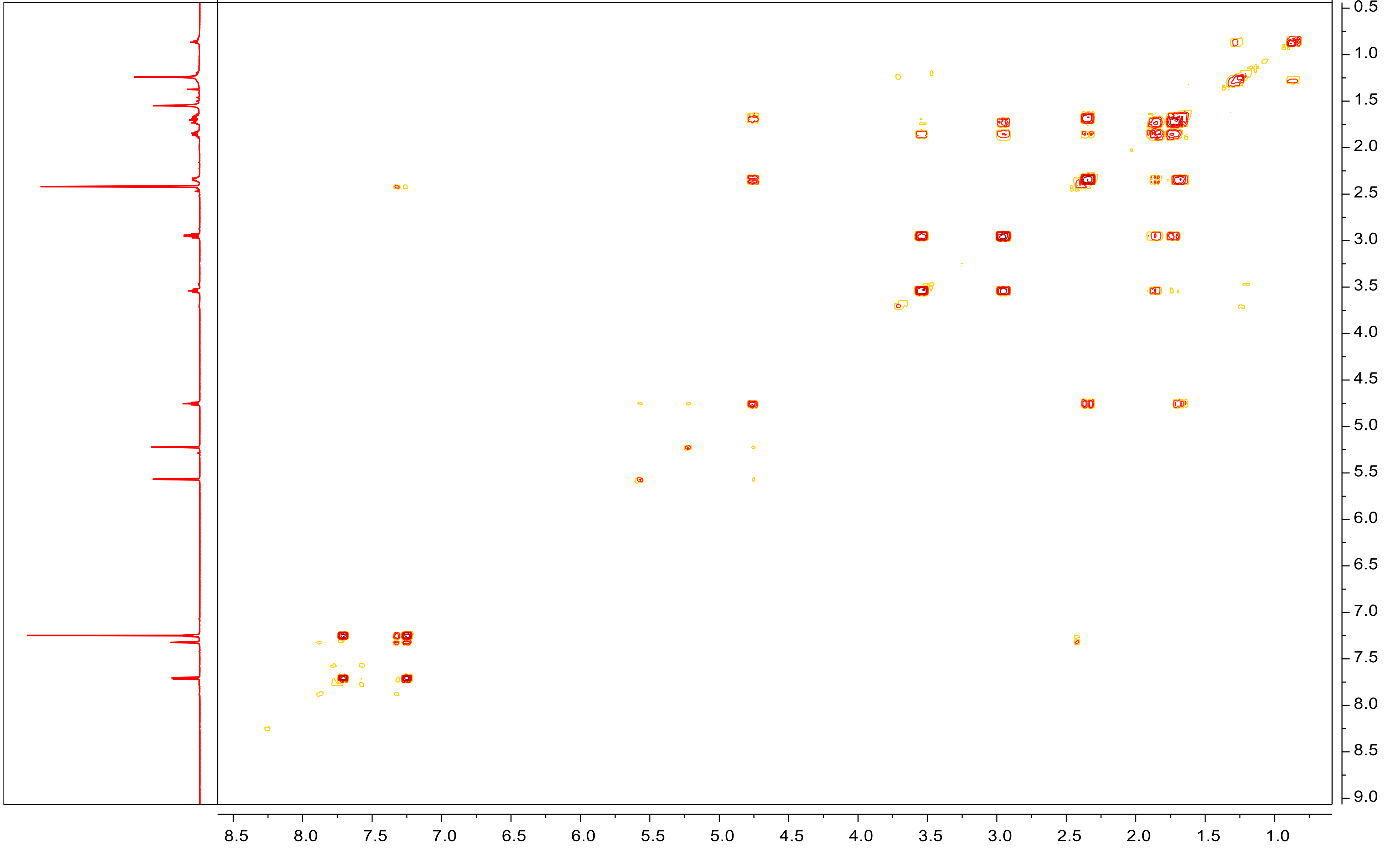

$^{13}C\{^1H\}$ NMR (150 MHz, $CDCl_3$)

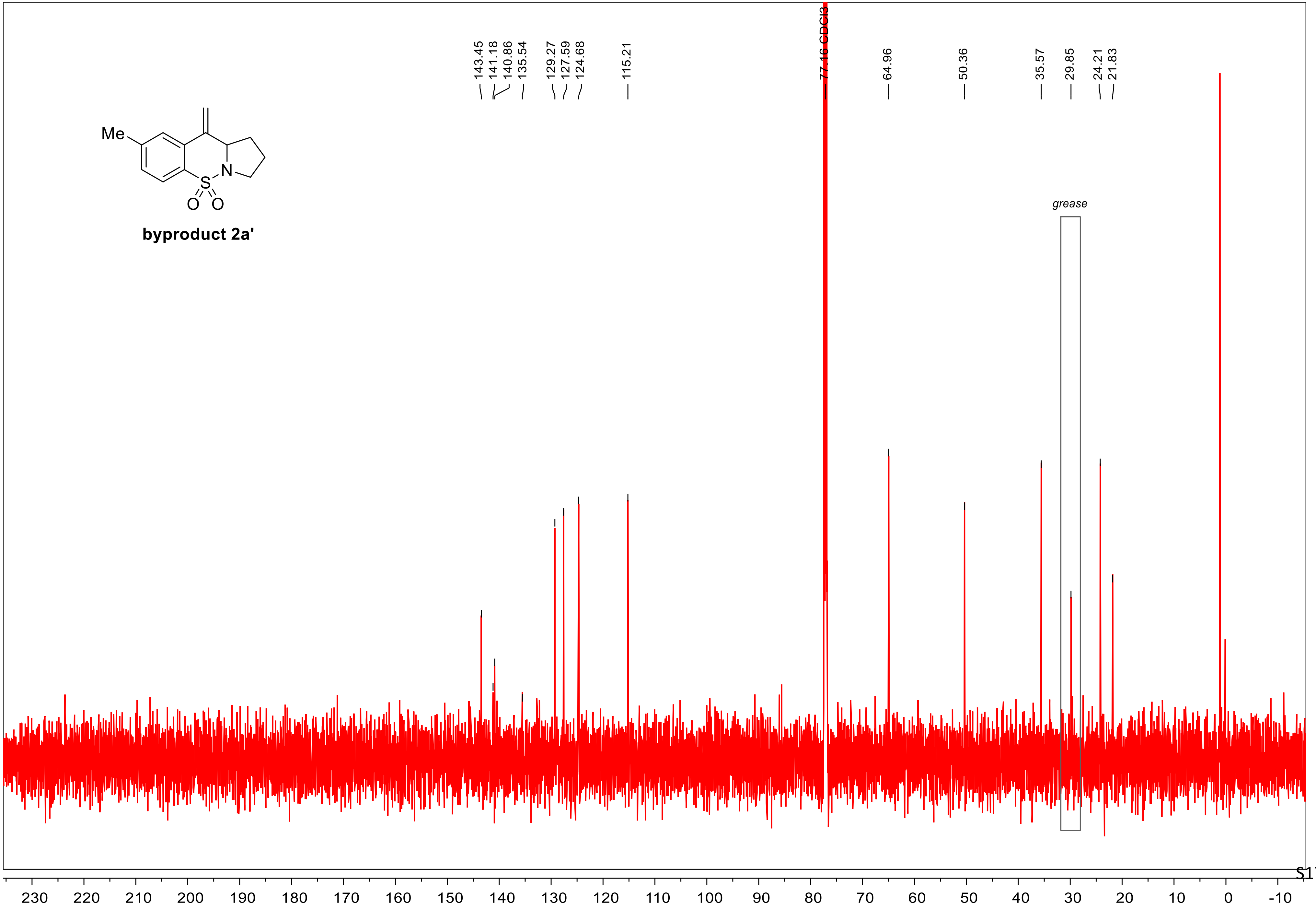

DEPT 135 (150 MHz, $CDCl_3$)

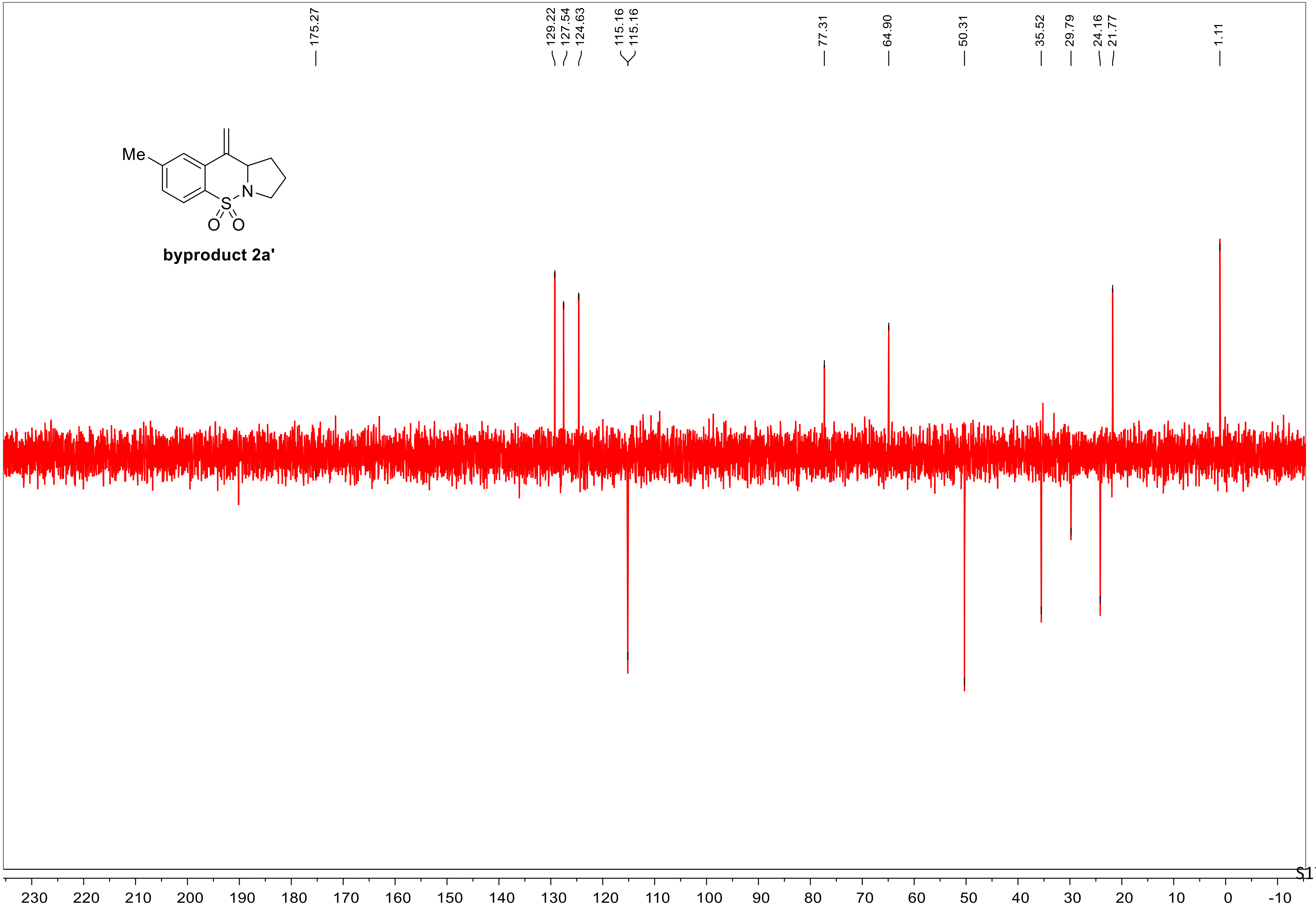

Supplement: Supplementary file 2 — jo2c01963_si_002.pdf [file jo2c01963_si_002.pdf]
